# High Throughput Phenotypic Analysis of *Mycobacterium tuberculosis* and *Mycobacterium bovis* Strains' Metabolism Using Biolog Phenotype Microarrays

**DOI:** 10.1371/journal.pone.0052673

**Published:** 2013-01-10

**Authors:** Bhagwati Khatri, Mark Fielder, Gareth Jones, William Newell, Manal Abu-Oun, Paul R. Wheeler

**Affiliations:** 1 Animal Health Veterinary Laboratories (Weybridge), Department for Bovine Tuberculosis, New Haw, Surrey, United Kingdom; 2 School of Life Sciences, Kingston University London, Penrhyn Road, Kingston upon Thames, Surrey, United Kingdom; 3 Animal Health Veterinary Laboratories (Weybridge), Specialist Scientific Services Department, New Haw, Surrey, United Kingdom; 4 Animal Health Veterinary Laboratories (Weybridge), Department of Bacteriology, New Haw, Surrey, United Kingdom; University of Padova, Medical School, Italy

## Abstract

Tuberculosis is a major human and animal disease of major importance worldwide. Genetically, the closely related strains within the *Mycobacterium tuberculosis* complex which cause disease are well-characterized but there is an urgent need better to understand their phenotypes. To search rapidly for metabolic differences, a working method using Biolog Phenotype MicroArray analysis was developed. Of 380 substrates surveyed, 71 permitted tetrazolium dye reduction, the readout over 7 days in the method. By looking for ≥5-fold differences in dye reduction, 12 substrates differentiated *M. tuberculosis* H37Rv and *Mycobacterium bovis* AF2122/97. H37Rv and a Beijing strain of *M. tuberculosis* could also be distinguished in this way, as could field strains of *M. bovis*; even pairs of strains within one spoligotype could be distinguished by 2 to 3 substrates. Cluster analysis gave three clear groups: H37Rv, Beijing, and all the *M. bovis* strains. The substrates used agreed well with prior knowledge, though an unexpected finding that AF2122/97 gave greater dye reduction than H37Rv with hexoses was investigated further, in culture flasks, revealing that hexoses and Tween 80 were synergistic for growth and used simultaneously rather than in a diauxic fashion. Potential new substrates for growth media were revealed, too, most promisingly N-acetyl glucosamine. Osmotic and pH arrays divided the mycobacteria into two groups with different salt tolerance, though in contrast to the substrate arrays the groups did not entirely correlate with taxonomic differences. More interestingly, these arrays suggested differences between the amines used by the *M. tuberculosis* complex and enteric bacteria in acid tolerance, with some hydrophobic amino acids being highly effective. In contrast, γ-aminobutyrate, used in the enteric bacteria, had no effect in the mycobacteria. This study proved principle that Phenotype MicroArrays can be used with slow-growing pathogenic mycobacteria and already has generated interesting data worthy of further investigation.

## Introduction

Pathogenic, slow-growing mycobacteria include the *Mycobacterium tuberculosis* complex, of major importance as human and animal pathogens. While *Mycobacterium tuberculosis* causes death and disease in humans, leading to 2 million fatalities a year, *Mycobacterium bovis* (which is 99.95% similar at the nucleotide level) causes financial devastation with losses of $3 billion a year to agriculture [1].

At a genetic level, these organisms are well characterised: several strains have had their whole genome sequenced (http://www.sanger.ac.uk/resources/downloads/bacteria/mycobacterium.html; http://genolist.pasteur.fr/TubercuList/ and links therein). Intriguing links between their molecular typing and biology have been deduced. For example, a Beijing lineage has been identified as an increasing cause of disease, particularly in Asia, and is associated with outbreaks of drug-resistance elsewhere [2]. Gene chip technology has allowed polymorphisms to be studied across a worldwide distribution, giving deep insights into the population biology of *M. tuberculosis* [3]. This work suggested that *M. tuberculosis* strains evolve to adapt to local human populations. In the case of *M. bovis*, molecular typing of strains is used in surveillance of bovine tuberculosis in cattle and wildlife in the UK. One notable finding of the spacer-oligonucleotide typing (spoligotyping) done in the UK is that around 60% of the cattle are infected by just two spoligotypes of *M. bovis*, types 9 and 17. Type 17 represents an emerging microepidemic, increasing at a rate significantly faster than all other field strains of *M. bovis* [4].

Though molecular tracing helps us to identify emerging strains, it does not readily provide any information about their phenotypes. This is a substantial gap in our knowledge since it is the phenotype which is selectable and must relate to the apparent evolutionary advantage of one strain over another. Encouragingly, principal component analysis of metabolites clustered *M. bovis* strains with their spoligotypes [5] showing a link between molecular type and phenotype, without identifying the individual metabolites. A key metabolite was implicated in the Beijing strains that are hypervirulent in mice. They produce an immunomodulatory phenolic glycolipid located on the bacterial surface which, within this lineage, has been associated with virulence [6]. However, this glycolipid likely has to act in concert with other phenotypic characteristics [7], perhaps including those arising from constitutive expression of the *dosR* regulon [8] with its effects on global regulation of metabolism. A direct indication that differences in the utilisation of substrates may be important came from a study to reveal the nature of the phenotypic differences between the emerging type 17 strains and other *M. bovis* strains. While their lipid composition was indistinguishable, differences in the rates of incorporation of propionate and acetate into straight chain fatty acids and pyruvate was clearly evident [9]. Together, these data suggest differences in metabolism, and in metabolites produced, could be important in understanding the emergence of new strains and pathogens in the *M. tuberculosis* complex.

The targeted approaches outlined thus far have been slow and painstaking. Therefore, the use of a commercially available Phenotype MicroArray™ (Biolog) in which twenty-five 96-well plates in which nearly every well had different metabolites, substrates or conditions was assessed as a way of generating phenotypic data rapidly. This technology is based on bacteria producing NADH from which electrons reduce a tetrazolium dye in a redox reaction, resulting in irreversible formation of a purple colour. The rate of electron flow through the respiratory chain, and thus dye reduction, depends upon the conditions in each individual well of a microtitre plate. Biolog OmniLog instrumentation is used to read and record the colour change every 15 min so this provides quantitative and kinetic information about the response of bacteria to each condition in the Phenotype MicroArray (PM) [10]. This appeared a promising approach because the reduction of tetrazolium salts to formazan dyes has been used previously to detect mycobacterial respiration, viability and growth [11]. Moreover, tetrazolium dye reduction gave a perfect match with the original BACTEC method [12], which was used routinely in diagnostic work involving mycobacteria. For our work, we used Biolog plates PM1 to PM4, giving 190 carbon sources, 95 nitrogen sources, 59 phosphorous sources and 35 sulphur sources and the PM9 and PM10 plates giving 192 tests of environmental conditions such as pH and salt concentrations in a rich culture medium (Table S1 gives the conditions in each well of the PM1 to PM10 plates). Our experimental design involved using a low inoculum so that it needed growth to occur in a well for the density of bacteria to become high enough for clear dye reduction to be observed. However, it is a property of PM technology that if a high enough inoculum is used, growth is not necessary for the dye reduction to occur, and we cannot exclude the possibility of high respiration rates with minimal growth resulting in dye reduction in some wells.

In this paper, both the range of nutrients tested, and the use of tetrazolium dye reduction to indicate their use, is novel for the slow-growing *Mycobacterium tuberculosis* complex of pathogens.

## Methods

### Bacterial Strains and routinely used PM Conditions

Mycobacterial strains used in this study are described in Table 1. PM plates PM1, 2A, 3B, 4, 9 and 10, Inoculating Fluid-0a (IF-0a) and Dyes D and G (proprietary dye *mixes*) were obtained from Technopath, UK, and were *bona fide* Biolog™ (Hayward CA) materials. Middlebrook growth media and enrichment and yeast extract were obtained from Difco, all other chemicals were obtained from Sigma-Aldrich or VWR Merck.

**Table 1:** Strains used in this study.

Strains	Characteristics	Source or reference	Code	Colour
*M. tuberculosis* H37Rv	*M. tuberculosis* human strain used extensively in laboratory research. From two separate laboratories. Genome sequenced.	AHVLA Weybridge CollectionRoyal Veterinary College	RvpRvv	Blue
*M. tuberculosis* Tb12	Bangladeshi clinical isolate	Banu *et al.*(2004)	12	Purple
*M. tuberculosis* Tb30	Bangladeshi clinical isolate	Banu *et al.*(2004)	30	Torquoise
*M. tuberculosis* Bj5208	Beijing type clinical isolate	Brian Robertson, Imperial College; Martinez-Ganboa *et al.* (2008)	Bj	Green
*M. bovis* AF2122/97	Genome sequenced strain, type 9, VNTR profile 8555*33.1	Garnier *et al.* (2003)	9b	Orange
*M. bovis* 61/0038/01	Type 9, VNTR 6554*33.1	AHVLA Weybridge Collection	9a	
*M. bovis* 61/3558/00	Type 9, VNTR 7524*33.1	AHVLA Weybridge Collection	9c	
*M. bovis* 61/1121/01	Type 17, VNTR 7555*33.1	AHVLA Weybridge Collection	17a	Red
*M. bovis* 61/3139/06	Type 17, VNTR 7455*33.1	AHVLA Weybridge Collection	17b	
*M. bovis* 21/7917/05	Type 17, VNTR 7554*33.1	AHVLA Weybridge Collection	17c	
*M. bovis* 61/1307/01	Type 35, VNTR 3354*33.1	AHVLA Weybridge Collection	35a	Brown
*M. bovis* 61/0507/01	Type 35, VNTR 3354*33.1	AHVLA Weybridge Collection	35b	

The code is used in Figures 3 to 5, Table 2, Tables S1, S2, S3; S6, S7, S8, S9, S10, S11, S12, S13, S14 and Figure S6. In Figures S1, S2, S3, S4, S5, the human strains referred to in are H37Rv (from AHVLA Weybridge Collection), H37Rv DM (from the Royal Veterinary College) ; Bj5208 as H5208; the *M. bovis* strains abbreviated to the four figure part of their strain numbers. Elsewhere, as space permits, the full strain names are used. The colours are used in Figure 5 and 6, and Figures S6 and S7.

Development work leading to the routinely used PM protocol is summarised briefly, with the conclusions, in Text S1.

To prepare the inoculum for PM plates, bacteria were grown in Middlebrook 7H9 medium containing 10% (v/v) albumin-dextrose-catalase (ADC) enrichment, 0.42% sodium pyruvate and 0.05% (v/v) Tween 80. Bacteria were harvested at mid-logarithmic stage (OD_600 nm_ = 0.30 to 0.60), washed twice with 20 mM phosphate buffer (pH 6.8) containing 0.025% (v/v) tyloxapol and incubated in IF-0a GN/GP for 24 h at 25°C as a starvation step. Then the turbidity was adjusted to OD_600 nm_ = 0.60 (0.18 mg dry weight/ml) . PM additive solutions for each plate were made according to Table S2. For plates PM1 to PM4, PM plates were inoculated with 100 µl of the mixture made up with the following volumes per plate: IF-0a GN/GP at 1.2×(10 ml), Dye mix G at 100×(0.12 ml), PM additive appropriate to the plate (Table S2) at 12×(1 ml) and bacteria in IF-0a GN/GP at 13.64×(0.88 ml). The strength (e.g.100×) is calculated in according to the manufacturer's specifications. For plates PM9 and PM10, PM plates were inoculated with 100 µl of the mixture made up with the following volumes per plate: Middlebrook 7H9 broth (Difco) at 1.2×(10 ml), Dye mix G at 100×(0.12 ml), PM additive appropriate to the plate (table S2) at 12×(1 ml) and bacteria in IF-0a GN/GP at 13.64×(0.88 ml). For each plate, the final volume of mixture was 12 ml with OD_600 nm_ = 0.044. After plate inoculation, each plate was sealed with transparent film and the lid was placed on the sealed plate. The plate lid was securely taped around with transparent sealing tape, sprayed with 70% aq ethanol, transferred to an OmniLog (Biolog, Inc.) incubator and incubated at 37°C for 7 days. Taping likely helped to negate edge well effects as the volume of edge wells was the same as non edge wells after 7 days' incubation. Each inoculum was used to inoculate one of each type of plate; replicates were with separate inocula, and therefore biological replicates. Assays were performed in triplicate or quadruplicate plates, though in cases where plates could not be used (e.g. due to contamination), fewer replicates may be presented in the Results.

### Contents of PM plates

The content of every PM plate from PM1 to PM10, is given in Table S1. As alphabetical order of substrates/conditions and order on the PM plate do not correspond, wells are referred to after the substrate/condition name as the PM plate followed by the well identifier in the form (plate, well), for example (PM1, B9) where required for clarity in the text.

### PM Data handling and statistical analysis of data from PM1 to PM4 plates

The Biolog OmniLog PM software in Parametric module exported the data for each run into CSV files, these file contained measurements related to dye reduction at discrete time points (every 15 minutes) throughout the run (up to 168 h), and a final AUC (Area Under the Curve) figure for the entire run. Data sets were labelled to show the strains and spoligotypes that were represented within.

To check whether kinetic curves corresponded to growth, and resultant dye reduction, kinetic growth curves for strains within each well were also produced- in SAS using a GPLOT procedure (Statistical Analytical Software, Medmenham, Marlow, Buckinghamshire: http://www.sas.com/offices/europe/uk/index.html) - and used to judge the criteria used to accept or reject data (below). All the kinetic curves of PM data are presented as supplementary data (figures S1, S2, S3, S4, S5). Note, in some of these figures, data from “unstarved” bacteria are included. This data is not presented elsewhere in this paper and was not used in any of the analyses reported here.

Inspection of the kinetic curves revealed that the AUC values were often misleading, for example in wells where the baseline was >0, or if abiotic or unsustained dye reduction had occurred but then ceased. Using the PM software Parametric module, it was evident that any abiotic or unsustained dye reduction had ceased by 48 h. Therefore, instead of using AUC values, CSV files were copied into Excel spreadsheets. The dye reduction values at 0 h, 48 h and 165 h (the final time point in all sets of data) were used to calculate mean values, standard deviation (SD) and standard error of the mean (SEM) values for dye reduction (i) throughout the 165 h, calculated by subtracting the 0 h value from the 165 h value for each data set, and (ii) for “sustained dye reduction” which was calculated from the 165 h value minus the 48 h for each set of data. The units are referred to as “Omnilog units” and are generated by the Biolog OmniLog PM software from readings of colour intensity. Essentially, these are photographic readings: OD or absorbance reading were not taken. All these mean, SD and SEM values are shown in Table S3. Statistical significance for differences between strains were determined for each well by performing pairwise 2-tailed *t*- tests on values for each strain across the spreadsheet using Excel statistical functions. Thus, the *P* values from these *t*- tests are also all in Table S3.

### PM Data handling and statistical analysis of data from PM9 and PM10 plates

**Figure 1:**
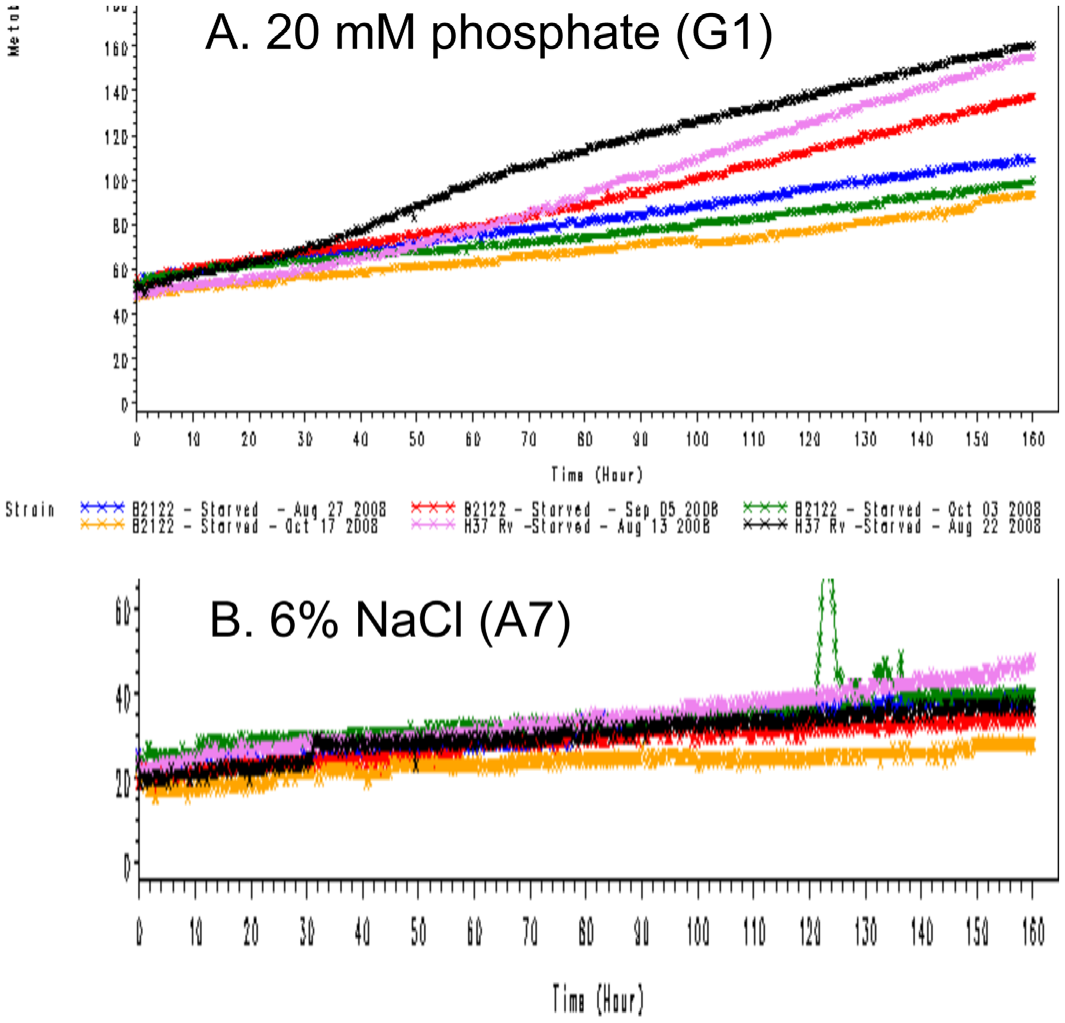
Dye reduction in PM9 and PM10 plates is generally more rapid for *M. tuberculosis* H37Rv than *M. bovis* AF2122/97. Omnilog units (arbitrary colour units due to dye reduction) were plotted against time (0 to 168 h). A. The well (PM9, G1) used as a control well, with 20 mM potassium phosphate is shown as representative of >70 wells on the PM9 plate in that they all had more rapid dye reduction with H37Rv (plots in pink and black) than AF2122/97 (plots: other colours). The other traces can be viewed in Figures S1, S2, S3, S4, S5 B. Dye reduction in the presence of 6% NaCl (PM9, A7) was similar with H37Rv and AF2122/97.

Dye reduction in PM9 and PM10 strains varied with strain, thus in nearly all the wells, dye reduction was higher for H37Rv and AF2122/97. Using such raw data gave highly significant differences between these two strains for ∼70 wells. Given that the objective of these plates is to compare the effects of different environmental conditions- pH, salt, osmolytes on strains, using raw data was unsuitable and it had to be converted to **relative dye reduction** values. A control well with no condition being tested, only the standard culture medium being present, was established as PM9, G1. This is suitable as a control well, as it reflects the pH and phosphate concentration in the Middlebrook (7H9) medium used in these plates. Next, given that t = 0 h values were not always baseline, we subtracted the starting dye reduction (t = 0 h) value for each incubation from every value for that incubation to give **generated dye reduction** values in each well during the incubation period. We cannot be sure why the baseline was sometimes high in PM9 and PM10 plates, but it is notable that a rich culture medium is used in these plates, while the basal medium used for PM1 to PM4 plates gave little dye reduction on its own. Next, the issue of the different rates of dye reduction for each strain was addressed by dividing every generated dye reduction value for each incubation by the final generated dye reduction value for the control- PM9, G1- (Figure 1a) well to give relative dye reduction values. Final time point (165 h) replicates (shown in Table S4) available in all incubations were used for statistical analysis and performing 2-tailed *t*-tests using Excel statistical functions (i) to investigate whether there were any significant differences (*P*<0.05) between strains, then (ii) to determine if any agents had a statistically significant effect on restoring activity lost in wells at pH 4.5, pH 9.5 and with 6% NaCl. Note that the 165 h reading is the last available readout in *all* plates, though in some incubations up to eight more readings- over 2 h- were made.

### Criteria to accept data for analysis- confirmation that dye reduction related to growth and metabolism

**Figure 2:**
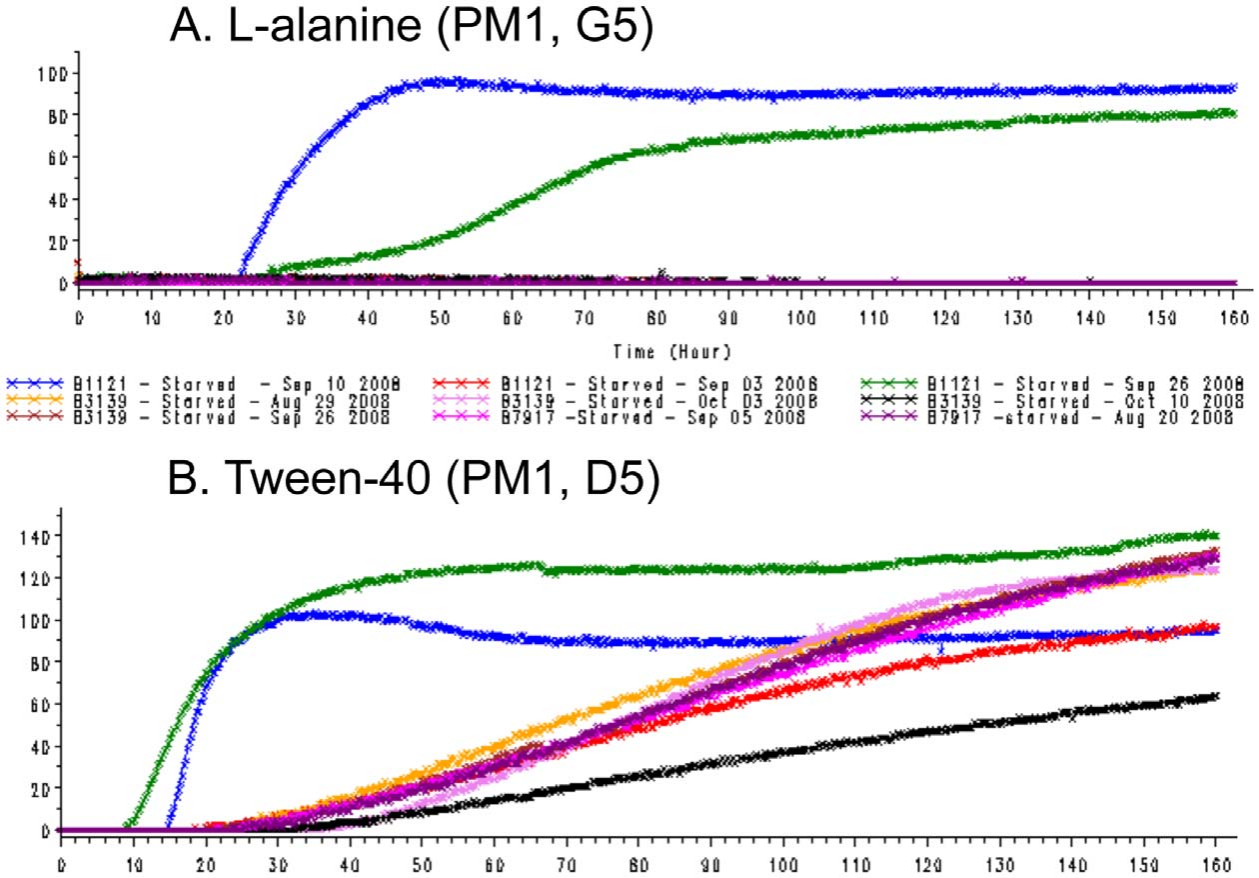
Dye reduction follows growth by *M. bovis* type 17 in Tween-40 but is only seen in contaminated wells in L-alanine. Omnilog units (arbitrary colour units due to dye reduction) were plotted against time (0 to 168 h). Colour coding for replicates is the same in both plots; blue and green plots were of data considered to be from contaminated inocula.

Regardless of the statistical analysis used, dye reduction due to the slow-growing mycobacteria was confirmed by inspecting kinetic curves. The following criteria were applied:

1. Wells with abiotic dye reduction, that occurred in wells without bacteria (Text S2), were discarded. Abiotic dye reduction was obvious even in plates with bacteria as it was observable within 4 h, while dye reduction due to bacteria was barely apparent until 48 h.
2. Wells in which dye reduction was observed at 0 h and essentially gave a flat line against time were discarded.
3. Wells where rapid dye reduction occurred, followed by a flat line or lag phase (blue and green lines in Figure 2), were discarded, as this suggested contamination, likely with a more rapidly growing microbe. Our experiments were planned with the size of the inocula low so that growth had to occur to get good dye reduction by the bacteria. With their mean generation time of 20 h or more, a straight line or slight exponential increase in dye reduction against time (up to 167 h) was seen (lines other than blue or green in Figure 2b). Experimental evidence for rapid dye reduction followed by a flat line or lag phase corresponding to contamination was obtained in wells with L-alanine and Tween 40 with *M. bovis* type 17 (Figure 2). L-alanine is not a substrate for *M. bovis* (Figure 2a) while we showed Tween 40 to be one. Thus, in figure 2b, the Tween 40 reveals all the suspensions to contain viable *M. bovis* but the curves obtained on 10 (blue) and 26 Sep (green) with both substrates must indicate contamination. On the basis of the curves generated in this project, any with a slope of ≥30 Omnilog units/10 h were rejected. This criterion applies to the current work: if, for instance, a higher inoculum is used this cut-off would not be applicable.
  These criteria may eliminate a few true positive wells but given the large amount of data generated a conservative approach, attempting to eliminate false positives, was taken. These criteria excluded 23 of the 390 wells in PM 1 to PM4 plates (390 wells), including all pentoses as they gave abiotic dye reduction.
4. Wells in which <3 Omnilog units dye reduction occurred between 48 h and 165 h. This low value was chosen as it was the median value for strains in PM1 well A3, N- acetyl D-glucosamine, a substrate which was shown to support growth in culture medium as well as permit dye reduction (the culture experiment is described in the Results section).

### Cluster analysis

The PM1 to PM4 plate data for all 28 incubations were clustered based on their phenotypic kinetic profiles according to the following procedure. For each incubation, all the measured profiles (one profile per measured test) were concatenated into a single vector of floating-point numbers. Where data were not available (e.g. data from an incubation was not measured for one or more of the plates), the missing values were flagged. A symmetrical 28×28 distance matrix was then constructed by calculating the Euclidean distance between each pair of incubations using the concatenated profile data for each incubation. Where data from one or both incubations were flagged as missing, data points were skipped and did not contribute to the distance measure. Optimal coordinates in 2 and 3 dimensions were then calculated from the distance matrix using the technique of Distance Geometry, as previously developed for the construction of genetic maps from a genetic distance matrix (Newell, W., personal communication). Incubations with similar profiles occur close to each other in this representation; incubations with very different kinetic profiles are far apart.

Incubations were colour-coded according to known genetic similarities of the bacteria in each incubation, and the coding is tabulated in Table 1. Replicate incubations, where available, are numbered 1 up to 4 after the code for the strain.

### Growth experiments in culture bottles to test hypotheses about substrates generated by our PM data

Dye reduction in PM wells suggests substrates in those wells as possible candidates for culture of mycobacteria. To test these, we inoculated selected strains in 10 ml culture medium in square-bottom 30 ml bottles, shaking them once a day. Sauton base or Roisins base were used for the media. Sauton base contained (per litre) 4 g of L-asparagine, 2 g of citric acid, 0.5 g of K_2_HPO_4_, 0.5 g of MgSO_4_·7H_2_O, and 0.05 g of ferric ammonium citrate, adjusted to pH 7.2 with KOH. Roisin's base [13] was essentially ammonium chloride with salts (Table S5). Individual carbon sources to be tested were added to a final concentration of 2 g/l in Sauton base or 5 g/l in Roisins base except for Tweens which were added to 2 ml/l. When Tweens were not added, 0.025% (v/v) of tyloxapol (not itself a carbon source) was added as a detergent. Experiments in which both Tween 80 and hexoses were added to culture media are described in full in the results.

## Results

### Wells with dye reduction in any strain

#### Effects of substrates- plates PM1 to PM4

**Figure 3:**
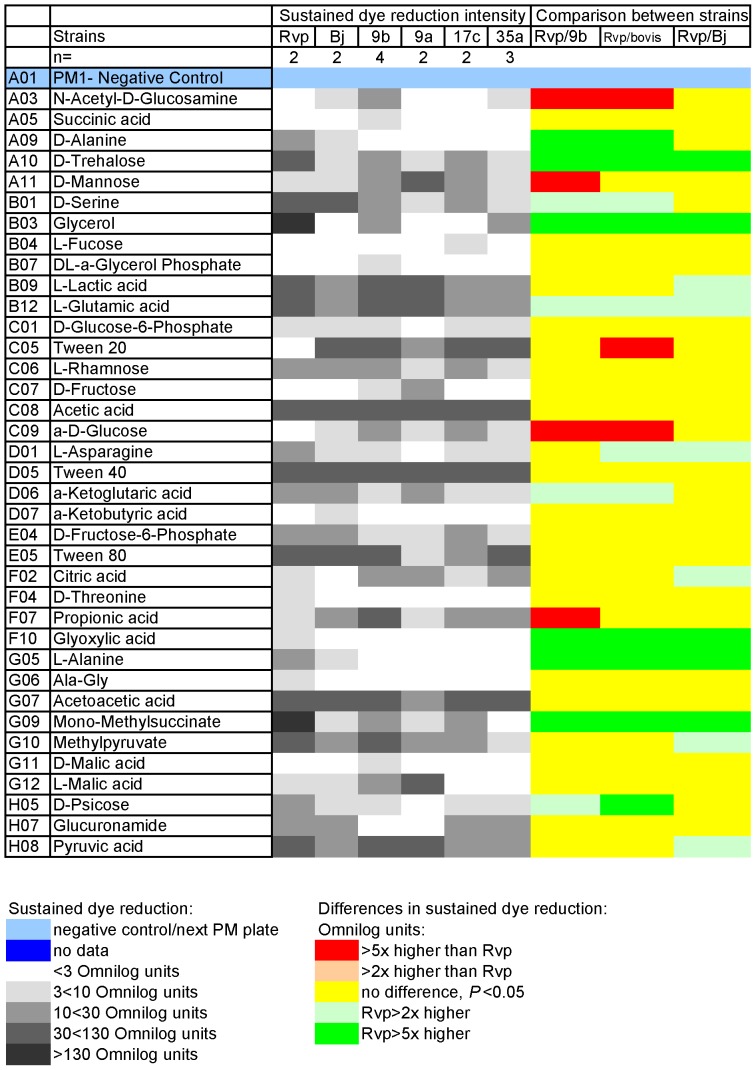
Major carbon sources (Plate PM1) giving sustained dye reduction, from 48 h reading to end (165 h). n = Number of incubations (from separate pre-cultures). Code (corresponds with Table 1): Rvp = H37Rv (AHVLA); Bj = Bj 5208; 9a = type 9 61/0038/01; 9b = type 9 AF2122/97; 17c = type 17 21/7917/05; 35a = type 35 61/1307/01. Cut off for +ve is 3, the median value for well PM01, A03, since we confirmed substrate use as a C-source by growing in culture flasks using it.

**Figure 4:**
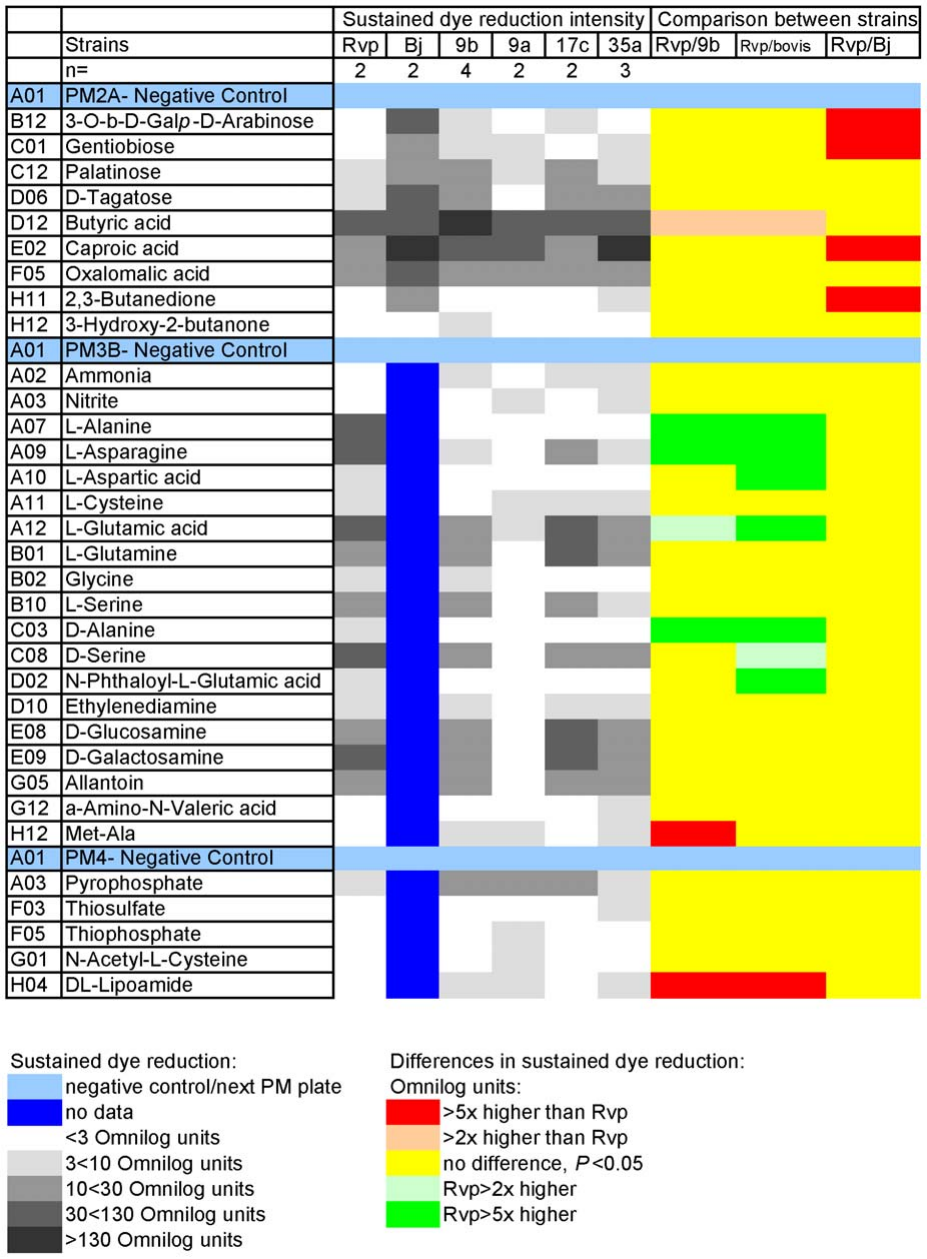
Carbon (plate PM2A), nitrogen (plate PM3B), phosphorous and sulphur sources (plate PM4A) giving sustained dye reduction, from 48 h reading to end (165 h), with carbon sources. n = Number of incubations (from separate pre-cultures). Code (corresponds with Table 1): Rvp = H37Rv (AHVLA); Bj = Bj 5208; 9a = type 9 61/0038/01; 9b = type 9 AF2122/97; 17c = type 17 21/7917/05; 35a = type 35 61/1307/01. Cut off for +ve is 3, the median value for well PM01, A03, since we confirmed substrate use as a C-source by growing in culture flasks using it. ^a^3-O-β-D-Galactopyranosyl-D-Arabinose. Not rejected, but there's a risk the D-ara is released and abiotic dye reduction follows; ^b^accepted, possible trace in a single strain.

Sustained dye reduction by two strains of *M. tuberculosis* and four strains of *M. bovis* is shown in greyscale in the heatmaps (Figures 3 and 4). The more intense the dye reduction, the darker the grey. These data established the starting point for this study, that dye reduction occurred with the *M. tuberculosis* complex strains and was equally demonstrable in field strains as laboratory strains. Essentially these data represent dye reduction from 48 h to 165 h of incubation, due to bacteria using the substrate added to each well. We regarded this as a reliable measure as we noted that glycerol (PM1, B03) allowed dye reduction between 0 h and 48 h in some *M. bovis* strains, even though they cannot grow on glycerol as a sole carbon source. This dye reduction by *M. bovis* was not sustained much after 48 h, while a steady, exponential increase occurred in wells with *M. tuberculosis* throughout the 7 days' incubation. The kinetic curves for the glycerol well, and indeed for every well, can be viewed in figures S1, S2, S3, S4, S5. Actual values in Omnilog dye reduction units from 48 h to 165 h are given in Table S6, together with notes on the application of criteria for acceptance or rejection described in the methods. Dye reduction values from t = 0 h to 165 h are shown in Table S7.

### Differences in substrate use between *M. tuberculosis* and *M. bovis*

In the heatmaps, significantly higher dye reduction is shown in red for *M. bovis* and green for *M. tuberculosis* H37Rv (Figures 3 and 4). First, the originally sequenced strains were compared- H37Rv [14] and *M. bovis* (spoligotype 9, AF2122/97: [1]). There were 21 substrates that gave significantly different dye reduction and at least a 2-fold difference in the dye reduction values. However, some substrates for which there were differences gave good dye reduction by both strains, for example D-serine and L-glutamic acid, while others such as methionyl-alanine (Met-Ala) and DL-lipoamide gave barely above trace dye reduction (pale grey in heatmaps). Therefore, the more stringent comparison where dye reduction was ≥10 Omnilog units and the difference was ≥5-fold is used routinely: these are indicated by darker greys and deep colours respectively in the heatmaps. Thus, when we compared every strain of *M. bovis* (“bovis” in the heatmap strains titles) used in this study against *M. tuberculosis* H37Rv, the differences in substrates that gave dye reduction were very similar to when AF2122/97 and H37Rv were compared. Just one substrate, Tween 20, which gave no dye reduction with *M. tuberculosis* but 38.4 Omnilog units (mean for 48 h to 165 h) with *M. bovis*, was added to the major differences as *P*<0.05 when all the *M. bovis* data were taken into account (Figure 3). Detailed statistical analysis is given in Table S8.

### Differences in substrates supporting dye reduction between *M. tuberculosis* H37Rv and a Beijing strain, Bj5208

Bj5208 gave by far the highest dye reduction (Figure 4) of the strains in this study with gentiobiose and 2,3-butanedione, neither of which gave dye reduction with H37Rv. The substrates (shown in green) that gave significantly higher dye reduction with H37Rv than Bj5208, but did not distinguish H37Rv and *M. bovis*, were citrate and lactate, though the differences were <5-fold (Figure 3). Other substrates that gave wells with dye reduction that distinguished these two human strains were similar to those that distinguished *M. tuberculosis* and *M. bovis* as shown in Figures 3 and 4.

Dye reduction with caproic acid was notably high and like butyric (where it just failed to meet the 2-fold criterion- see Table S9), gave significantly higher dye reduction with Bj5208. The difference in dye reduction with these two short chain fatty acids between H37Rv and other strains, including Bj5208, was due to the long lag phase before dye reduction commenced only in the case of H37Rv (Figure S1).

### Differences in substrates supporting dye reduction between *M. bovis* strains

Not surprisingly, metabolic diversity between the closely related *M .bovis* strains (Table 2) was less than between *M. tuberculosis* and *M. bovis*. Notable differences appeared to be due to AF2122/97 (strain 9b) being more metabolically active, perhaps even to some extent laboratory adapted with its higher dye reduction in wells with glycerol and Tween 80 which are standard culture media constituents. Consistent with this, there were no differences between the type 9 strains less often used in the laboratory, 9a and 9c. The impression from Table 2 is that 35a was the least metabolically active, though actually 35a is the only strain for which dye reduction could be detected- albeit at trace activities which are not presented in Table 2 - with α- amino-N-valeric acid (PM3, G12) and thiosulphate (PM4, F3) (Figure 4). Strain 17c appeared to give dye reduction with a wider range of nitrogen sources than 17b, with D-glucosamine (PM3, B10) and ethylenediamine (PM3, D10) distinguishing only these strains (Table 2). Full detail is presented separately for between- spoligotype (Table S10) and within - spoligotype (Table S11) comparisons of strains.

**Table 2:** Major differences in substrates giving dye reduction between spoligotypes of *M .bovis* and within spoligotypes of *M .bovis*.

		Between *M. bovis* spoligotypes						Within spoligotypes
PM1 plate		9 vs 17		17 vs 35		9 vs 35		
A03	N-ac-D-Glucosamine		9b>17c				9b>35a	9b>9c
B03	Glycerol	9>17	9b>17c	35a>17				9b>9a = 9c
C09	D-Glucose						9b>35a	
E05	Tween 80							9b>9a
G09	monomethylsuccinate			17>35a	17c>35a	9>35a	9b>35a	
G10	methylpyruvate					9>35a	9b>35a	
**PM2A plate**								
D06	D-Tagatose							9b>9a
E02	Caproic acid			35a>17	35a>17c			
**PM3B plate**								
B10	L-Serine							17c>17b
E08	D-Glucosamine							17c>17b

All differences were significant at *P*<0.05. There was at least a 5-fold difference in dye reduction (Omnilog units) between the strains. Dye reduction was >10 Omnilog units for the strain with the highest value. Differences were tested between spoligotypes by (i) comparing pooled dye reduction data for all strains tested and (ii) comparing data from the most used strains: 9b, 17c and 35a. Note that 35a was the only representative of spoligotype 35. Given that there are ten pairwise differences, these data are shown in a table rather than heatmaps.

### Clustering of *M. tuberculosis* complex strains based on their substrate use

**Figure 5:**
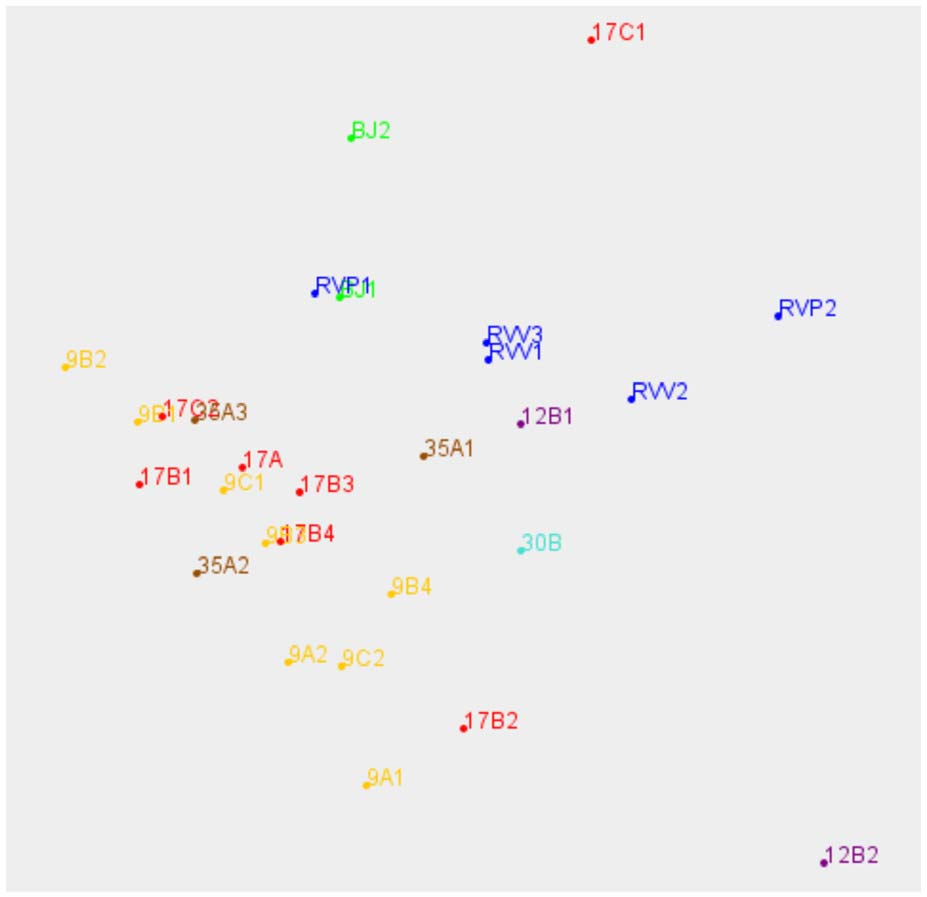
Cluster analysis of *M. tuberculosis* complex strains using Biolog PM data . Each replicate inoculum is displayed with its coordinates in the 3 major dimensions, rotated to display groupings. Coding, including colour, is explained in the list of strains used in this study (Table 1).

Results of the clustering procedure are shown in Figure S6 (2D) and Figure 5 (3D). The members of the *M. tuberculosis* H37Rv (blue) cluster relatively close to one another, as do the members of *M. bovis* clusters for type 9 (orange) and type 17 (red). All the *M. bovis* types co-cluster, including type 35 (brown) and form a separate cluster from the human strains. The two Beijing strain replicates (green) occur close to each other and apparently with some separation from H37Rv (blue). The Bangladeshi strains, Tb 12 and Tb 30, could be investigated by cluster analysis. They had no replicates for at least some of the PM plates but this is acceptable as in cluster analysis each point is generated from a single set of data. They appeared to be in an extended *M. tuberculosis* cluster on one edge of the H37Rv cluster (Figure 5).

### Investigation of unexpected PM data on glycolysis substrates with a growth experiment

PM technology is essentially a first pass screening procedure, generating findings and hypotheses to be tested. One unexpected finding, given the lesion in glycolysis of an inactive pyruvate kinase [15] in *M. bovis*, was dye reduction with the hexoses glucose and mannose by *M. bovis*. This suggested a hypothesis that glycolytic substrates stimulate the use of other substrates. In the PM plates, Tween 80 could be such a substrate. It is a source of oleic and some other fatty acids and gave good dye reduction in its E2 well (Figure 3). In the rest of the wells, Tween 80 is at a suboptimal concentration of 0.1 ml/l. We tested the above hypothesis by performing growth experiments in 10 ml culture media. Tween 80, at 0.1 ml/l, was added to culture media with and without D-glucose and D-mannose. On its own, this concentration of Tween 80 was enough to give only very low growth yields (Table 3). As the results with mannose were almost identical to glucose, only the glucose data are shown (Table 3). Although glucose alone did not support growth of *M. bovis*, it stimulated growth in 0.1 ml/l Tween 80 four-fold (Table 3). The effect was confirmed in Roisins base in which there is no other carbon source (Table 3). These findings could explain the positive wells in PM1 plates inoculated with *M. bovis* (Figure 3) as in the short incubations of the high-throughput PM method very little growth is needed to get good dye reduction.

**Table 3:** Growth of *M. tuberculosis* and *M. bovis* in glucose and Tween 80 as sole usable carbon sources in liquid media.

Medium	Sauton		Roisin's	
Bacteria	*M. bovis*	*M. tuberculosis*	*M. bovis*	*M. tuberculosis*
Glucose (2 g/l)+tyloxapol (0.25 ml/l)	<1	450–600	<1	78–96
Glucose (2 g/l)+Tween 80 (0.1 ml/l)	90–126	450–720	60–82	96–102
Tween 80 (0.1 ml/l)	21–30	24–33	17–19	18–21

Bottles with 10 ml culture medium were set up to investigate synergistic use of hexoses and Tween 80 suggested by Biolog PM data. Tween 80 is a source of fatty acids and a detergent while tyloxapol only acts as a detergent. Strains used in Sauton medium: *M. bovis*: AF2122/97 (9b), 61/0038/01 (9a), 21/7917/05 (17c) and 61/1307/01 (35a); *M. tuberculosis* H37Rv and Tb30. Only AF2122/97 and H37Rv were used in Roisin's medium. Values are growth yield (mg dry weight bacteria/l culture) after 25 day culture. A range is given for all strains- duplicate cultures were done.

### Growth of strains of *M. tuberculosis* and *M. bovis* on substrates that allowed dye reduction in PM plates

One of the advantages of the PM approach is that data output might suggest new substrates for culture and diagnostics of bacteria. In this work, a few carbon sources suggested for growth by their dye reduction in PM plate wells were tested to prove principle. N-acetylglucosamine, methyl-succinate and acetic acid supported growth, though to obtain good yields or get growth of *M. bovis*, Sauton base had to be used. Propionic acid did not support growth in these experiments (Table S12). Like the yeast extract and Tween 80 in the PM plate wells, Sauton base contains additional carbon and nitrogen sources that appear to stimulate growth, though they did not allow growth on their own in the period of the experiments reported in Table S12.

Our PM data suggested differential growth might be obtained with Tween 20. In fact, Tween 20, like Tween 40 and Tween 80 supported growth of all strains tested (Table S12). However, the H37Rv growth was highly flocculent, while *M. bovis* strains gave more dispersed growth. This difference may be related to the lack of dye reduction with Tween 20 (PM1, C5) by H37Rv, in contrast to *M. bovis* strains which reduced dye in the C5 well (Figure 3).

### Effect of osmotic conditions and pH on growth in rich medium- PM9 and 10 plates

**Figure 6:**
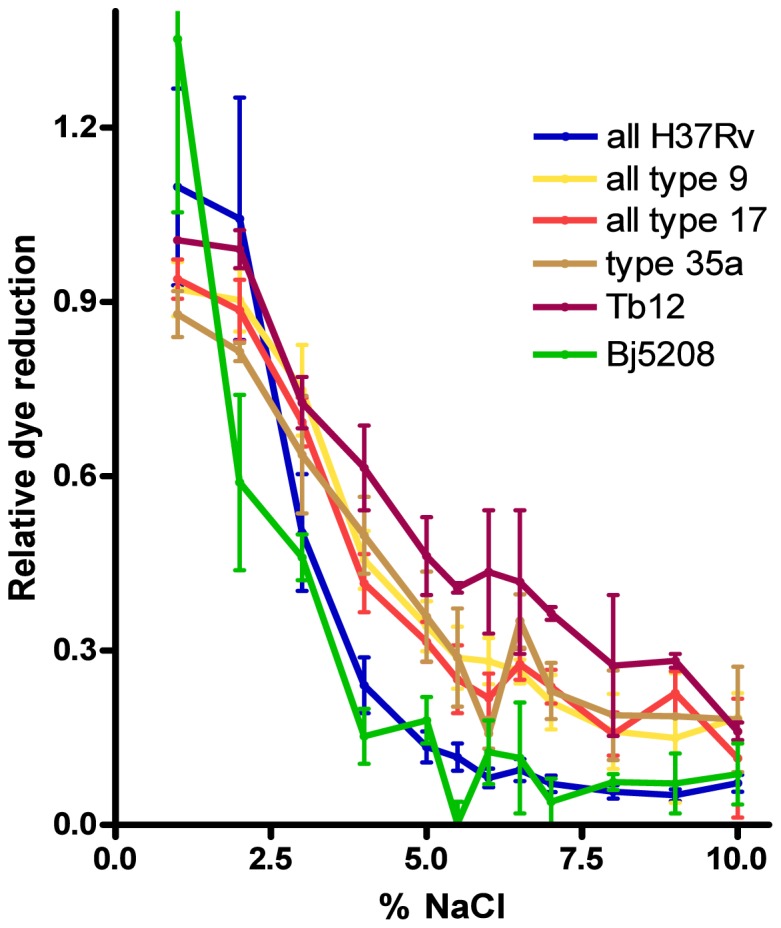
Effect of NaCl on relative dye reduction in *M. tuberculosis* complex strains. Mean ± SEM relative dye reduction values for 2 to 4 experiments are shown for each strain. Incubations were in the wells of PM9 plates.

*M. bovis* strains and *M. tuberculosis* Tb12 were clearly shown to be more salt-tolerant than *M. tuberculosis* H37Rv and Bj5208 (Figure 6). Similar effects were seen with NaCl (Figure 6), KCl (PM9, wells D1 to D4) and sodium formate (PM9, wells E1 to E6) (Table S4, wells are along top row). Statistical analysis shows the above-mentioned differences were significant- this is compelling as *P*<0.05 at a range of concentrations for each agent (Table S4). There was no obvious differential effect of pH: the optimum pH of 6 or 7 and the shape of the relative dye reduction vs. pH plot were similar for all strains (Figure S7), though dye reduction values varied between strains.

**Figure 7:**
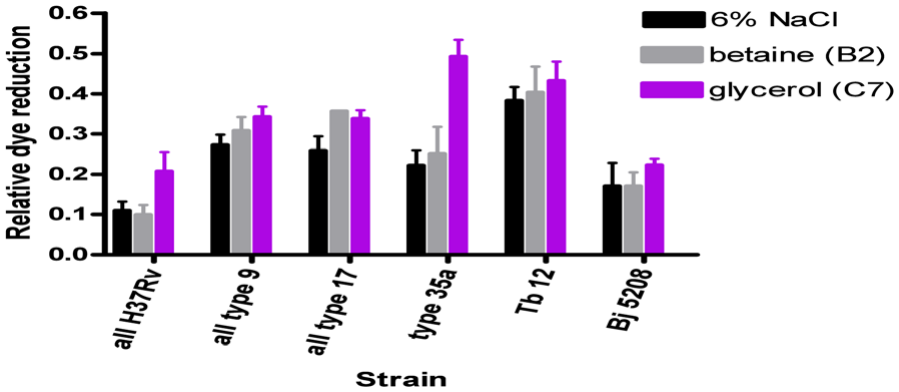
Effect of osmolytes on relative dye reduction in *M. tuberculosis* complex strains. Mean ± SEM relative dye reduction values for 2 to 4 experiments are shown for each strain for a range of candidate osmolytes. The relative dye reduction with no NaCl was always 1.0 or very close to 1.0 (0.98 to 1.02: actual values are for PM9, G1; given in Table S1).

PM9 plates also screen for osmolytes by including a range of compounds in wells with 6% NaCl. Only glycerol restored dye reduction statistically significantly, and then in only *M. bovis* AF2122/97 and *M. bovis* 61/1307/01 (Figure 7). Osmolytes restore activity and enable salt tolerance. However, in very few wells was even one-third of the relative dye activity restored compared with controls without NaCl (well G1). Note, although there is no “0% NaCl” well in the plate, dye reduction in the presence of 1% NaCl (well A1) is not notably different from in the control well (Figure 6). The effects of all osmolytes tested are shown in Table S13. Glutathione appeared to have an effect but this was probably due to abiotic dye reduction (Text S2).

**Figure 8:**
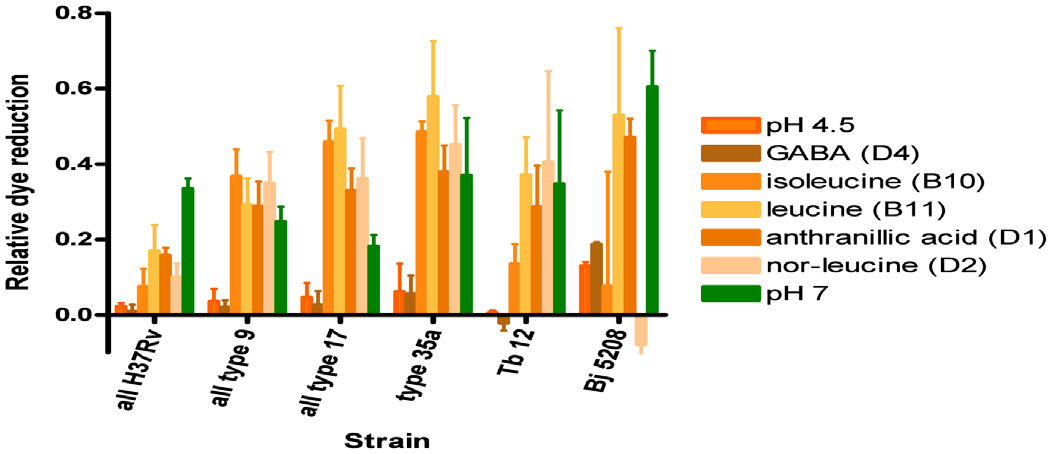
Effect of amines at pH 4.5 on relative dye reduction in *M. tuberculosis* complex strains. Mean ± SEM relative dye reduction values for 2 to 4 experiments are shown for each strain for a range of amines. The bars in green show relative dye reduction values at pH 7 to enable interpretation of the degree of restoration of dye reduction in wells in PM10 plates with the amines at pH 4.5. The basal dye reduction, in wells at pH 4.5 with no addition, is shown in orange.

**Figure 9:**
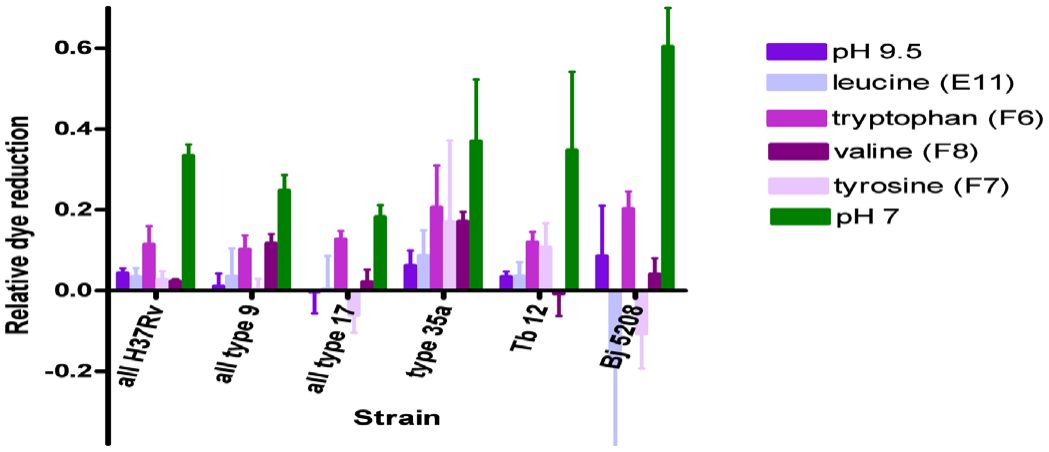
Effect of amines at pH 9.5 on relative dye reduction in *M. tuberculosis* complex strains. Mean ± SEM relative dye reduction values for 2 to 4 experiments are shown for each strain for a range of amines. The bars in green show relative dye reduction values at pH 7 to enable interpretation of the degree of restoration of dye reduction in wells in PM10 plates with the amines at pH 9.5. The basal dye reduction, in wells at pH 9.5 with no addition, is shown in violet.

PM10 plates also screen for amines that act as protectants against acidic and alkaline (pH 4.5 and pH 9.5) conditions. At pH 4.5, isoleucine (well B10) fully restored dye reduction in all *M. bovis* strains and leucine (well B11) fully restored dye reduction in all the bacteria studied (Figure 8). Anthranillic acid (well D1) and L-norleucine (well D2) also restored dye activity to some extent in all the bacteria studied (Figure 8). The effects at pH 9.5 were less clear cut- some kinetic curves fell with time, for others such as when cadaverine (well G5) was added, all the stimulation of dye reduction occurred in the initial ∼6 h (figures S1, S2, S3, S4, S5). Those data had to be rejected but L- tryptophan (well F6) restored dye reduction in all strains, L-tyrosine had a similar effect (well F7) while L-valine (well F8) restored dye reduction in *M. bovis* types 9 and 35 and in *M. tuberculosis* Tb12 (Figure 9). Interpretation of the statistical significance of the differential effects of amines on strains is complicated by the relative dye reduction values being due to both the effect of pH and of the amine but clearly significant differences (*P*<0.021) in the above mentioned wells in PM10 plates, between *M. tuberculosis* H37Rv and *M. bovis* were obtained with norleucine (D2 well) and L-valine (F8 well). In contrast, for well F6, *P*>0.23 in pairwise comparisons between strains, consistent with L-tryptophan being equally effective for all strains (Table S14).

## Discussion

Rapid, high throughput methods for diagnosis and phenotypic analysis of slow growing, pathogenic mycobacteria are highly desirable, for two reasons in particular. The first is the slow growth of these bacteria and consequent long time to culture using traditional diagnostic methods. More intriguingly, genetic diversity is revealed by molecular typing of the *M. tuberculosis* complex but it is barely understood if there are corresponding phenotypic differences. Such methods have been hitherto elusive, but we have achieved proof of principle that a Phenotype MicroArray (PM) method, developed by Biolog, gives signals and differentiates strains of the *M. tuberculosis* complex.

### Slow-growing mycobacteria give dye reduction signals in PM

Overall, 71 of 390 wells in the array gave dye reduction- the signal- in at least one of the strains of the *M. tuberculosis* complex that we tested. The highest signals were 130–140 Omnilog dye reduction units- for glycerol, monomethyl-succinate and butyric acid. Glycerol only supported this level of dye reduction with *M. tuberculosis*, not *M. bovis*, in agreement with it not supporting growth of *M. bovis* in culture medium [15]. Well-known substrates used in culture media generally gave good dye reduction (Figure 3), including Tween 80 and pyruvate as carbon sources. The best nitrogen source across all strains was L-glutamatic acid, notably this is the main nitrogen source in the classic Middlebrook media which are optimal for the culture of the entire *M. tuberculosis* complex.

Generally, fewer wells in the PM3B plate to test nitrogen sources gave dye reduction than expected. For instance, ammonia and aspartic acid can be used in culture media [16] but they gave no, or trace dye reduction depending upon the strain. It may be that they would give better dye reduction if they were used in the pre-culture to prepare the inocula for the PM plates. Amino acids that are known to be taken up but not used as sole nitrogen sources did not give dye reduction either. These included (PM3B plate wells given in brackets) histidine (B3), isoleucine (B4), leucine (B5), lysine (B6), methionine (B7), proline (B9), tryptophan (B12) and valine (C2), for which auxotrophs have been constructed [17], [18], [19], [20]. Interestingly, evidence for the uptake and utilisation of leucine, tryptophan and valine was obtained in PM10 plates as these amino acids ameliorated the inhibitory effect of acid and alkaline conditions on dye reduction.

Of the 71 positive wells, 28 gave <10 Omnilog units in any strain, essentially trace dye reduction. This could be due to respiration without growth, but it is notable that N-acetyl D-glucosamine, which gave 6 Omnilog units or below in all but one strain, supported growth when it was tried as a carbon source in culture media.

Some substrates were used in some, but not all, of the biological replicates. These are indicated by high standard error values in Table S6. This finding was similar to the discovery of “low confidence” substrates, defined as giving dye reduction in 3 to 5 out of 8 replicates, during a genome-scale reconstruction of *Bacillus subtilis* based on PM data, [21]. These represent substrates that the bacteria can use, but are clearly not always used in apparently subtly different experimental conditions.

A way of increasing the number of wells giving dye reduction for mycobacteria may be to have more wells with lipids and fatty acids, given that they are preferred carbon source for mycobacteria [22], [23]. In this work the Tweens, fatty acid esters of sorbitan, gave good dye reduction. In early work, leading up to the development of BACTEC, mycobacterial species were distinguished by using a range of ^14^C -labelled fatty acids and ^14^C -labelled amino acids [24], [25], [26] and monitoring ^14^CO_2_ evolution. Given that the ^14^CO_2_ evolution was related to respiration, this suggests that similar substrates might permit dye reduction, also a correlate of respiration, in PM plates. Further development with lipid substrates is a realistic prospect as there is a lipid plate currently in development (B. Bochner, personal communication).

### Using PM to show phenotypic differences between strains

As well as detecting metabolism, PM also distinguished between strains' use of substrates. Using a simple *t*-test of statistical significance, and for substrates where *P*<0.05 applying a cut-off of at least a 5-fold difference in dye reduction, pairwise comparisons revealed that 12 substrates differentiated from species level between *M. bovis* and *M. tuberculosis* and even to 3 between strain level within one spoligotype of *M. bovis*, where up to 3 substrates were discriminatory. The diagnostic potential of PM technology was further illustrated by cluster analysis of the data, which gave compelling clusters for *M. bovis* and *M. tuberculosis*. *M. tuberculosis* appeared to separate into two clusters- one for the Beijing strain and one for all other strains. This cluster analysis essentially gives a visual representation of the similarities between a set of samples, and is an exploratory data analysis tool. Although this analysis was based on substrate utilisation it was comparable with clustering based on principal component analysis of metabolic products (‘fingerprints’) [5], though the fingerprint analysis better separated *M. bovis* spoligotypes.

### Agreement of PM data with prior knowledge and unexpected findings

Many of the differences between *M. bovis* and *M. tuberculosis* were expected and corresponded with known genetic differences. For instance, lesions in alanine dehydrogenase and pyruvate kinase [1] that prevent *M. bovis* from using alanine as a sole nitrogen or carbon source, or glycerol as a sole carbon source were reflected in PM data with *M. tuberculosis* but not *M. bovis*, giving sustained dye reduction (Figure 3). For nitrogen sources, glutamine and asparagine gave very good dye reduction with *M. tuberculosis*, but were considerably lower with *M. bovis* strains (Figure 4). This apparent loss of metabolic capability may reflect the loss of genetic material as *M. bovis* strains have descended from a progenitor close to *M. tuberculosis* by reductive evolution [27].

Though much of the PM data agreed with previous knowledge, there were some apparent discrepancies. Some could be due to differences in methodology, for example PM additives are not the same as culture medium supplements. We argue in the results section that glycolytic intermediates may stimulate utilisation of PM additives and show this to be the case with Tween 80 when we tested it in an experiment, measuring growth yield instead of dye reduction. Independently, tracer experiments with labelled glucose and acetate showed simultaneous use of these substrates even in *M. tuberculosis*, which is able to use glucose as a sole carbon source for growth [28]. These experiments raise fundamental issues in questioning how, if at all, feedback repression occurs in slow-growing mycobacteria.

Low dye reduction with glucose by *M. tuberculosis* H37Rv may seem surprising given that glucose is a constituent of pre-culture medium in which we grew the inocula for PM plates. However, pre-culture medium was supplemented with pyruvate and Tween 80. We found H37Rv from the preculture inoculated into Roisin medium with glucose as the sole carbon source took 3 weeks to start growing, while H37Rv grew with a lag phase of a few days in standard Roisin medium including glycerol and Tween 80 (unpublished data). Thus, both Roisins and PM data may reflect the limited sugar transporters in *M. tuberculosis* [29] and tight regulation of glucose metabolism [30]. It was puzzling that the Beijing strain used in this study did not give dye reduction with glycerol. This could be due to the toxic methylglyoxal building up from glycerol in this strain [31], [32]. It is also possible that genetic lesions may develop in widely used strains. One of the H37Rv isolates (Rvv) we obtained gave no dye reduction with L-alanine, amongst some other nitrogen sources (Table S3). The natural null mutation of the gene for alanine dehydrogenase [1] in *M. bovis* gave a similar result in PM so, although we did not characterise the gene in Rvv, we speculate a similar lesion may have happened in Rvv. This shows the potential of PM technology to analyse and characterise mutants.

### New ideas and future questions

Essentially, PM is a scanning technology, generating hypotheses to be followed up. We proved this principle by showing the suitability of some new carbon sources for growth that were suggested by their permitting dye reduction in PM plate wells. This is encouraging for following up intriguing findings on metabolism revealed by PM in this work, for instance the better use of short-chain fatty acids by the Beijing strain than the H37Rv strain of *M. tuberculosis* as well as between spoligotype 35 and spoligotype 17 of *M. bovis*.

Further new ideas were generated from the environmental PM9 and PM10 plates. Though they did not characterize strains, they divided the *M. tuberculosis* complex into two groups based on their salt-tolerance in some strains. Interestingly, a classical osmolyte- betaine [33] had no effect on salt-tolerance though glycerol [33], [34] and possibly trehalose did give some salt tolerance. Similarly, γ- aminobutyrate, classically involved in acid resistance in enteric bacteria [33], [35] had no effect in the *M. tuberculosis* complex. Instead some hydrophobic amino acids gave full protection against the effects of incubation at pH 4.5. Thus far, data suggest phosphoinsoitol- based signalling systems [36] and induction of membrane proteins in acid and salt stress [37] but this is the first suggestion that osmoadaptation or pH tolerance through amines occur in these mycobacteria [37] and suggest distinctive mechanisms and agents might be involved. The few leucine decarboxylases found in nature have a wide substrate specificity [38], [39] so could provide a mechanism for the effect of this range of hydrophobic amino acids. This enzyme has not been annotated in the *M. tuberculosis* genome but there are several un-annotated proteins with pyridoxal phosphate predicted binding sites that could be candidates [14].

Although we describe the method we present here as a working method, we have established procedures that will have to be incorporated into any PM protocol. These include the use of liquid preculture rather than harvesting colonies from solid medium and the starvation of bacteria for 24 h, which was needed to minimise background well dye reduction (Text S1). However, the PM technology readily permits adaptation and modification. One change that could be investigated in future could be whether the Tween 80 added at suboptimal level to stimulate metabolism in PM wells should be replaced by a non-metabolizable detergent. This would take the methodology closer to revealing sole carbon sources and should avoid the “false positives” where hexoses gave high reduction by *M. bovis*, which lacks the final enzyme in the glycolytic pathway. It is important to emphasise that although the substrate in each well is needed for dye reduction, there are other additives in each well. Thus, to test for use of a substrate truly as a sole carbon source, dye reduction in buffer, or growth in a minimal medium such as our modified Roisin's medium without Tween 80 ( a carbon source for mycobacteria) would have to be done.

Potentially, the incubations could be made shorter by increasing the inoculum. Indeed a high inoculum might enable dye reduction to be read without growth, extending the method to the uncultivable species *Mycobacterium leprae* [40]. Indeed, PM has already been used to analyse metabolism of the *Coxiella burnetti* without growth [41] in work that led to its axenic cultivation for the first time. However, we found it useful in this study to view kinetic curves (figures S1, S2, S3, S4, S5) to recognise readily increase in dye reduction related to growth. One major point that arose from inspecting kinetic curves was that dye reduction sustained through from 48 h to 165 h best gave data that matched the properties of the mycobacteria in this study- their growth rate, strain differences and known metabolism. In some curves, rapid early dye reduction was abiotic or evidently due to contamination. A consequence of this point is that if PM plates were to be used without a Biolog OmniLog incubator, dye intensity should be read at 0, 48 and 165 h and positives only recorded where the 165 h reading is >48 h reading. Readings without a Biolog OmniLog incubator would be better done as pixels than as OD values since the bacterial suspension contributes to the OD value as well as the dye.

In conclusion, this publication should encourage and stimulate attempts to tailor PMs to address specific questions and develop robust methodologies for the use of phenotype arrays with this difficult, but hugely important group of bacteria.

Refs mentioned only in Table 1: [42], [43]

## Supporting Information

Figure S1 **Kinetic curves for all PM plates with** ***Mycobacterium tuberculosis*** **H37Rv and Bj5208 strains.**
(ZIP)

Figure S2 **Kinetic curves for all PM plates with** ***M. tuberculosis*** **H37Rv from two separate laboratories.**
(ZIP)

Figure S3 **Kinetic curves for all PM plates with** ***Mycobacterium bovis*** **Type 9 strains.**
(ZIP)

Figure S4 **Kinetic curves for all PM plates with** ***M. bovis*** **Type 17 strains.**
(ZIP)

Figure S5 **Kinetic curves for all PM plates with** ***M. bovis*** **Type 35 strains.** Figures S1 to S5 were generated in SAS using a GPLOT procedure, as described in the methods. Each figure is a Zip file containing plots of Omnilog units (due to dye reduction) against time (0 to 168 h) for all wells of each of the six 96 well plates. Each well is identified by (plate, well) and a list of the contents of wells is in Supplementary Table S1.
(ZIP)

Figure S6 **Clustering of strains from PM1 to PM4 data, in 2 dimensions.** An aspect of the 3D clustering is presented in main paper and the key is in Table 1
(DOC)

Figure S7 **pH optima of strains.** Mean ± SEM relative dye reduction values for 2 to 4 experiments are shown for each strain.
(PPT)

Text S1 Method development for preparing suspensions of the *M. tuberculosis* complex to inoculate PM plates
(DOC)

Text S2 Wells with abiotic dye reduction
(DOC)

Table S1 **List of all the conditions/substrates in PM wells for plates PM1 to PM10.**
(XLS)

Table S2 **Preparation and concentration of PM additives for PM plates used in this study.**
(DOC)

Table S3 **Means, SD and SEM and t-tests and ratios for dye reduction data from plates PM1 to PM4.** Pairwise *t*-tests were done for all 384 wells and the actual *P* values are given in this spreadsheet. The data used in the tests are Omnilog units (dye reduction). Notes and colour coding are explained on the spreadsheet.
(XLS)

Table S4 **Differences between strains deduced from data from PM9 and PM10 wells.** Pairwise *t*-tests were done for all 192 wells. The data used in the tests are relative dye reduction values (defined in full in the methods section). As they are relative to (PM9, G1) the relative dye reduction value for that well is, perforce, always 1 or very close to 1. *t*-Tests performed are described in the first column and highlighted in yellow and the *P* values are along the row, corresponding to each well. Where *P*<0.05, the result is highlighted in red where the ratios of mean relative dye reduction values between the strains are >1.5; where they are <1.5 so the difference is slight, although statistically significant, they are highlighted in pink. Where there is almost no difference between the mean relative dye reduction values or when there is a negative value, yet the difference statistically significant, they are highlighted in pink with the *P* value in white .The ratios of mean relative dye reduction values are in rows highlighted in blue.
(XLS)

Table S5 **Roisin's medium.**
(DOC)

Table S6 **Plates PM1 to PM4: Sustained dye reduction, from 48 h reading to end (165 h).** There is one row for every well in the PM1 to PM4 plates including means and SDs for strains coded (in Table 1): Rvp, Bj, 9b,9a,17c and 35a along with all the pairwise *t*-tests summarised in Tables S7 to S11 that gave *P*<0.05.
(XLS)

Table S7 **Plates PM1 to PM4: Dye reduction, from 0 h reading to end (165 h).** There is one row for every well in the PM1 to PM4 plates including means and SDs for strains coded (in Table 1): Rvp, Bj, 9b,9a,17c and 35a
(XLS)

Table S8 **Plates PM1 to PM4: Wells with significantly different dye reduction between** ***M .tuberculosis*** **H37Rv and** ***M. bovis*** **.** Data for wells for which *P*<0.05, there are at least 2-fold differences, and at least one strain has a mean value of at least 3 Omnilog units are summarised here.
(XLS)

Table S9 **Plates PM1 to PM4: Wells with significantly different dye reduction between** ***M .tuberculosis*** **H37Rv and a Beijing strain.** Data for wells for which *P*<0.05, there are at least 2-fold differences, and at least one strain has a mean value of at least 3 Omnilog units are summarised here.
(XLS)

Table S10 **Plate PM1 to PM4: Wells with significantly different dye reduction between** ***M .bovis*** **spoligotypes.** Data for wells for which *P*<0.05, there are at least 2-fold differences, and at least one strain has a mean value of at least 3 Omnilog units are summarised here.
(XLS)

Table S11 **Plates PM1 to PM4: Wells with significantly different dye reduction within** ***M .bovis*** **spoligotypes.** Data for wells for which *P*<0.05, there are at least 2-fold differences, and at least one strain has a mean value of at least 3 Omnilog units are summarised here.
(XLS)

Table S12 **Investigation of substrates that gave dye reduction in PM plates as growth substrates in culture medium.**
(XLS)

Table S13 **Effect of osmolytes using t-tests to show those having a significant effect at 6% NaCl concentration.** Pairwise *t*-tests were performed for the 23 wells in plate PM9 with additions to wells with 6% NaCl. Colour coding (explained in the spreadsheet notes) is used to show the degree of restoration of relative dye reduction where *P*<0.05: the key is at the foot of the spreadsheet.
(XLS)

Table S14 **Effect of amines using t-tests to show those having a significant effect at pH 4.5 and pH 9.5.** Pairwise *t*-tests were performed for the 70 wells in plate PM10 with additions to wells at pH 4.5 and pH 9.5. Colour coding (explained in the spreadsheet notes) is used to show the degree of restoration of relative dye reduction where *P*<0.05: the key is at the foot of the spreadsheet.
(XLS)
