# Supplementary figures and images for: High Throughput Phenotypic Analysis of Mycobacterium tuberculosis and Mycobacterium bovis Strains' Metabolism Using Biolog Phenotype Microarrays (part 2 of 11)
Source: PLoS One. 2013 Jan 10;8(1):e52673. doi: 10.1371/journal.pone.0052673 (PMC3542357; doi:10.1371/journal.pone.0052673)

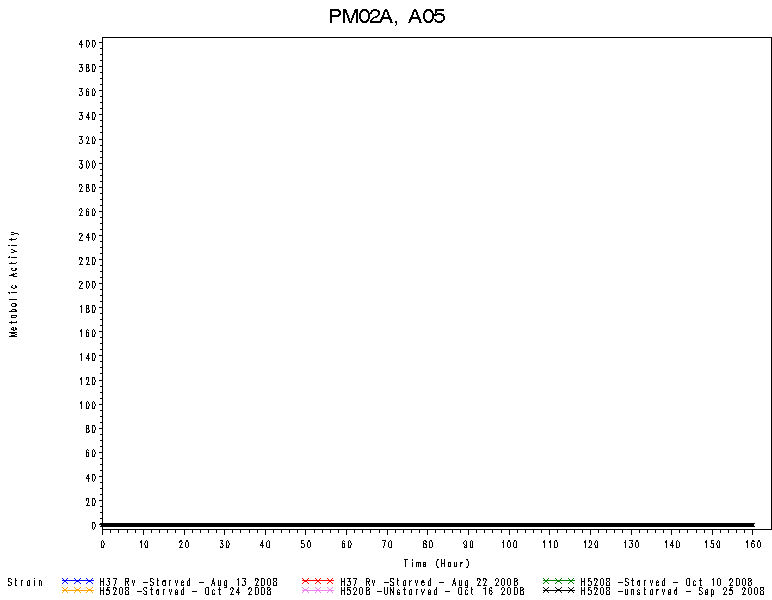

Supplement: Figure S1 — Kinetic curves for all PM plates with Mycobacterium tuberculosis H37Rv and Bj5208 strains. (ZIP) [file pone.0052673.s001.zip › suppl fig 1G H37Rv and Bj5208/Plate02A/pm02aa05.gif]

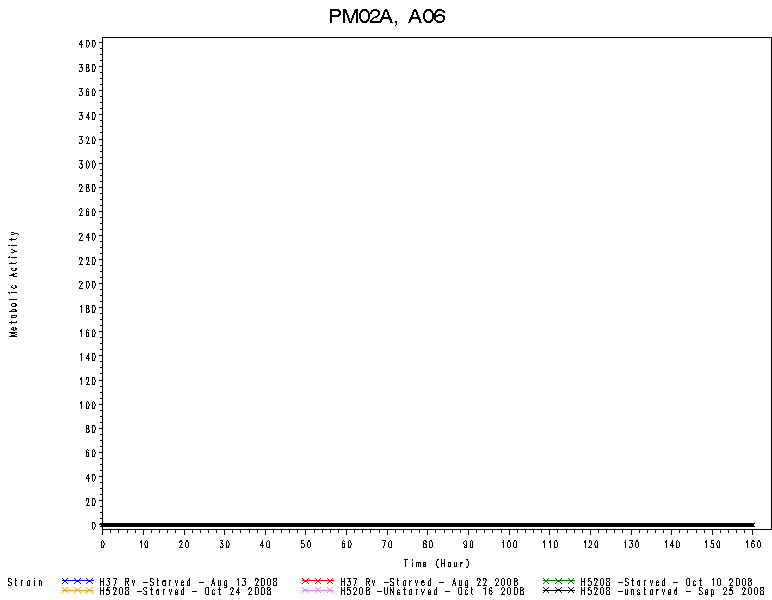

Supplement: Figure S1 — Kinetic curves for all PM plates with Mycobacterium tuberculosis H37Rv and Bj5208 strains. (ZIP) [file pone.0052673.s001.zip › suppl fig 1G H37Rv and Bj5208/Plate02A/pm02aa06.gif]

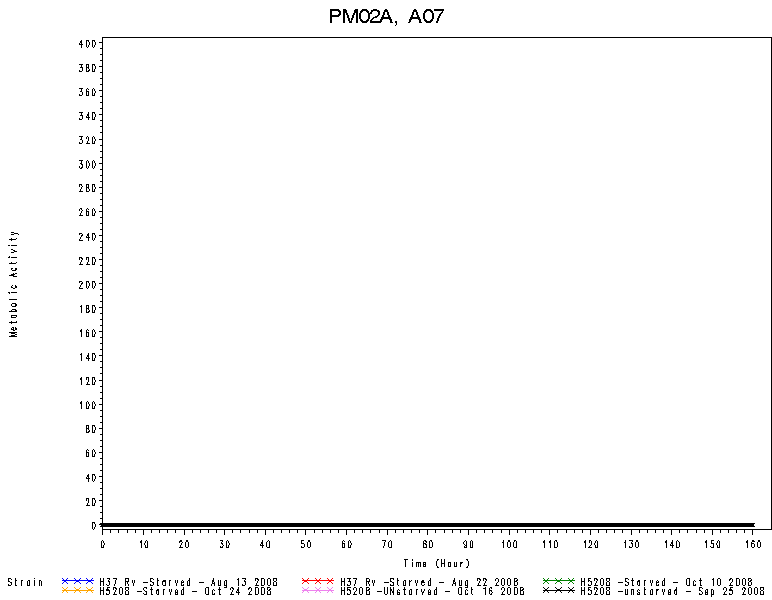

Supplement: Figure S1 — Kinetic curves for all PM plates with Mycobacterium tuberculosis H37Rv and Bj5208 strains. (ZIP) [file pone.0052673.s001.zip › suppl fig 1G H37Rv and Bj5208/Plate02A/pm02aa07.gif]

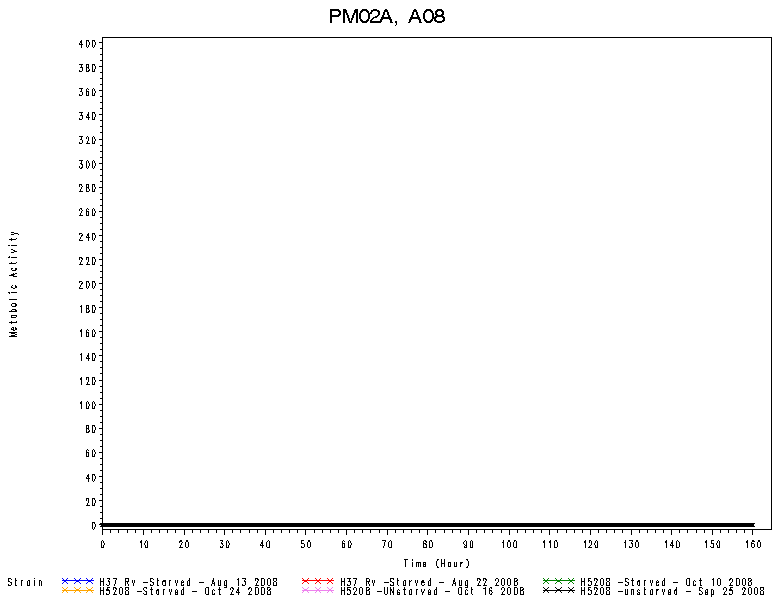

Supplement: Figure S1 — Kinetic curves for all PM plates with Mycobacterium tuberculosis H37Rv and Bj5208 strains. (ZIP) [file pone.0052673.s001.zip › suppl fig 1G H37Rv and Bj5208/Plate02A/pm02aa08.gif]

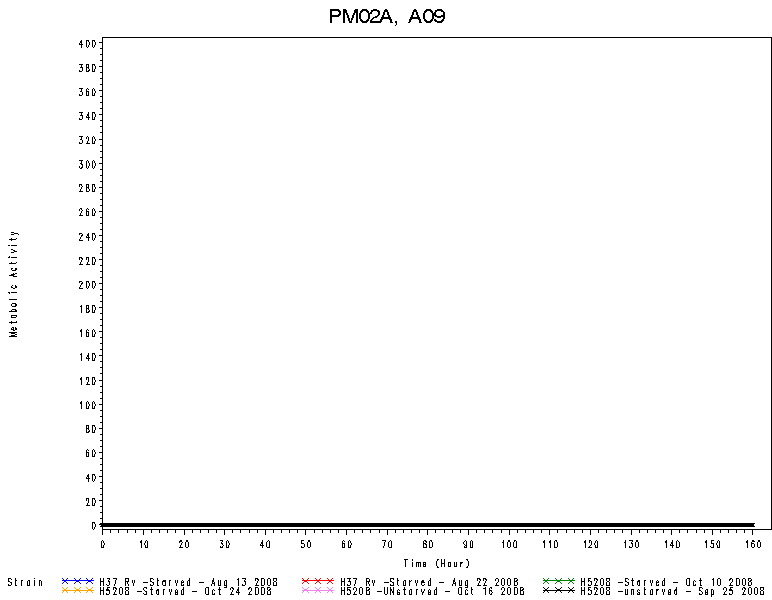

Supplement: Figure S1 — Kinetic curves for all PM plates with Mycobacterium tuberculosis H37Rv and Bj5208 strains. (ZIP) [file pone.0052673.s001.zip › suppl fig 1G H37Rv and Bj5208/Plate02A/pm02aa09.gif]

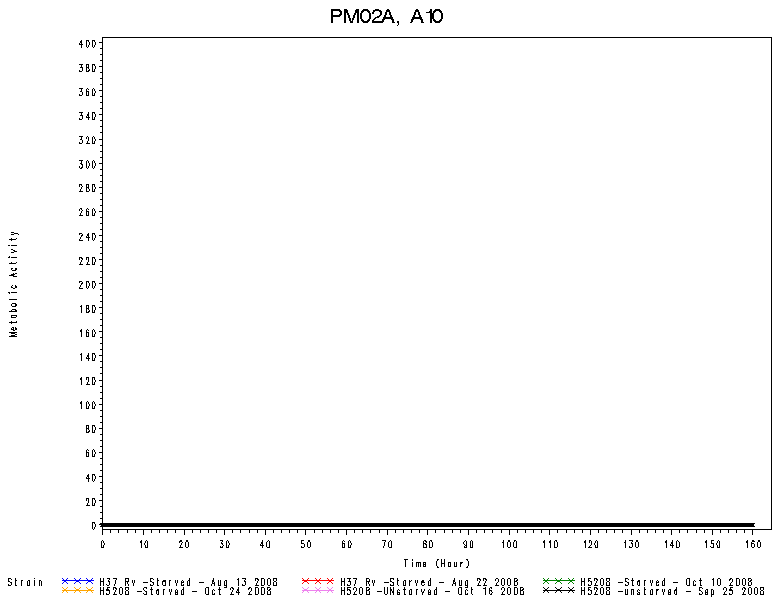

Supplement: Figure S1 — Kinetic curves for all PM plates with Mycobacterium tuberculosis H37Rv and Bj5208 strains. (ZIP) [file pone.0052673.s001.zip › suppl fig 1G H37Rv and Bj5208/Plate02A/pm02aa10.gif]

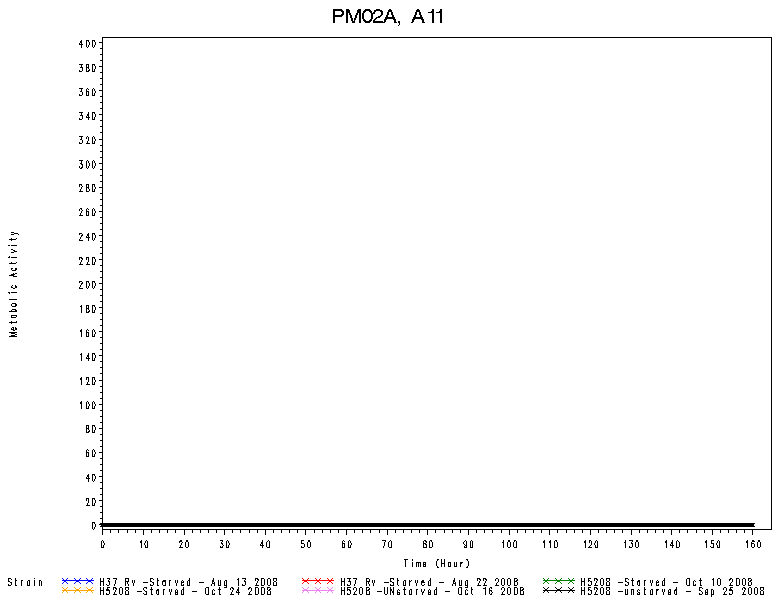

Supplement: Figure S1 — Kinetic curves for all PM plates with Mycobacterium tuberculosis H37Rv and Bj5208 strains. (ZIP) [file pone.0052673.s001.zip › suppl fig 1G H37Rv and Bj5208/Plate02A/pm02aa11.gif]

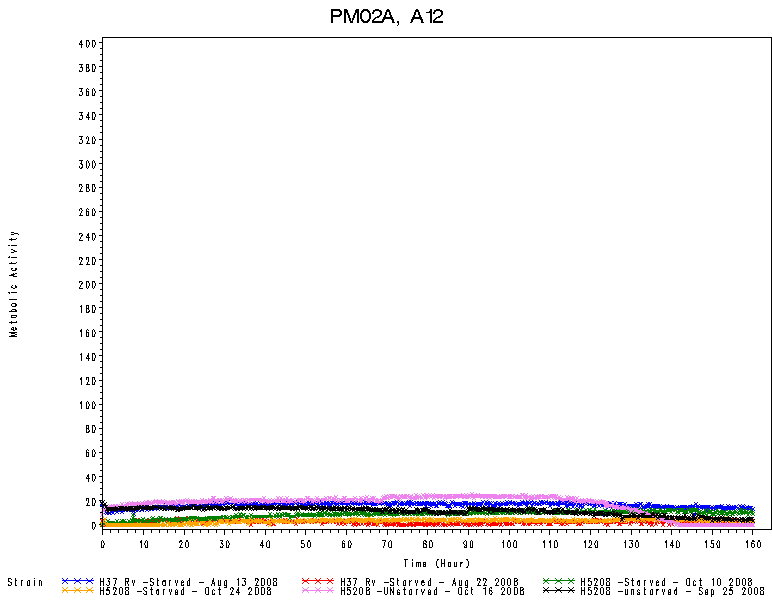

Supplement: Figure S1 — Kinetic curves for all PM plates with Mycobacterium tuberculosis H37Rv and Bj5208 strains. (ZIP) [file pone.0052673.s001.zip › suppl fig 1G H37Rv and Bj5208/Plate02A/pm02aa12.gif]

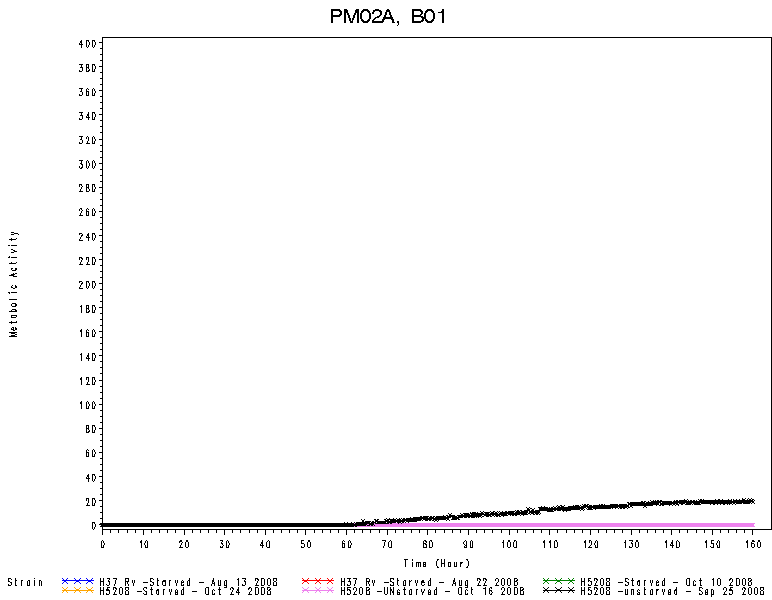

Supplement: Figure S1 — Kinetic curves for all PM plates with Mycobacterium tuberculosis H37Rv and Bj5208 strains. (ZIP) [file pone.0052673.s001.zip › suppl fig 1G H37Rv and Bj5208/Plate02A/pm02ab01.gif]

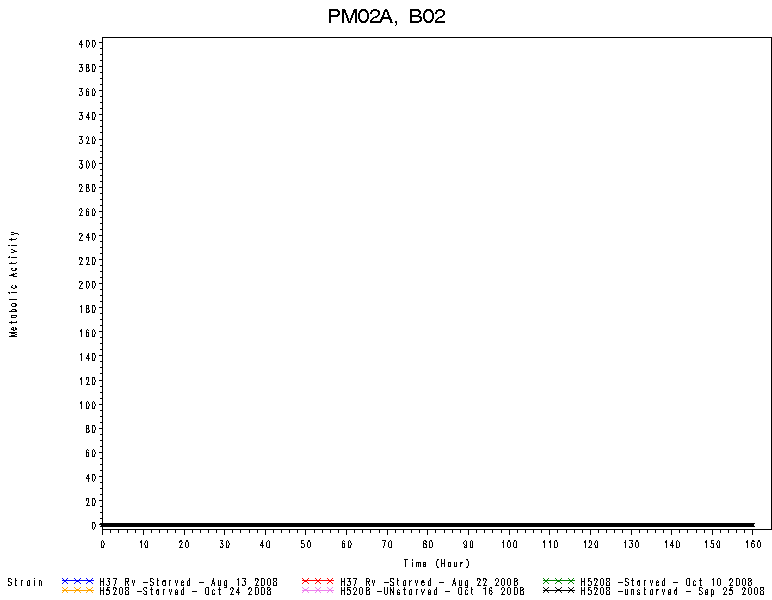

Supplement: Figure S1 — Kinetic curves for all PM plates with Mycobacterium tuberculosis H37Rv and Bj5208 strains. (ZIP) [file pone.0052673.s001.zip › suppl fig 1G H37Rv and Bj5208/Plate02A/pm02ab02.gif]

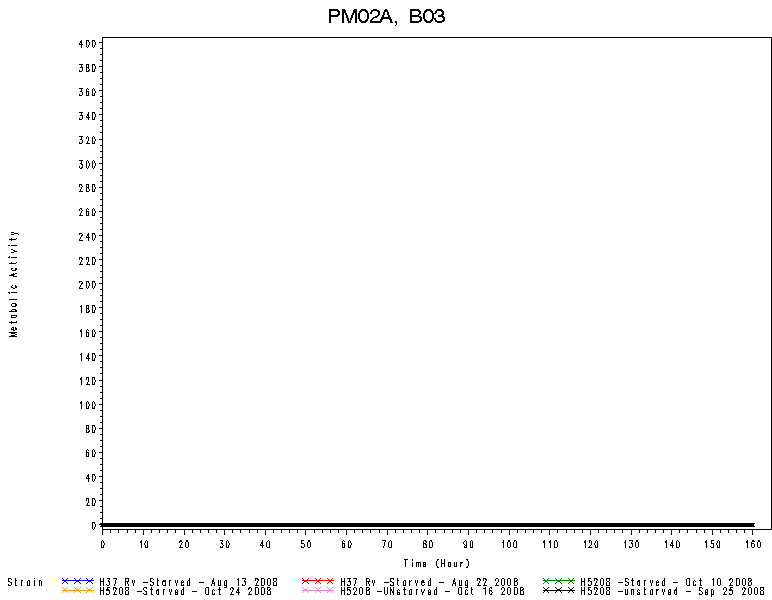

Supplement: Figure S1 — Kinetic curves for all PM plates with Mycobacterium tuberculosis H37Rv and Bj5208 strains. (ZIP) [file pone.0052673.s001.zip › suppl fig 1G H37Rv and Bj5208/Plate02A/pm02ab03.gif]

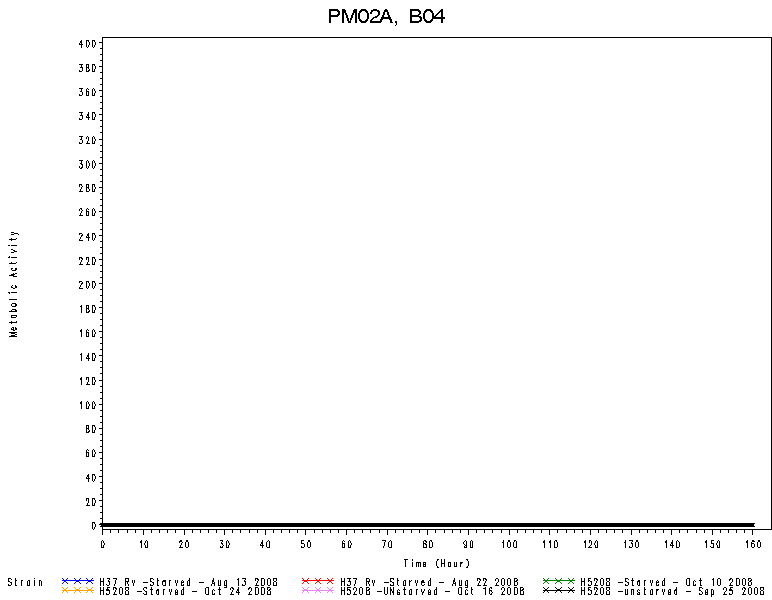

Supplement: Figure S1 — Kinetic curves for all PM plates with Mycobacterium tuberculosis H37Rv and Bj5208 strains. (ZIP) [file pone.0052673.s001.zip › suppl fig 1G H37Rv and Bj5208/Plate02A/pm02ab04.gif]

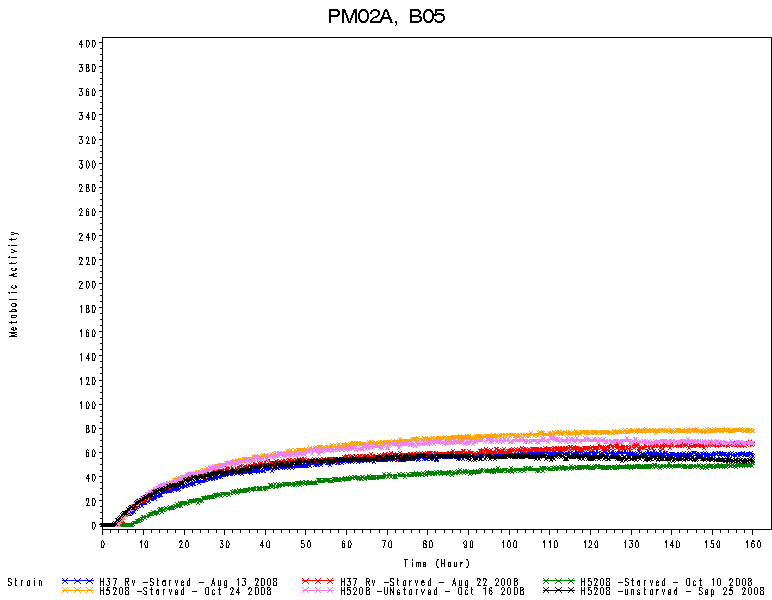

Supplement: Figure S1 — Kinetic curves for all PM plates with Mycobacterium tuberculosis H37Rv and Bj5208 strains. (ZIP) [file pone.0052673.s001.zip › suppl fig 1G H37Rv and Bj5208/Plate02A/pm02ab05.gif]

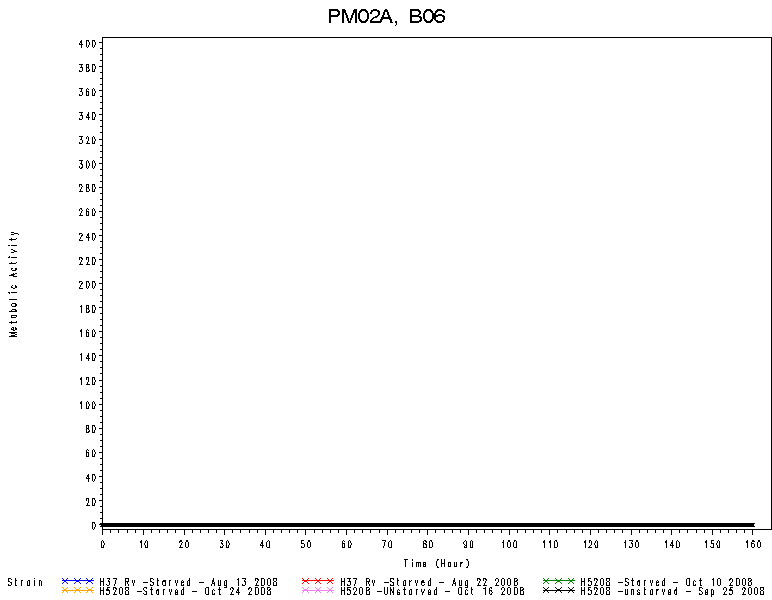

Supplement: Figure S1 — Kinetic curves for all PM plates with Mycobacterium tuberculosis H37Rv and Bj5208 strains. (ZIP) [file pone.0052673.s001.zip › suppl fig 1G H37Rv and Bj5208/Plate02A/pm02ab06.gif]

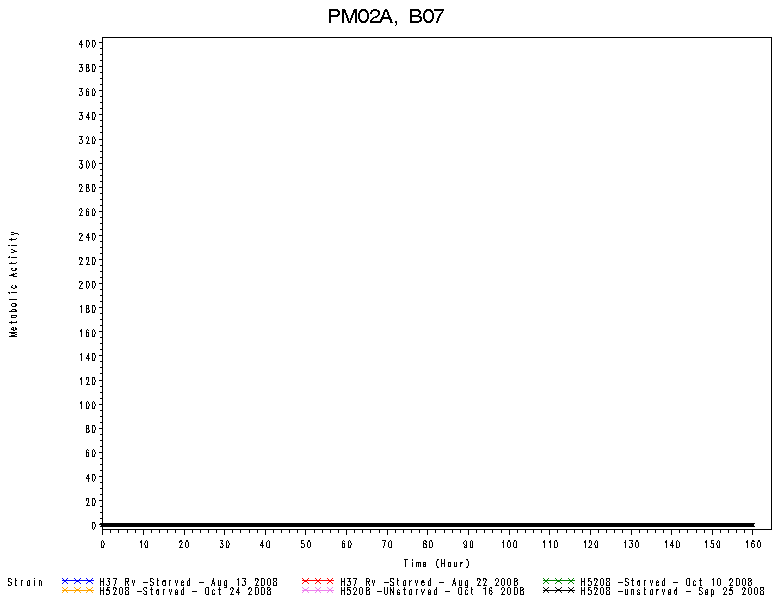

Supplement: Figure S1 — Kinetic curves for all PM plates with Mycobacterium tuberculosis H37Rv and Bj5208 strains. (ZIP) [file pone.0052673.s001.zip › suppl fig 1G H37Rv and Bj5208/Plate02A/pm02ab07.gif]

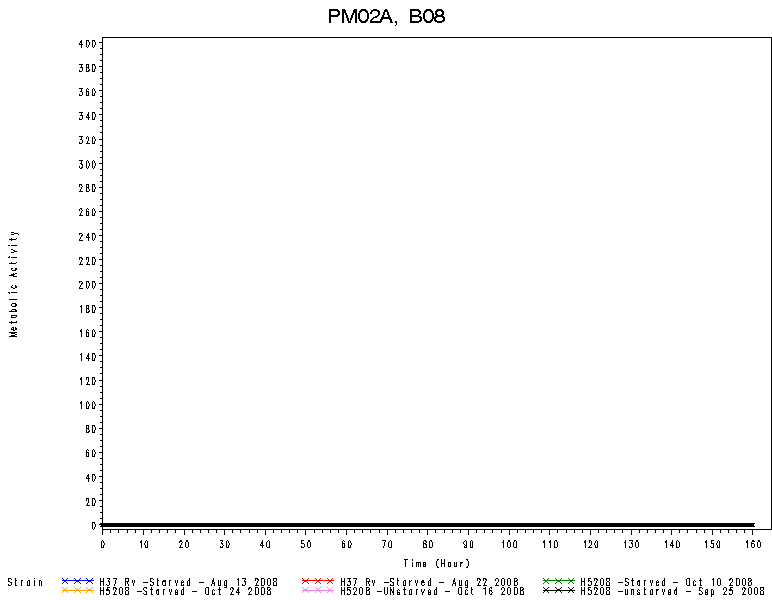

Supplement: Figure S1 — Kinetic curves for all PM plates with Mycobacterium tuberculosis H37Rv and Bj5208 strains. (ZIP) [file pone.0052673.s001.zip › suppl fig 1G H37Rv and Bj5208/Plate02A/pm02ab08.gif]

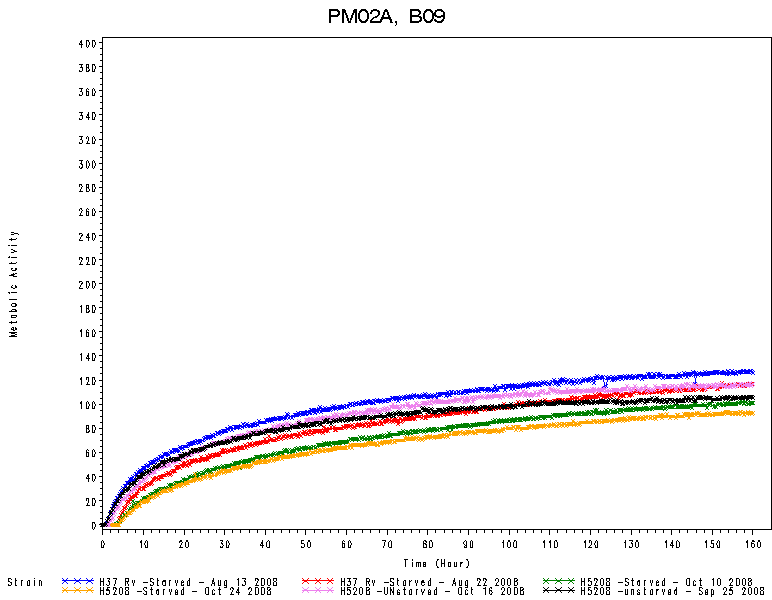

Supplement: Figure S1 — Kinetic curves for all PM plates with Mycobacterium tuberculosis H37Rv and Bj5208 strains. (ZIP) [file pone.0052673.s001.zip › suppl fig 1G H37Rv and Bj5208/Plate02A/pm02ab09.gif]

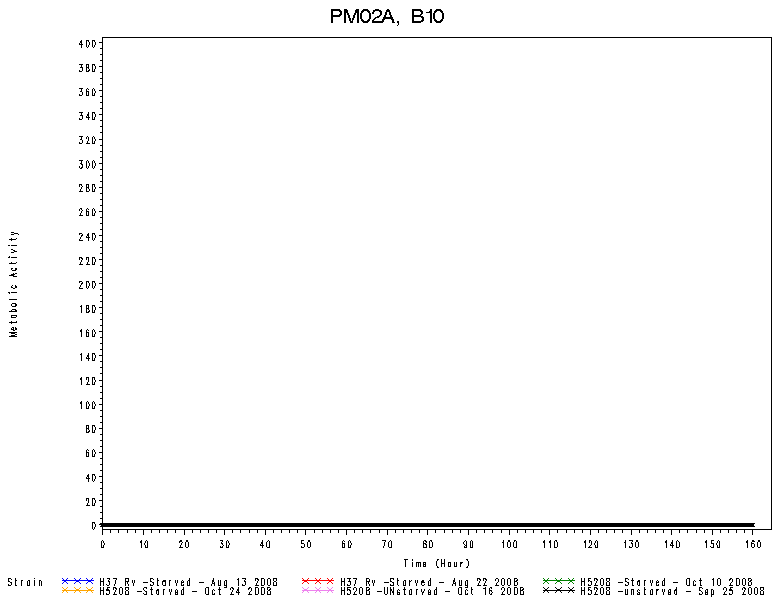

Supplement: Figure S1 — Kinetic curves for all PM plates with Mycobacterium tuberculosis H37Rv and Bj5208 strains. (ZIP) [file pone.0052673.s001.zip › suppl fig 1G H37Rv and Bj5208/Plate02A/pm02ab10.gif]

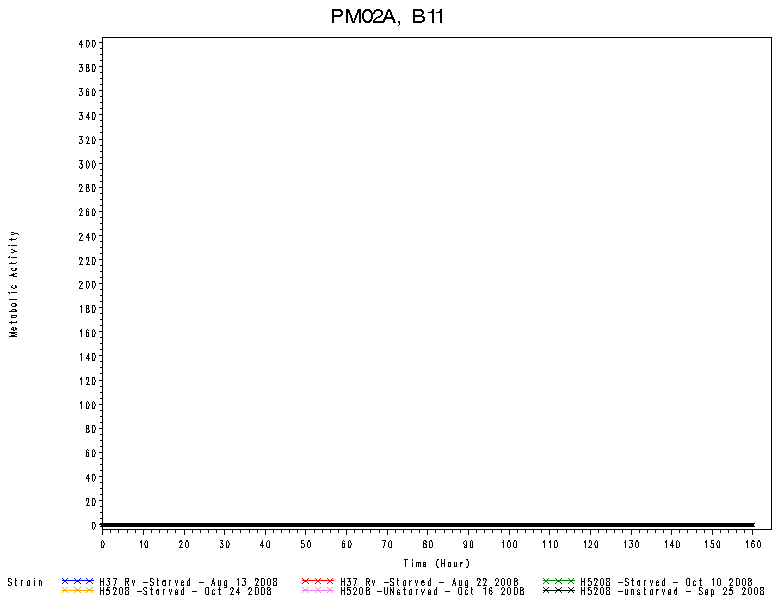

Supplement: Figure S1 — Kinetic curves for all PM plates with Mycobacterium tuberculosis H37Rv and Bj5208 strains. (ZIP) [file pone.0052673.s001.zip › suppl fig 1G H37Rv and Bj5208/Plate02A/pm02ab11.gif]

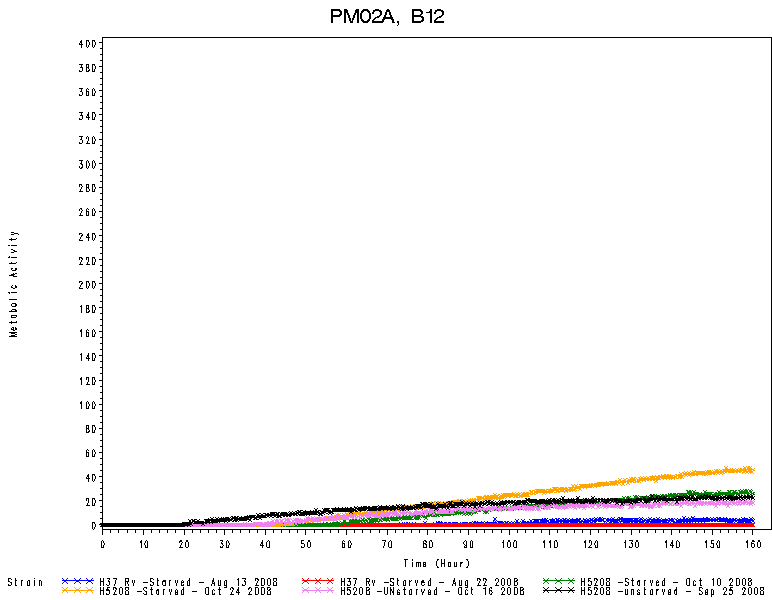

Supplement: Figure S1 — Kinetic curves for all PM plates with Mycobacterium tuberculosis H37Rv and Bj5208 strains. (ZIP) [file pone.0052673.s001.zip › suppl fig 1G H37Rv and Bj5208/Plate02A/pm02ab12.gif]

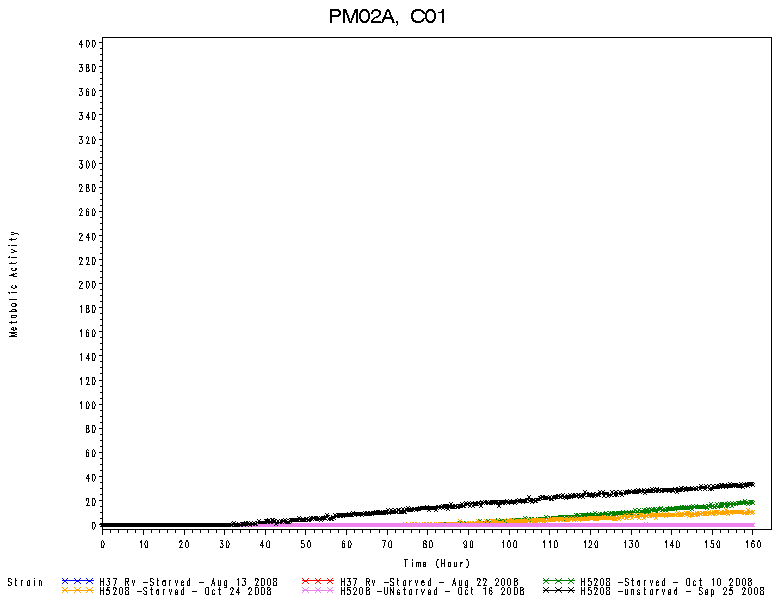

Supplement: Figure S1 — Kinetic curves for all PM plates with Mycobacterium tuberculosis H37Rv and Bj5208 strains. (ZIP) [file pone.0052673.s001.zip › suppl fig 1G H37Rv and Bj5208/Plate02A/pm02ac01.gif]

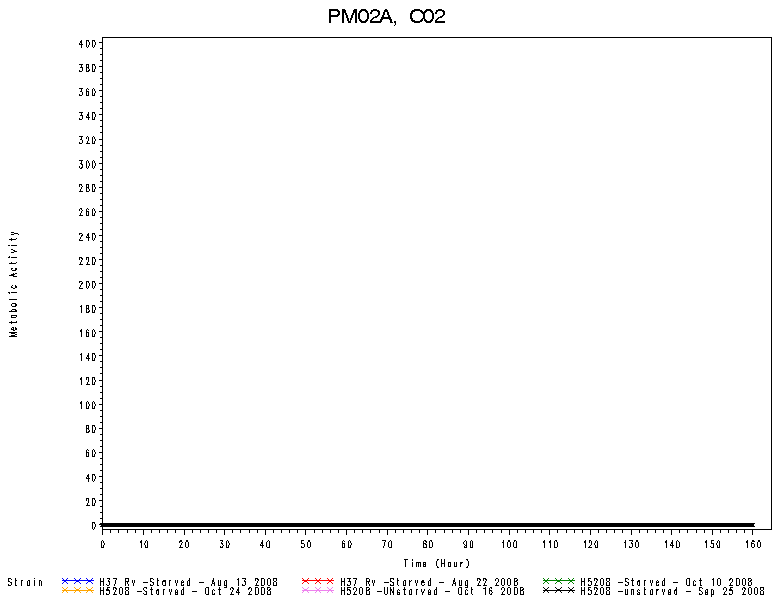

Supplement: Figure S1 — Kinetic curves for all PM plates with Mycobacterium tuberculosis H37Rv and Bj5208 strains. (ZIP) [file pone.0052673.s001.zip › suppl fig 1G H37Rv and Bj5208/Plate02A/pm02ac02.gif]

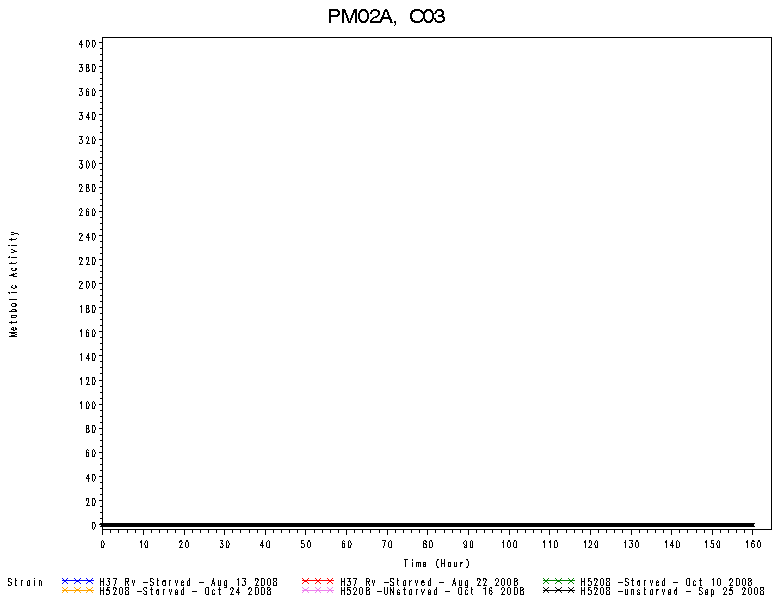

Supplement: Figure S1 — Kinetic curves for all PM plates with Mycobacterium tuberculosis H37Rv and Bj5208 strains. (ZIP) [file pone.0052673.s001.zip › suppl fig 1G H37Rv and Bj5208/Plate02A/pm02ac03.gif]

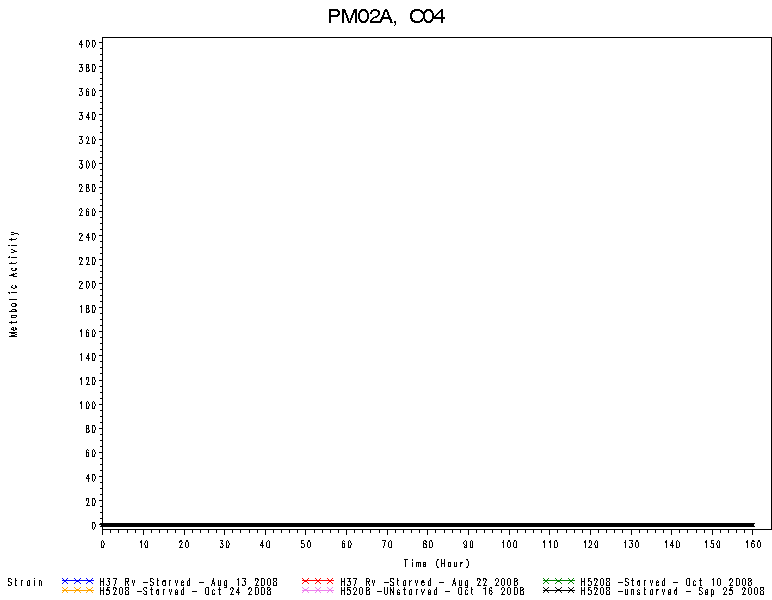

Supplement: Figure S1 — Kinetic curves for all PM plates with Mycobacterium tuberculosis H37Rv and Bj5208 strains. (ZIP) [file pone.0052673.s001.zip › suppl fig 1G H37Rv and Bj5208/Plate02A/pm02ac04.gif]

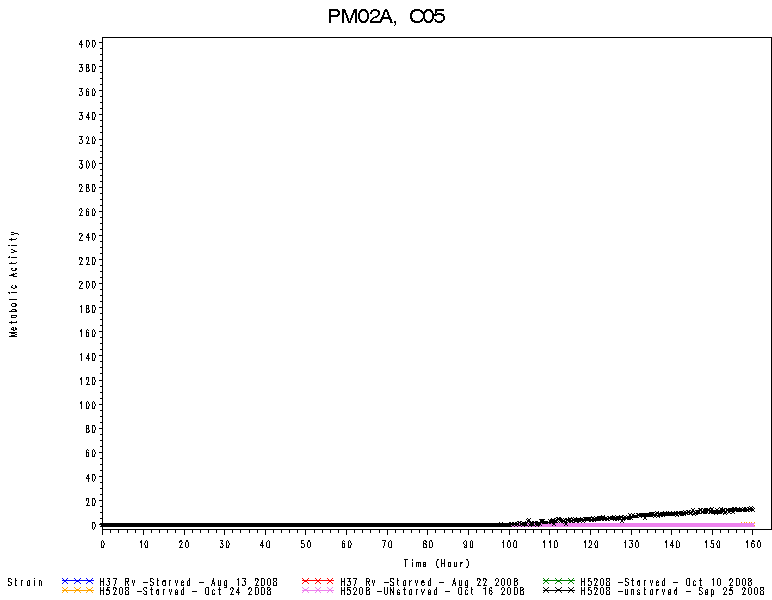

Supplement: Figure S1 — Kinetic curves for all PM plates with Mycobacterium tuberculosis H37Rv and Bj5208 strains. (ZIP) [file pone.0052673.s001.zip › suppl fig 1G H37Rv and Bj5208/Plate02A/pm02ac05.gif]

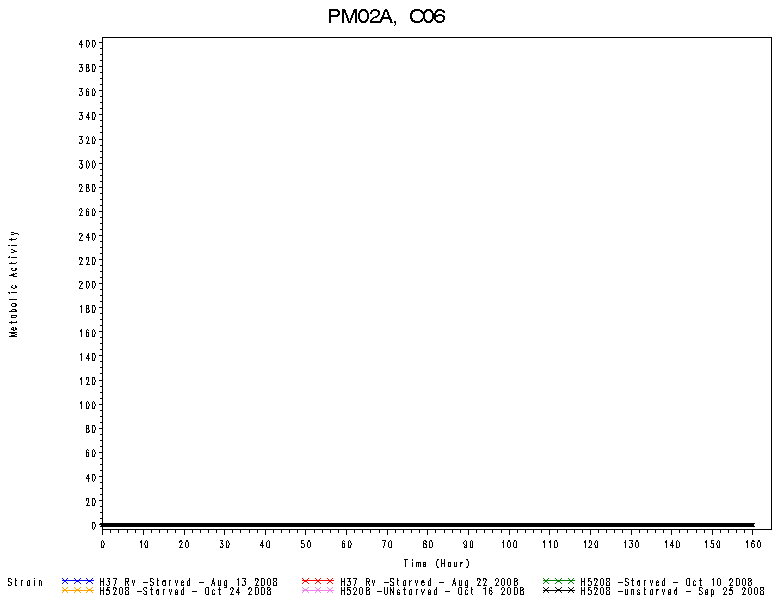

Supplement: Figure S1 — Kinetic curves for all PM plates with Mycobacterium tuberculosis H37Rv and Bj5208 strains. (ZIP) [file pone.0052673.s001.zip › suppl fig 1G H37Rv and Bj5208/Plate02A/pm02ac06.gif]

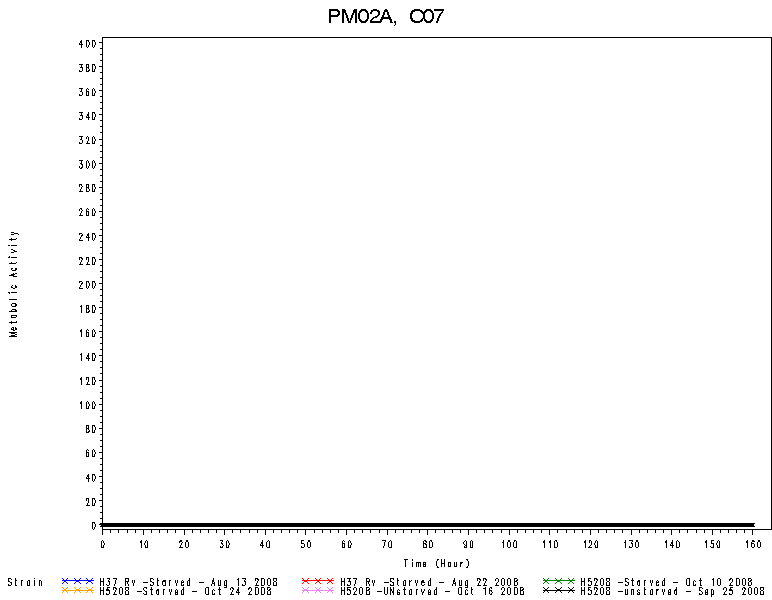

Supplement: Figure S1 — Kinetic curves for all PM plates with Mycobacterium tuberculosis H37Rv and Bj5208 strains. (ZIP) [file pone.0052673.s001.zip › suppl fig 1G H37Rv and Bj5208/Plate02A/pm02ac07.gif]

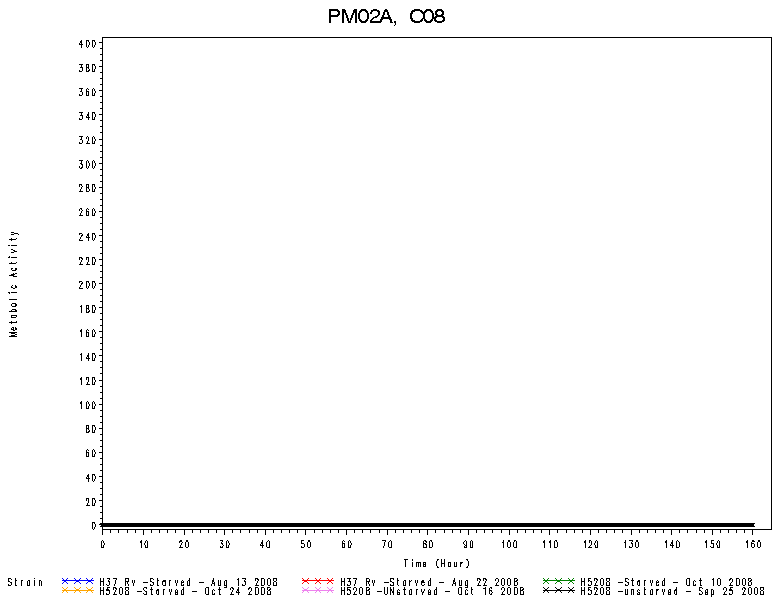

Supplement: Figure S1 — Kinetic curves for all PM plates with Mycobacterium tuberculosis H37Rv and Bj5208 strains. (ZIP) [file pone.0052673.s001.zip › suppl fig 1G H37Rv and Bj5208/Plate02A/pm02ac08.gif]

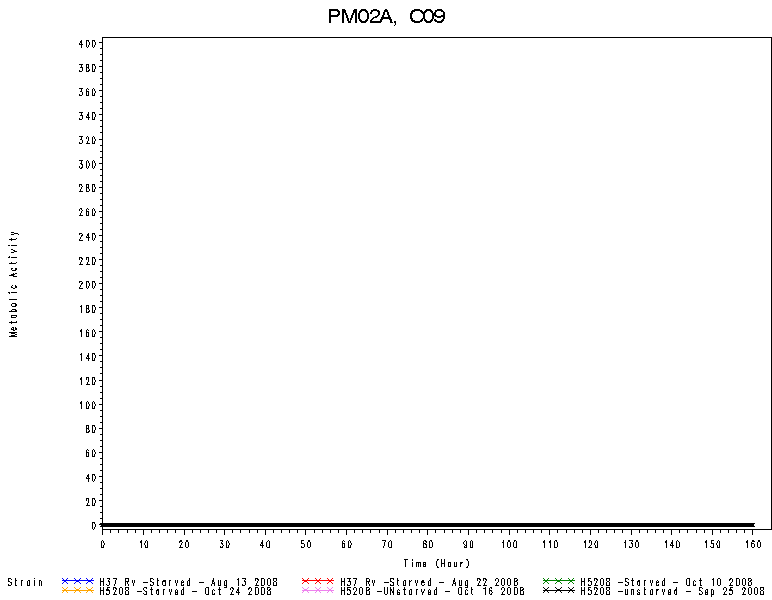

Supplement: Figure S1 — Kinetic curves for all PM plates with Mycobacterium tuberculosis H37Rv and Bj5208 strains. (ZIP) [file pone.0052673.s001.zip › suppl fig 1G H37Rv and Bj5208/Plate02A/pm02ac09.gif]

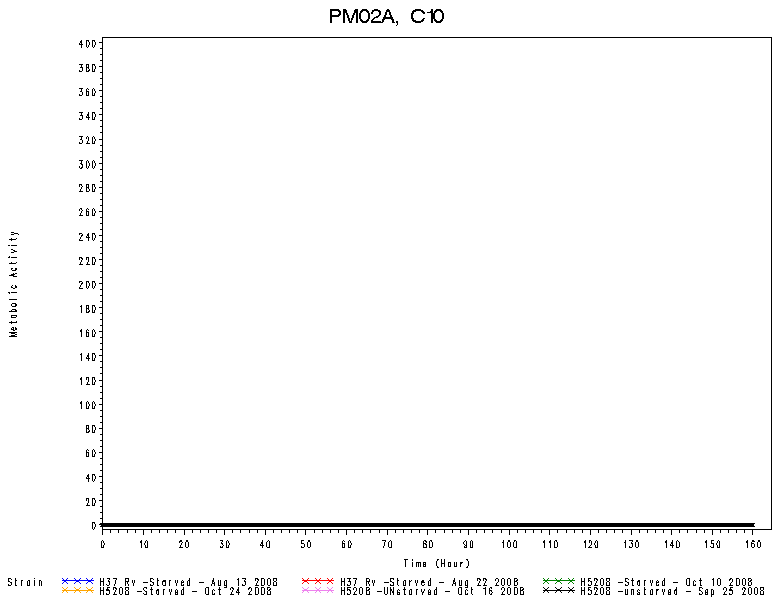

Supplement: Figure S1 — Kinetic curves for all PM plates with Mycobacterium tuberculosis H37Rv and Bj5208 strains. (ZIP) [file pone.0052673.s001.zip › suppl fig 1G H37Rv and Bj5208/Plate02A/pm02ac10.gif]

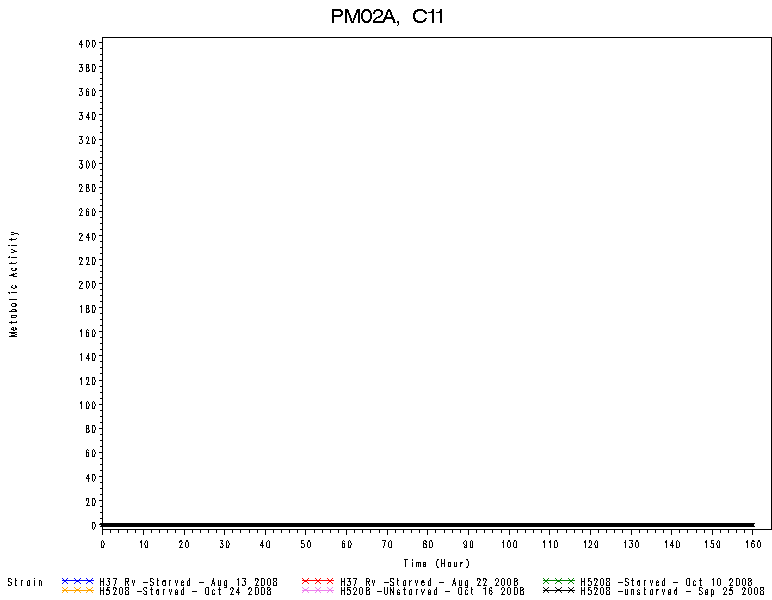

Supplement: Figure S1 — Kinetic curves for all PM plates with Mycobacterium tuberculosis H37Rv and Bj5208 strains. (ZIP) [file pone.0052673.s001.zip › suppl fig 1G H37Rv and Bj5208/Plate02A/pm02ac11.gif]

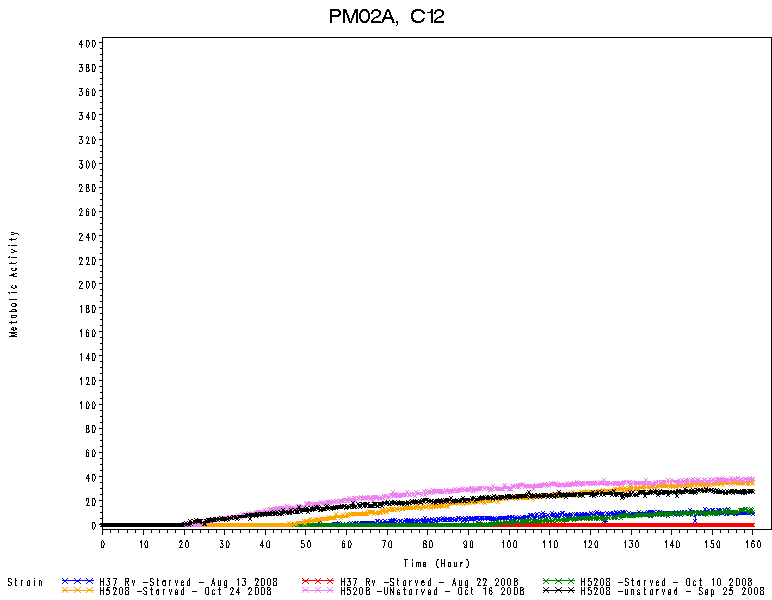

Supplement: Figure S1 — Kinetic curves for all PM plates with Mycobacterium tuberculosis H37Rv and Bj5208 strains. (ZIP) [file pone.0052673.s001.zip › suppl fig 1G H37Rv and Bj5208/Plate02A/pm02ac12.gif]

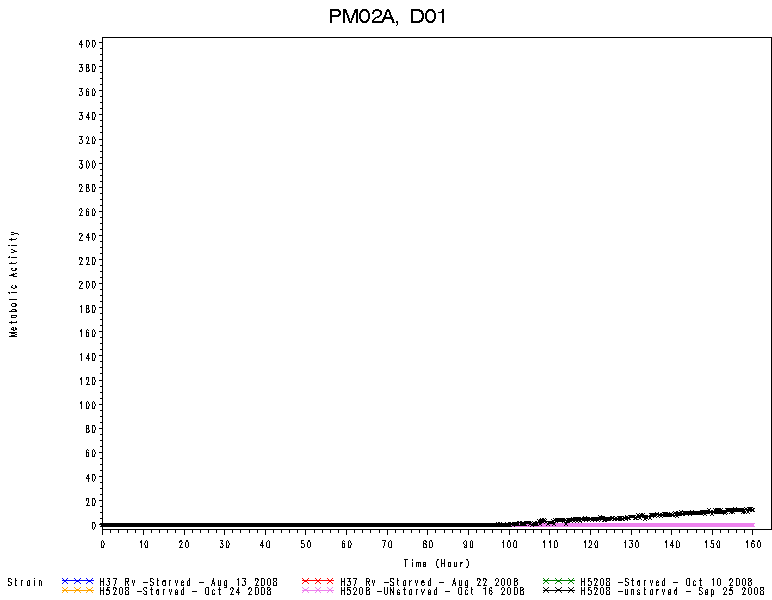

Supplement: Figure S1 — Kinetic curves for all PM plates with Mycobacterium tuberculosis H37Rv and Bj5208 strains. (ZIP) [file pone.0052673.s001.zip › suppl fig 1G H37Rv and Bj5208/Plate02A/pm02ad01.gif]

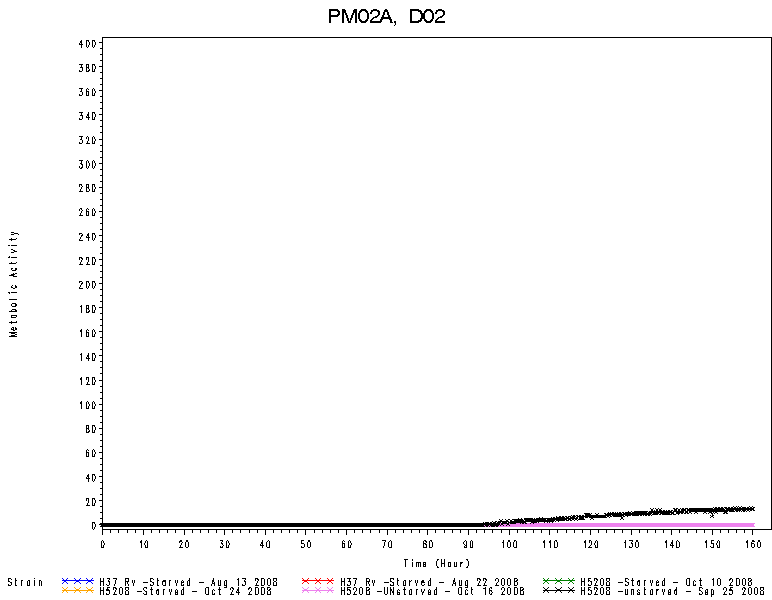

Supplement: Figure S1 — Kinetic curves for all PM plates with Mycobacterium tuberculosis H37Rv and Bj5208 strains. (ZIP) [file pone.0052673.s001.zip › suppl fig 1G H37Rv and Bj5208/Plate02A/pm02ad02.gif]

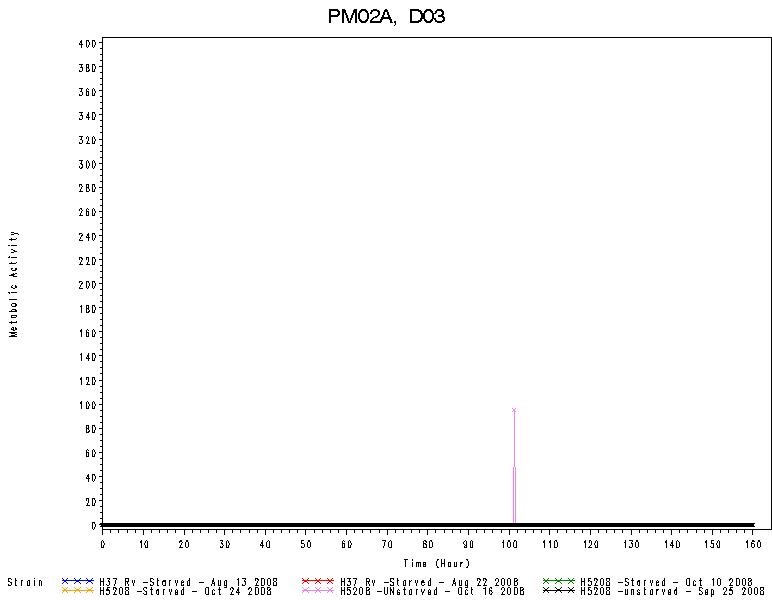

Supplement: Figure S1 — Kinetic curves for all PM plates with Mycobacterium tuberculosis H37Rv and Bj5208 strains. (ZIP) [file pone.0052673.s001.zip › suppl fig 1G H37Rv and Bj5208/Plate02A/pm02ad03.gif]

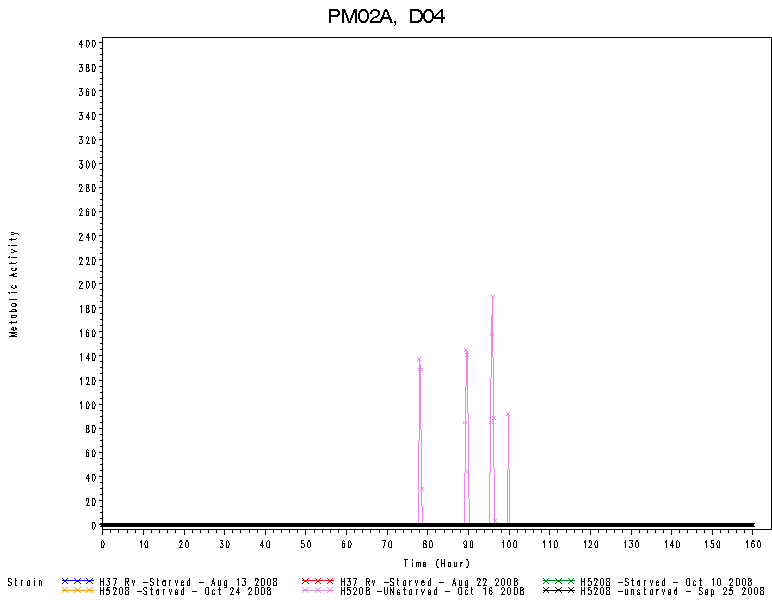

Supplement: Figure S1 — Kinetic curves for all PM plates with Mycobacterium tuberculosis H37Rv and Bj5208 strains. (ZIP) [file pone.0052673.s001.zip › suppl fig 1G H37Rv and Bj5208/Plate02A/pm02ad04.gif]

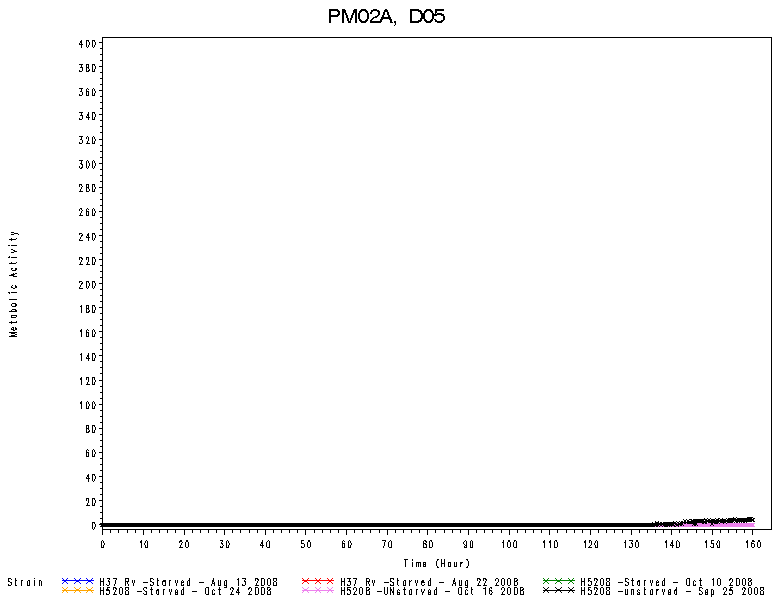

Supplement: Figure S1 — Kinetic curves for all PM plates with Mycobacterium tuberculosis H37Rv and Bj5208 strains. (ZIP) [file pone.0052673.s001.zip › suppl fig 1G H37Rv and Bj5208/Plate02A/pm02ad05.gif]

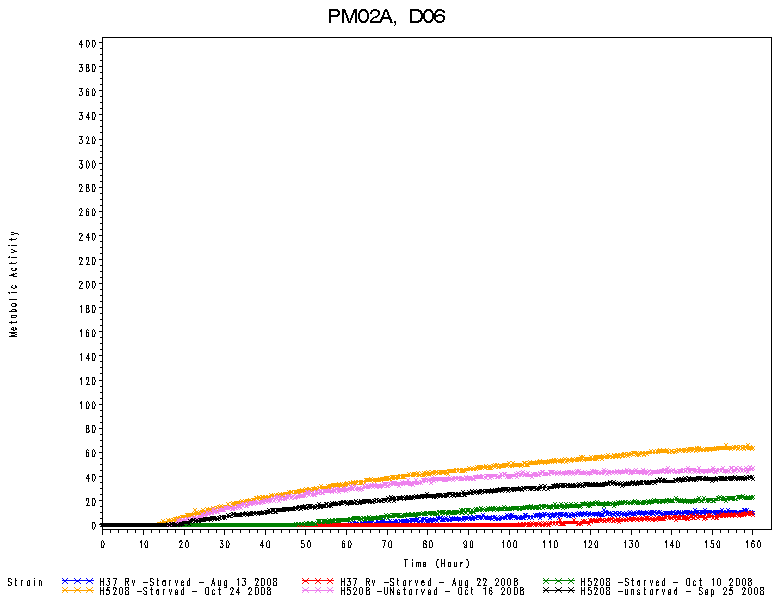

Supplement: Figure S1 — Kinetic curves for all PM plates with Mycobacterium tuberculosis H37Rv and Bj5208 strains. (ZIP) [file pone.0052673.s001.zip › suppl fig 1G H37Rv and Bj5208/Plate02A/pm02ad06.gif]

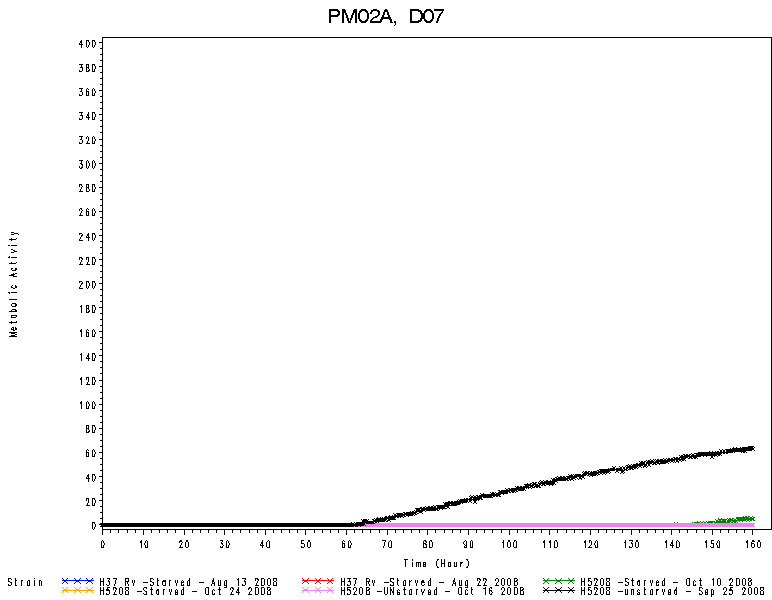

Supplement: Figure S1 — Kinetic curves for all PM plates with Mycobacterium tuberculosis H37Rv and Bj5208 strains. (ZIP) [file pone.0052673.s001.zip › suppl fig 1G H37Rv and Bj5208/Plate02A/pm02ad07.gif]

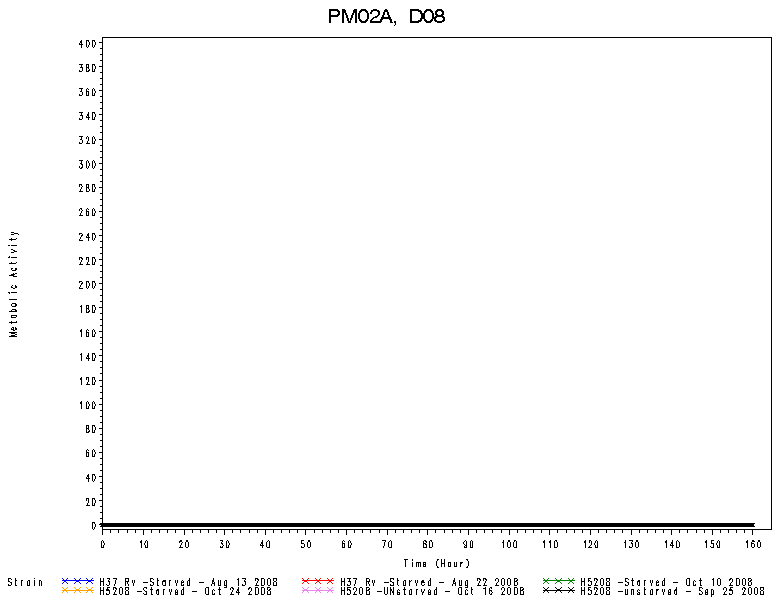

Supplement: Figure S1 — Kinetic curves for all PM plates with Mycobacterium tuberculosis H37Rv and Bj5208 strains. (ZIP) [file pone.0052673.s001.zip › suppl fig 1G H37Rv and Bj5208/Plate02A/pm02ad08.gif]

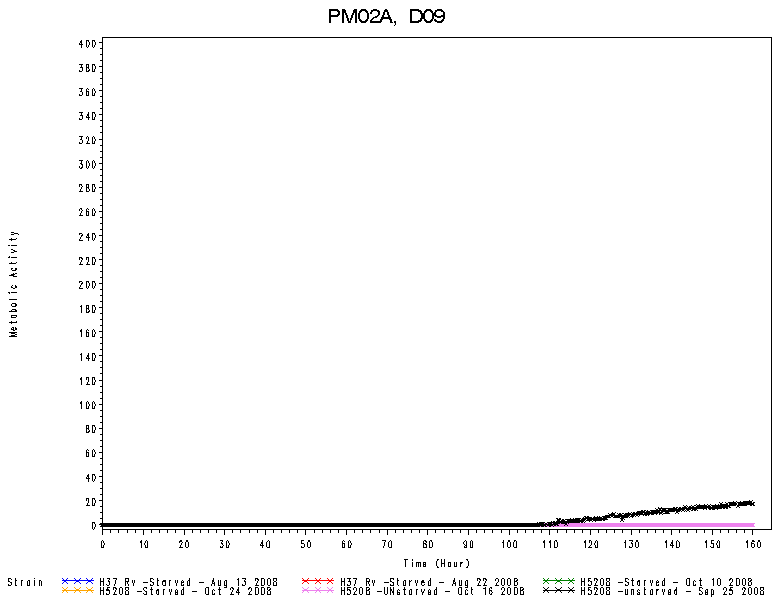

Supplement: Figure S1 — Kinetic curves for all PM plates with Mycobacterium tuberculosis H37Rv and Bj5208 strains. (ZIP) [file pone.0052673.s001.zip › suppl fig 1G H37Rv and Bj5208/Plate02A/pm02ad09.gif]

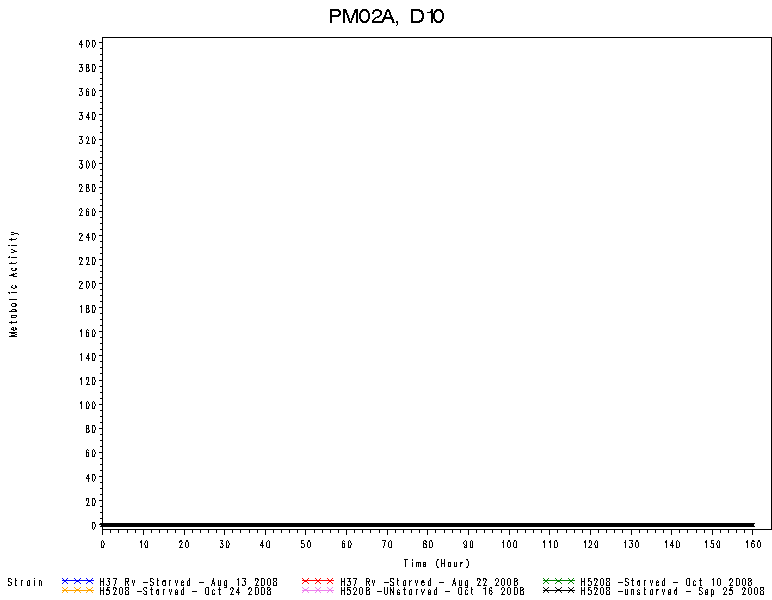

Supplement: Figure S1 — Kinetic curves for all PM plates with Mycobacterium tuberculosis H37Rv and Bj5208 strains. (ZIP) [file pone.0052673.s001.zip › suppl fig 1G H37Rv and Bj5208/Plate02A/pm02ad10.gif]

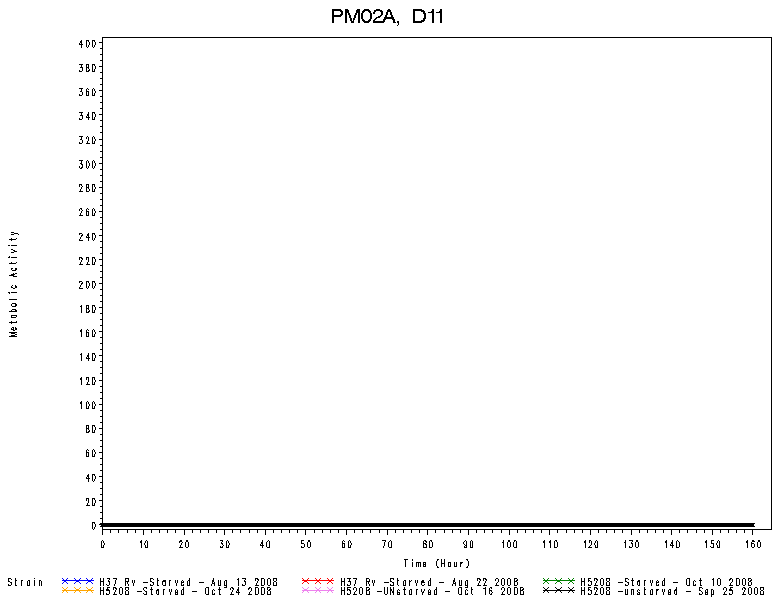

Supplement: Figure S1 — Kinetic curves for all PM plates with Mycobacterium tuberculosis H37Rv and Bj5208 strains. (ZIP) [file pone.0052673.s001.zip › suppl fig 1G H37Rv and Bj5208/Plate02A/pm02ad11.gif]

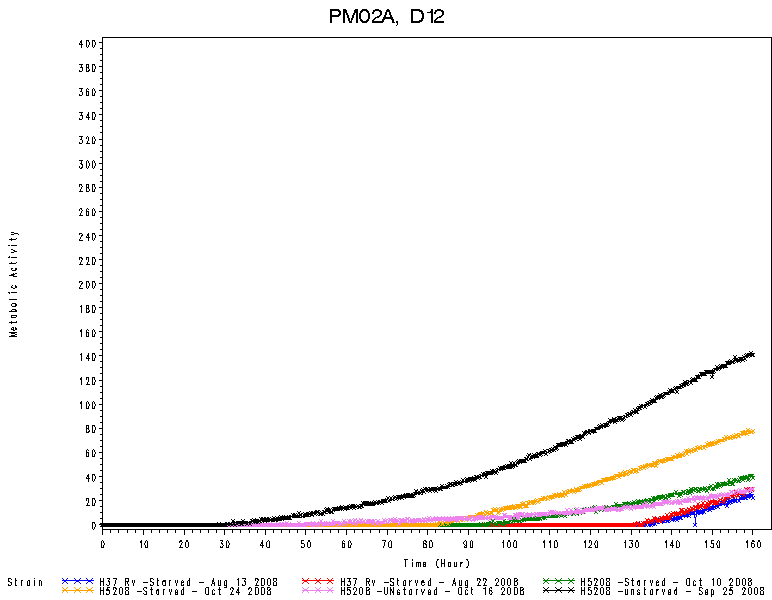

Supplement: Figure S1 — Kinetic curves for all PM plates with Mycobacterium tuberculosis H37Rv and Bj5208 strains. (ZIP) [file pone.0052673.s001.zip › suppl fig 1G H37Rv and Bj5208/Plate02A/pm02ad12.gif]

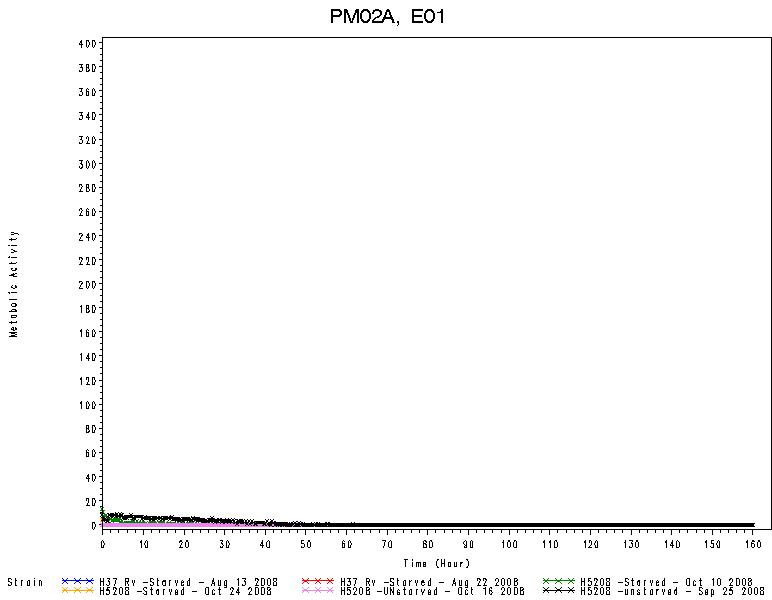

Supplement: Figure S1 — Kinetic curves for all PM plates with Mycobacterium tuberculosis H37Rv and Bj5208 strains. (ZIP) [file pone.0052673.s001.zip › suppl fig 1G H37Rv and Bj5208/Plate02A/pm02ae01.gif]

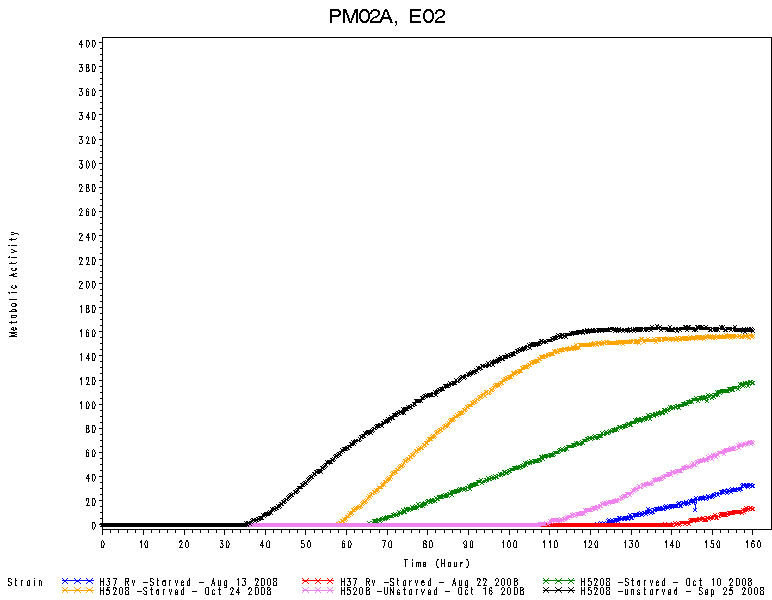

Supplement: Figure S1 — Kinetic curves for all PM plates with Mycobacterium tuberculosis H37Rv and Bj5208 strains. (ZIP) [file pone.0052673.s001.zip › suppl fig 1G H37Rv and Bj5208/Plate02A/pm02ae02.gif]

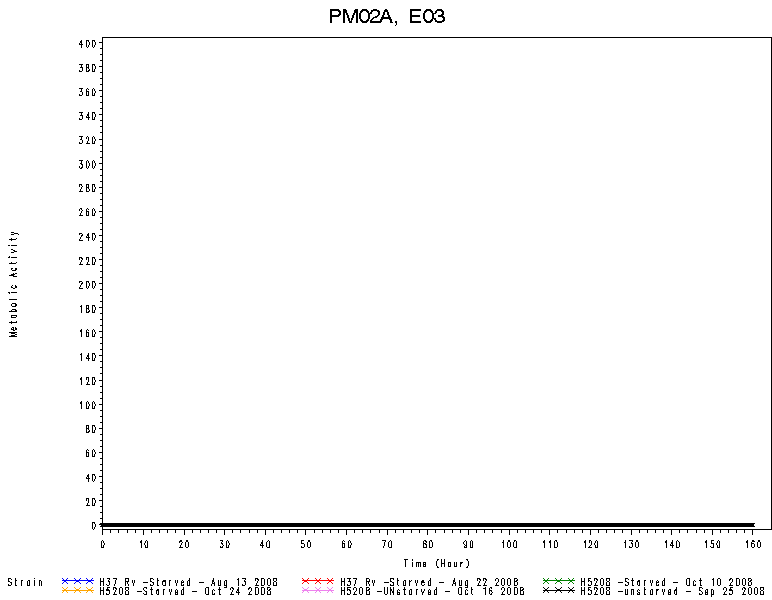

Supplement: Figure S1 — Kinetic curves for all PM plates with Mycobacterium tuberculosis H37Rv and Bj5208 strains. (ZIP) [file pone.0052673.s001.zip › suppl fig 1G H37Rv and Bj5208/Plate02A/pm02ae03.gif]

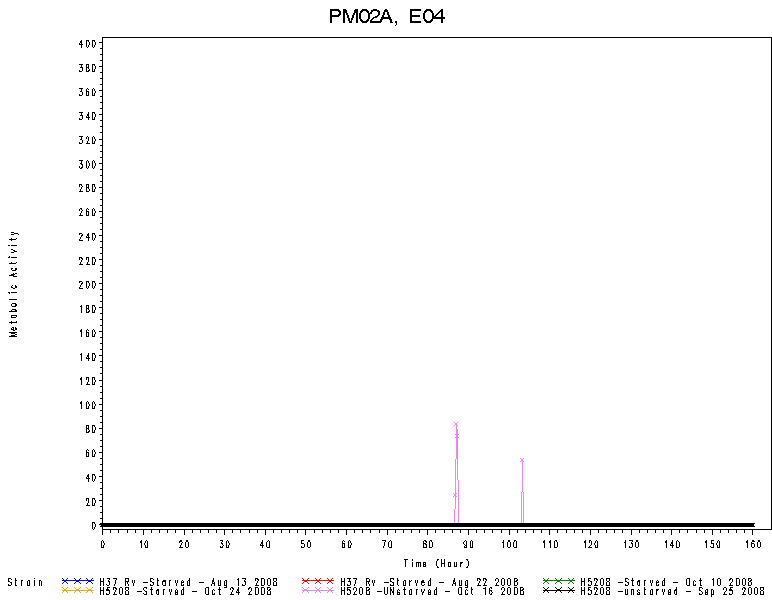

Supplement: Figure S1 — Kinetic curves for all PM plates with Mycobacterium tuberculosis H37Rv and Bj5208 strains. (ZIP) [file pone.0052673.s001.zip › suppl fig 1G H37Rv and Bj5208/Plate02A/pm02ae04.gif]

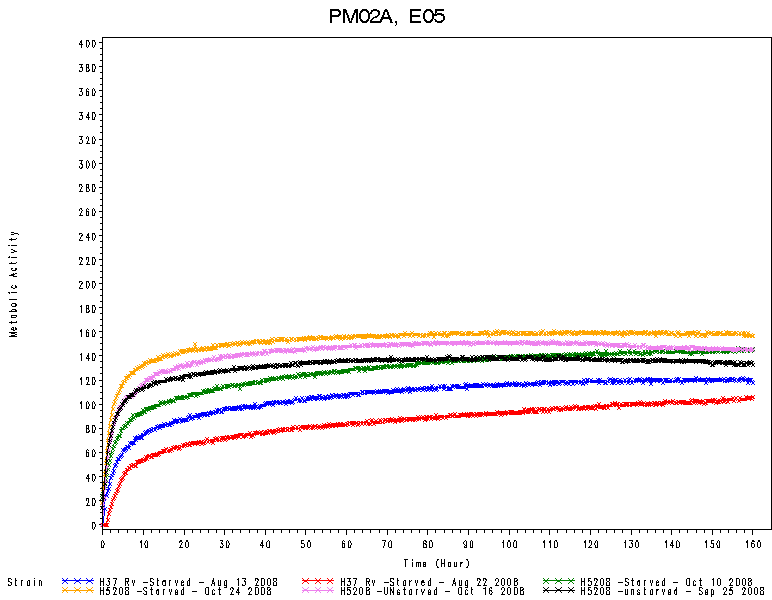

Supplement: Figure S1 — Kinetic curves for all PM plates with Mycobacterium tuberculosis H37Rv and Bj5208 strains. (ZIP) [file pone.0052673.s001.zip › suppl fig 1G H37Rv and Bj5208/Plate02A/pm02ae05.gif]

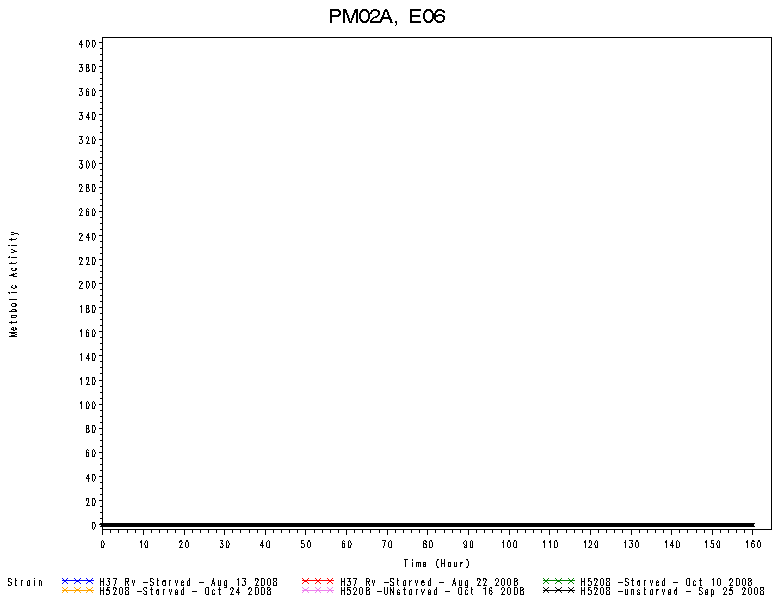

Supplement: Figure S1 — Kinetic curves for all PM plates with Mycobacterium tuberculosis H37Rv and Bj5208 strains. (ZIP) [file pone.0052673.s001.zip › suppl fig 1G H37Rv and Bj5208/Plate02A/pm02ae06.gif]

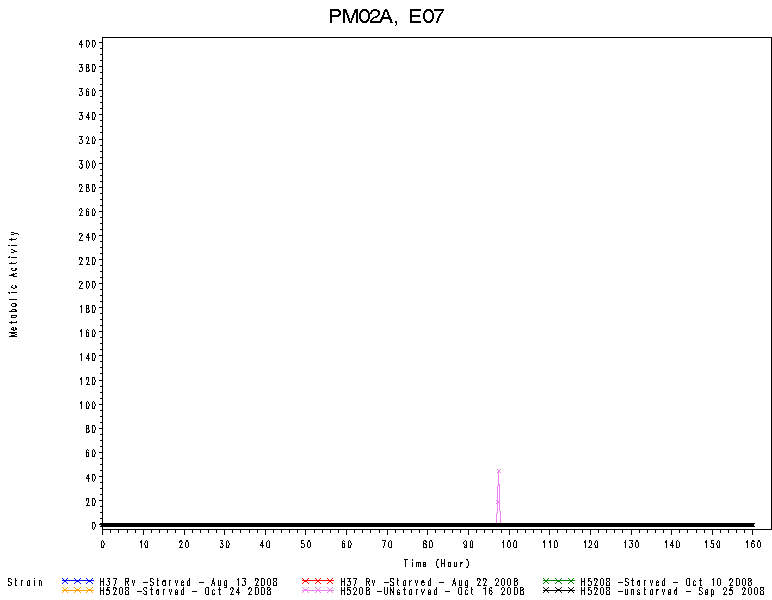

Supplement: Figure S1 — Kinetic curves for all PM plates with Mycobacterium tuberculosis H37Rv and Bj5208 strains. (ZIP) [file pone.0052673.s001.zip › suppl fig 1G H37Rv and Bj5208/Plate02A/pm02ae07.gif]

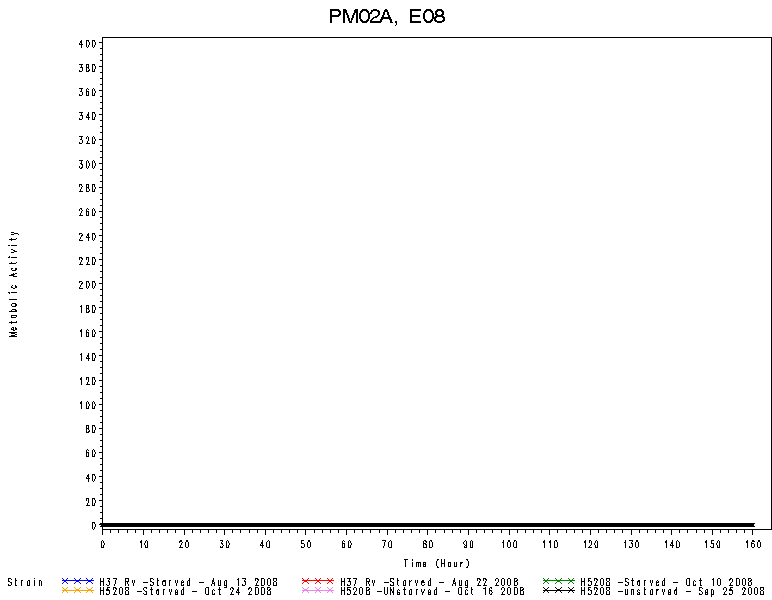

Supplement: Figure S1 — Kinetic curves for all PM plates with Mycobacterium tuberculosis H37Rv and Bj5208 strains. (ZIP) [file pone.0052673.s001.zip › suppl fig 1G H37Rv and Bj5208/Plate02A/pm02ae08.gif]

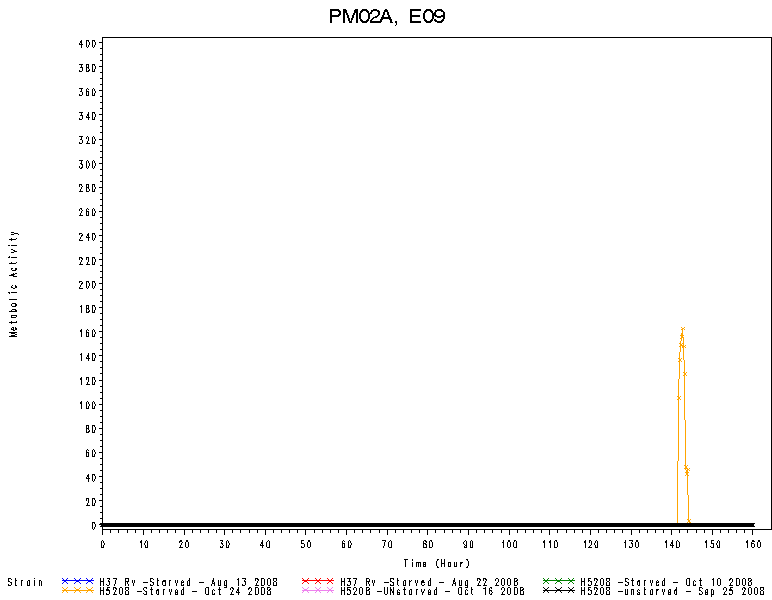

Supplement: Figure S1 — Kinetic curves for all PM plates with Mycobacterium tuberculosis H37Rv and Bj5208 strains. (ZIP) [file pone.0052673.s001.zip › suppl fig 1G H37Rv and Bj5208/Plate02A/pm02ae09.gif]

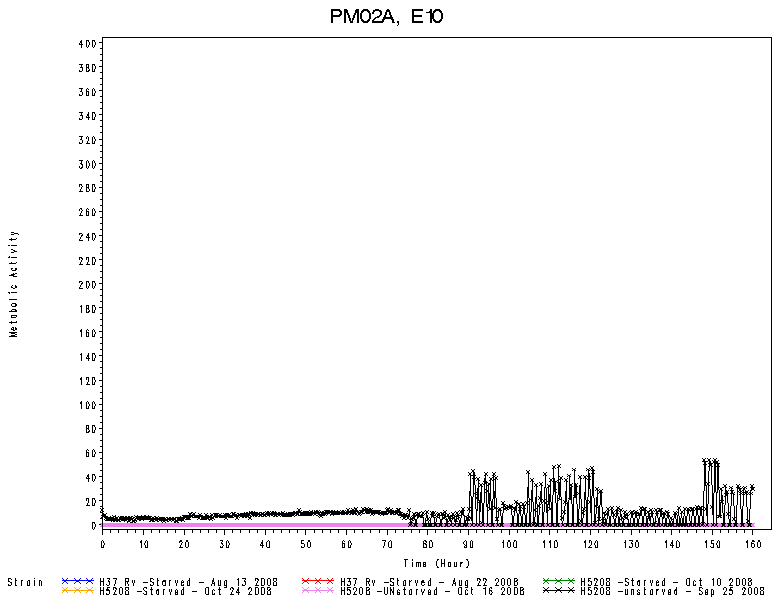

Supplement: Figure S1 — Kinetic curves for all PM plates with Mycobacterium tuberculosis H37Rv and Bj5208 strains. (ZIP) [file pone.0052673.s001.zip › suppl fig 1G H37Rv and Bj5208/Plate02A/pm02ae10.gif]

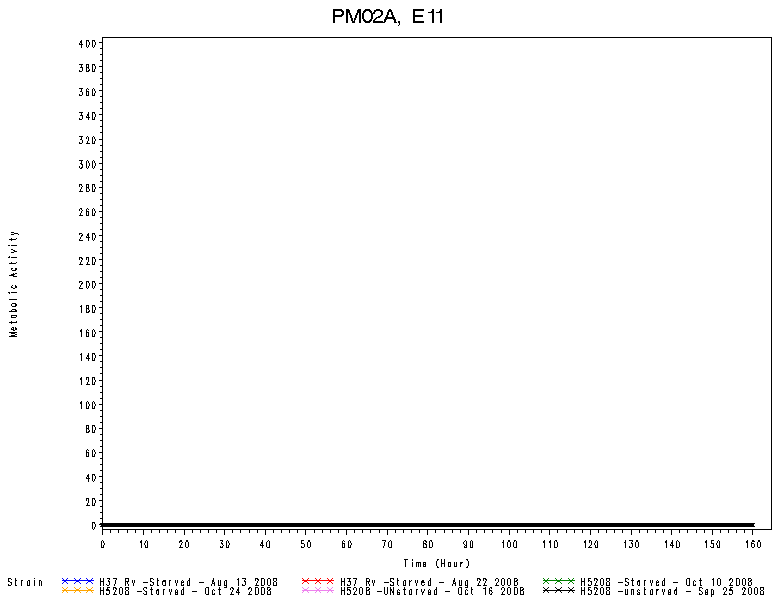

Supplement: Figure S1 — Kinetic curves for all PM plates with Mycobacterium tuberculosis H37Rv and Bj5208 strains. (ZIP) [file pone.0052673.s001.zip › suppl fig 1G H37Rv and Bj5208/Plate02A/pm02ae11.gif]

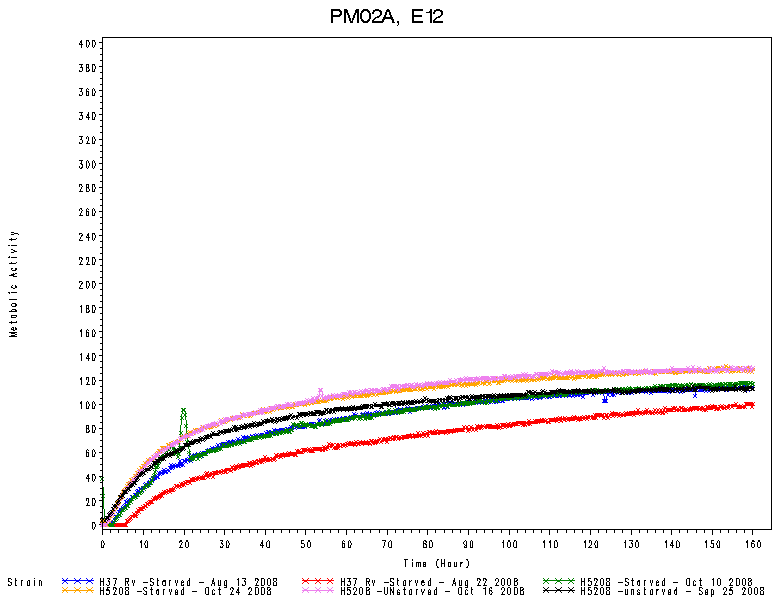

Supplement: Figure S1 — Kinetic curves for all PM plates with Mycobacterium tuberculosis H37Rv and Bj5208 strains. (ZIP) [file pone.0052673.s001.zip › suppl fig 1G H37Rv and Bj5208/Plate02A/pm02ae12.gif]

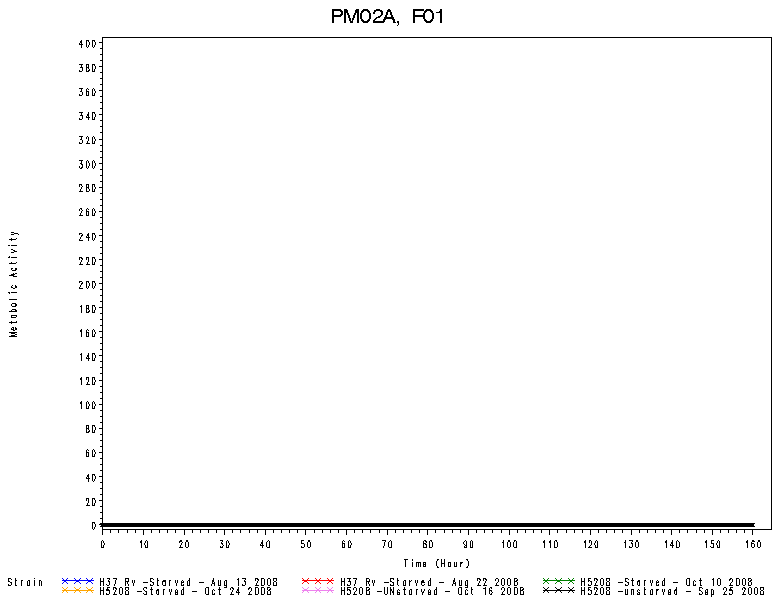

Supplement: Figure S1 — Kinetic curves for all PM plates with Mycobacterium tuberculosis H37Rv and Bj5208 strains. (ZIP) [file pone.0052673.s001.zip › suppl fig 1G H37Rv and Bj5208/Plate02A/pm02af01.gif]

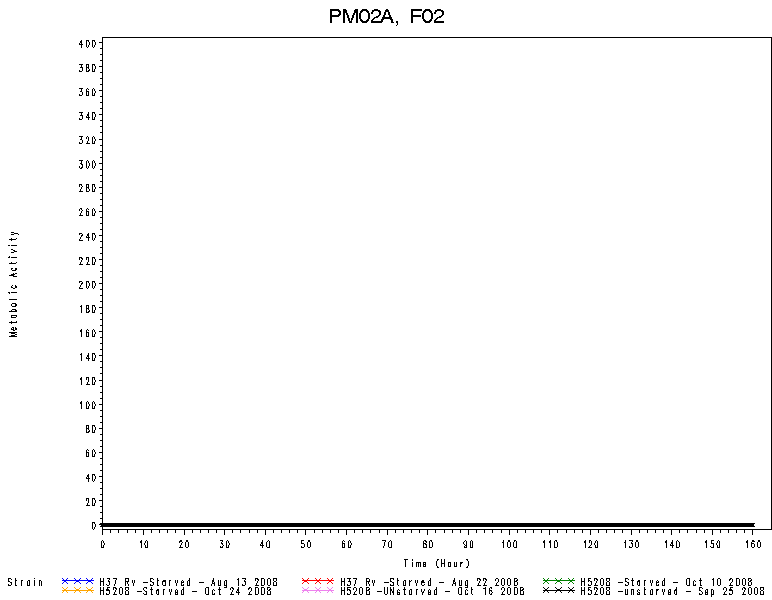

Supplement: Figure S1 — Kinetic curves for all PM plates with Mycobacterium tuberculosis H37Rv and Bj5208 strains. (ZIP) [file pone.0052673.s001.zip › suppl fig 1G H37Rv and Bj5208/Plate02A/pm02af02.gif]

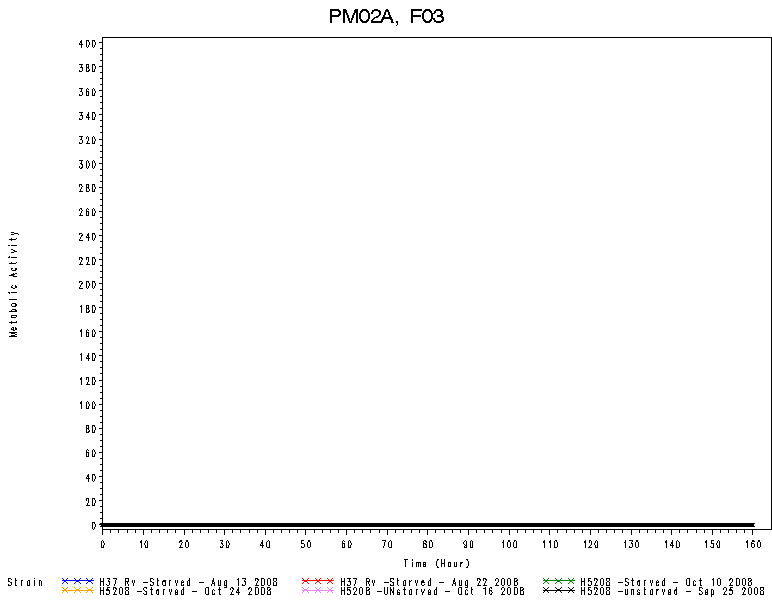

Supplement: Figure S1 — Kinetic curves for all PM plates with Mycobacterium tuberculosis H37Rv and Bj5208 strains. (ZIP) [file pone.0052673.s001.zip › suppl fig 1G H37Rv and Bj5208/Plate02A/pm02af03.gif]

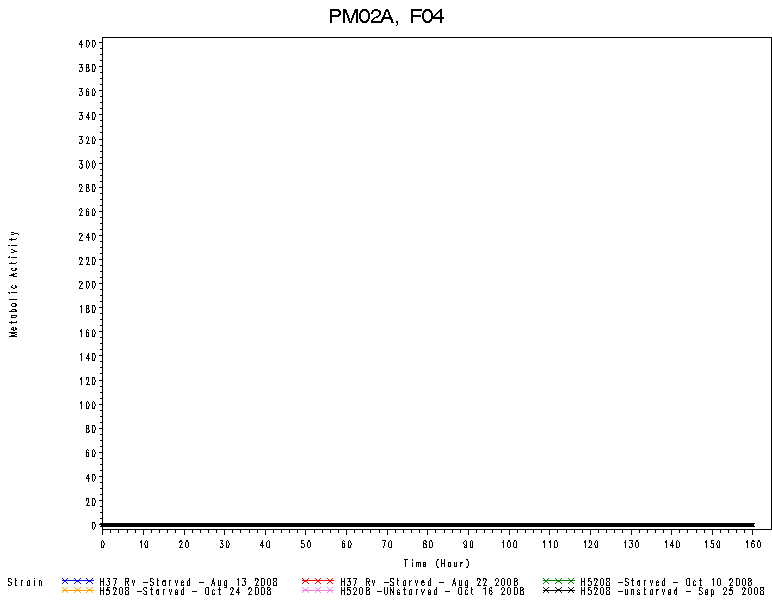

Supplement: Figure S1 — Kinetic curves for all PM plates with Mycobacterium tuberculosis H37Rv and Bj5208 strains. (ZIP) [file pone.0052673.s001.zip › suppl fig 1G H37Rv and Bj5208/Plate02A/pm02af04.gif]

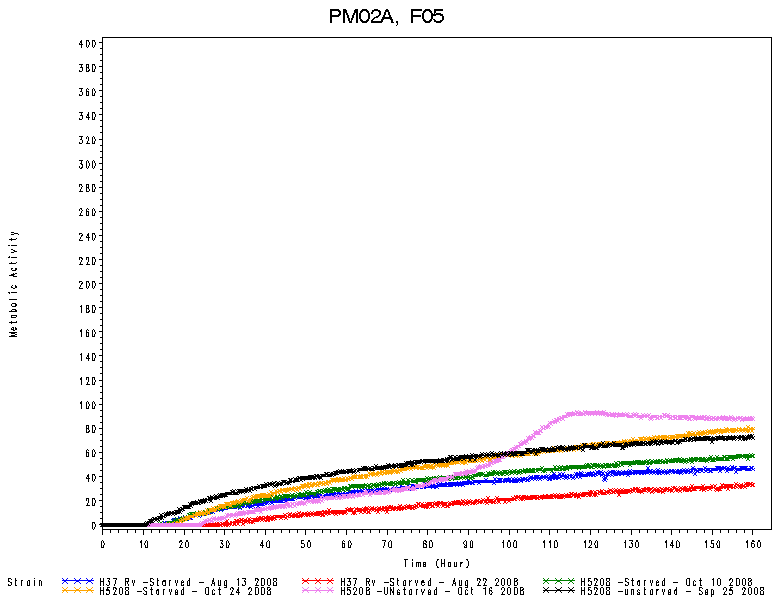

Supplement: Figure S1 — Kinetic curves for all PM plates with Mycobacterium tuberculosis H37Rv and Bj5208 strains. (ZIP) [file pone.0052673.s001.zip › suppl fig 1G H37Rv and Bj5208/Plate02A/pm02af05.gif]

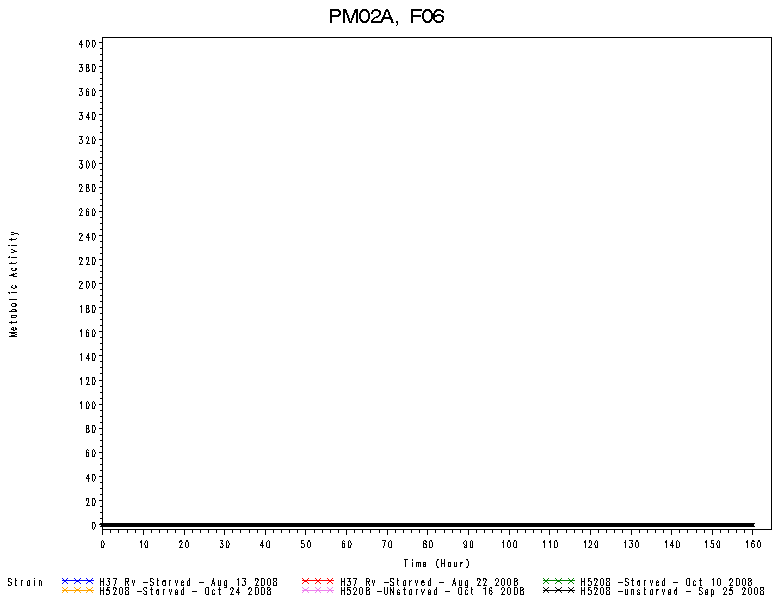

Supplement: Figure S1 — Kinetic curves for all PM plates with Mycobacterium tuberculosis H37Rv and Bj5208 strains. (ZIP) [file pone.0052673.s001.zip › suppl fig 1G H37Rv and Bj5208/Plate02A/pm02af06.gif]

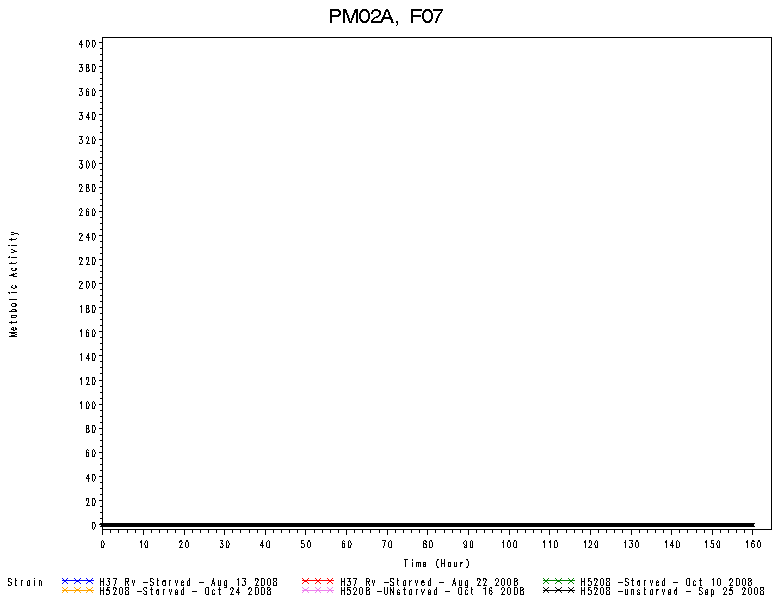

Supplement: Figure S1 — Kinetic curves for all PM plates with Mycobacterium tuberculosis H37Rv and Bj5208 strains. (ZIP) [file pone.0052673.s001.zip › suppl fig 1G H37Rv and Bj5208/Plate02A/pm02af07.gif]

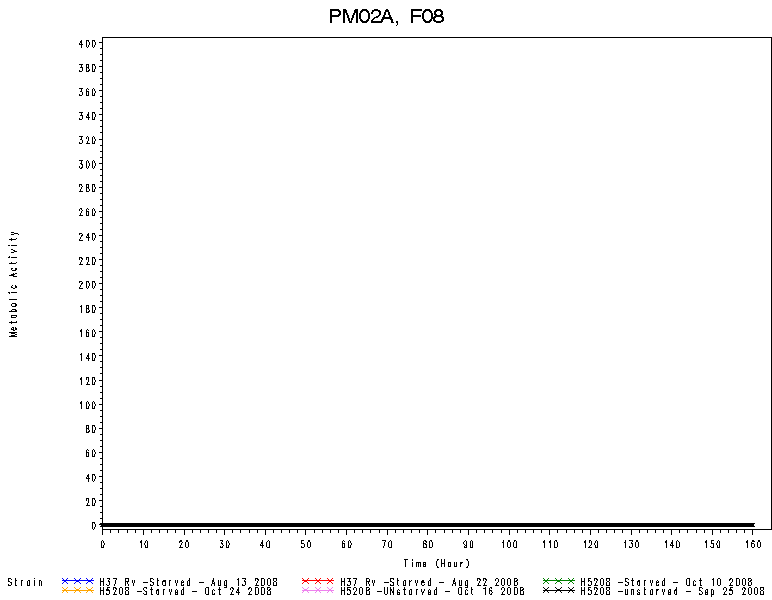

Supplement: Figure S1 — Kinetic curves for all PM plates with Mycobacterium tuberculosis H37Rv and Bj5208 strains. (ZIP) [file pone.0052673.s001.zip › suppl fig 1G H37Rv and Bj5208/Plate02A/pm02af08.gif]

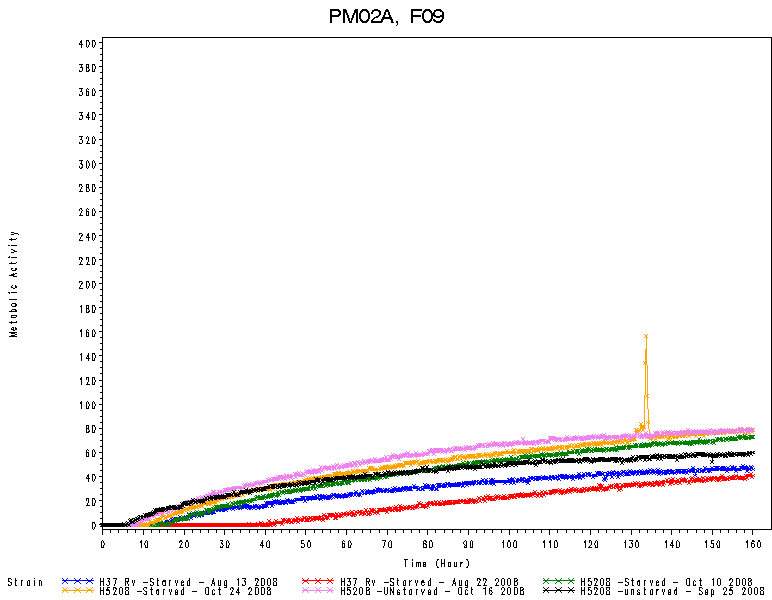

Supplement: Figure S1 — Kinetic curves for all PM plates with Mycobacterium tuberculosis H37Rv and Bj5208 strains. (ZIP) [file pone.0052673.s001.zip › suppl fig 1G H37Rv and Bj5208/Plate02A/pm02af09.gif]

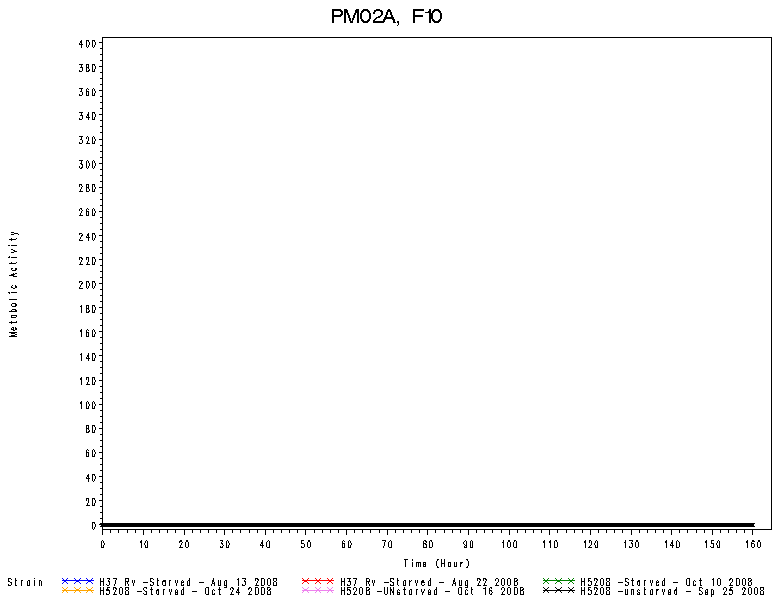

Supplement: Figure S1 — Kinetic curves for all PM plates with Mycobacterium tuberculosis H37Rv and Bj5208 strains. (ZIP) [file pone.0052673.s001.zip › suppl fig 1G H37Rv and Bj5208/Plate02A/pm02af10.gif]

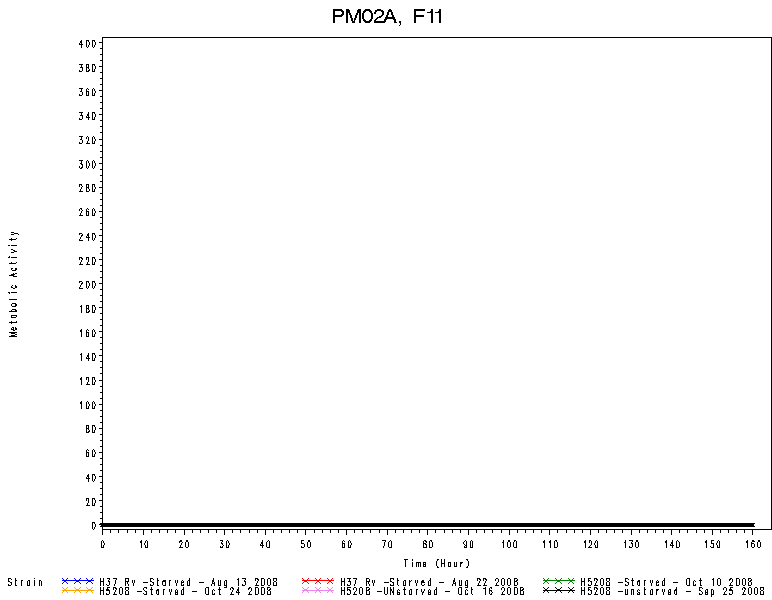

Supplement: Figure S1 — Kinetic curves for all PM plates with Mycobacterium tuberculosis H37Rv and Bj5208 strains. (ZIP) [file pone.0052673.s001.zip › suppl fig 1G H37Rv and Bj5208/Plate02A/pm02af11.gif]

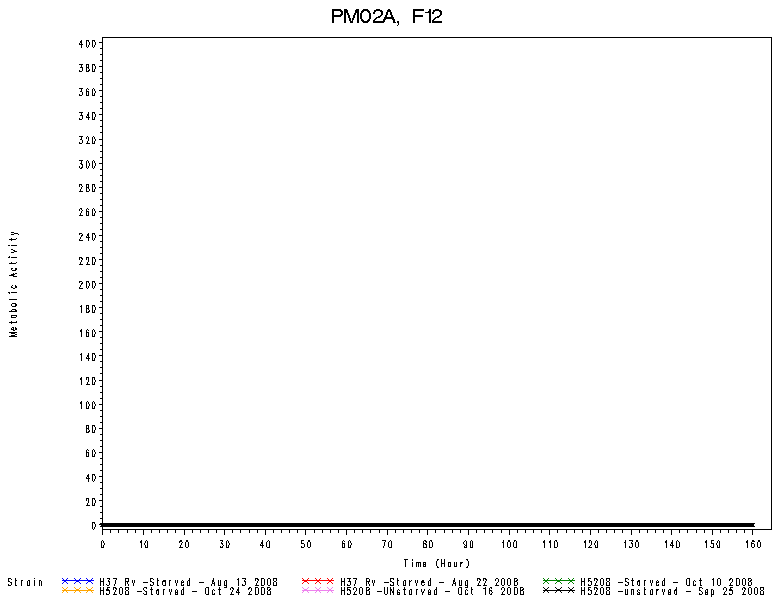

Supplement: Figure S1 — Kinetic curves for all PM plates with Mycobacterium tuberculosis H37Rv and Bj5208 strains. (ZIP) [file pone.0052673.s001.zip › suppl fig 1G H37Rv and Bj5208/Plate02A/pm02af12.gif]

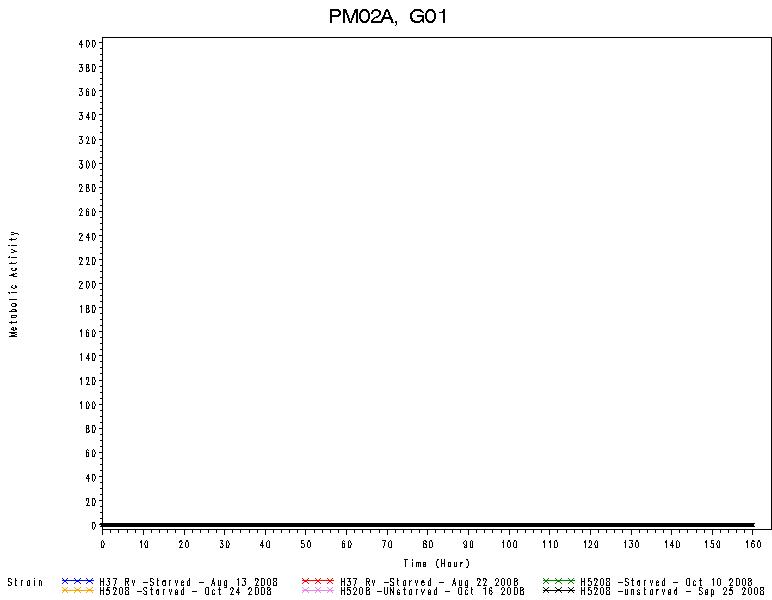

Supplement: Figure S1 — Kinetic curves for all PM plates with Mycobacterium tuberculosis H37Rv and Bj5208 strains. (ZIP) [file pone.0052673.s001.zip › suppl fig 1G H37Rv and Bj5208/Plate02A/pm02ag01.gif]

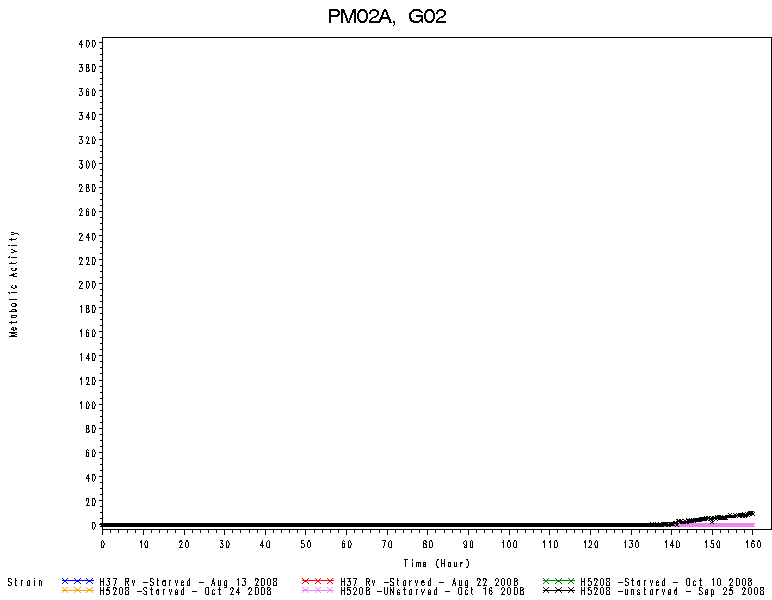

Supplement: Figure S1 — Kinetic curves for all PM plates with Mycobacterium tuberculosis H37Rv and Bj5208 strains. (ZIP) [file pone.0052673.s001.zip › suppl fig 1G H37Rv and Bj5208/Plate02A/pm02ag02.gif]

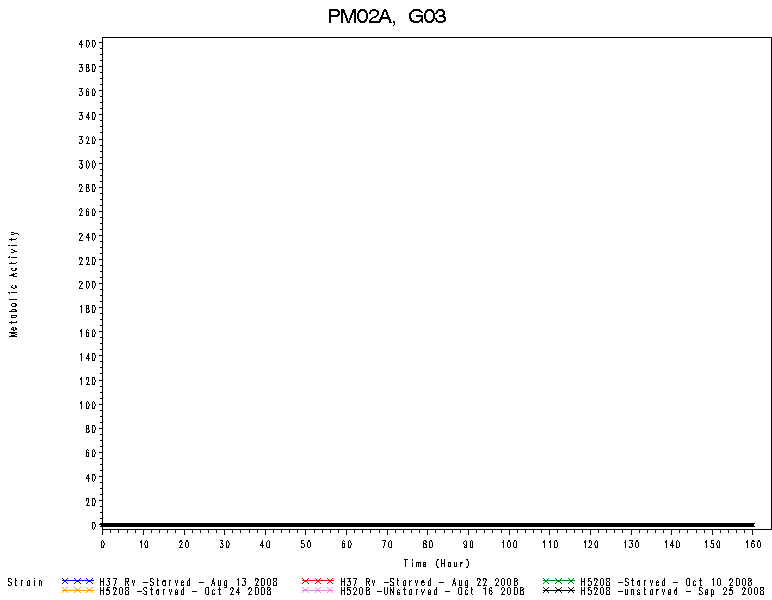

Supplement: Figure S1 — Kinetic curves for all PM plates with Mycobacterium tuberculosis H37Rv and Bj5208 strains. (ZIP) [file pone.0052673.s001.zip › suppl fig 1G H37Rv and Bj5208/Plate02A/pm02ag03.gif]

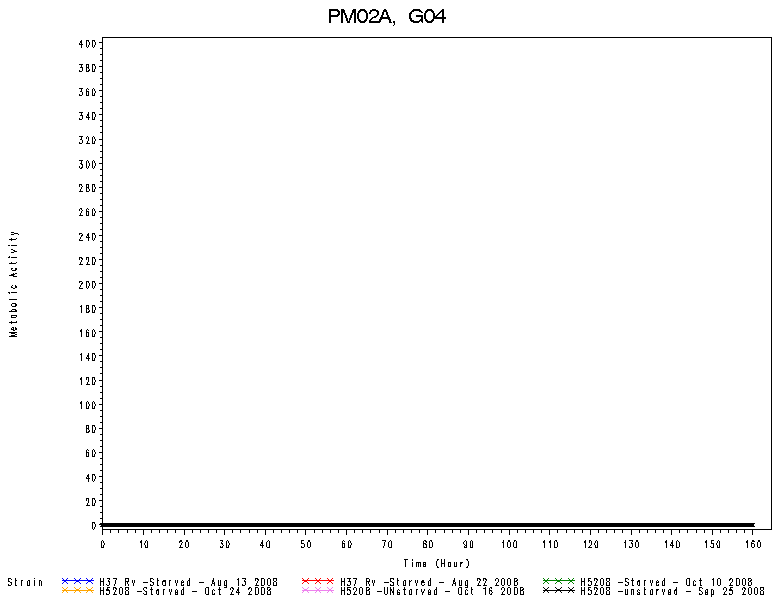

Supplement: Figure S1 — Kinetic curves for all PM plates with Mycobacterium tuberculosis H37Rv and Bj5208 strains. (ZIP) [file pone.0052673.s001.zip › suppl fig 1G H37Rv and Bj5208/Plate02A/pm02ag04.gif]

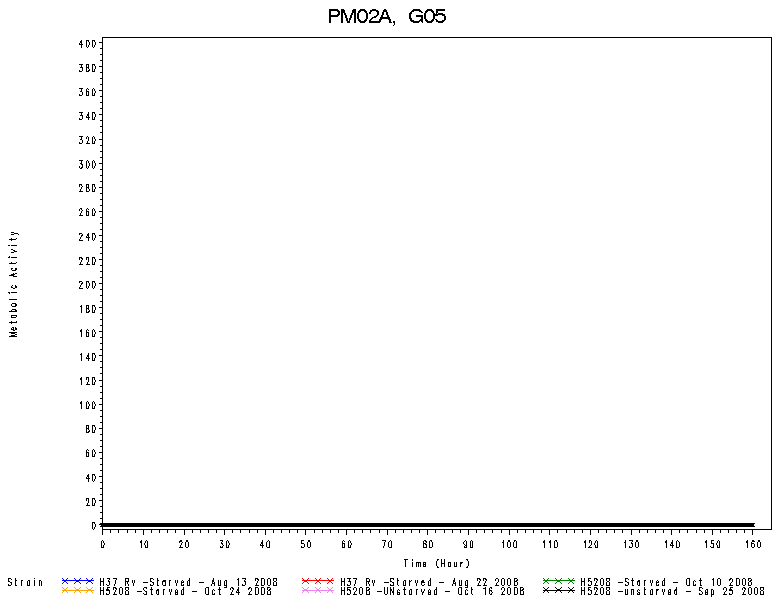

Supplement: Figure S1 — Kinetic curves for all PM plates with Mycobacterium tuberculosis H37Rv and Bj5208 strains. (ZIP) [file pone.0052673.s001.zip › suppl fig 1G H37Rv and Bj5208/Plate02A/pm02ag05.gif]

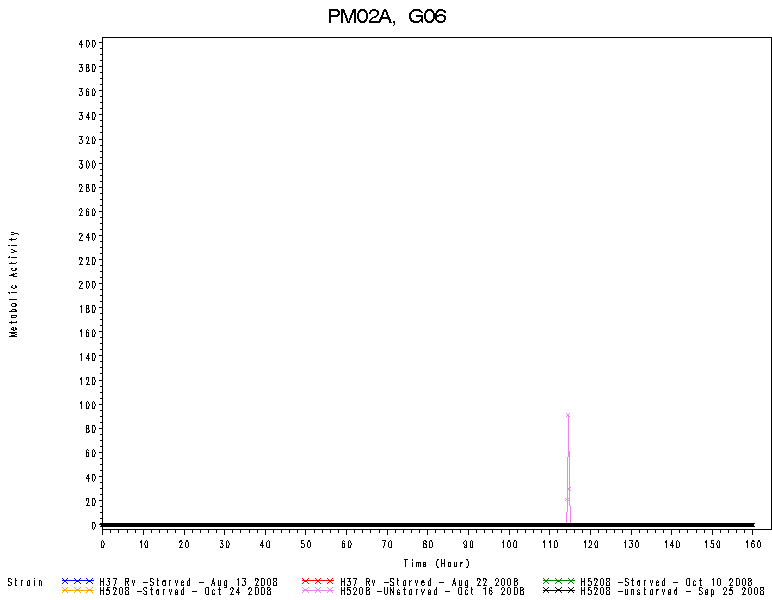

Supplement: Figure S1 — Kinetic curves for all PM plates with Mycobacterium tuberculosis H37Rv and Bj5208 strains. (ZIP) [file pone.0052673.s001.zip › suppl fig 1G H37Rv and Bj5208/Plate02A/pm02ag06.gif]

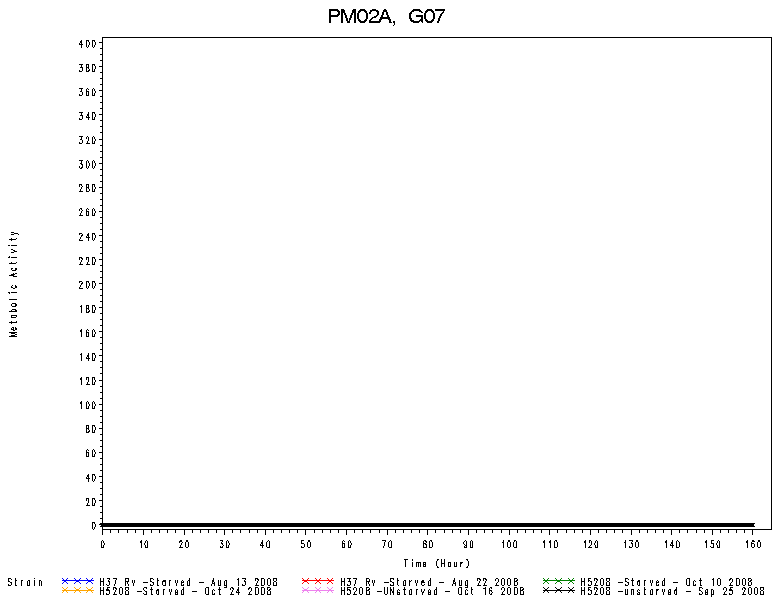

Supplement: Figure S1 — Kinetic curves for all PM plates with Mycobacterium tuberculosis H37Rv and Bj5208 strains. (ZIP) [file pone.0052673.s001.zip › suppl fig 1G H37Rv and Bj5208/Plate02A/pm02ag07.gif]

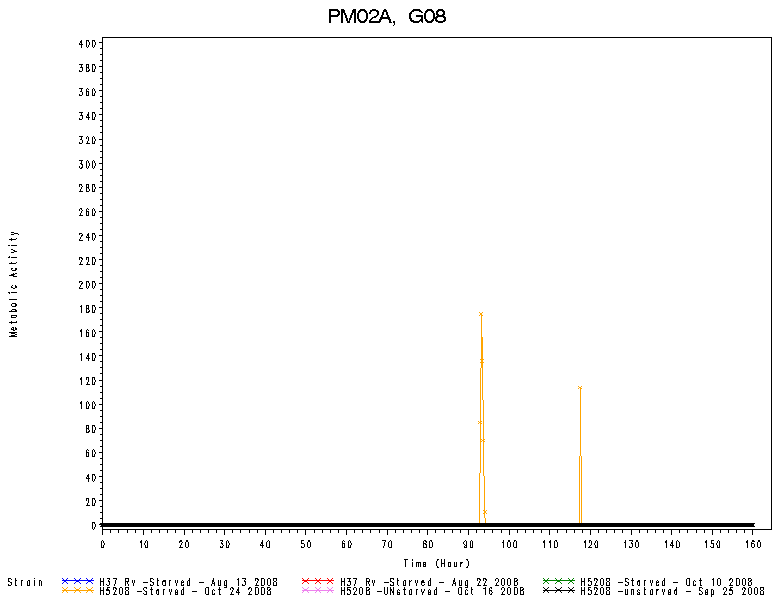

Supplement: Figure S1 — Kinetic curves for all PM plates with Mycobacterium tuberculosis H37Rv and Bj5208 strains. (ZIP) [file pone.0052673.s001.zip › suppl fig 1G H37Rv and Bj5208/Plate02A/pm02ag08.gif]

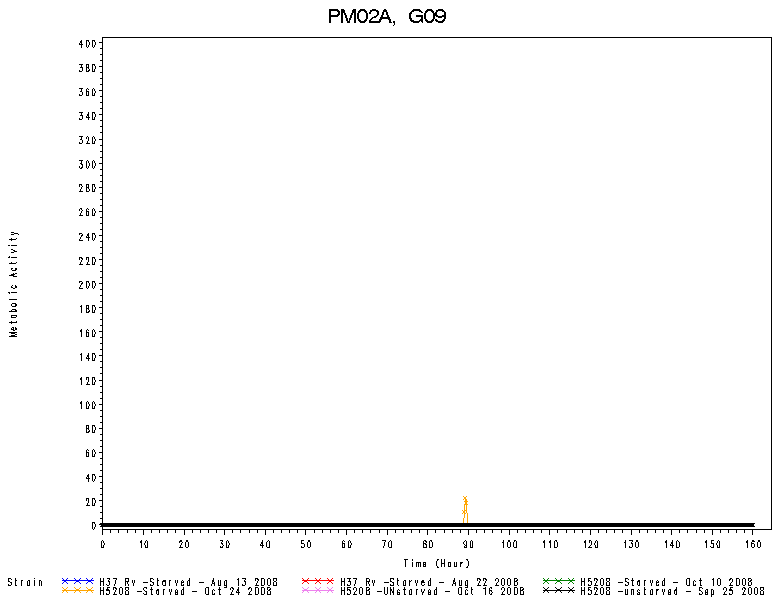

Supplement: Figure S1 — Kinetic curves for all PM plates with Mycobacterium tuberculosis H37Rv and Bj5208 strains. (ZIP) [file pone.0052673.s001.zip › suppl fig 1G H37Rv and Bj5208/Plate02A/pm02ag09.gif]

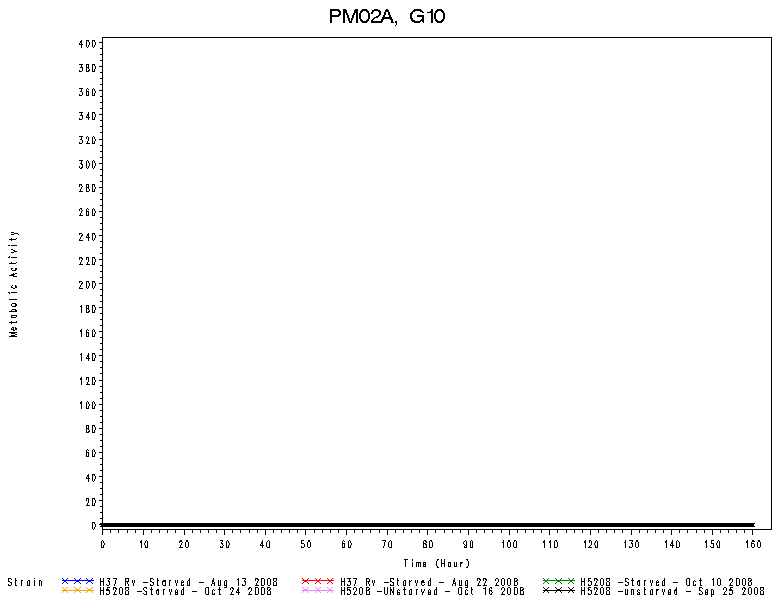

Supplement: Figure S1 — Kinetic curves for all PM plates with Mycobacterium tuberculosis H37Rv and Bj5208 strains. (ZIP) [file pone.0052673.s001.zip › suppl fig 1G H37Rv and Bj5208/Plate02A/pm02ag10.gif]

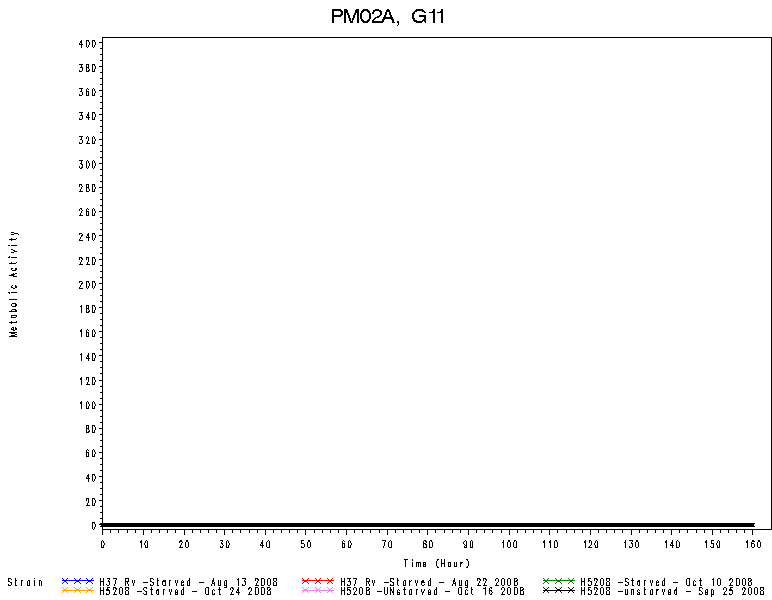

Supplement: Figure S1 — Kinetic curves for all PM plates with Mycobacterium tuberculosis H37Rv and Bj5208 strains. (ZIP) [file pone.0052673.s001.zip › suppl fig 1G H37Rv and Bj5208/Plate02A/pm02ag11.gif]

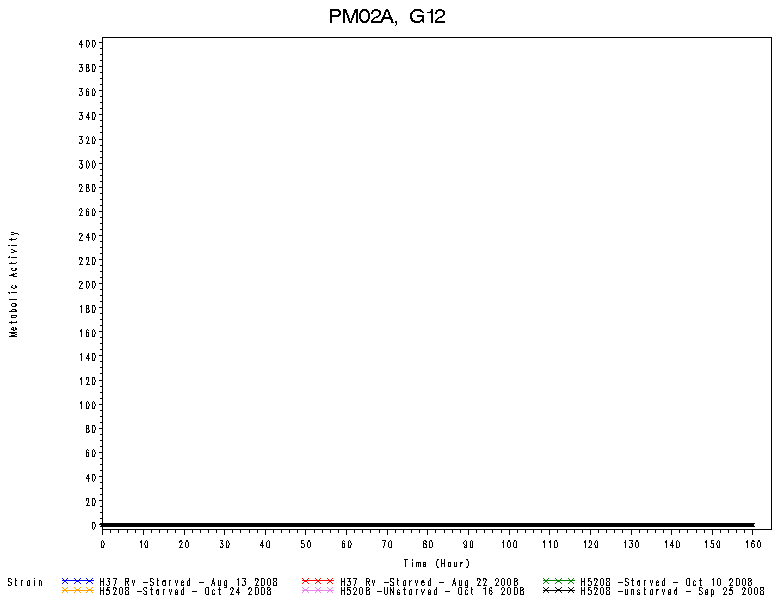

Supplement: Figure S1 — Kinetic curves for all PM plates with Mycobacterium tuberculosis H37Rv and Bj5208 strains. (ZIP) [file pone.0052673.s001.zip › suppl fig 1G H37Rv and Bj5208/Plate02A/pm02ag12.gif]

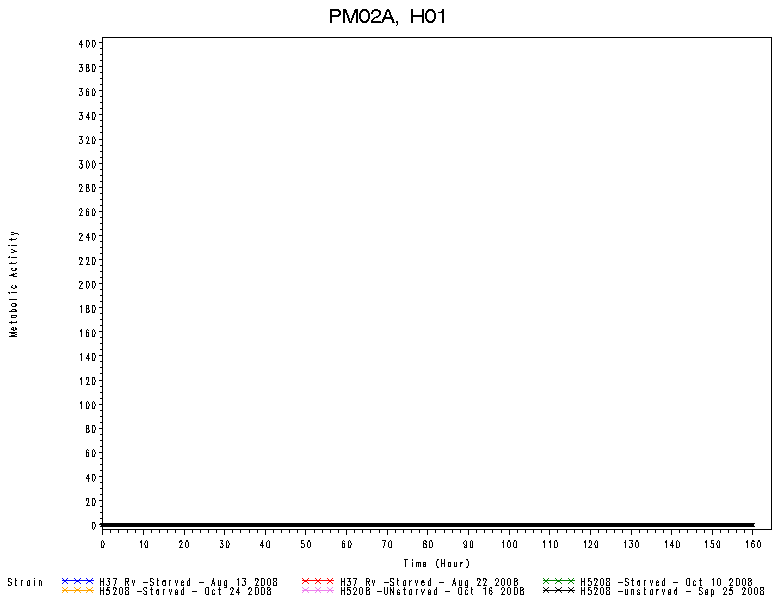

Supplement: Figure S1 — Kinetic curves for all PM plates with Mycobacterium tuberculosis H37Rv and Bj5208 strains. (ZIP) [file pone.0052673.s001.zip › suppl fig 1G H37Rv and Bj5208/Plate02A/pm02ah01.gif]

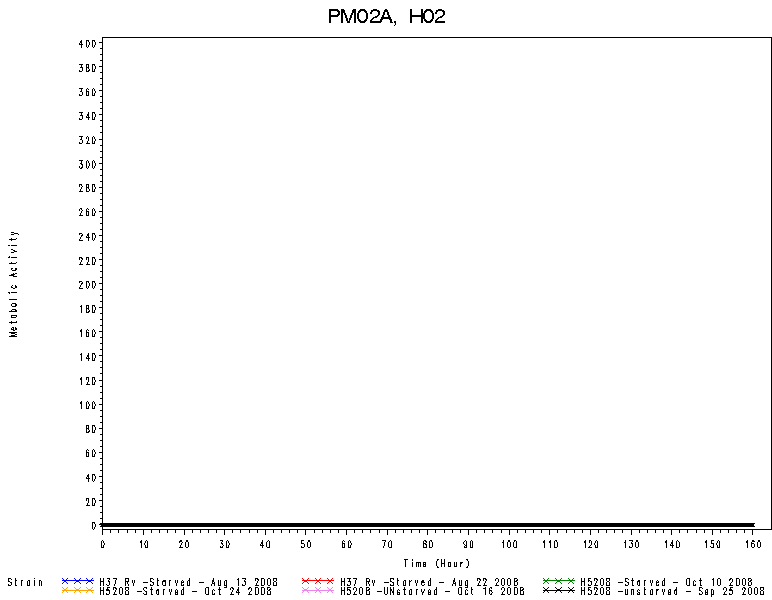

Supplement: Figure S1 — Kinetic curves for all PM plates with Mycobacterium tuberculosis H37Rv and Bj5208 strains. (ZIP) [file pone.0052673.s001.zip › suppl fig 1G H37Rv and Bj5208/Plate02A/pm02ah02.gif]

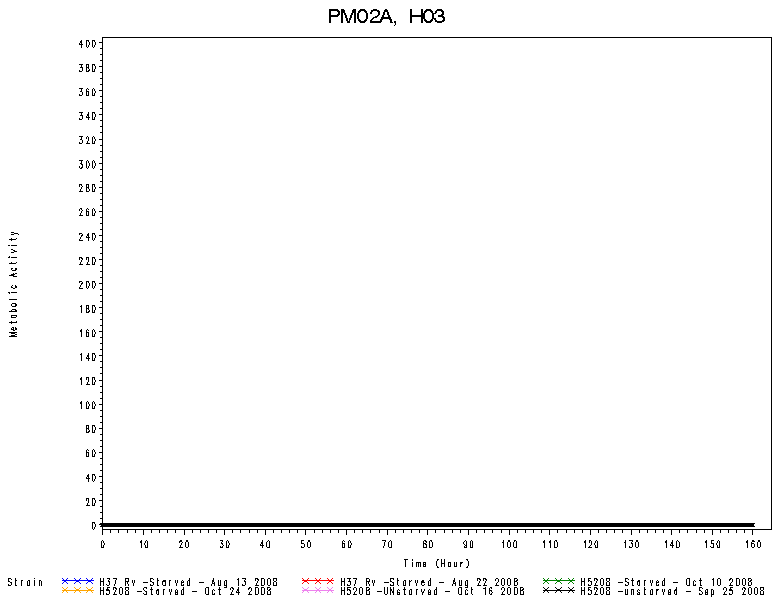

Supplement: Figure S1 — Kinetic curves for all PM plates with Mycobacterium tuberculosis H37Rv and Bj5208 strains. (ZIP) [file pone.0052673.s001.zip › suppl fig 1G H37Rv and Bj5208/Plate02A/pm02ah03.gif]

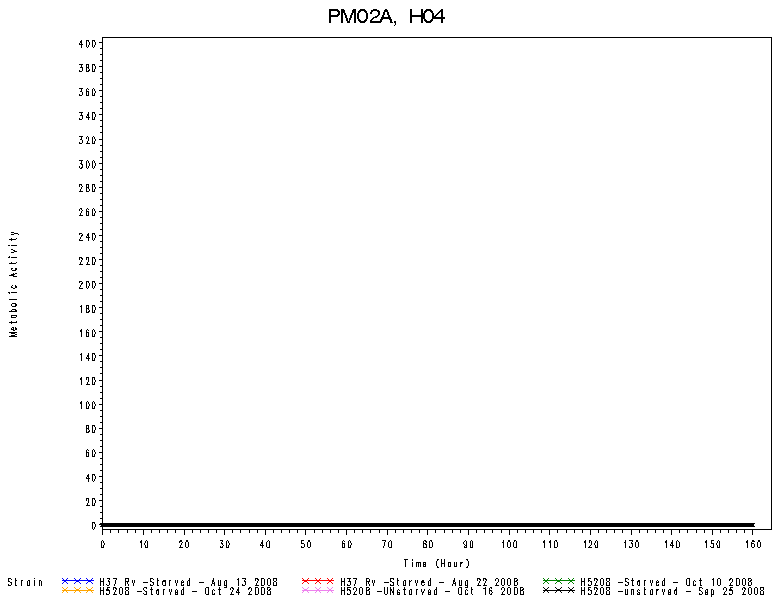

Supplement: Figure S1 — Kinetic curves for all PM plates with Mycobacterium tuberculosis H37Rv and Bj5208 strains. (ZIP) [file pone.0052673.s001.zip › suppl fig 1G H37Rv and Bj5208/Plate02A/pm02ah04.gif]

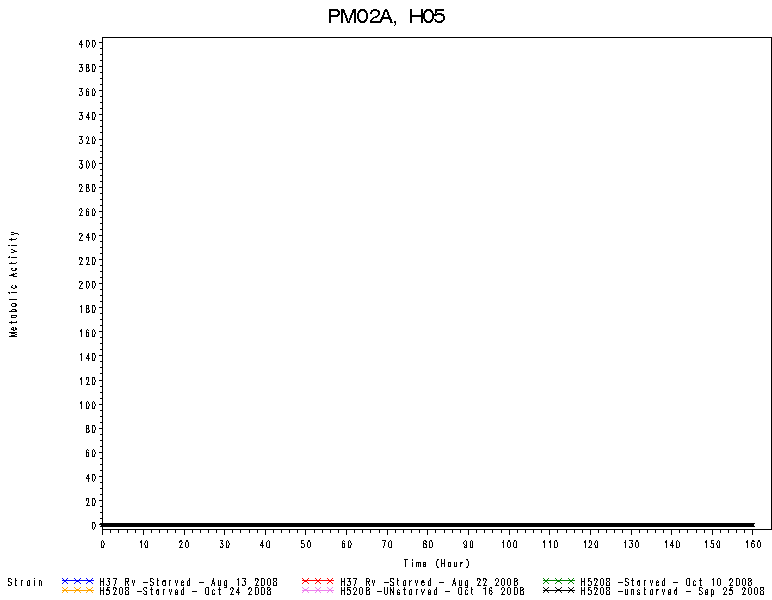

Supplement: Figure S1 — Kinetic curves for all PM plates with Mycobacterium tuberculosis H37Rv and Bj5208 strains. (ZIP) [file pone.0052673.s001.zip › suppl fig 1G H37Rv and Bj5208/Plate02A/pm02ah05.gif]

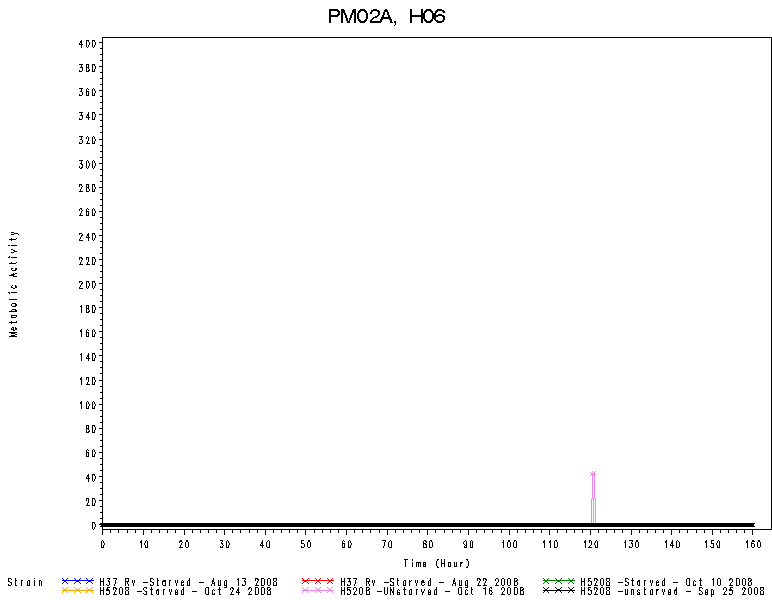

Supplement: Figure S1 — Kinetic curves for all PM plates with Mycobacterium tuberculosis H37Rv and Bj5208 strains. (ZIP) [file pone.0052673.s001.zip › suppl fig 1G H37Rv and Bj5208/Plate02A/pm02ah06.gif]

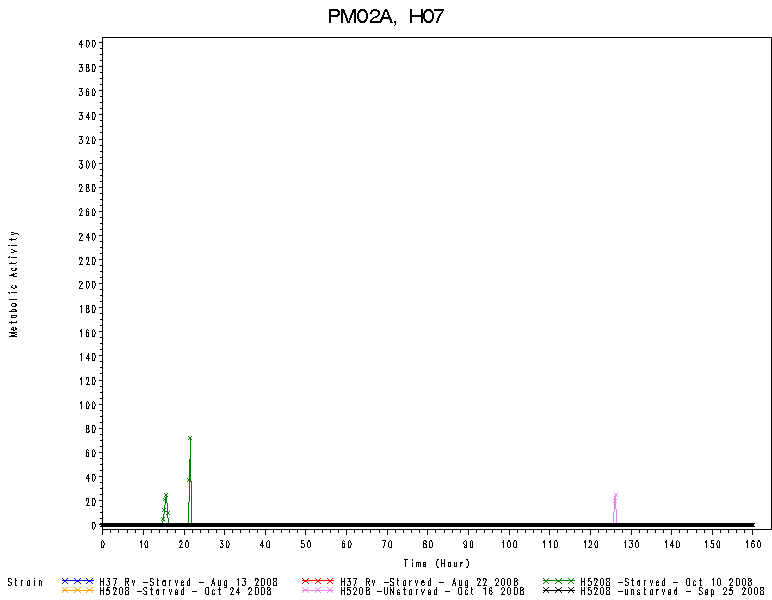

Supplement: Figure S1 — Kinetic curves for all PM plates with Mycobacterium tuberculosis H37Rv and Bj5208 strains. (ZIP) [file pone.0052673.s001.zip › suppl fig 1G H37Rv and Bj5208/Plate02A/pm02ah07.gif]

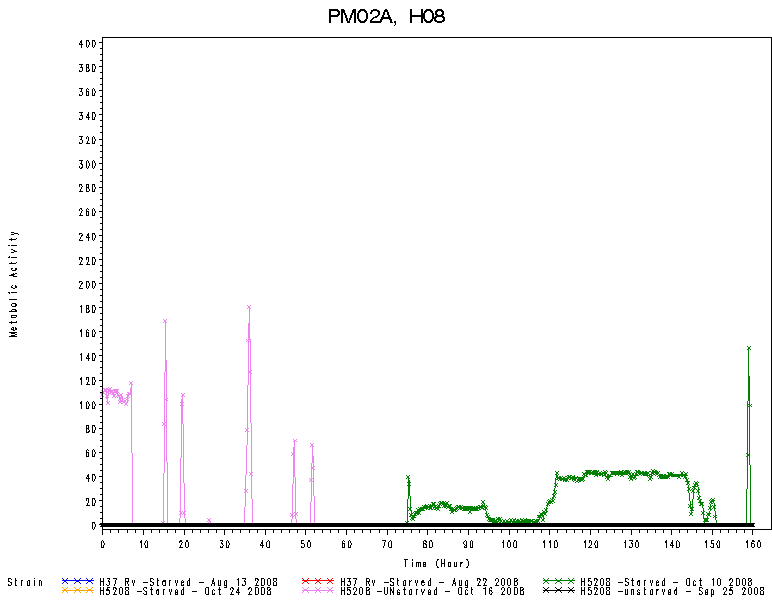

Supplement: Figure S1 — Kinetic curves for all PM plates with Mycobacterium tuberculosis H37Rv and Bj5208 strains. (ZIP) [file pone.0052673.s001.zip › suppl fig 1G H37Rv and Bj5208/Plate02A/pm02ah08.gif]

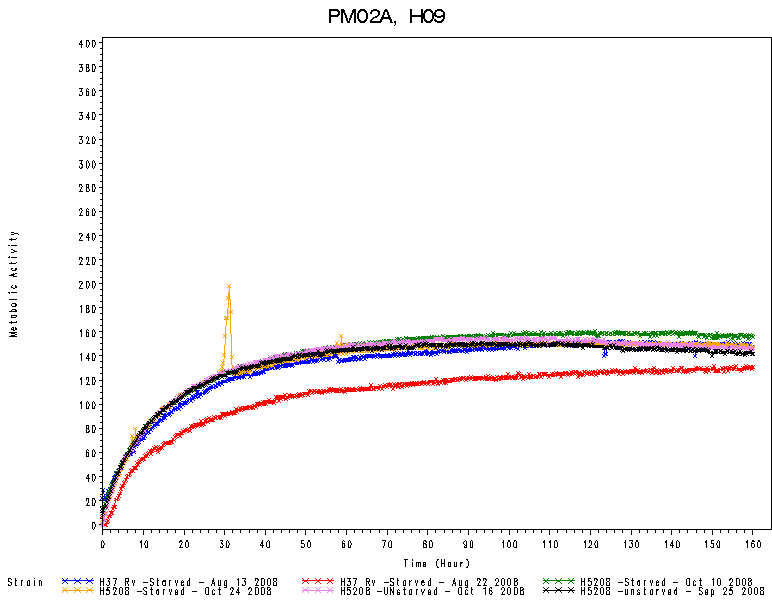

Supplement: Figure S1 — Kinetic curves for all PM plates with Mycobacterium tuberculosis H37Rv and Bj5208 strains. (ZIP) [file pone.0052673.s001.zip › suppl fig 1G H37Rv and Bj5208/Plate02A/pm02ah09.gif]

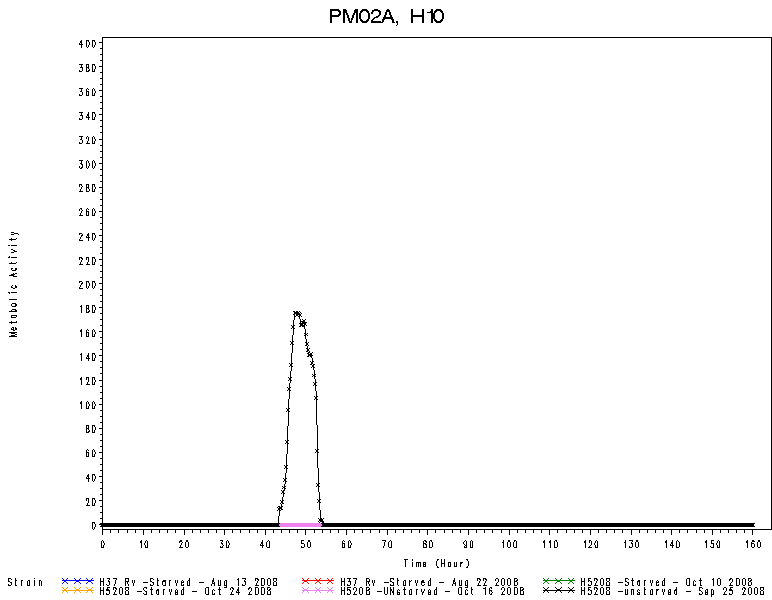

Supplement: Figure S1 — Kinetic curves for all PM plates with Mycobacterium tuberculosis H37Rv and Bj5208 strains. (ZIP) [file pone.0052673.s001.zip › suppl fig 1G H37Rv and Bj5208/Plate02A/pm02ah10.gif]

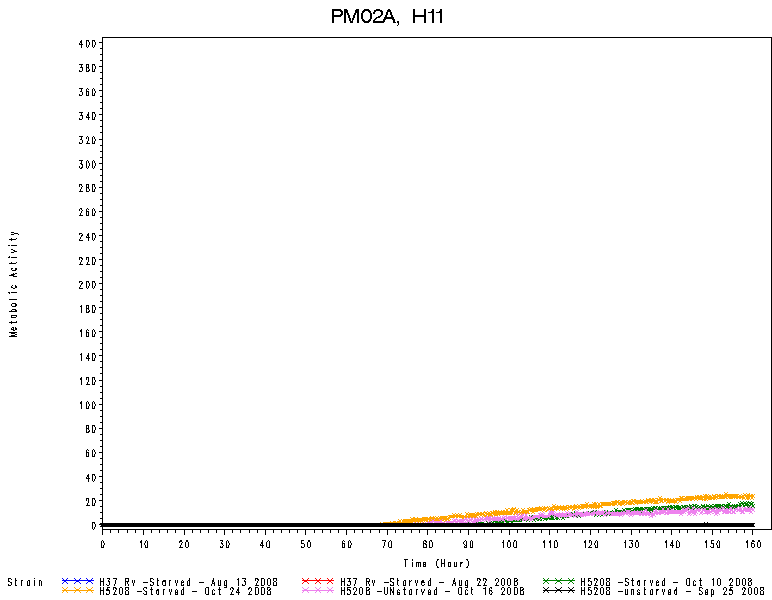

Supplement: Figure S1 — Kinetic curves for all PM plates with Mycobacterium tuberculosis H37Rv and Bj5208 strains. (ZIP) [file pone.0052673.s001.zip › suppl fig 1G H37Rv and Bj5208/Plate02A/pm02ah11.gif]

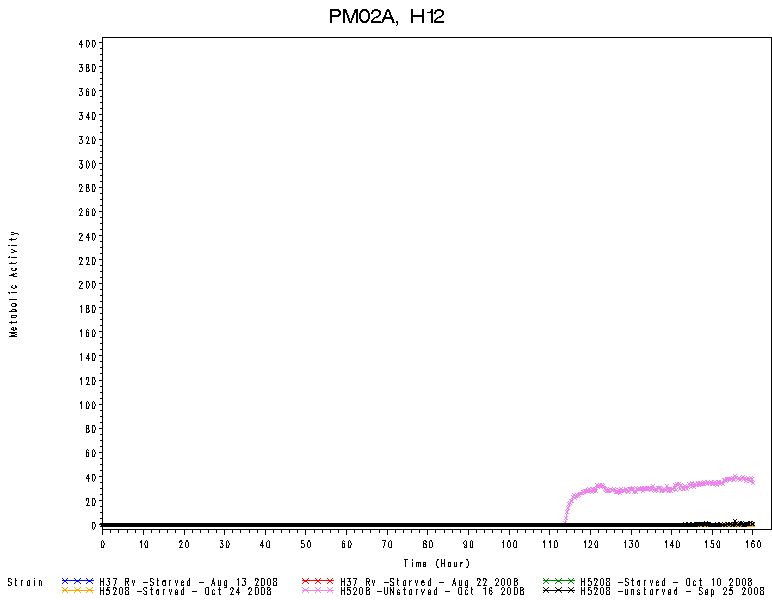

Supplement: Figure S1 — Kinetic curves for all PM plates with Mycobacterium tuberculosis H37Rv and Bj5208 strains. (ZIP) [file pone.0052673.s001.zip › suppl fig 1G H37Rv and Bj5208/Plate02A/pm02ah12.gif]

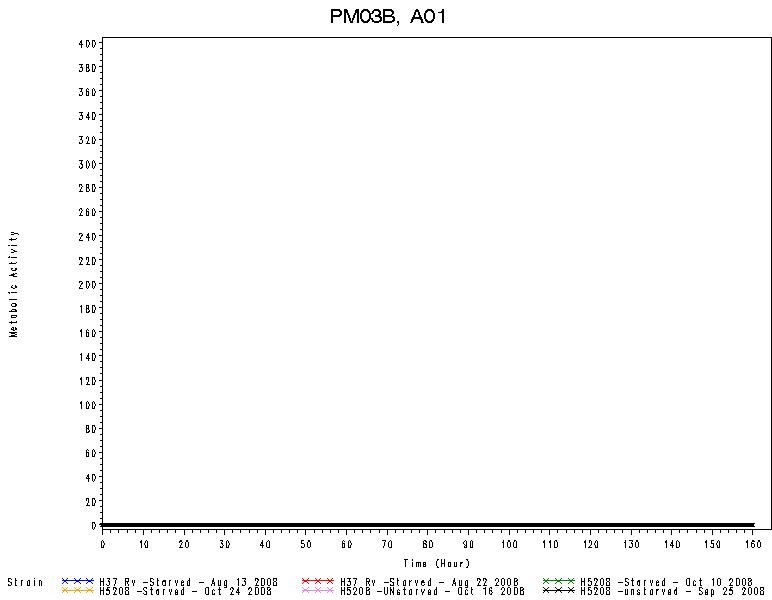

Supplement: Figure S1 — Kinetic curves for all PM plates with Mycobacterium tuberculosis H37Rv and Bj5208 strains. (ZIP) [file pone.0052673.s001.zip › suppl fig 1G H37Rv and Bj5208/Plate03B/pm03ba01.gif]

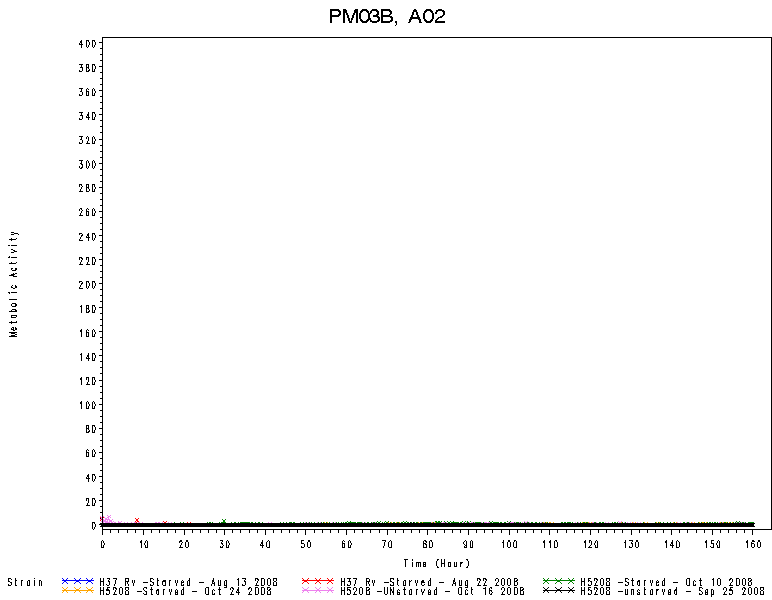

Supplement: Figure S1 — Kinetic curves for all PM plates with Mycobacterium tuberculosis H37Rv and Bj5208 strains. (ZIP) [file pone.0052673.s001.zip › suppl fig 1G H37Rv and Bj5208/Plate03B/pm03ba02.gif]

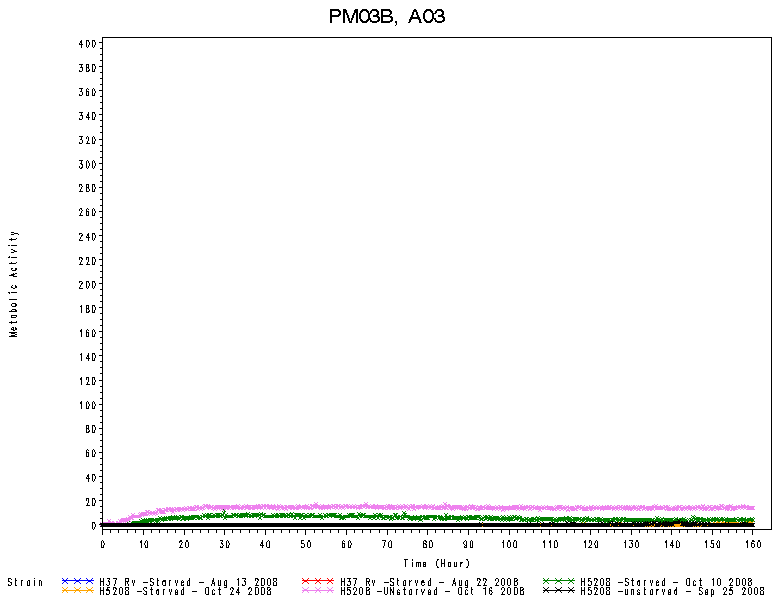

Supplement: Figure S1 — Kinetic curves for all PM plates with Mycobacterium tuberculosis H37Rv and Bj5208 strains. (ZIP) [file pone.0052673.s001.zip › suppl fig 1G H37Rv and Bj5208/Plate03B/pm03ba03.gif]

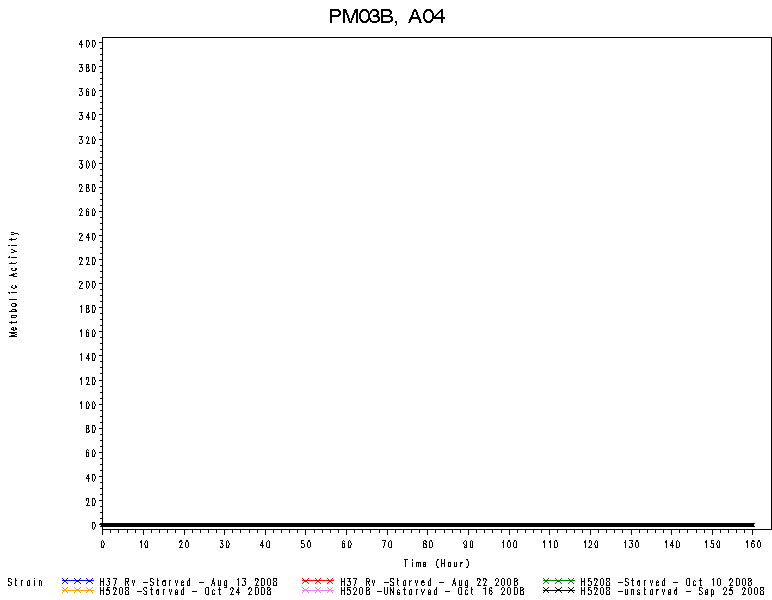

Supplement: Figure S1 — Kinetic curves for all PM plates with Mycobacterium tuberculosis H37Rv and Bj5208 strains. (ZIP) [file pone.0052673.s001.zip › suppl fig 1G H37Rv and Bj5208/Plate03B/pm03ba04.gif]

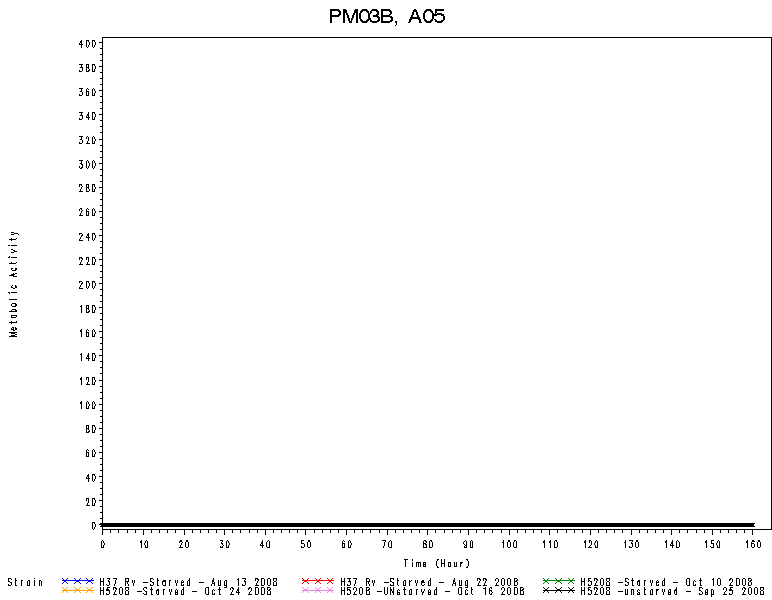

Supplement: Figure S1 — Kinetic curves for all PM plates with Mycobacterium tuberculosis H37Rv and Bj5208 strains. (ZIP) [file pone.0052673.s001.zip › suppl fig 1G H37Rv and Bj5208/Plate03B/pm03ba05.gif]

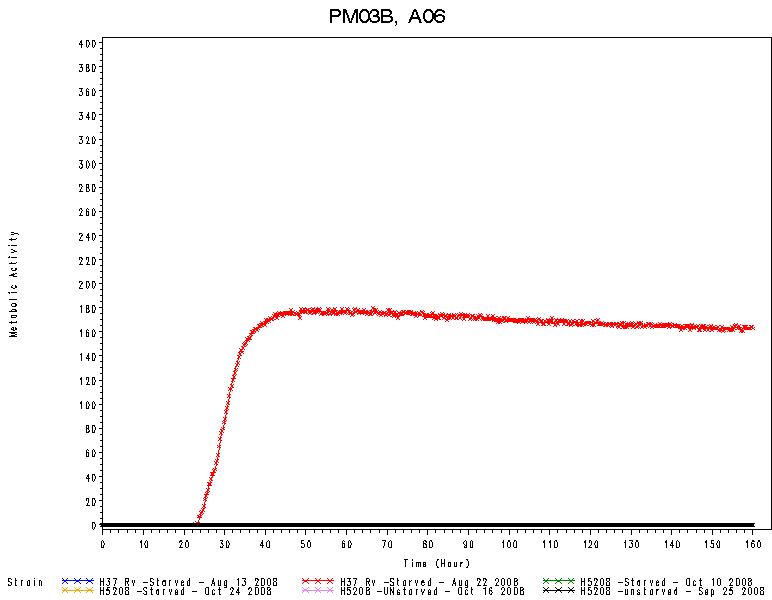

Supplement: Figure S1 — Kinetic curves for all PM plates with Mycobacterium tuberculosis H37Rv and Bj5208 strains. (ZIP) [file pone.0052673.s001.zip › suppl fig 1G H37Rv and Bj5208/Plate03B/pm03ba06.gif]

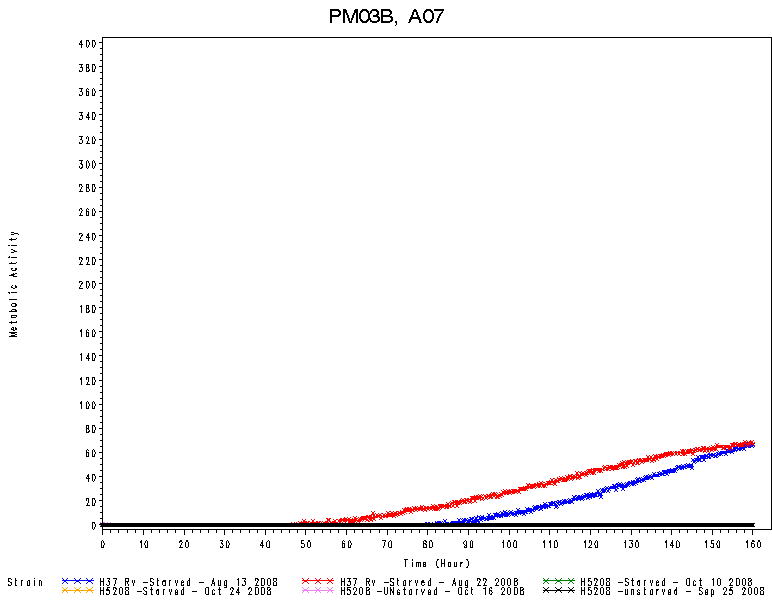

Supplement: Figure S1 — Kinetic curves for all PM plates with Mycobacterium tuberculosis H37Rv and Bj5208 strains. (ZIP) [file pone.0052673.s001.zip › suppl fig 1G H37Rv and Bj5208/Plate03B/pm03ba07.gif]

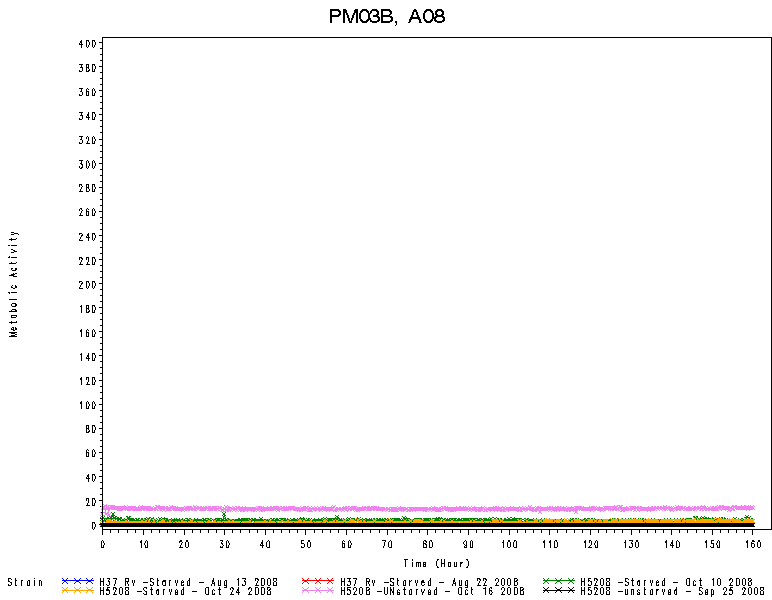

Supplement: Figure S1 — Kinetic curves for all PM plates with Mycobacterium tuberculosis H37Rv and Bj5208 strains. (ZIP) [file pone.0052673.s001.zip › suppl fig 1G H37Rv and Bj5208/Plate03B/pm03ba08.gif]
