# Supplementary figures and images for: High Throughput Phenotypic Analysis of Mycobacterium tuberculosis and Mycobacterium bovis Strains' Metabolism Using Biolog Phenotype Microarrays (part 1 of 11)
Source: PLoS One. 2013 Jan 10;8(1):e52673. doi: 10.1371/journal.pone.0052673 (PMC3542357; doi:10.1371/journal.pone.0052673)

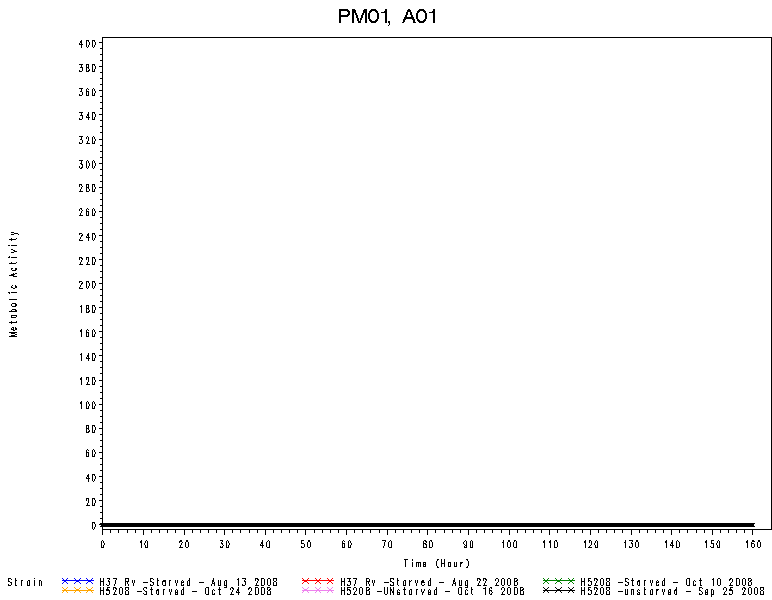

Supplement: Figure S1 — Kinetic curves for all PM plates with Mycobacterium tuberculosis H37Rv and Bj5208 strains. (ZIP) [file pone.0052673.s001.zip › suppl fig 1G H37Rv and Bj5208/Plate01/pm01a01.gif]

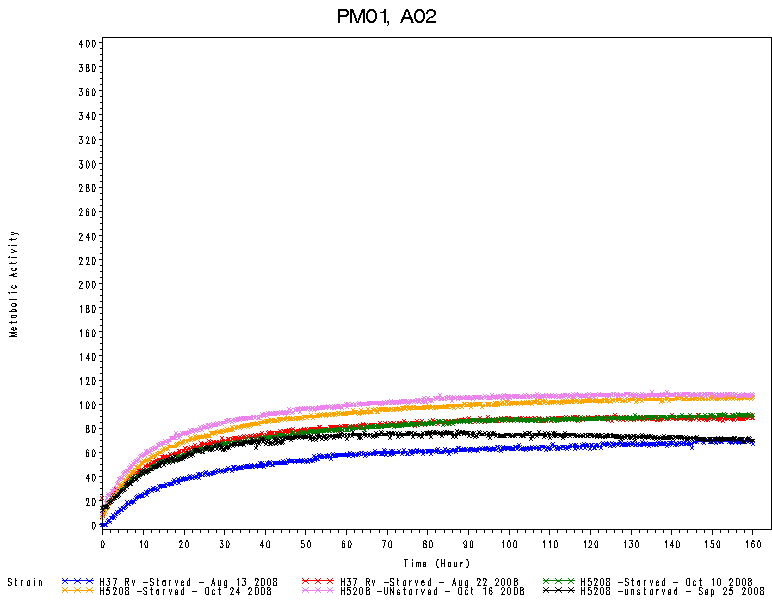

Supplement: Figure S1 — Kinetic curves for all PM plates with Mycobacterium tuberculosis H37Rv and Bj5208 strains. (ZIP) [file pone.0052673.s001.zip › suppl fig 1G H37Rv and Bj5208/Plate01/pm01a02.gif]

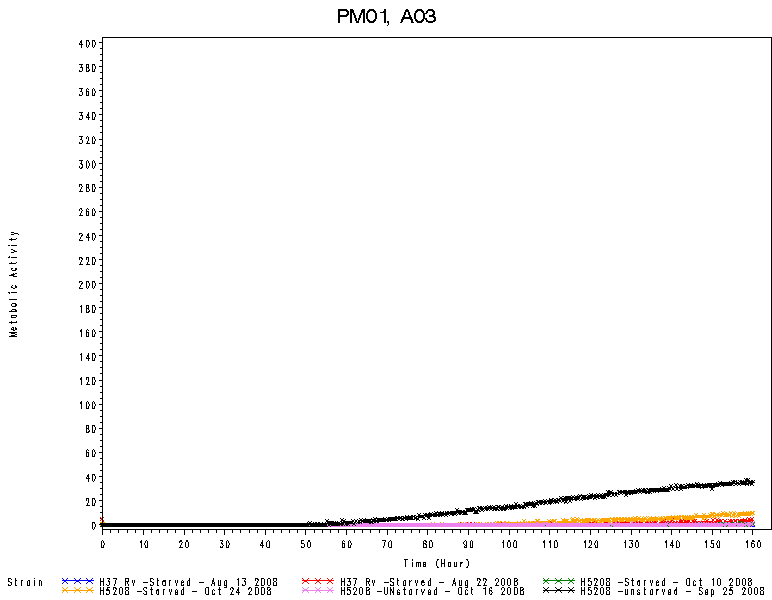

Supplement: Figure S1 — Kinetic curves for all PM plates with Mycobacterium tuberculosis H37Rv and Bj5208 strains. (ZIP) [file pone.0052673.s001.zip › suppl fig 1G H37Rv and Bj5208/Plate01/pm01a03.gif]

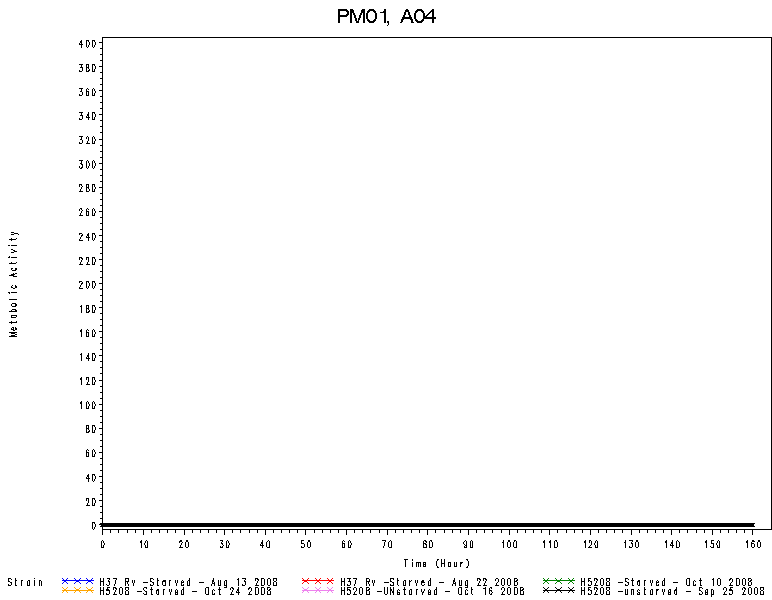

Supplement: Figure S1 — Kinetic curves for all PM plates with Mycobacterium tuberculosis H37Rv and Bj5208 strains. (ZIP) [file pone.0052673.s001.zip › suppl fig 1G H37Rv and Bj5208/Plate01/pm01a04.gif]

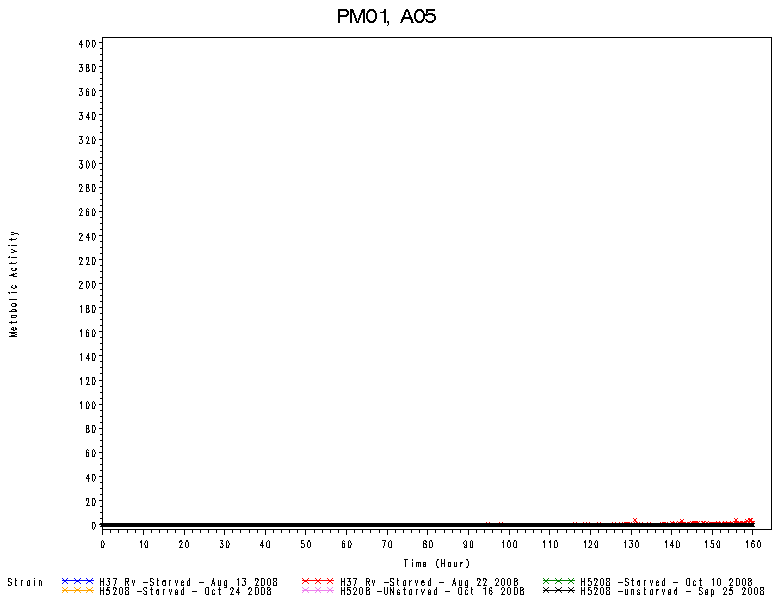

Supplement: Figure S1 — Kinetic curves for all PM plates with Mycobacterium tuberculosis H37Rv and Bj5208 strains. (ZIP) [file pone.0052673.s001.zip › suppl fig 1G H37Rv and Bj5208/Plate01/pm01a05.gif]

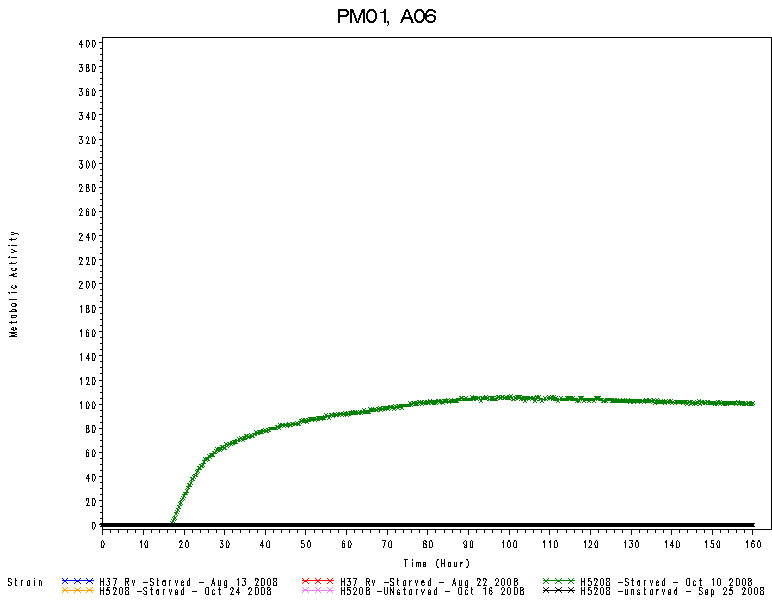

Supplement: Figure S1 — Kinetic curves for all PM plates with Mycobacterium tuberculosis H37Rv and Bj5208 strains. (ZIP) [file pone.0052673.s001.zip › suppl fig 1G H37Rv and Bj5208/Plate01/pm01a06.gif]

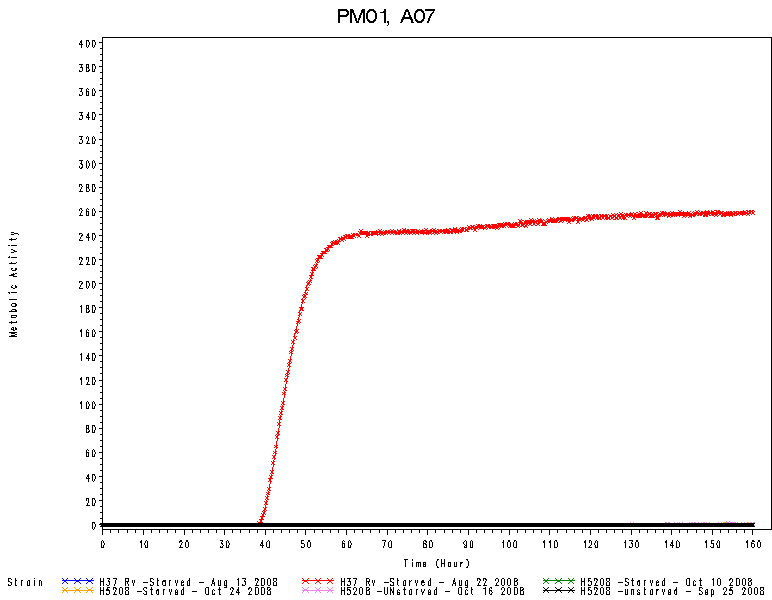

Supplement: Figure S1 — Kinetic curves for all PM plates with Mycobacterium tuberculosis H37Rv and Bj5208 strains. (ZIP) [file pone.0052673.s001.zip › suppl fig 1G H37Rv and Bj5208/Plate01/pm01a07.gif]

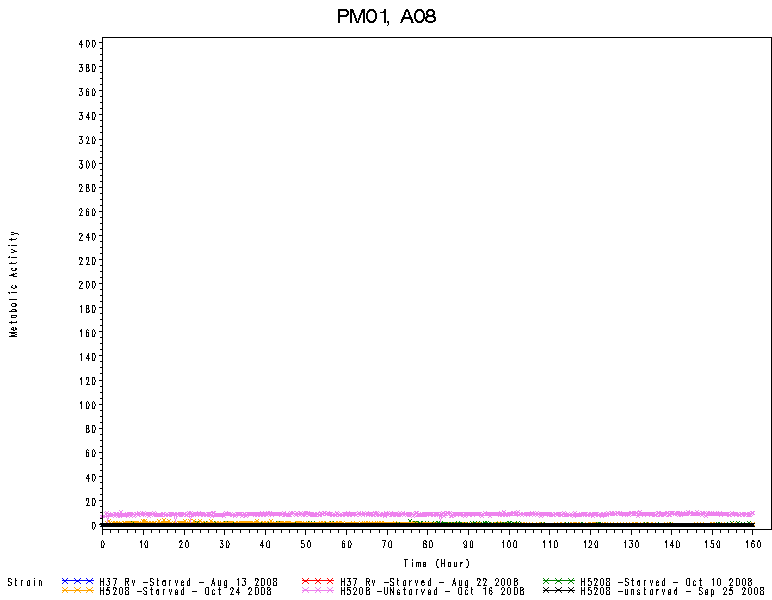

Supplement: Figure S1 — Kinetic curves for all PM plates with Mycobacterium tuberculosis H37Rv and Bj5208 strains. (ZIP) [file pone.0052673.s001.zip › suppl fig 1G H37Rv and Bj5208/Plate01/pm01a08.gif]

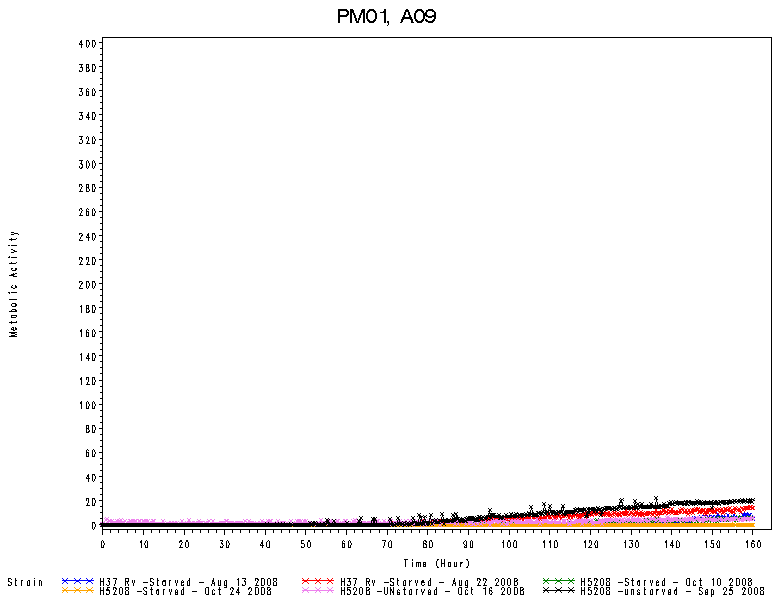

Supplement: Figure S1 — Kinetic curves for all PM plates with Mycobacterium tuberculosis H37Rv and Bj5208 strains. (ZIP) [file pone.0052673.s001.zip › suppl fig 1G H37Rv and Bj5208/Plate01/pm01a09.gif]

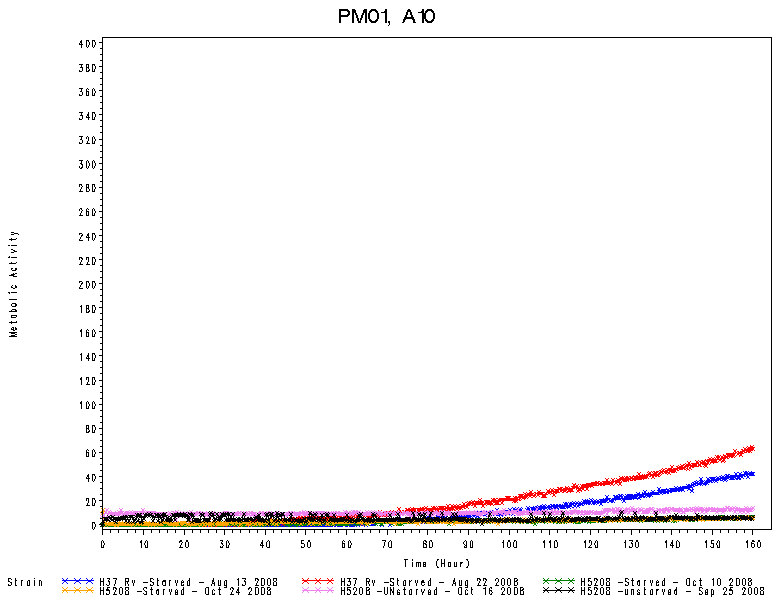

Supplement: Figure S1 — Kinetic curves for all PM plates with Mycobacterium tuberculosis H37Rv and Bj5208 strains. (ZIP) [file pone.0052673.s001.zip › suppl fig 1G H37Rv and Bj5208/Plate01/pm01a10.gif]

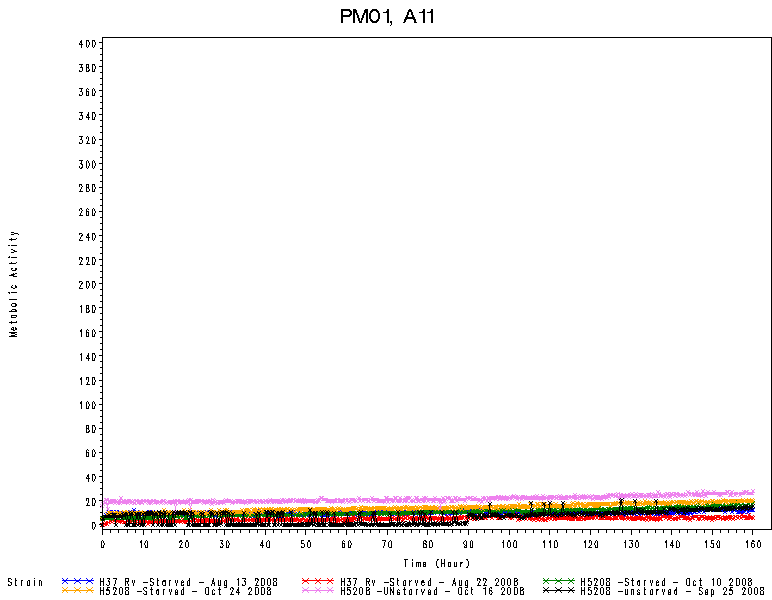

Supplement: Figure S1 — Kinetic curves for all PM plates with Mycobacterium tuberculosis H37Rv and Bj5208 strains. (ZIP) [file pone.0052673.s001.zip › suppl fig 1G H37Rv and Bj5208/Plate01/pm01a11.gif]

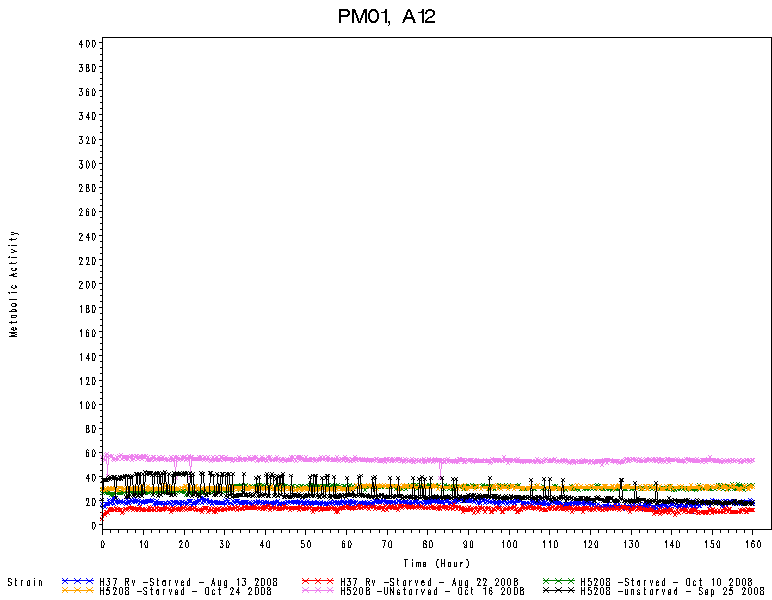

Supplement: Figure S1 — Kinetic curves for all PM plates with Mycobacterium tuberculosis H37Rv and Bj5208 strains. (ZIP) [file pone.0052673.s001.zip › suppl fig 1G H37Rv and Bj5208/Plate01/pm01a12.gif]

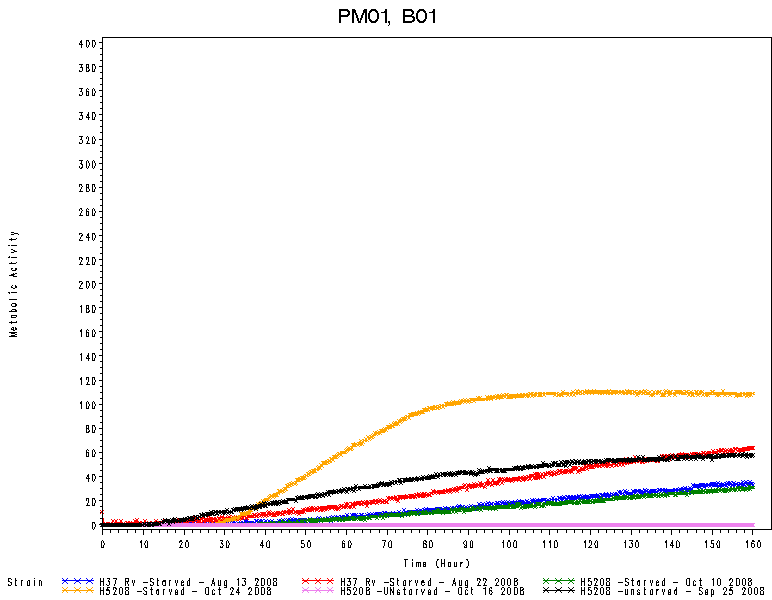

Supplement: Figure S1 — Kinetic curves for all PM plates with Mycobacterium tuberculosis H37Rv and Bj5208 strains. (ZIP) [file pone.0052673.s001.zip › suppl fig 1G H37Rv and Bj5208/Plate01/pm01b01.gif]

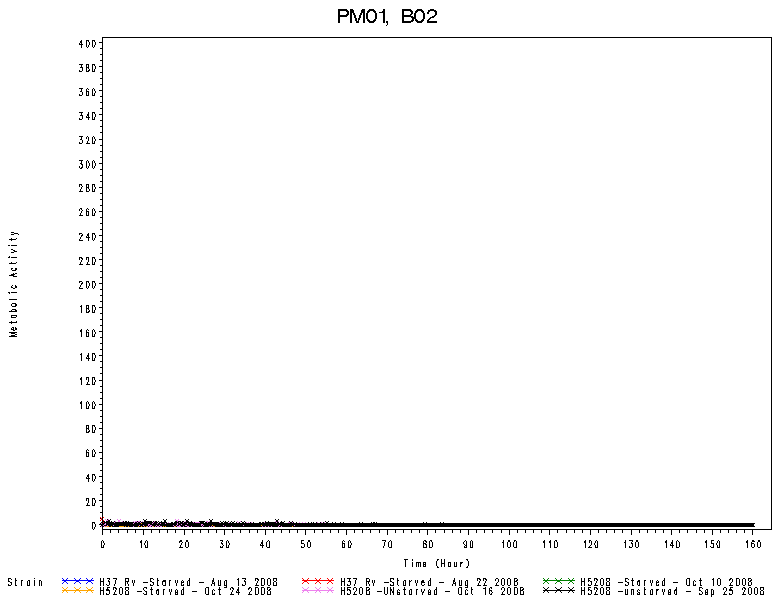

Supplement: Figure S1 — Kinetic curves for all PM plates with Mycobacterium tuberculosis H37Rv and Bj5208 strains. (ZIP) [file pone.0052673.s001.zip › suppl fig 1G H37Rv and Bj5208/Plate01/pm01b02.gif]

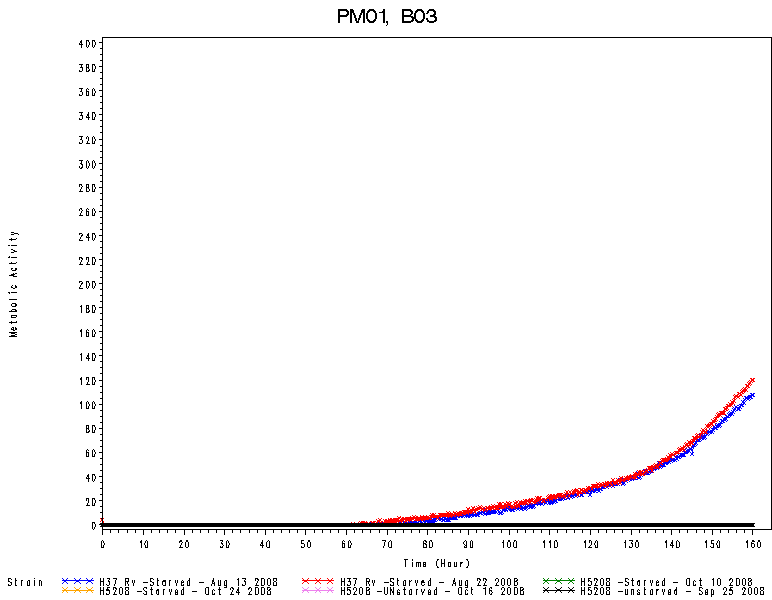

Supplement: Figure S1 — Kinetic curves for all PM plates with Mycobacterium tuberculosis H37Rv and Bj5208 strains. (ZIP) [file pone.0052673.s001.zip › suppl fig 1G H37Rv and Bj5208/Plate01/pm01b03.gif]

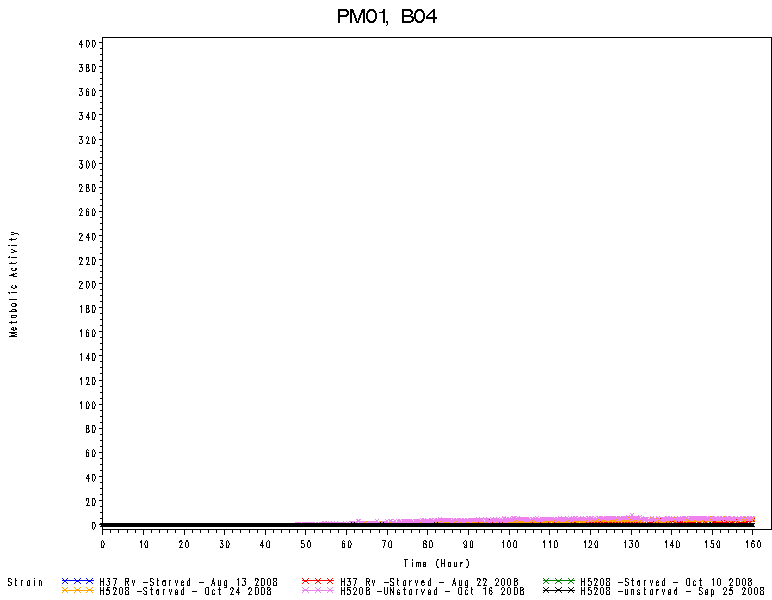

Supplement: Figure S1 — Kinetic curves for all PM plates with Mycobacterium tuberculosis H37Rv and Bj5208 strains. (ZIP) [file pone.0052673.s001.zip › suppl fig 1G H37Rv and Bj5208/Plate01/pm01b04.gif]

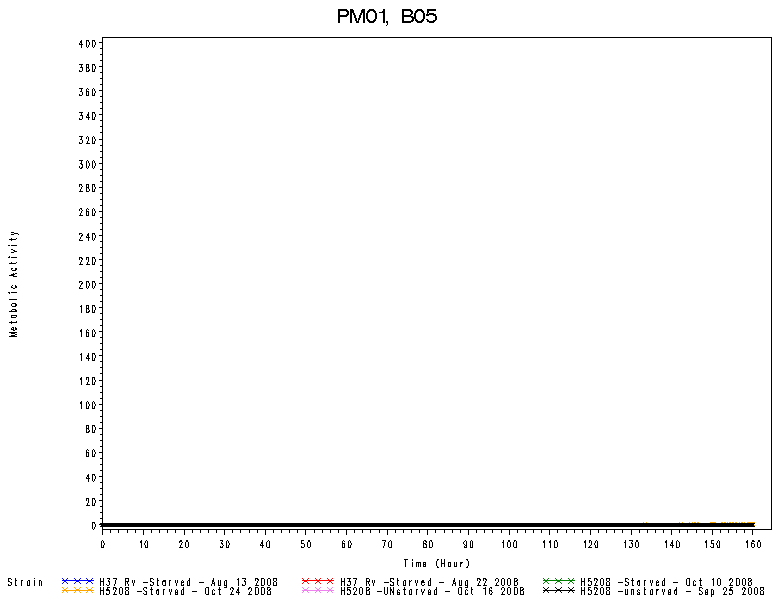

Supplement: Figure S1 — Kinetic curves for all PM plates with Mycobacterium tuberculosis H37Rv and Bj5208 strains. (ZIP) [file pone.0052673.s001.zip › suppl fig 1G H37Rv and Bj5208/Plate01/pm01b05.gif]

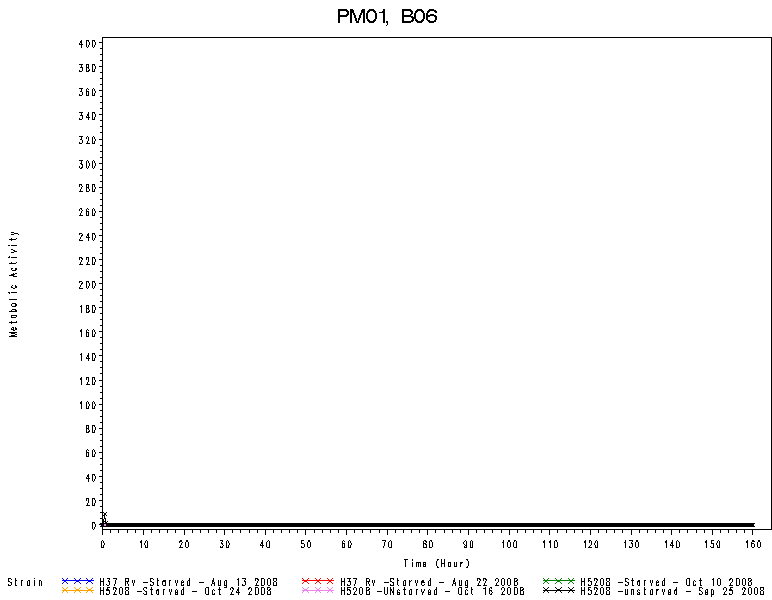

Supplement: Figure S1 — Kinetic curves for all PM plates with Mycobacterium tuberculosis H37Rv and Bj5208 strains. (ZIP) [file pone.0052673.s001.zip › suppl fig 1G H37Rv and Bj5208/Plate01/pm01b06.gif]

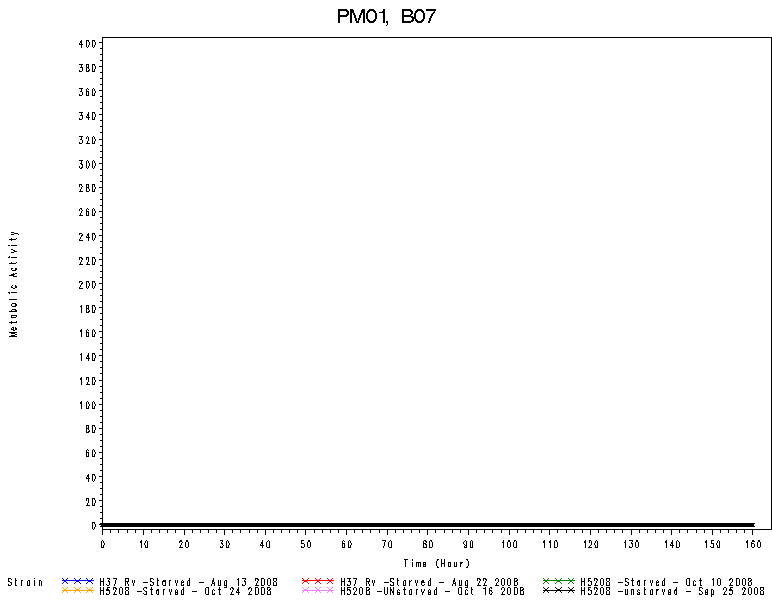

Supplement: Figure S1 — Kinetic curves for all PM plates with Mycobacterium tuberculosis H37Rv and Bj5208 strains. (ZIP) [file pone.0052673.s001.zip › suppl fig 1G H37Rv and Bj5208/Plate01/pm01b07.gif]

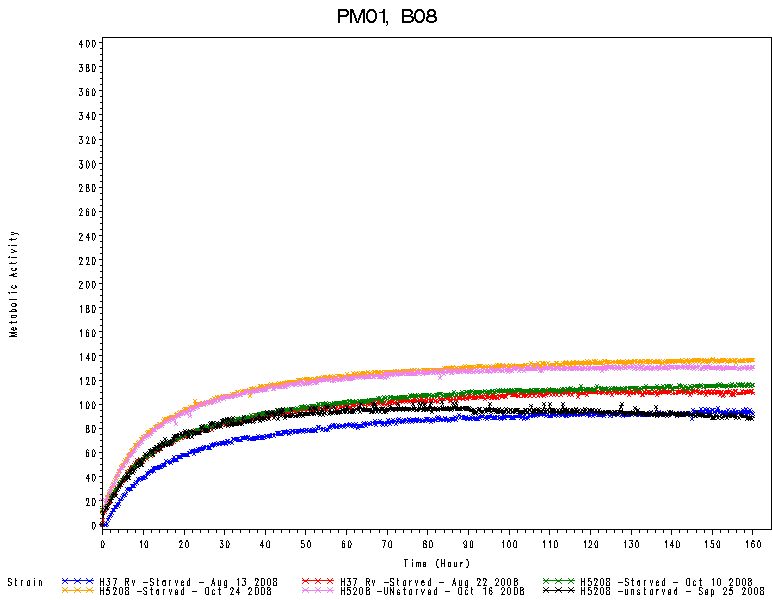

Supplement: Figure S1 — Kinetic curves for all PM plates with Mycobacterium tuberculosis H37Rv and Bj5208 strains. (ZIP) [file pone.0052673.s001.zip › suppl fig 1G H37Rv and Bj5208/Plate01/pm01b08.gif]

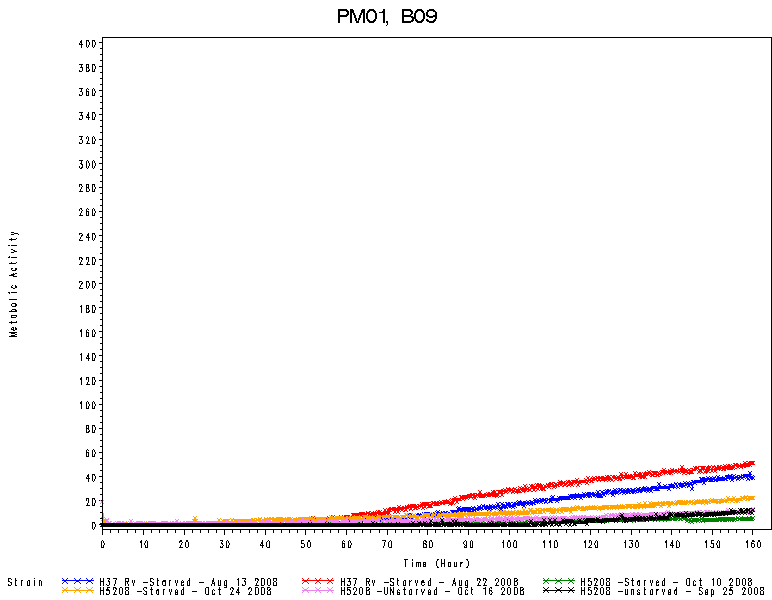

Supplement: Figure S1 — Kinetic curves for all PM plates with Mycobacterium tuberculosis H37Rv and Bj5208 strains. (ZIP) [file pone.0052673.s001.zip › suppl fig 1G H37Rv and Bj5208/Plate01/pm01b09.gif]

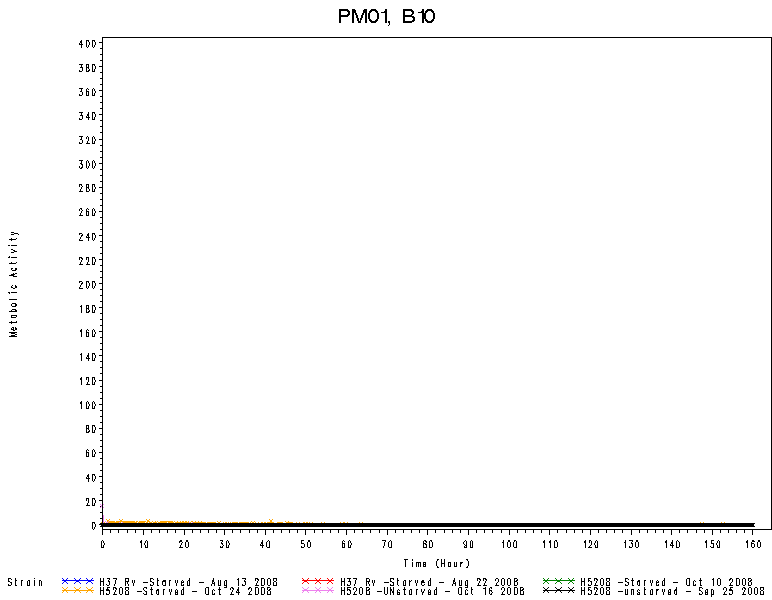

Supplement: Figure S1 — Kinetic curves for all PM plates with Mycobacterium tuberculosis H37Rv and Bj5208 strains. (ZIP) [file pone.0052673.s001.zip › suppl fig 1G H37Rv and Bj5208/Plate01/pm01b10.gif]

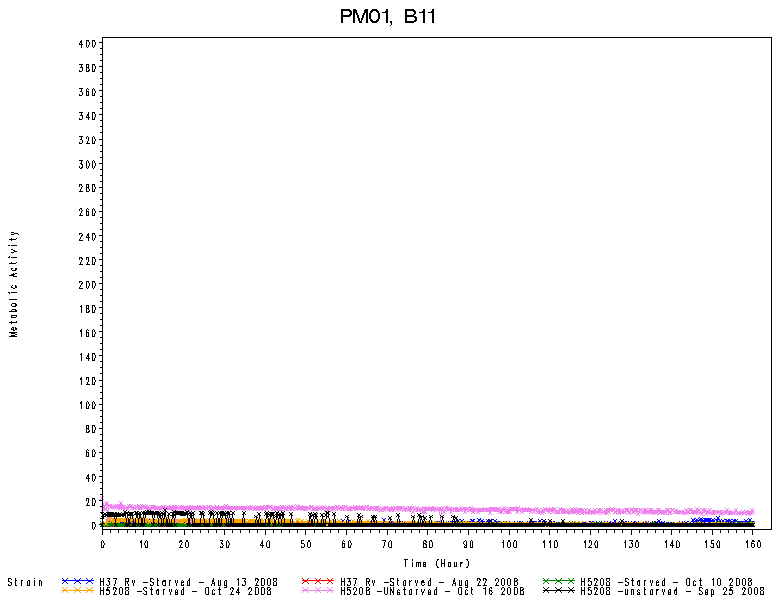

Supplement: Figure S1 — Kinetic curves for all PM plates with Mycobacterium tuberculosis H37Rv and Bj5208 strains. (ZIP) [file pone.0052673.s001.zip › suppl fig 1G H37Rv and Bj5208/Plate01/pm01b11.gif]

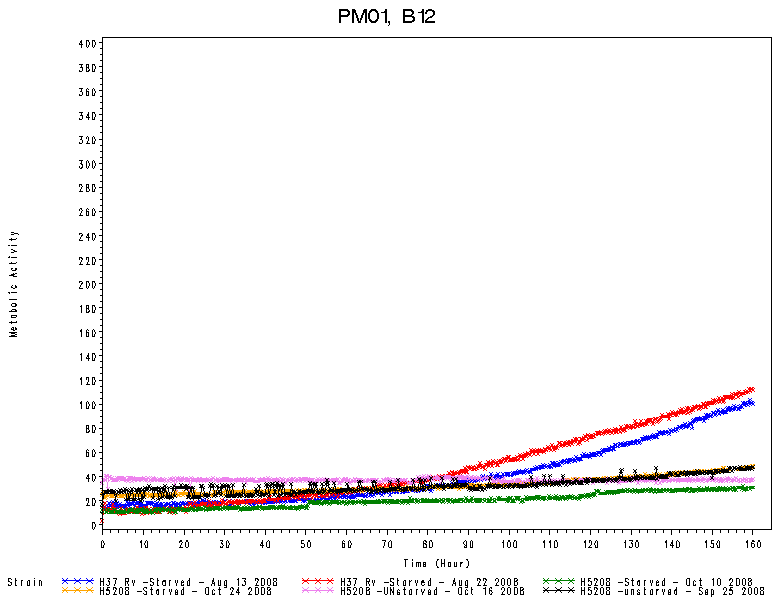

Supplement: Figure S1 — Kinetic curves for all PM plates with Mycobacterium tuberculosis H37Rv and Bj5208 strains. (ZIP) [file pone.0052673.s001.zip › suppl fig 1G H37Rv and Bj5208/Plate01/pm01b12.gif]

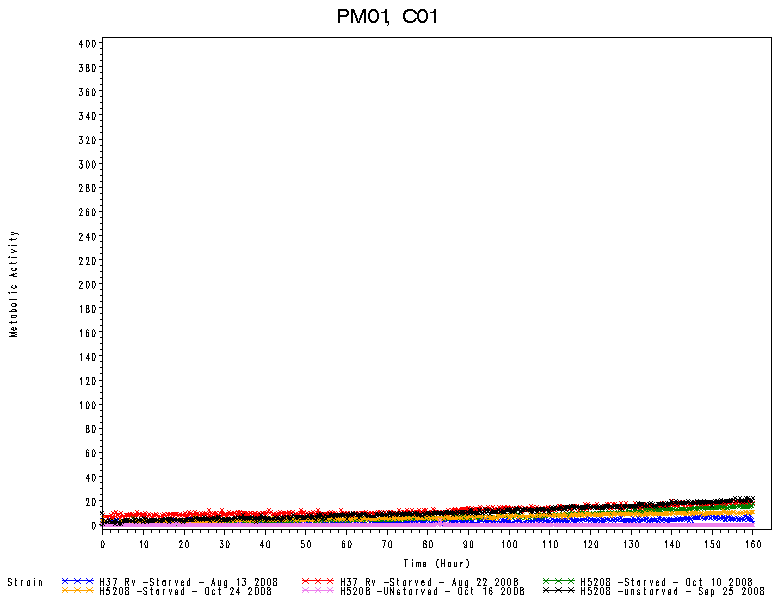

Supplement: Figure S1 — Kinetic curves for all PM plates with Mycobacterium tuberculosis H37Rv and Bj5208 strains. (ZIP) [file pone.0052673.s001.zip › suppl fig 1G H37Rv and Bj5208/Plate01/pm01c01.gif]

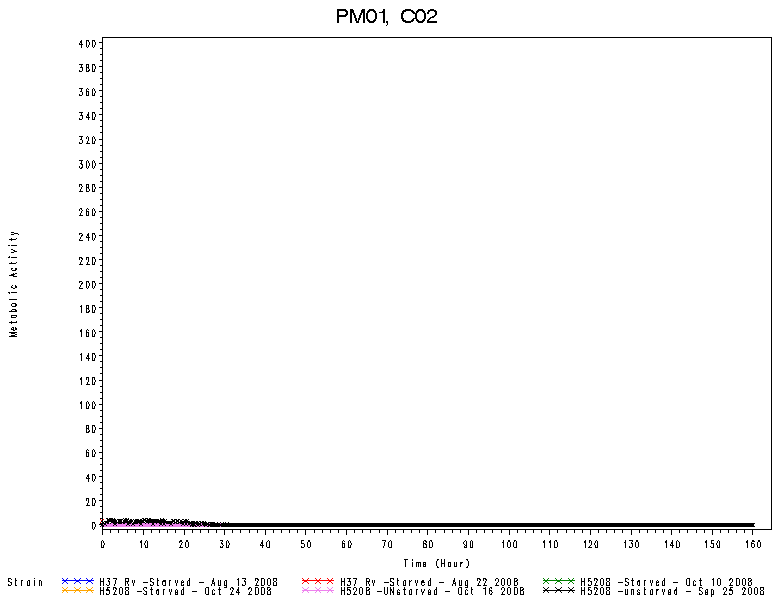

Supplement: Figure S1 — Kinetic curves for all PM plates with Mycobacterium tuberculosis H37Rv and Bj5208 strains. (ZIP) [file pone.0052673.s001.zip › suppl fig 1G H37Rv and Bj5208/Plate01/pm01c02.gif]

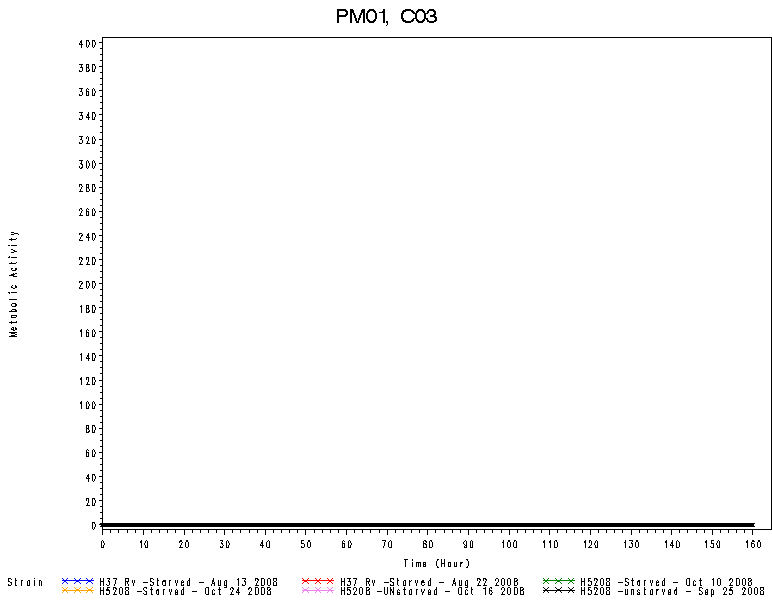

Supplement: Figure S1 — Kinetic curves for all PM plates with Mycobacterium tuberculosis H37Rv and Bj5208 strains. (ZIP) [file pone.0052673.s001.zip › suppl fig 1G H37Rv and Bj5208/Plate01/pm01c03.gif]

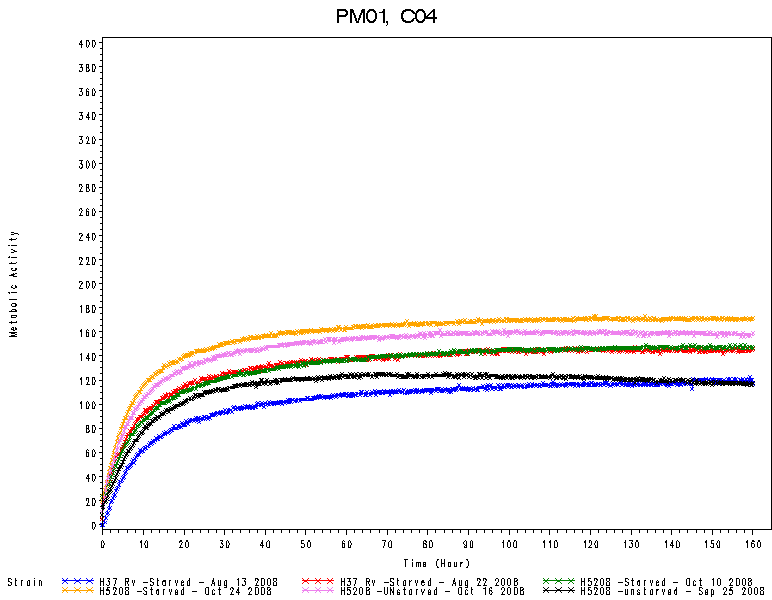

Supplement: Figure S1 — Kinetic curves for all PM plates with Mycobacterium tuberculosis H37Rv and Bj5208 strains. (ZIP) [file pone.0052673.s001.zip › suppl fig 1G H37Rv and Bj5208/Plate01/pm01c04.gif]

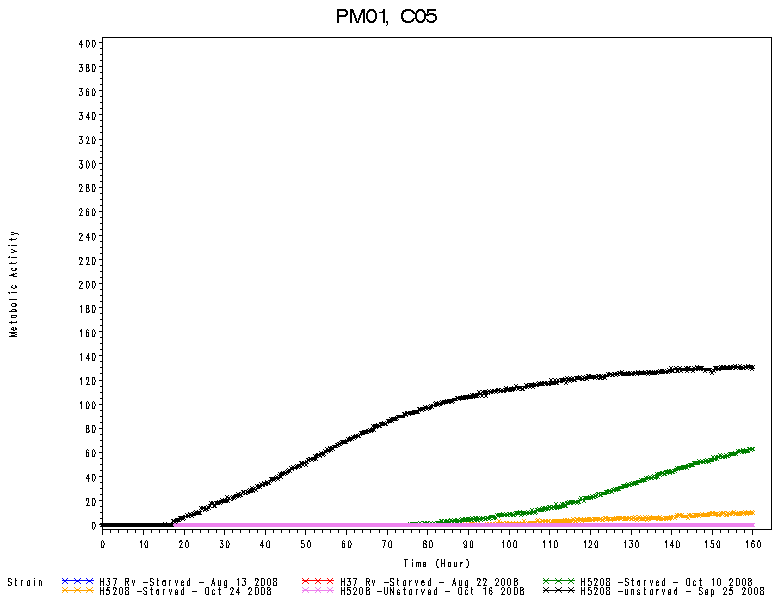

Supplement: Figure S1 — Kinetic curves for all PM plates with Mycobacterium tuberculosis H37Rv and Bj5208 strains. (ZIP) [file pone.0052673.s001.zip › suppl fig 1G H37Rv and Bj5208/Plate01/pm01c05.gif]

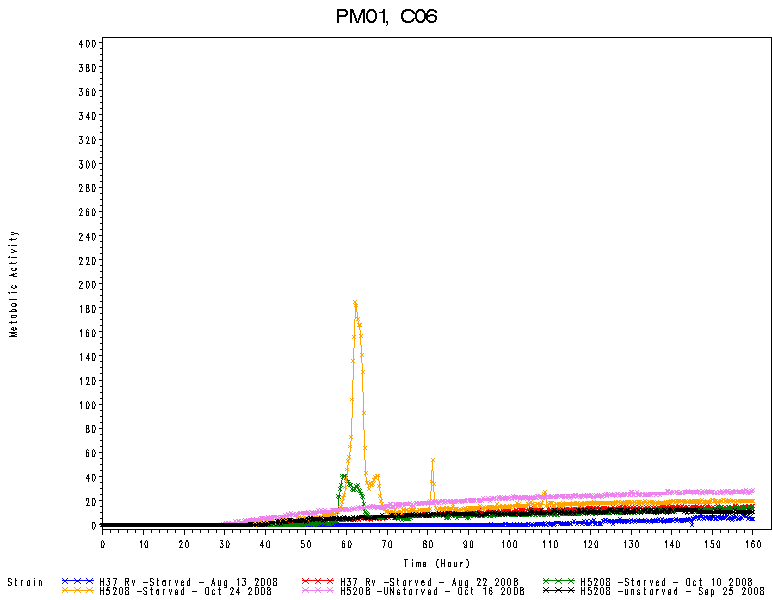

Supplement: Figure S1 — Kinetic curves for all PM plates with Mycobacterium tuberculosis H37Rv and Bj5208 strains. (ZIP) [file pone.0052673.s001.zip › suppl fig 1G H37Rv and Bj5208/Plate01/pm01c06.gif]

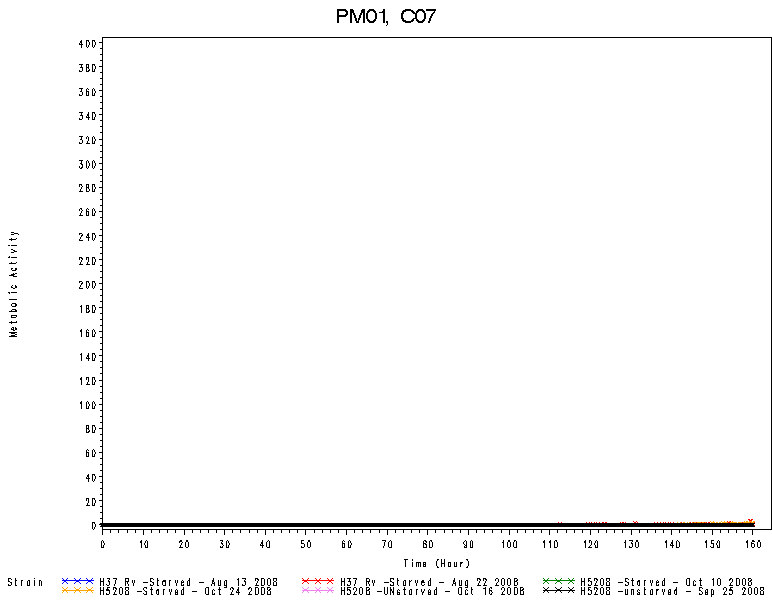

Supplement: Figure S1 — Kinetic curves for all PM plates with Mycobacterium tuberculosis H37Rv and Bj5208 strains. (ZIP) [file pone.0052673.s001.zip › suppl fig 1G H37Rv and Bj5208/Plate01/pm01c07.gif]

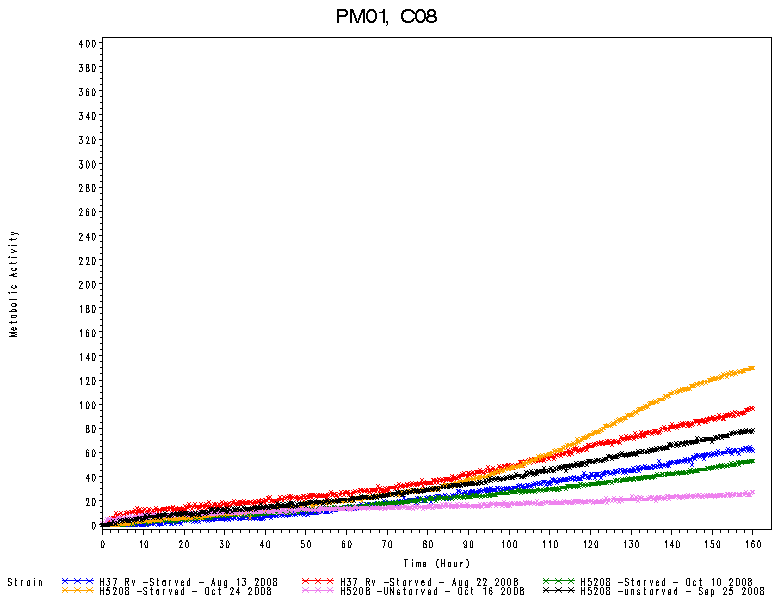

Supplement: Figure S1 — Kinetic curves for all PM plates with Mycobacterium tuberculosis H37Rv and Bj5208 strains. (ZIP) [file pone.0052673.s001.zip › suppl fig 1G H37Rv and Bj5208/Plate01/pm01c08.gif]

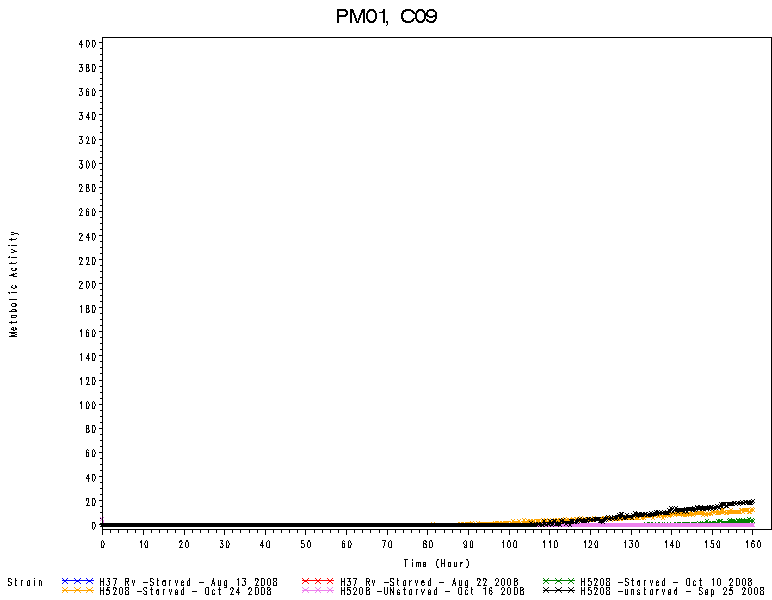

Supplement: Figure S1 — Kinetic curves for all PM plates with Mycobacterium tuberculosis H37Rv and Bj5208 strains. (ZIP) [file pone.0052673.s001.zip › suppl fig 1G H37Rv and Bj5208/Plate01/pm01c09.gif]

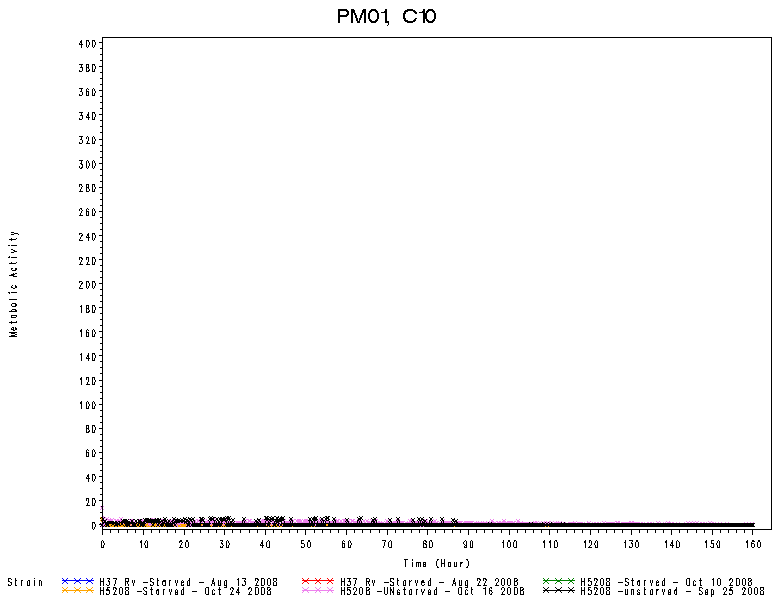

Supplement: Figure S1 — Kinetic curves for all PM plates with Mycobacterium tuberculosis H37Rv and Bj5208 strains. (ZIP) [file pone.0052673.s001.zip › suppl fig 1G H37Rv and Bj5208/Plate01/pm01c10.gif]

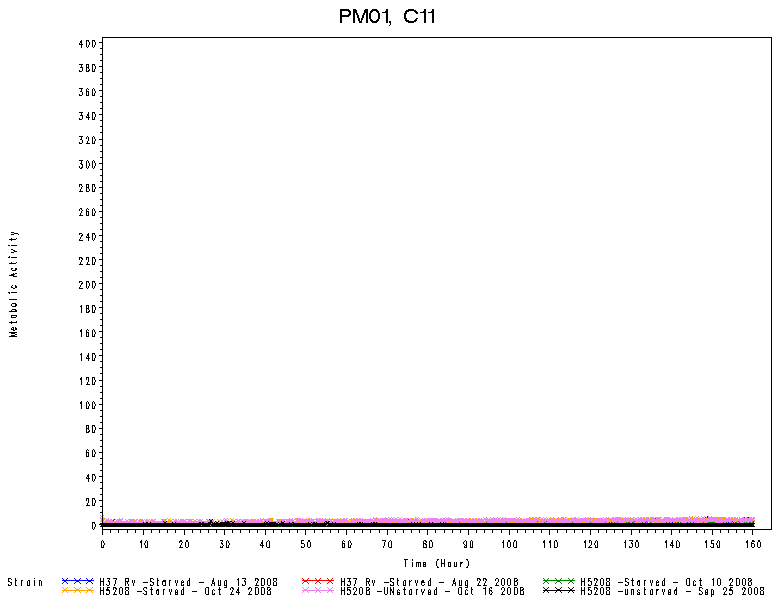

Supplement: Figure S1 — Kinetic curves for all PM plates with Mycobacterium tuberculosis H37Rv and Bj5208 strains. (ZIP) [file pone.0052673.s001.zip › suppl fig 1G H37Rv and Bj5208/Plate01/pm01c11.gif]

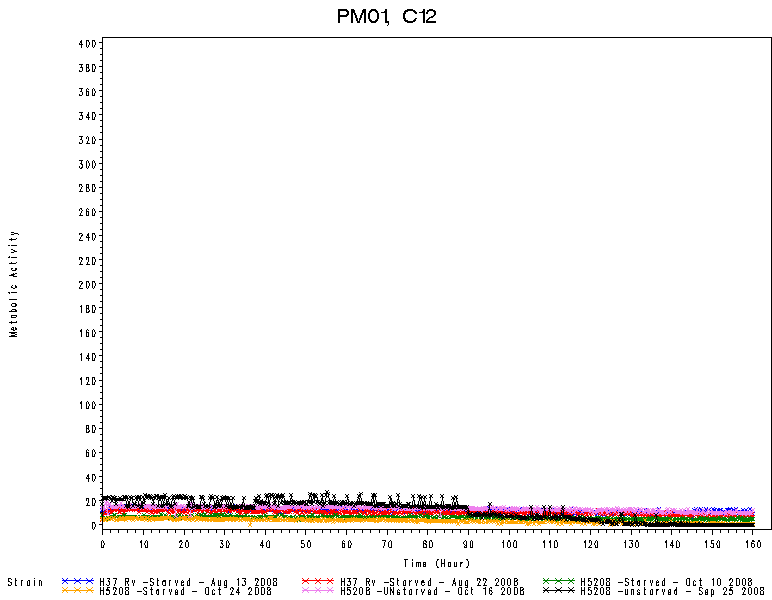

Supplement: Figure S1 — Kinetic curves for all PM plates with Mycobacterium tuberculosis H37Rv and Bj5208 strains. (ZIP) [file pone.0052673.s001.zip › suppl fig 1G H37Rv and Bj5208/Plate01/pm01c12.gif]

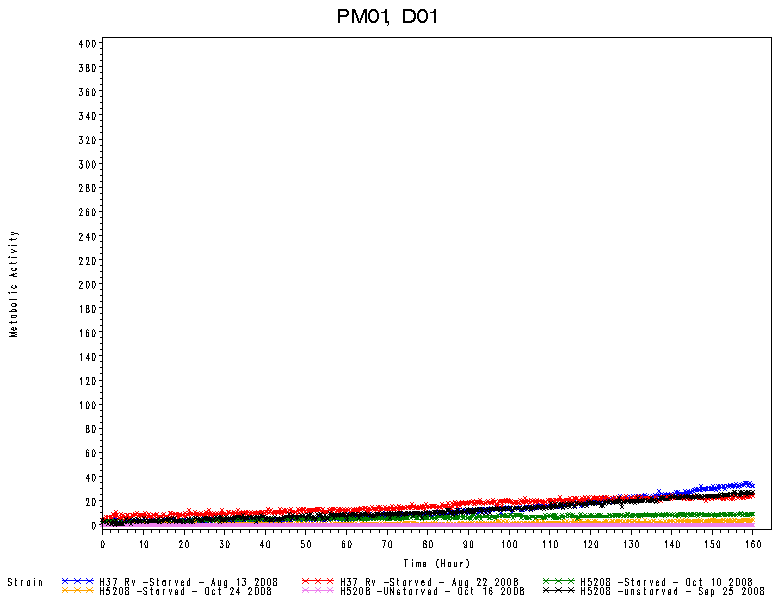

Supplement: Figure S1 — Kinetic curves for all PM plates with Mycobacterium tuberculosis H37Rv and Bj5208 strains. (ZIP) [file pone.0052673.s001.zip › suppl fig 1G H37Rv and Bj5208/Plate01/pm01d01.gif]

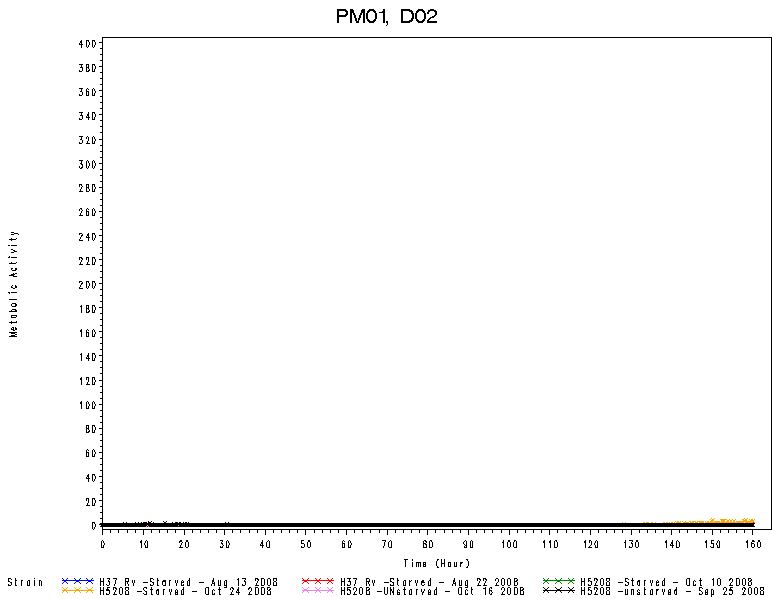

Supplement: Figure S1 — Kinetic curves for all PM plates with Mycobacterium tuberculosis H37Rv and Bj5208 strains. (ZIP) [file pone.0052673.s001.zip › suppl fig 1G H37Rv and Bj5208/Plate01/pm01d02.gif]

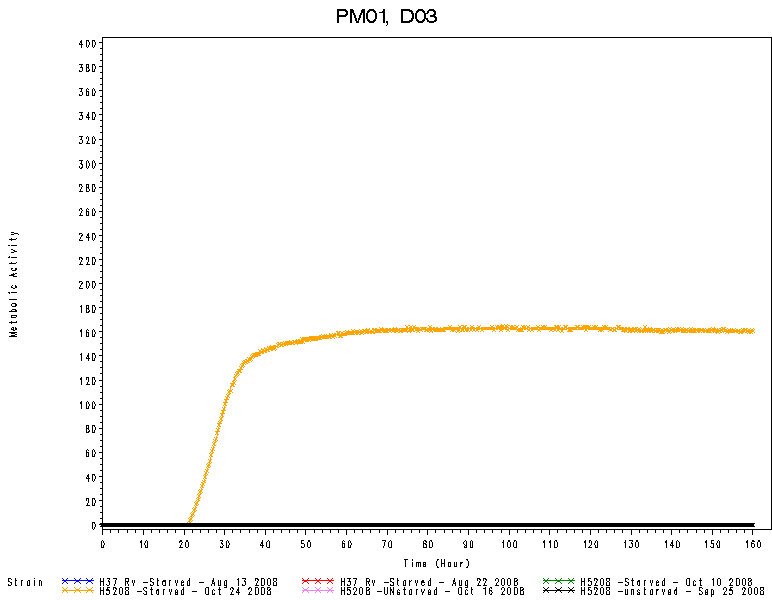

Supplement: Figure S1 — Kinetic curves for all PM plates with Mycobacterium tuberculosis H37Rv and Bj5208 strains. (ZIP) [file pone.0052673.s001.zip › suppl fig 1G H37Rv and Bj5208/Plate01/pm01d03.gif]

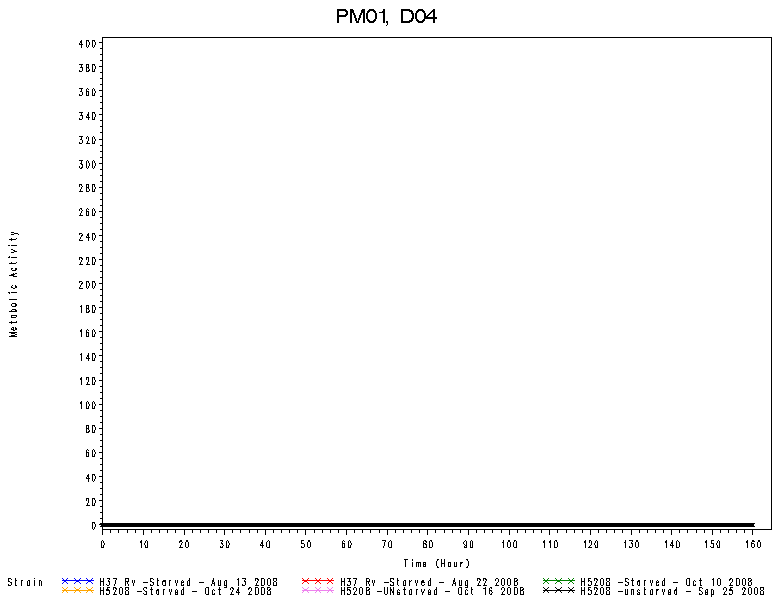

Supplement: Figure S1 — Kinetic curves for all PM plates with Mycobacterium tuberculosis H37Rv and Bj5208 strains. (ZIP) [file pone.0052673.s001.zip › suppl fig 1G H37Rv and Bj5208/Plate01/pm01d04.gif]

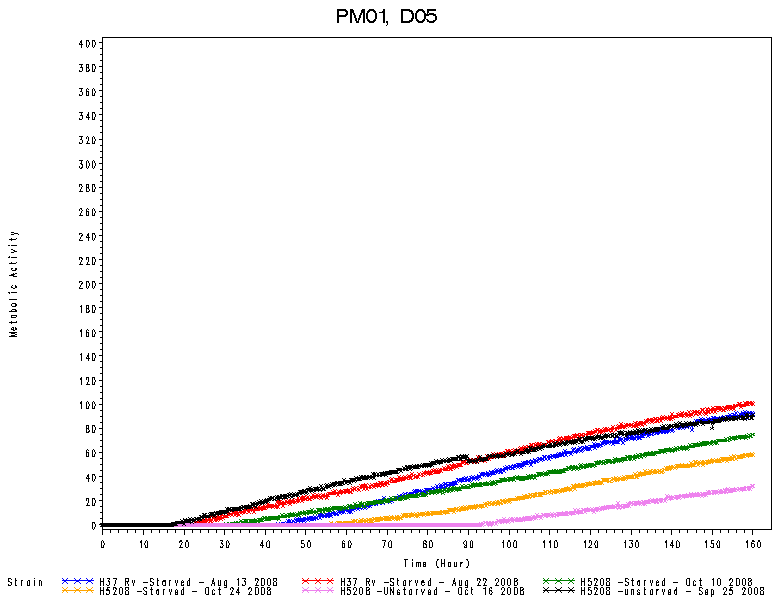

Supplement: Figure S1 — Kinetic curves for all PM plates with Mycobacterium tuberculosis H37Rv and Bj5208 strains. (ZIP) [file pone.0052673.s001.zip › suppl fig 1G H37Rv and Bj5208/Plate01/pm01d05.gif]

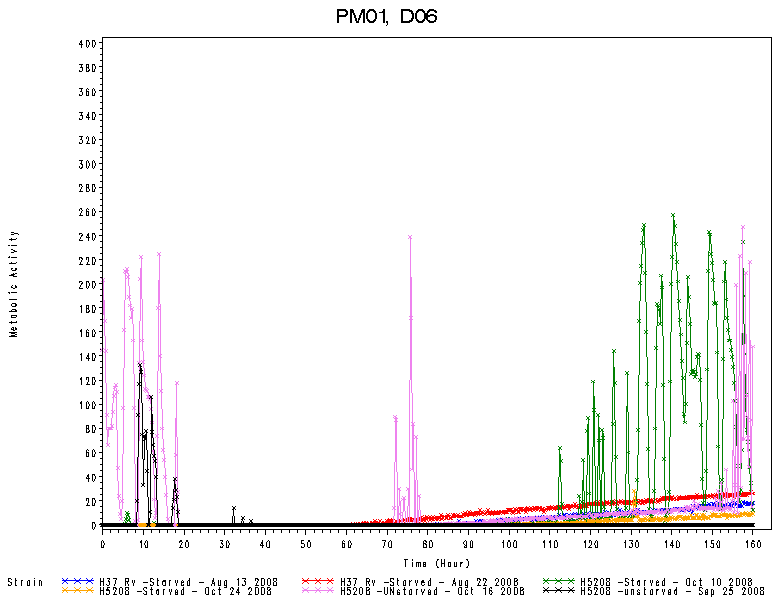

Supplement: Figure S1 — Kinetic curves for all PM plates with Mycobacterium tuberculosis H37Rv and Bj5208 strains. (ZIP) [file pone.0052673.s001.zip › suppl fig 1G H37Rv and Bj5208/Plate01/pm01d06.gif]

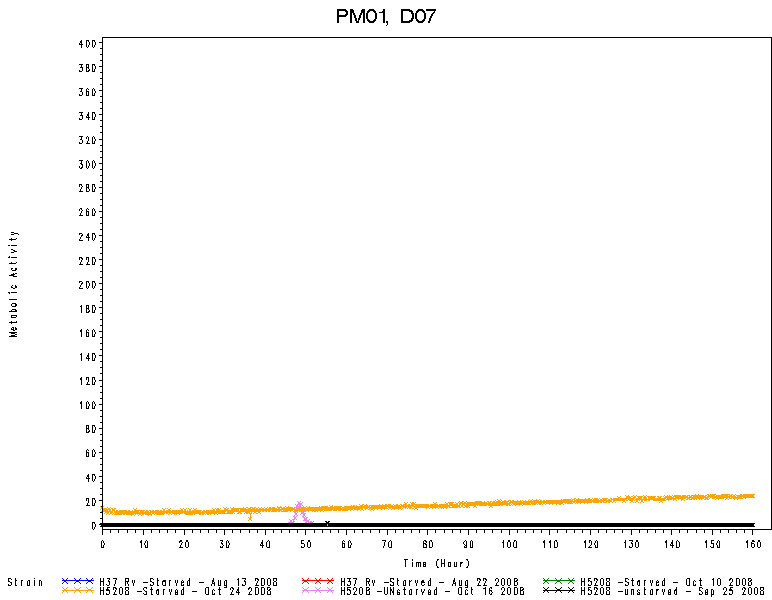

Supplement: Figure S1 — Kinetic curves for all PM plates with Mycobacterium tuberculosis H37Rv and Bj5208 strains. (ZIP) [file pone.0052673.s001.zip › suppl fig 1G H37Rv and Bj5208/Plate01/pm01d07.gif]

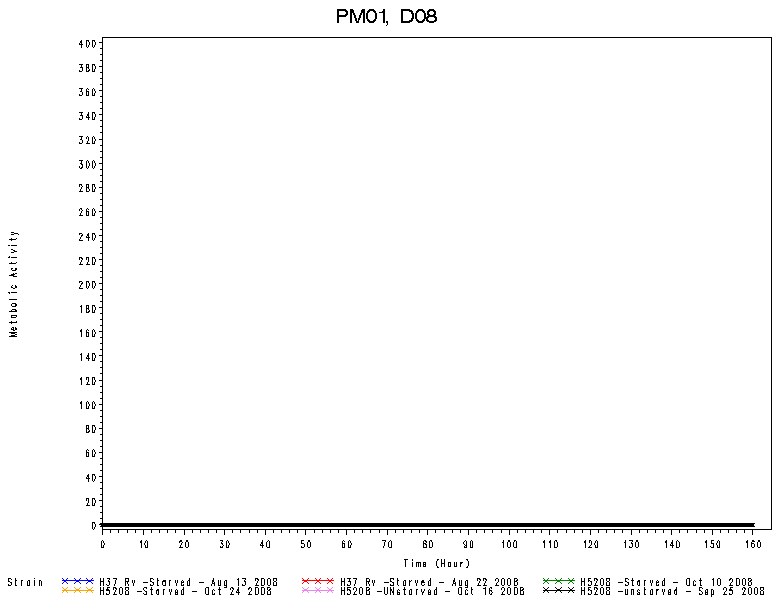

Supplement: Figure S1 — Kinetic curves for all PM plates with Mycobacterium tuberculosis H37Rv and Bj5208 strains. (ZIP) [file pone.0052673.s001.zip › suppl fig 1G H37Rv and Bj5208/Plate01/pm01d08.gif]

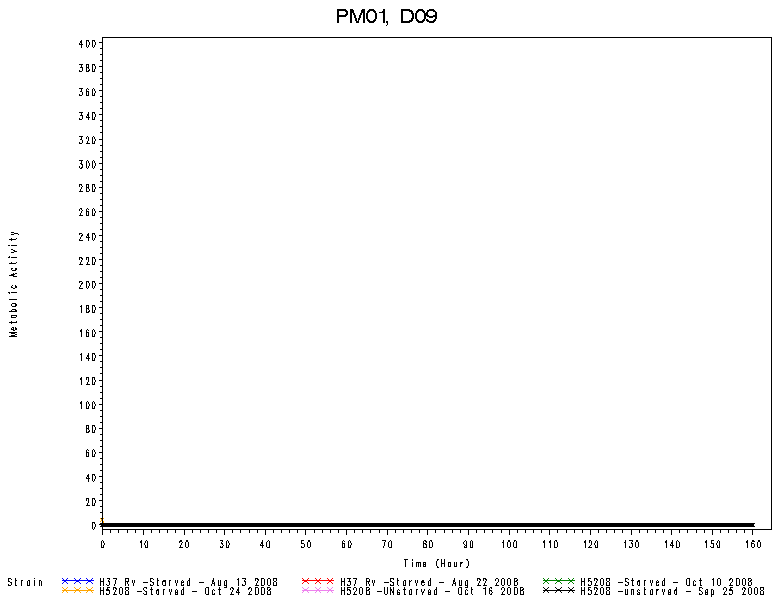

Supplement: Figure S1 — Kinetic curves for all PM plates with Mycobacterium tuberculosis H37Rv and Bj5208 strains. (ZIP) [file pone.0052673.s001.zip › suppl fig 1G H37Rv and Bj5208/Plate01/pm01d09.gif]

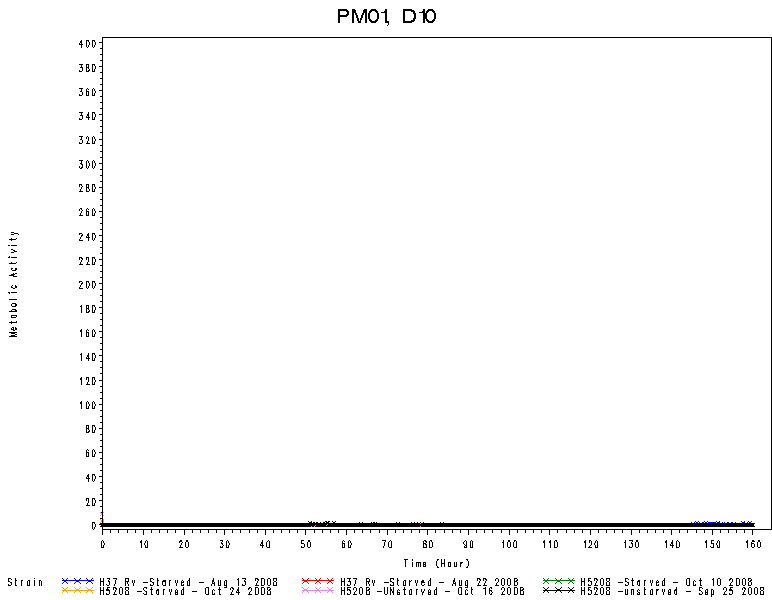

Supplement: Figure S1 — Kinetic curves for all PM plates with Mycobacterium tuberculosis H37Rv and Bj5208 strains. (ZIP) [file pone.0052673.s001.zip › suppl fig 1G H37Rv and Bj5208/Plate01/pm01d10.gif]

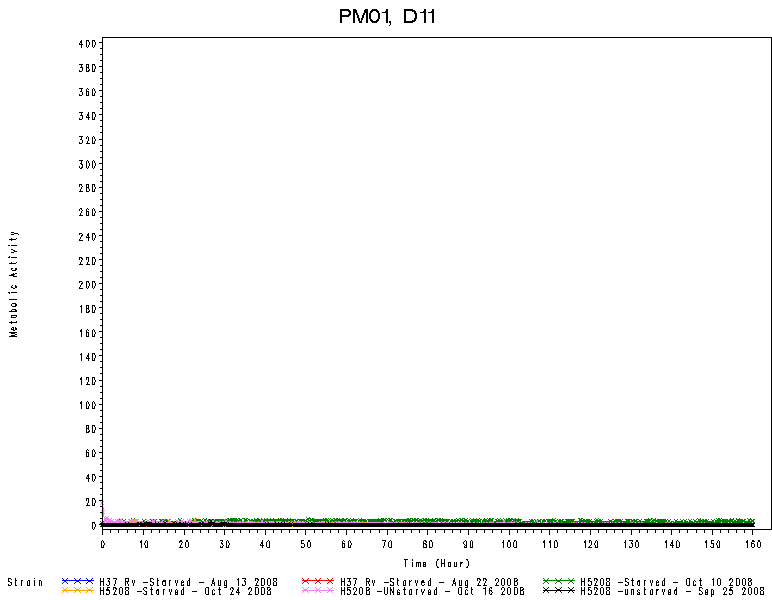

Supplement: Figure S1 — Kinetic curves for all PM plates with Mycobacterium tuberculosis H37Rv and Bj5208 strains. (ZIP) [file pone.0052673.s001.zip › suppl fig 1G H37Rv and Bj5208/Plate01/pm01d11.gif]

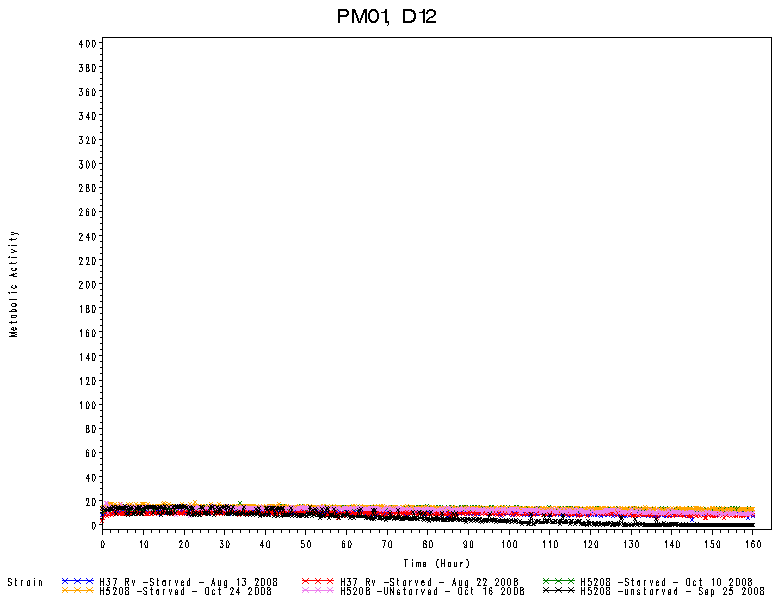

Supplement: Figure S1 — Kinetic curves for all PM plates with Mycobacterium tuberculosis H37Rv and Bj5208 strains. (ZIP) [file pone.0052673.s001.zip › suppl fig 1G H37Rv and Bj5208/Plate01/pm01d12.gif]

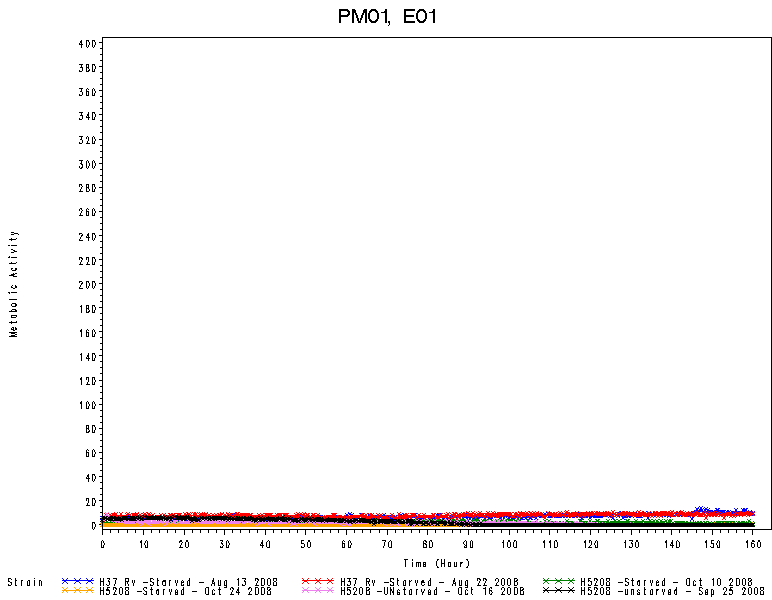

Supplement: Figure S1 — Kinetic curves for all PM plates with Mycobacterium tuberculosis H37Rv and Bj5208 strains. (ZIP) [file pone.0052673.s001.zip › suppl fig 1G H37Rv and Bj5208/Plate01/pm01e01.gif]

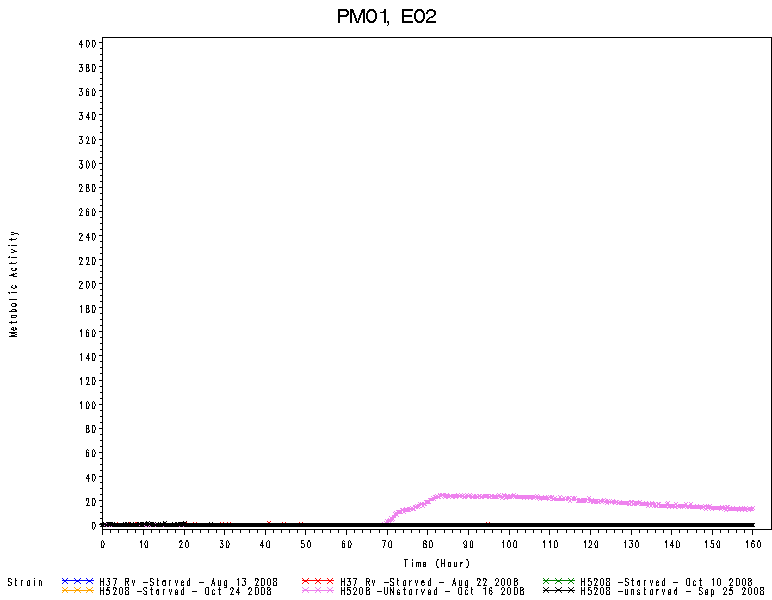

Supplement: Figure S1 — Kinetic curves for all PM plates with Mycobacterium tuberculosis H37Rv and Bj5208 strains. (ZIP) [file pone.0052673.s001.zip › suppl fig 1G H37Rv and Bj5208/Plate01/pm01e02.gif]

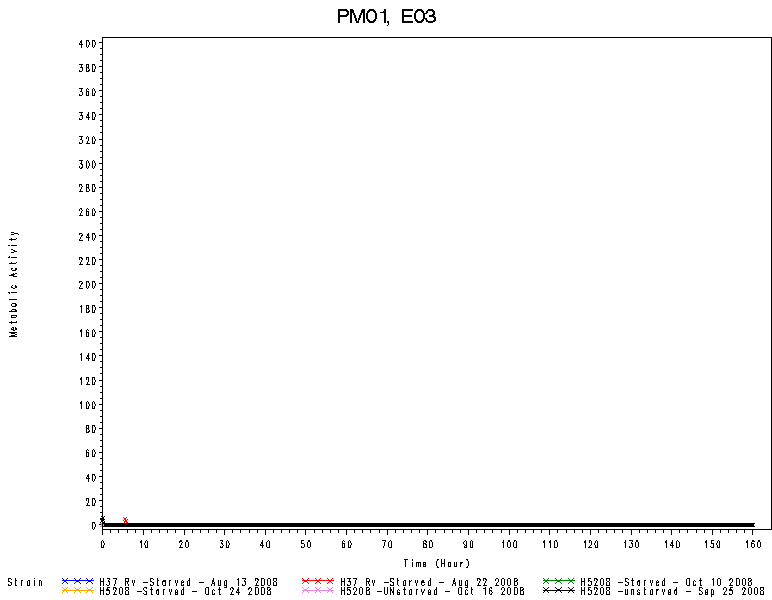

Supplement: Figure S1 — Kinetic curves for all PM plates with Mycobacterium tuberculosis H37Rv and Bj5208 strains. (ZIP) [file pone.0052673.s001.zip › suppl fig 1G H37Rv and Bj5208/Plate01/pm01e03.gif]

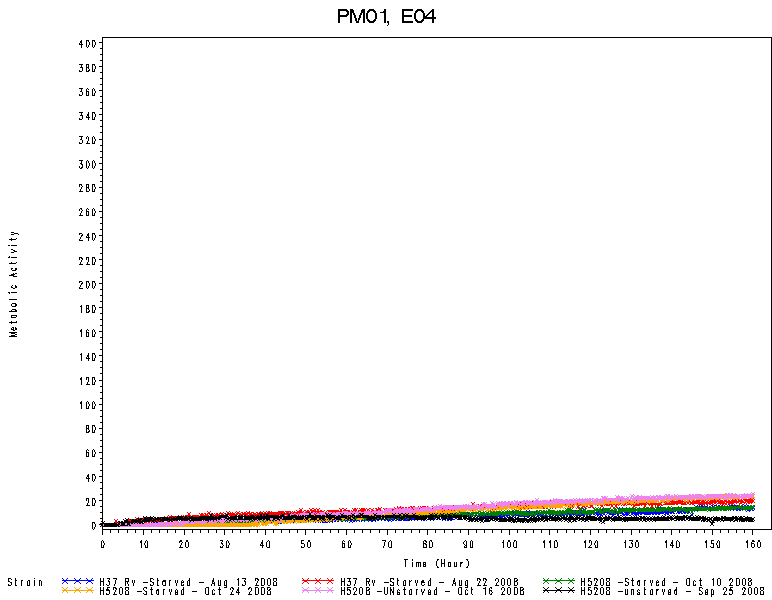

Supplement: Figure S1 — Kinetic curves for all PM plates with Mycobacterium tuberculosis H37Rv and Bj5208 strains. (ZIP) [file pone.0052673.s001.zip › suppl fig 1G H37Rv and Bj5208/Plate01/pm01e04.gif]

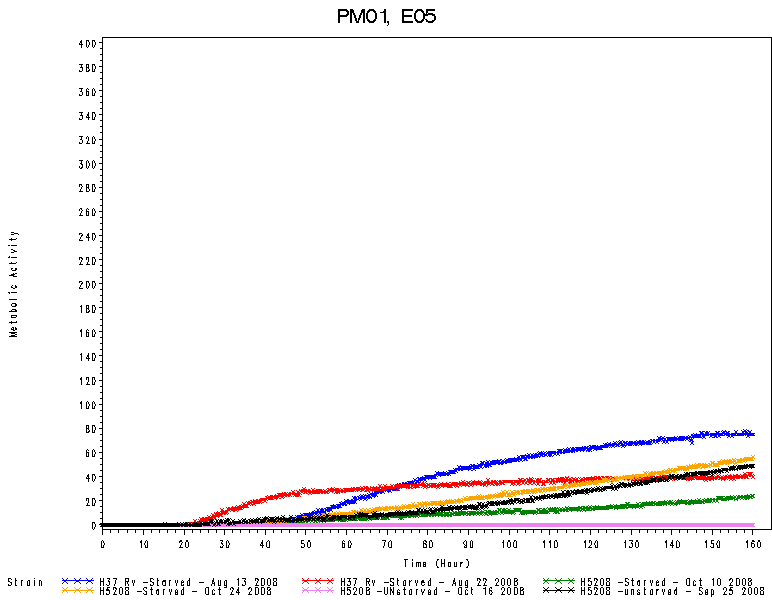

Supplement: Figure S1 — Kinetic curves for all PM plates with Mycobacterium tuberculosis H37Rv and Bj5208 strains. (ZIP) [file pone.0052673.s001.zip › suppl fig 1G H37Rv and Bj5208/Plate01/pm01e05.gif]

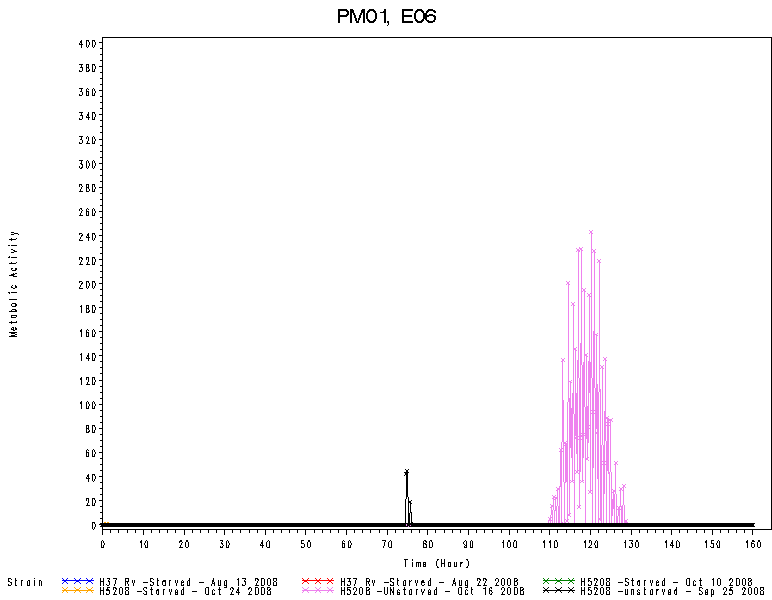

Supplement: Figure S1 — Kinetic curves for all PM plates with Mycobacterium tuberculosis H37Rv and Bj5208 strains. (ZIP) [file pone.0052673.s001.zip › suppl fig 1G H37Rv and Bj5208/Plate01/pm01e06.gif]

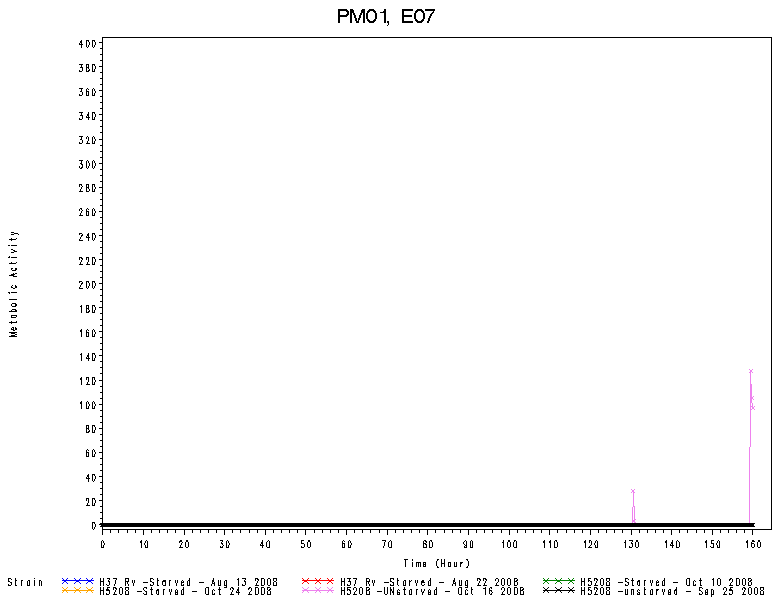

Supplement: Figure S1 — Kinetic curves for all PM plates with Mycobacterium tuberculosis H37Rv and Bj5208 strains. (ZIP) [file pone.0052673.s001.zip › suppl fig 1G H37Rv and Bj5208/Plate01/pm01e07.gif]

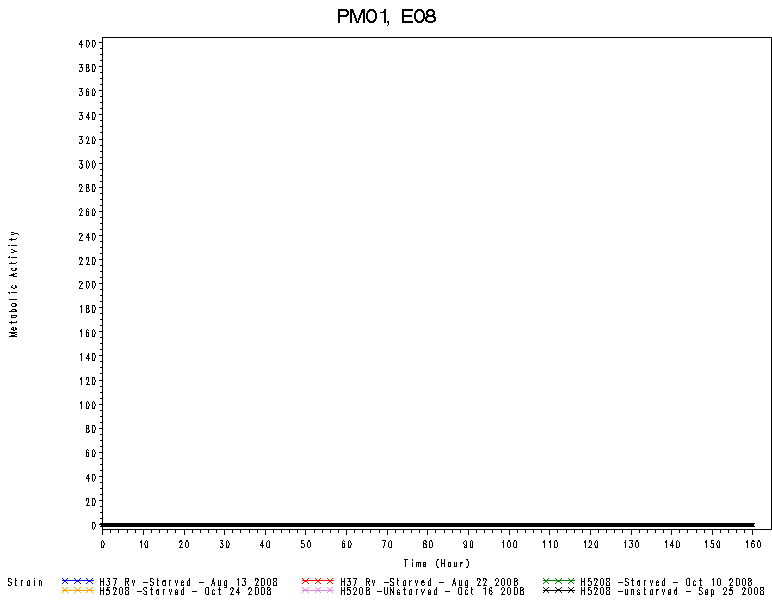

Supplement: Figure S1 — Kinetic curves for all PM plates with Mycobacterium tuberculosis H37Rv and Bj5208 strains. (ZIP) [file pone.0052673.s001.zip › suppl fig 1G H37Rv and Bj5208/Plate01/pm01e08.gif]

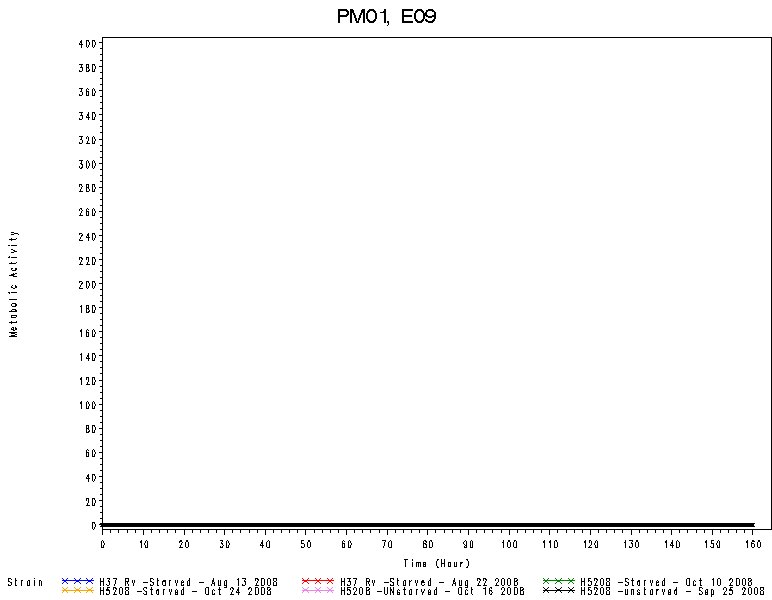

Supplement: Figure S1 — Kinetic curves for all PM plates with Mycobacterium tuberculosis H37Rv and Bj5208 strains. (ZIP) [file pone.0052673.s001.zip › suppl fig 1G H37Rv and Bj5208/Plate01/pm01e09.gif]

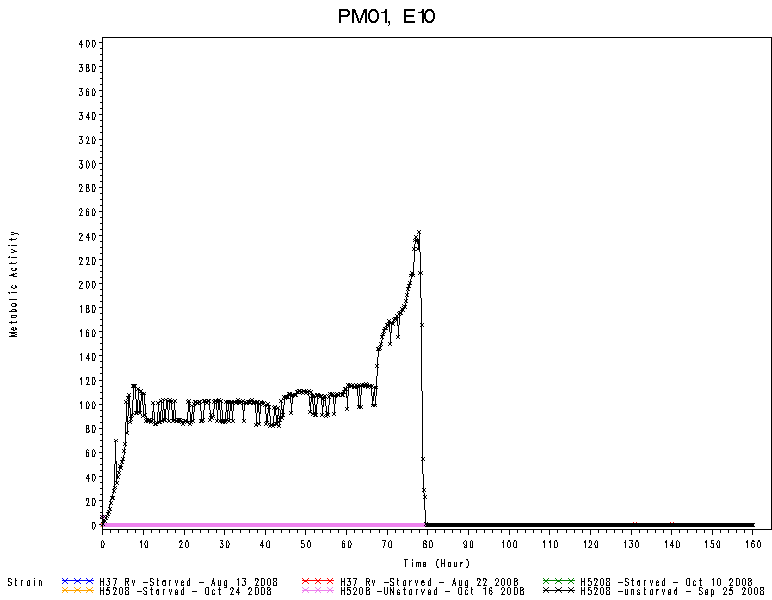

Supplement: Figure S1 — Kinetic curves for all PM plates with Mycobacterium tuberculosis H37Rv and Bj5208 strains. (ZIP) [file pone.0052673.s001.zip › suppl fig 1G H37Rv and Bj5208/Plate01/pm01e10.gif]

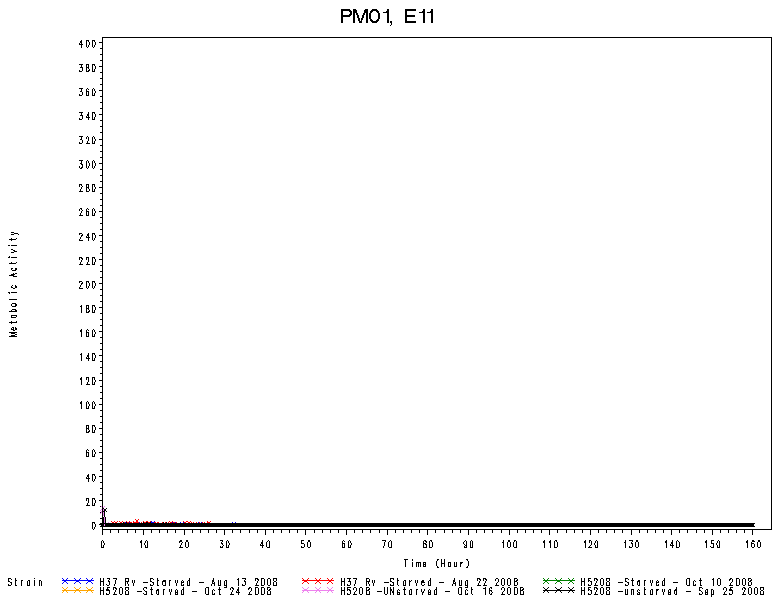

Supplement: Figure S1 — Kinetic curves for all PM plates with Mycobacterium tuberculosis H37Rv and Bj5208 strains. (ZIP) [file pone.0052673.s001.zip › suppl fig 1G H37Rv and Bj5208/Plate01/pm01e11.gif]

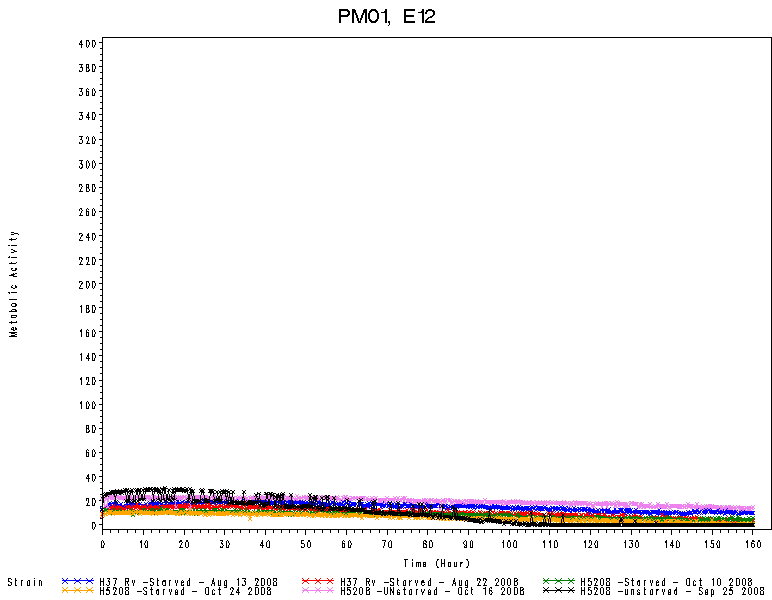

Supplement: Figure S1 — Kinetic curves for all PM plates with Mycobacterium tuberculosis H37Rv and Bj5208 strains. (ZIP) [file pone.0052673.s001.zip › suppl fig 1G H37Rv and Bj5208/Plate01/pm01e12.gif]

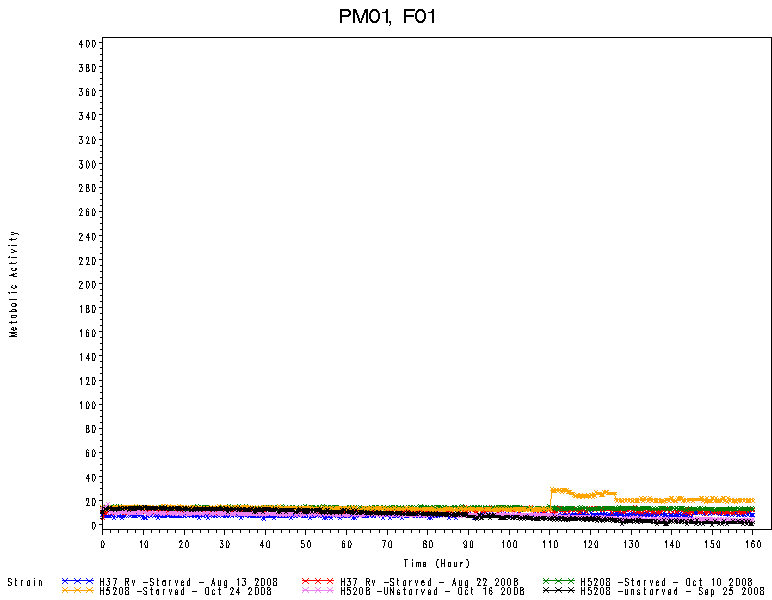

Supplement: Figure S1 — Kinetic curves for all PM plates with Mycobacterium tuberculosis H37Rv and Bj5208 strains. (ZIP) [file pone.0052673.s001.zip › suppl fig 1G H37Rv and Bj5208/Plate01/pm01f01.gif]

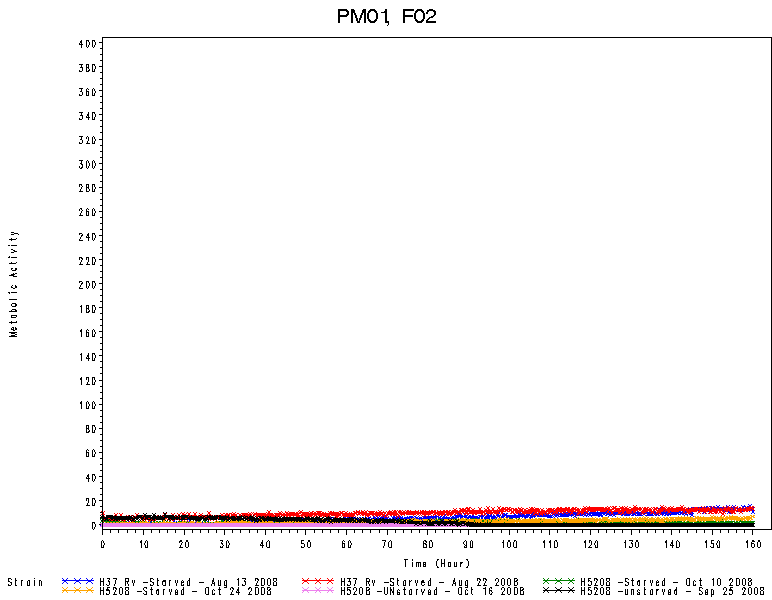

Supplement: Figure S1 — Kinetic curves for all PM plates with Mycobacterium tuberculosis H37Rv and Bj5208 strains. (ZIP) [file pone.0052673.s001.zip › suppl fig 1G H37Rv and Bj5208/Plate01/pm01f02.gif]

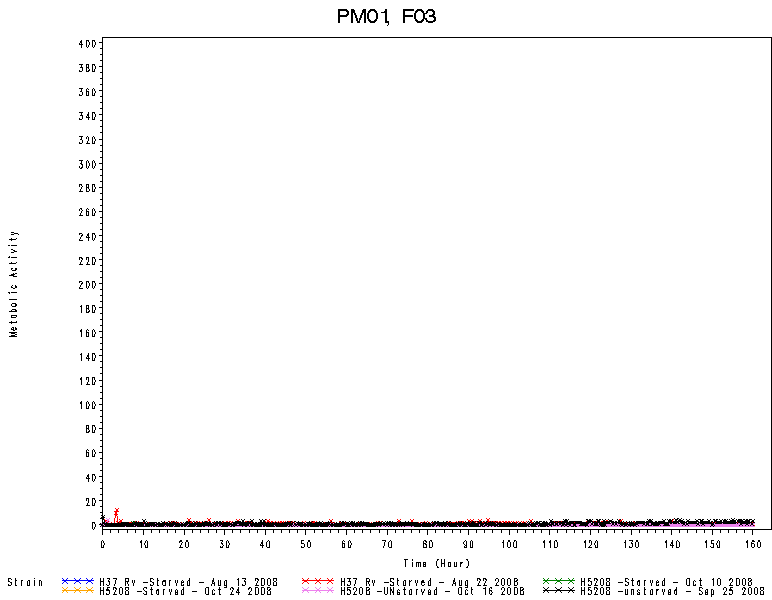

Supplement: Figure S1 — Kinetic curves for all PM plates with Mycobacterium tuberculosis H37Rv and Bj5208 strains. (ZIP) [file pone.0052673.s001.zip › suppl fig 1G H37Rv and Bj5208/Plate01/pm01f03.gif]

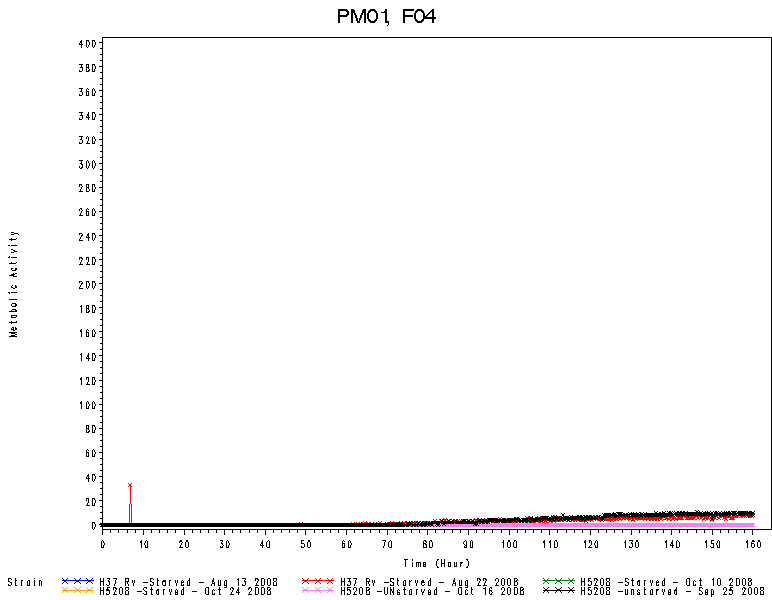

Supplement: Figure S1 — Kinetic curves for all PM plates with Mycobacterium tuberculosis H37Rv and Bj5208 strains. (ZIP) [file pone.0052673.s001.zip › suppl fig 1G H37Rv and Bj5208/Plate01/pm01f04.gif]

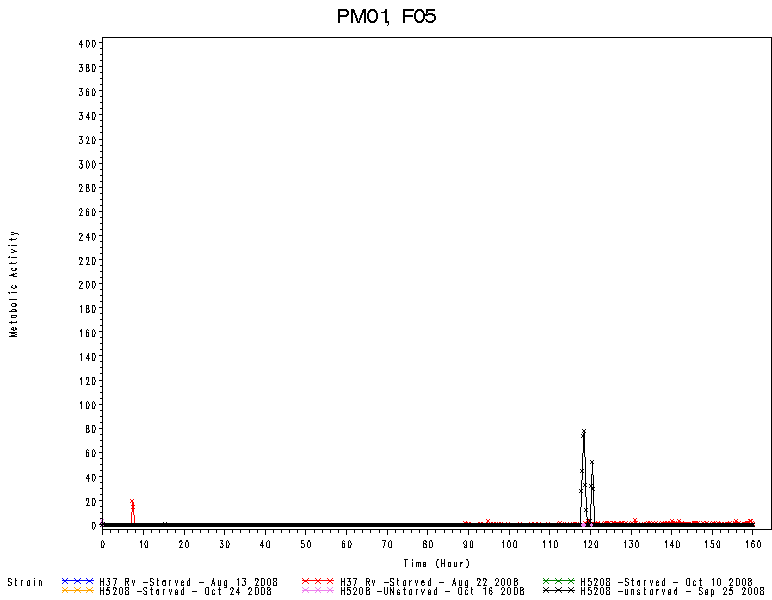

Supplement: Figure S1 — Kinetic curves for all PM plates with Mycobacterium tuberculosis H37Rv and Bj5208 strains. (ZIP) [file pone.0052673.s001.zip › suppl fig 1G H37Rv and Bj5208/Plate01/pm01f05.gif]

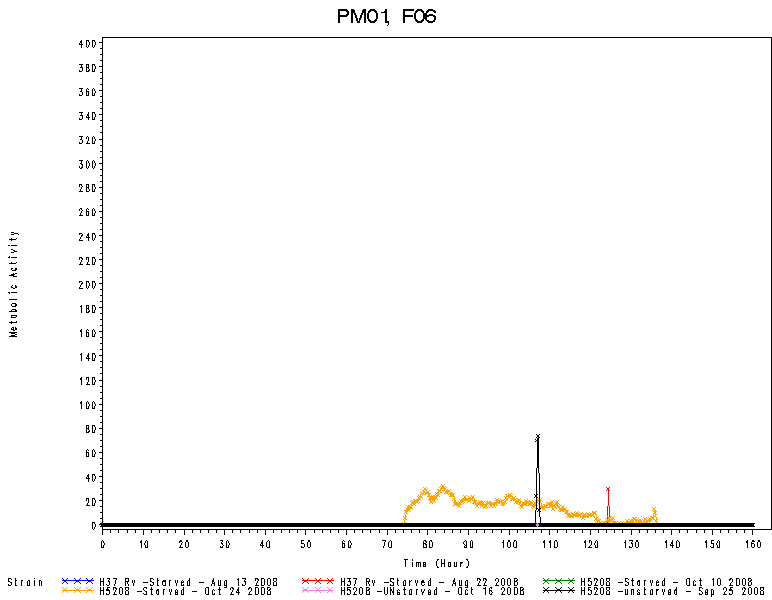

Supplement: Figure S1 — Kinetic curves for all PM plates with Mycobacterium tuberculosis H37Rv and Bj5208 strains. (ZIP) [file pone.0052673.s001.zip › suppl fig 1G H37Rv and Bj5208/Plate01/pm01f06.gif]

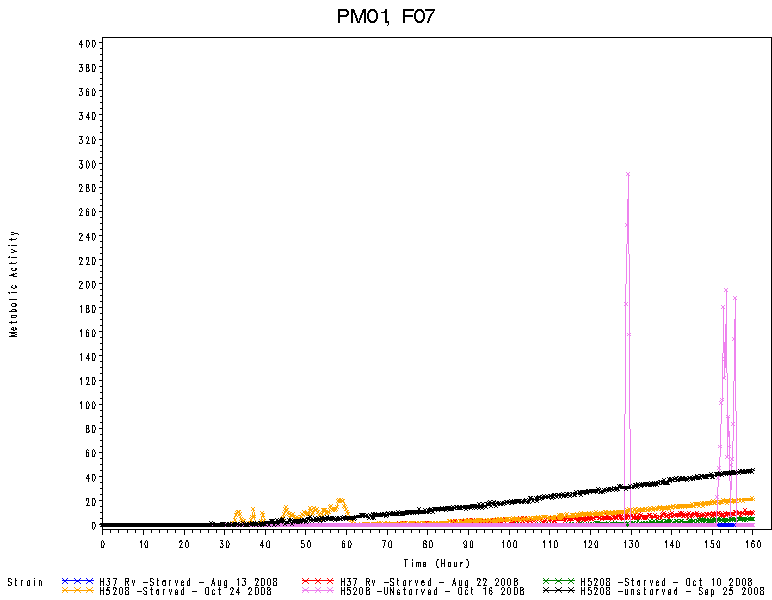

Supplement: Figure S1 — Kinetic curves for all PM plates with Mycobacterium tuberculosis H37Rv and Bj5208 strains. (ZIP) [file pone.0052673.s001.zip › suppl fig 1G H37Rv and Bj5208/Plate01/pm01f07.gif]

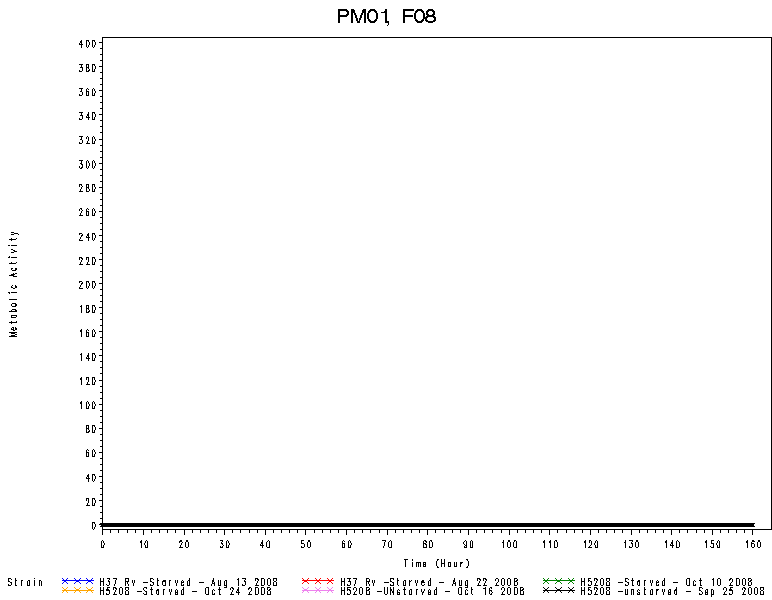

Supplement: Figure S1 — Kinetic curves for all PM plates with Mycobacterium tuberculosis H37Rv and Bj5208 strains. (ZIP) [file pone.0052673.s001.zip › suppl fig 1G H37Rv and Bj5208/Plate01/pm01f08.gif]

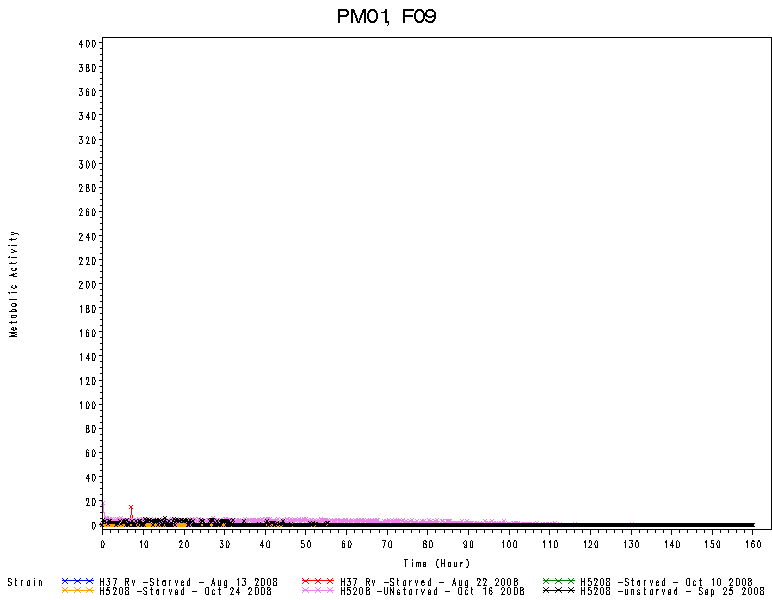

Supplement: Figure S1 — Kinetic curves for all PM plates with Mycobacterium tuberculosis H37Rv and Bj5208 strains. (ZIP) [file pone.0052673.s001.zip › suppl fig 1G H37Rv and Bj5208/Plate01/pm01f09.gif]

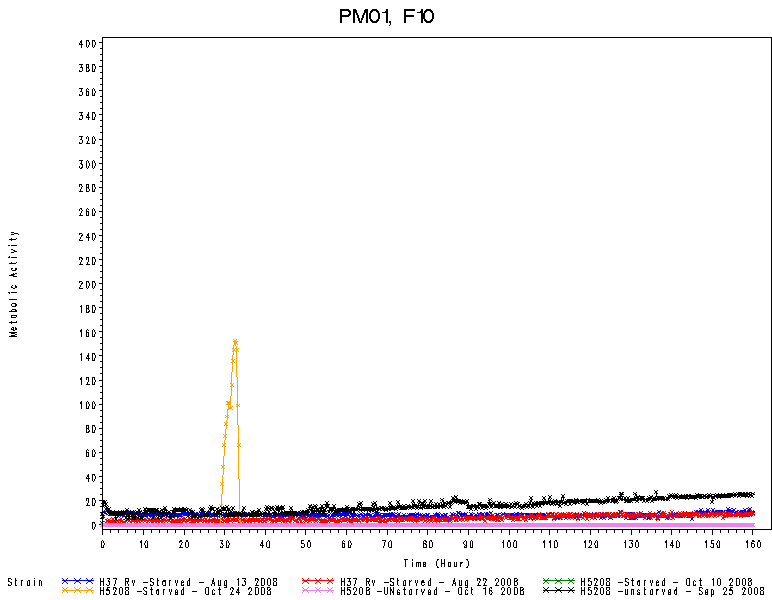

Supplement: Figure S1 — Kinetic curves for all PM plates with Mycobacterium tuberculosis H37Rv and Bj5208 strains. (ZIP) [file pone.0052673.s001.zip › suppl fig 1G H37Rv and Bj5208/Plate01/pm01f10.gif]

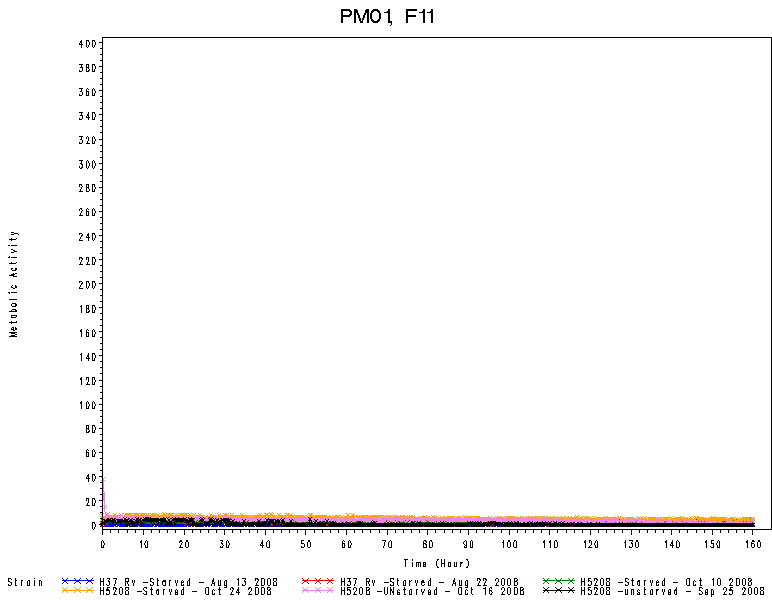

Supplement: Figure S1 — Kinetic curves for all PM plates with Mycobacterium tuberculosis H37Rv and Bj5208 strains. (ZIP) [file pone.0052673.s001.zip › suppl fig 1G H37Rv and Bj5208/Plate01/pm01f11.gif]

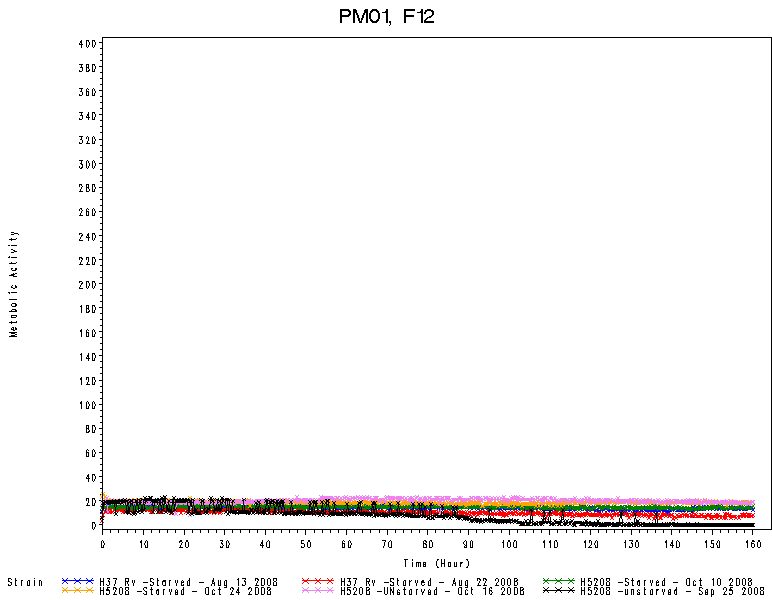

Supplement: Figure S1 — Kinetic curves for all PM plates with Mycobacterium tuberculosis H37Rv and Bj5208 strains. (ZIP) [file pone.0052673.s001.zip › suppl fig 1G H37Rv and Bj5208/Plate01/pm01f12.gif]

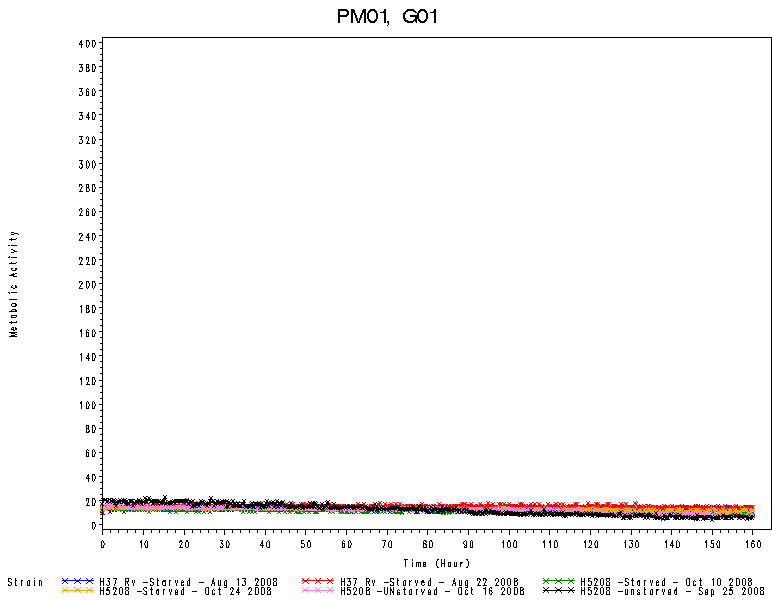

Supplement: Figure S1 — Kinetic curves for all PM plates with Mycobacterium tuberculosis H37Rv and Bj5208 strains. (ZIP) [file pone.0052673.s001.zip › suppl fig 1G H37Rv and Bj5208/Plate01/pm01g01.gif]

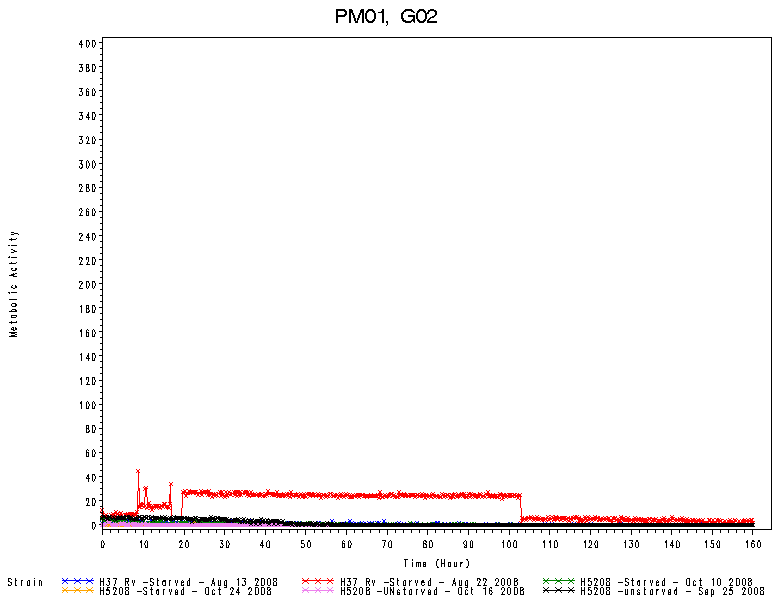

Supplement: Figure S1 — Kinetic curves for all PM plates with Mycobacterium tuberculosis H37Rv and Bj5208 strains. (ZIP) [file pone.0052673.s001.zip › suppl fig 1G H37Rv and Bj5208/Plate01/pm01g02.gif]

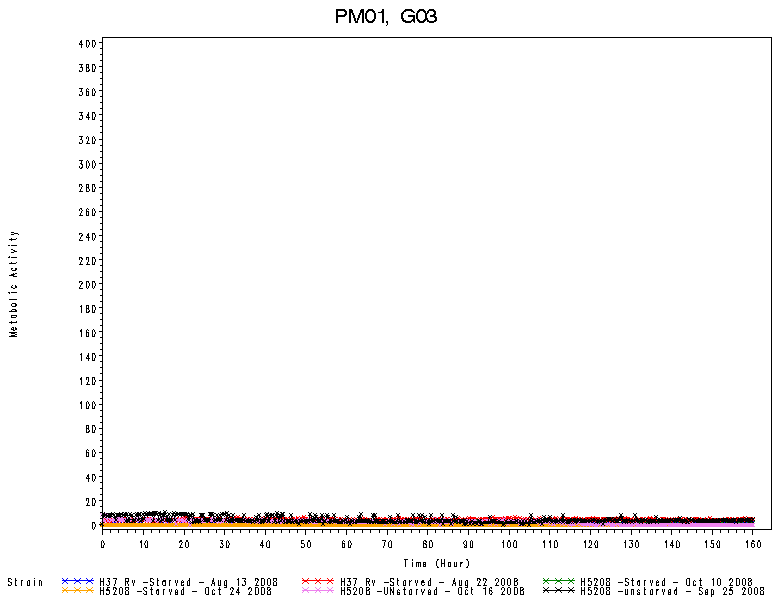

Supplement: Figure S1 — Kinetic curves for all PM plates with Mycobacterium tuberculosis H37Rv and Bj5208 strains. (ZIP) [file pone.0052673.s001.zip › suppl fig 1G H37Rv and Bj5208/Plate01/pm01g03.gif]

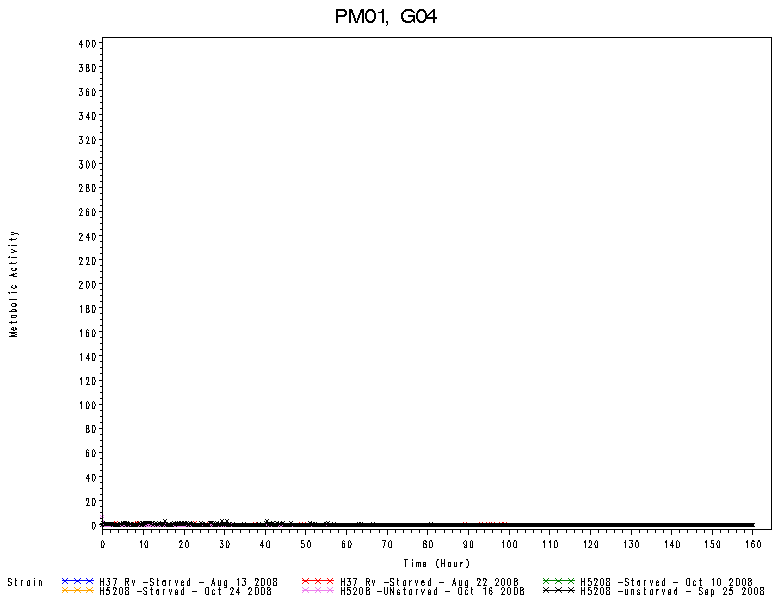

Supplement: Figure S1 — Kinetic curves for all PM plates with Mycobacterium tuberculosis H37Rv and Bj5208 strains. (ZIP) [file pone.0052673.s001.zip › suppl fig 1G H37Rv and Bj5208/Plate01/pm01g04.gif]

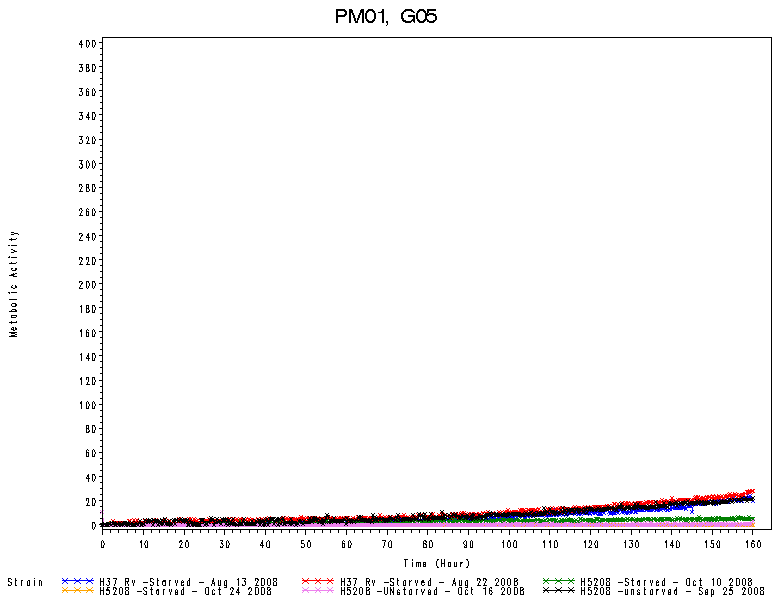

Supplement: Figure S1 — Kinetic curves for all PM plates with Mycobacterium tuberculosis H37Rv and Bj5208 strains. (ZIP) [file pone.0052673.s001.zip › suppl fig 1G H37Rv and Bj5208/Plate01/pm01g05.gif]

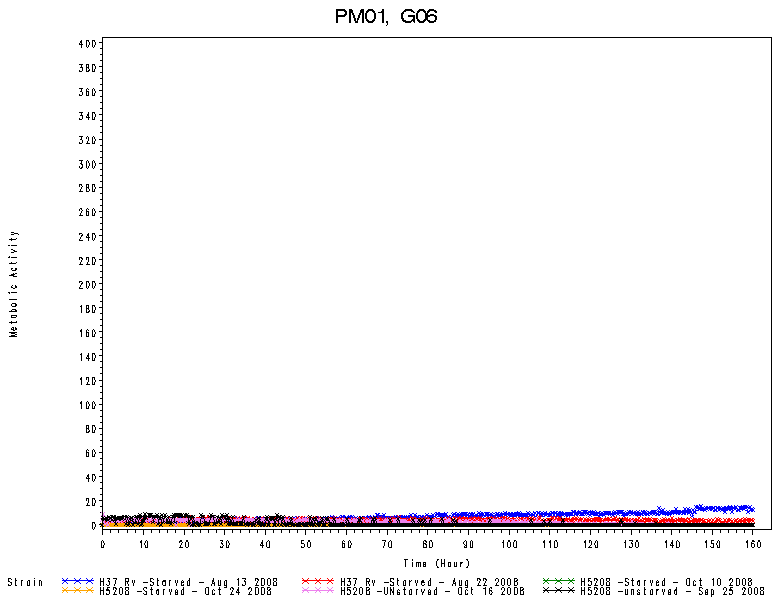

Supplement: Figure S1 — Kinetic curves for all PM plates with Mycobacterium tuberculosis H37Rv and Bj5208 strains. (ZIP) [file pone.0052673.s001.zip › suppl fig 1G H37Rv and Bj5208/Plate01/pm01g06.gif]

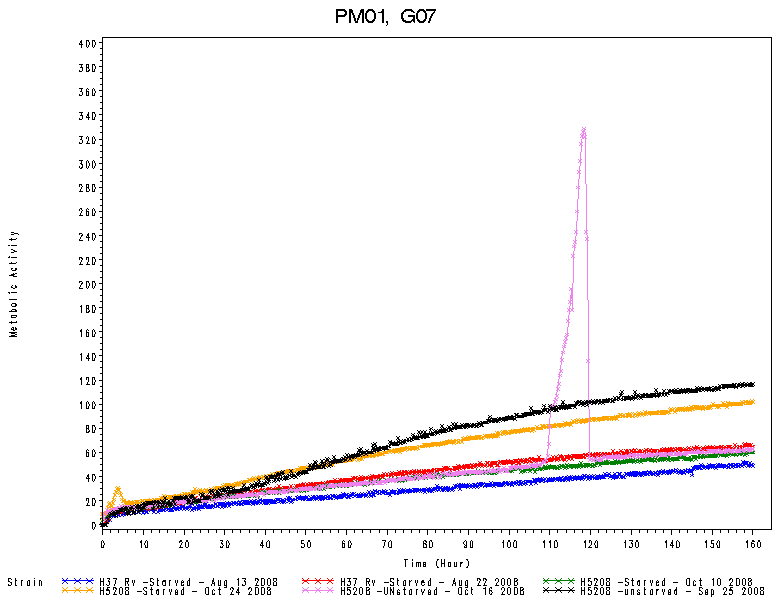

Supplement: Figure S1 — Kinetic curves for all PM plates with Mycobacterium tuberculosis H37Rv and Bj5208 strains. (ZIP) [file pone.0052673.s001.zip › suppl fig 1G H37Rv and Bj5208/Plate01/pm01g07.gif]

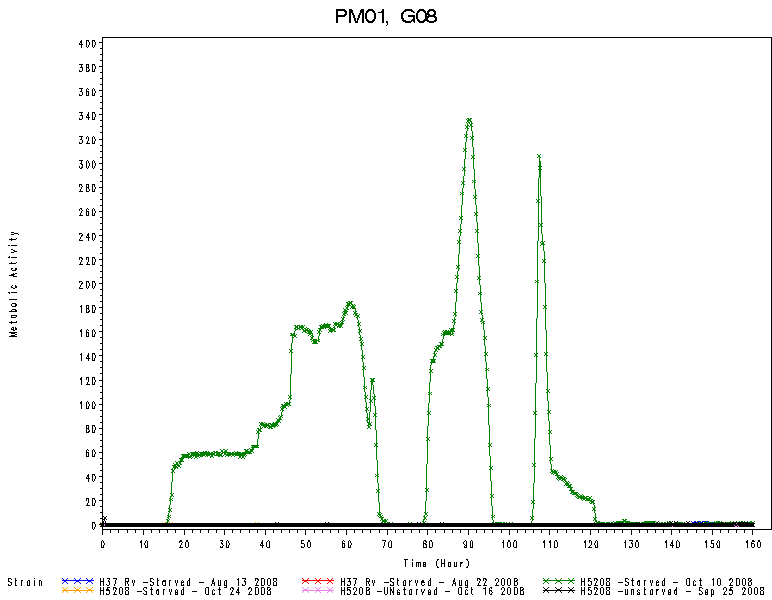

Supplement: Figure S1 — Kinetic curves for all PM plates with Mycobacterium tuberculosis H37Rv and Bj5208 strains. (ZIP) [file pone.0052673.s001.zip › suppl fig 1G H37Rv and Bj5208/Plate01/pm01g08.gif]

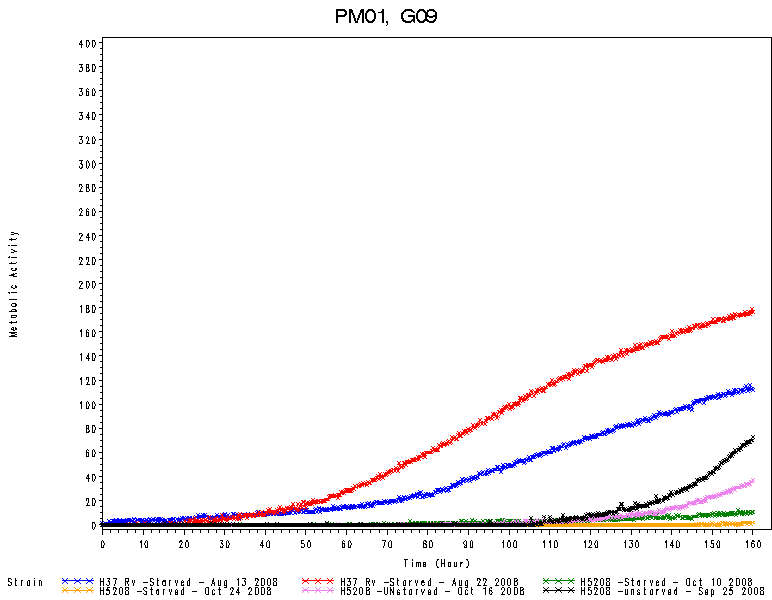

Supplement: Figure S1 — Kinetic curves for all PM plates with Mycobacterium tuberculosis H37Rv and Bj5208 strains. (ZIP) [file pone.0052673.s001.zip › suppl fig 1G H37Rv and Bj5208/Plate01/pm01g09.gif]

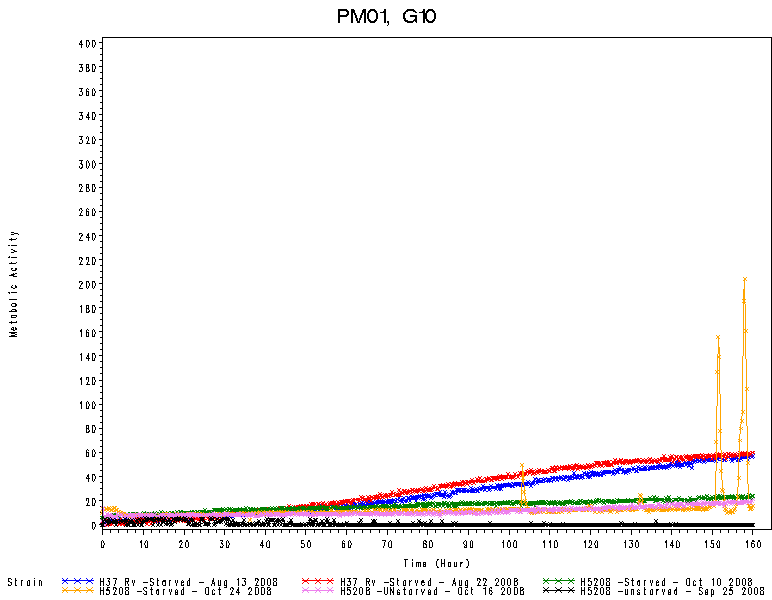

Supplement: Figure S1 — Kinetic curves for all PM plates with Mycobacterium tuberculosis H37Rv and Bj5208 strains. (ZIP) [file pone.0052673.s001.zip › suppl fig 1G H37Rv and Bj5208/Plate01/pm01g10.gif]

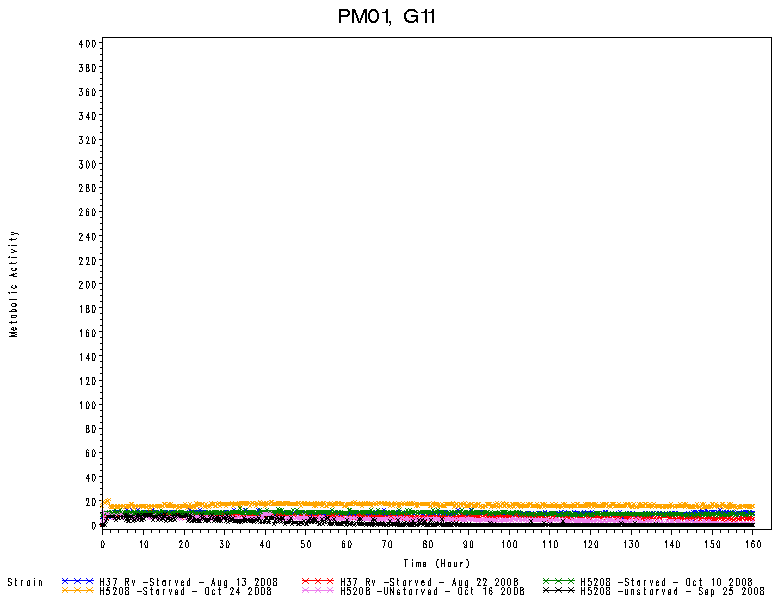

Supplement: Figure S1 — Kinetic curves for all PM plates with Mycobacterium tuberculosis H37Rv and Bj5208 strains. (ZIP) [file pone.0052673.s001.zip › suppl fig 1G H37Rv and Bj5208/Plate01/pm01g11.gif]

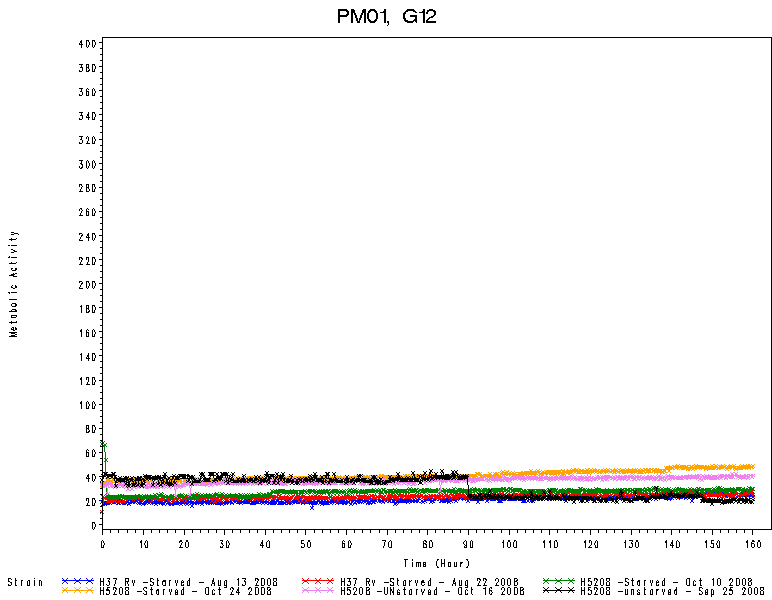

Supplement: Figure S1 — Kinetic curves for all PM plates with Mycobacterium tuberculosis H37Rv and Bj5208 strains. (ZIP) [file pone.0052673.s001.zip › suppl fig 1G H37Rv and Bj5208/Plate01/pm01g12.gif]

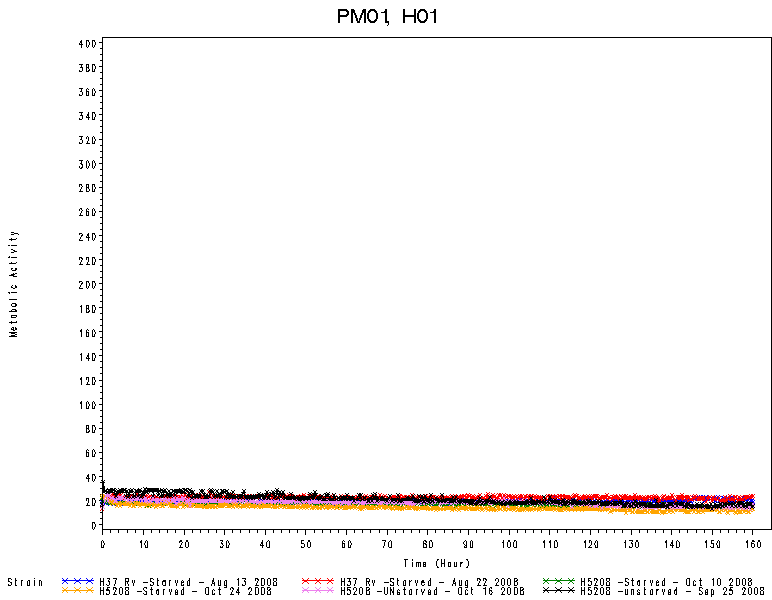

Supplement: Figure S1 — Kinetic curves for all PM plates with Mycobacterium tuberculosis H37Rv and Bj5208 strains. (ZIP) [file pone.0052673.s001.zip › suppl fig 1G H37Rv and Bj5208/Plate01/pm01h01.gif]

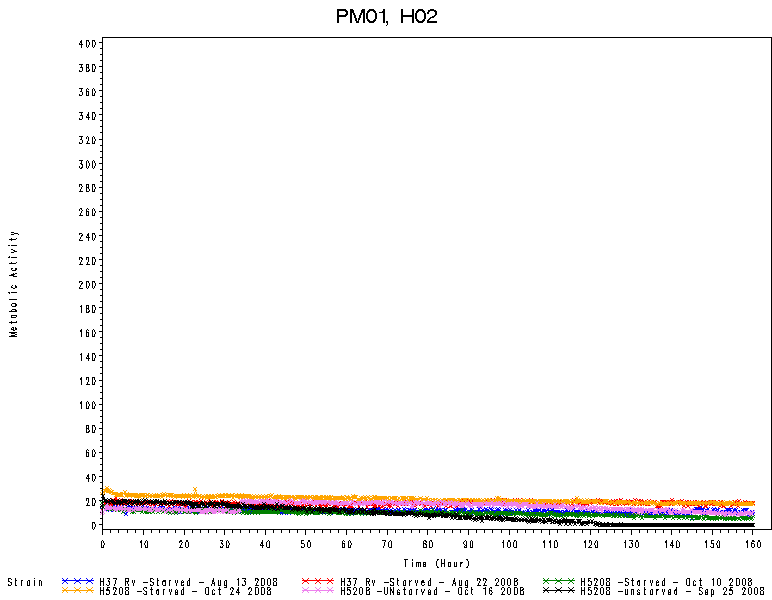

Supplement: Figure S1 — Kinetic curves for all PM plates with Mycobacterium tuberculosis H37Rv and Bj5208 strains. (ZIP) [file pone.0052673.s001.zip › suppl fig 1G H37Rv and Bj5208/Plate01/pm01h02.gif]

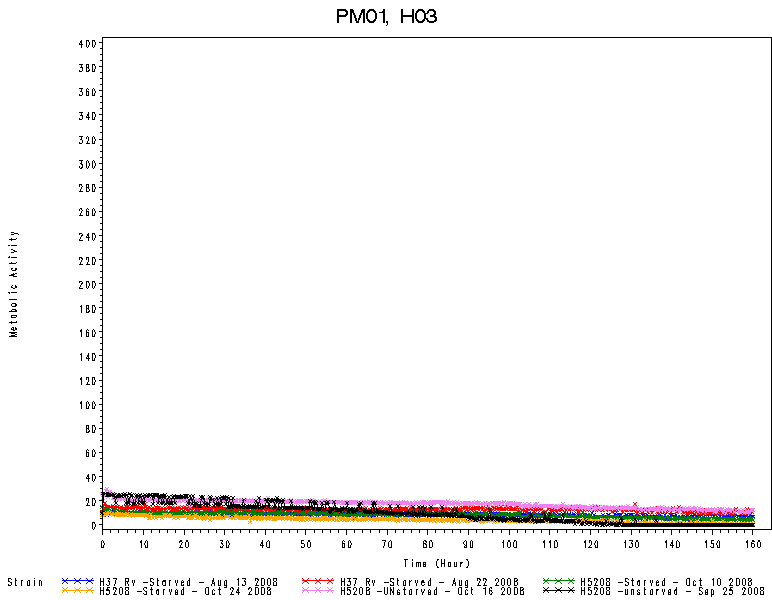

Supplement: Figure S1 — Kinetic curves for all PM plates with Mycobacterium tuberculosis H37Rv and Bj5208 strains. (ZIP) [file pone.0052673.s001.zip › suppl fig 1G H37Rv and Bj5208/Plate01/pm01h03.gif]

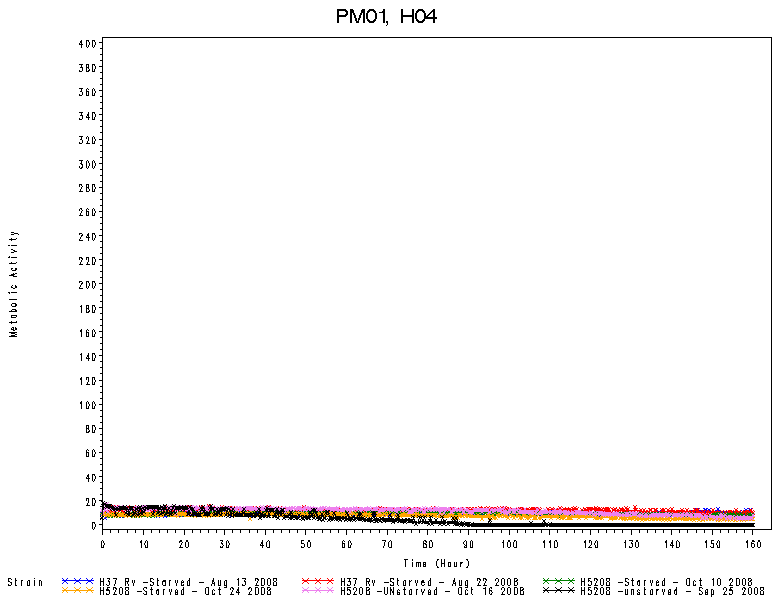

Supplement: Figure S1 — Kinetic curves for all PM plates with Mycobacterium tuberculosis H37Rv and Bj5208 strains. (ZIP) [file pone.0052673.s001.zip › suppl fig 1G H37Rv and Bj5208/Plate01/pm01h04.gif]

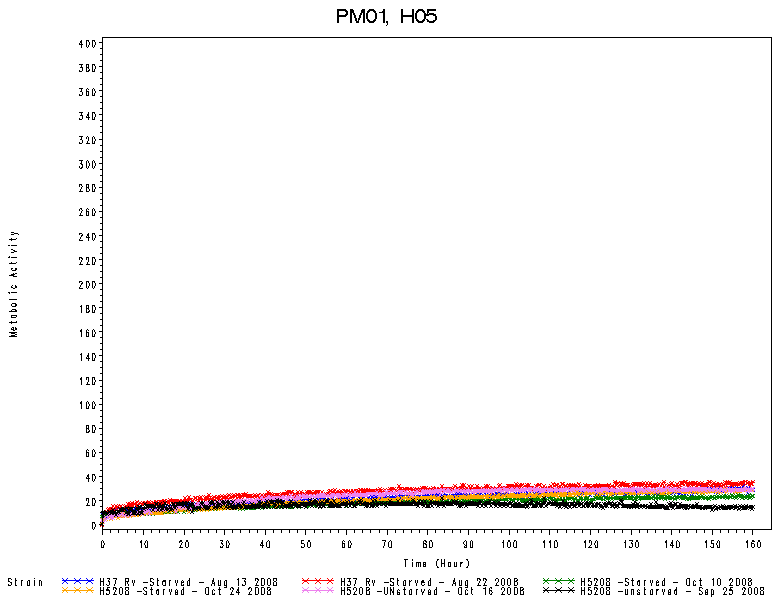

Supplement: Figure S1 — Kinetic curves for all PM plates with Mycobacterium tuberculosis H37Rv and Bj5208 strains. (ZIP) [file pone.0052673.s001.zip › suppl fig 1G H37Rv and Bj5208/Plate01/pm01h05.gif]

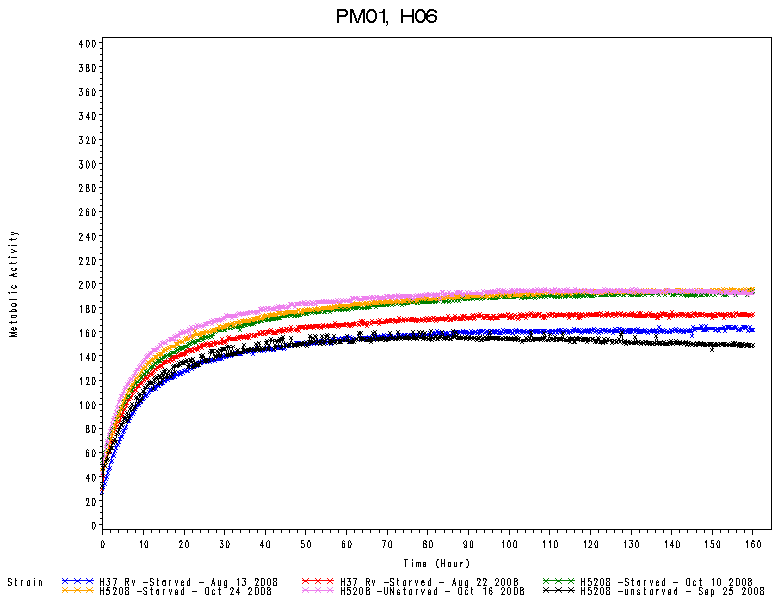

Supplement: Figure S1 — Kinetic curves for all PM plates with Mycobacterium tuberculosis H37Rv and Bj5208 strains. (ZIP) [file pone.0052673.s001.zip › suppl fig 1G H37Rv and Bj5208/Plate01/pm01h06.gif]

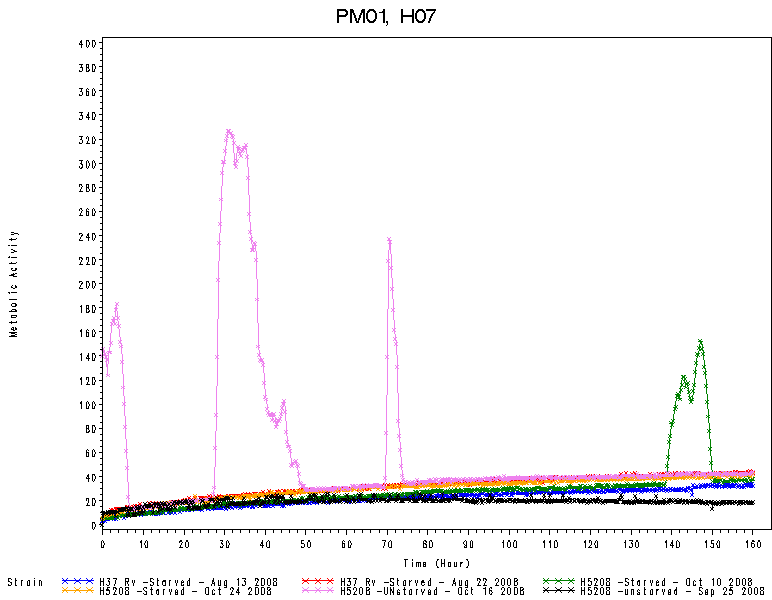

Supplement: Figure S1 — Kinetic curves for all PM plates with Mycobacterium tuberculosis H37Rv and Bj5208 strains. (ZIP) [file pone.0052673.s001.zip › suppl fig 1G H37Rv and Bj5208/Plate01/pm01h07.gif]

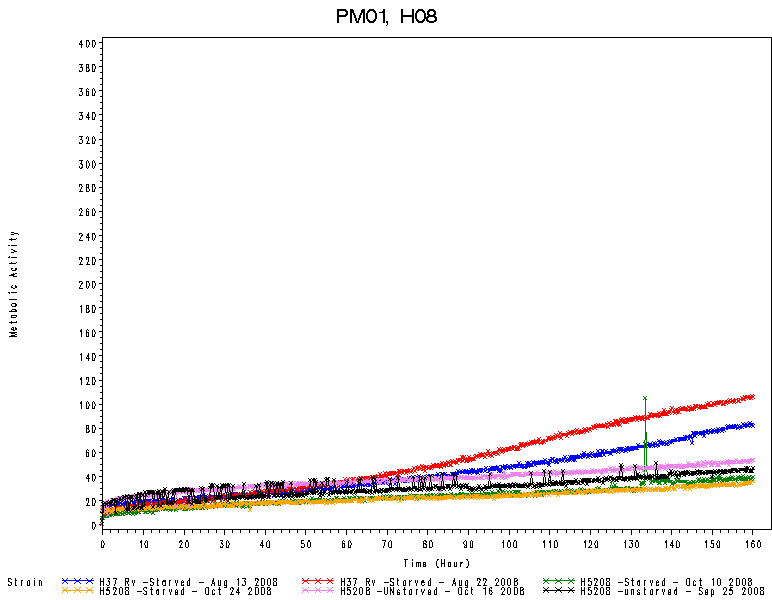

Supplement: Figure S1 — Kinetic curves for all PM plates with Mycobacterium tuberculosis H37Rv and Bj5208 strains. (ZIP) [file pone.0052673.s001.zip › suppl fig 1G H37Rv and Bj5208/Plate01/pm01h08.gif]

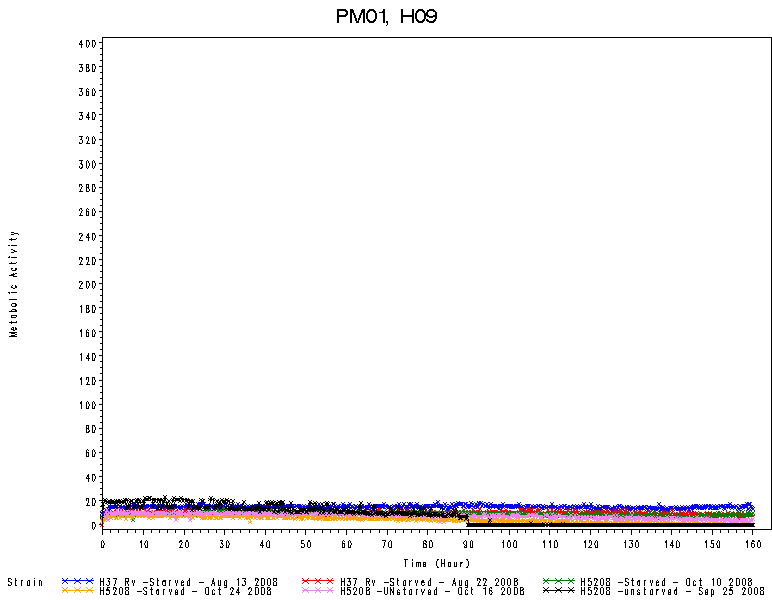

Supplement: Figure S1 — Kinetic curves for all PM plates with Mycobacterium tuberculosis H37Rv and Bj5208 strains. (ZIP) [file pone.0052673.s001.zip › suppl fig 1G H37Rv and Bj5208/Plate01/pm01h09.gif]

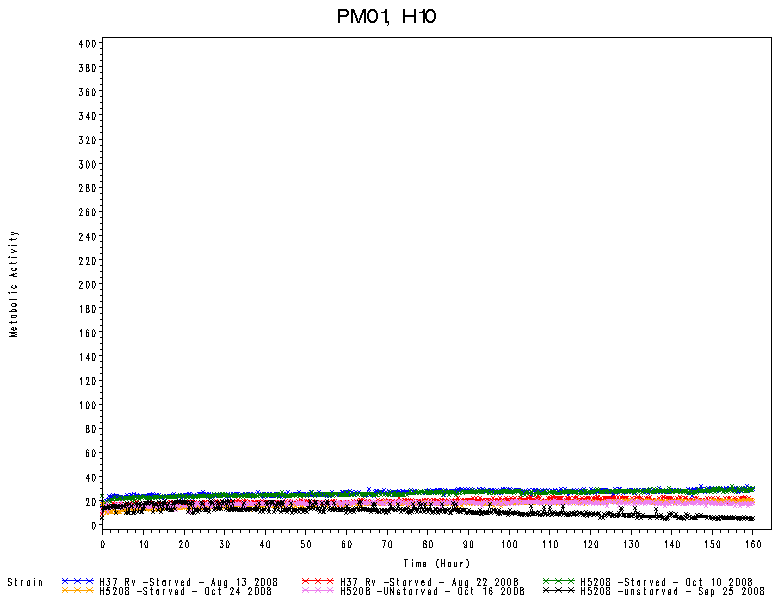

Supplement: Figure S1 — Kinetic curves for all PM plates with Mycobacterium tuberculosis H37Rv and Bj5208 strains. (ZIP) [file pone.0052673.s001.zip › suppl fig 1G H37Rv and Bj5208/Plate01/pm01h10.gif]

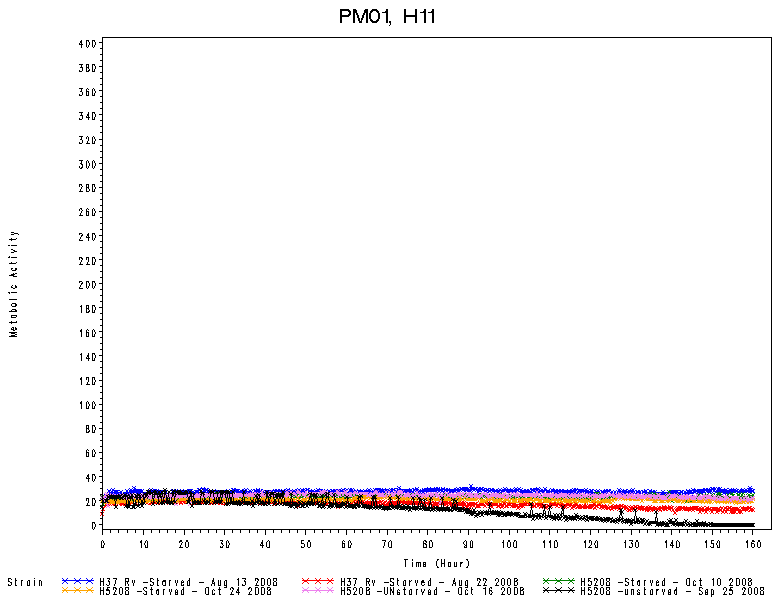

Supplement: Figure S1 — Kinetic curves for all PM plates with Mycobacterium tuberculosis H37Rv and Bj5208 strains. (ZIP) [file pone.0052673.s001.zip › suppl fig 1G H37Rv and Bj5208/Plate01/pm01h11.gif]

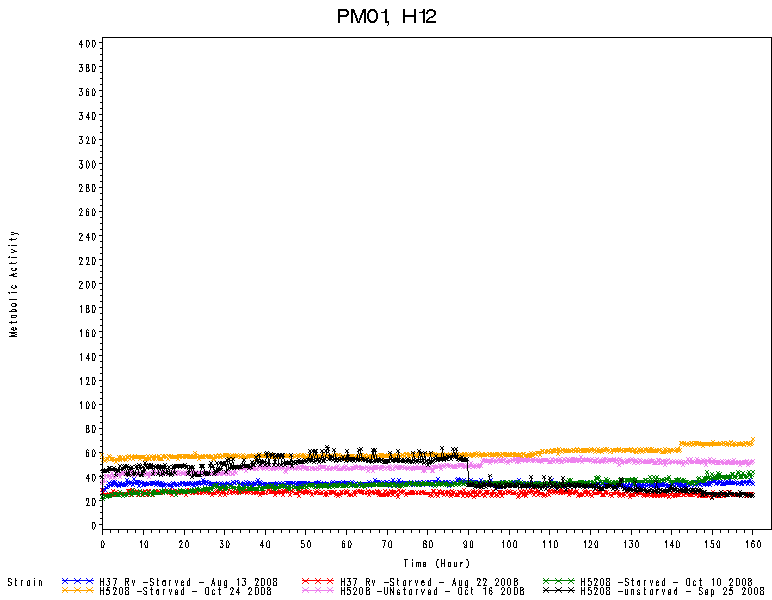

Supplement: Figure S1 — Kinetic curves for all PM plates with Mycobacterium tuberculosis H37Rv and Bj5208 strains. (ZIP) [file pone.0052673.s001.zip › suppl fig 1G H37Rv and Bj5208/Plate01/pm01h12.gif]

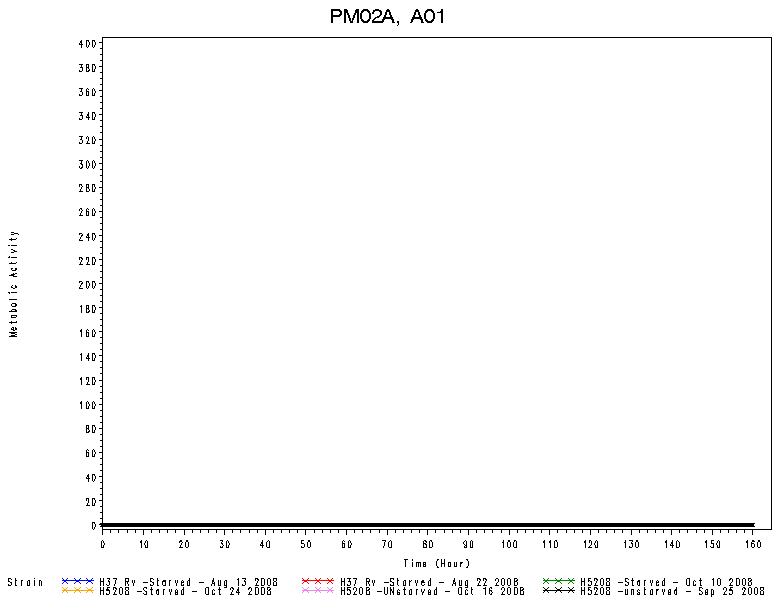

Supplement: Figure S1 — Kinetic curves for all PM plates with Mycobacterium tuberculosis H37Rv and Bj5208 strains. (ZIP) [file pone.0052673.s001.zip › suppl fig 1G H37Rv and Bj5208/Plate02A/pm02aa01.gif]

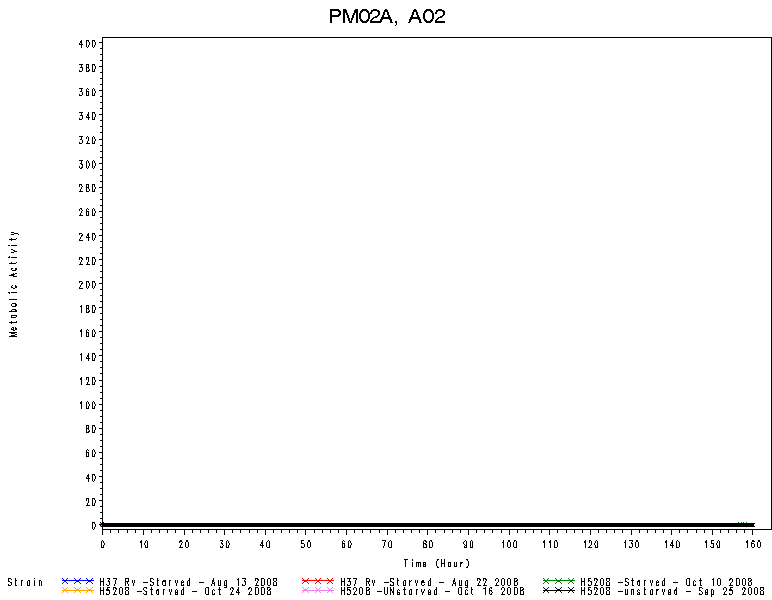

Supplement: Figure S1 — Kinetic curves for all PM plates with Mycobacterium tuberculosis H37Rv and Bj5208 strains. (ZIP) [file pone.0052673.s001.zip › suppl fig 1G H37Rv and Bj5208/Plate02A/pm02aa02.gif]

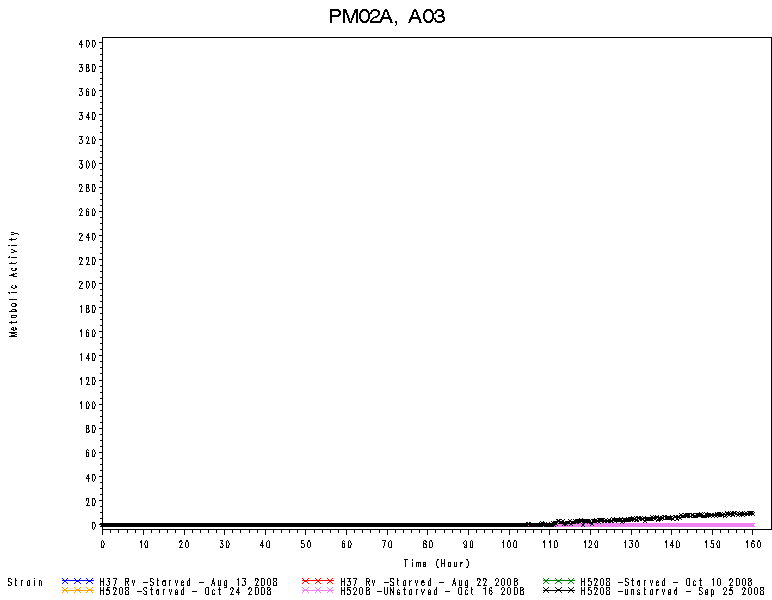

Supplement: Figure S1 — Kinetic curves for all PM plates with Mycobacterium tuberculosis H37Rv and Bj5208 strains. (ZIP) [file pone.0052673.s001.zip › suppl fig 1G H37Rv and Bj5208/Plate02A/pm02aa03.gif]

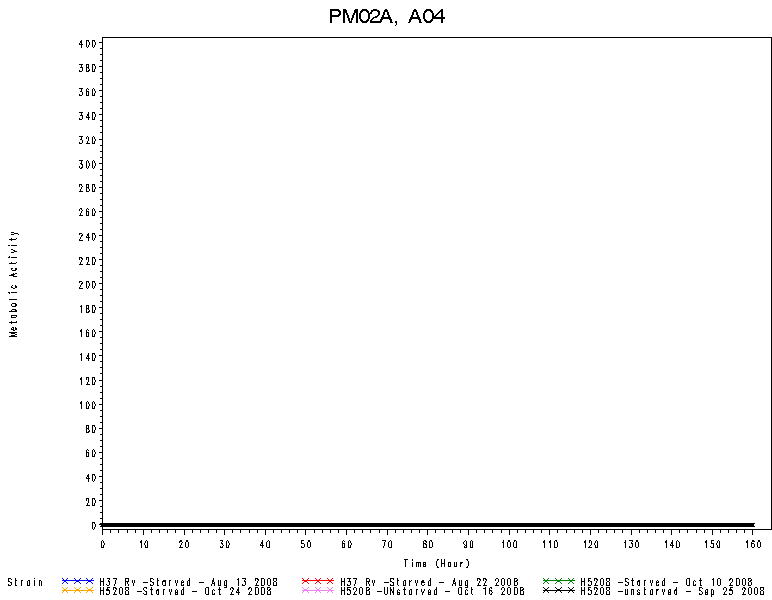

Supplement: Figure S1 — Kinetic curves for all PM plates with Mycobacterium tuberculosis H37Rv and Bj5208 strains. (ZIP) [file pone.0052673.s001.zip › suppl fig 1G H37Rv and Bj5208/Plate02A/pm02aa04.gif]
