# Supplementary figures and images for: High Throughput Phenotypic Analysis of Mycobacterium tuberculosis and Mycobacterium bovis Strains' Metabolism Using Biolog Phenotype Microarrays (part 6 of 11)
Source: PLoS One. 2013 Jan 10;8(1):e52673. doi: 10.1371/journal.pone.0052673 (PMC3542357; doi:10.1371/journal.pone.0052673)

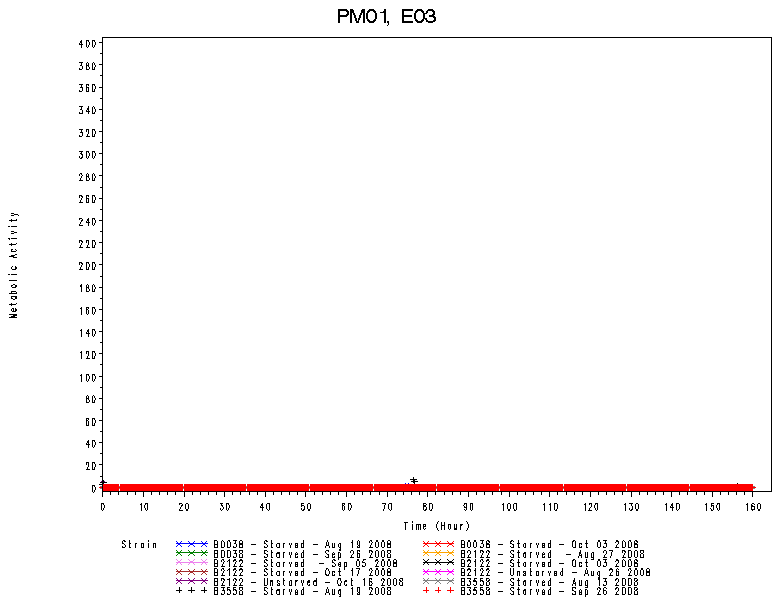

Supplement: Figure S3 — Kinetic curves for all PM plates with Mycobacterium bovis Type 9 strains. (ZIP) [file pone.0052673.s003.zip › suppl fig 3G type 9/Plate01/pm01e03.gif]

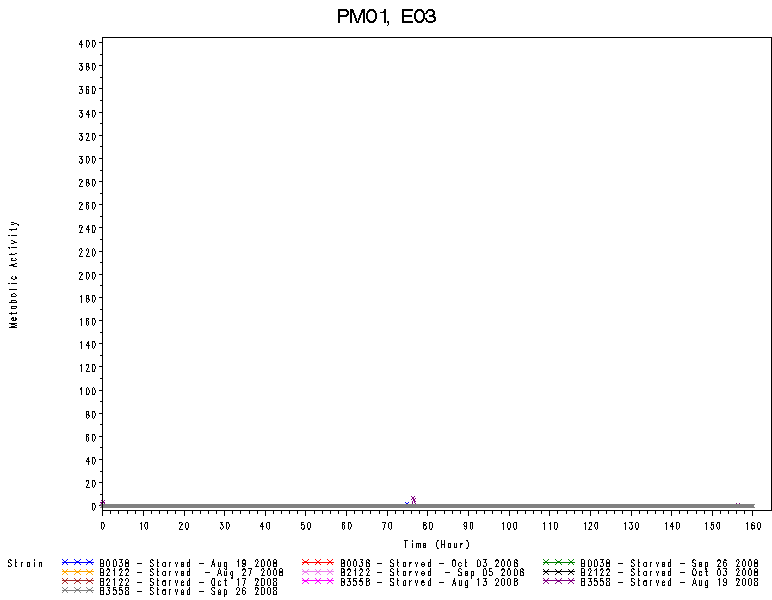

Supplement: Figure S3 — Kinetic curves for all PM plates with Mycobacterium bovis Type 9 strains. (ZIP) [file pone.0052673.s003.zip › suppl fig 3G type 9/Plate01/pm01e031.gif]

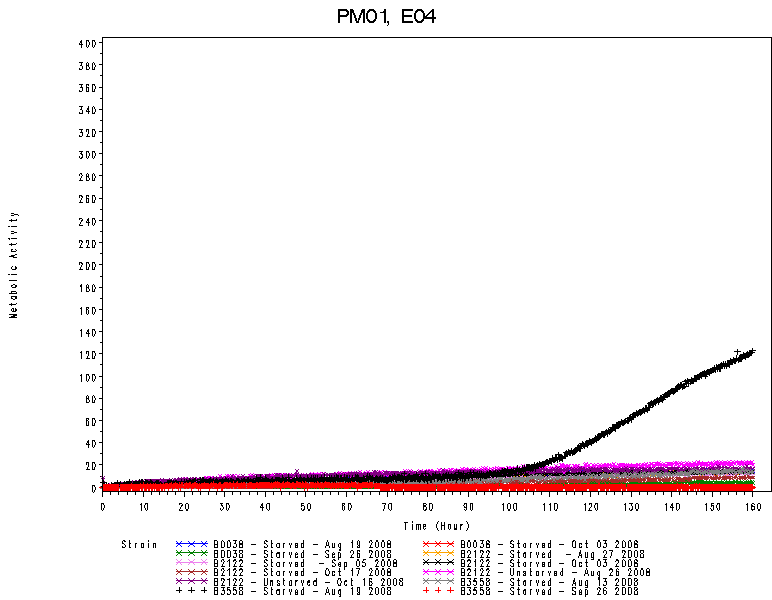

Supplement: Figure S3 — Kinetic curves for all PM plates with Mycobacterium bovis Type 9 strains. (ZIP) [file pone.0052673.s003.zip › suppl fig 3G type 9/Plate01/pm01e04.gif]

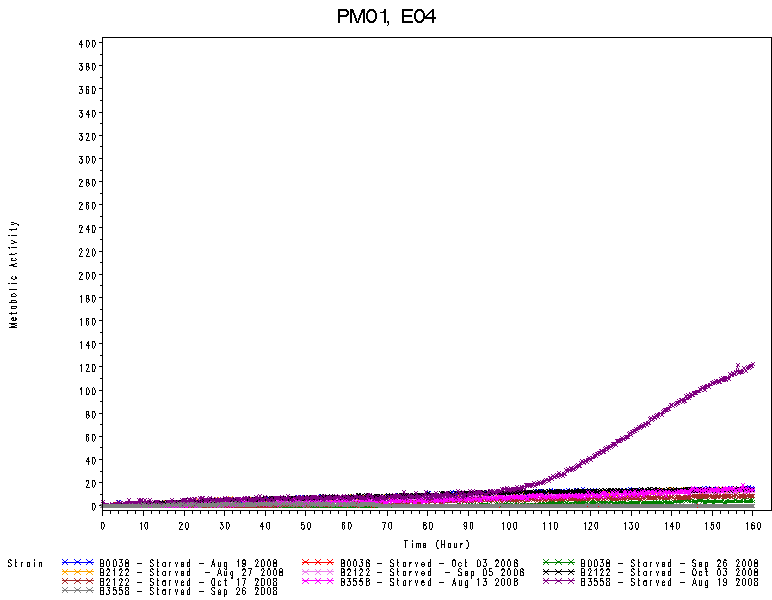

Supplement: Figure S3 — Kinetic curves for all PM plates with Mycobacterium bovis Type 9 strains. (ZIP) [file pone.0052673.s003.zip › suppl fig 3G type 9/Plate01/pm01e041.gif]

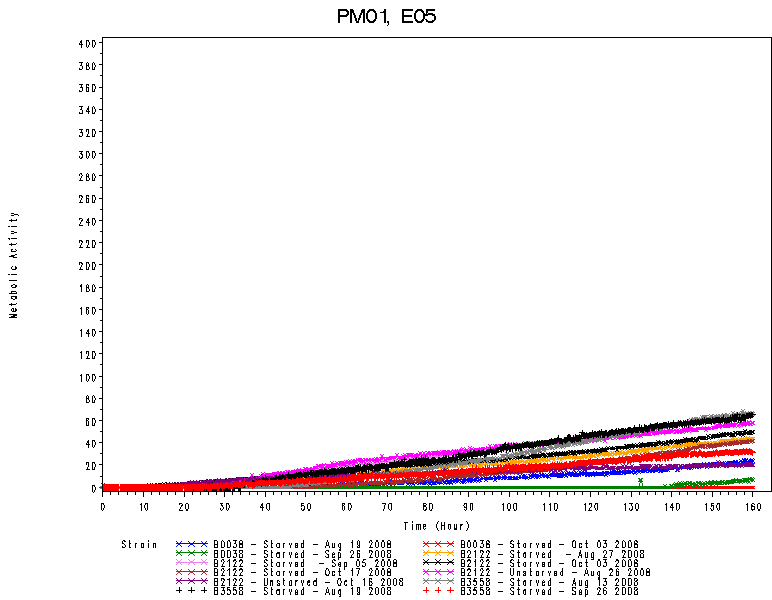

Supplement: Figure S3 — Kinetic curves for all PM plates with Mycobacterium bovis Type 9 strains. (ZIP) [file pone.0052673.s003.zip › suppl fig 3G type 9/Plate01/pm01e05.gif]

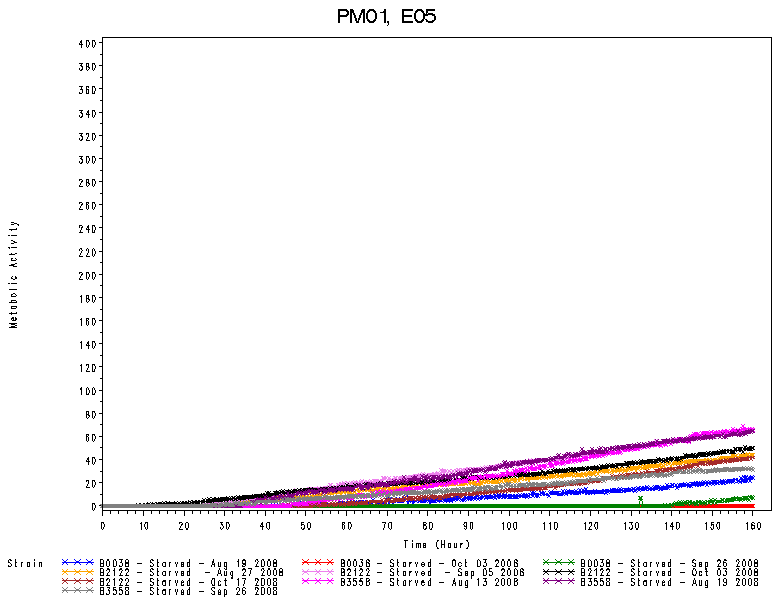

Supplement: Figure S3 — Kinetic curves for all PM plates with Mycobacterium bovis Type 9 strains. (ZIP) [file pone.0052673.s003.zip › suppl fig 3G type 9/Plate01/pm01e051.gif]

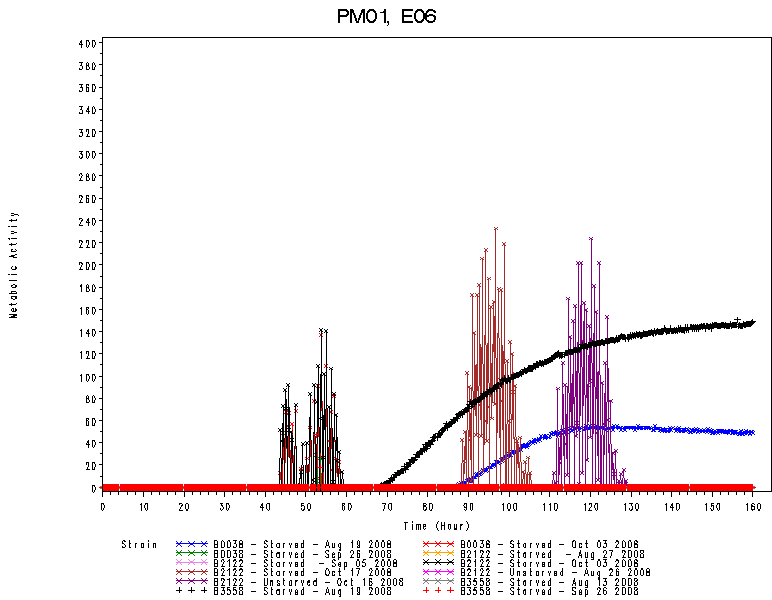

Supplement: Figure S3 — Kinetic curves for all PM plates with Mycobacterium bovis Type 9 strains. (ZIP) [file pone.0052673.s003.zip › suppl fig 3G type 9/Plate01/pm01e06.gif]

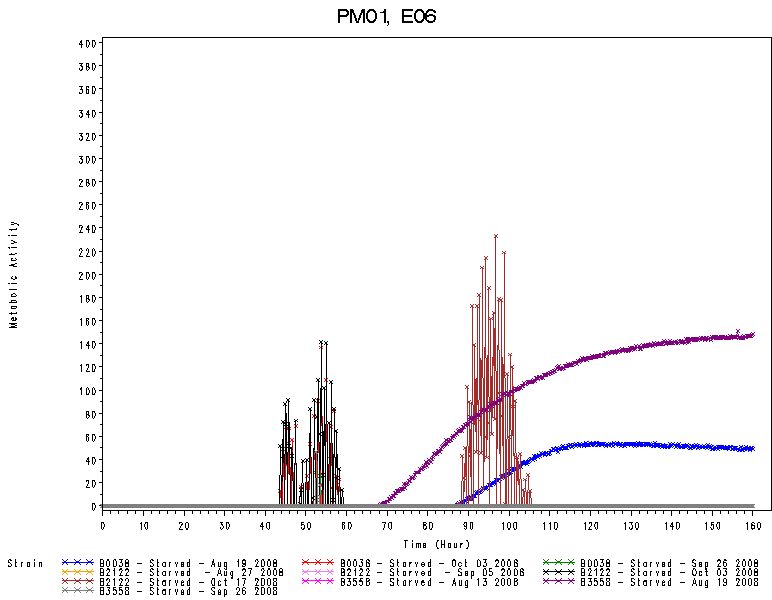

Supplement: Figure S3 — Kinetic curves for all PM plates with Mycobacterium bovis Type 9 strains. (ZIP) [file pone.0052673.s003.zip › suppl fig 3G type 9/Plate01/pm01e061.gif]

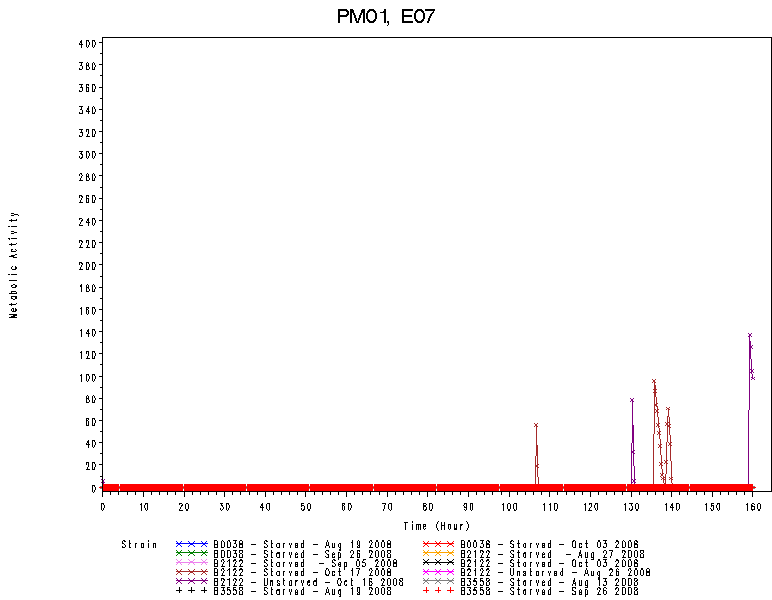

Supplement: Figure S3 — Kinetic curves for all PM plates with Mycobacterium bovis Type 9 strains. (ZIP) [file pone.0052673.s003.zip › suppl fig 3G type 9/Plate01/pm01e07.gif]

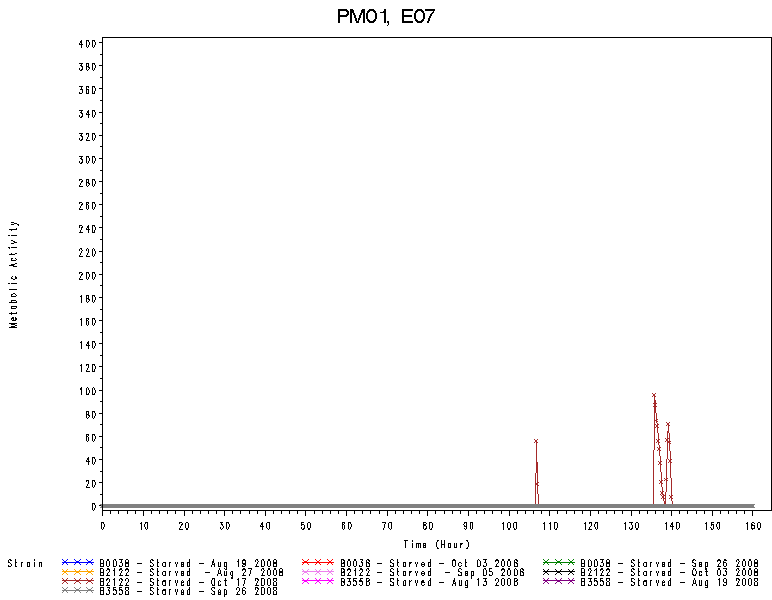

Supplement: Figure S3 — Kinetic curves for all PM plates with Mycobacterium bovis Type 9 strains. (ZIP) [file pone.0052673.s003.zip › suppl fig 3G type 9/Plate01/pm01e071.gif]

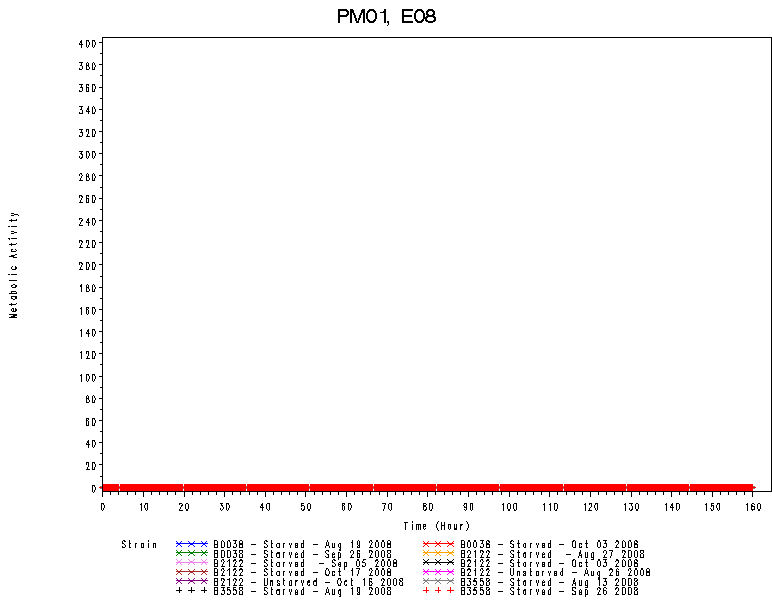

Supplement: Figure S3 — Kinetic curves for all PM plates with Mycobacterium bovis Type 9 strains. (ZIP) [file pone.0052673.s003.zip › suppl fig 3G type 9/Plate01/pm01e08.gif]

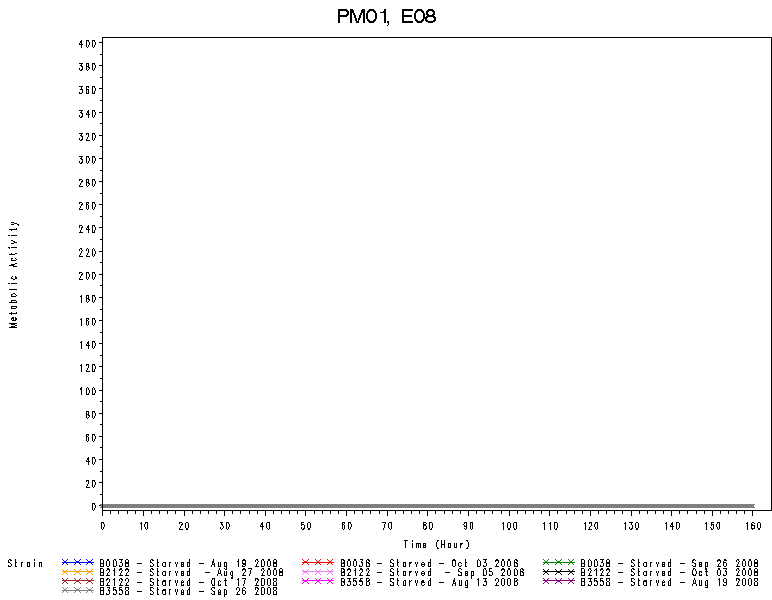

Supplement: Figure S3 — Kinetic curves for all PM plates with Mycobacterium bovis Type 9 strains. (ZIP) [file pone.0052673.s003.zip › suppl fig 3G type 9/Plate01/pm01e081.gif]

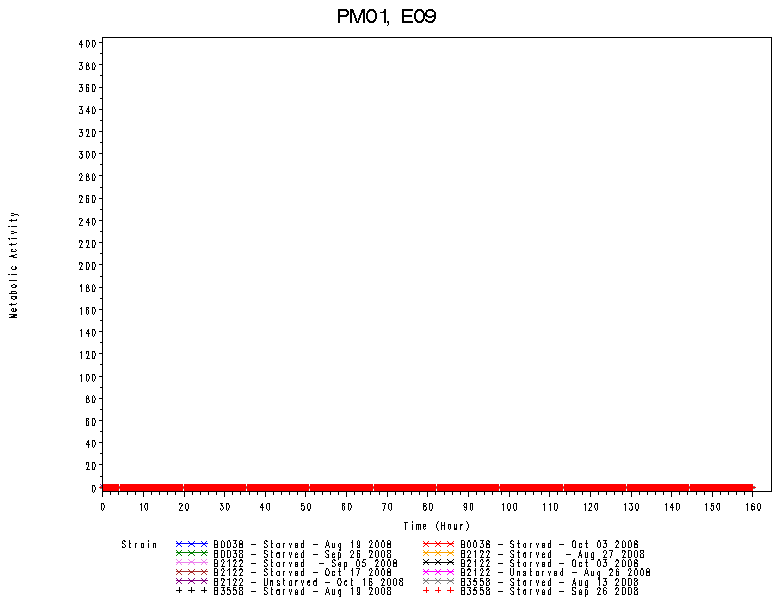

Supplement: Figure S3 — Kinetic curves for all PM plates with Mycobacterium bovis Type 9 strains. (ZIP) [file pone.0052673.s003.zip › suppl fig 3G type 9/Plate01/pm01e09.gif]

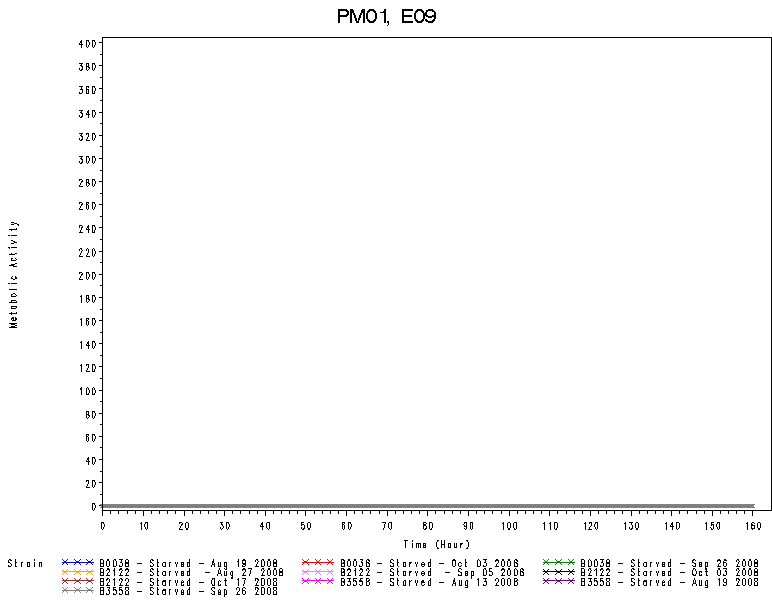

Supplement: Figure S3 — Kinetic curves for all PM plates with Mycobacterium bovis Type 9 strains. (ZIP) [file pone.0052673.s003.zip › suppl fig 3G type 9/Plate01/pm01e091.gif]

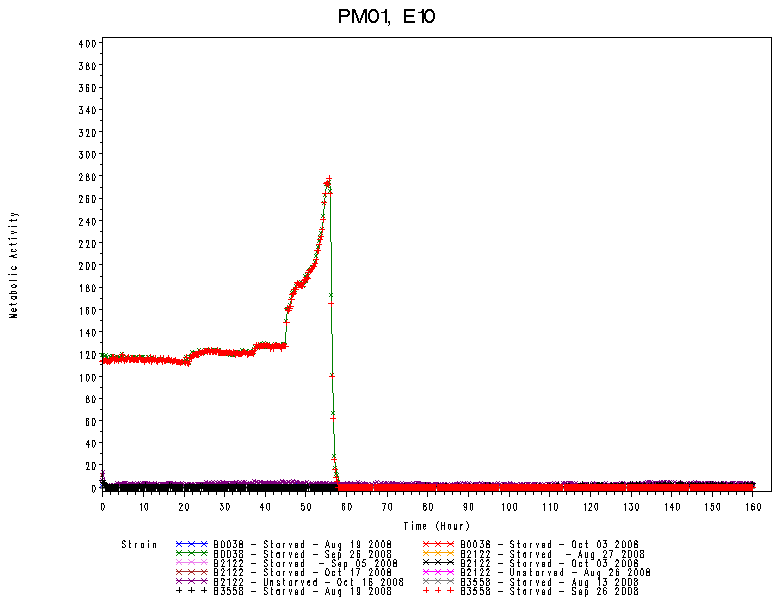

Supplement: Figure S3 — Kinetic curves for all PM plates with Mycobacterium bovis Type 9 strains. (ZIP) [file pone.0052673.s003.zip › suppl fig 3G type 9/Plate01/pm01e10.gif]

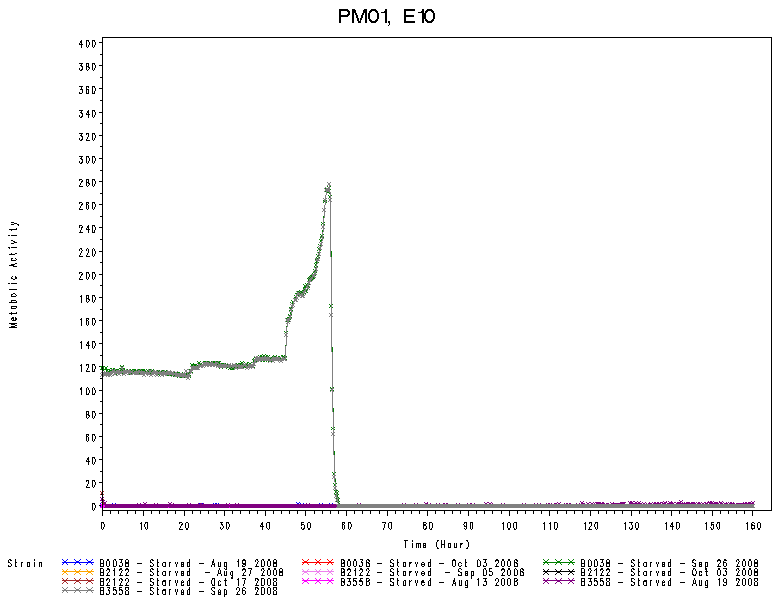

Supplement: Figure S3 — Kinetic curves for all PM plates with Mycobacterium bovis Type 9 strains. (ZIP) [file pone.0052673.s003.zip › suppl fig 3G type 9/Plate01/pm01e101.gif]

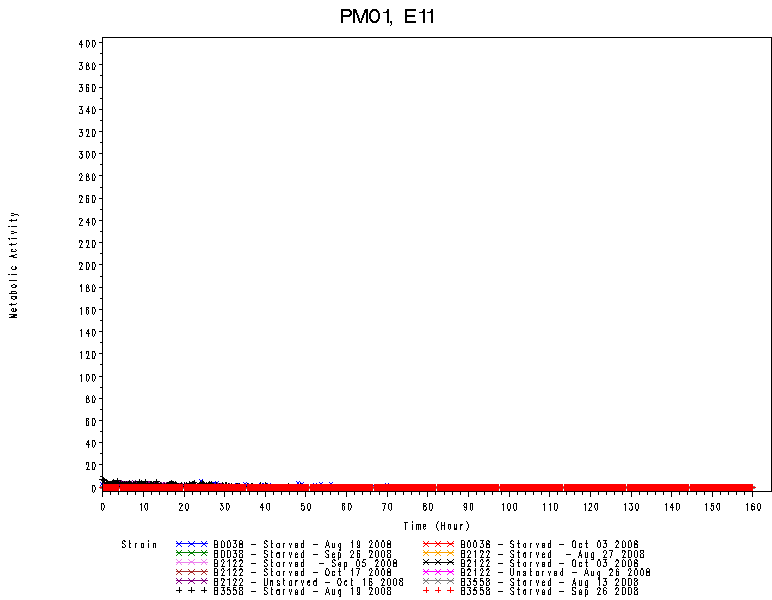

Supplement: Figure S3 — Kinetic curves for all PM plates with Mycobacterium bovis Type 9 strains. (ZIP) [file pone.0052673.s003.zip › suppl fig 3G type 9/Plate01/pm01e11.gif]

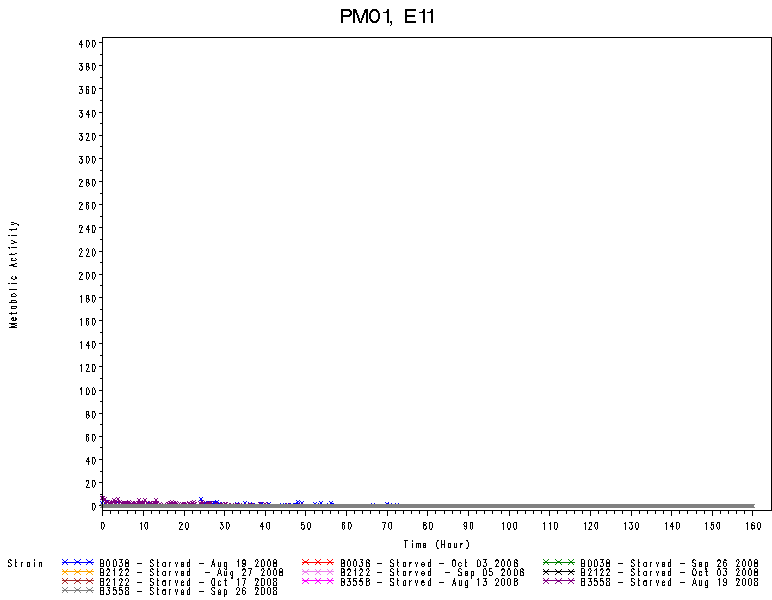

Supplement: Figure S3 — Kinetic curves for all PM plates with Mycobacterium bovis Type 9 strains. (ZIP) [file pone.0052673.s003.zip › suppl fig 3G type 9/Plate01/pm01e111.gif]

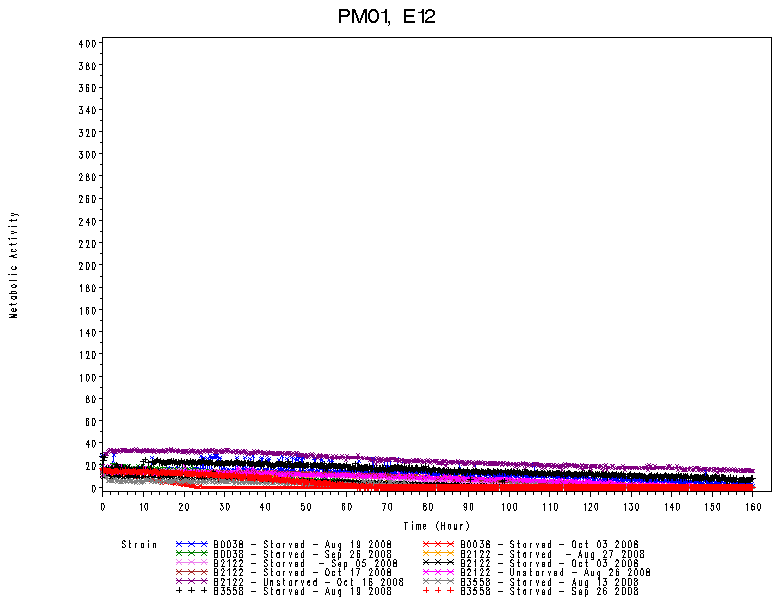

Supplement: Figure S3 — Kinetic curves for all PM plates with Mycobacterium bovis Type 9 strains. (ZIP) [file pone.0052673.s003.zip › suppl fig 3G type 9/Plate01/pm01e12.gif]

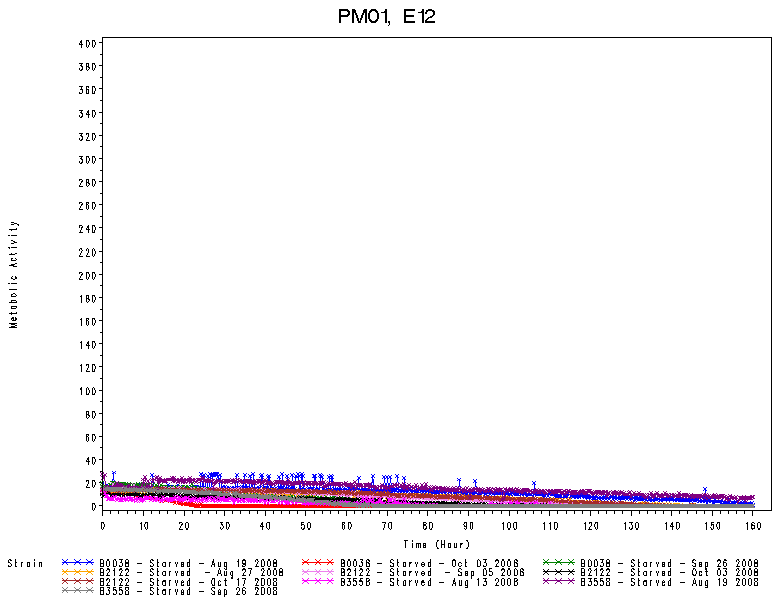

Supplement: Figure S3 — Kinetic curves for all PM plates with Mycobacterium bovis Type 9 strains. (ZIP) [file pone.0052673.s003.zip › suppl fig 3G type 9/Plate01/pm01e121.gif]

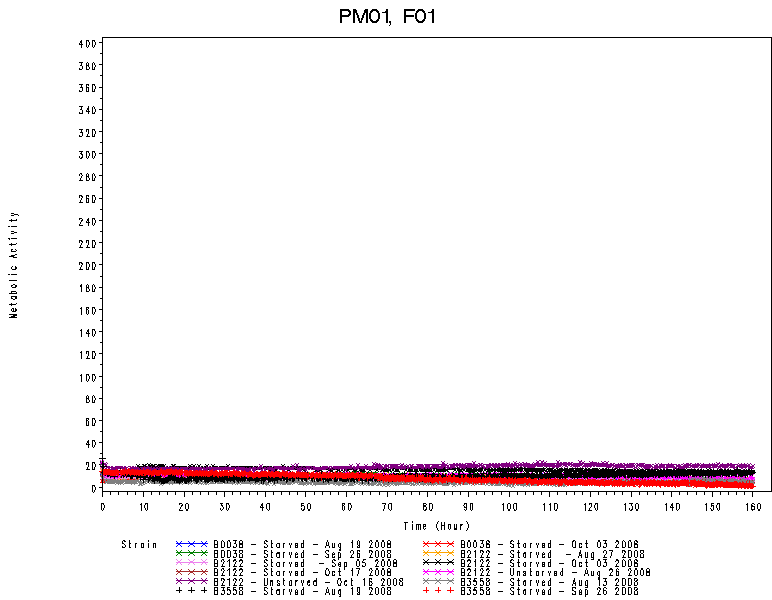

Supplement: Figure S3 — Kinetic curves for all PM plates with Mycobacterium bovis Type 9 strains. (ZIP) [file pone.0052673.s003.zip › suppl fig 3G type 9/Plate01/pm01f01.gif]

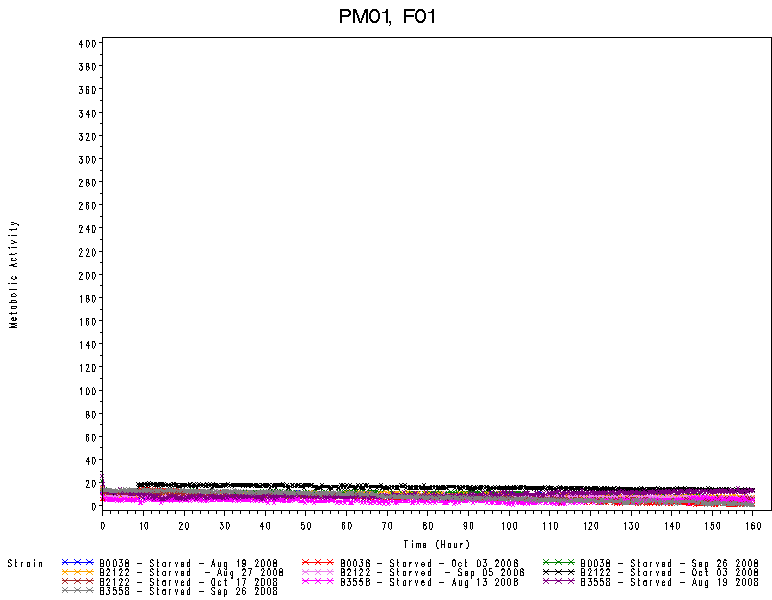

Supplement: Figure S3 — Kinetic curves for all PM plates with Mycobacterium bovis Type 9 strains. (ZIP) [file pone.0052673.s003.zip › suppl fig 3G type 9/Plate01/pm01f011.gif]

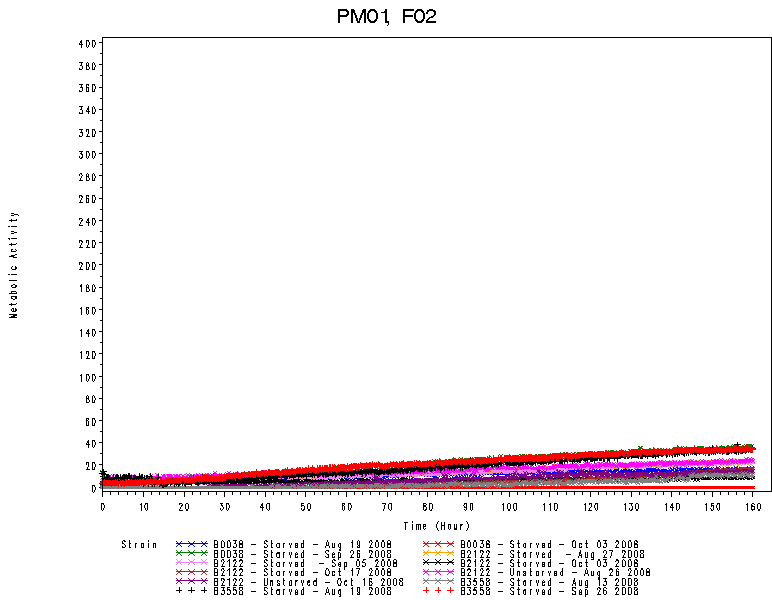

Supplement: Figure S3 — Kinetic curves for all PM plates with Mycobacterium bovis Type 9 strains. (ZIP) [file pone.0052673.s003.zip › suppl fig 3G type 9/Plate01/pm01f02.gif]

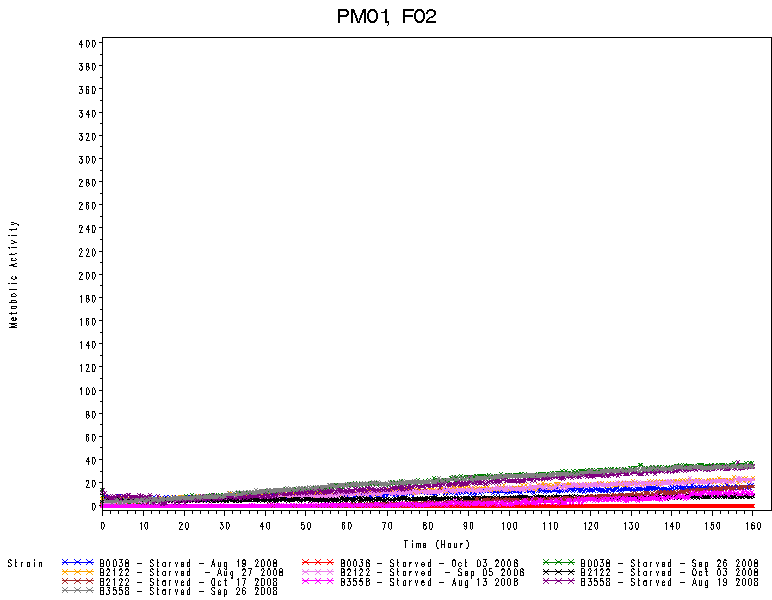

Supplement: Figure S3 — Kinetic curves for all PM plates with Mycobacterium bovis Type 9 strains. (ZIP) [file pone.0052673.s003.zip › suppl fig 3G type 9/Plate01/pm01f021.gif]

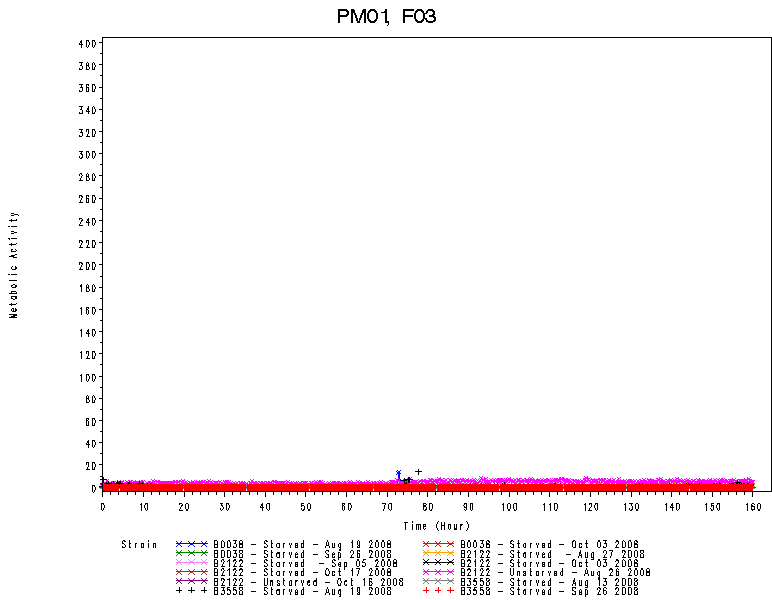

Supplement: Figure S3 — Kinetic curves for all PM plates with Mycobacterium bovis Type 9 strains. (ZIP) [file pone.0052673.s003.zip › suppl fig 3G type 9/Plate01/pm01f03.gif]

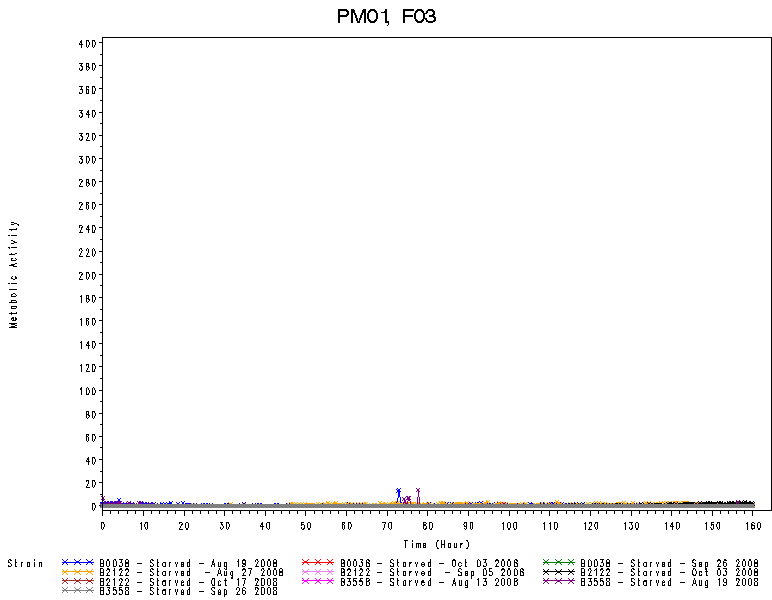

Supplement: Figure S3 — Kinetic curves for all PM plates with Mycobacterium bovis Type 9 strains. (ZIP) [file pone.0052673.s003.zip › suppl fig 3G type 9/Plate01/pm01f031.gif]

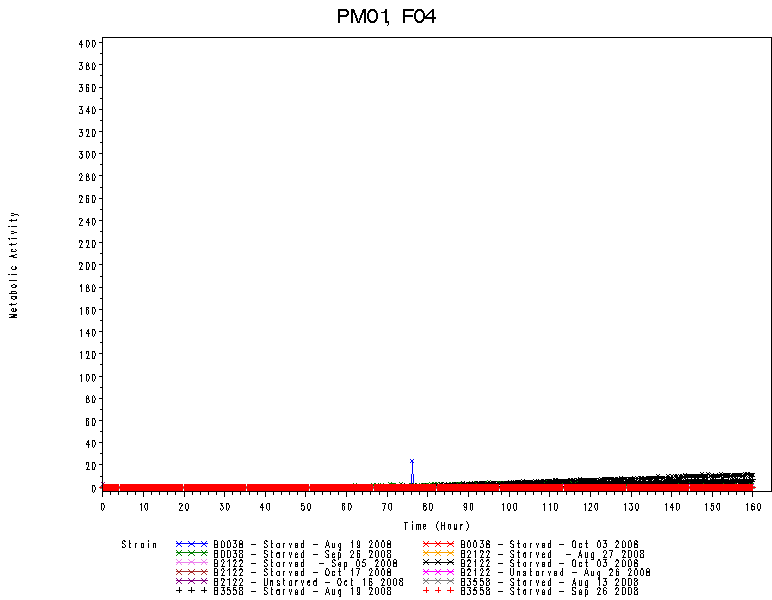

Supplement: Figure S3 — Kinetic curves for all PM plates with Mycobacterium bovis Type 9 strains. (ZIP) [file pone.0052673.s003.zip › suppl fig 3G type 9/Plate01/pm01f04.gif]

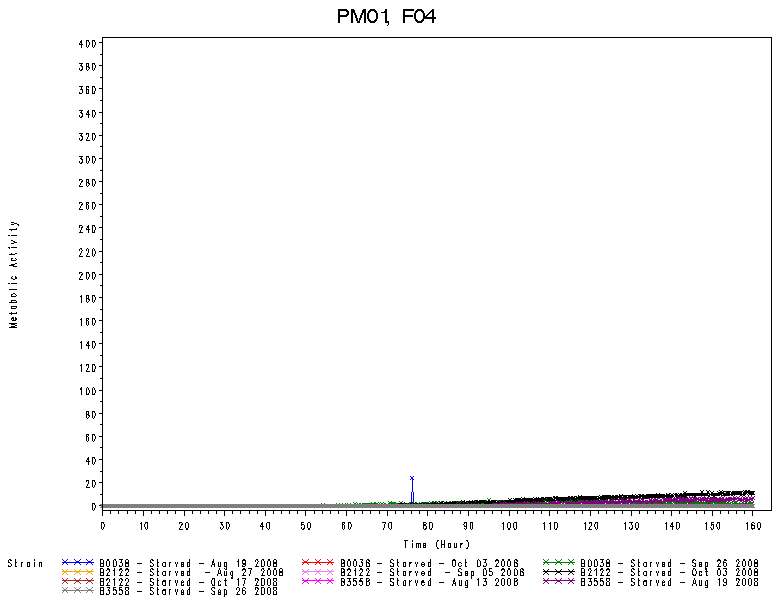

Supplement: Figure S3 — Kinetic curves for all PM plates with Mycobacterium bovis Type 9 strains. (ZIP) [file pone.0052673.s003.zip › suppl fig 3G type 9/Plate01/pm01f041.gif]

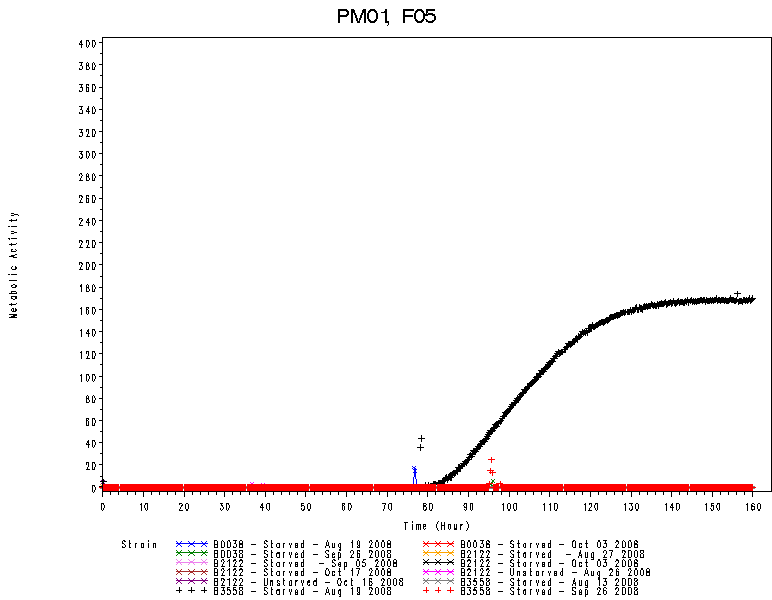

Supplement: Figure S3 — Kinetic curves for all PM plates with Mycobacterium bovis Type 9 strains. (ZIP) [file pone.0052673.s003.zip › suppl fig 3G type 9/Plate01/pm01f05.gif]

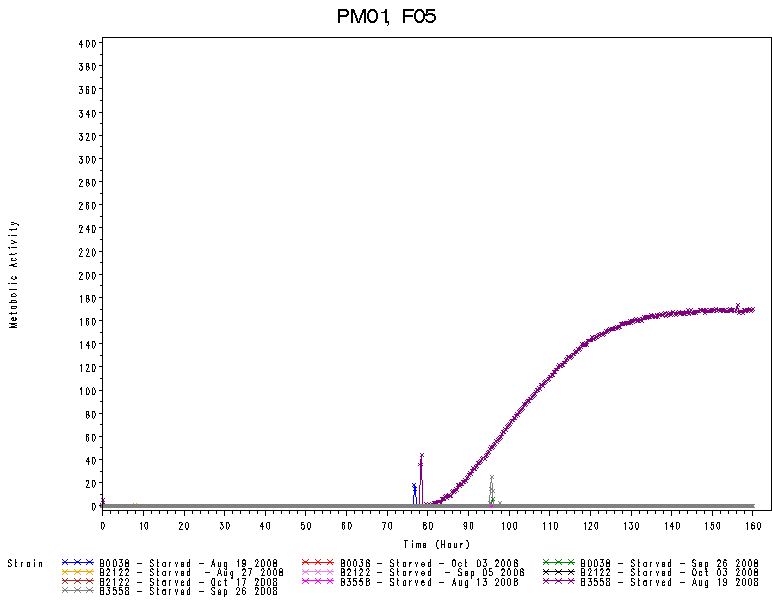

Supplement: Figure S3 — Kinetic curves for all PM plates with Mycobacterium bovis Type 9 strains. (ZIP) [file pone.0052673.s003.zip › suppl fig 3G type 9/Plate01/pm01f051.gif]

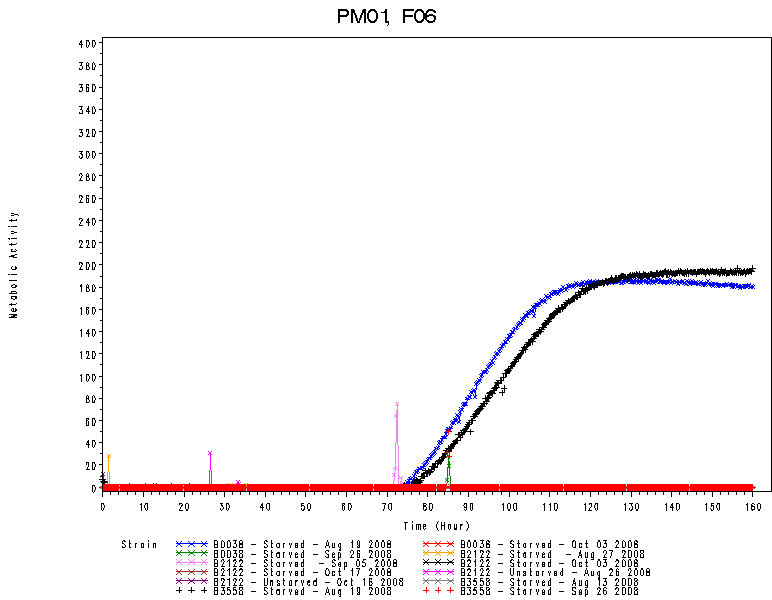

Supplement: Figure S3 — Kinetic curves for all PM plates with Mycobacterium bovis Type 9 strains. (ZIP) [file pone.0052673.s003.zip › suppl fig 3G type 9/Plate01/pm01f06.gif]

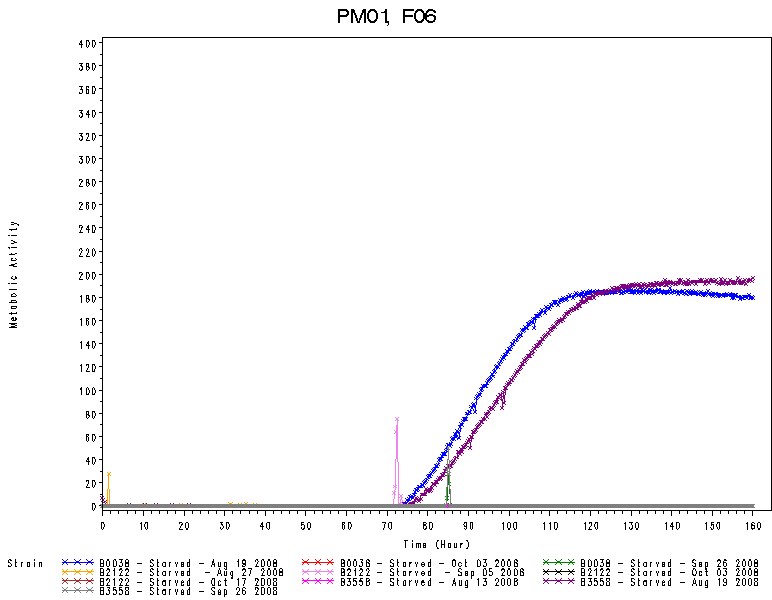

Supplement: Figure S3 — Kinetic curves for all PM plates with Mycobacterium bovis Type 9 strains. (ZIP) [file pone.0052673.s003.zip › suppl fig 3G type 9/Plate01/pm01f061.gif]

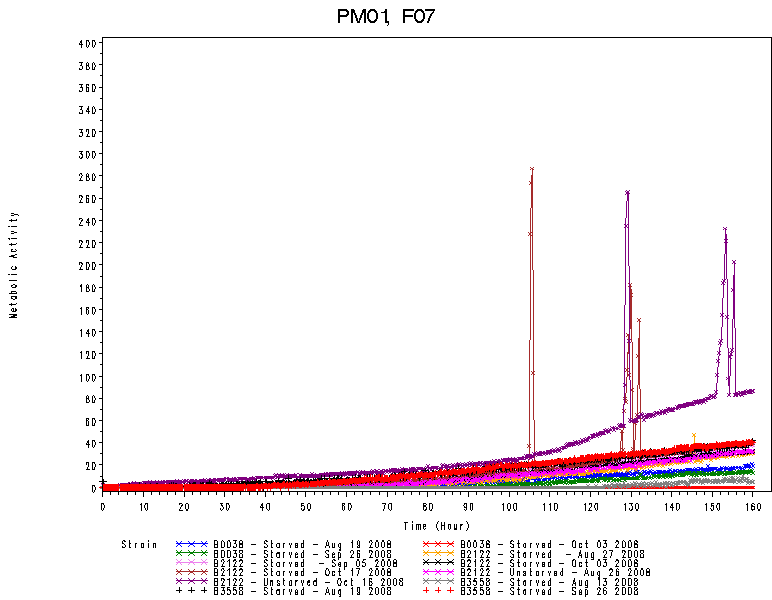

Supplement: Figure S3 — Kinetic curves for all PM plates with Mycobacterium bovis Type 9 strains. (ZIP) [file pone.0052673.s003.zip › suppl fig 3G type 9/Plate01/pm01f07.gif]

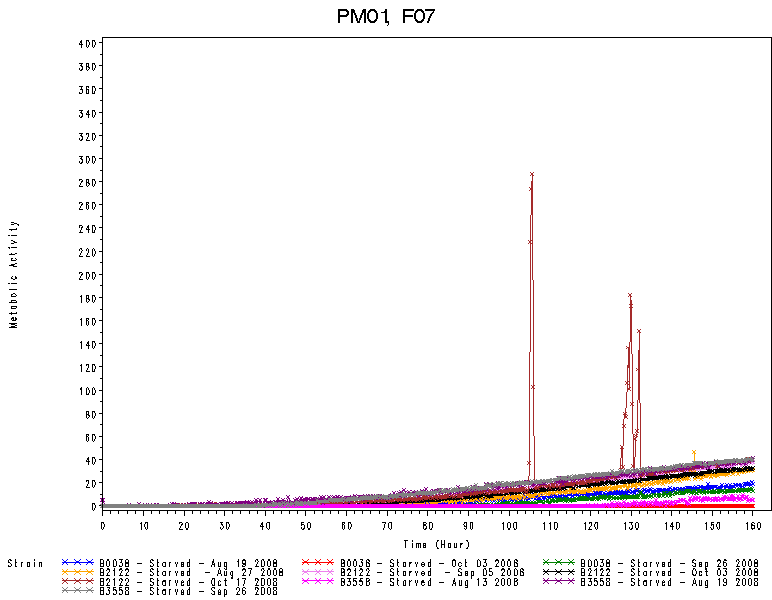

Supplement: Figure S3 — Kinetic curves for all PM plates with Mycobacterium bovis Type 9 strains. (ZIP) [file pone.0052673.s003.zip › suppl fig 3G type 9/Plate01/pm01f071.gif]

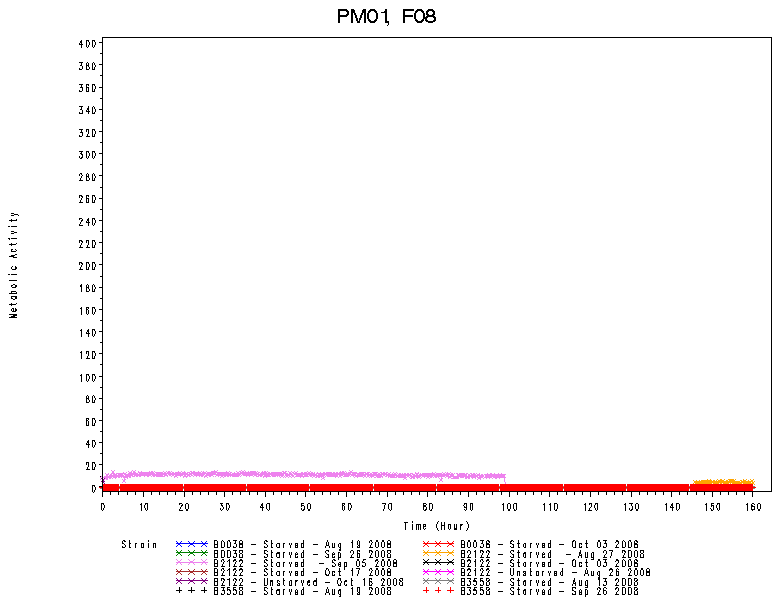

Supplement: Figure S3 — Kinetic curves for all PM plates with Mycobacterium bovis Type 9 strains. (ZIP) [file pone.0052673.s003.zip › suppl fig 3G type 9/Plate01/pm01f08.gif]

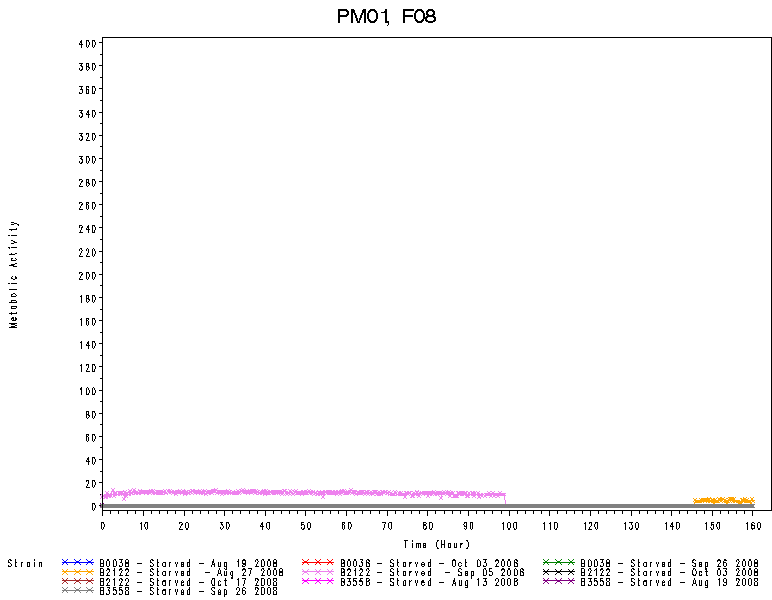

Supplement: Figure S3 — Kinetic curves for all PM plates with Mycobacterium bovis Type 9 strains. (ZIP) [file pone.0052673.s003.zip › suppl fig 3G type 9/Plate01/pm01f081.gif]

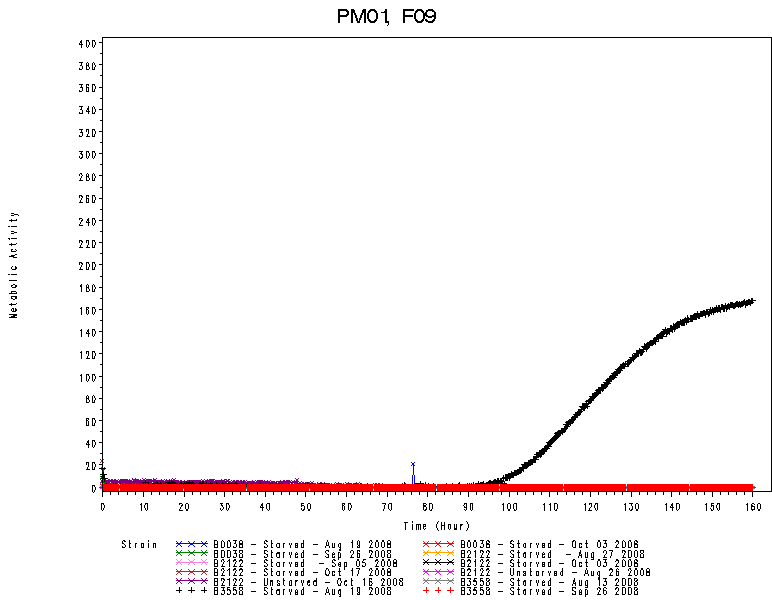

Supplement: Figure S3 — Kinetic curves for all PM plates with Mycobacterium bovis Type 9 strains. (ZIP) [file pone.0052673.s003.zip › suppl fig 3G type 9/Plate01/pm01f09.gif]

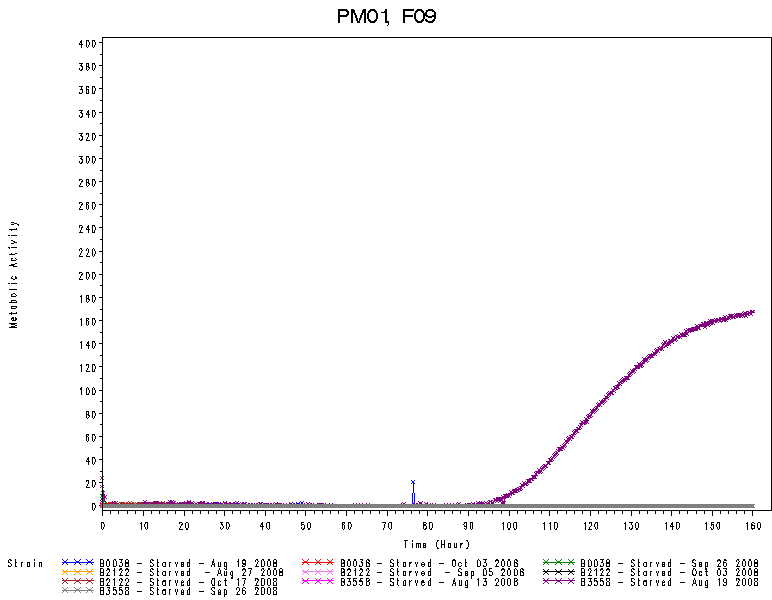

Supplement: Figure S3 — Kinetic curves for all PM plates with Mycobacterium bovis Type 9 strains. (ZIP) [file pone.0052673.s003.zip › suppl fig 3G type 9/Plate01/pm01f091.gif]

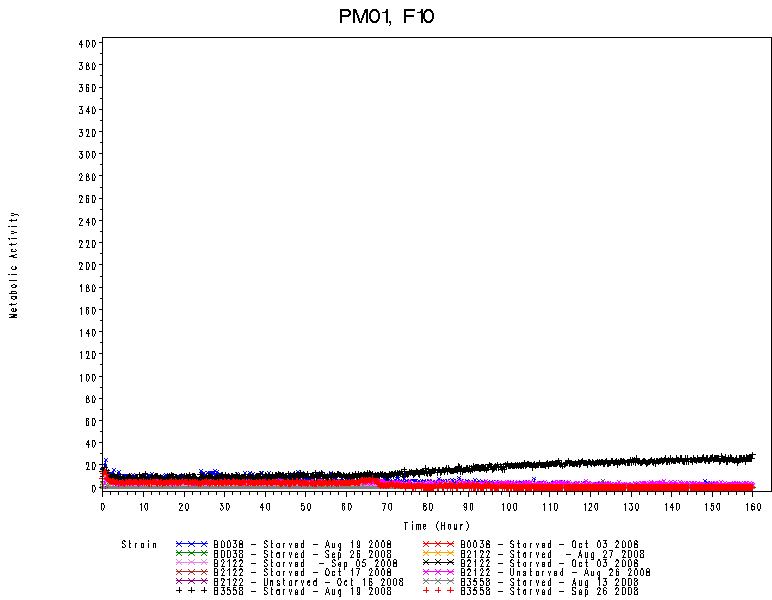

Supplement: Figure S3 — Kinetic curves for all PM plates with Mycobacterium bovis Type 9 strains. (ZIP) [file pone.0052673.s003.zip › suppl fig 3G type 9/Plate01/pm01f10.gif]

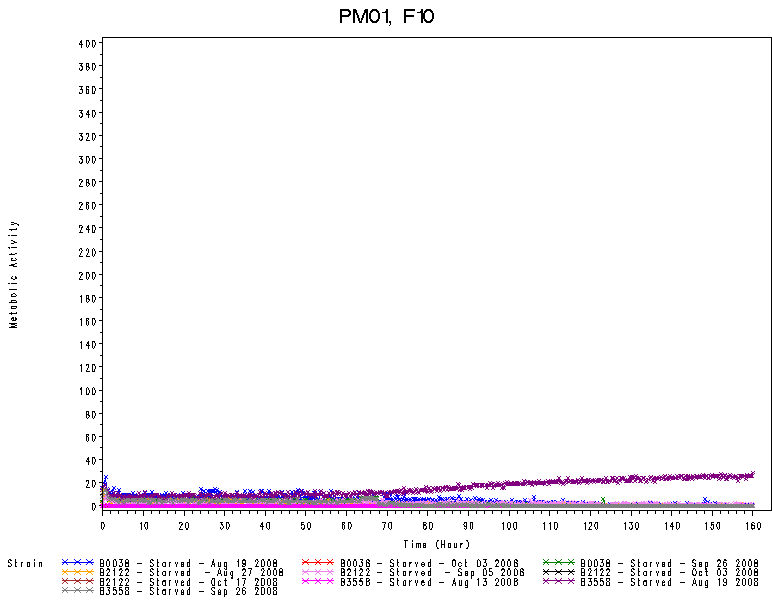

Supplement: Figure S3 — Kinetic curves for all PM plates with Mycobacterium bovis Type 9 strains. (ZIP) [file pone.0052673.s003.zip › suppl fig 3G type 9/Plate01/pm01f101.gif]

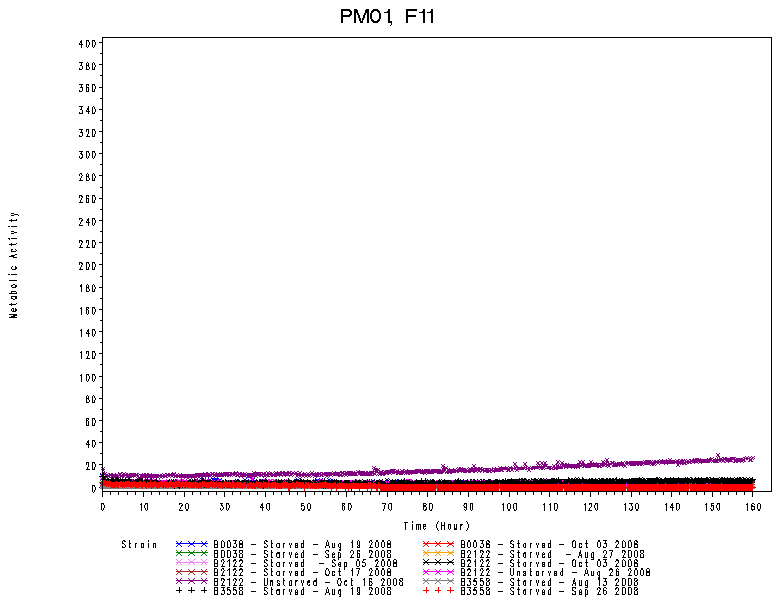

Supplement: Figure S3 — Kinetic curves for all PM plates with Mycobacterium bovis Type 9 strains. (ZIP) [file pone.0052673.s003.zip › suppl fig 3G type 9/Plate01/pm01f11.gif]

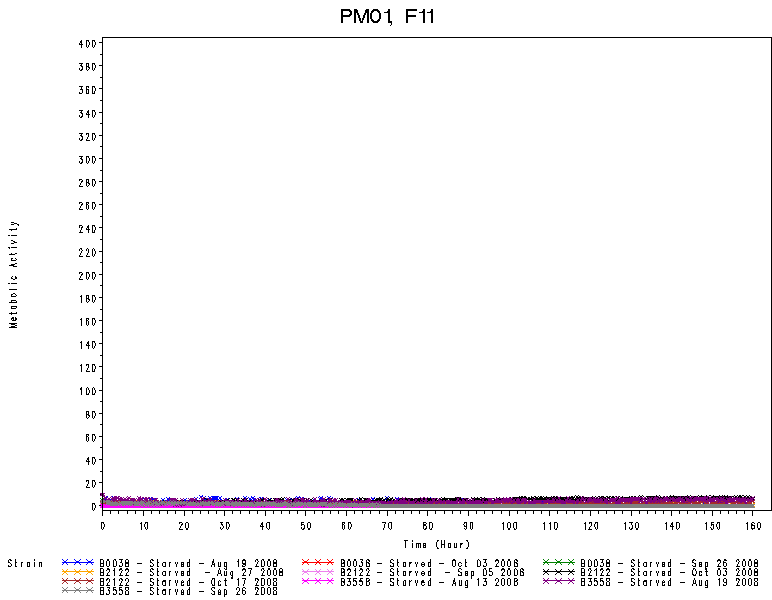

Supplement: Figure S3 — Kinetic curves for all PM plates with Mycobacterium bovis Type 9 strains. (ZIP) [file pone.0052673.s003.zip › suppl fig 3G type 9/Plate01/pm01f111.gif]

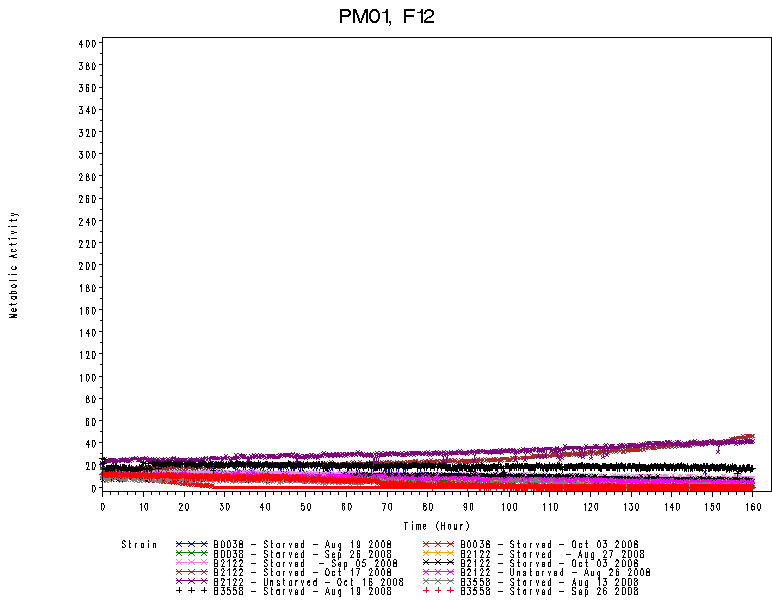

Supplement: Figure S3 — Kinetic curves for all PM plates with Mycobacterium bovis Type 9 strains. (ZIP) [file pone.0052673.s003.zip › suppl fig 3G type 9/Plate01/pm01f12.gif]

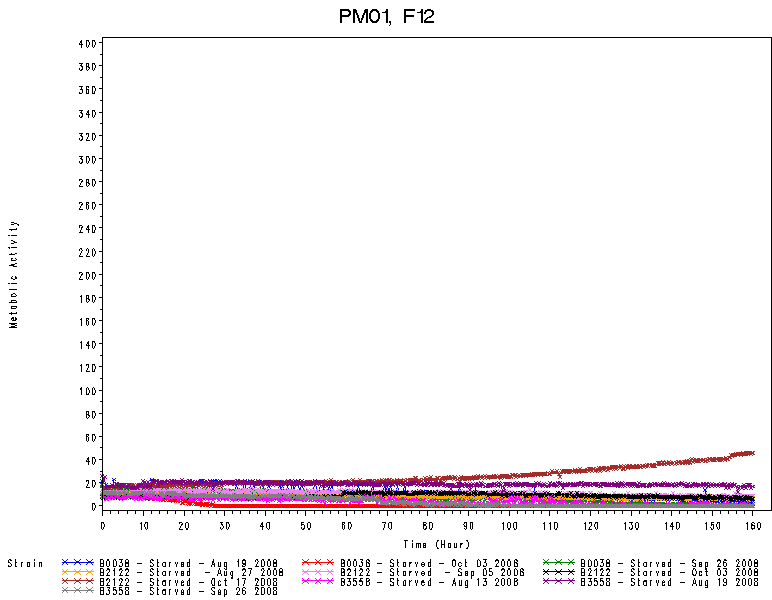

Supplement: Figure S3 — Kinetic curves for all PM plates with Mycobacterium bovis Type 9 strains. (ZIP) [file pone.0052673.s003.zip › suppl fig 3G type 9/Plate01/pm01f121.gif]

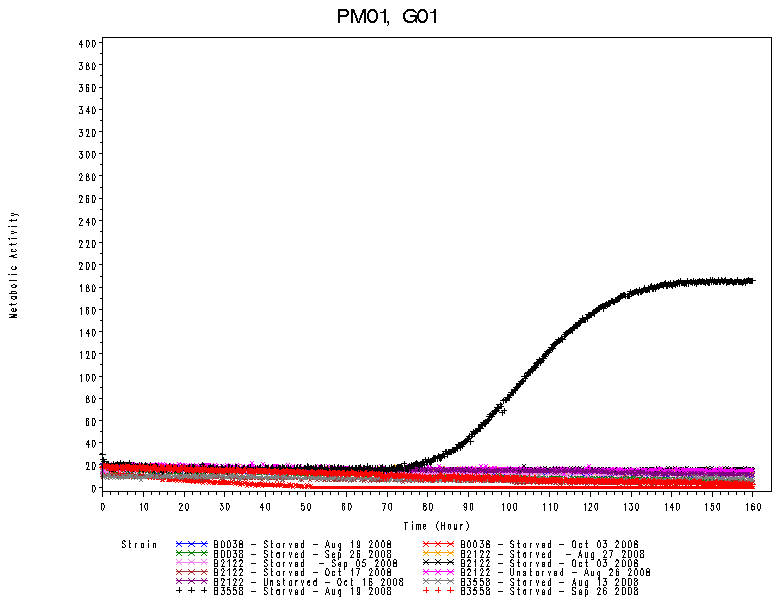

Supplement: Figure S3 — Kinetic curves for all PM plates with Mycobacterium bovis Type 9 strains. (ZIP) [file pone.0052673.s003.zip › suppl fig 3G type 9/Plate01/pm01g01.gif]

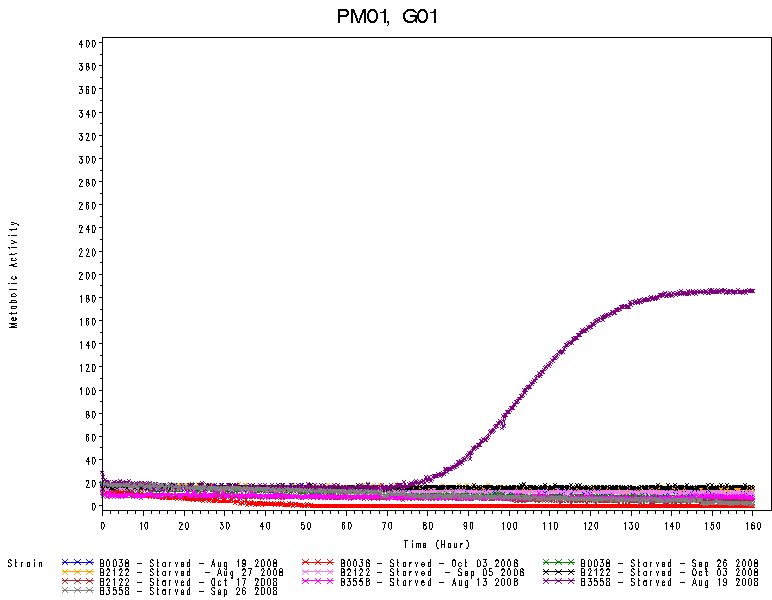

Supplement: Figure S3 — Kinetic curves for all PM plates with Mycobacterium bovis Type 9 strains. (ZIP) [file pone.0052673.s003.zip › suppl fig 3G type 9/Plate01/pm01g011.gif]

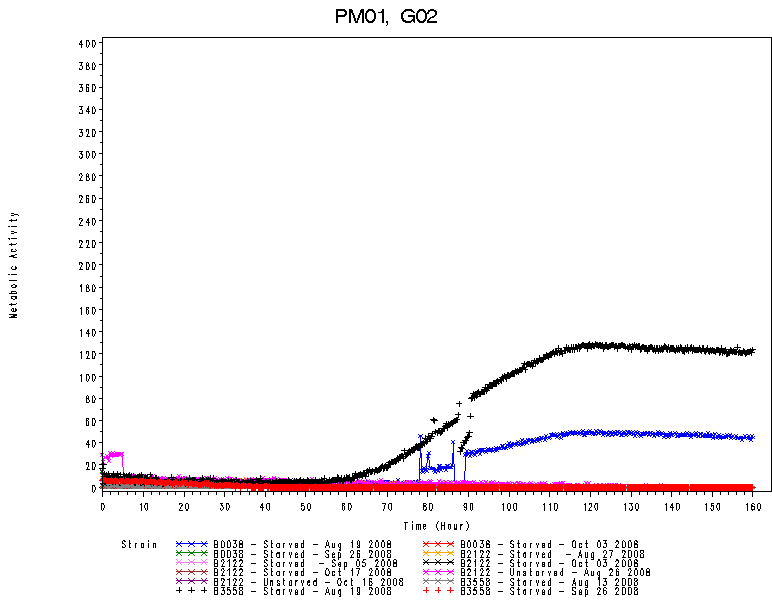

Supplement: Figure S3 — Kinetic curves for all PM plates with Mycobacterium bovis Type 9 strains. (ZIP) [file pone.0052673.s003.zip › suppl fig 3G type 9/Plate01/pm01g02.gif]

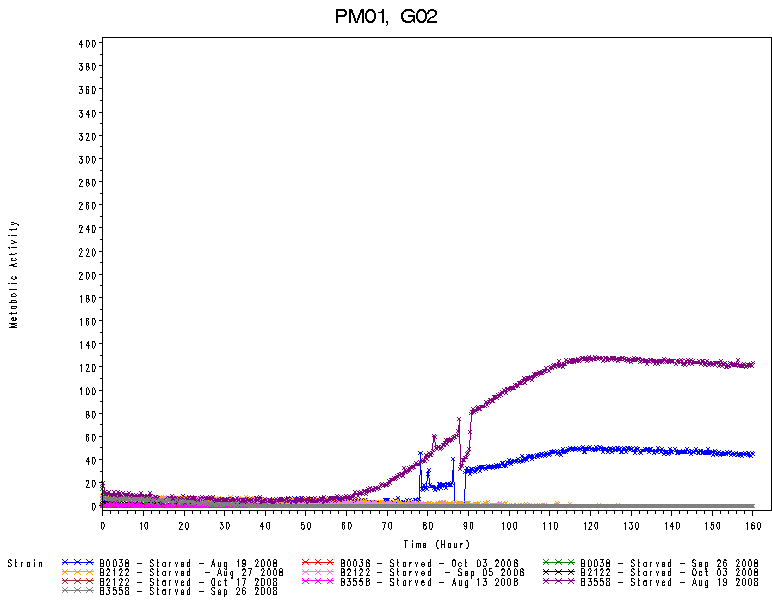

Supplement: Figure S3 — Kinetic curves for all PM plates with Mycobacterium bovis Type 9 strains. (ZIP) [file pone.0052673.s003.zip › suppl fig 3G type 9/Plate01/pm01g021.gif]

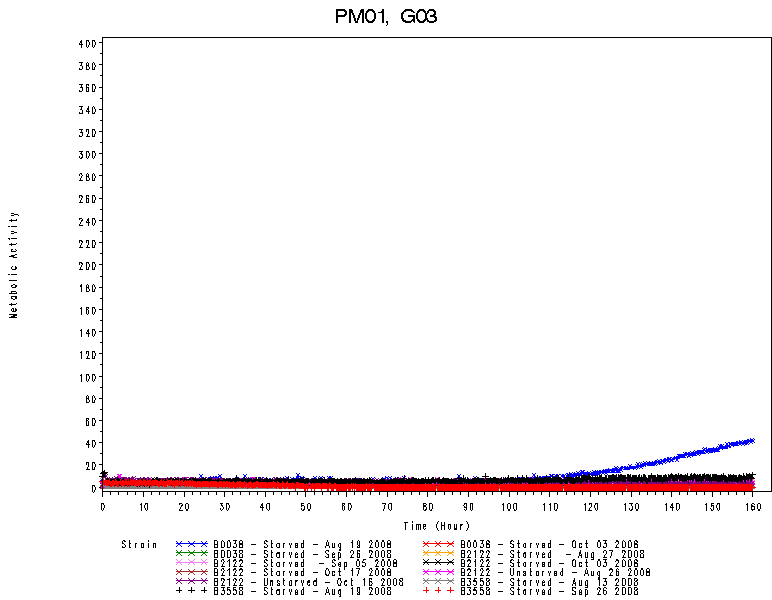

Supplement: Figure S3 — Kinetic curves for all PM plates with Mycobacterium bovis Type 9 strains. (ZIP) [file pone.0052673.s003.zip › suppl fig 3G type 9/Plate01/pm01g03.gif]

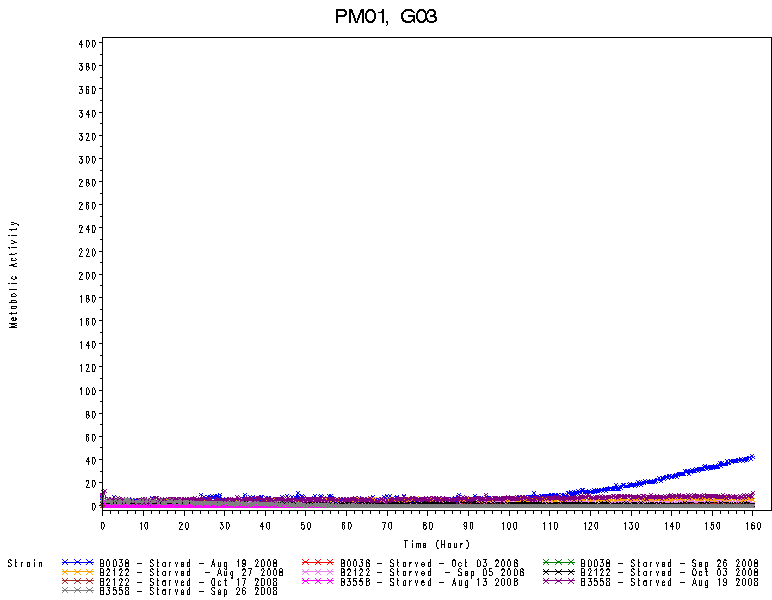

Supplement: Figure S3 — Kinetic curves for all PM plates with Mycobacterium bovis Type 9 strains. (ZIP) [file pone.0052673.s003.zip › suppl fig 3G type 9/Plate01/pm01g031.gif]

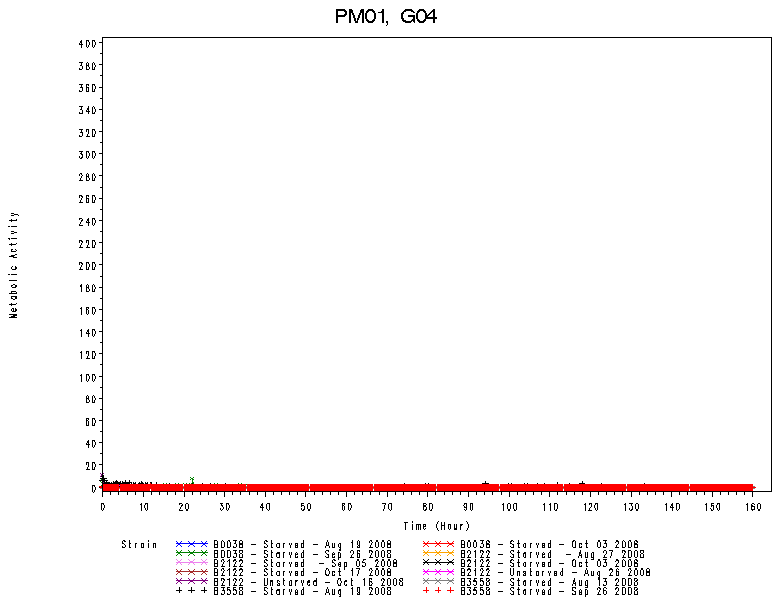

Supplement: Figure S3 — Kinetic curves for all PM plates with Mycobacterium bovis Type 9 strains. (ZIP) [file pone.0052673.s003.zip › suppl fig 3G type 9/Plate01/pm01g04.gif]

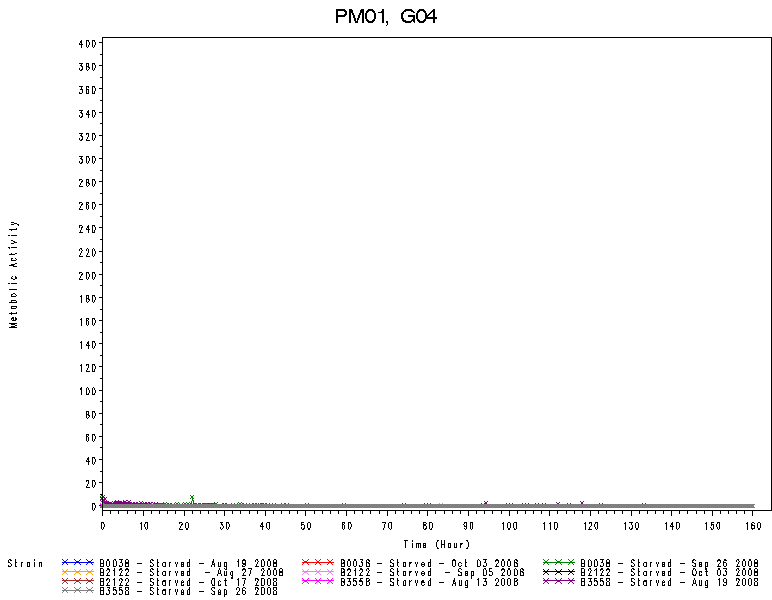

Supplement: Figure S3 — Kinetic curves for all PM plates with Mycobacterium bovis Type 9 strains. (ZIP) [file pone.0052673.s003.zip › suppl fig 3G type 9/Plate01/pm01g041.gif]

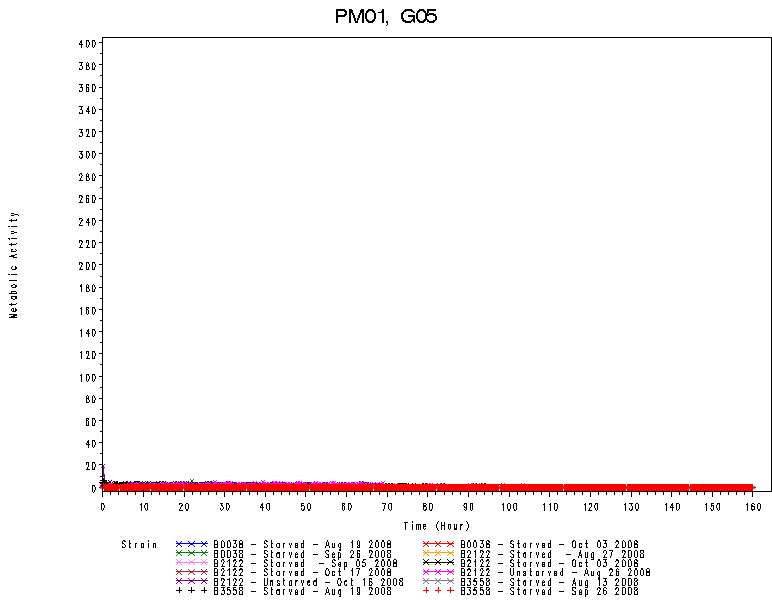

Supplement: Figure S3 — Kinetic curves for all PM plates with Mycobacterium bovis Type 9 strains. (ZIP) [file pone.0052673.s003.zip › suppl fig 3G type 9/Plate01/pm01g05.gif]

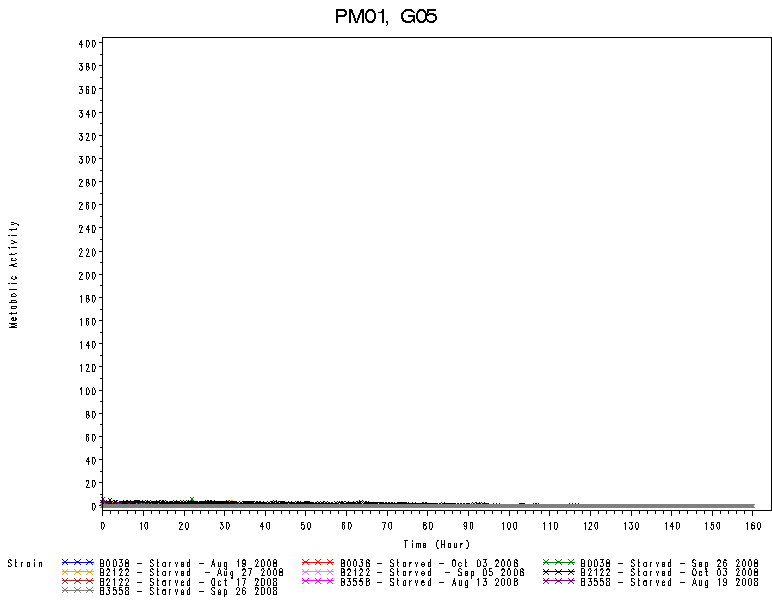

Supplement: Figure S3 — Kinetic curves for all PM plates with Mycobacterium bovis Type 9 strains. (ZIP) [file pone.0052673.s003.zip › suppl fig 3G type 9/Plate01/pm01g051.gif]

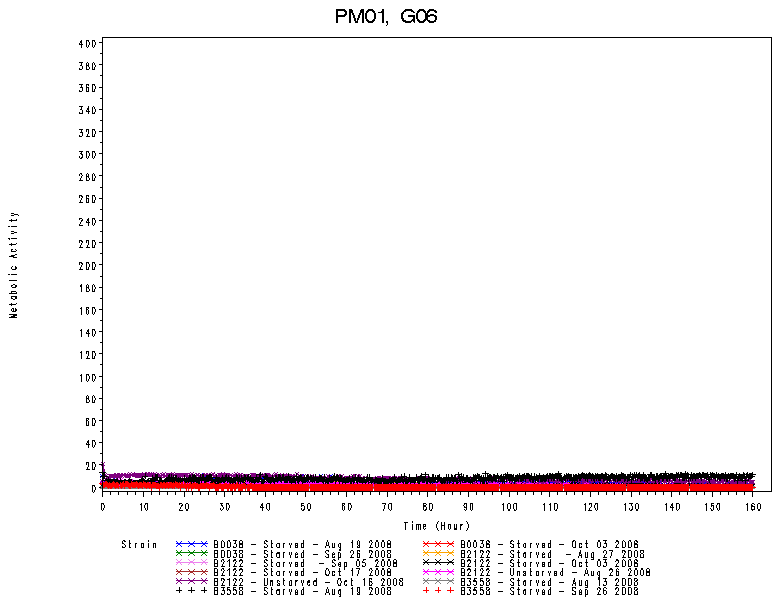

Supplement: Figure S3 — Kinetic curves for all PM plates with Mycobacterium bovis Type 9 strains. (ZIP) [file pone.0052673.s003.zip › suppl fig 3G type 9/Plate01/pm01g06.gif]

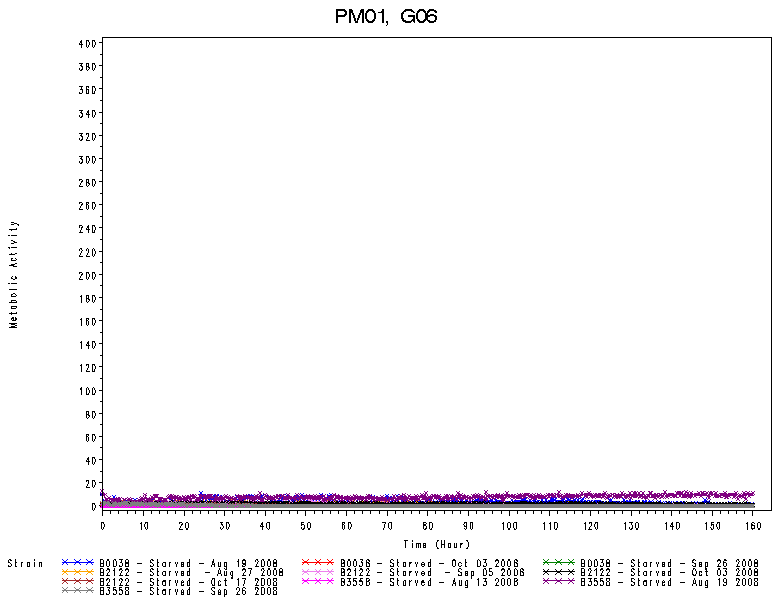

Supplement: Figure S3 — Kinetic curves for all PM plates with Mycobacterium bovis Type 9 strains. (ZIP) [file pone.0052673.s003.zip › suppl fig 3G type 9/Plate01/pm01g061.gif]

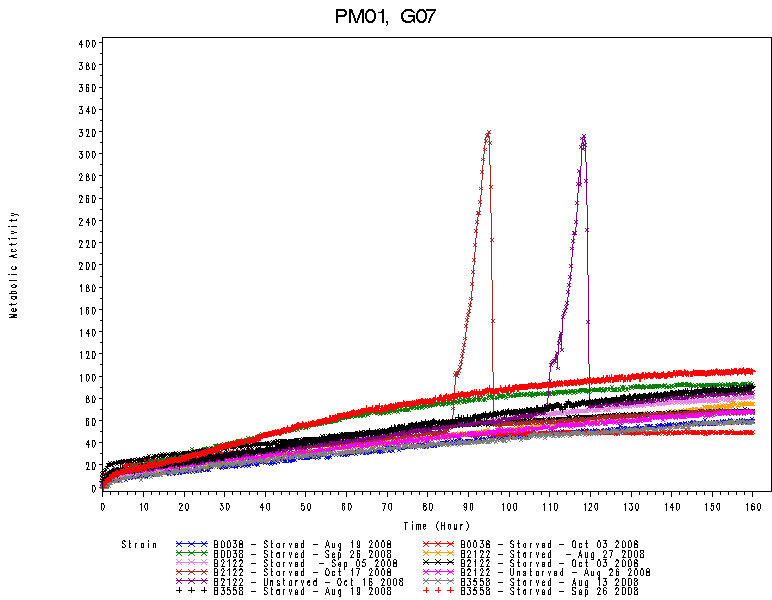

Supplement: Figure S3 — Kinetic curves for all PM plates with Mycobacterium bovis Type 9 strains. (ZIP) [file pone.0052673.s003.zip › suppl fig 3G type 9/Plate01/pm01g07.gif]

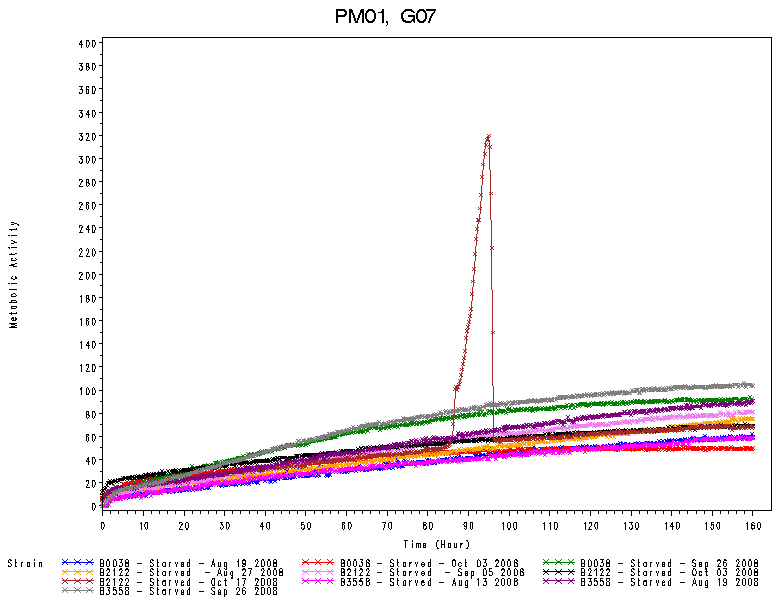

Supplement: Figure S3 — Kinetic curves for all PM plates with Mycobacterium bovis Type 9 strains. (ZIP) [file pone.0052673.s003.zip › suppl fig 3G type 9/Plate01/pm01g071.gif]

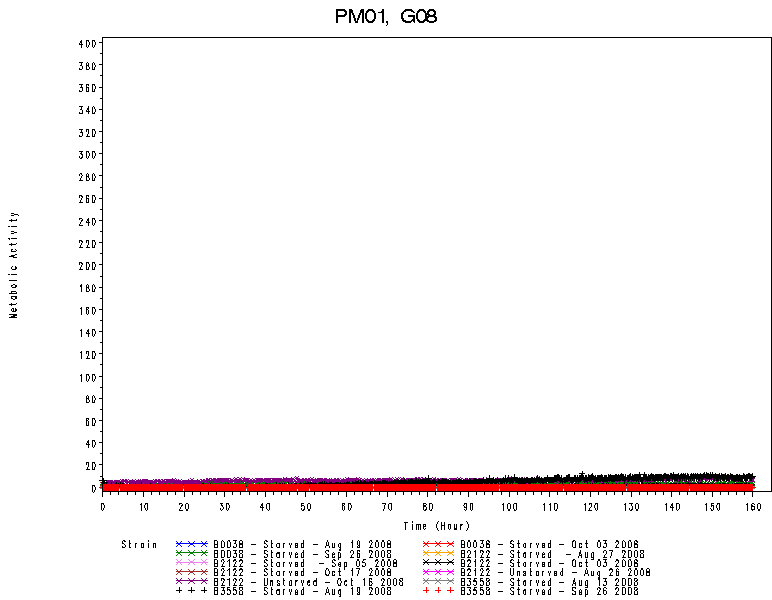

Supplement: Figure S3 — Kinetic curves for all PM plates with Mycobacterium bovis Type 9 strains. (ZIP) [file pone.0052673.s003.zip › suppl fig 3G type 9/Plate01/pm01g08.gif]

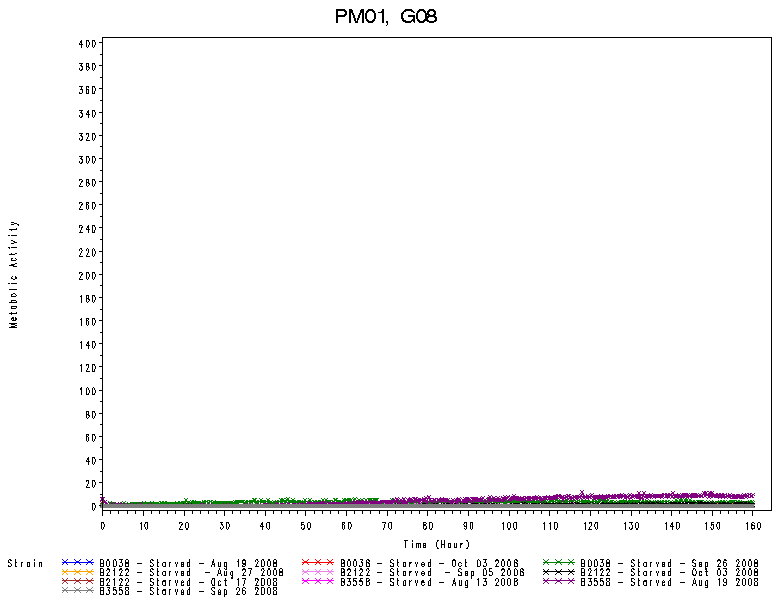

Supplement: Figure S3 — Kinetic curves for all PM plates with Mycobacterium bovis Type 9 strains. (ZIP) [file pone.0052673.s003.zip › suppl fig 3G type 9/Plate01/pm01g081.gif]

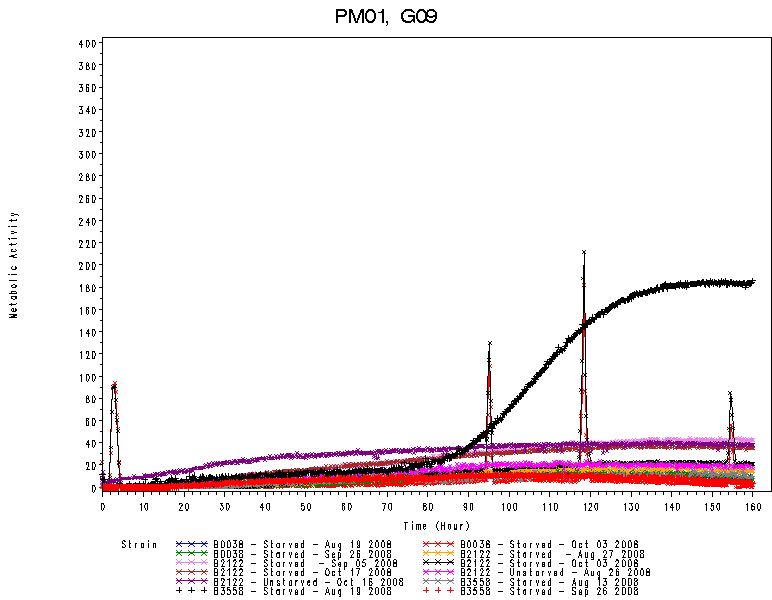

Supplement: Figure S3 — Kinetic curves for all PM plates with Mycobacterium bovis Type 9 strains. (ZIP) [file pone.0052673.s003.zip › suppl fig 3G type 9/Plate01/pm01g09.gif]

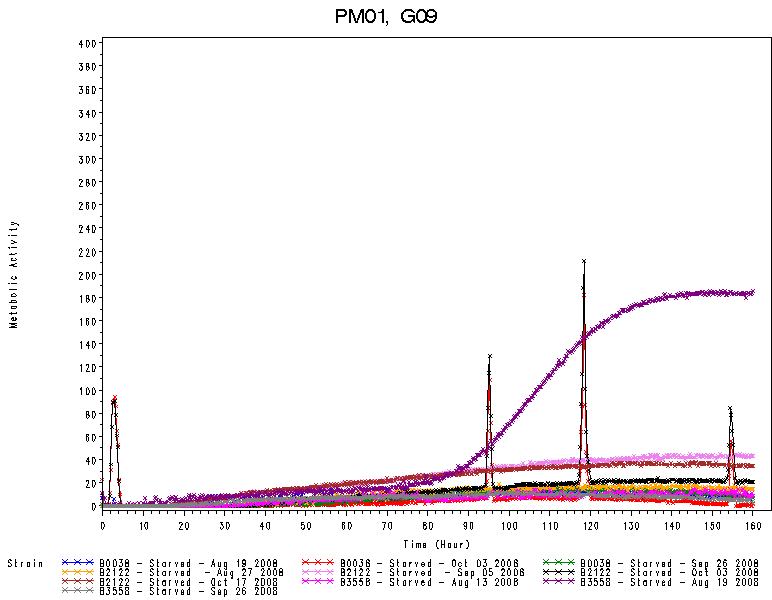

Supplement: Figure S3 — Kinetic curves for all PM plates with Mycobacterium bovis Type 9 strains. (ZIP) [file pone.0052673.s003.zip › suppl fig 3G type 9/Plate01/pm01g091.gif]

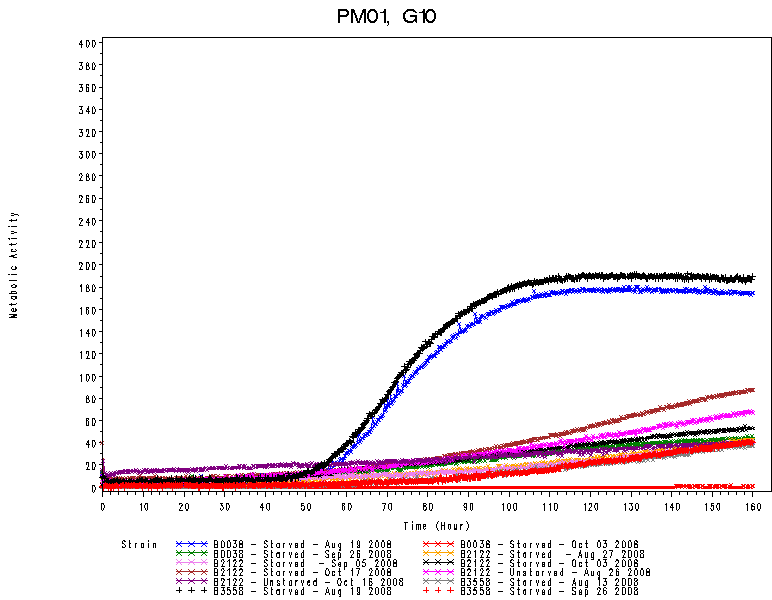

Supplement: Figure S3 — Kinetic curves for all PM plates with Mycobacterium bovis Type 9 strains. (ZIP) [file pone.0052673.s003.zip › suppl fig 3G type 9/Plate01/pm01g10.gif]

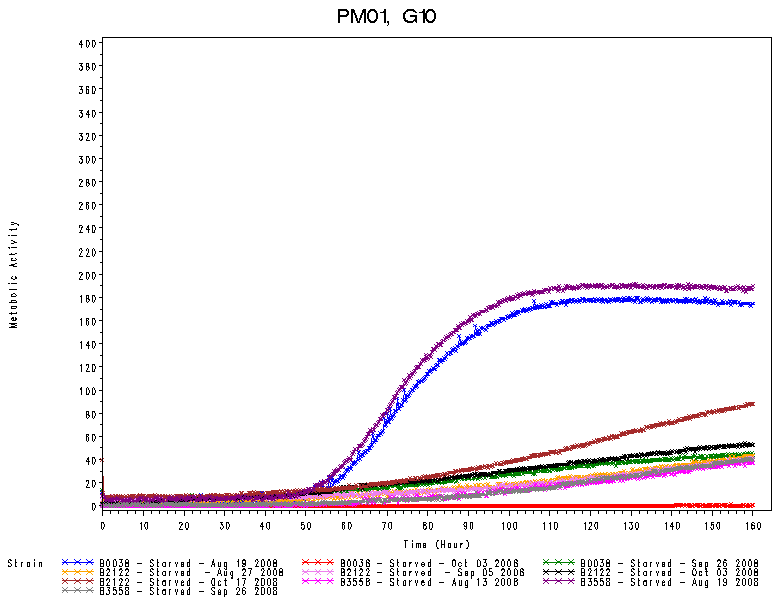

Supplement: Figure S3 — Kinetic curves for all PM plates with Mycobacterium bovis Type 9 strains. (ZIP) [file pone.0052673.s003.zip › suppl fig 3G type 9/Plate01/pm01g101.gif]

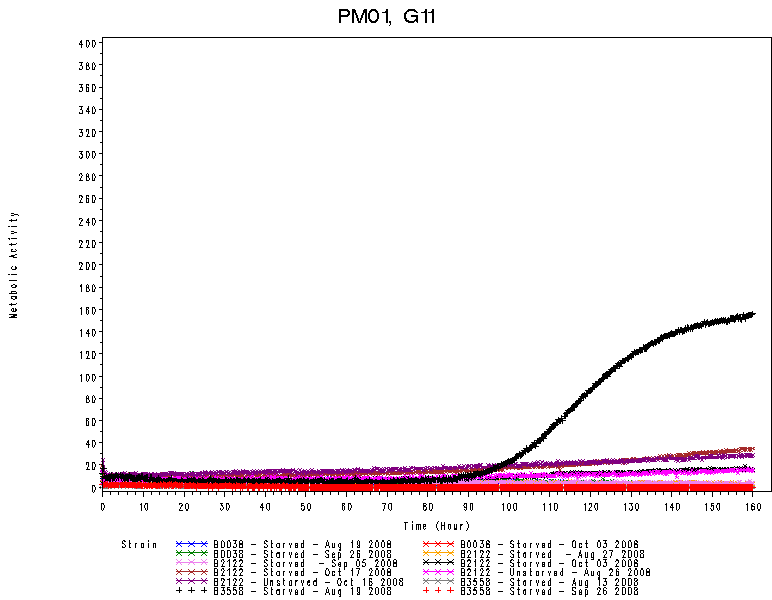

Supplement: Figure S3 — Kinetic curves for all PM plates with Mycobacterium bovis Type 9 strains. (ZIP) [file pone.0052673.s003.zip › suppl fig 3G type 9/Plate01/pm01g11.gif]

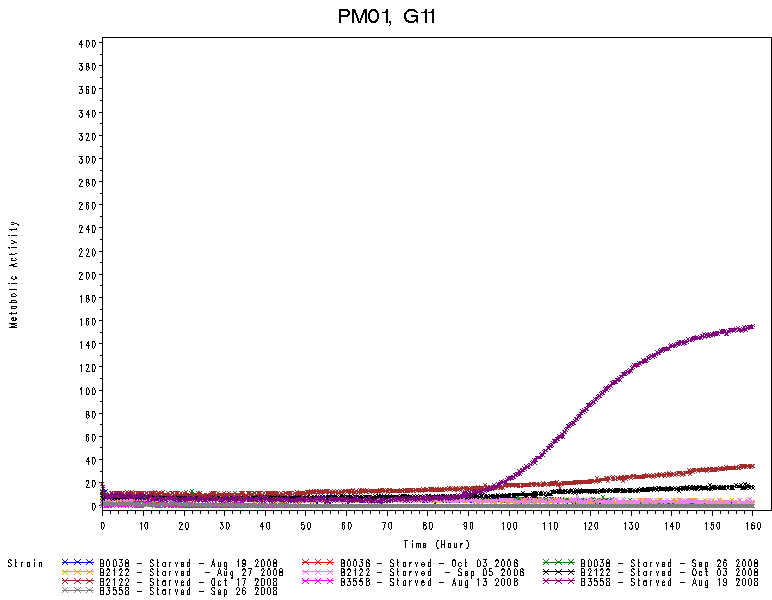

Supplement: Figure S3 — Kinetic curves for all PM plates with Mycobacterium bovis Type 9 strains. (ZIP) [file pone.0052673.s003.zip › suppl fig 3G type 9/Plate01/pm01g111.gif]

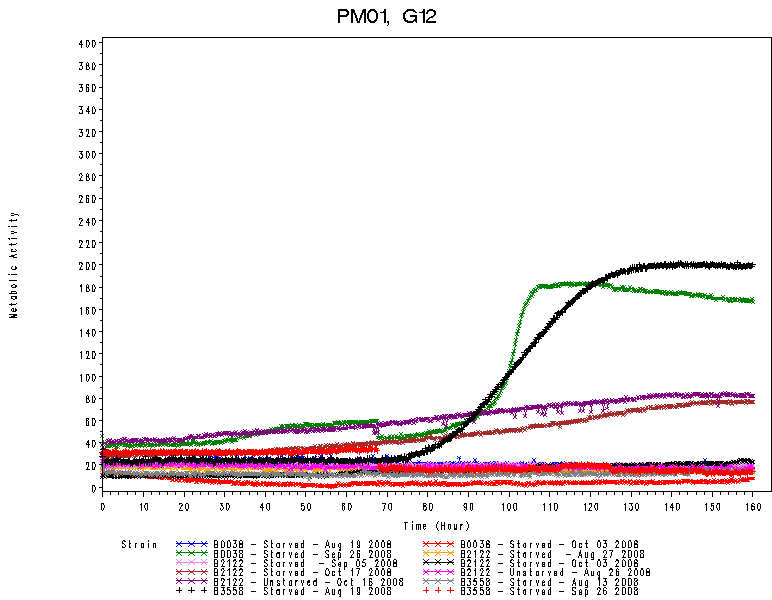

Supplement: Figure S3 — Kinetic curves for all PM plates with Mycobacterium bovis Type 9 strains. (ZIP) [file pone.0052673.s003.zip › suppl fig 3G type 9/Plate01/pm01g12.gif]

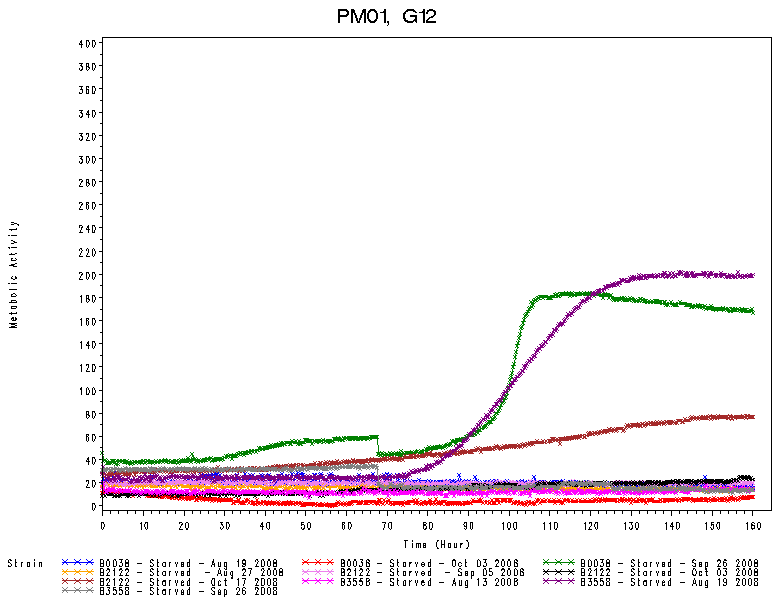

Supplement: Figure S3 — Kinetic curves for all PM plates with Mycobacterium bovis Type 9 strains. (ZIP) [file pone.0052673.s003.zip › suppl fig 3G type 9/Plate01/pm01g121.gif]

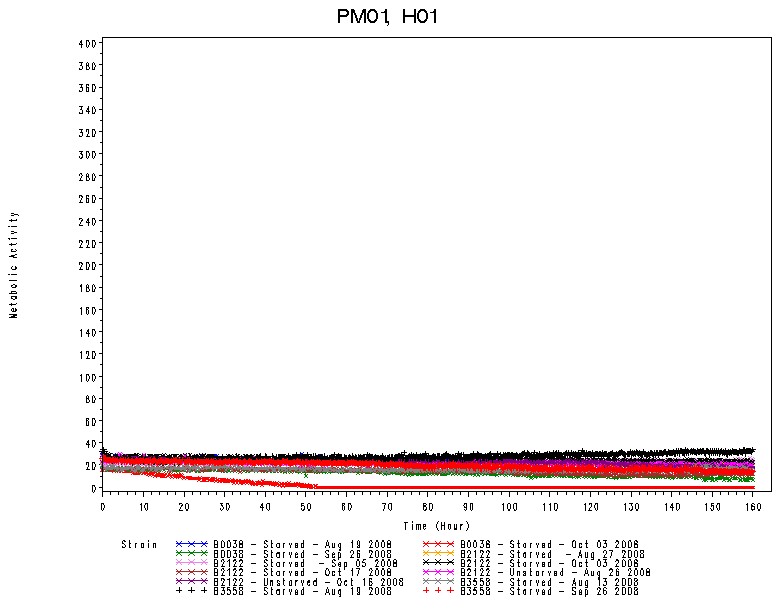

Supplement: Figure S3 — Kinetic curves for all PM plates with Mycobacterium bovis Type 9 strains. (ZIP) [file pone.0052673.s003.zip › suppl fig 3G type 9/Plate01/pm01h01.gif]

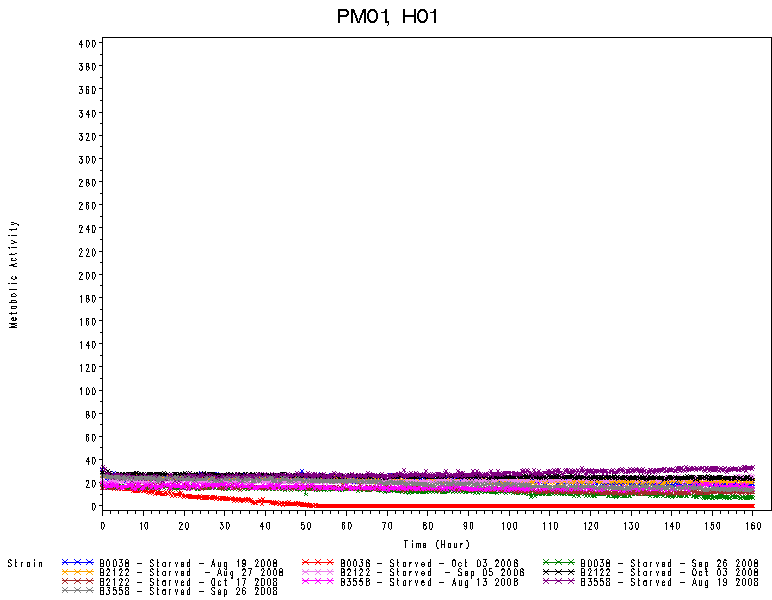

Supplement: Figure S3 — Kinetic curves for all PM plates with Mycobacterium bovis Type 9 strains. (ZIP) [file pone.0052673.s003.zip › suppl fig 3G type 9/Plate01/pm01h011.gif]

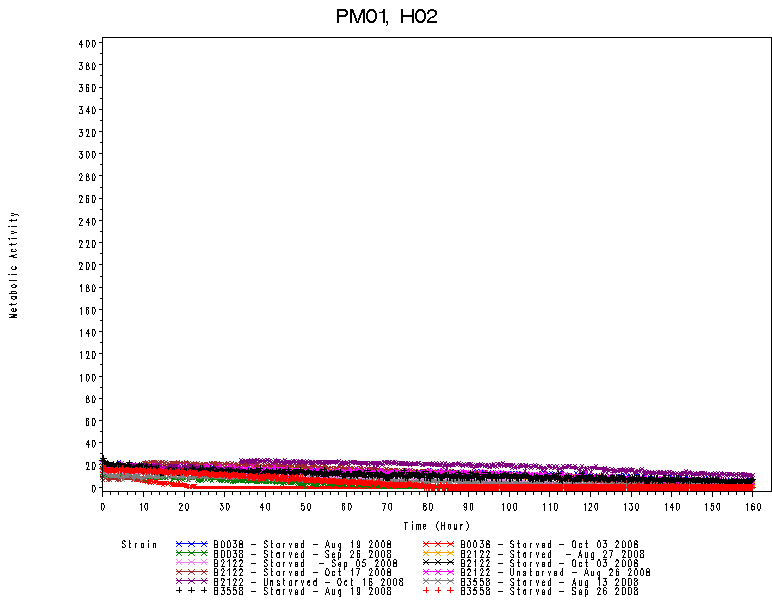

Supplement: Figure S3 — Kinetic curves for all PM plates with Mycobacterium bovis Type 9 strains. (ZIP) [file pone.0052673.s003.zip › suppl fig 3G type 9/Plate01/pm01h02.gif]

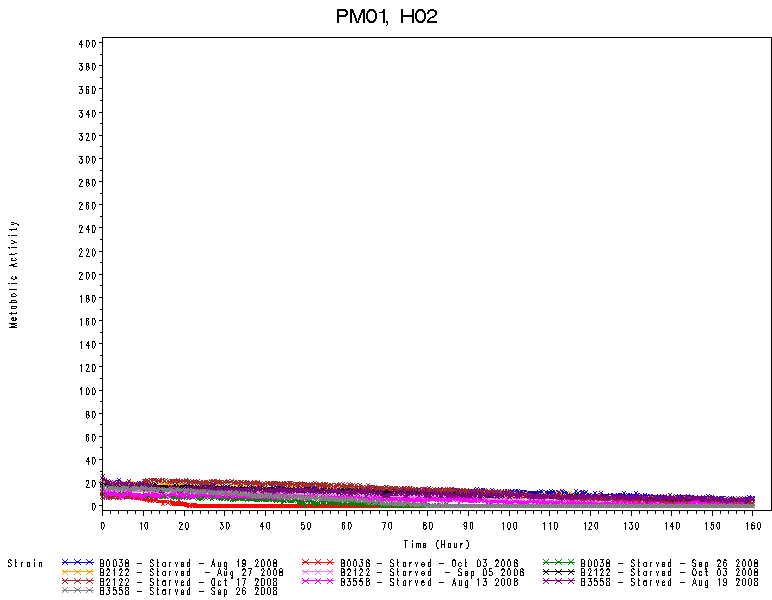

Supplement: Figure S3 — Kinetic curves for all PM plates with Mycobacterium bovis Type 9 strains. (ZIP) [file pone.0052673.s003.zip › suppl fig 3G type 9/Plate01/pm01h021.gif]

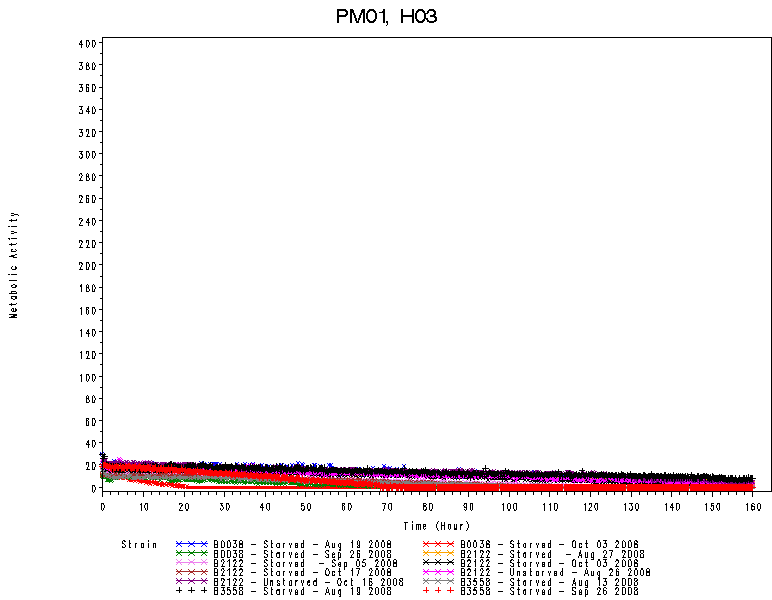

Supplement: Figure S3 — Kinetic curves for all PM plates with Mycobacterium bovis Type 9 strains. (ZIP) [file pone.0052673.s003.zip › suppl fig 3G type 9/Plate01/pm01h03.gif]

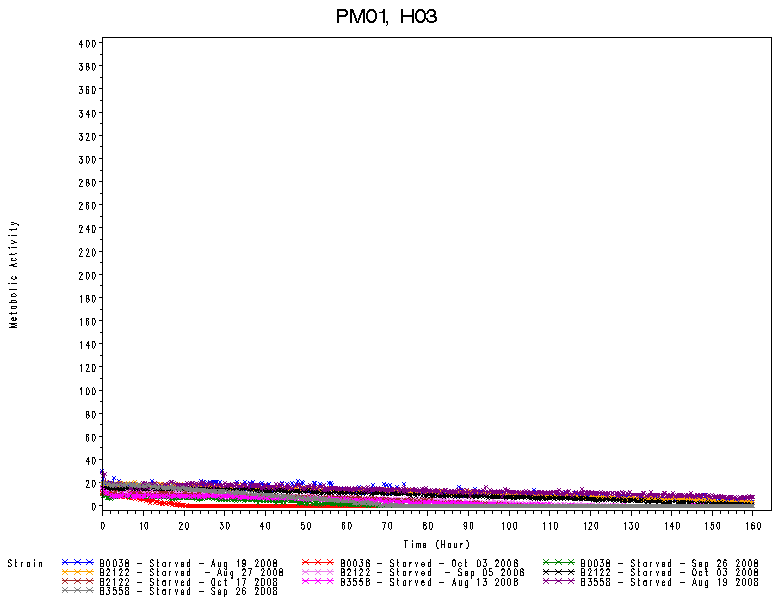

Supplement: Figure S3 — Kinetic curves for all PM plates with Mycobacterium bovis Type 9 strains. (ZIP) [file pone.0052673.s003.zip › suppl fig 3G type 9/Plate01/pm01h031.gif]

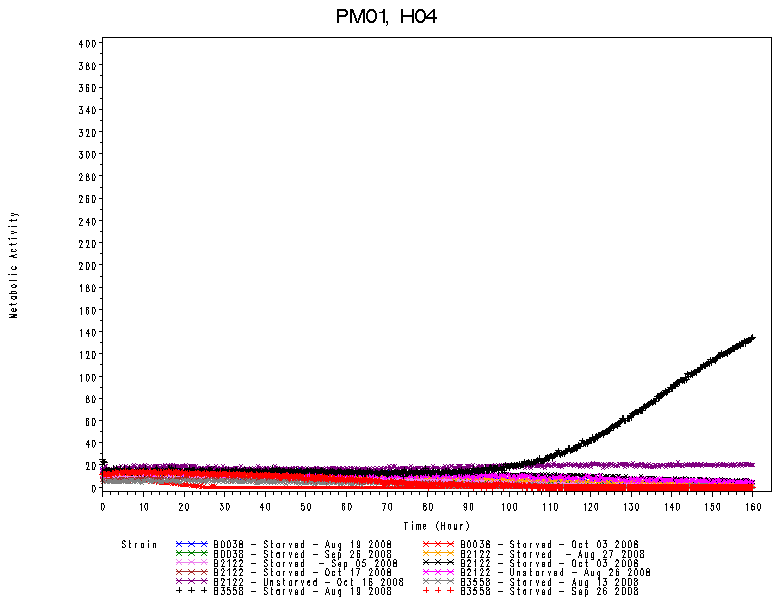

Supplement: Figure S3 — Kinetic curves for all PM plates with Mycobacterium bovis Type 9 strains. (ZIP) [file pone.0052673.s003.zip › suppl fig 3G type 9/Plate01/pm01h04.gif]

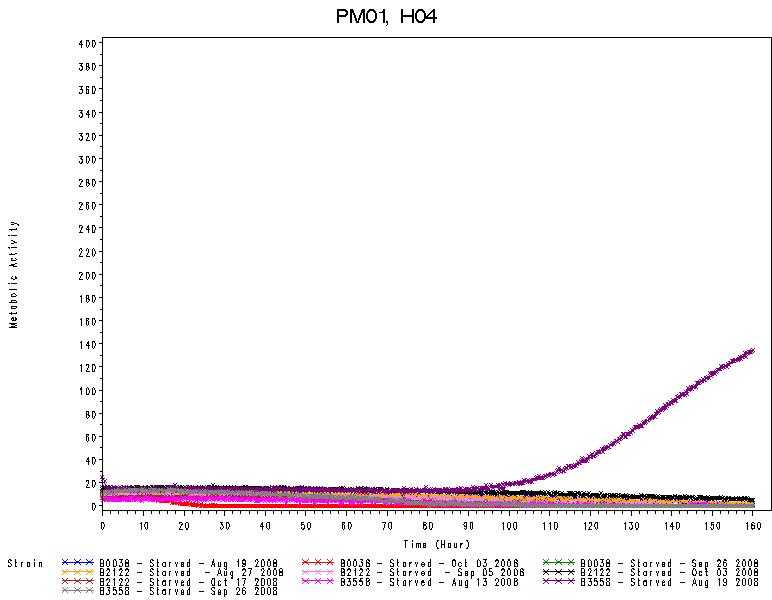

Supplement: Figure S3 — Kinetic curves for all PM plates with Mycobacterium bovis Type 9 strains. (ZIP) [file pone.0052673.s003.zip › suppl fig 3G type 9/Plate01/pm01h041.gif]

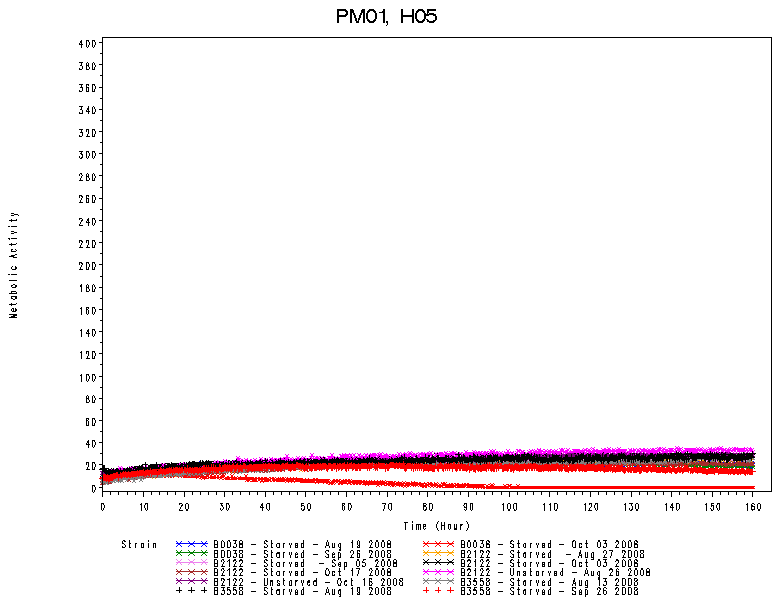

Supplement: Figure S3 — Kinetic curves for all PM plates with Mycobacterium bovis Type 9 strains. (ZIP) [file pone.0052673.s003.zip › suppl fig 3G type 9/Plate01/pm01h05.gif]

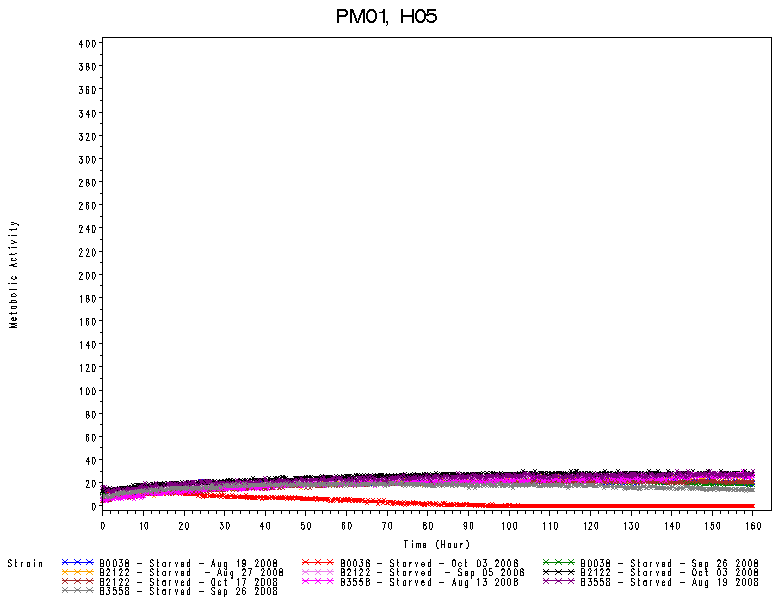

Supplement: Figure S3 — Kinetic curves for all PM plates with Mycobacterium bovis Type 9 strains. (ZIP) [file pone.0052673.s003.zip › suppl fig 3G type 9/Plate01/pm01h051.gif]

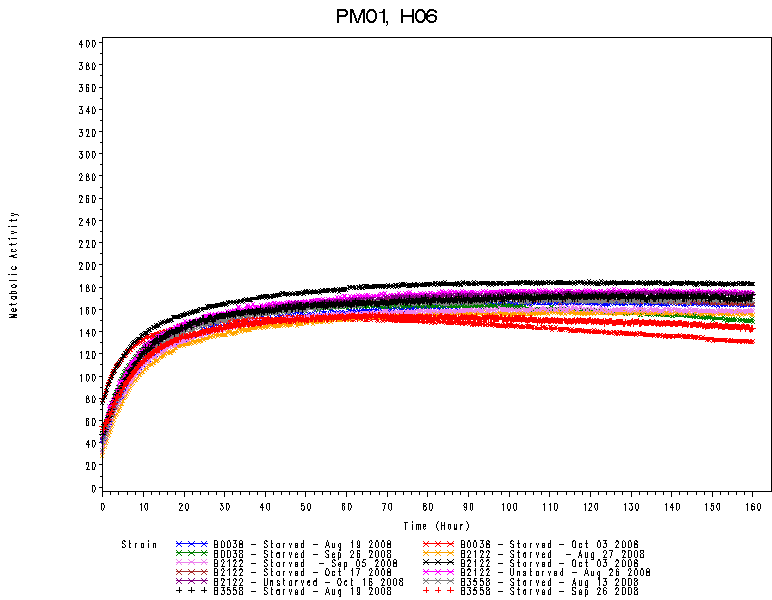

Supplement: Figure S3 — Kinetic curves for all PM plates with Mycobacterium bovis Type 9 strains. (ZIP) [file pone.0052673.s003.zip › suppl fig 3G type 9/Plate01/pm01h06.gif]

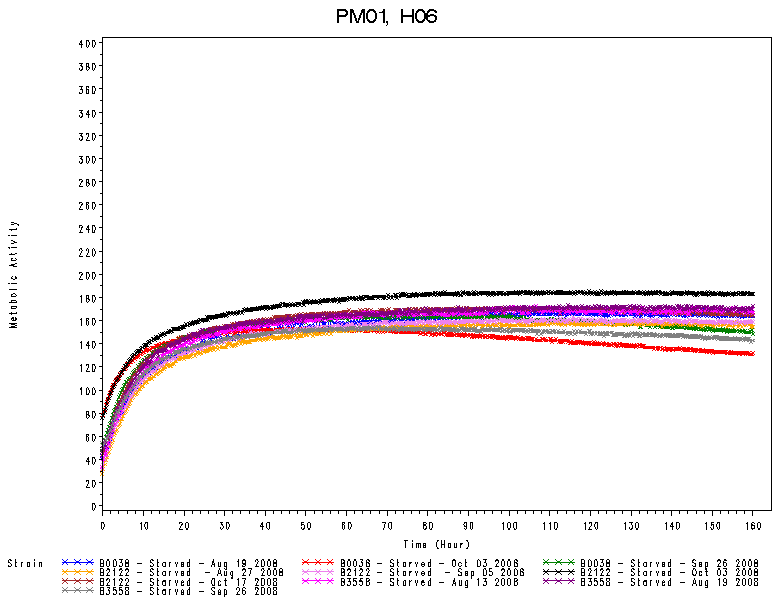

Supplement: Figure S3 — Kinetic curves for all PM plates with Mycobacterium bovis Type 9 strains. (ZIP) [file pone.0052673.s003.zip › suppl fig 3G type 9/Plate01/pm01h061.gif]

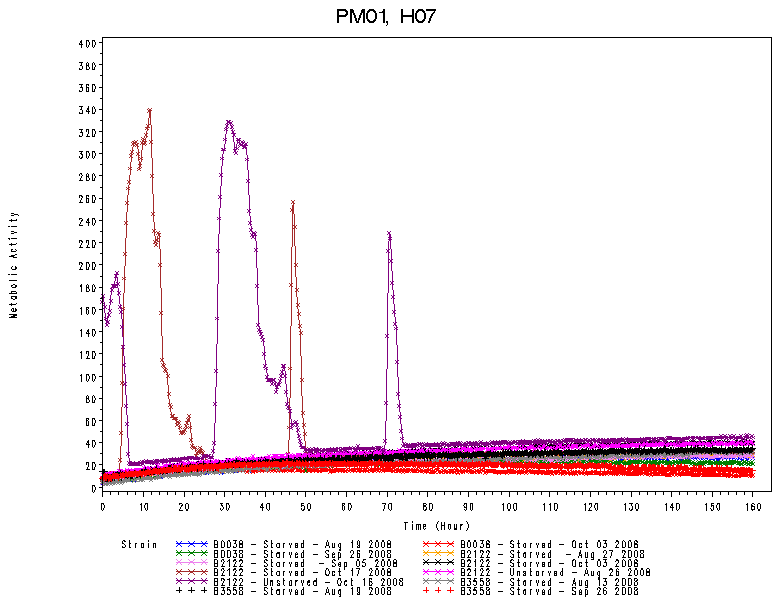

Supplement: Figure S3 — Kinetic curves for all PM plates with Mycobacterium bovis Type 9 strains. (ZIP) [file pone.0052673.s003.zip › suppl fig 3G type 9/Plate01/pm01h07.gif]

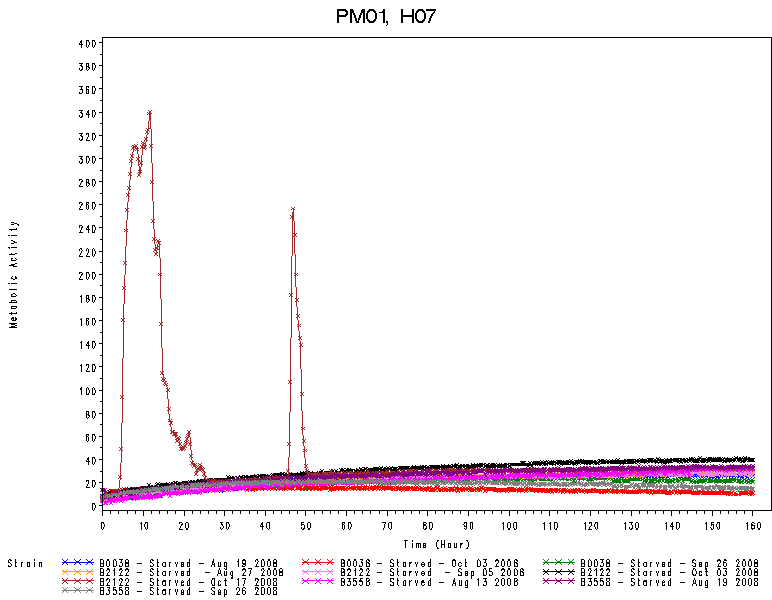

Supplement: Figure S3 — Kinetic curves for all PM plates with Mycobacterium bovis Type 9 strains. (ZIP) [file pone.0052673.s003.zip › suppl fig 3G type 9/Plate01/pm01h071.gif]

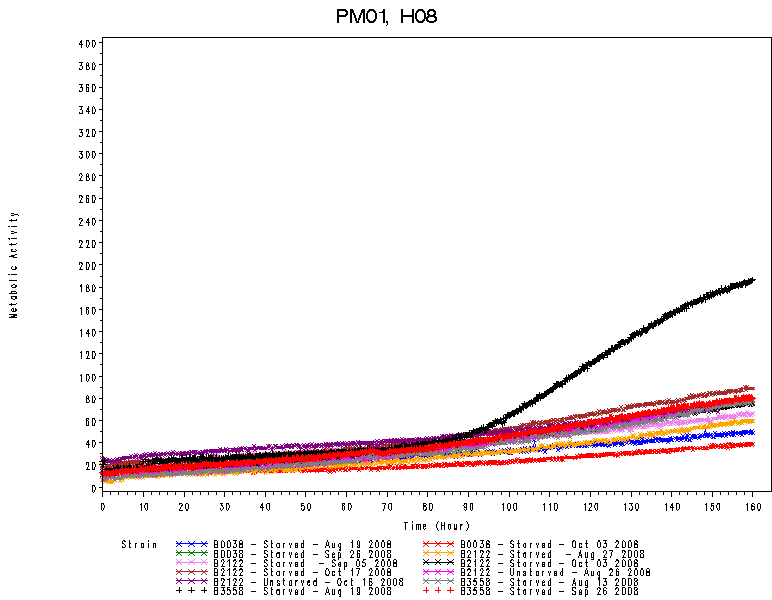

Supplement: Figure S3 — Kinetic curves for all PM plates with Mycobacterium bovis Type 9 strains. (ZIP) [file pone.0052673.s003.zip › suppl fig 3G type 9/Plate01/pm01h08.gif]

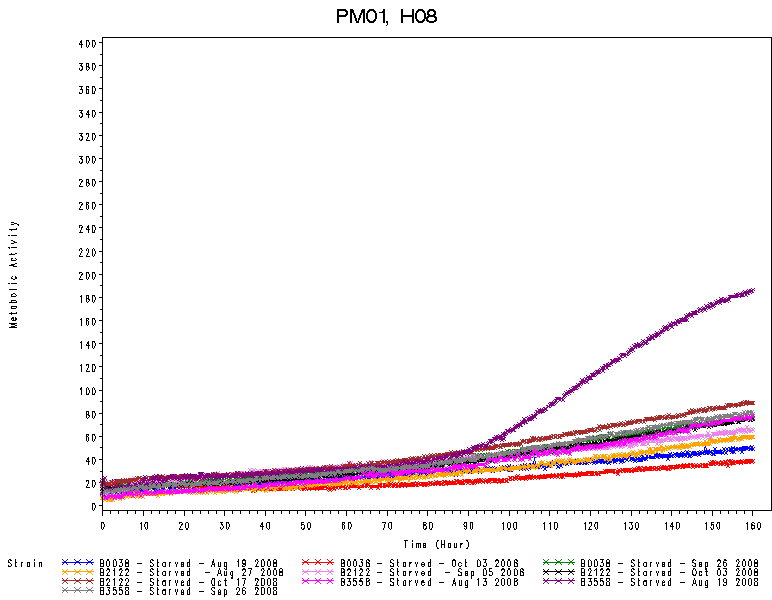

Supplement: Figure S3 — Kinetic curves for all PM plates with Mycobacterium bovis Type 9 strains. (ZIP) [file pone.0052673.s003.zip › suppl fig 3G type 9/Plate01/pm01h081.gif]

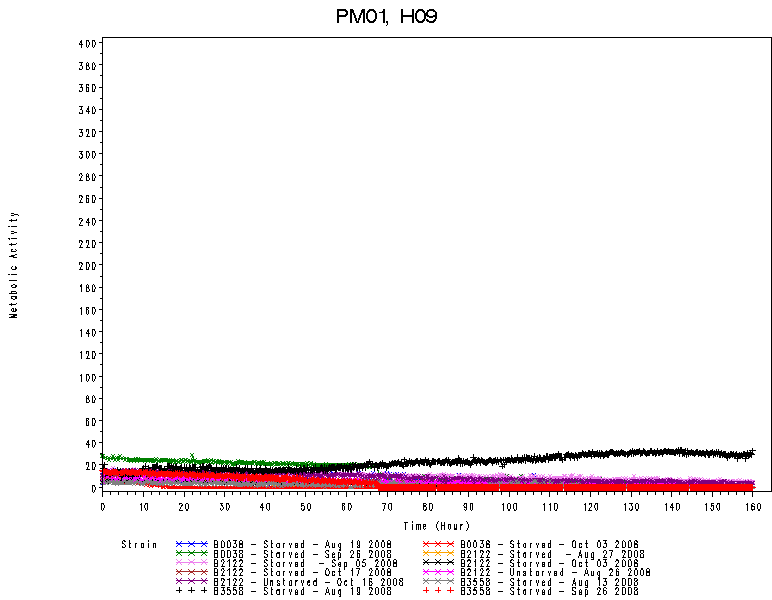

Supplement: Figure S3 — Kinetic curves for all PM plates with Mycobacterium bovis Type 9 strains. (ZIP) [file pone.0052673.s003.zip › suppl fig 3G type 9/Plate01/pm01h09.gif]

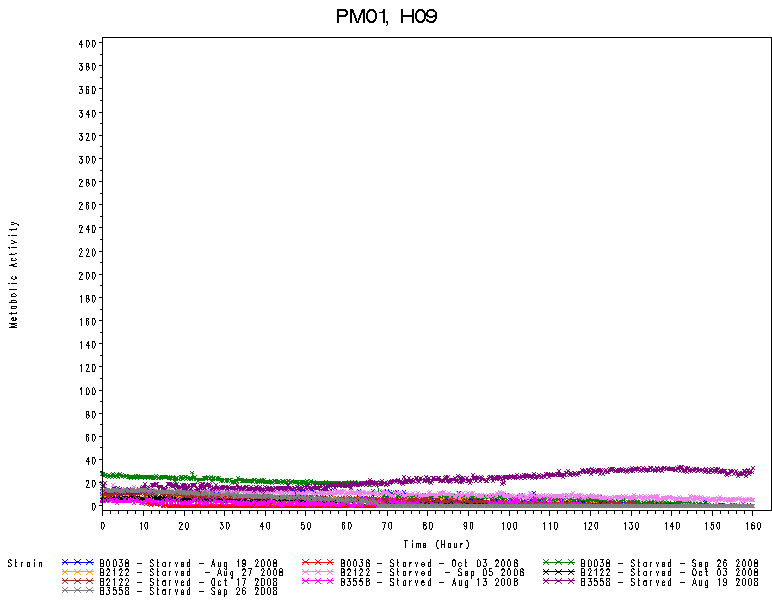

Supplement: Figure S3 — Kinetic curves for all PM plates with Mycobacterium bovis Type 9 strains. (ZIP) [file pone.0052673.s003.zip › suppl fig 3G type 9/Plate01/pm01h091.gif]

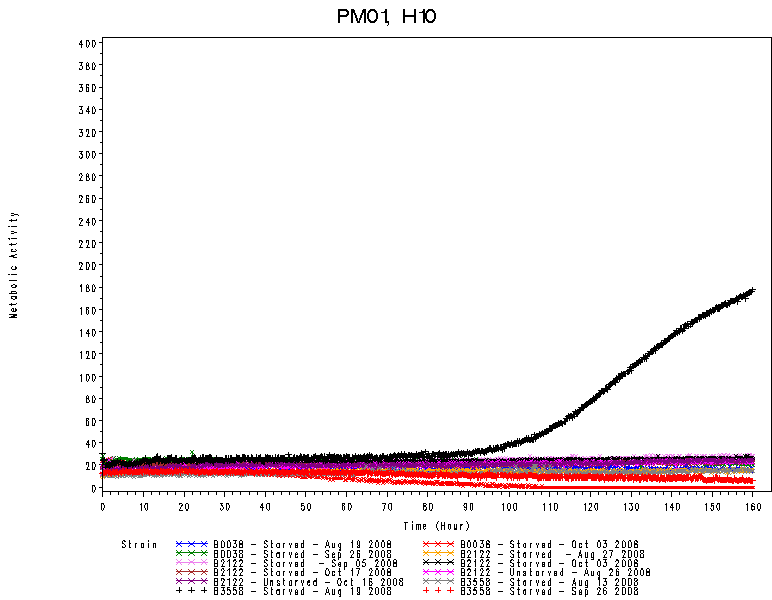

Supplement: Figure S3 — Kinetic curves for all PM plates with Mycobacterium bovis Type 9 strains. (ZIP) [file pone.0052673.s003.zip › suppl fig 3G type 9/Plate01/pm01h10.gif]

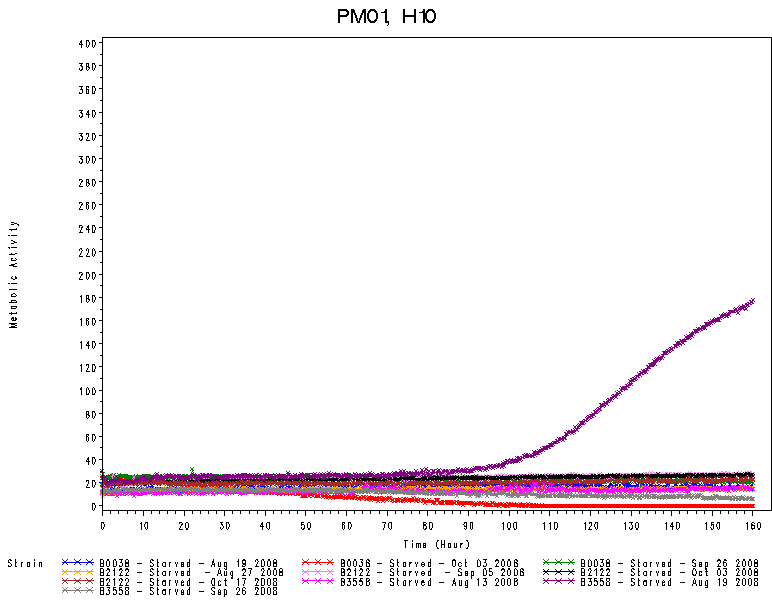

Supplement: Figure S3 — Kinetic curves for all PM plates with Mycobacterium bovis Type 9 strains. (ZIP) [file pone.0052673.s003.zip › suppl fig 3G type 9/Plate01/pm01h101.gif]

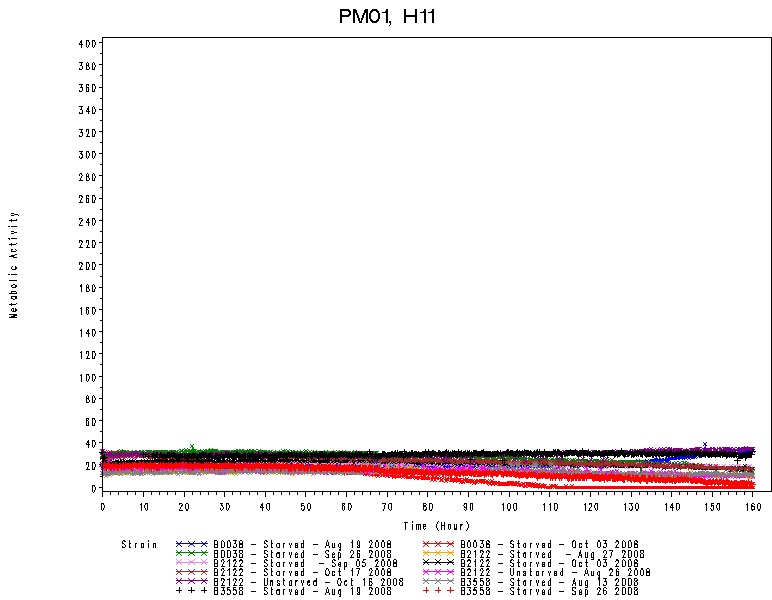

Supplement: Figure S3 — Kinetic curves for all PM plates with Mycobacterium bovis Type 9 strains. (ZIP) [file pone.0052673.s003.zip › suppl fig 3G type 9/Plate01/pm01h11.gif]

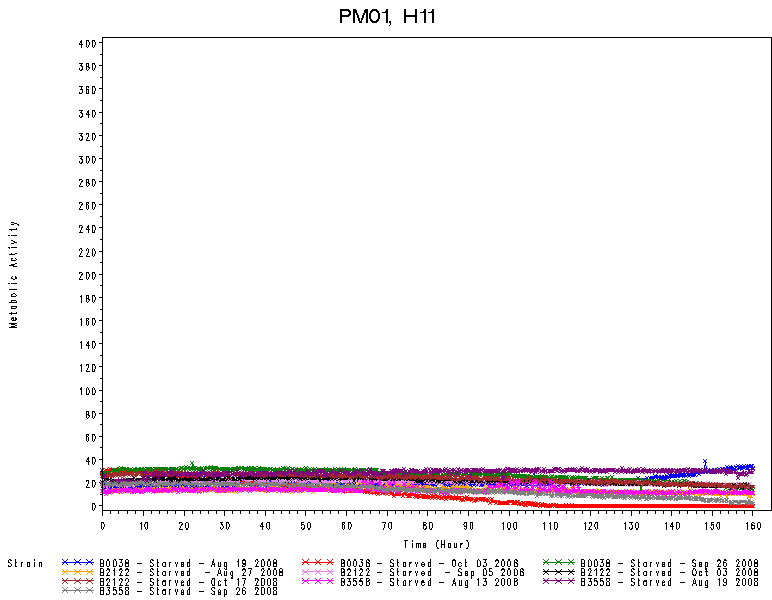

Supplement: Figure S3 — Kinetic curves for all PM plates with Mycobacterium bovis Type 9 strains. (ZIP) [file pone.0052673.s003.zip › suppl fig 3G type 9/Plate01/pm01h111.gif]

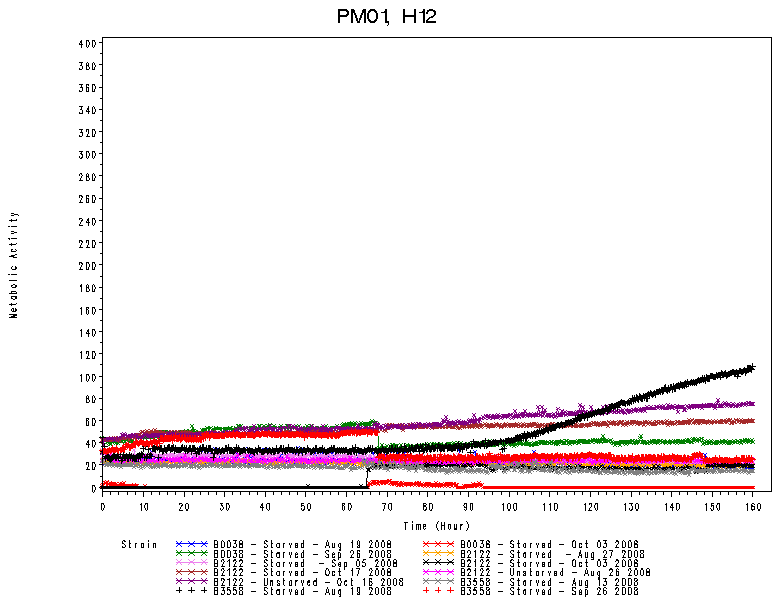

Supplement: Figure S3 — Kinetic curves for all PM plates with Mycobacterium bovis Type 9 strains. (ZIP) [file pone.0052673.s003.zip › suppl fig 3G type 9/Plate01/pm01h12.gif]

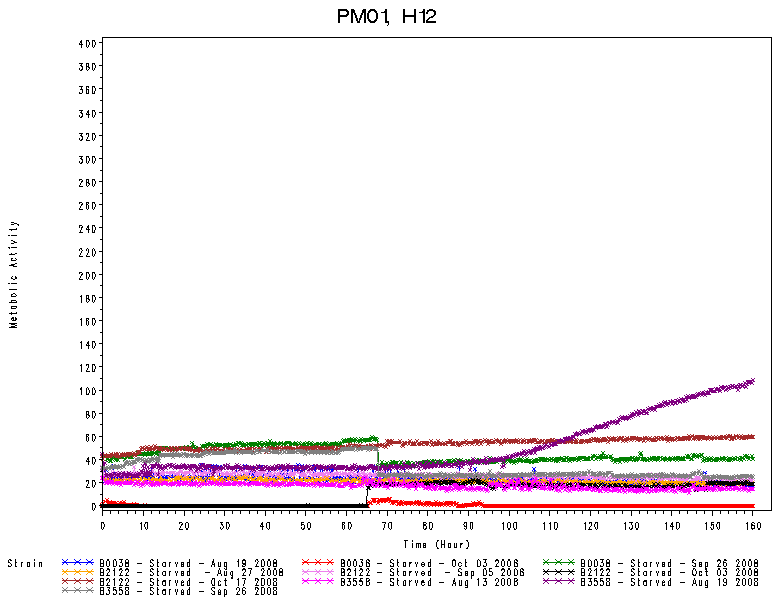

Supplement: Figure S3 — Kinetic curves for all PM plates with Mycobacterium bovis Type 9 strains. (ZIP) [file pone.0052673.s003.zip › suppl fig 3G type 9/Plate01/pm01h121.gif]

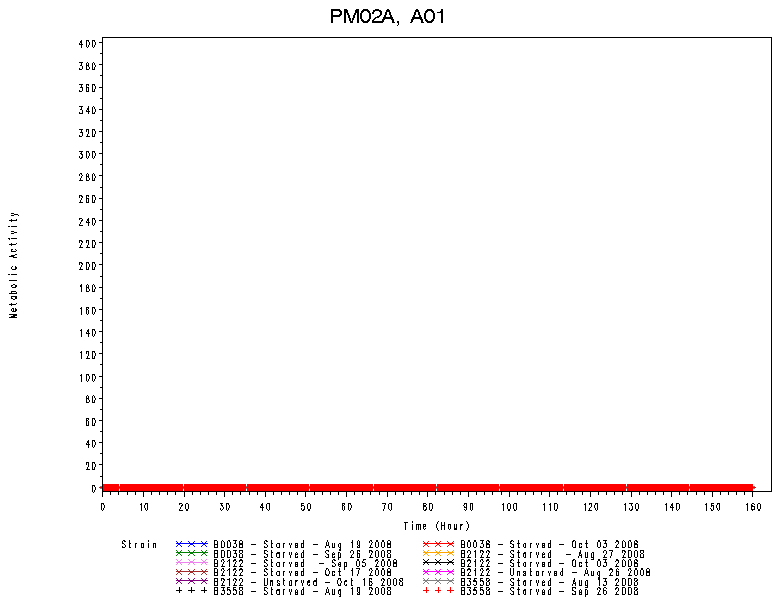

Supplement: Figure S3 — Kinetic curves for all PM plates with Mycobacterium bovis Type 9 strains. (ZIP) [file pone.0052673.s003.zip › suppl fig 3G type 9/Plate02A/pm02aa01.gif]

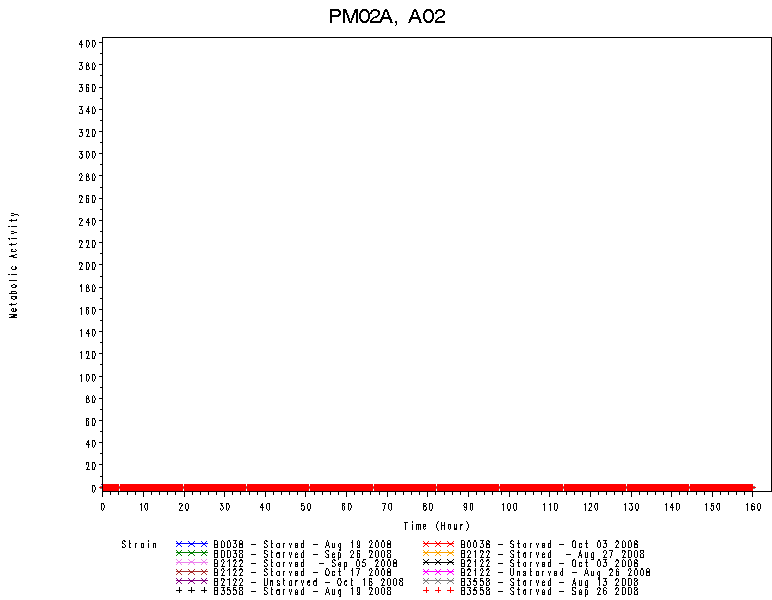

Supplement: Figure S3 — Kinetic curves for all PM plates with Mycobacterium bovis Type 9 strains. (ZIP) [file pone.0052673.s003.zip › suppl fig 3G type 9/Plate02A/pm02aa02.gif]

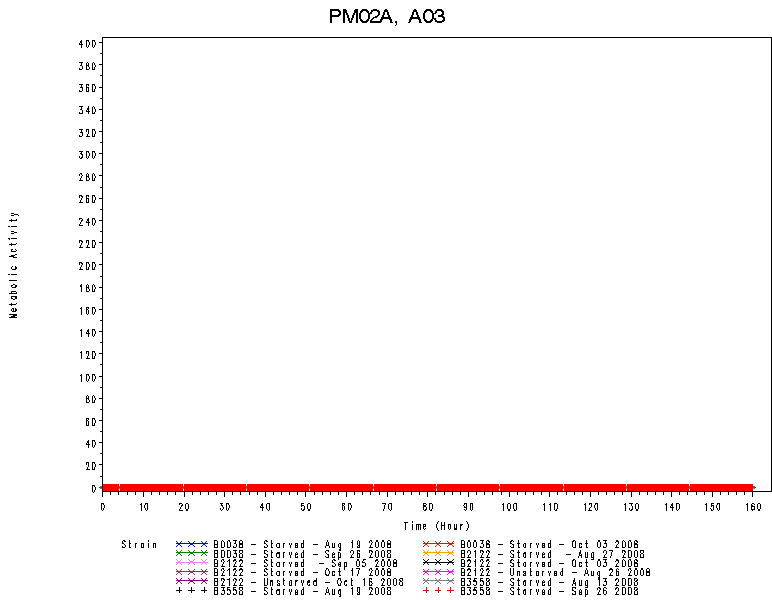

Supplement: Figure S3 — Kinetic curves for all PM plates with Mycobacterium bovis Type 9 strains. (ZIP) [file pone.0052673.s003.zip › suppl fig 3G type 9/Plate02A/pm02aa03.gif]

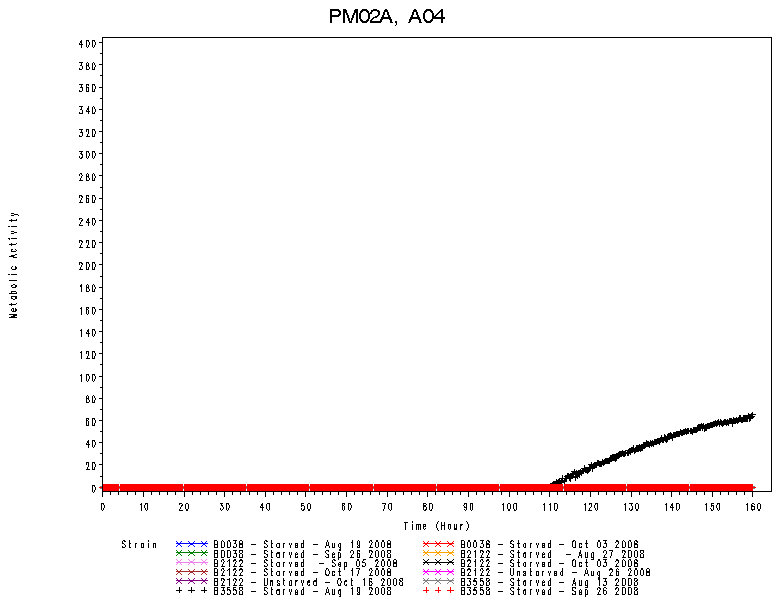

Supplement: Figure S3 — Kinetic curves for all PM plates with Mycobacterium bovis Type 9 strains. (ZIP) [file pone.0052673.s003.zip › suppl fig 3G type 9/Plate02A/pm02aa04.gif]

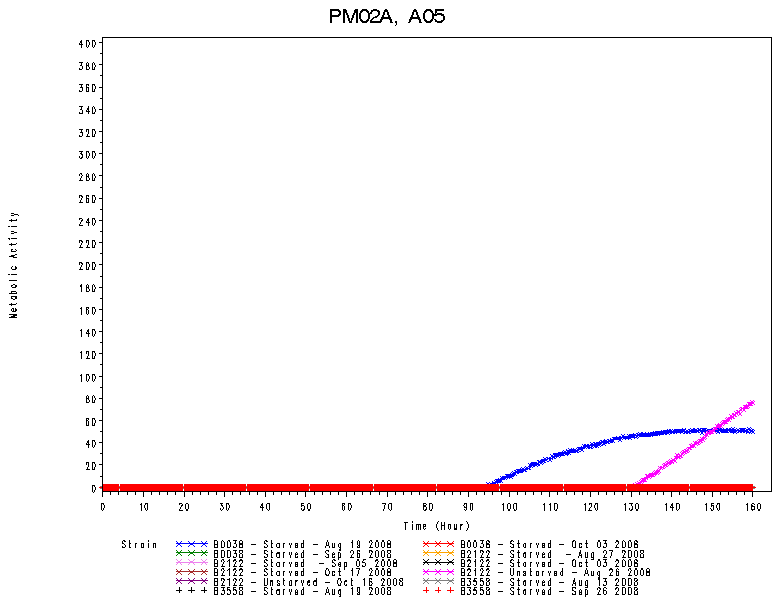

Supplement: Figure S3 — Kinetic curves for all PM plates with Mycobacterium bovis Type 9 strains. (ZIP) [file pone.0052673.s003.zip › suppl fig 3G type 9/Plate02A/pm02aa05.gif]

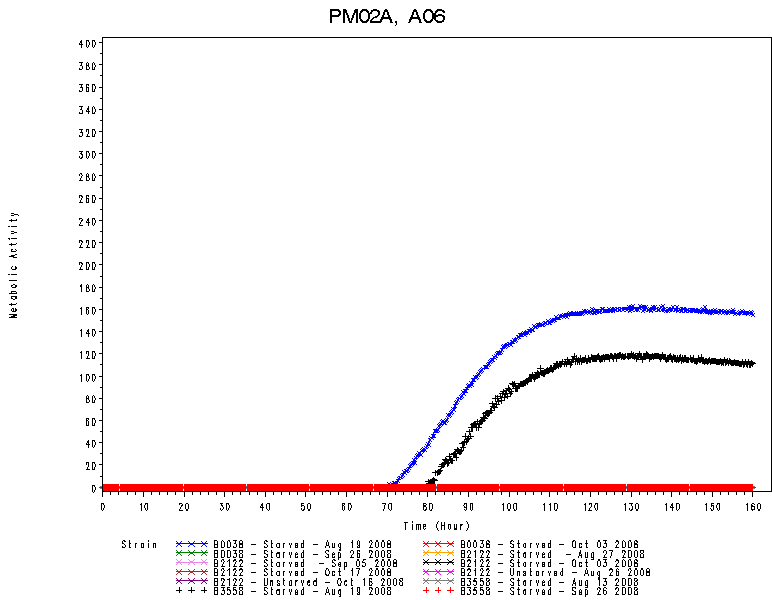

Supplement: Figure S3 — Kinetic curves for all PM plates with Mycobacterium bovis Type 9 strains. (ZIP) [file pone.0052673.s003.zip › suppl fig 3G type 9/Plate02A/pm02aa06.gif]

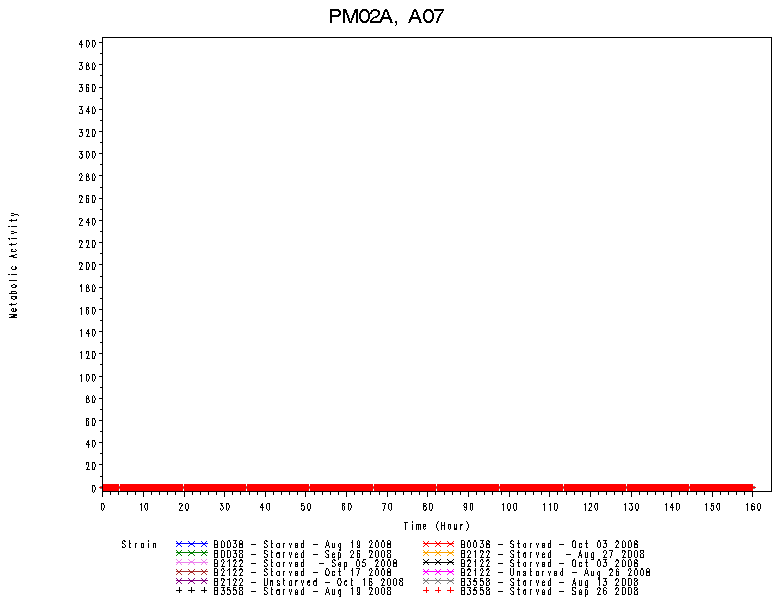

Supplement: Figure S3 — Kinetic curves for all PM plates with Mycobacterium bovis Type 9 strains. (ZIP) [file pone.0052673.s003.zip › suppl fig 3G type 9/Plate02A/pm02aa07.gif]

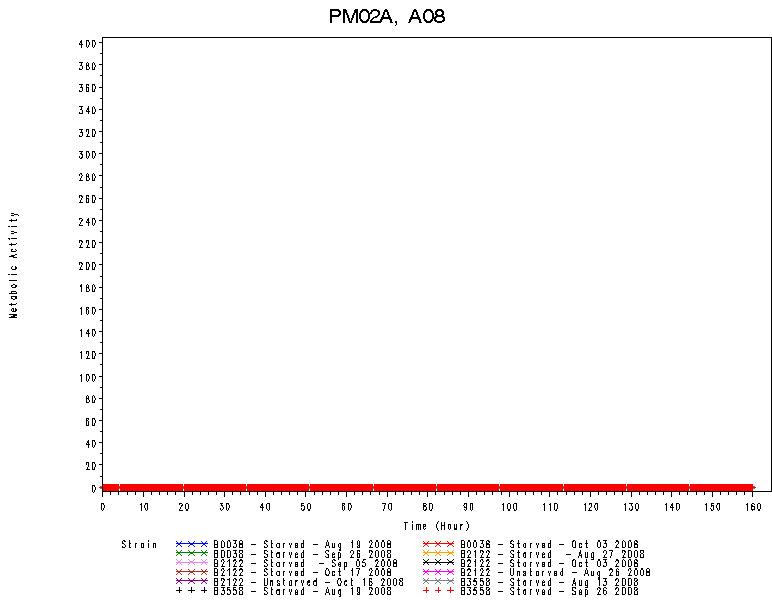

Supplement: Figure S3 — Kinetic curves for all PM plates with Mycobacterium bovis Type 9 strains. (ZIP) [file pone.0052673.s003.zip › suppl fig 3G type 9/Plate02A/pm02aa08.gif]
