# Supplementary figures and images for: High Throughput Phenotypic Analysis of Mycobacterium tuberculosis and Mycobacterium bovis Strains' Metabolism Using Biolog Phenotype Microarrays (part 5 of 11)
Source: PLoS One. 2013 Jan 10;8(1):e52673. doi: 10.1371/journal.pone.0052673 (PMC3542357; doi:10.1371/journal.pone.0052673)

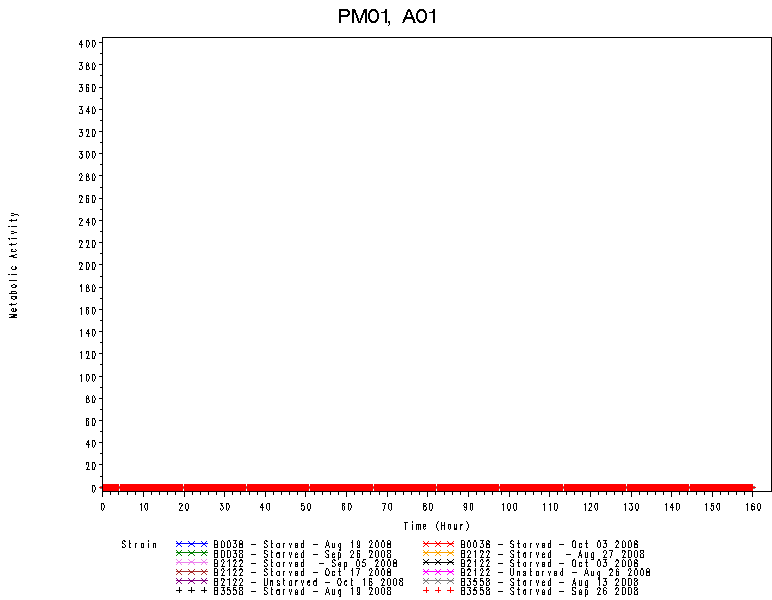

Supplement: Figure S3 — Kinetic curves for all PM plates with Mycobacterium bovis Type 9 strains. (ZIP) [file pone.0052673.s003.zip › suppl fig 3G type 9/Plate01/pm01a01.gif]

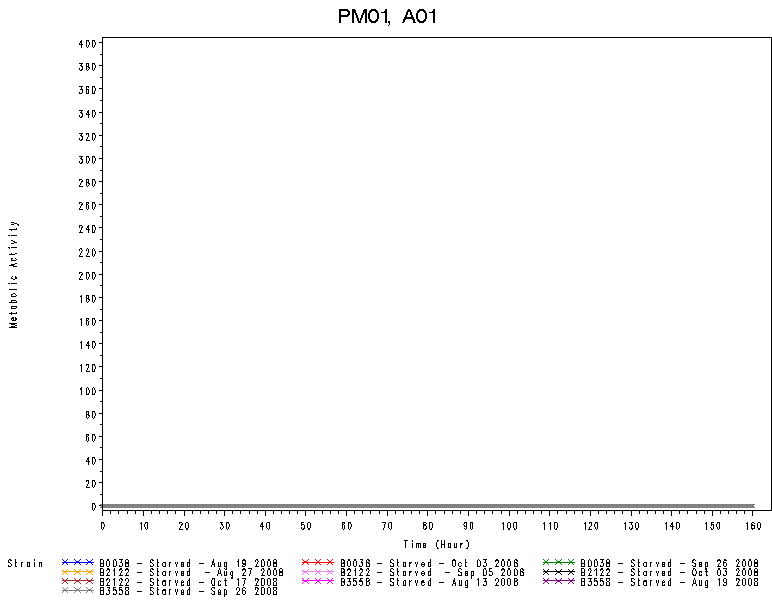

Supplement: Figure S3 — Kinetic curves for all PM plates with Mycobacterium bovis Type 9 strains. (ZIP) [file pone.0052673.s003.zip › suppl fig 3G type 9/Plate01/pm01a011.gif]

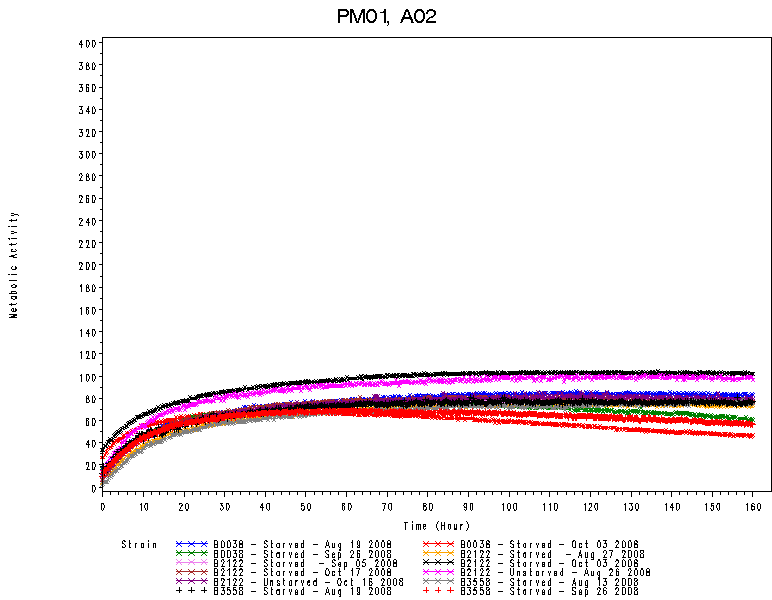

Supplement: Figure S3 — Kinetic curves for all PM plates with Mycobacterium bovis Type 9 strains. (ZIP) [file pone.0052673.s003.zip › suppl fig 3G type 9/Plate01/pm01a02.gif]

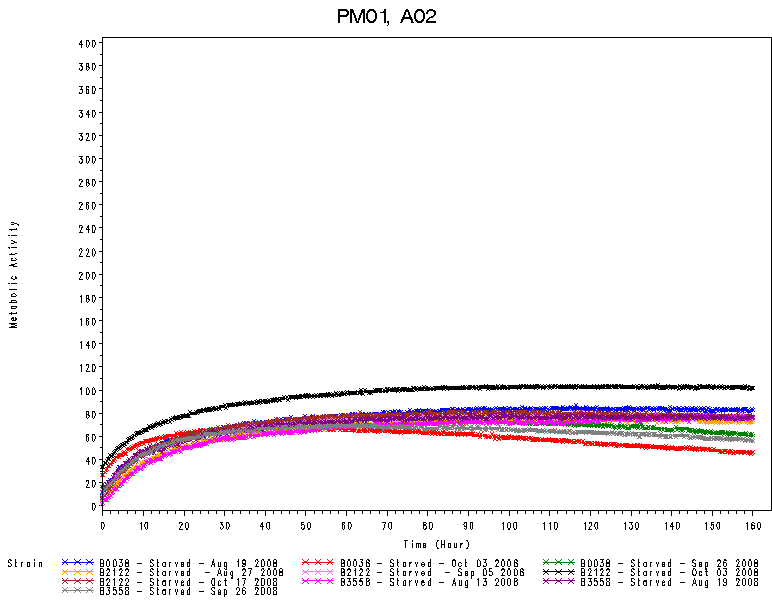

Supplement: Figure S3 — Kinetic curves for all PM plates with Mycobacterium bovis Type 9 strains. (ZIP) [file pone.0052673.s003.zip › suppl fig 3G type 9/Plate01/pm01a021.gif]

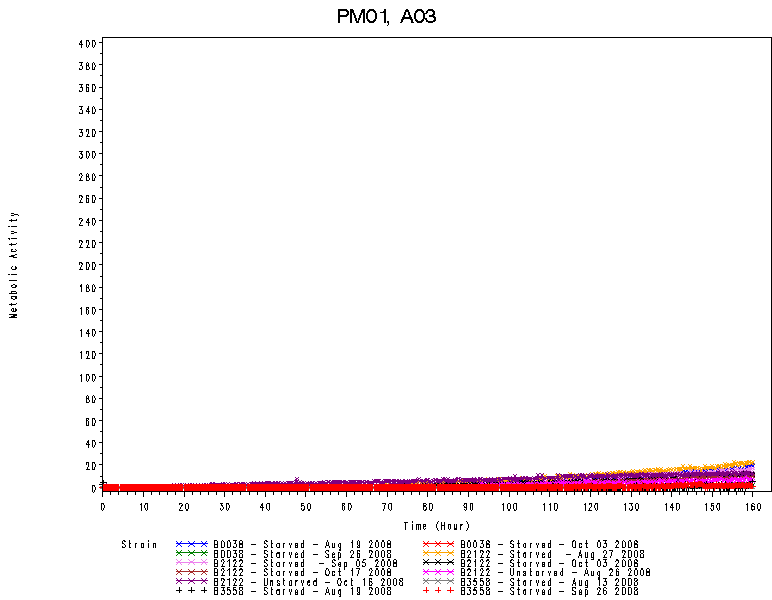

Supplement: Figure S3 — Kinetic curves for all PM plates with Mycobacterium bovis Type 9 strains. (ZIP) [file pone.0052673.s003.zip › suppl fig 3G type 9/Plate01/pm01a03.gif]

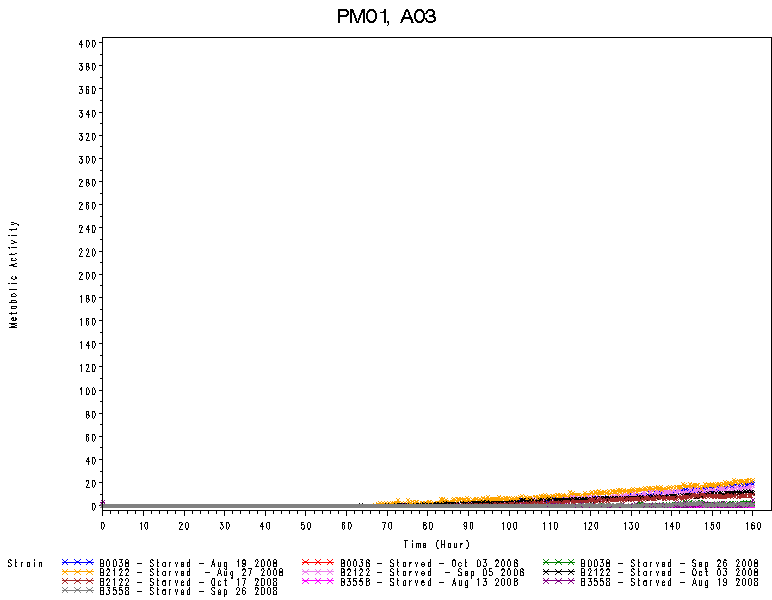

Supplement: Figure S3 — Kinetic curves for all PM plates with Mycobacterium bovis Type 9 strains. (ZIP) [file pone.0052673.s003.zip › suppl fig 3G type 9/Plate01/pm01a031.gif]

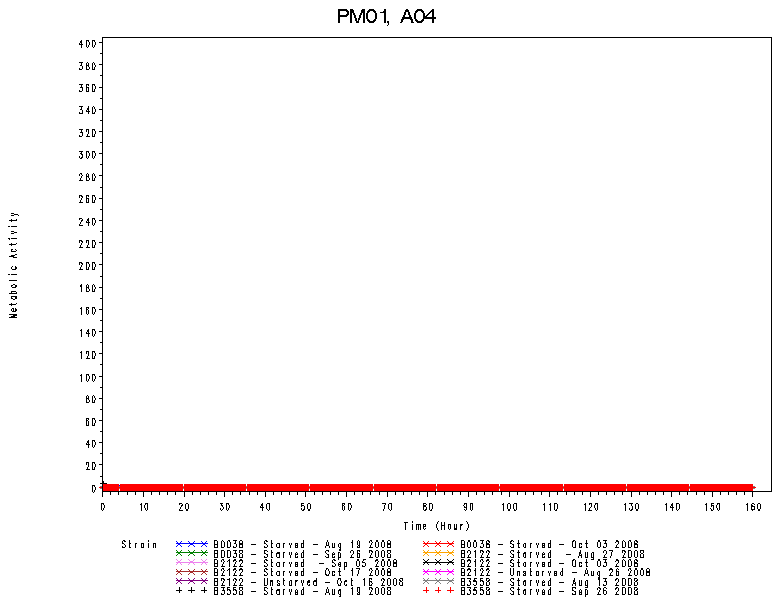

Supplement: Figure S3 — Kinetic curves for all PM plates with Mycobacterium bovis Type 9 strains. (ZIP) [file pone.0052673.s003.zip › suppl fig 3G type 9/Plate01/pm01a04.gif]

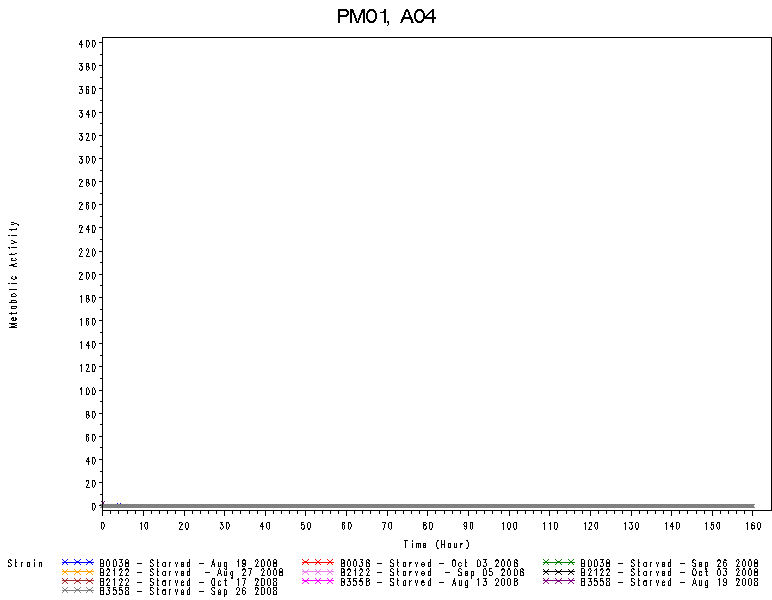

Supplement: Figure S3 — Kinetic curves for all PM plates with Mycobacterium bovis Type 9 strains. (ZIP) [file pone.0052673.s003.zip › suppl fig 3G type 9/Plate01/pm01a041.gif]

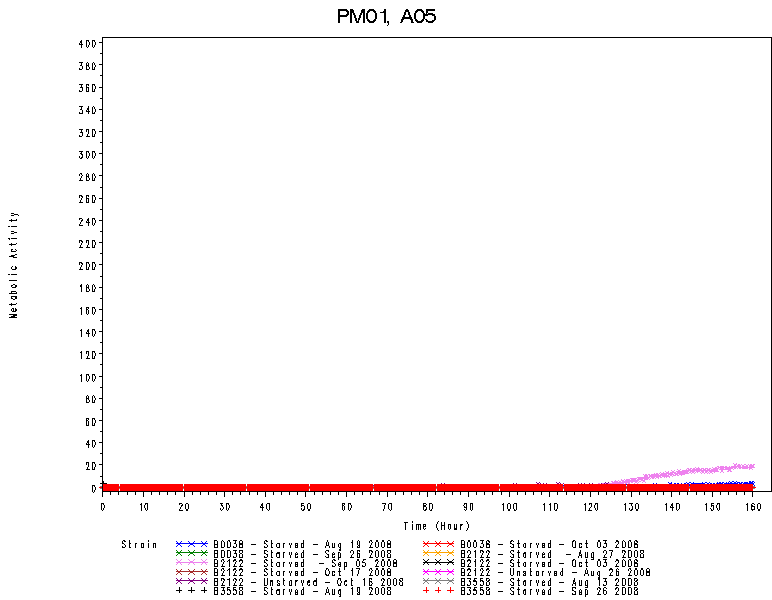

Supplement: Figure S3 — Kinetic curves for all PM plates with Mycobacterium bovis Type 9 strains. (ZIP) [file pone.0052673.s003.zip › suppl fig 3G type 9/Plate01/pm01a05.gif]

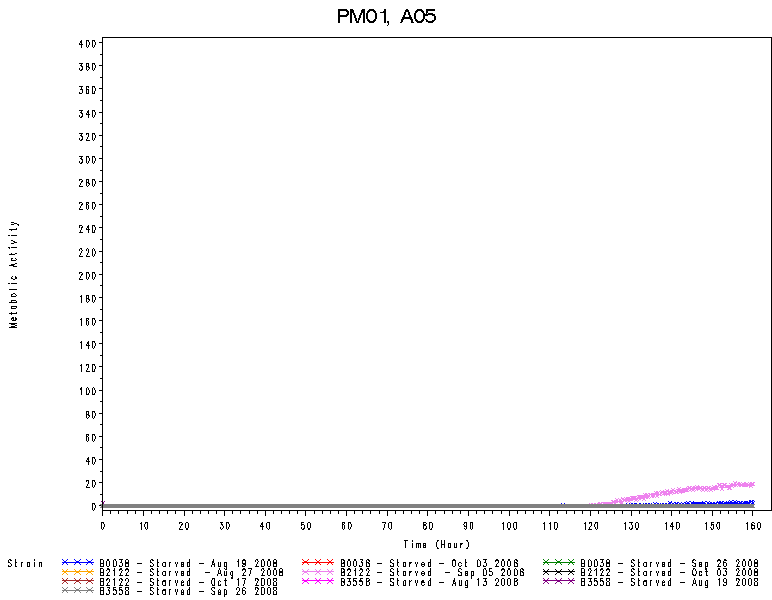

Supplement: Figure S3 — Kinetic curves for all PM plates with Mycobacterium bovis Type 9 strains. (ZIP) [file pone.0052673.s003.zip › suppl fig 3G type 9/Plate01/pm01a051.gif]

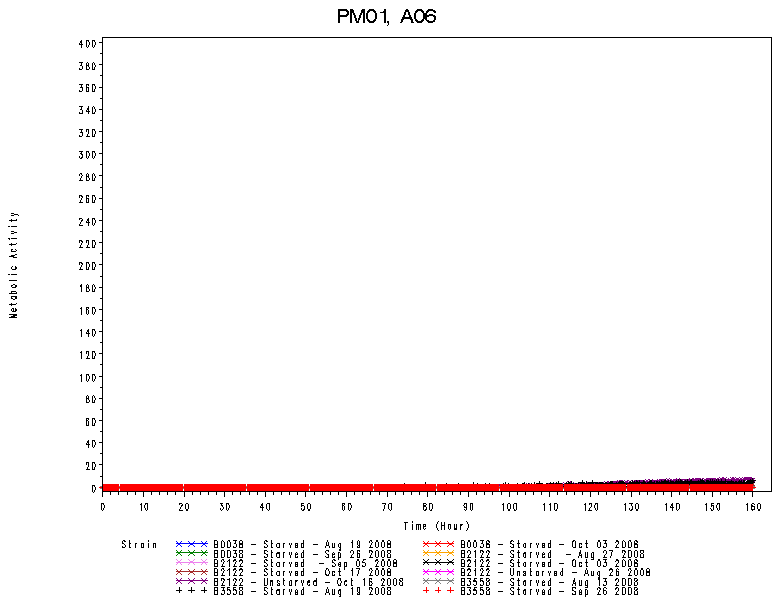

Supplement: Figure S3 — Kinetic curves for all PM plates with Mycobacterium bovis Type 9 strains. (ZIP) [file pone.0052673.s003.zip › suppl fig 3G type 9/Plate01/pm01a06.gif]

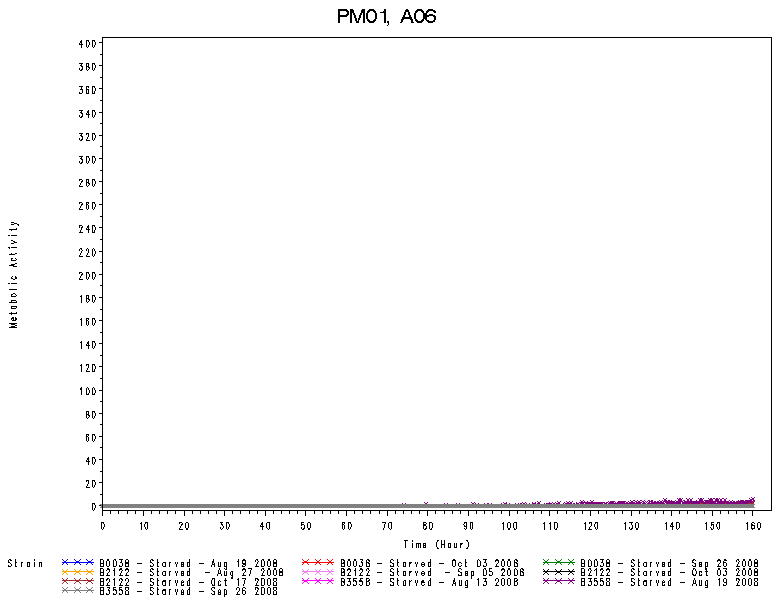

Supplement: Figure S3 — Kinetic curves for all PM plates with Mycobacterium bovis Type 9 strains. (ZIP) [file pone.0052673.s003.zip › suppl fig 3G type 9/Plate01/pm01a061.gif]

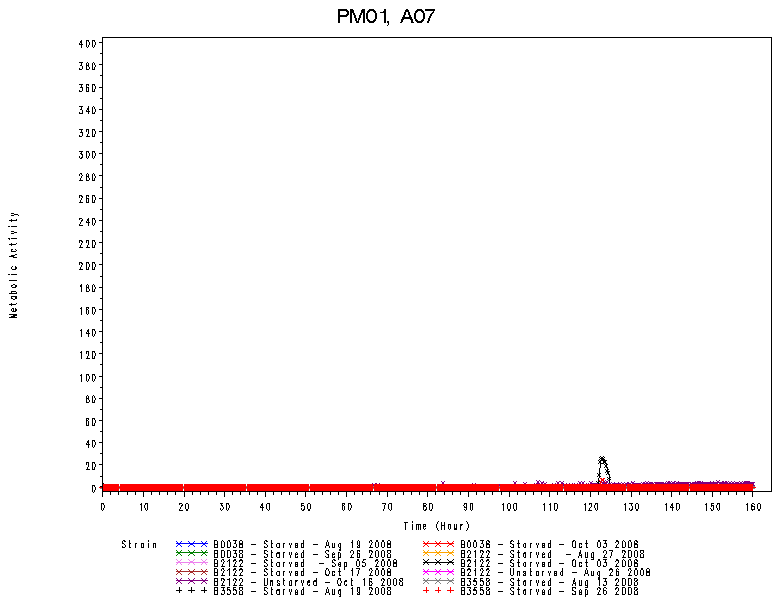

Supplement: Figure S3 — Kinetic curves for all PM plates with Mycobacterium bovis Type 9 strains. (ZIP) [file pone.0052673.s003.zip › suppl fig 3G type 9/Plate01/pm01a07.gif]

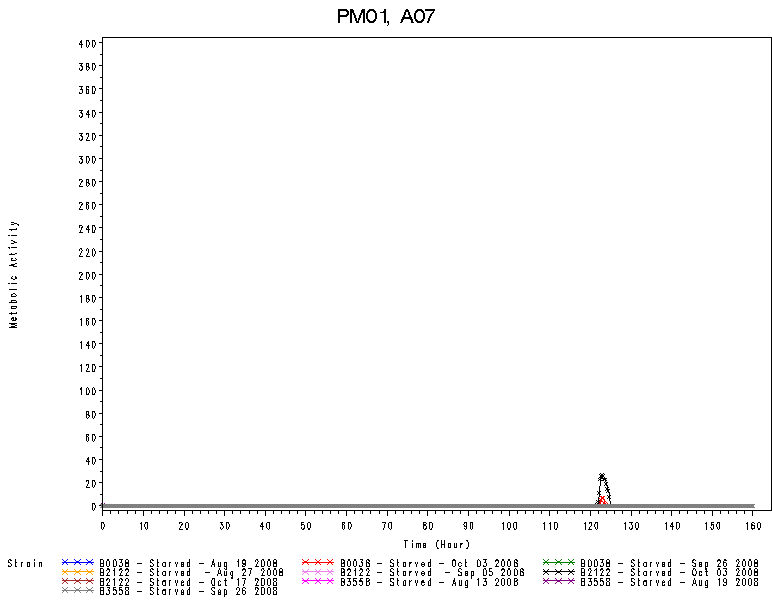

Supplement: Figure S3 — Kinetic curves for all PM plates with Mycobacterium bovis Type 9 strains. (ZIP) [file pone.0052673.s003.zip › suppl fig 3G type 9/Plate01/pm01a071.gif]

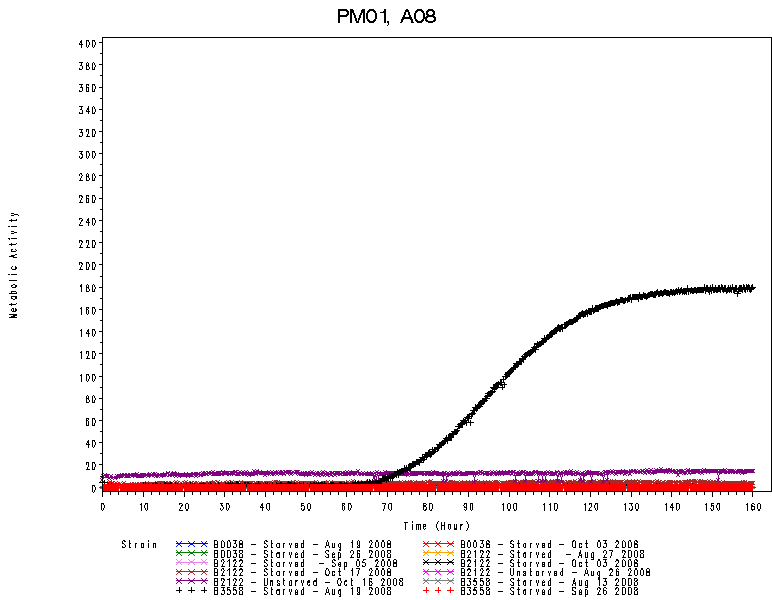

Supplement: Figure S3 — Kinetic curves for all PM plates with Mycobacterium bovis Type 9 strains. (ZIP) [file pone.0052673.s003.zip › suppl fig 3G type 9/Plate01/pm01a08.gif]

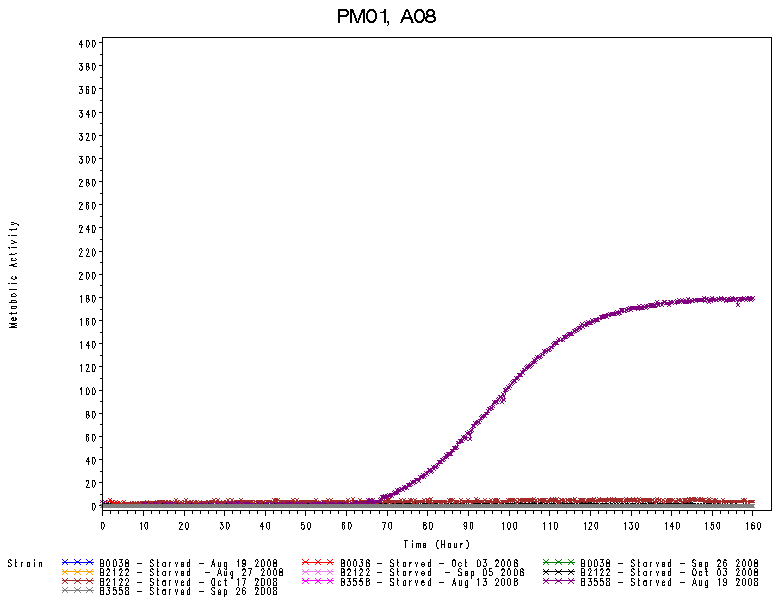

Supplement: Figure S3 — Kinetic curves for all PM plates with Mycobacterium bovis Type 9 strains. (ZIP) [file pone.0052673.s003.zip › suppl fig 3G type 9/Plate01/pm01a081.gif]

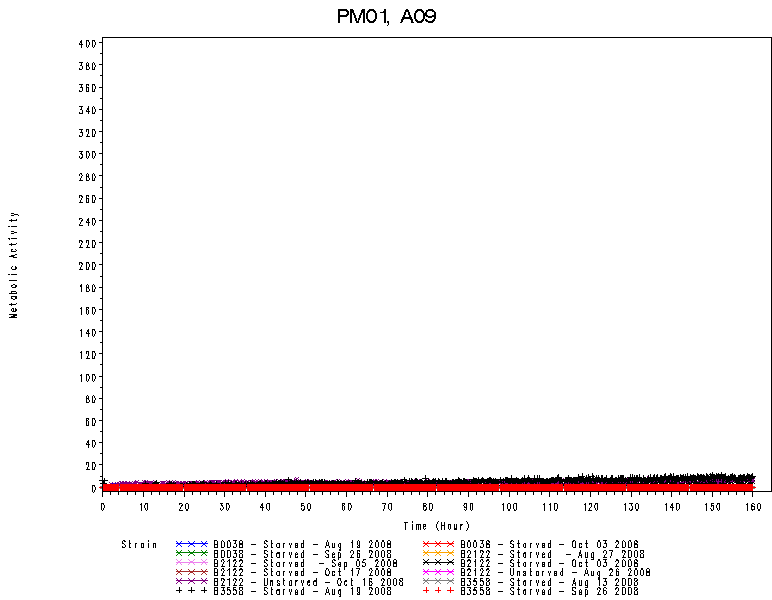

Supplement: Figure S3 — Kinetic curves for all PM plates with Mycobacterium bovis Type 9 strains. (ZIP) [file pone.0052673.s003.zip › suppl fig 3G type 9/Plate01/pm01a09.gif]

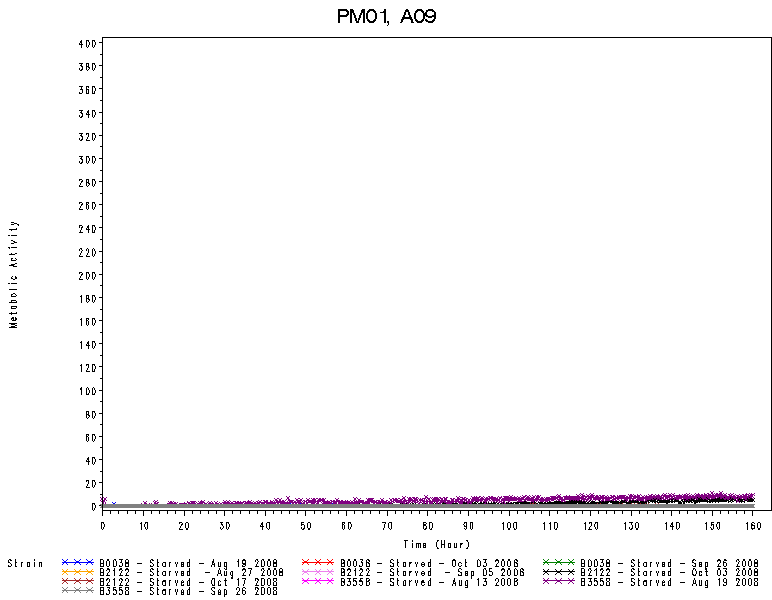

Supplement: Figure S3 — Kinetic curves for all PM plates with Mycobacterium bovis Type 9 strains. (ZIP) [file pone.0052673.s003.zip › suppl fig 3G type 9/Plate01/pm01a091.gif]

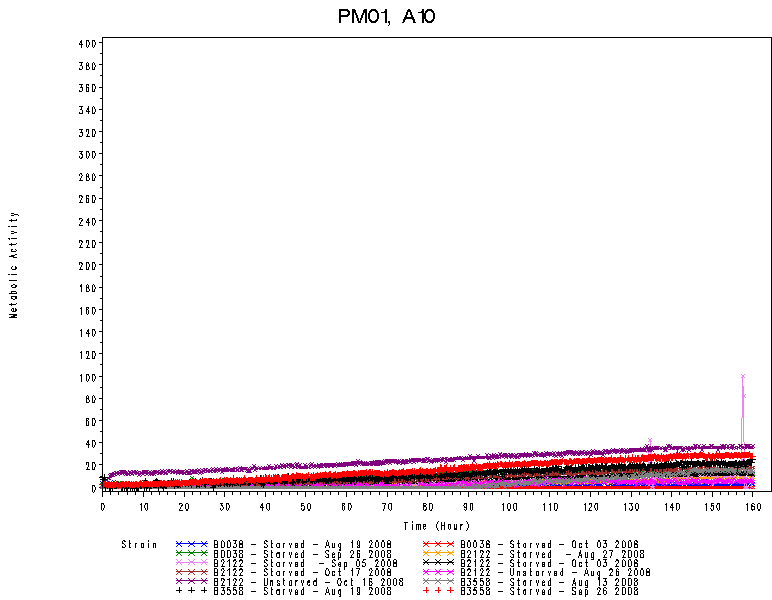

Supplement: Figure S3 — Kinetic curves for all PM plates with Mycobacterium bovis Type 9 strains. (ZIP) [file pone.0052673.s003.zip › suppl fig 3G type 9/Plate01/pm01a10.gif]

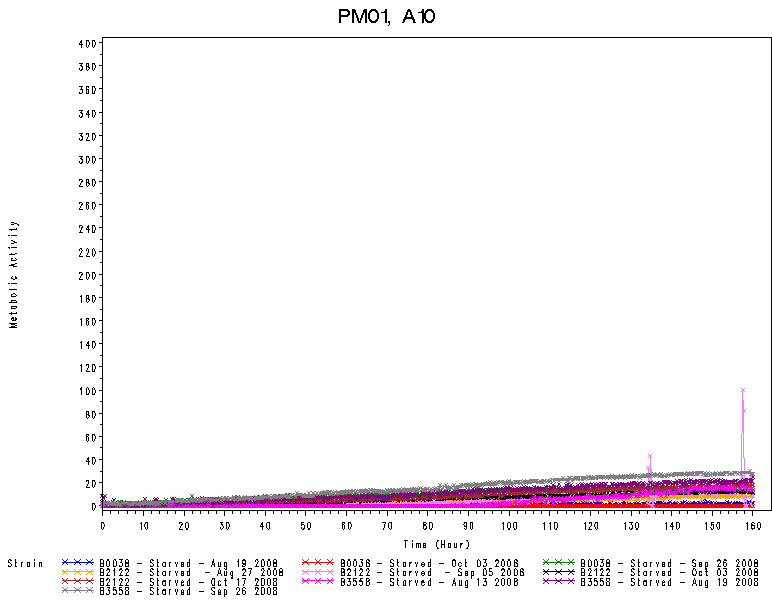

Supplement: Figure S3 — Kinetic curves for all PM plates with Mycobacterium bovis Type 9 strains. (ZIP) [file pone.0052673.s003.zip › suppl fig 3G type 9/Plate01/pm01a101.gif]

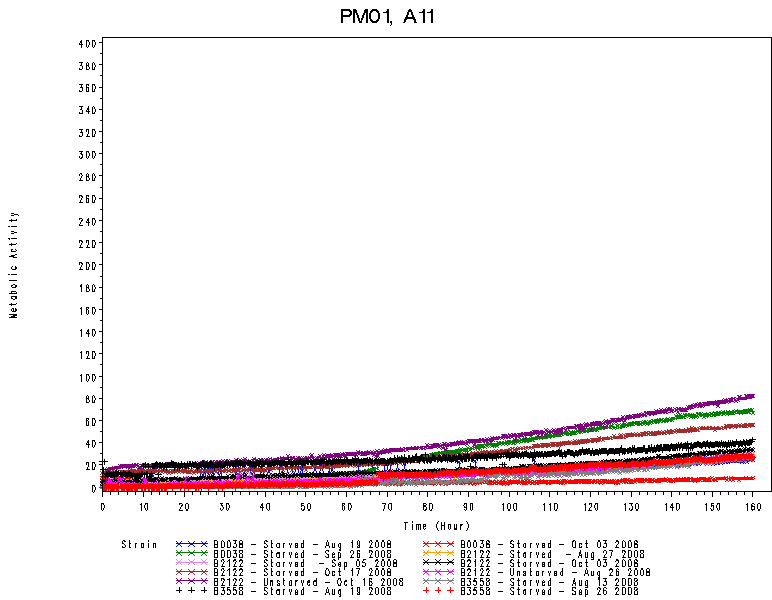

Supplement: Figure S3 — Kinetic curves for all PM plates with Mycobacterium bovis Type 9 strains. (ZIP) [file pone.0052673.s003.zip › suppl fig 3G type 9/Plate01/pm01a11.gif]

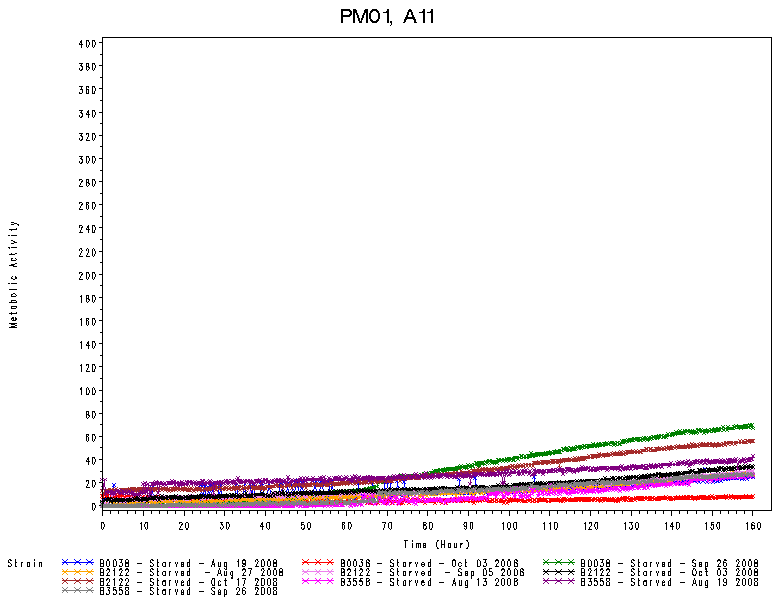

Supplement: Figure S3 — Kinetic curves for all PM plates with Mycobacterium bovis Type 9 strains. (ZIP) [file pone.0052673.s003.zip › suppl fig 3G type 9/Plate01/pm01a111.gif]

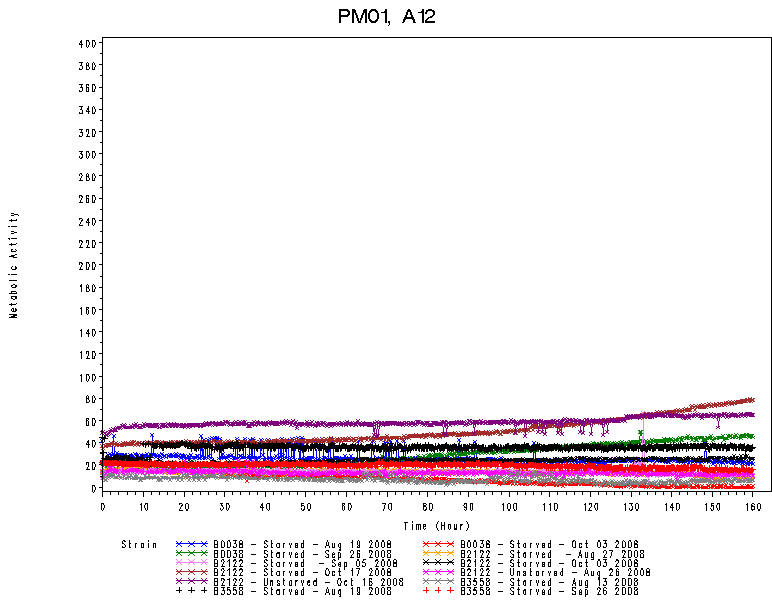

Supplement: Figure S3 — Kinetic curves for all PM plates with Mycobacterium bovis Type 9 strains. (ZIP) [file pone.0052673.s003.zip › suppl fig 3G type 9/Plate01/pm01a12.gif]

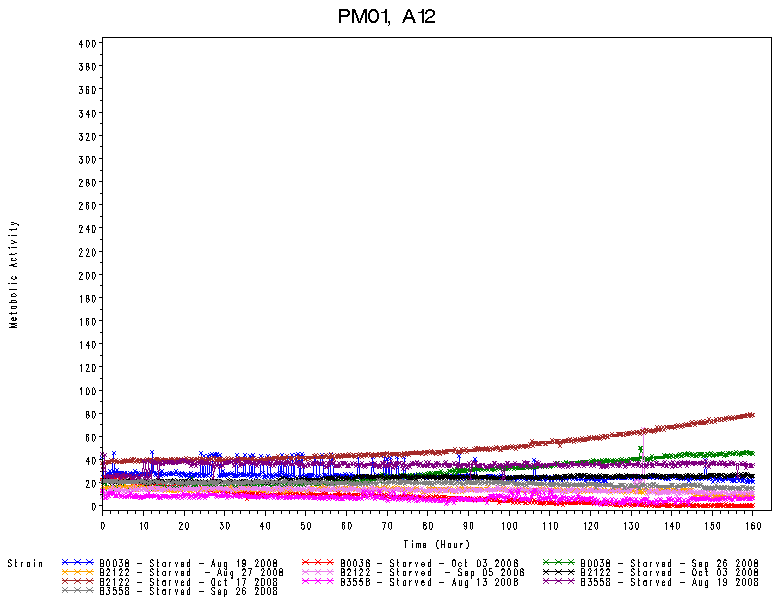

Supplement: Figure S3 — Kinetic curves for all PM plates with Mycobacterium bovis Type 9 strains. (ZIP) [file pone.0052673.s003.zip › suppl fig 3G type 9/Plate01/pm01a121.gif]

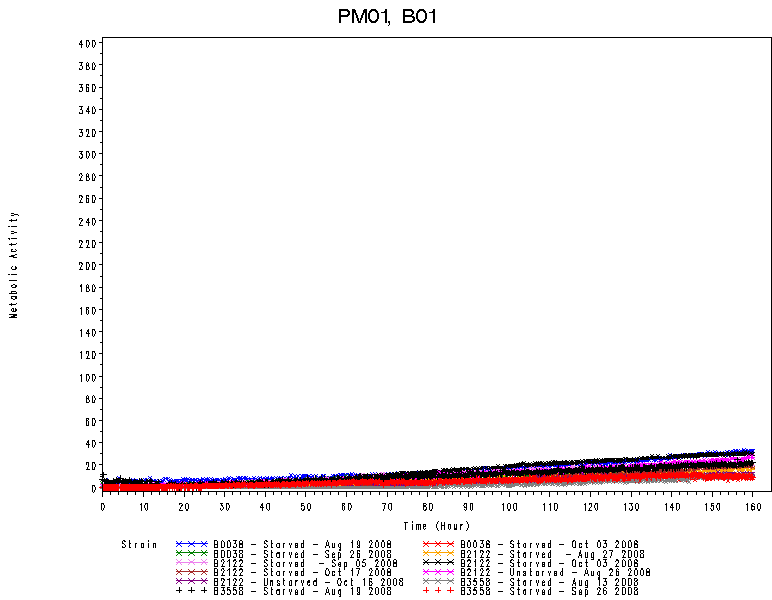

Supplement: Figure S3 — Kinetic curves for all PM plates with Mycobacterium bovis Type 9 strains. (ZIP) [file pone.0052673.s003.zip › suppl fig 3G type 9/Plate01/pm01b01.gif]

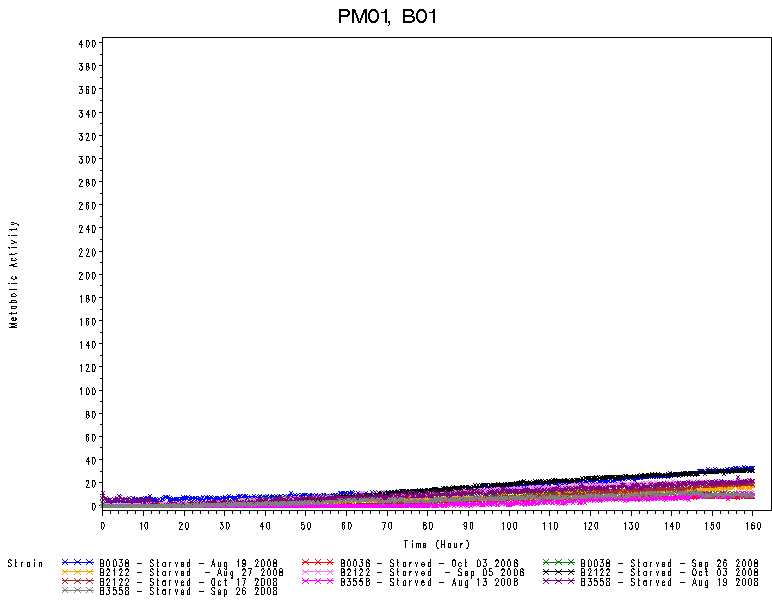

Supplement: Figure S3 — Kinetic curves for all PM plates with Mycobacterium bovis Type 9 strains. (ZIP) [file pone.0052673.s003.zip › suppl fig 3G type 9/Plate01/pm01b011.gif]

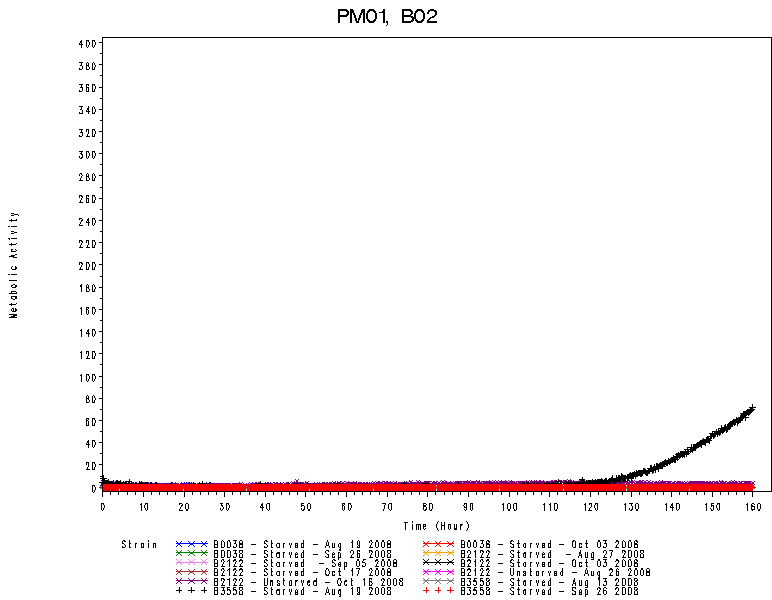

Supplement: Figure S3 — Kinetic curves for all PM plates with Mycobacterium bovis Type 9 strains. (ZIP) [file pone.0052673.s003.zip › suppl fig 3G type 9/Plate01/pm01b02.gif]

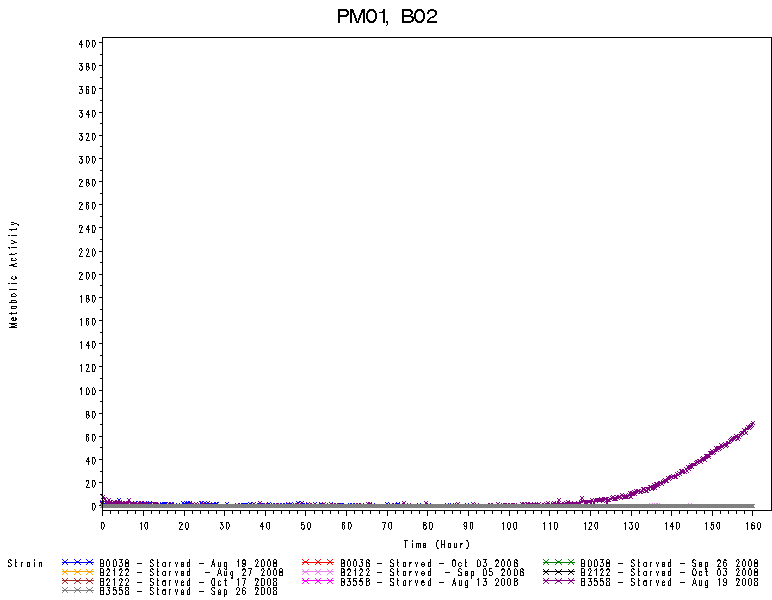

Supplement: Figure S3 — Kinetic curves for all PM plates with Mycobacterium bovis Type 9 strains. (ZIP) [file pone.0052673.s003.zip › suppl fig 3G type 9/Plate01/pm01b021.gif]

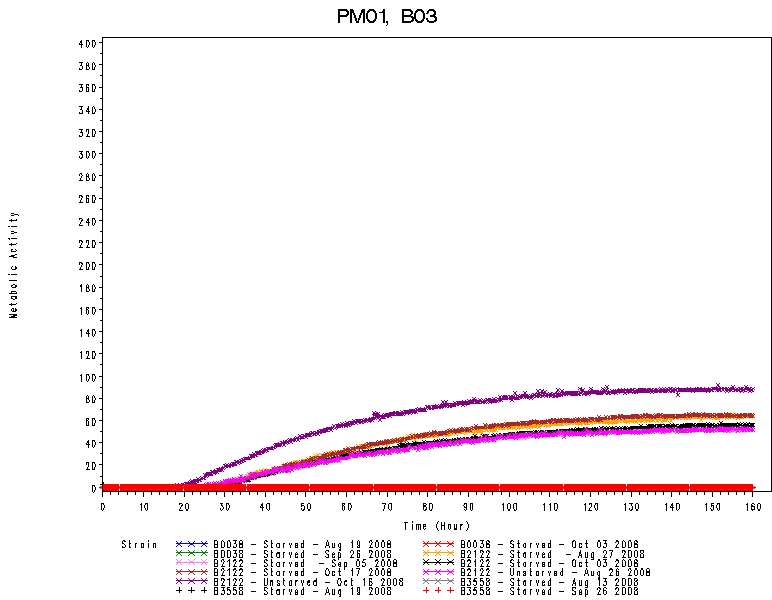

Supplement: Figure S3 — Kinetic curves for all PM plates with Mycobacterium bovis Type 9 strains. (ZIP) [file pone.0052673.s003.zip › suppl fig 3G type 9/Plate01/pm01b03.gif]

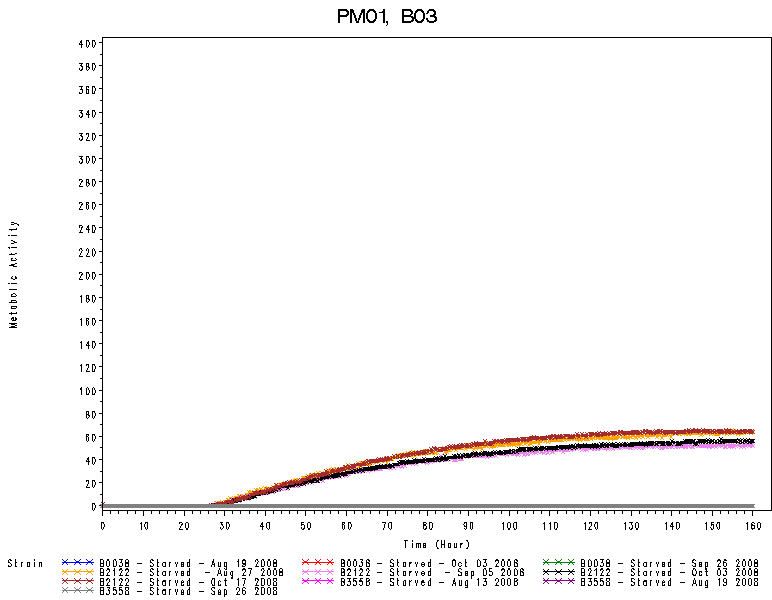

Supplement: Figure S3 — Kinetic curves for all PM plates with Mycobacterium bovis Type 9 strains. (ZIP) [file pone.0052673.s003.zip › suppl fig 3G type 9/Plate01/pm01b031.gif]

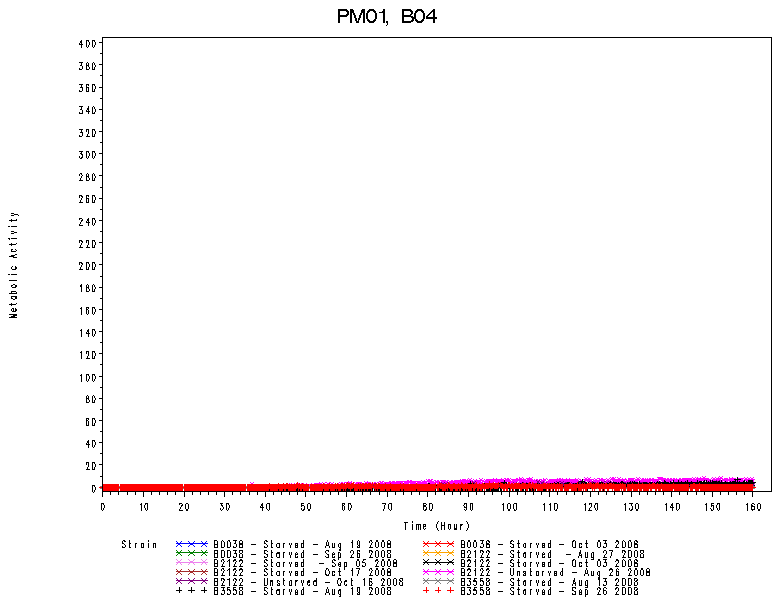

Supplement: Figure S3 — Kinetic curves for all PM plates with Mycobacterium bovis Type 9 strains. (ZIP) [file pone.0052673.s003.zip › suppl fig 3G type 9/Plate01/pm01b04.gif]

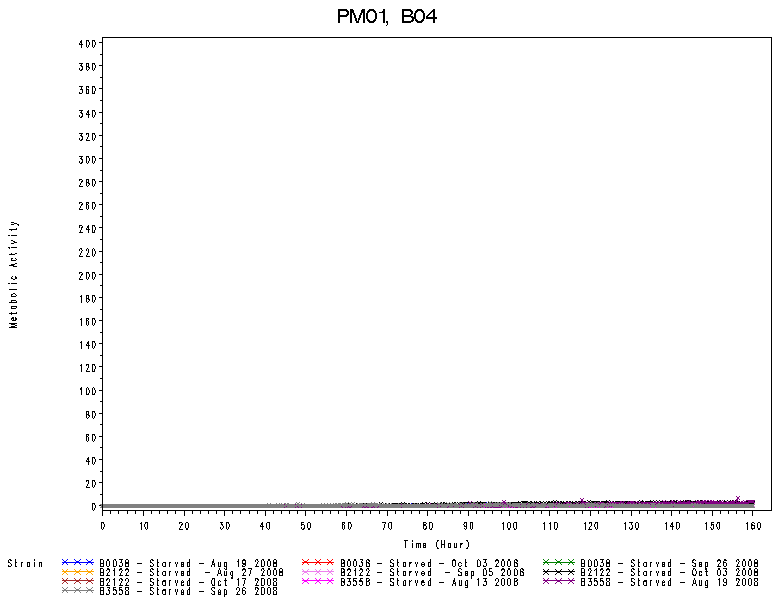

Supplement: Figure S3 — Kinetic curves for all PM plates with Mycobacterium bovis Type 9 strains. (ZIP) [file pone.0052673.s003.zip › suppl fig 3G type 9/Plate01/pm01b041.gif]

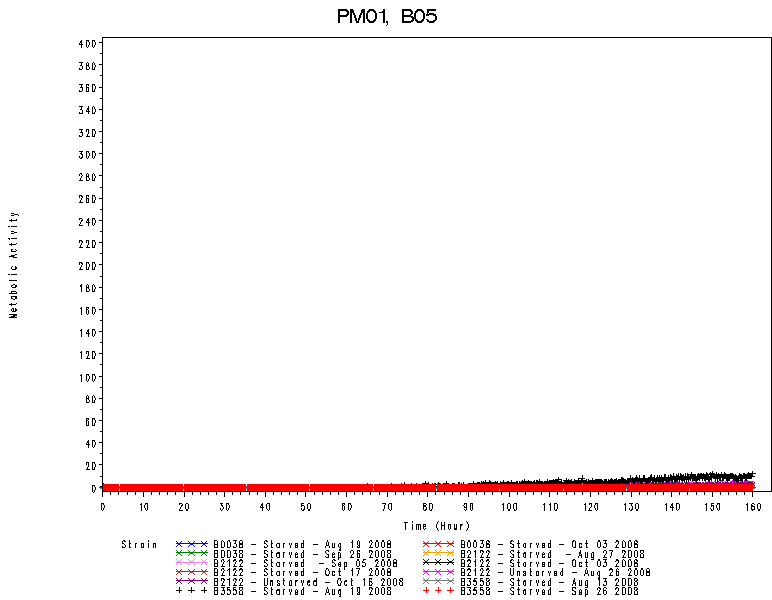

Supplement: Figure S3 — Kinetic curves for all PM plates with Mycobacterium bovis Type 9 strains. (ZIP) [file pone.0052673.s003.zip › suppl fig 3G type 9/Plate01/pm01b05.gif]

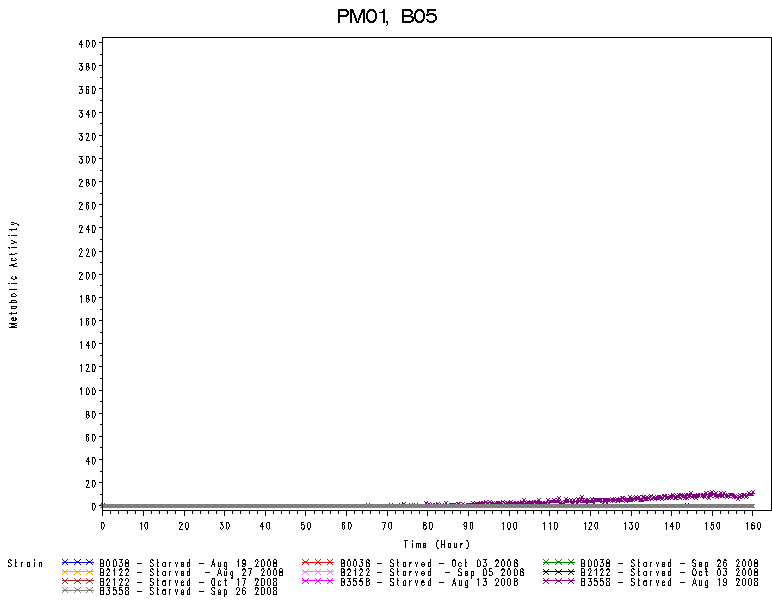

Supplement: Figure S3 — Kinetic curves for all PM plates with Mycobacterium bovis Type 9 strains. (ZIP) [file pone.0052673.s003.zip › suppl fig 3G type 9/Plate01/pm01b051.gif]

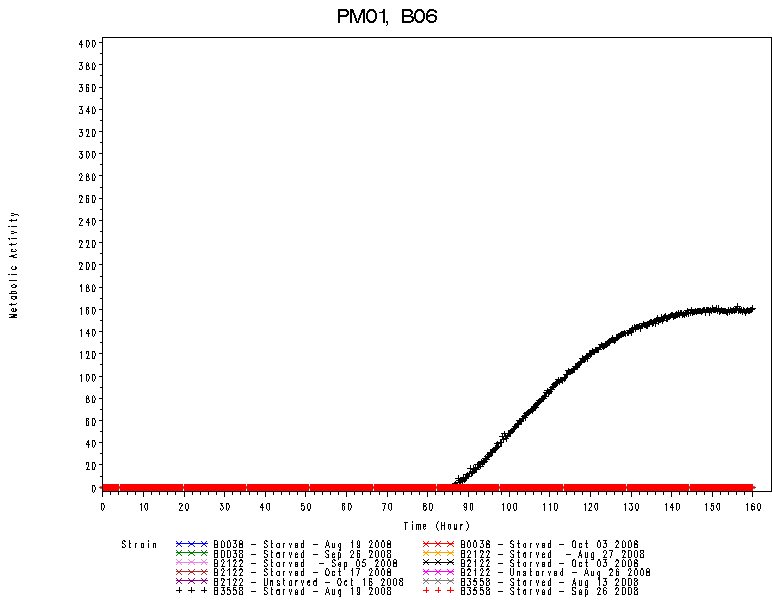

Supplement: Figure S3 — Kinetic curves for all PM plates with Mycobacterium bovis Type 9 strains. (ZIP) [file pone.0052673.s003.zip › suppl fig 3G type 9/Plate01/pm01b06.gif]

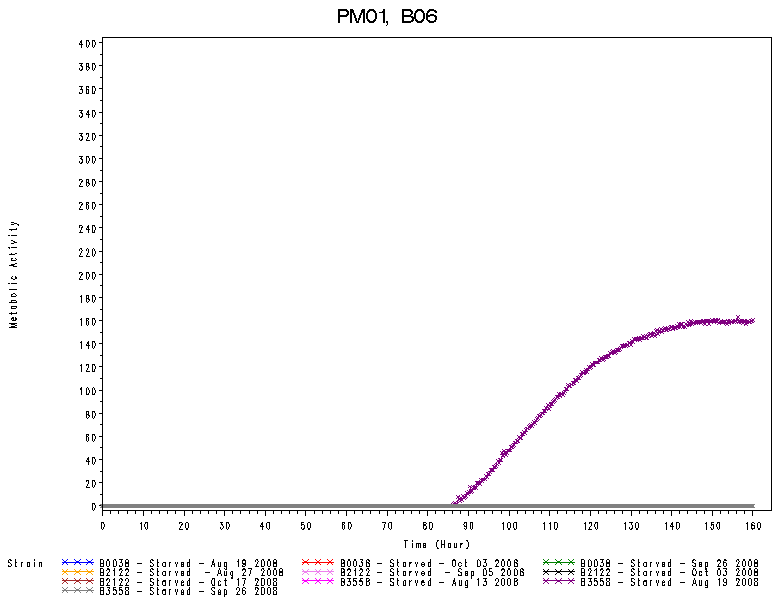

Supplement: Figure S3 — Kinetic curves for all PM plates with Mycobacterium bovis Type 9 strains. (ZIP) [file pone.0052673.s003.zip › suppl fig 3G type 9/Plate01/pm01b061.gif]

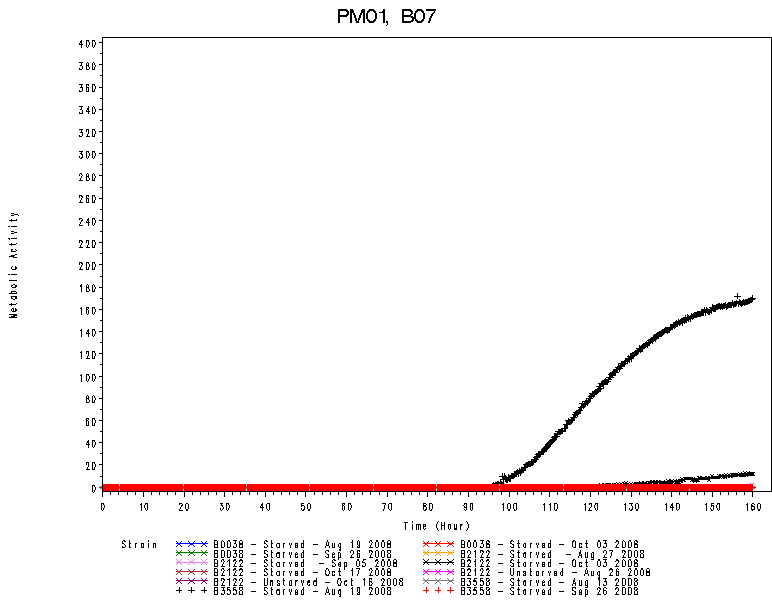

Supplement: Figure S3 — Kinetic curves for all PM plates with Mycobacterium bovis Type 9 strains. (ZIP) [file pone.0052673.s003.zip › suppl fig 3G type 9/Plate01/pm01b07.gif]

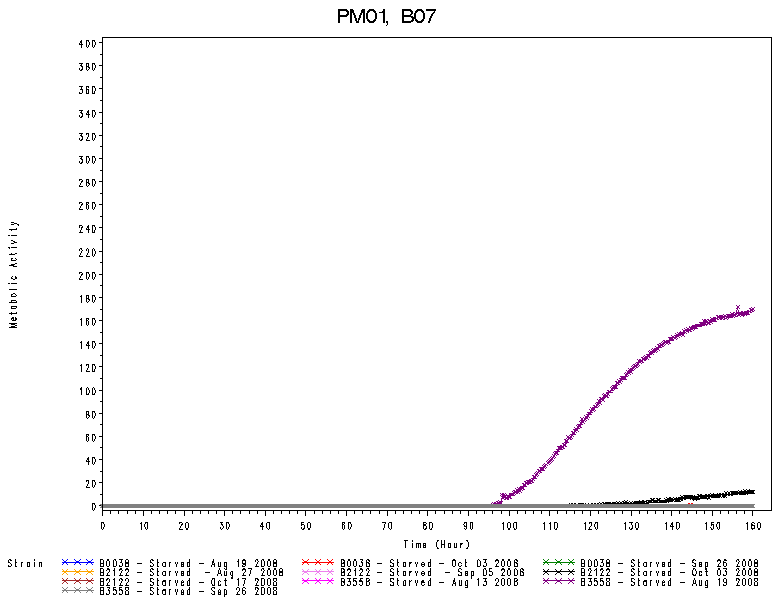

Supplement: Figure S3 — Kinetic curves for all PM plates with Mycobacterium bovis Type 9 strains. (ZIP) [file pone.0052673.s003.zip › suppl fig 3G type 9/Plate01/pm01b071.gif]

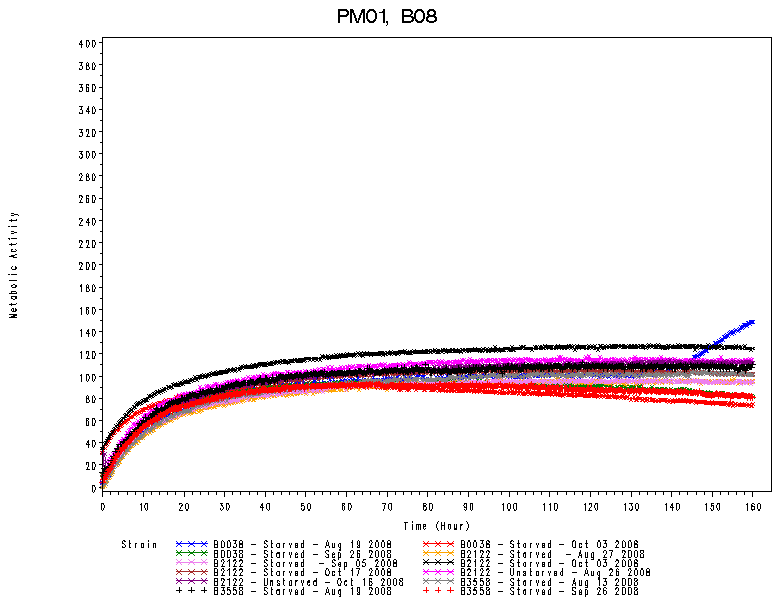

Supplement: Figure S3 — Kinetic curves for all PM plates with Mycobacterium bovis Type 9 strains. (ZIP) [file pone.0052673.s003.zip › suppl fig 3G type 9/Plate01/pm01b08.gif]

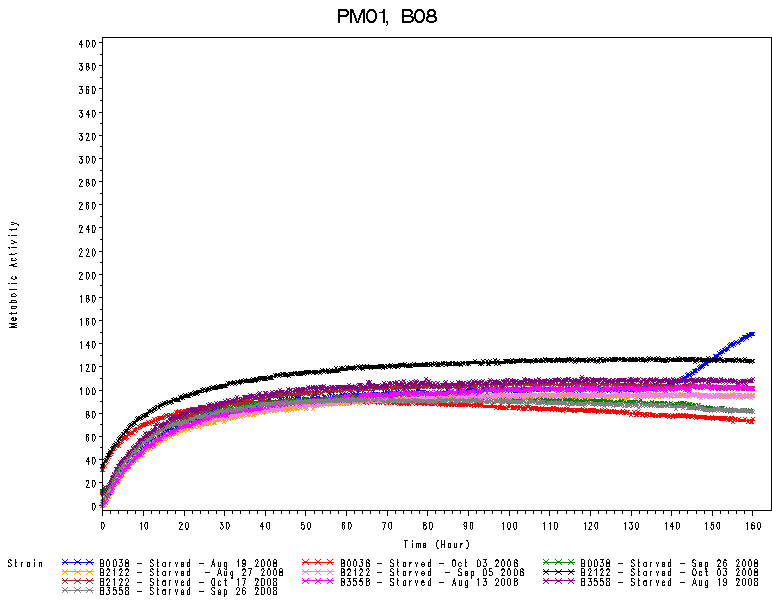

Supplement: Figure S3 — Kinetic curves for all PM plates with Mycobacterium bovis Type 9 strains. (ZIP) [file pone.0052673.s003.zip › suppl fig 3G type 9/Plate01/pm01b081.gif]

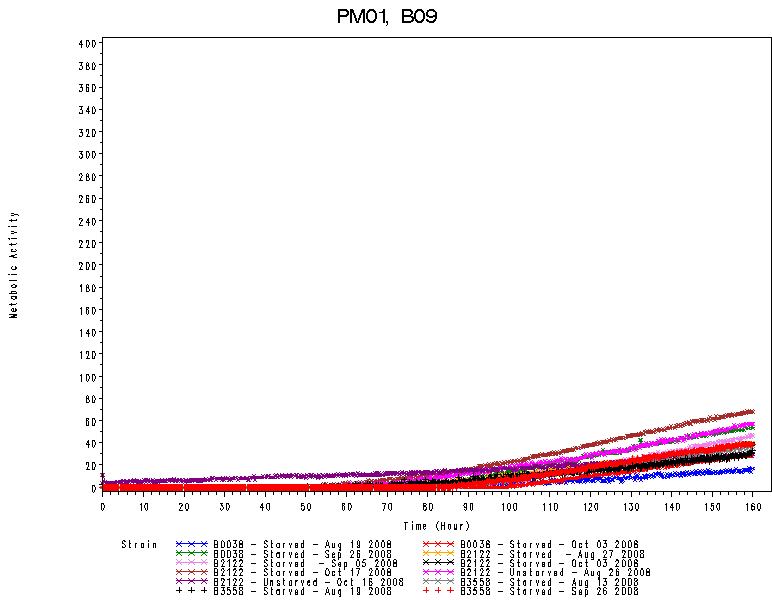

Supplement: Figure S3 — Kinetic curves for all PM plates with Mycobacterium bovis Type 9 strains. (ZIP) [file pone.0052673.s003.zip › suppl fig 3G type 9/Plate01/pm01b09.gif]

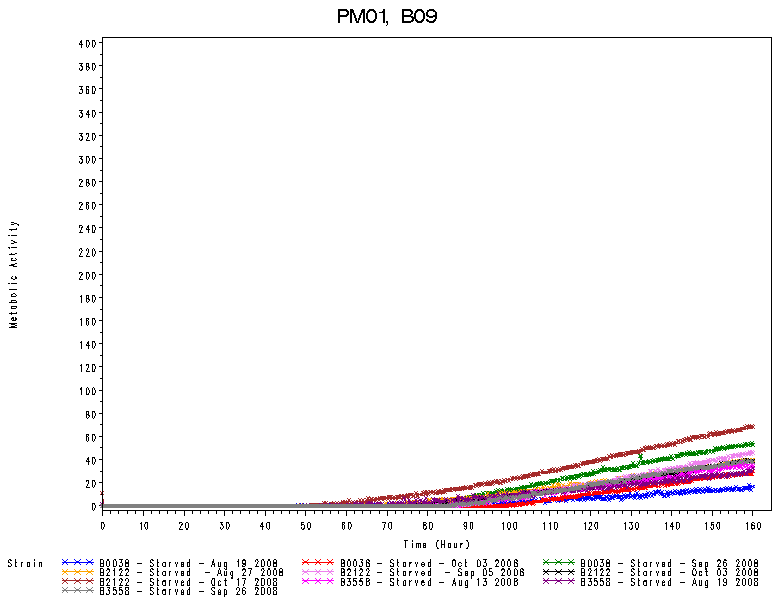

Supplement: Figure S3 — Kinetic curves for all PM plates with Mycobacterium bovis Type 9 strains. (ZIP) [file pone.0052673.s003.zip › suppl fig 3G type 9/Plate01/pm01b091.gif]

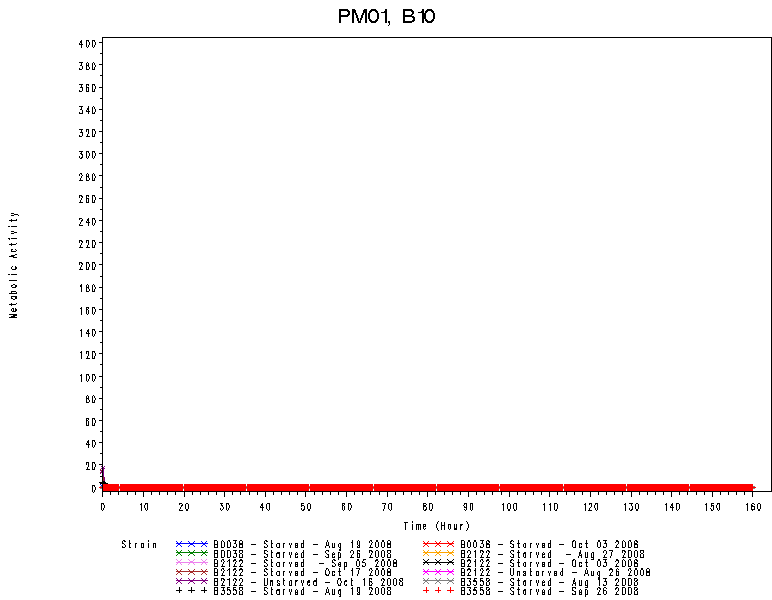

Supplement: Figure S3 — Kinetic curves for all PM plates with Mycobacterium bovis Type 9 strains. (ZIP) [file pone.0052673.s003.zip › suppl fig 3G type 9/Plate01/pm01b10.gif]

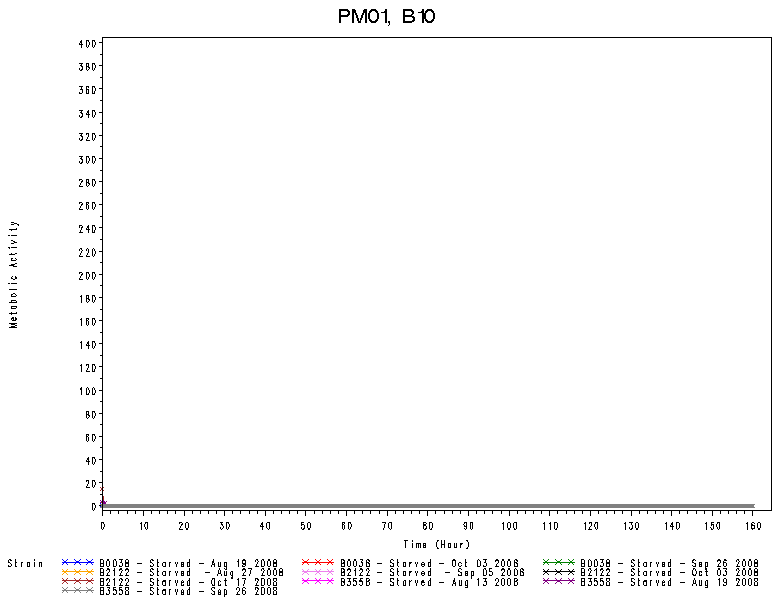

Supplement: Figure S3 — Kinetic curves for all PM plates with Mycobacterium bovis Type 9 strains. (ZIP) [file pone.0052673.s003.zip › suppl fig 3G type 9/Plate01/pm01b101.gif]

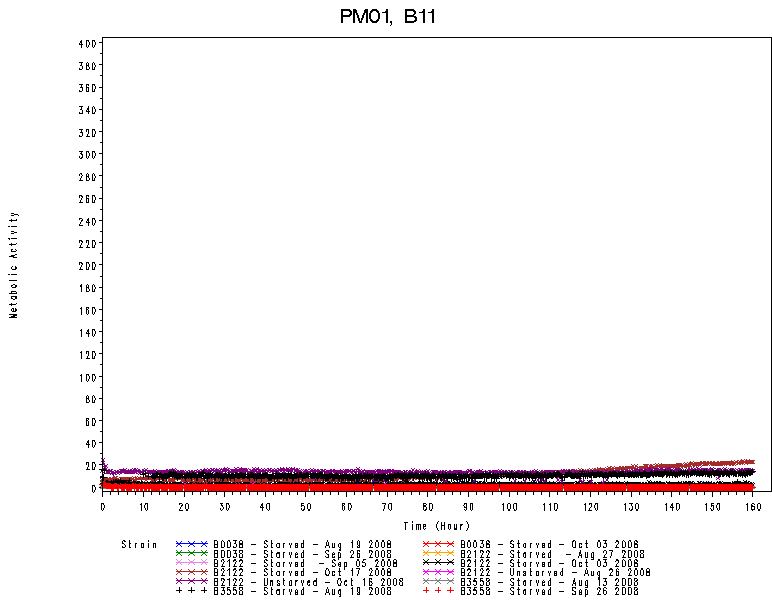

Supplement: Figure S3 — Kinetic curves for all PM plates with Mycobacterium bovis Type 9 strains. (ZIP) [file pone.0052673.s003.zip › suppl fig 3G type 9/Plate01/pm01b11.gif]

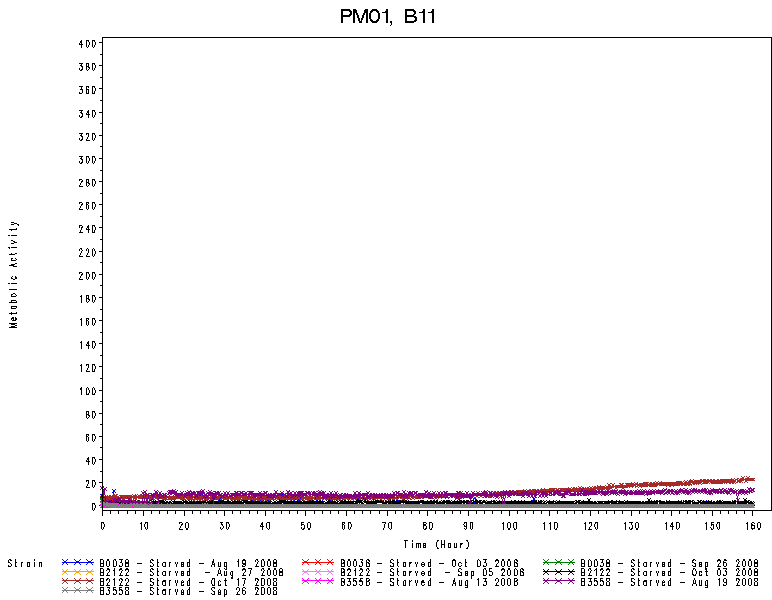

Supplement: Figure S3 — Kinetic curves for all PM plates with Mycobacterium bovis Type 9 strains. (ZIP) [file pone.0052673.s003.zip › suppl fig 3G type 9/Plate01/pm01b111.gif]

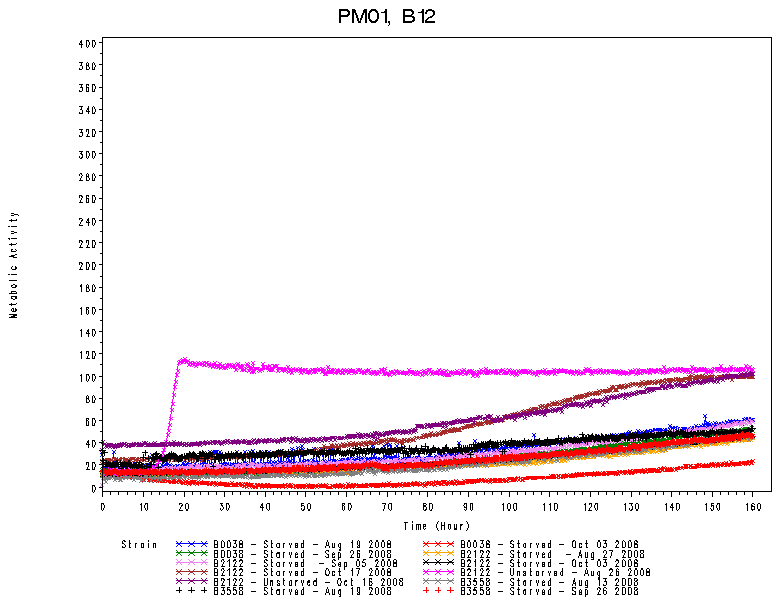

Supplement: Figure S3 — Kinetic curves for all PM plates with Mycobacterium bovis Type 9 strains. (ZIP) [file pone.0052673.s003.zip › suppl fig 3G type 9/Plate01/pm01b12.gif]

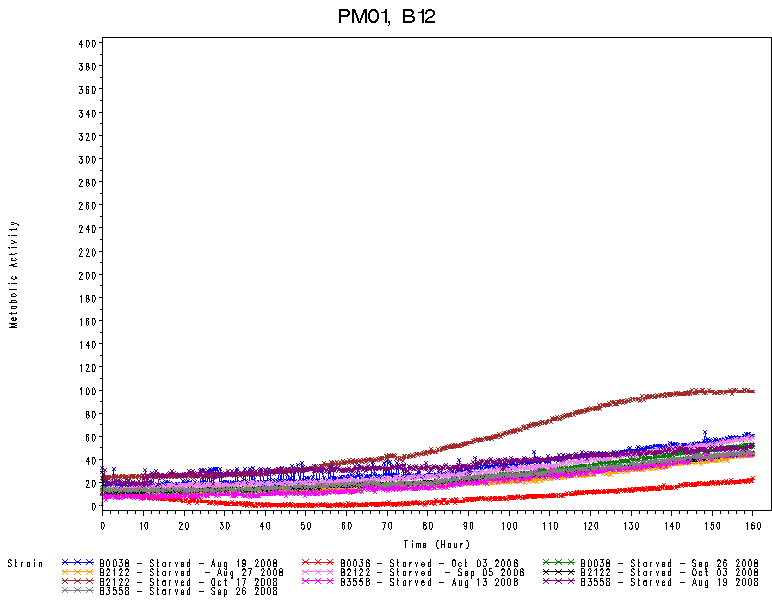

Supplement: Figure S3 — Kinetic curves for all PM plates with Mycobacterium bovis Type 9 strains. (ZIP) [file pone.0052673.s003.zip › suppl fig 3G type 9/Plate01/pm01b121.gif]

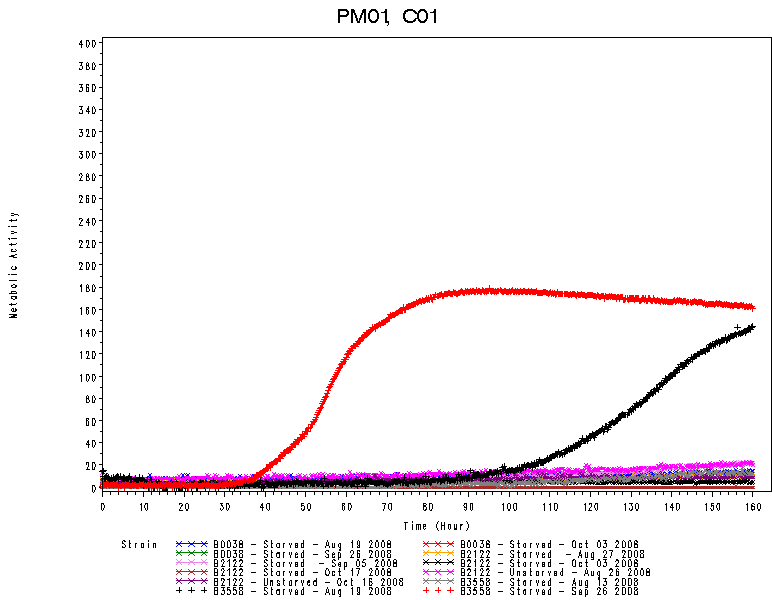

Supplement: Figure S3 — Kinetic curves for all PM plates with Mycobacterium bovis Type 9 strains. (ZIP) [file pone.0052673.s003.zip › suppl fig 3G type 9/Plate01/pm01c01.gif]

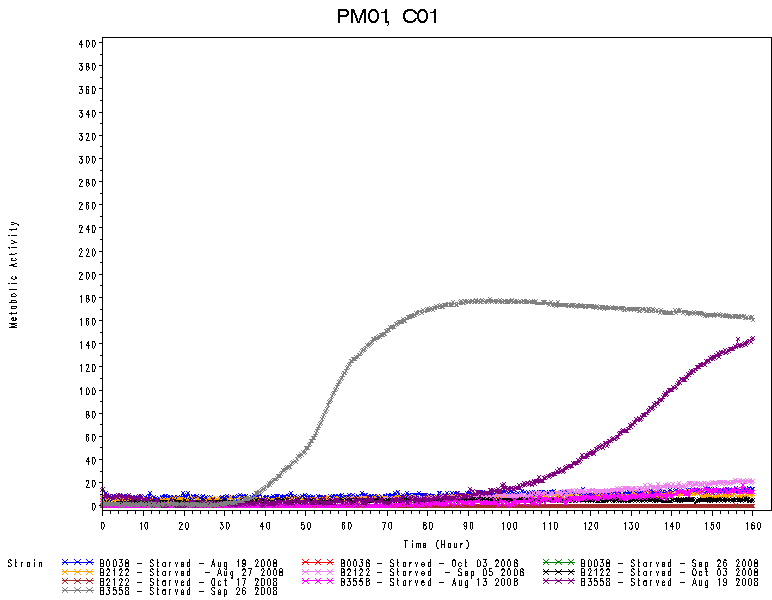

Supplement: Figure S3 — Kinetic curves for all PM plates with Mycobacterium bovis Type 9 strains. (ZIP) [file pone.0052673.s003.zip › suppl fig 3G type 9/Plate01/pm01c011.gif]

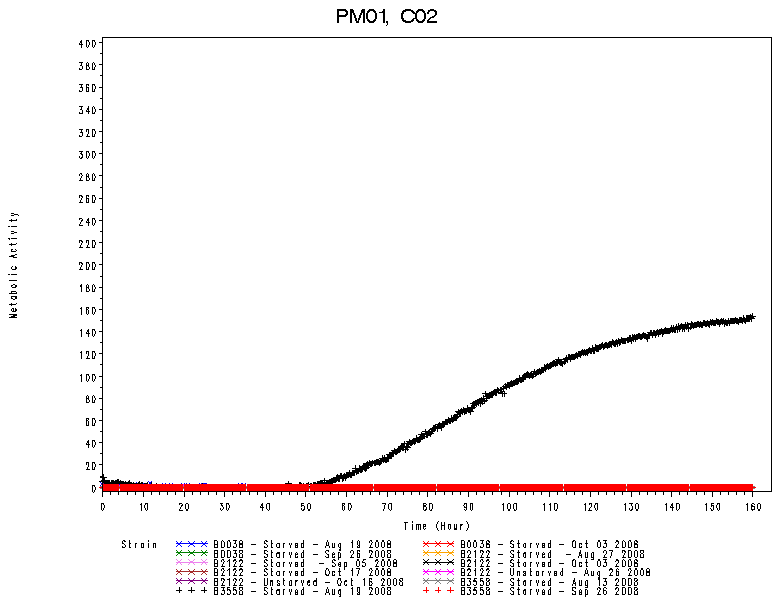

Supplement: Figure S3 — Kinetic curves for all PM plates with Mycobacterium bovis Type 9 strains. (ZIP) [file pone.0052673.s003.zip › suppl fig 3G type 9/Plate01/pm01c02.gif]

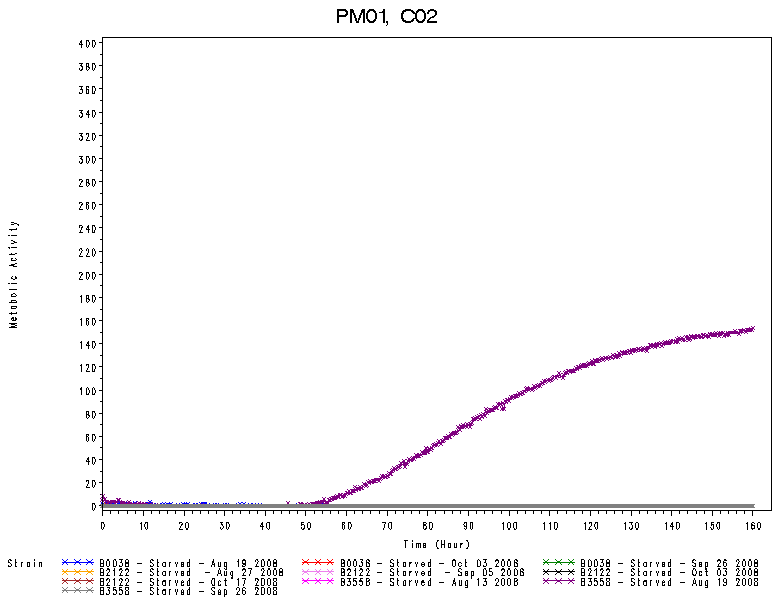

Supplement: Figure S3 — Kinetic curves for all PM plates with Mycobacterium bovis Type 9 strains. (ZIP) [file pone.0052673.s003.zip › suppl fig 3G type 9/Plate01/pm01c021.gif]

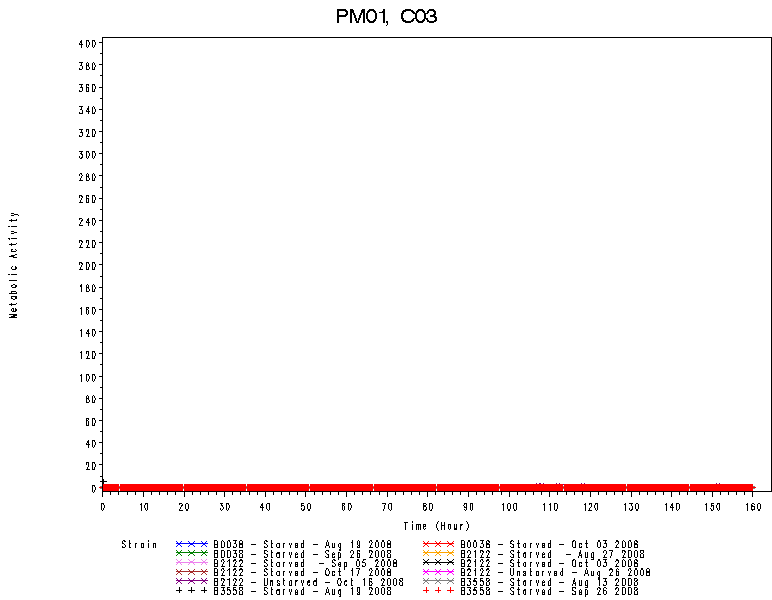

Supplement: Figure S3 — Kinetic curves for all PM plates with Mycobacterium bovis Type 9 strains. (ZIP) [file pone.0052673.s003.zip › suppl fig 3G type 9/Plate01/pm01c03.gif]

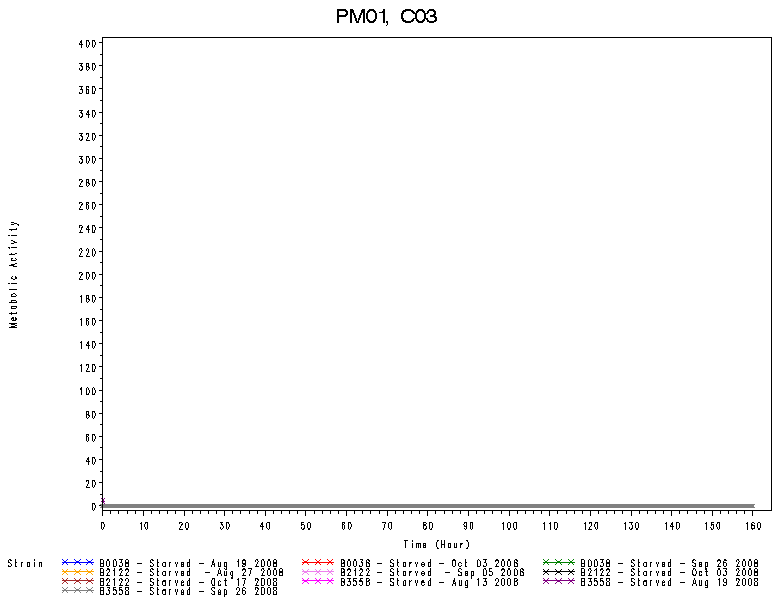

Supplement: Figure S3 — Kinetic curves for all PM plates with Mycobacterium bovis Type 9 strains. (ZIP) [file pone.0052673.s003.zip › suppl fig 3G type 9/Plate01/pm01c031.gif]

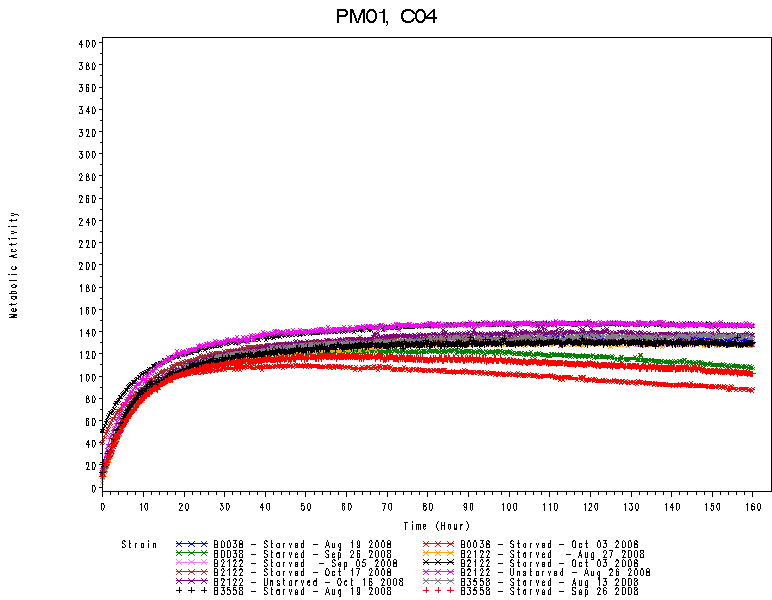

Supplement: Figure S3 — Kinetic curves for all PM plates with Mycobacterium bovis Type 9 strains. (ZIP) [file pone.0052673.s003.zip › suppl fig 3G type 9/Plate01/pm01c04.gif]

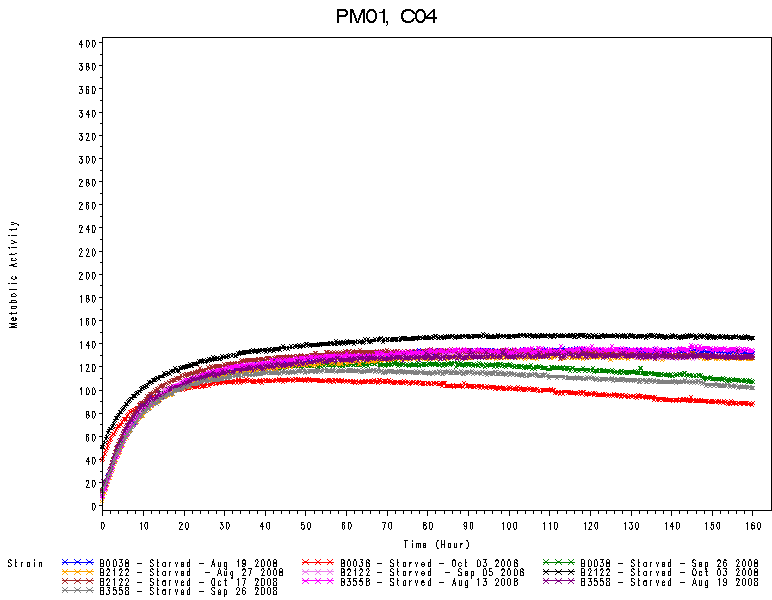

Supplement: Figure S3 — Kinetic curves for all PM plates with Mycobacterium bovis Type 9 strains. (ZIP) [file pone.0052673.s003.zip › suppl fig 3G type 9/Plate01/pm01c041.gif]

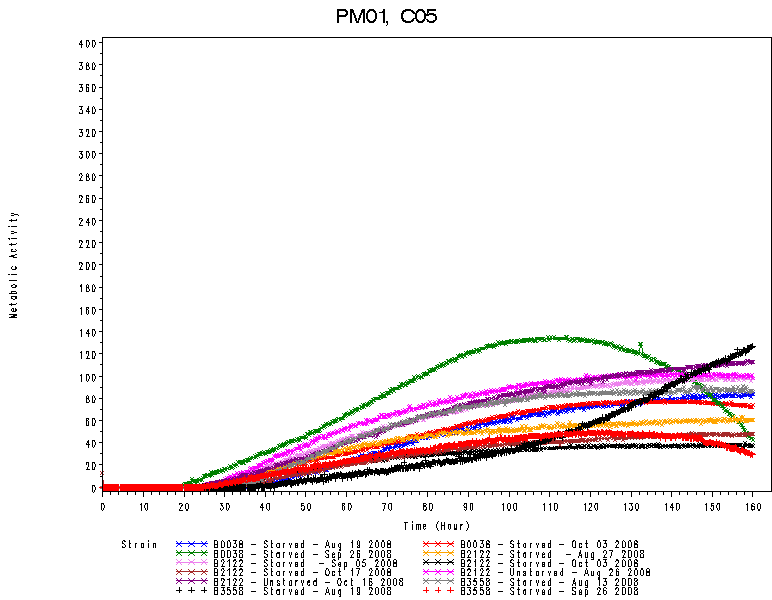

Supplement: Figure S3 — Kinetic curves for all PM plates with Mycobacterium bovis Type 9 strains. (ZIP) [file pone.0052673.s003.zip › suppl fig 3G type 9/Plate01/pm01c05.gif]

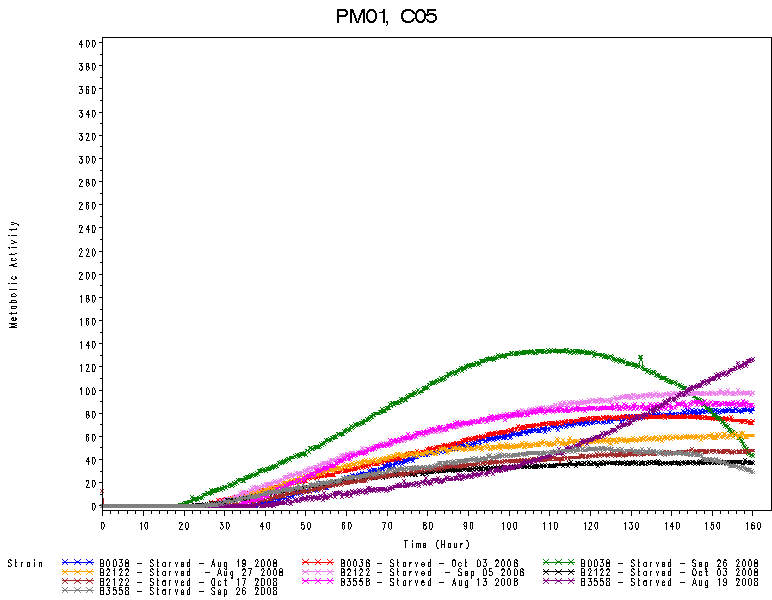

Supplement: Figure S3 — Kinetic curves for all PM plates with Mycobacterium bovis Type 9 strains. (ZIP) [file pone.0052673.s003.zip › suppl fig 3G type 9/Plate01/pm01c051.gif]

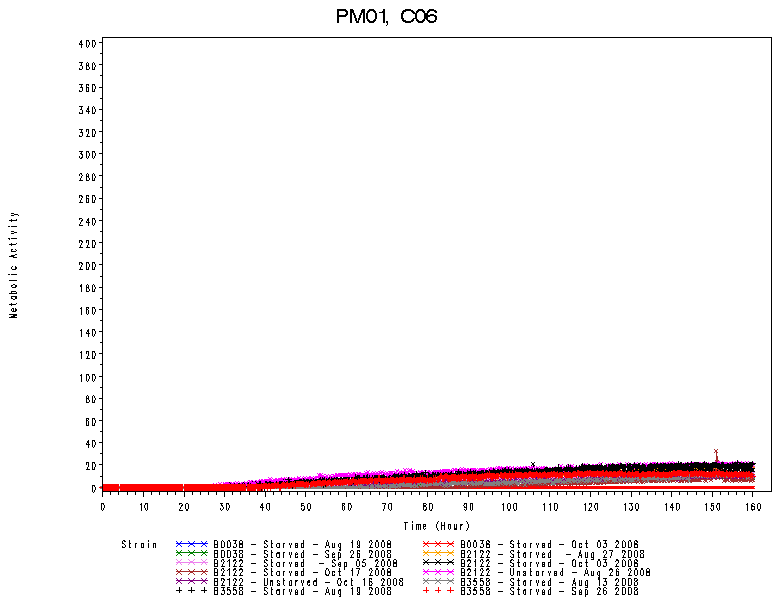

Supplement: Figure S3 — Kinetic curves for all PM plates with Mycobacterium bovis Type 9 strains. (ZIP) [file pone.0052673.s003.zip › suppl fig 3G type 9/Plate01/pm01c06.gif]

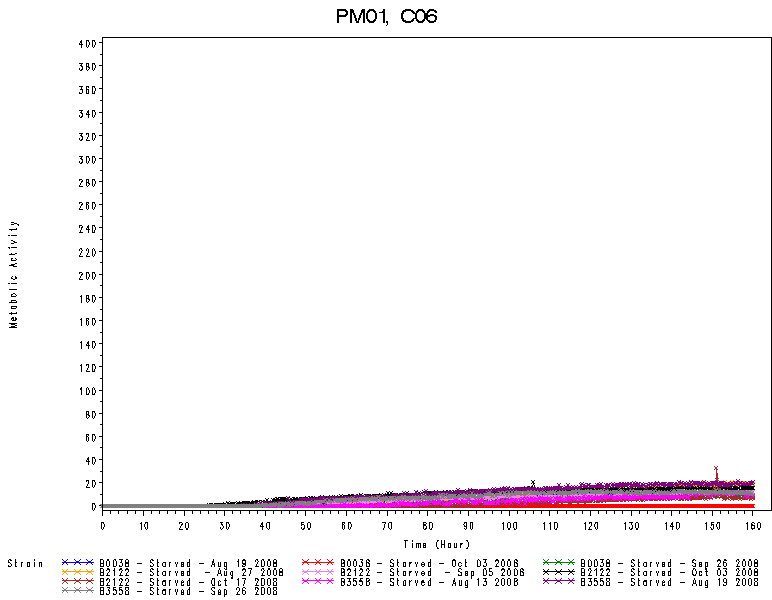

Supplement: Figure S3 — Kinetic curves for all PM plates with Mycobacterium bovis Type 9 strains. (ZIP) [file pone.0052673.s003.zip › suppl fig 3G type 9/Plate01/pm01c061.gif]

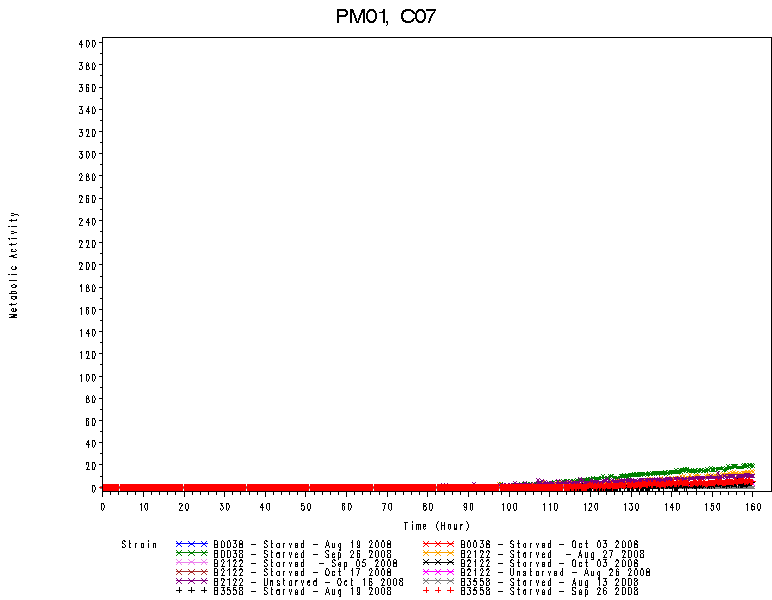

Supplement: Figure S3 — Kinetic curves for all PM plates with Mycobacterium bovis Type 9 strains. (ZIP) [file pone.0052673.s003.zip › suppl fig 3G type 9/Plate01/pm01c07.gif]

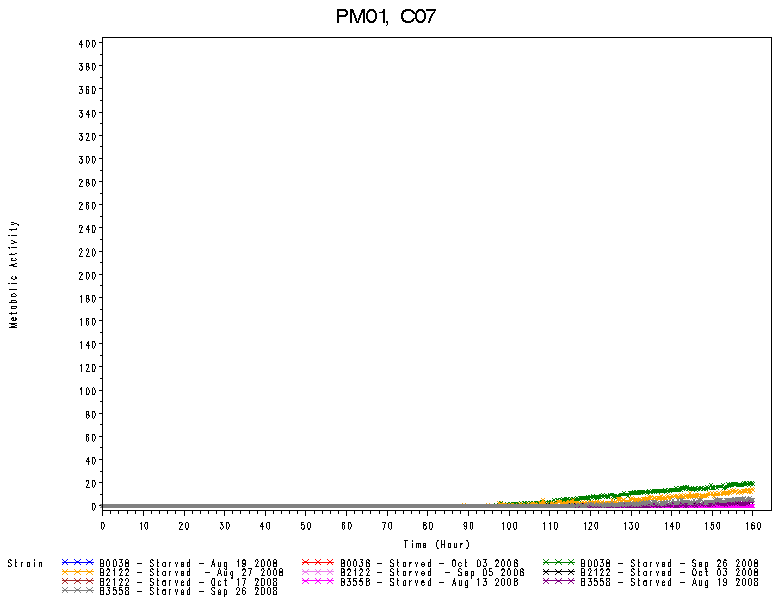

Supplement: Figure S3 — Kinetic curves for all PM plates with Mycobacterium bovis Type 9 strains. (ZIP) [file pone.0052673.s003.zip › suppl fig 3G type 9/Plate01/pm01c071.gif]

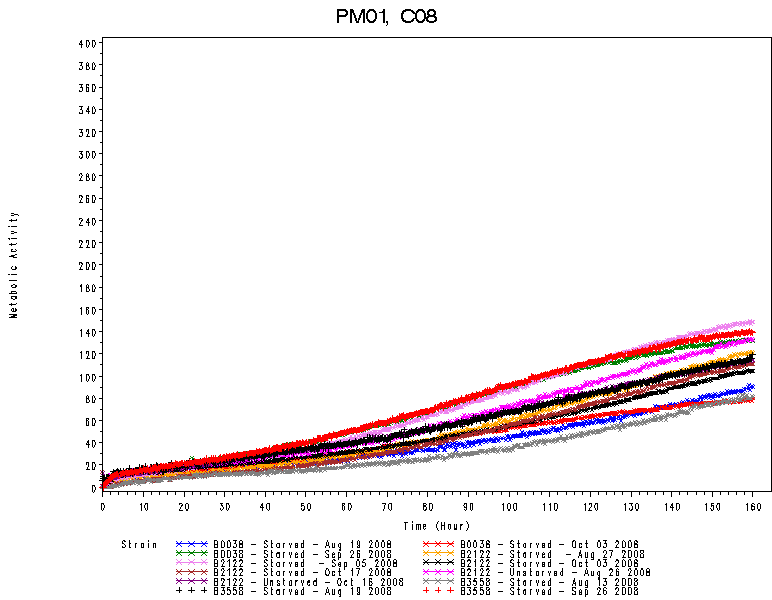

Supplement: Figure S3 — Kinetic curves for all PM plates with Mycobacterium bovis Type 9 strains. (ZIP) [file pone.0052673.s003.zip › suppl fig 3G type 9/Plate01/pm01c08.gif]

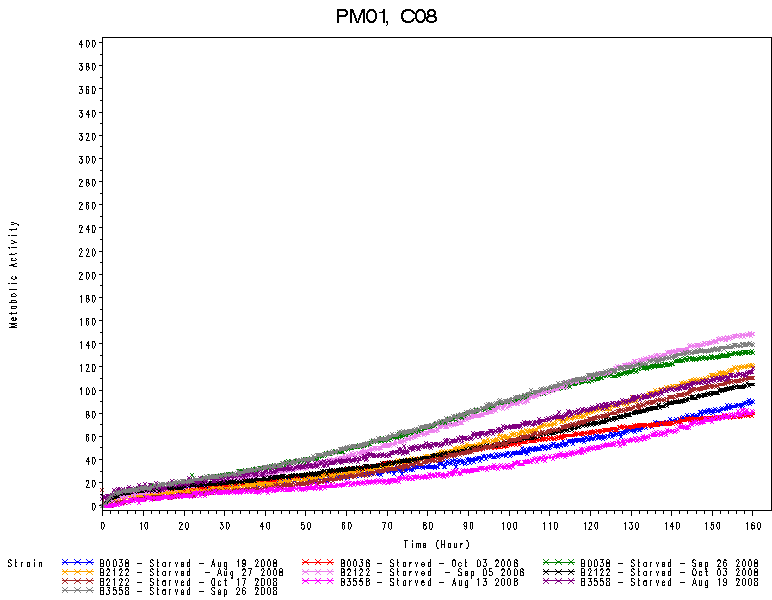

Supplement: Figure S3 — Kinetic curves for all PM plates with Mycobacterium bovis Type 9 strains. (ZIP) [file pone.0052673.s003.zip › suppl fig 3G type 9/Plate01/pm01c081.gif]

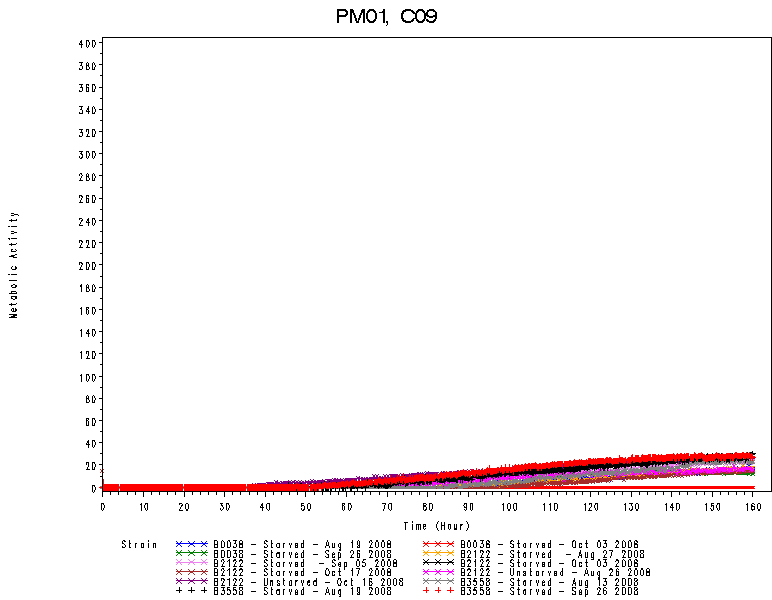

Supplement: Figure S3 — Kinetic curves for all PM plates with Mycobacterium bovis Type 9 strains. (ZIP) [file pone.0052673.s003.zip › suppl fig 3G type 9/Plate01/pm01c09.gif]

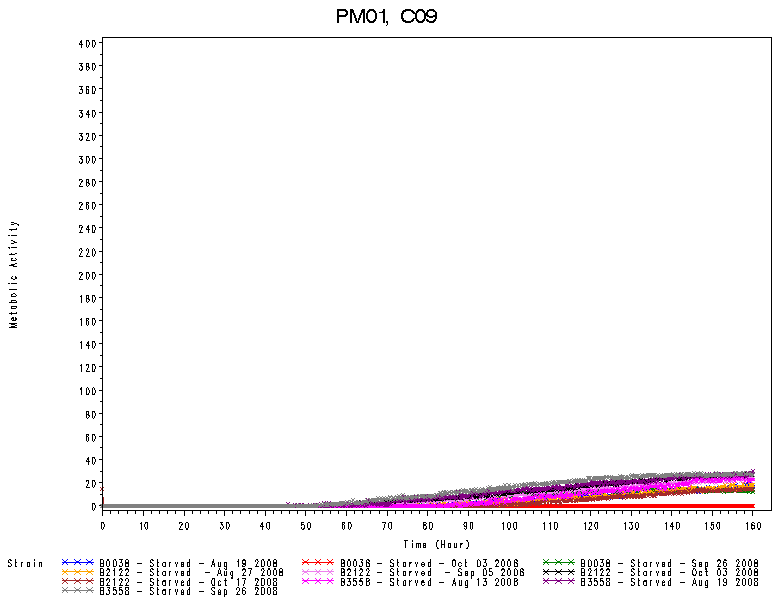

Supplement: Figure S3 — Kinetic curves for all PM plates with Mycobacterium bovis Type 9 strains. (ZIP) [file pone.0052673.s003.zip › suppl fig 3G type 9/Plate01/pm01c091.gif]

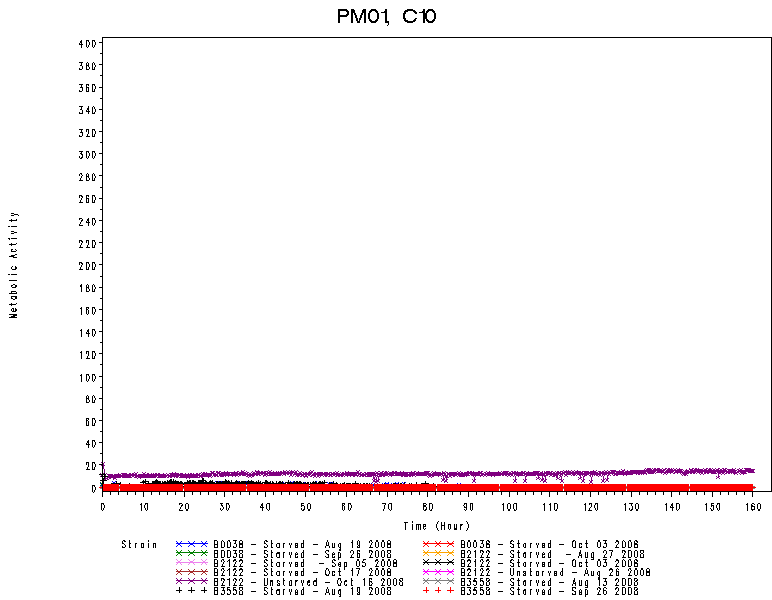

Supplement: Figure S3 — Kinetic curves for all PM plates with Mycobacterium bovis Type 9 strains. (ZIP) [file pone.0052673.s003.zip › suppl fig 3G type 9/Plate01/pm01c10.gif]

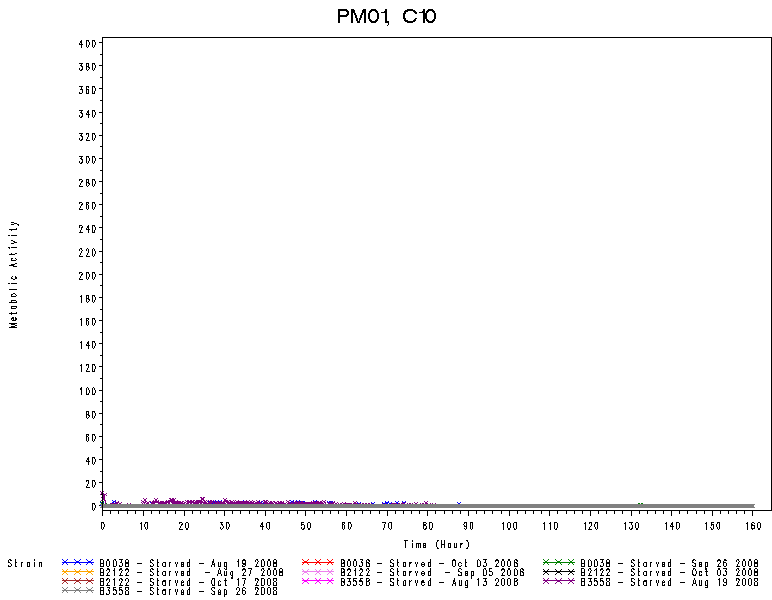

Supplement: Figure S3 — Kinetic curves for all PM plates with Mycobacterium bovis Type 9 strains. (ZIP) [file pone.0052673.s003.zip › suppl fig 3G type 9/Plate01/pm01c101.gif]

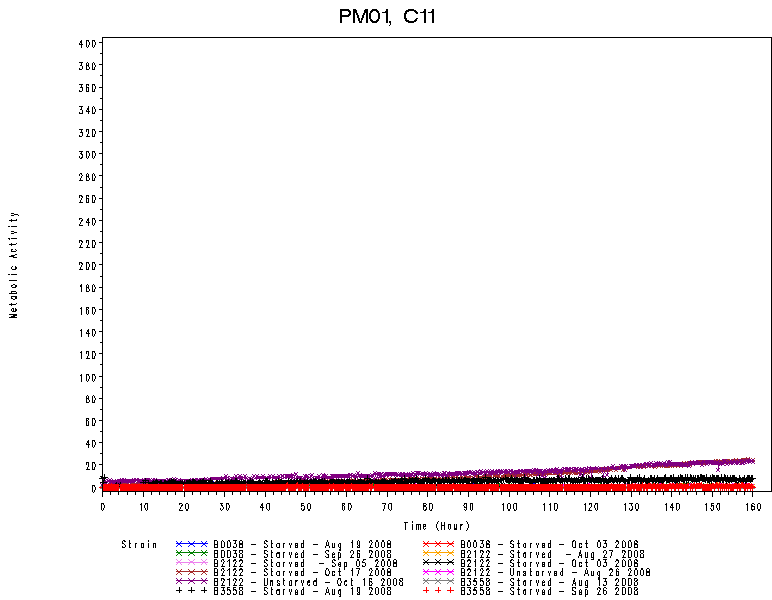

Supplement: Figure S3 — Kinetic curves for all PM plates with Mycobacterium bovis Type 9 strains. (ZIP) [file pone.0052673.s003.zip › suppl fig 3G type 9/Plate01/pm01c11.gif]

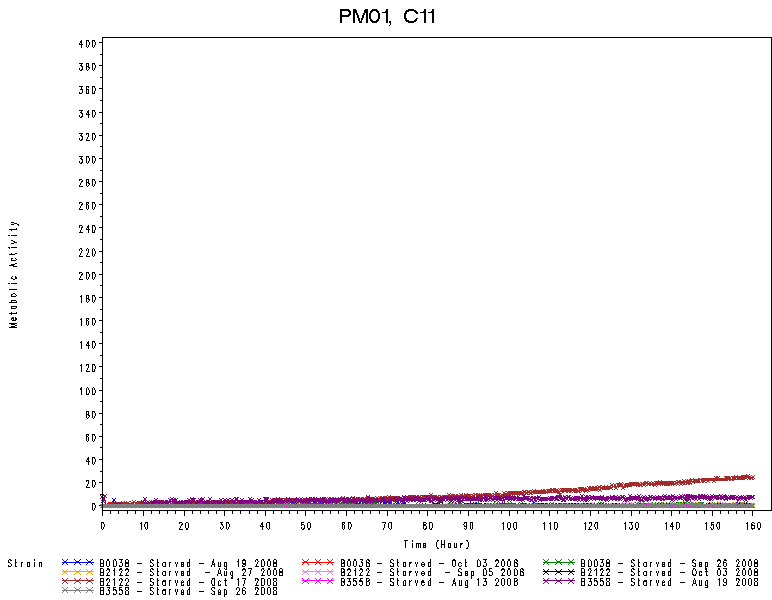

Supplement: Figure S3 — Kinetic curves for all PM plates with Mycobacterium bovis Type 9 strains. (ZIP) [file pone.0052673.s003.zip › suppl fig 3G type 9/Plate01/pm01c111.gif]

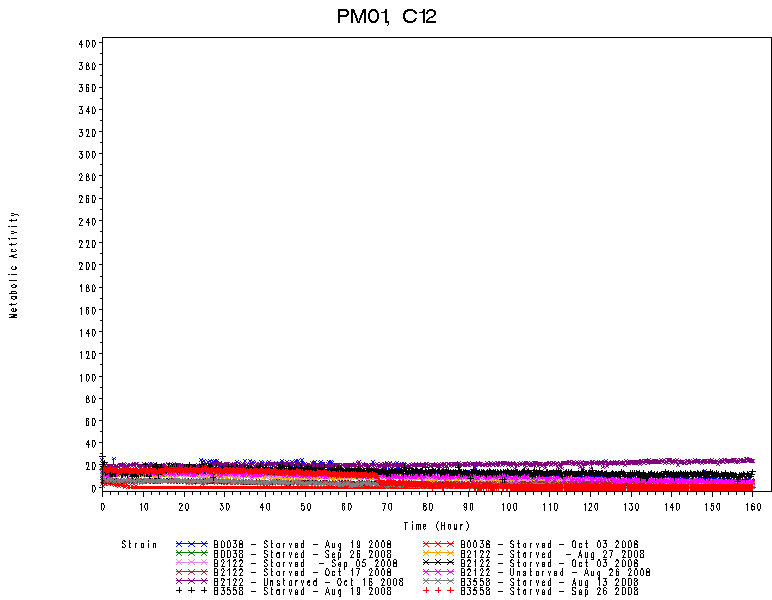

Supplement: Figure S3 — Kinetic curves for all PM plates with Mycobacterium bovis Type 9 strains. (ZIP) [file pone.0052673.s003.zip › suppl fig 3G type 9/Plate01/pm01c12.gif]

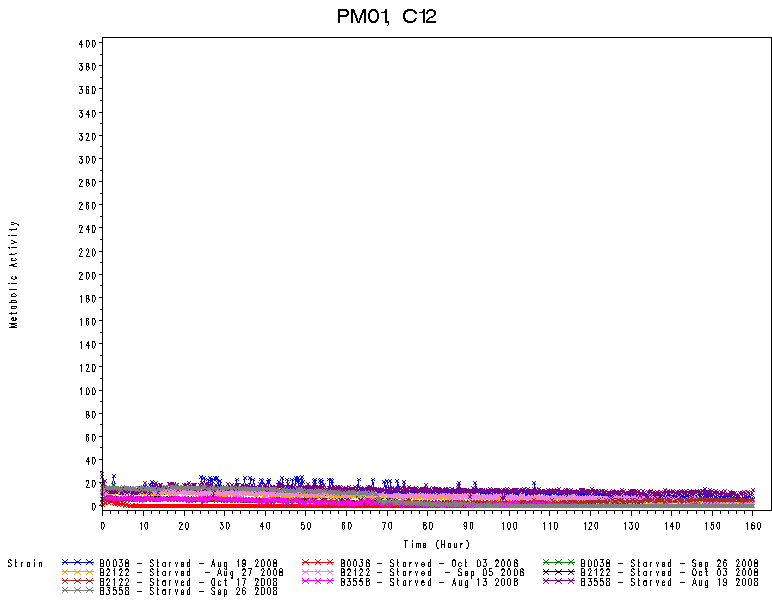

Supplement: Figure S3 — Kinetic curves for all PM plates with Mycobacterium bovis Type 9 strains. (ZIP) [file pone.0052673.s003.zip › suppl fig 3G type 9/Plate01/pm01c121.gif]

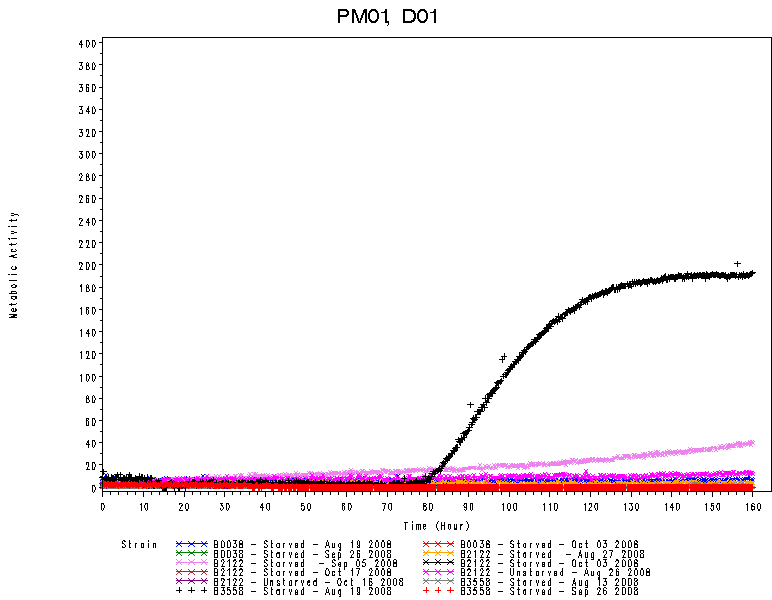

Supplement: Figure S3 — Kinetic curves for all PM plates with Mycobacterium bovis Type 9 strains. (ZIP) [file pone.0052673.s003.zip › suppl fig 3G type 9/Plate01/pm01d01.gif]

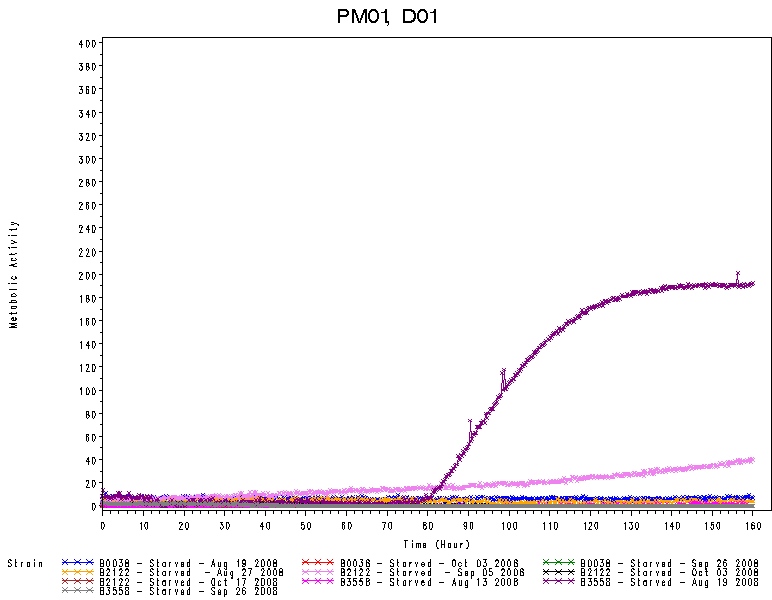

Supplement: Figure S3 — Kinetic curves for all PM plates with Mycobacterium bovis Type 9 strains. (ZIP) [file pone.0052673.s003.zip › suppl fig 3G type 9/Plate01/pm01d011.gif]

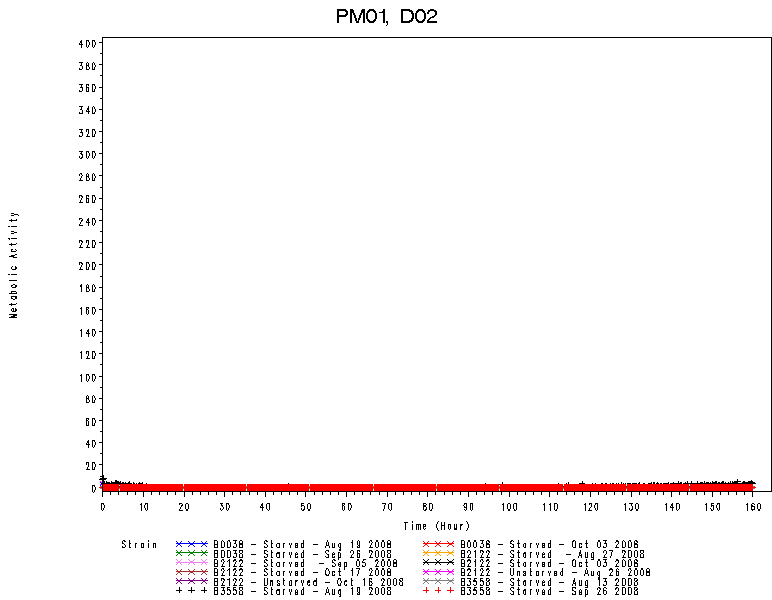

Supplement: Figure S3 — Kinetic curves for all PM plates with Mycobacterium bovis Type 9 strains. (ZIP) [file pone.0052673.s003.zip › suppl fig 3G type 9/Plate01/pm01d02.gif]

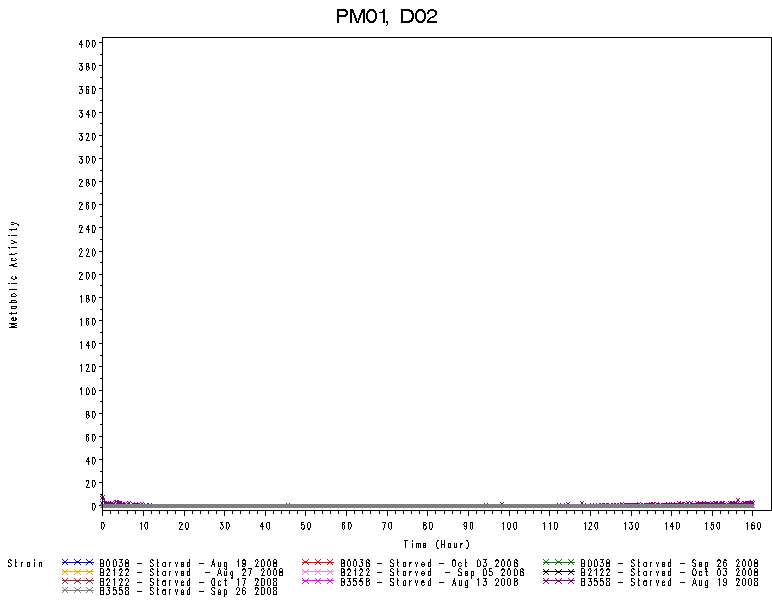

Supplement: Figure S3 — Kinetic curves for all PM plates with Mycobacterium bovis Type 9 strains. (ZIP) [file pone.0052673.s003.zip › suppl fig 3G type 9/Plate01/pm01d021.gif]

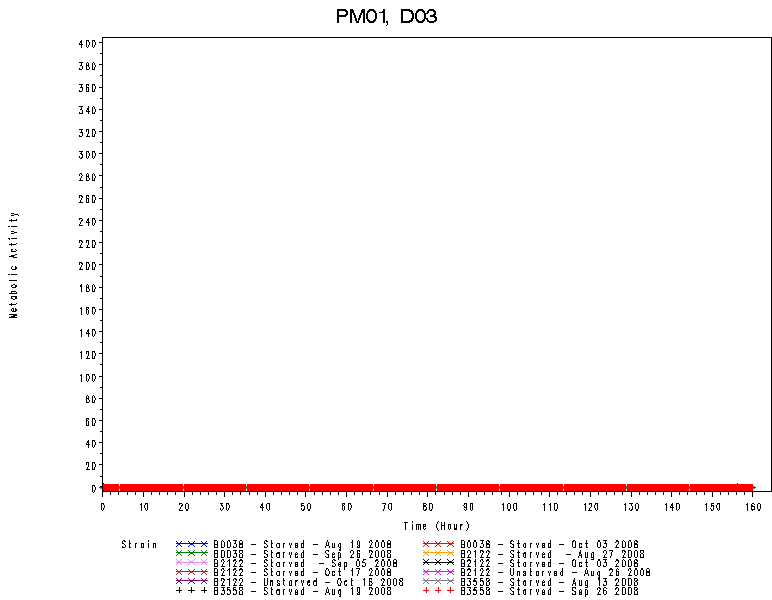

Supplement: Figure S3 — Kinetic curves for all PM plates with Mycobacterium bovis Type 9 strains. (ZIP) [file pone.0052673.s003.zip › suppl fig 3G type 9/Plate01/pm01d03.gif]

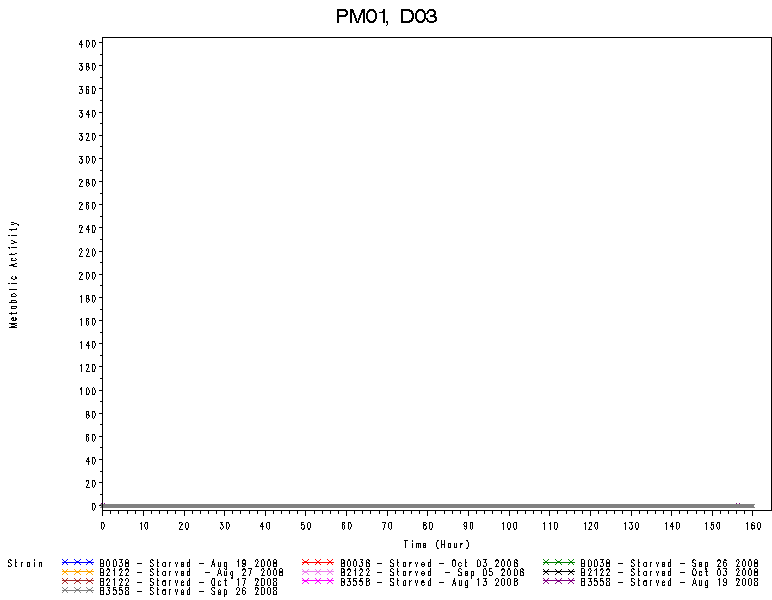

Supplement: Figure S3 — Kinetic curves for all PM plates with Mycobacterium bovis Type 9 strains. (ZIP) [file pone.0052673.s003.zip › suppl fig 3G type 9/Plate01/pm01d031.gif]

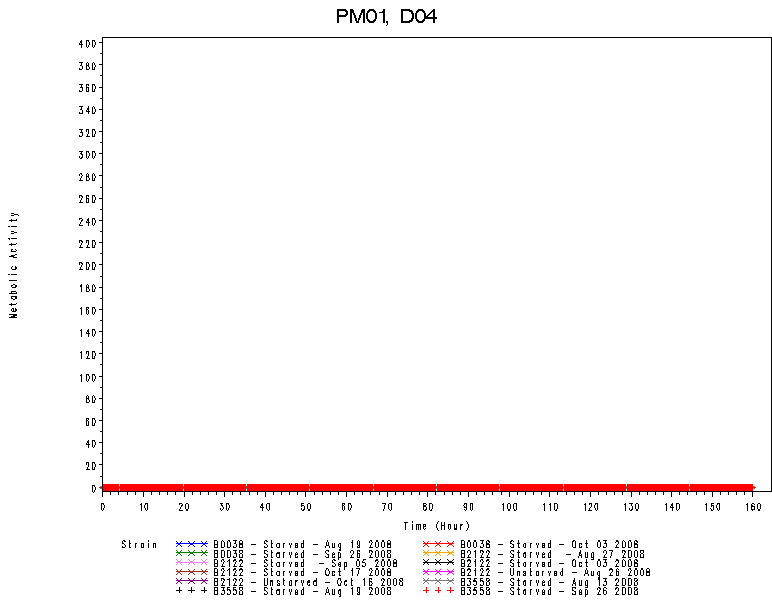

Supplement: Figure S3 — Kinetic curves for all PM plates with Mycobacterium bovis Type 9 strains. (ZIP) [file pone.0052673.s003.zip › suppl fig 3G type 9/Plate01/pm01d04.gif]

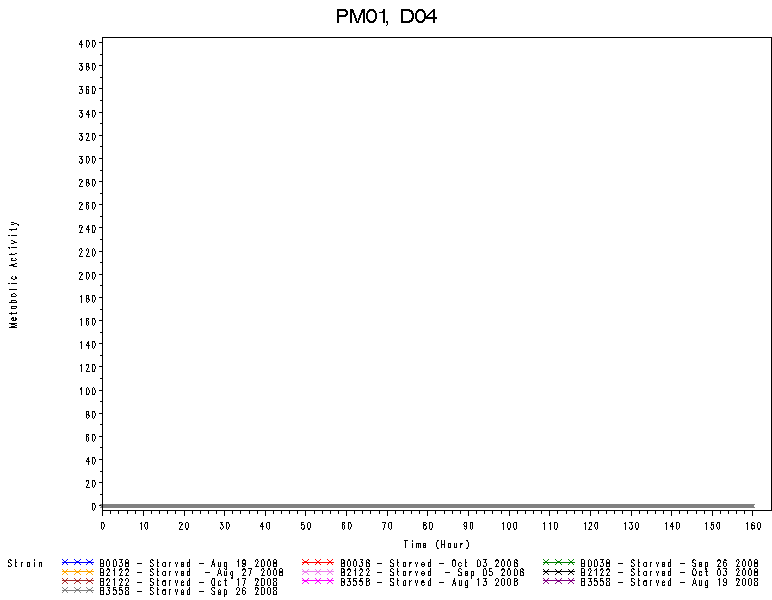

Supplement: Figure S3 — Kinetic curves for all PM plates with Mycobacterium bovis Type 9 strains. (ZIP) [file pone.0052673.s003.zip › suppl fig 3G type 9/Plate01/pm01d041.gif]

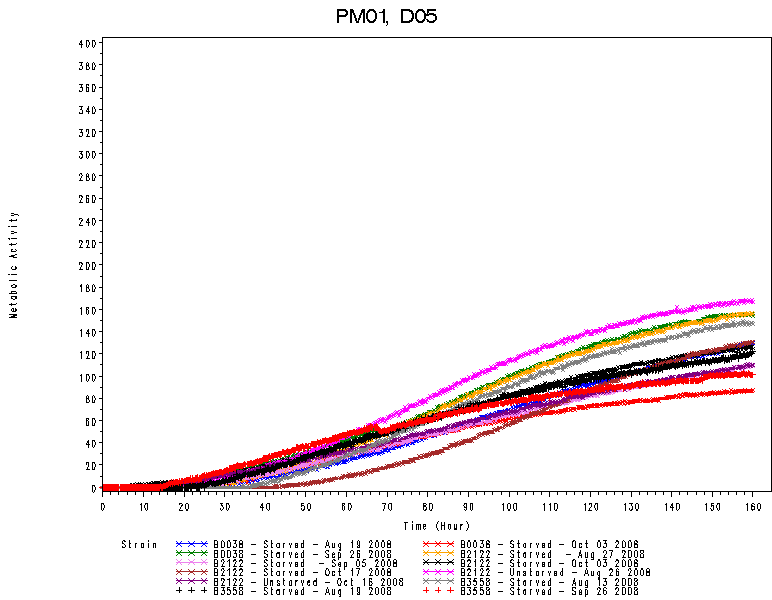

Supplement: Figure S3 — Kinetic curves for all PM plates with Mycobacterium bovis Type 9 strains. (ZIP) [file pone.0052673.s003.zip › suppl fig 3G type 9/Plate01/pm01d05.gif]

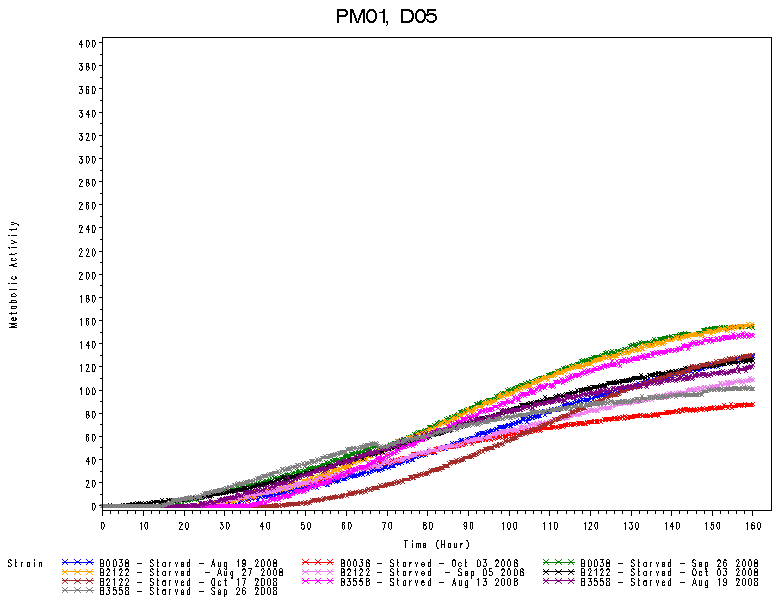

Supplement: Figure S3 — Kinetic curves for all PM plates with Mycobacterium bovis Type 9 strains. (ZIP) [file pone.0052673.s003.zip › suppl fig 3G type 9/Plate01/pm01d051.gif]

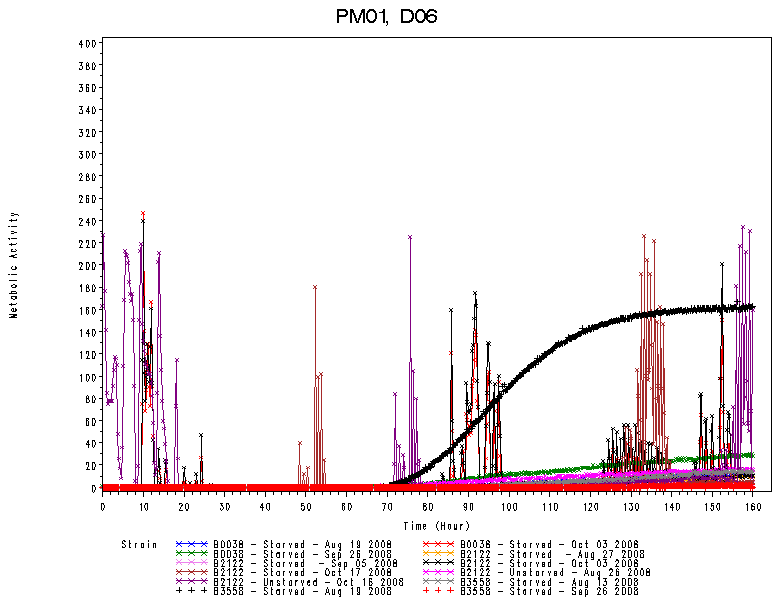

Supplement: Figure S3 — Kinetic curves for all PM plates with Mycobacterium bovis Type 9 strains. (ZIP) [file pone.0052673.s003.zip › suppl fig 3G type 9/Plate01/pm01d06.gif]

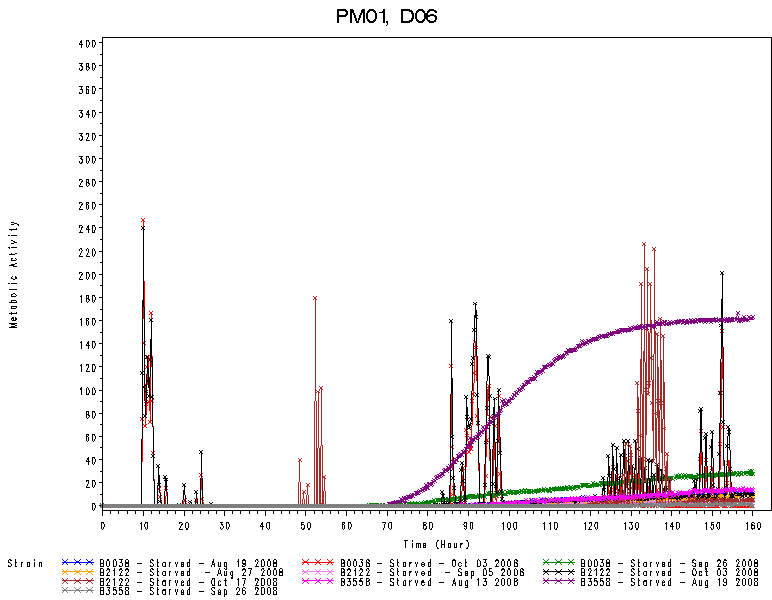

Supplement: Figure S3 — Kinetic curves for all PM plates with Mycobacterium bovis Type 9 strains. (ZIP) [file pone.0052673.s003.zip › suppl fig 3G type 9/Plate01/pm01d061.gif]

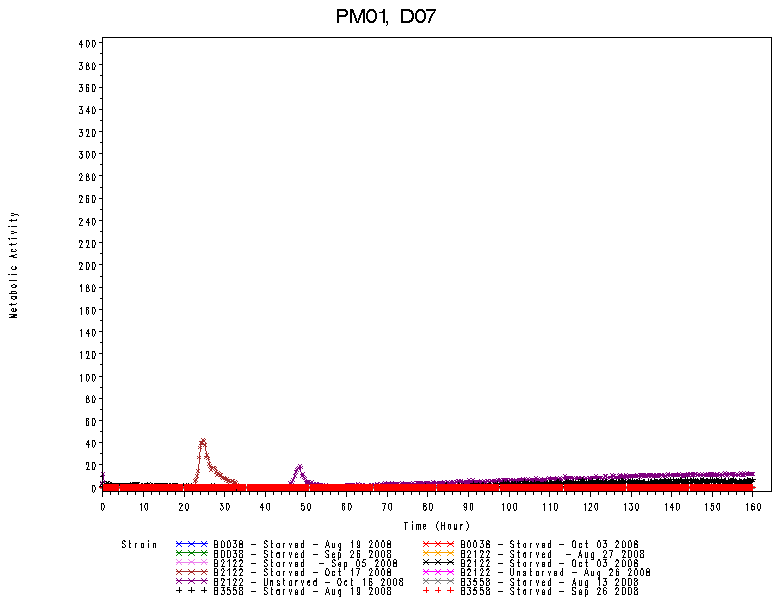

Supplement: Figure S3 — Kinetic curves for all PM plates with Mycobacterium bovis Type 9 strains. (ZIP) [file pone.0052673.s003.zip › suppl fig 3G type 9/Plate01/pm01d07.gif]

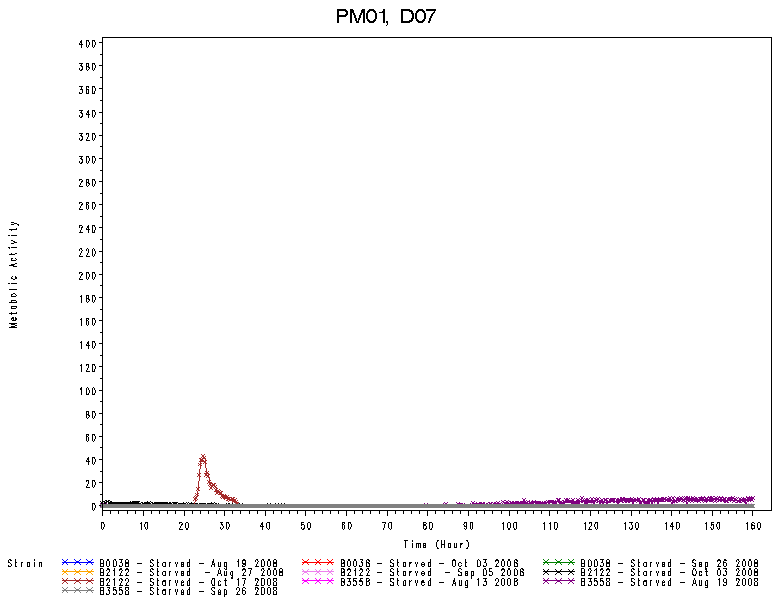

Supplement: Figure S3 — Kinetic curves for all PM plates with Mycobacterium bovis Type 9 strains. (ZIP) [file pone.0052673.s003.zip › suppl fig 3G type 9/Plate01/pm01d071.gif]

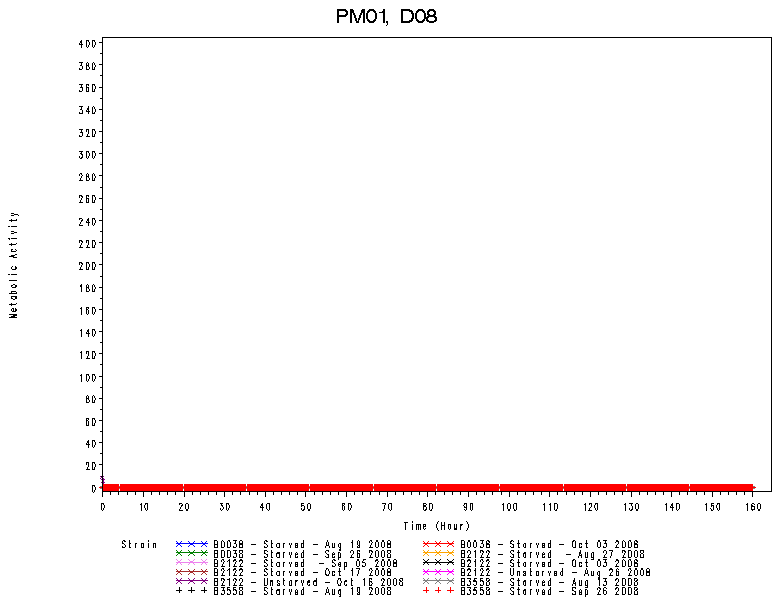

Supplement: Figure S3 — Kinetic curves for all PM plates with Mycobacterium bovis Type 9 strains. (ZIP) [file pone.0052673.s003.zip › suppl fig 3G type 9/Plate01/pm01d08.gif]

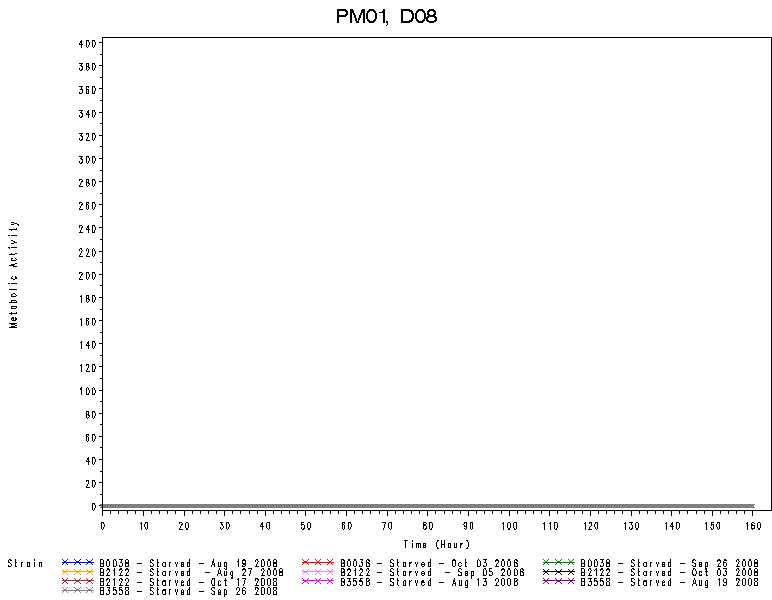

Supplement: Figure S3 — Kinetic curves for all PM plates with Mycobacterium bovis Type 9 strains. (ZIP) [file pone.0052673.s003.zip › suppl fig 3G type 9/Plate01/pm01d081.gif]

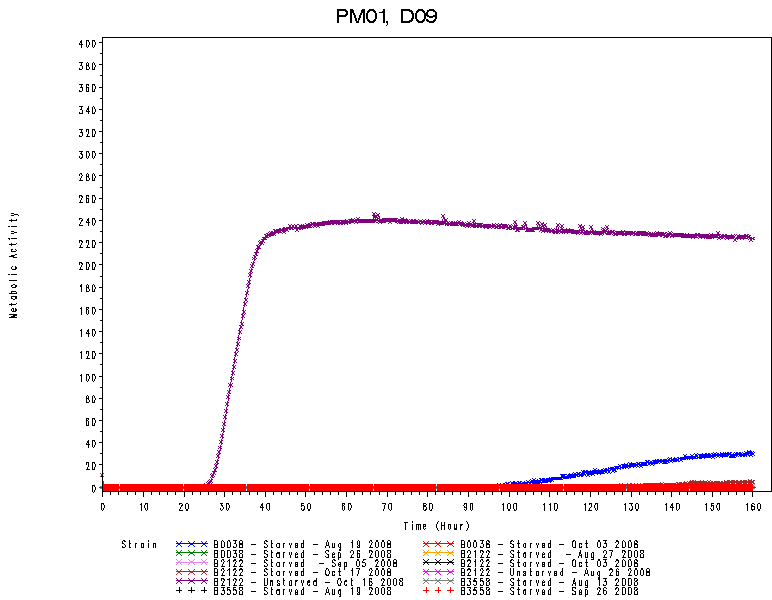

Supplement: Figure S3 — Kinetic curves for all PM plates with Mycobacterium bovis Type 9 strains. (ZIP) [file pone.0052673.s003.zip › suppl fig 3G type 9/Plate01/pm01d09.gif]

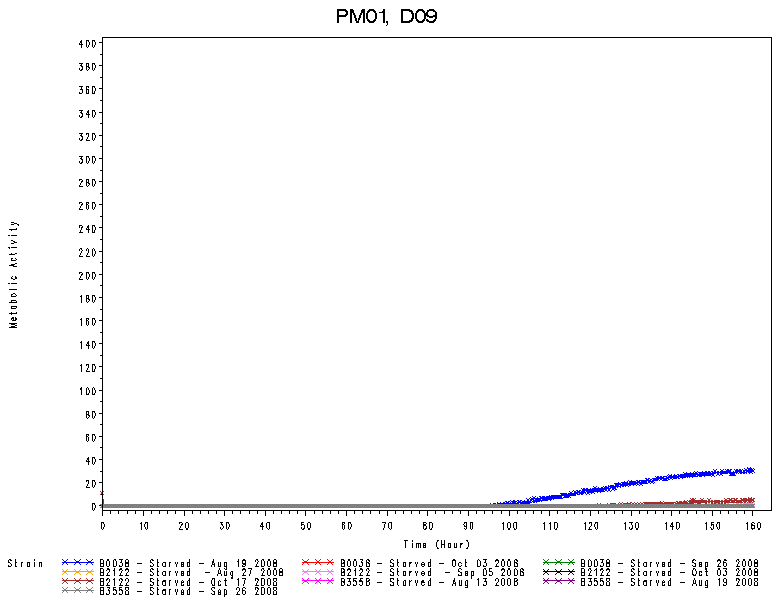

Supplement: Figure S3 — Kinetic curves for all PM plates with Mycobacterium bovis Type 9 strains. (ZIP) [file pone.0052673.s003.zip › suppl fig 3G type 9/Plate01/pm01d091.gif]

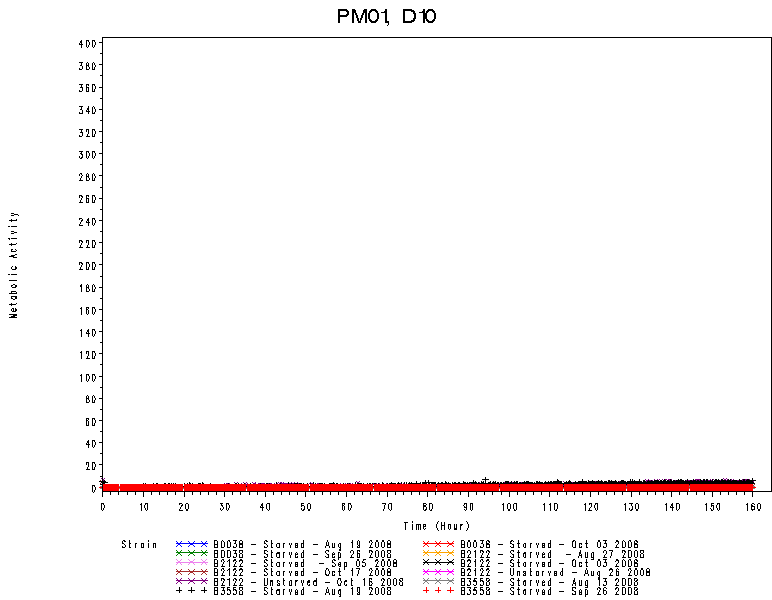

Supplement: Figure S3 — Kinetic curves for all PM plates with Mycobacterium bovis Type 9 strains. (ZIP) [file pone.0052673.s003.zip › suppl fig 3G type 9/Plate01/pm01d10.gif]

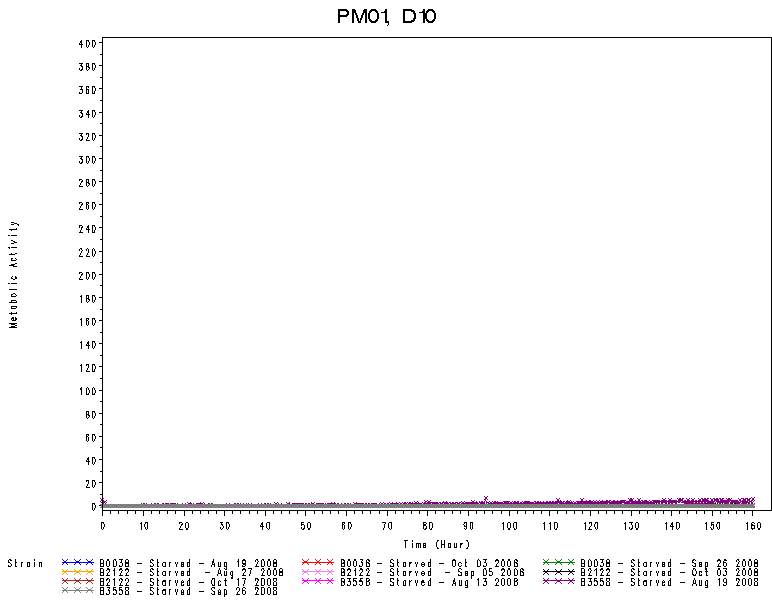

Supplement: Figure S3 — Kinetic curves for all PM plates with Mycobacterium bovis Type 9 strains. (ZIP) [file pone.0052673.s003.zip › suppl fig 3G type 9/Plate01/pm01d101.gif]

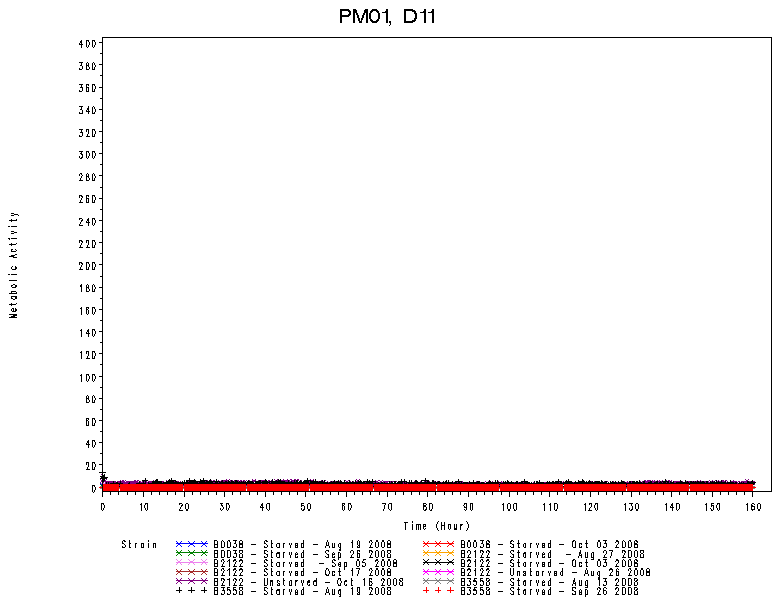

Supplement: Figure S3 — Kinetic curves for all PM plates with Mycobacterium bovis Type 9 strains. (ZIP) [file pone.0052673.s003.zip › suppl fig 3G type 9/Plate01/pm01d11.gif]

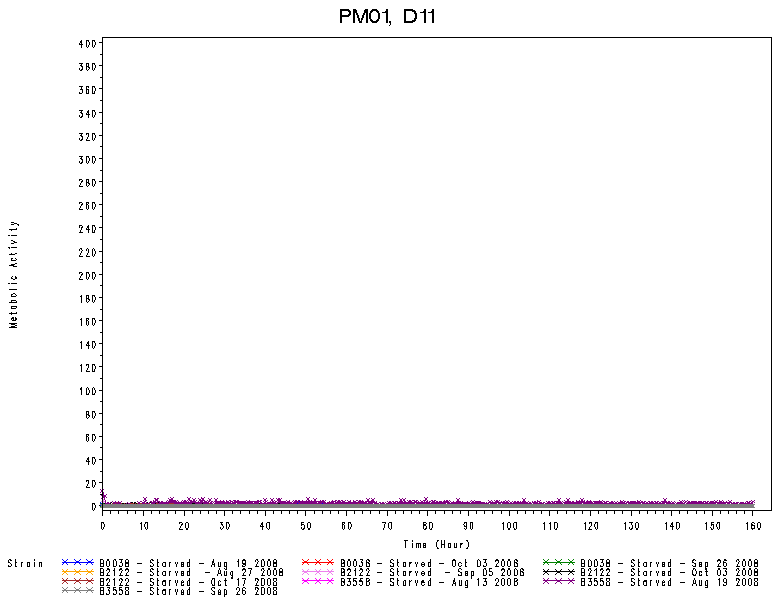

Supplement: Figure S3 — Kinetic curves for all PM plates with Mycobacterium bovis Type 9 strains. (ZIP) [file pone.0052673.s003.zip › suppl fig 3G type 9/Plate01/pm01d111.gif]

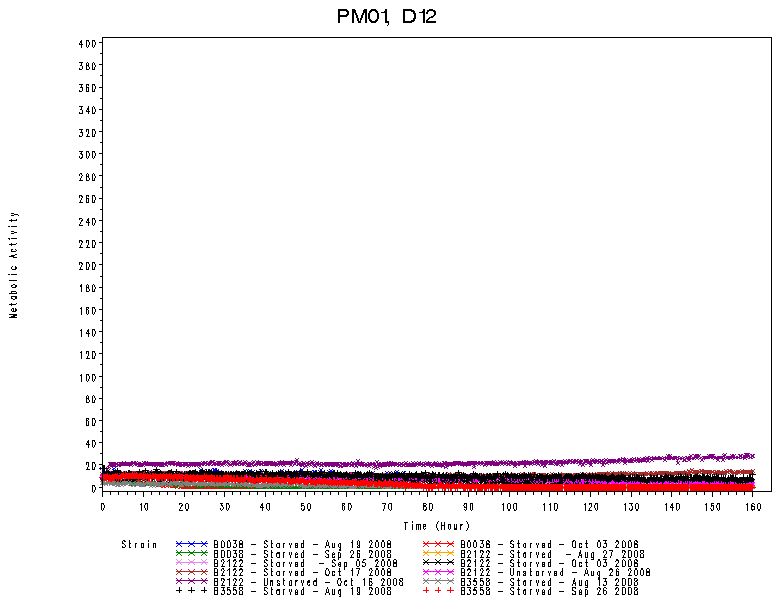

Supplement: Figure S3 — Kinetic curves for all PM plates with Mycobacterium bovis Type 9 strains. (ZIP) [file pone.0052673.s003.zip › suppl fig 3G type 9/Plate01/pm01d12.gif]

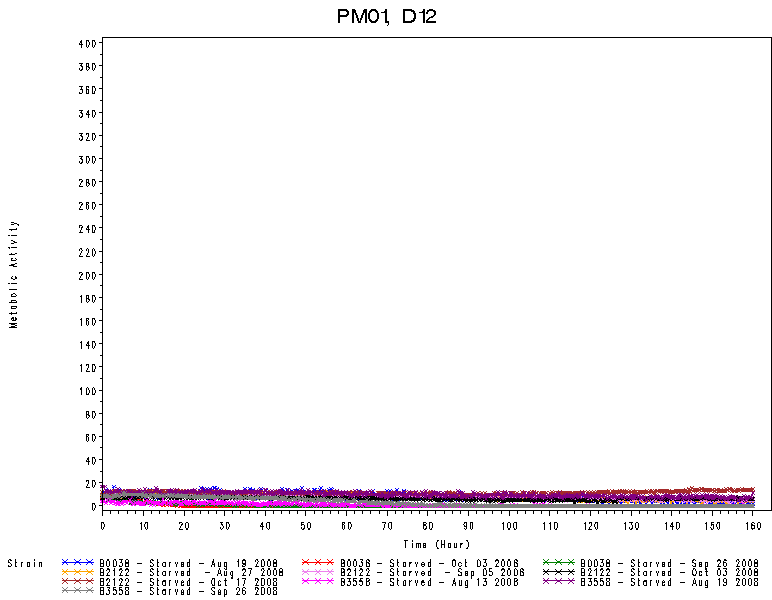

Supplement: Figure S3 — Kinetic curves for all PM plates with Mycobacterium bovis Type 9 strains. (ZIP) [file pone.0052673.s003.zip › suppl fig 3G type 9/Plate01/pm01d121.gif]

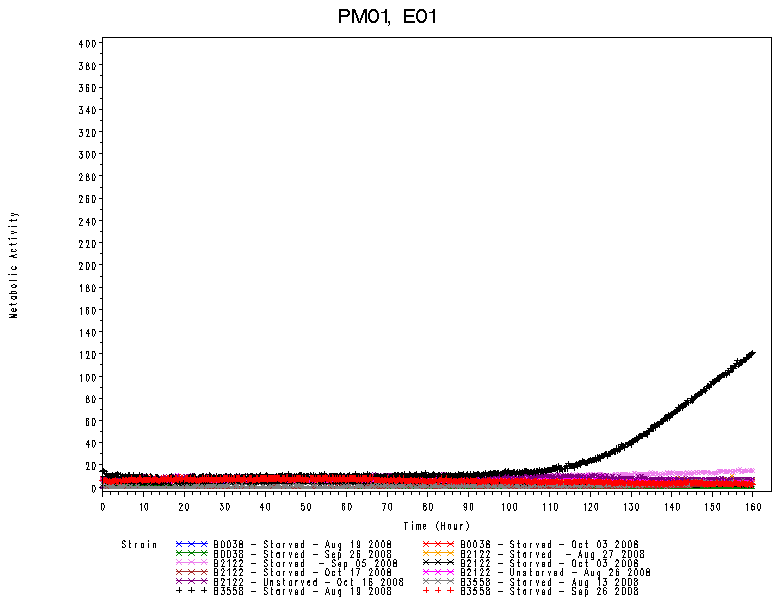

Supplement: Figure S3 — Kinetic curves for all PM plates with Mycobacterium bovis Type 9 strains. (ZIP) [file pone.0052673.s003.zip › suppl fig 3G type 9/Plate01/pm01e01.gif]

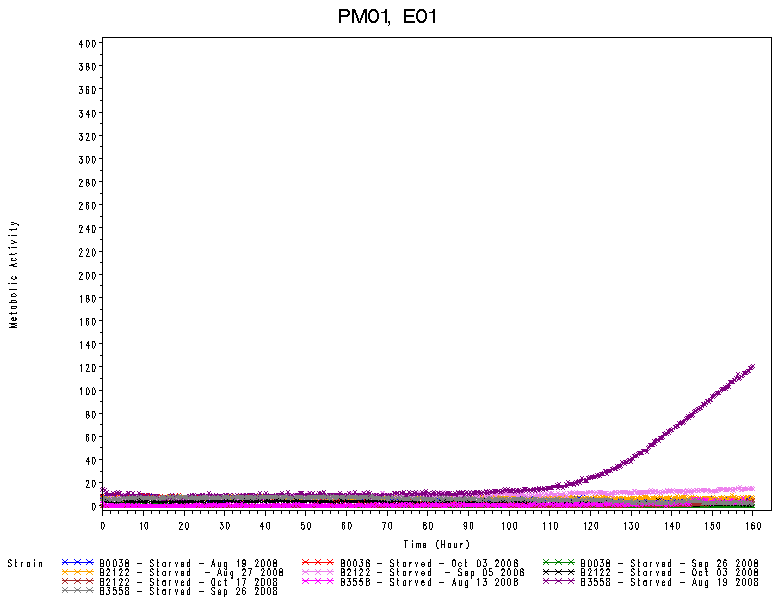

Supplement: Figure S3 — Kinetic curves for all PM plates with Mycobacterium bovis Type 9 strains. (ZIP) [file pone.0052673.s003.zip › suppl fig 3G type 9/Plate01/pm01e011.gif]

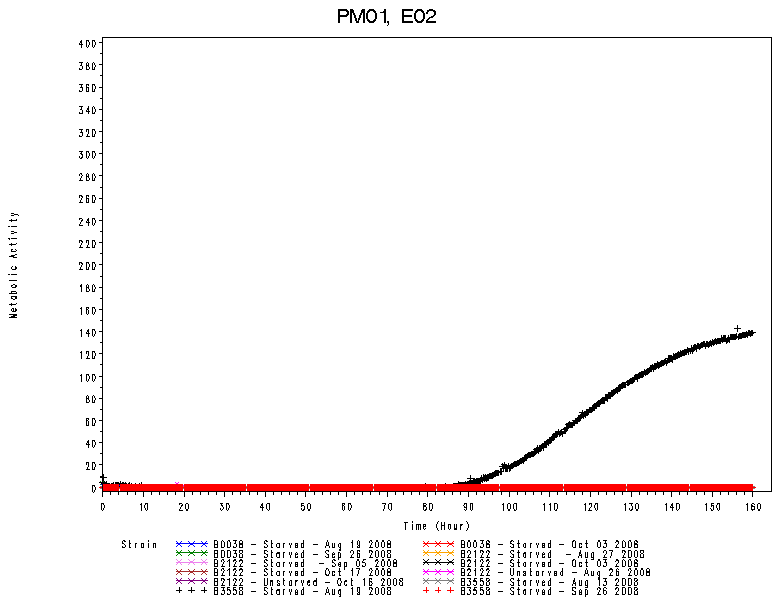

Supplement: Figure S3 — Kinetic curves for all PM plates with Mycobacterium bovis Type 9 strains. (ZIP) [file pone.0052673.s003.zip › suppl fig 3G type 9/Plate01/pm01e02.gif]

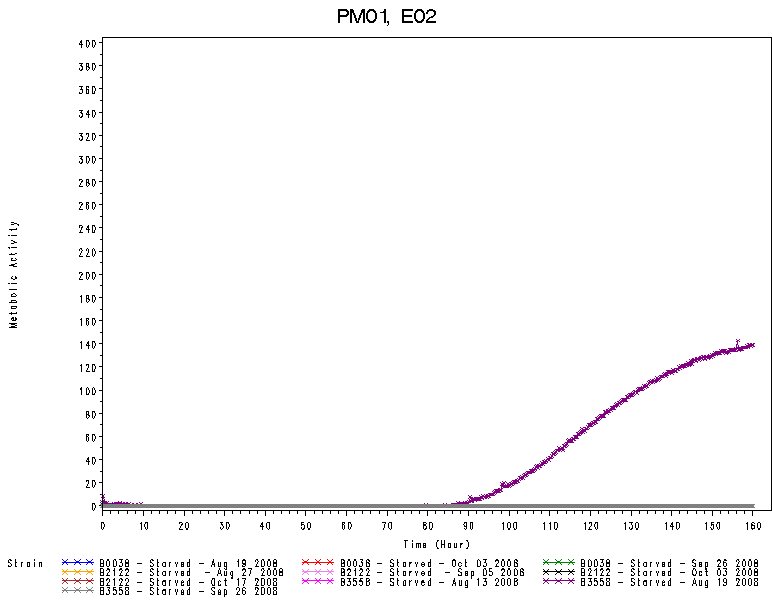

Supplement: Figure S3 — Kinetic curves for all PM plates with Mycobacterium bovis Type 9 strains. (ZIP) [file pone.0052673.s003.zip › suppl fig 3G type 9/Plate01/pm01e021.gif]
