# Supplementary figures and images for: High Throughput Phenotypic Analysis of Mycobacterium tuberculosis and Mycobacterium bovis Strains' Metabolism Using Biolog Phenotype Microarrays (part 9 of 11)
Source: PLoS One. 2013 Jan 10;8(1):e52673. doi: 10.1371/journal.pone.0052673 (PMC3542357; doi:10.1371/journal.pone.0052673)

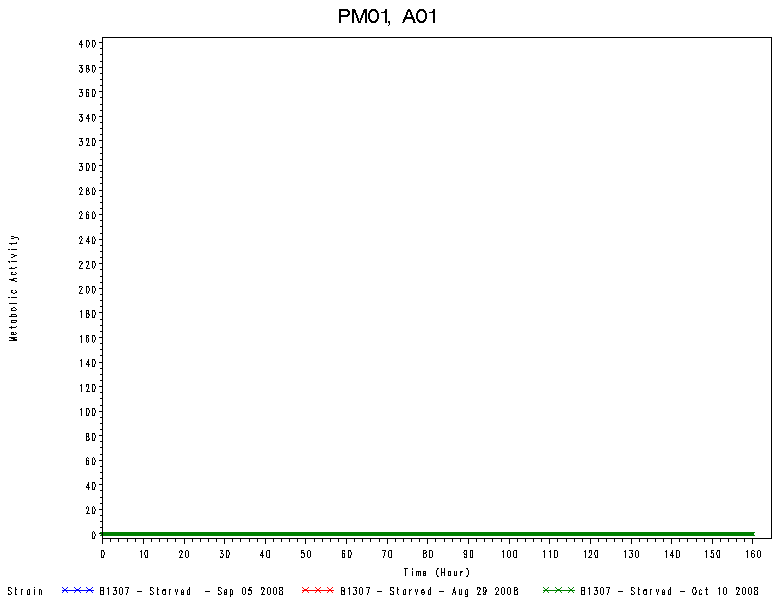

Supplement: Figure S5 — Kinetic curves for all PM plates with M. bovis Type 35 strains. Figures S1 to S5 were generated in SAS using a GPLOT procedure, as described in the methods. Each figure is a Zip file containing plots of Omnilog units (due to dye reduction) against time (0 to 168 h) for all wells of each of the six 96 well plates. Each well is identified by (plate, well) and a list of the contents of wells is in Supplementary Table S1. (ZIP) [file pone.0052673.s005.zip › suppl fig 5G type 35/Plate01/pm01a01.gif]

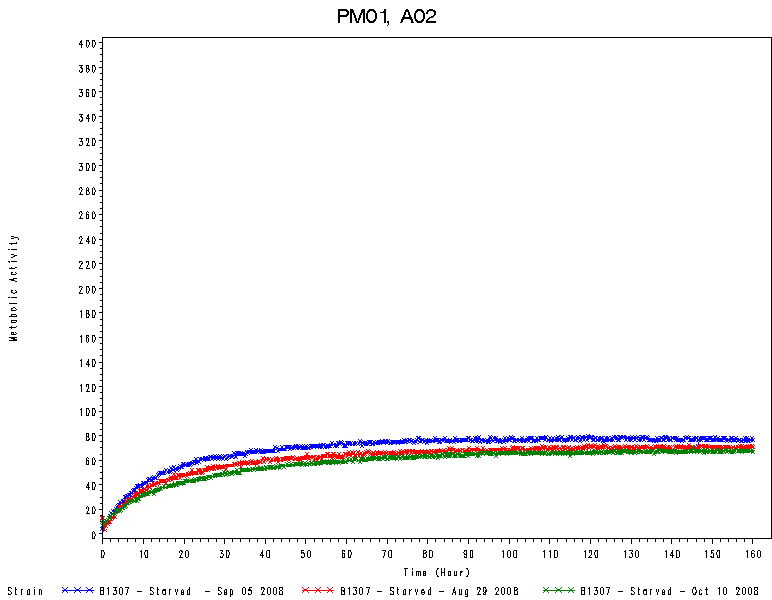

Supplement: Figure S5 — Kinetic curves for all PM plates with M. bovis Type 35 strains. Figures S1 to S5 were generated in SAS using a GPLOT procedure, as described in the methods. Each figure is a Zip file containing plots of Omnilog units (due to dye reduction) against time (0 to 168 h) for all wells of each of the six 96 well plates. Each well is identified by (plate, well) and a list of the contents of wells is in Supplementary Table S1. (ZIP) [file pone.0052673.s005.zip › suppl fig 5G type 35/Plate01/pm01a02.gif]

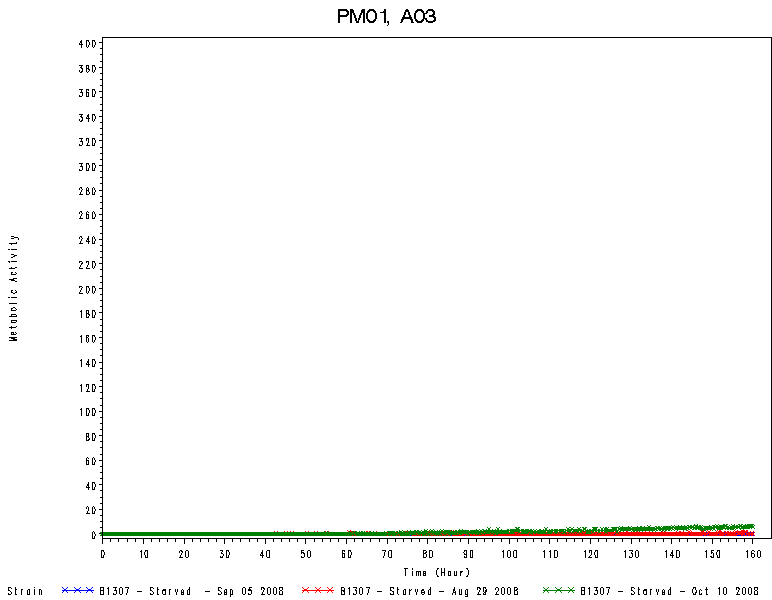

Supplement: Figure S5 — Kinetic curves for all PM plates with M. bovis Type 35 strains. Figures S1 to S5 were generated in SAS using a GPLOT procedure, as described in the methods. Each figure is a Zip file containing plots of Omnilog units (due to dye reduction) against time (0 to 168 h) for all wells of each of the six 96 well plates. Each well is identified by (plate, well) and a list of the contents of wells is in Supplementary Table S1. (ZIP) [file pone.0052673.s005.zip › suppl fig 5G type 35/Plate01/pm01a03.gif]

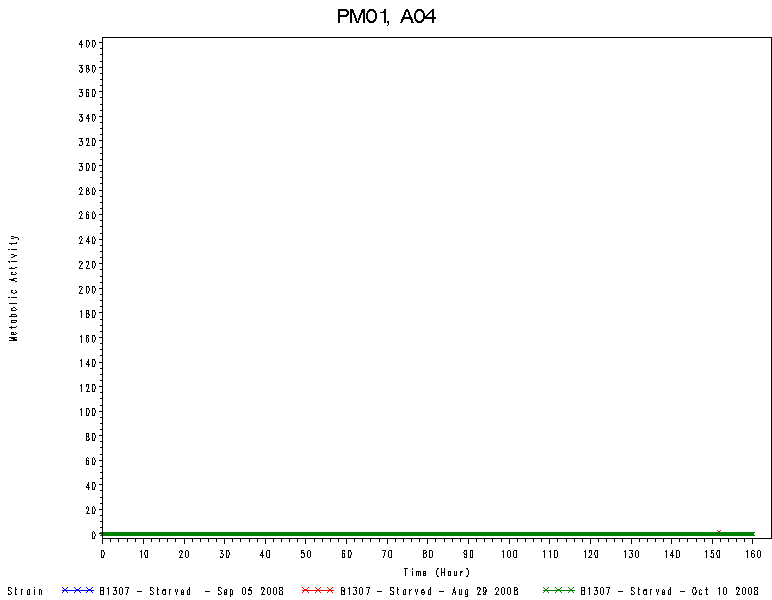

Supplement: Figure S5 — Kinetic curves for all PM plates with M. bovis Type 35 strains. Figures S1 to S5 were generated in SAS using a GPLOT procedure, as described in the methods. Each figure is a Zip file containing plots of Omnilog units (due to dye reduction) against time (0 to 168 h) for all wells of each of the six 96 well plates. Each well is identified by (plate, well) and a list of the contents of wells is in Supplementary Table S1. (ZIP) [file pone.0052673.s005.zip › suppl fig 5G type 35/Plate01/pm01a04.gif]

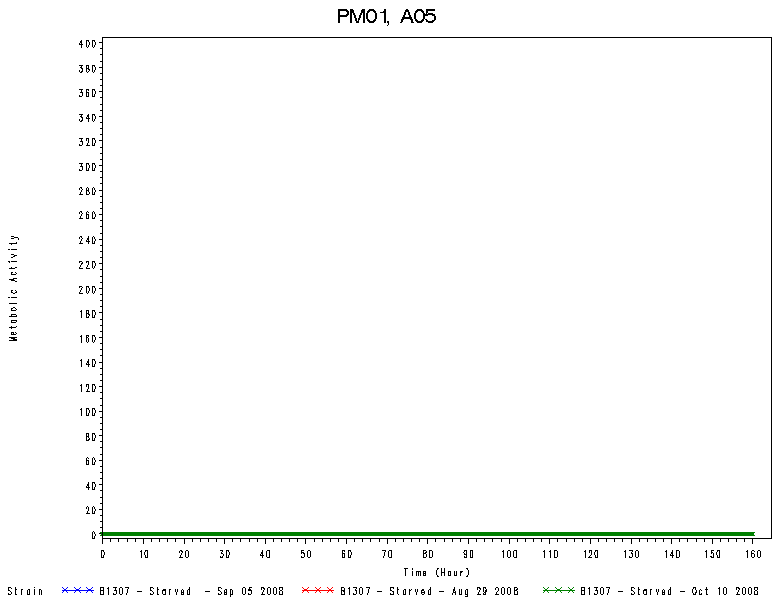

Supplement: Figure S5 — Kinetic curves for all PM plates with M. bovis Type 35 strains. Figures S1 to S5 were generated in SAS using a GPLOT procedure, as described in the methods. Each figure is a Zip file containing plots of Omnilog units (due to dye reduction) against time (0 to 168 h) for all wells of each of the six 96 well plates. Each well is identified by (plate, well) and a list of the contents of wells is in Supplementary Table S1. (ZIP) [file pone.0052673.s005.zip › suppl fig 5G type 35/Plate01/pm01a05.gif]

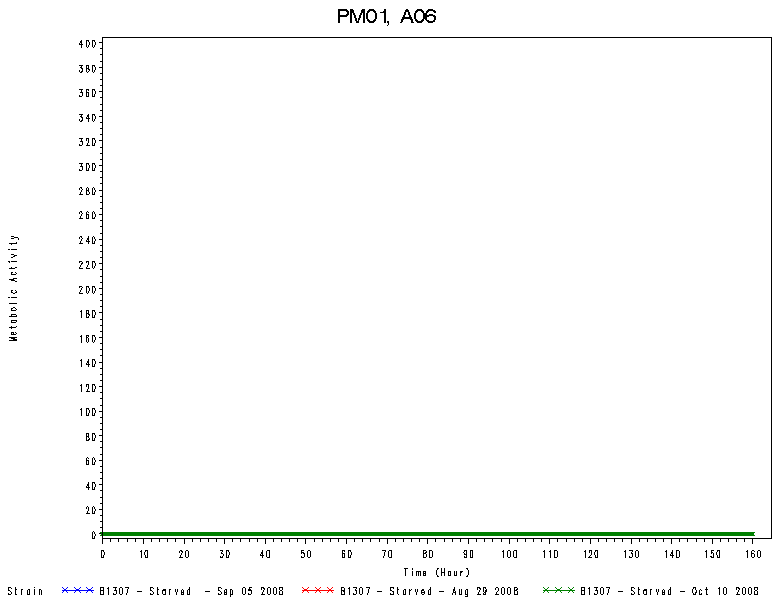

Supplement: Figure S5 — Kinetic curves for all PM plates with M. bovis Type 35 strains. Figures S1 to S5 were generated in SAS using a GPLOT procedure, as described in the methods. Each figure is a Zip file containing plots of Omnilog units (due to dye reduction) against time (0 to 168 h) for all wells of each of the six 96 well plates. Each well is identified by (plate, well) and a list of the contents of wells is in Supplementary Table S1. (ZIP) [file pone.0052673.s005.zip › suppl fig 5G type 35/Plate01/pm01a06.gif]

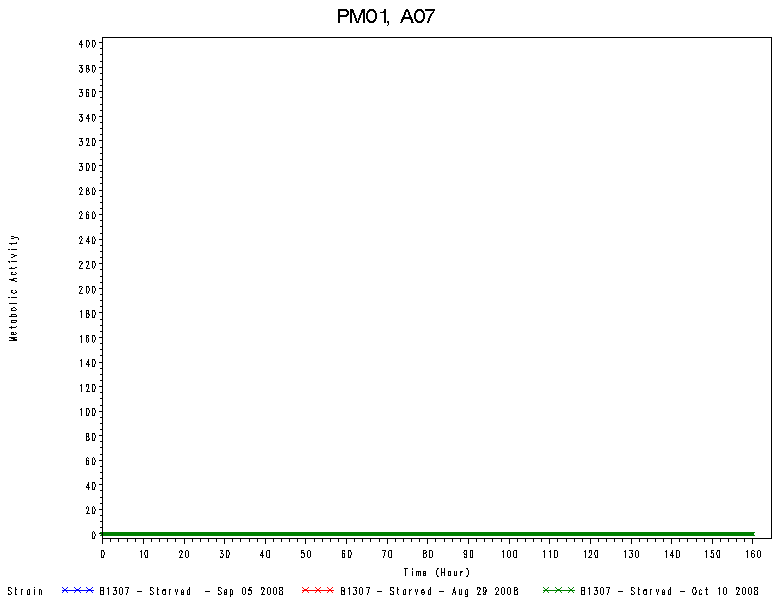

Supplement: Figure S5 — Kinetic curves for all PM plates with M. bovis Type 35 strains. Figures S1 to S5 were generated in SAS using a GPLOT procedure, as described in the methods. Each figure is a Zip file containing plots of Omnilog units (due to dye reduction) against time (0 to 168 h) for all wells of each of the six 96 well plates. Each well is identified by (plate, well) and a list of the contents of wells is in Supplementary Table S1. (ZIP) [file pone.0052673.s005.zip › suppl fig 5G type 35/Plate01/pm01a07.gif]

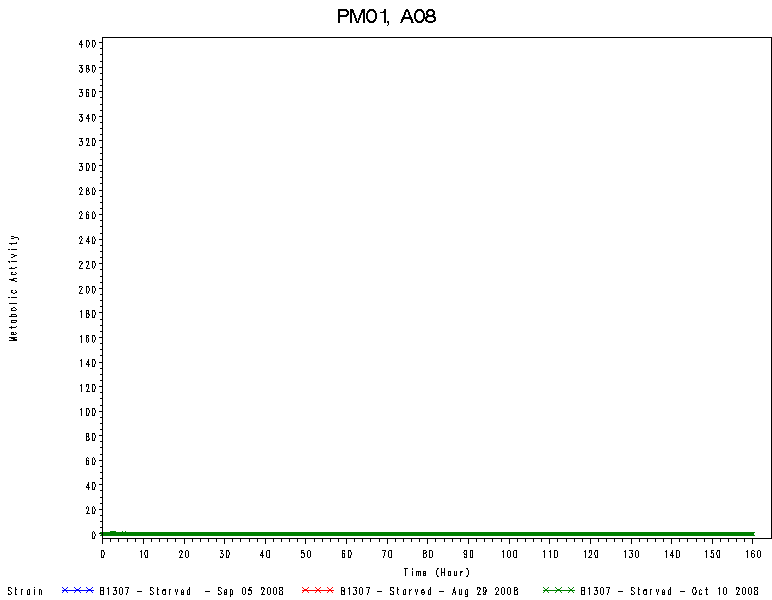

Supplement: Figure S5 — Kinetic curves for all PM plates with M. bovis Type 35 strains. Figures S1 to S5 were generated in SAS using a GPLOT procedure, as described in the methods. Each figure is a Zip file containing plots of Omnilog units (due to dye reduction) against time (0 to 168 h) for all wells of each of the six 96 well plates. Each well is identified by (plate, well) and a list of the contents of wells is in Supplementary Table S1. (ZIP) [file pone.0052673.s005.zip › suppl fig 5G type 35/Plate01/pm01a08.gif]

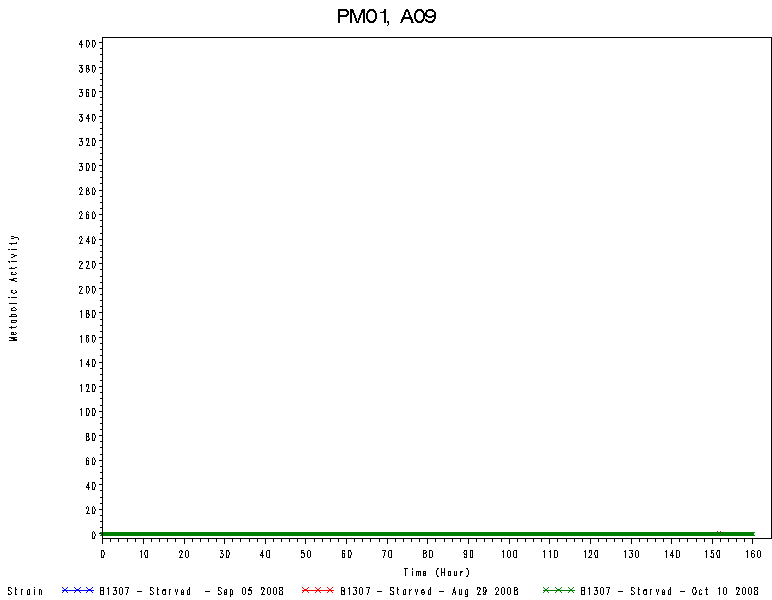

Supplement: Figure S5 — Kinetic curves for all PM plates with M. bovis Type 35 strains. Figures S1 to S5 were generated in SAS using a GPLOT procedure, as described in the methods. Each figure is a Zip file containing plots of Omnilog units (due to dye reduction) against time (0 to 168 h) for all wells of each of the six 96 well plates. Each well is identified by (plate, well) and a list of the contents of wells is in Supplementary Table S1. (ZIP) [file pone.0052673.s005.zip › suppl fig 5G type 35/Plate01/pm01a09.gif]

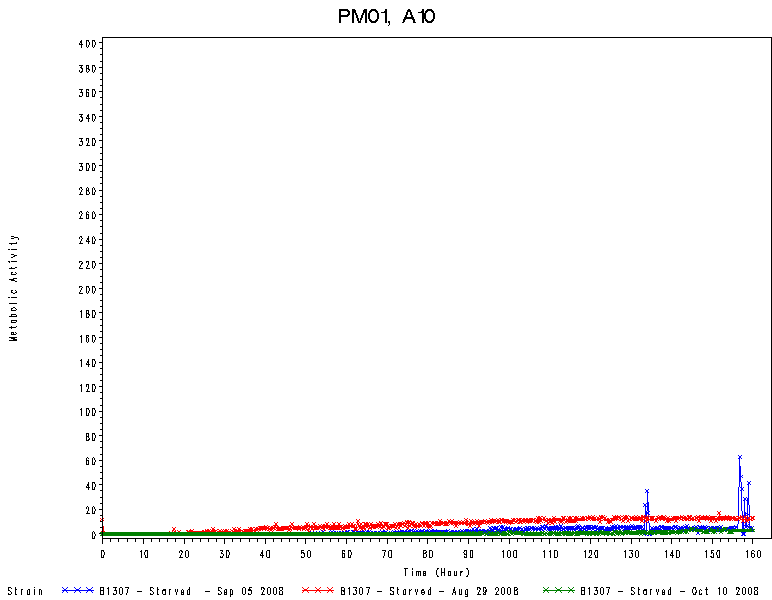

Supplement: Figure S5 — Kinetic curves for all PM plates with M. bovis Type 35 strains. Figures S1 to S5 were generated in SAS using a GPLOT procedure, as described in the methods. Each figure is a Zip file containing plots of Omnilog units (due to dye reduction) against time (0 to 168 h) for all wells of each of the six 96 well plates. Each well is identified by (plate, well) and a list of the contents of wells is in Supplementary Table S1. (ZIP) [file pone.0052673.s005.zip › suppl fig 5G type 35/Plate01/pm01a10.gif]

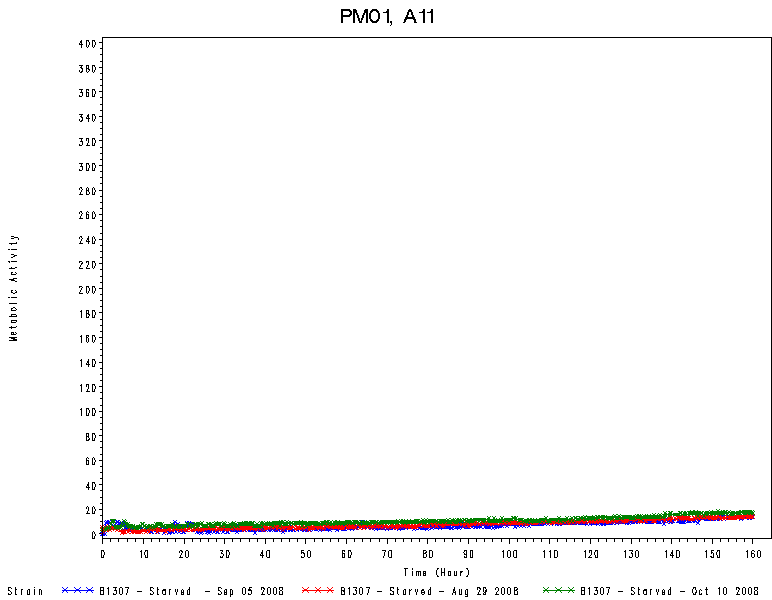

Supplement: Figure S5 — Kinetic curves for all PM plates with M. bovis Type 35 strains. Figures S1 to S5 were generated in SAS using a GPLOT procedure, as described in the methods. Each figure is a Zip file containing plots of Omnilog units (due to dye reduction) against time (0 to 168 h) for all wells of each of the six 96 well plates. Each well is identified by (plate, well) and a list of the contents of wells is in Supplementary Table S1. (ZIP) [file pone.0052673.s005.zip › suppl fig 5G type 35/Plate01/pm01a11.gif]

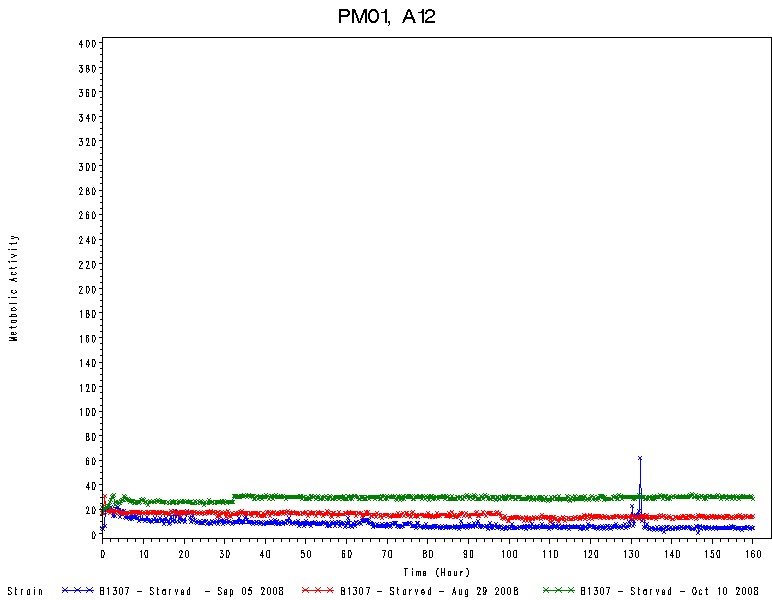

Supplement: Figure S5 — Kinetic curves for all PM plates with M. bovis Type 35 strains. Figures S1 to S5 were generated in SAS using a GPLOT procedure, as described in the methods. Each figure is a Zip file containing plots of Omnilog units (due to dye reduction) against time (0 to 168 h) for all wells of each of the six 96 well plates. Each well is identified by (plate, well) and a list of the contents of wells is in Supplementary Table S1. (ZIP) [file pone.0052673.s005.zip › suppl fig 5G type 35/Plate01/pm01a12.gif]

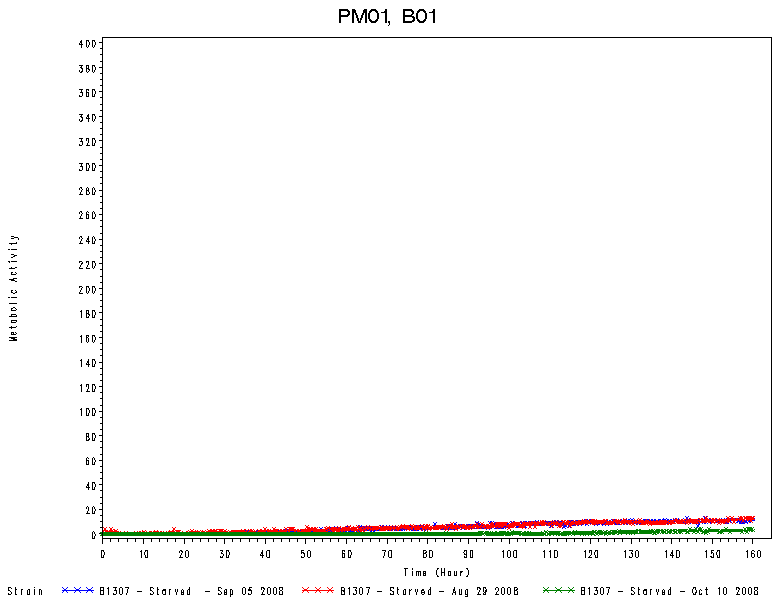

Supplement: Figure S5 — Kinetic curves for all PM plates with M. bovis Type 35 strains. Figures S1 to S5 were generated in SAS using a GPLOT procedure, as described in the methods. Each figure is a Zip file containing plots of Omnilog units (due to dye reduction) against time (0 to 168 h) for all wells of each of the six 96 well plates. Each well is identified by (plate, well) and a list of the contents of wells is in Supplementary Table S1. (ZIP) [file pone.0052673.s005.zip › suppl fig 5G type 35/Plate01/pm01b01.gif]

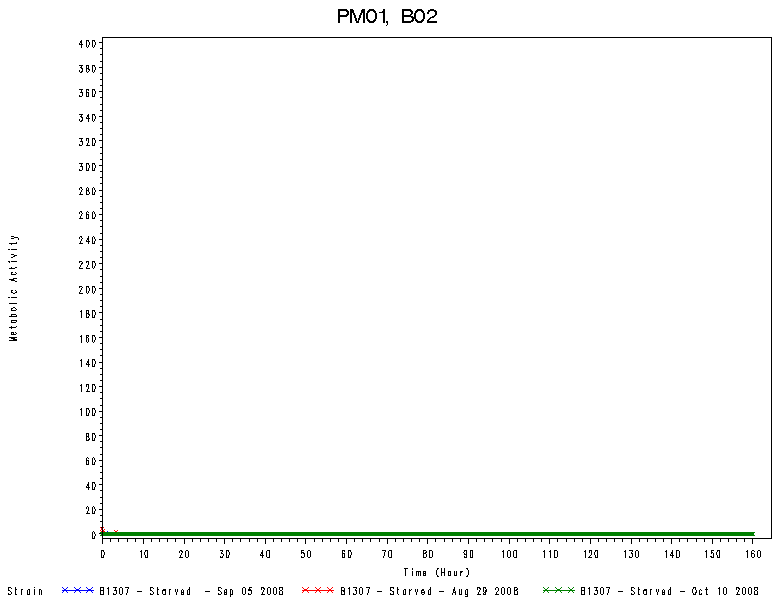

Supplement: Figure S5 — Kinetic curves for all PM plates with M. bovis Type 35 strains. Figures S1 to S5 were generated in SAS using a GPLOT procedure, as described in the methods. Each figure is a Zip file containing plots of Omnilog units (due to dye reduction) against time (0 to 168 h) for all wells of each of the six 96 well plates. Each well is identified by (plate, well) and a list of the contents of wells is in Supplementary Table S1. (ZIP) [file pone.0052673.s005.zip › suppl fig 5G type 35/Plate01/pm01b02.gif]

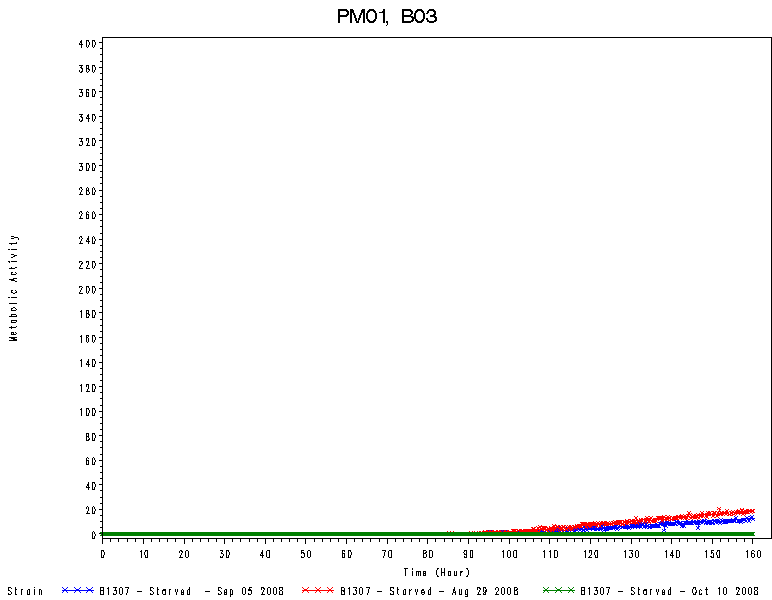

Supplement: Figure S5 — Kinetic curves for all PM plates with M. bovis Type 35 strains. Figures S1 to S5 were generated in SAS using a GPLOT procedure, as described in the methods. Each figure is a Zip file containing plots of Omnilog units (due to dye reduction) against time (0 to 168 h) for all wells of each of the six 96 well plates. Each well is identified by (plate, well) and a list of the contents of wells is in Supplementary Table S1. (ZIP) [file pone.0052673.s005.zip › suppl fig 5G type 35/Plate01/pm01b03.gif]

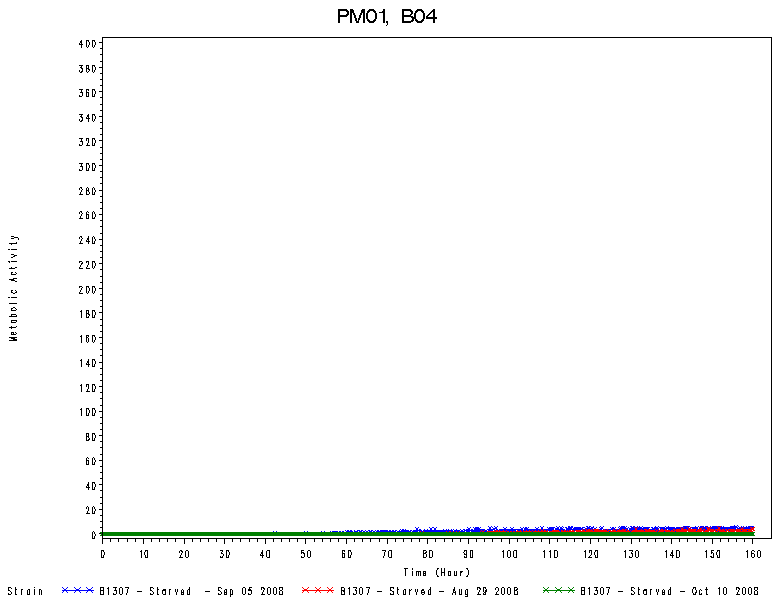

Supplement: Figure S5 — Kinetic curves for all PM plates with M. bovis Type 35 strains. Figures S1 to S5 were generated in SAS using a GPLOT procedure, as described in the methods. Each figure is a Zip file containing plots of Omnilog units (due to dye reduction) against time (0 to 168 h) for all wells of each of the six 96 well plates. Each well is identified by (plate, well) and a list of the contents of wells is in Supplementary Table S1. (ZIP) [file pone.0052673.s005.zip › suppl fig 5G type 35/Plate01/pm01b04.gif]

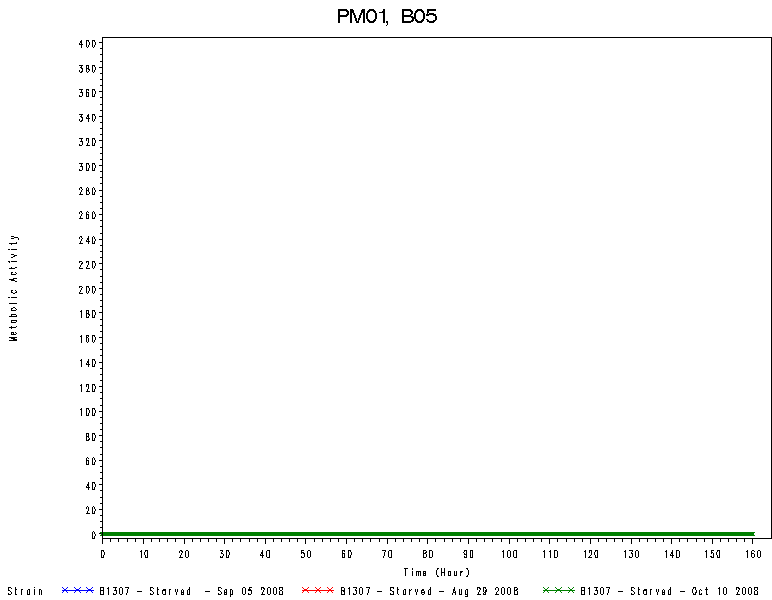

Supplement: Figure S5 — Kinetic curves for all PM plates with M. bovis Type 35 strains. Figures S1 to S5 were generated in SAS using a GPLOT procedure, as described in the methods. Each figure is a Zip file containing plots of Omnilog units (due to dye reduction) against time (0 to 168 h) for all wells of each of the six 96 well plates. Each well is identified by (plate, well) and a list of the contents of wells is in Supplementary Table S1. (ZIP) [file pone.0052673.s005.zip › suppl fig 5G type 35/Plate01/pm01b05.gif]

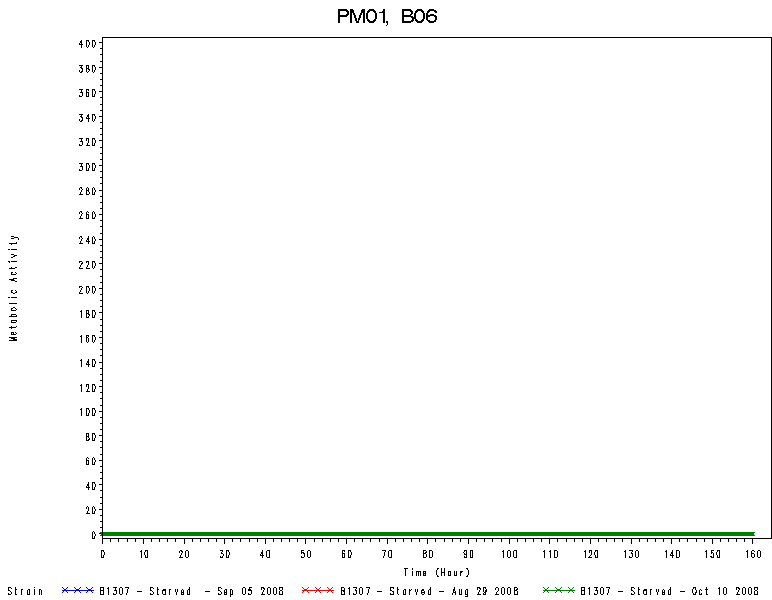

Supplement: Figure S5 — Kinetic curves for all PM plates with M. bovis Type 35 strains. Figures S1 to S5 were generated in SAS using a GPLOT procedure, as described in the methods. Each figure is a Zip file containing plots of Omnilog units (due to dye reduction) against time (0 to 168 h) for all wells of each of the six 96 well plates. Each well is identified by (plate, well) and a list of the contents of wells is in Supplementary Table S1. (ZIP) [file pone.0052673.s005.zip › suppl fig 5G type 35/Plate01/pm01b06.gif]

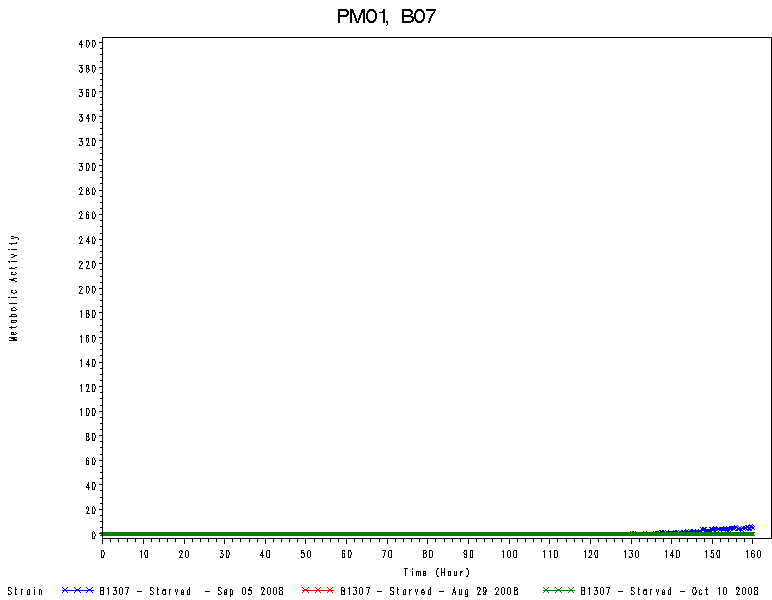

Supplement: Figure S5 — Kinetic curves for all PM plates with M. bovis Type 35 strains. Figures S1 to S5 were generated in SAS using a GPLOT procedure, as described in the methods. Each figure is a Zip file containing plots of Omnilog units (due to dye reduction) against time (0 to 168 h) for all wells of each of the six 96 well plates. Each well is identified by (plate, well) and a list of the contents of wells is in Supplementary Table S1. (ZIP) [file pone.0052673.s005.zip › suppl fig 5G type 35/Plate01/pm01b07.gif]

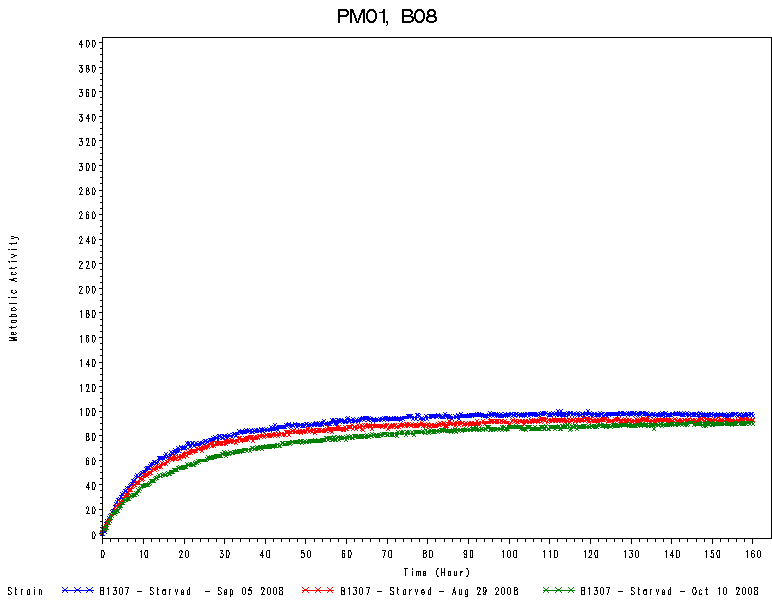

Supplement: Figure S5 — Kinetic curves for all PM plates with M. bovis Type 35 strains. Figures S1 to S5 were generated in SAS using a GPLOT procedure, as described in the methods. Each figure is a Zip file containing plots of Omnilog units (due to dye reduction) against time (0 to 168 h) for all wells of each of the six 96 well plates. Each well is identified by (plate, well) and a list of the contents of wells is in Supplementary Table S1. (ZIP) [file pone.0052673.s005.zip › suppl fig 5G type 35/Plate01/pm01b08.gif]

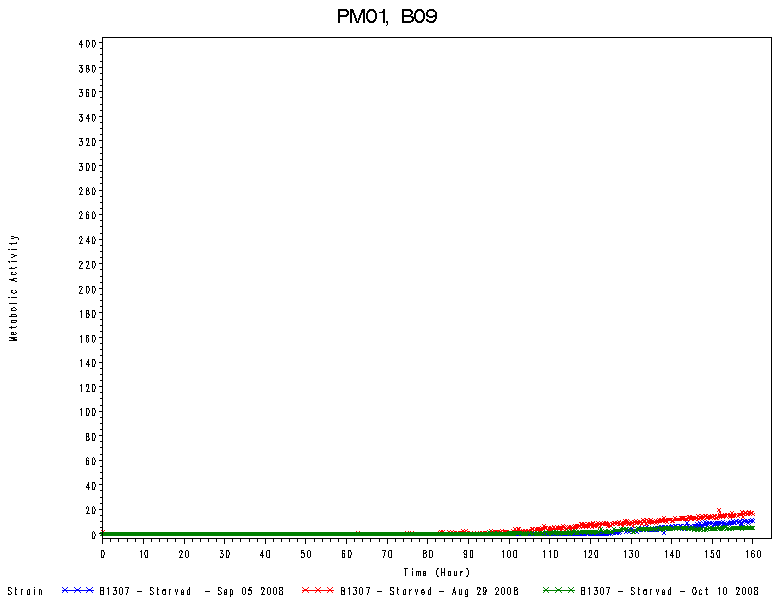

Supplement: Figure S5 — Kinetic curves for all PM plates with M. bovis Type 35 strains. Figures S1 to S5 were generated in SAS using a GPLOT procedure, as described in the methods. Each figure is a Zip file containing plots of Omnilog units (due to dye reduction) against time (0 to 168 h) for all wells of each of the six 96 well plates. Each well is identified by (plate, well) and a list of the contents of wells is in Supplementary Table S1. (ZIP) [file pone.0052673.s005.zip › suppl fig 5G type 35/Plate01/pm01b09.gif]

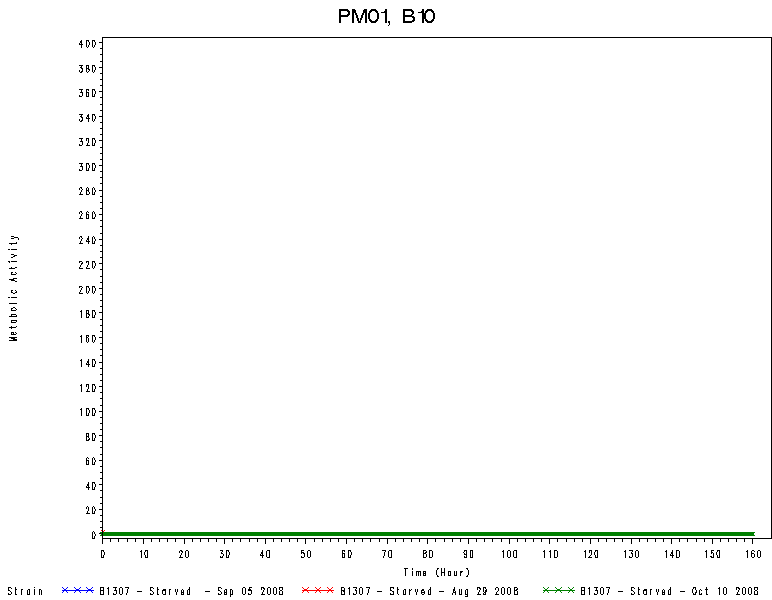

Supplement: Figure S5 — Kinetic curves for all PM plates with M. bovis Type 35 strains. Figures S1 to S5 were generated in SAS using a GPLOT procedure, as described in the methods. Each figure is a Zip file containing plots of Omnilog units (due to dye reduction) against time (0 to 168 h) for all wells of each of the six 96 well plates. Each well is identified by (plate, well) and a list of the contents of wells is in Supplementary Table S1. (ZIP) [file pone.0052673.s005.zip › suppl fig 5G type 35/Plate01/pm01b10.gif]

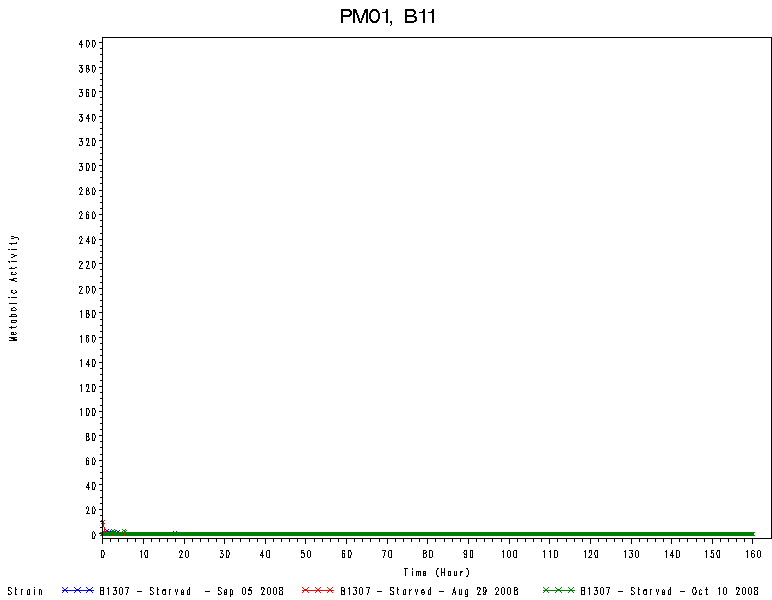

Supplement: Figure S5 — Kinetic curves for all PM plates with M. bovis Type 35 strains. Figures S1 to S5 were generated in SAS using a GPLOT procedure, as described in the methods. Each figure is a Zip file containing plots of Omnilog units (due to dye reduction) against time (0 to 168 h) for all wells of each of the six 96 well plates. Each well is identified by (plate, well) and a list of the contents of wells is in Supplementary Table S1. (ZIP) [file pone.0052673.s005.zip › suppl fig 5G type 35/Plate01/pm01b11.gif]

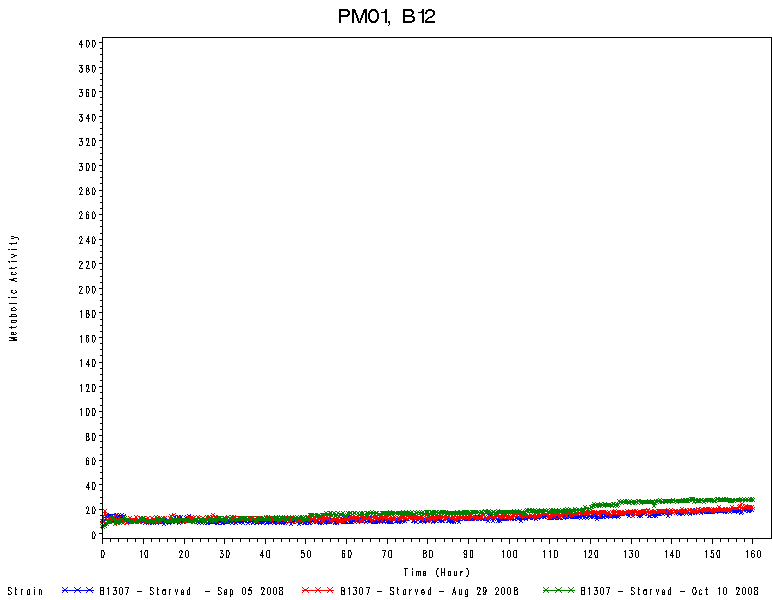

Supplement: Figure S5 — Kinetic curves for all PM plates with M. bovis Type 35 strains. Figures S1 to S5 were generated in SAS using a GPLOT procedure, as described in the methods. Each figure is a Zip file containing plots of Omnilog units (due to dye reduction) against time (0 to 168 h) for all wells of each of the six 96 well plates. Each well is identified by (plate, well) and a list of the contents of wells is in Supplementary Table S1. (ZIP) [file pone.0052673.s005.zip › suppl fig 5G type 35/Plate01/pm01b12.gif]

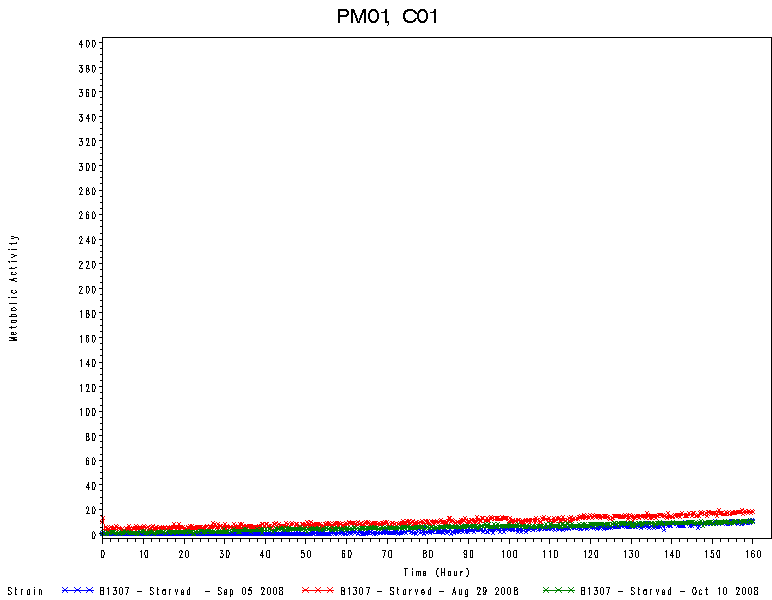

Supplement: Figure S5 — Kinetic curves for all PM plates with M. bovis Type 35 strains. Figures S1 to S5 were generated in SAS using a GPLOT procedure, as described in the methods. Each figure is a Zip file containing plots of Omnilog units (due to dye reduction) against time (0 to 168 h) for all wells of each of the six 96 well plates. Each well is identified by (plate, well) and a list of the contents of wells is in Supplementary Table S1. (ZIP) [file pone.0052673.s005.zip › suppl fig 5G type 35/Plate01/pm01c01.gif]

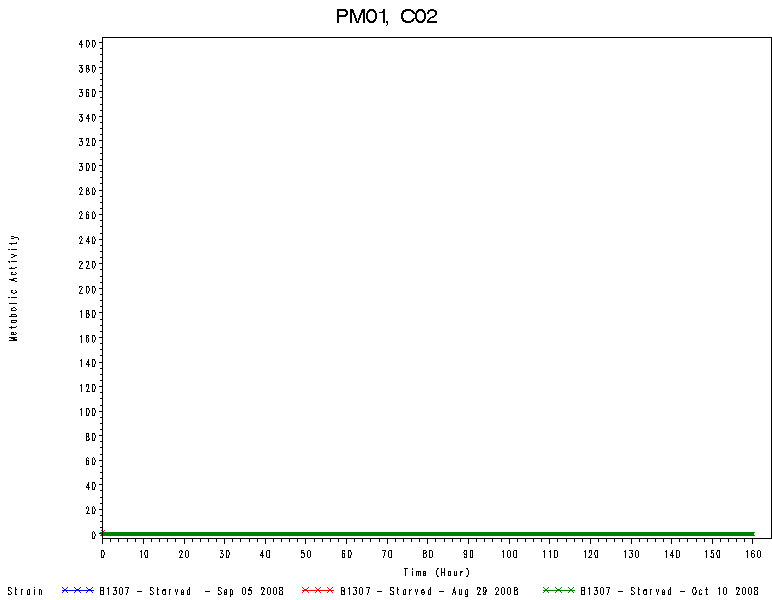

Supplement: Figure S5 — Kinetic curves for all PM plates with M. bovis Type 35 strains. Figures S1 to S5 were generated in SAS using a GPLOT procedure, as described in the methods. Each figure is a Zip file containing plots of Omnilog units (due to dye reduction) against time (0 to 168 h) for all wells of each of the six 96 well plates. Each well is identified by (plate, well) and a list of the contents of wells is in Supplementary Table S1. (ZIP) [file pone.0052673.s005.zip › suppl fig 5G type 35/Plate01/pm01c02.gif]

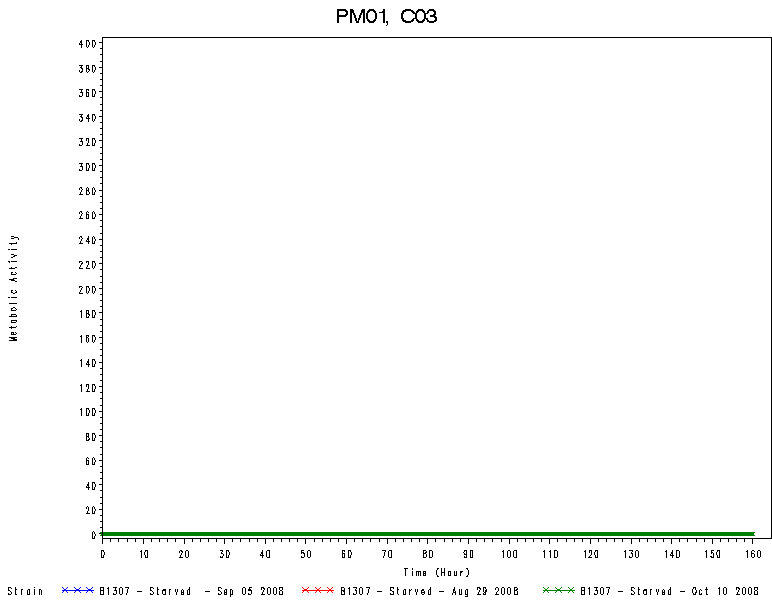

Supplement: Figure S5 — Kinetic curves for all PM plates with M. bovis Type 35 strains. Figures S1 to S5 were generated in SAS using a GPLOT procedure, as described in the methods. Each figure is a Zip file containing plots of Omnilog units (due to dye reduction) against time (0 to 168 h) for all wells of each of the six 96 well plates. Each well is identified by (plate, well) and a list of the contents of wells is in Supplementary Table S1. (ZIP) [file pone.0052673.s005.zip › suppl fig 5G type 35/Plate01/pm01c03.gif]

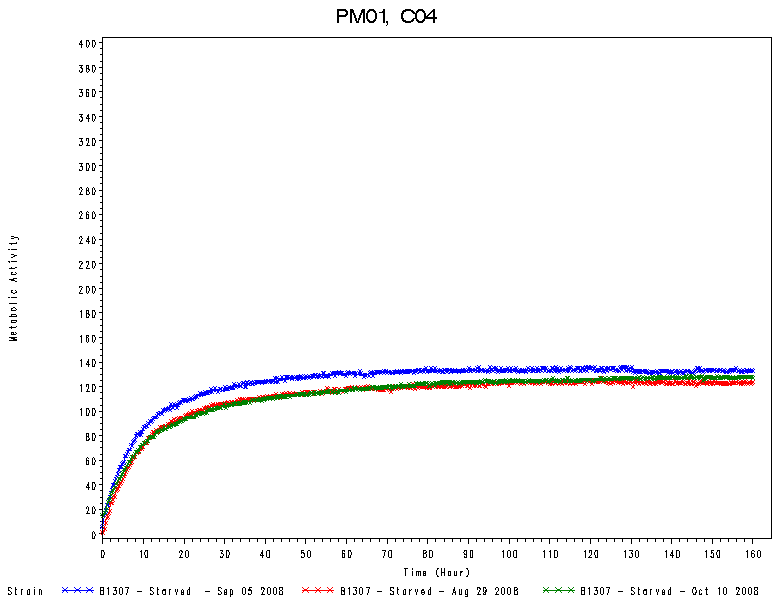

Supplement: Figure S5 — Kinetic curves for all PM plates with M. bovis Type 35 strains. Figures S1 to S5 were generated in SAS using a GPLOT procedure, as described in the methods. Each figure is a Zip file containing plots of Omnilog units (due to dye reduction) against time (0 to 168 h) for all wells of each of the six 96 well plates. Each well is identified by (plate, well) and a list of the contents of wells is in Supplementary Table S1. (ZIP) [file pone.0052673.s005.zip › suppl fig 5G type 35/Plate01/pm01c04.gif]

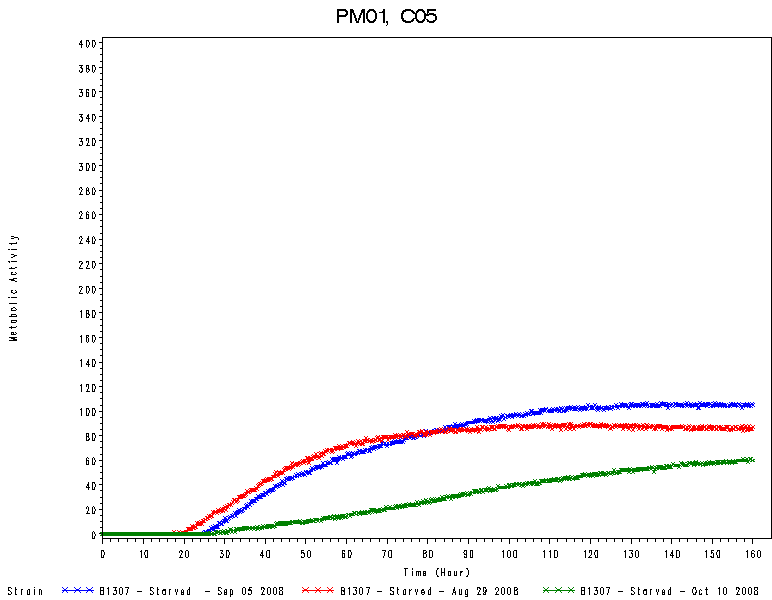

Supplement: Figure S5 — Kinetic curves for all PM plates with M. bovis Type 35 strains. Figures S1 to S5 were generated in SAS using a GPLOT procedure, as described in the methods. Each figure is a Zip file containing plots of Omnilog units (due to dye reduction) against time (0 to 168 h) for all wells of each of the six 96 well plates. Each well is identified by (plate, well) and a list of the contents of wells is in Supplementary Table S1. (ZIP) [file pone.0052673.s005.zip › suppl fig 5G type 35/Plate01/pm01c05.gif]

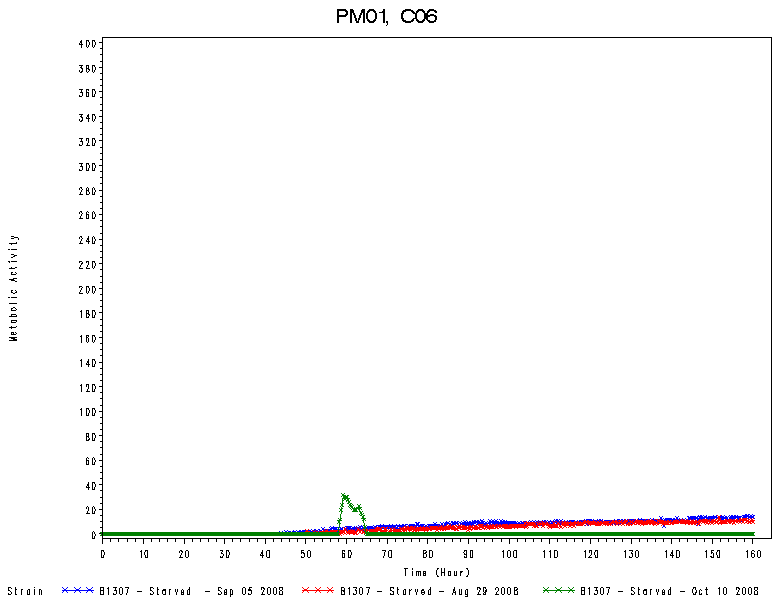

Supplement: Figure S5 — Kinetic curves for all PM plates with M. bovis Type 35 strains. Figures S1 to S5 were generated in SAS using a GPLOT procedure, as described in the methods. Each figure is a Zip file containing plots of Omnilog units (due to dye reduction) against time (0 to 168 h) for all wells of each of the six 96 well plates. Each well is identified by (plate, well) and a list of the contents of wells is in Supplementary Table S1. (ZIP) [file pone.0052673.s005.zip › suppl fig 5G type 35/Plate01/pm01c06.gif]

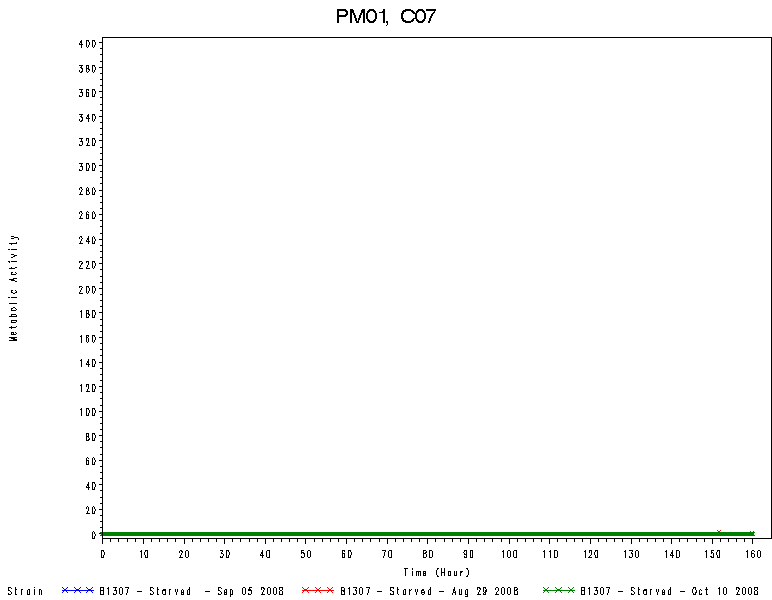

Supplement: Figure S5 — Kinetic curves for all PM plates with M. bovis Type 35 strains. Figures S1 to S5 were generated in SAS using a GPLOT procedure, as described in the methods. Each figure is a Zip file containing plots of Omnilog units (due to dye reduction) against time (0 to 168 h) for all wells of each of the six 96 well plates. Each well is identified by (plate, well) and a list of the contents of wells is in Supplementary Table S1. (ZIP) [file pone.0052673.s005.zip › suppl fig 5G type 35/Plate01/pm01c07.gif]

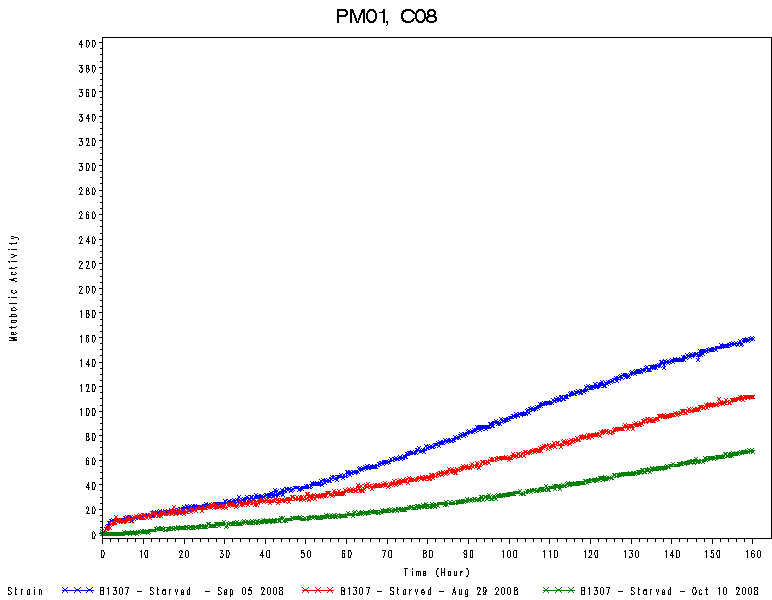

Supplement: Figure S5 — Kinetic curves for all PM plates with M. bovis Type 35 strains. Figures S1 to S5 were generated in SAS using a GPLOT procedure, as described in the methods. Each figure is a Zip file containing plots of Omnilog units (due to dye reduction) against time (0 to 168 h) for all wells of each of the six 96 well plates. Each well is identified by (plate, well) and a list of the contents of wells is in Supplementary Table S1. (ZIP) [file pone.0052673.s005.zip › suppl fig 5G type 35/Plate01/pm01c08.gif]

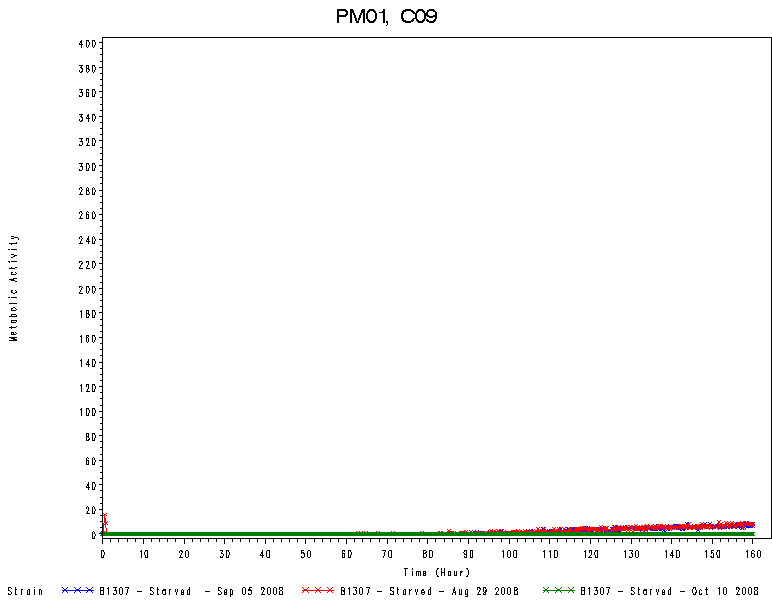

Supplement: Figure S5 — Kinetic curves for all PM plates with M. bovis Type 35 strains. Figures S1 to S5 were generated in SAS using a GPLOT procedure, as described in the methods. Each figure is a Zip file containing plots of Omnilog units (due to dye reduction) against time (0 to 168 h) for all wells of each of the six 96 well plates. Each well is identified by (plate, well) and a list of the contents of wells is in Supplementary Table S1. (ZIP) [file pone.0052673.s005.zip › suppl fig 5G type 35/Plate01/pm01c09.gif]

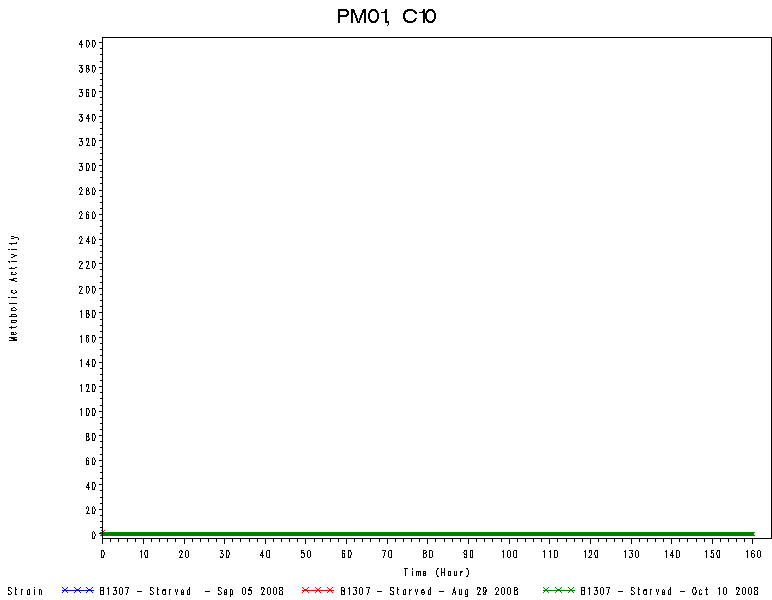

Supplement: Figure S5 — Kinetic curves for all PM plates with M. bovis Type 35 strains. Figures S1 to S5 were generated in SAS using a GPLOT procedure, as described in the methods. Each figure is a Zip file containing plots of Omnilog units (due to dye reduction) against time (0 to 168 h) for all wells of each of the six 96 well plates. Each well is identified by (plate, well) and a list of the contents of wells is in Supplementary Table S1. (ZIP) [file pone.0052673.s005.zip › suppl fig 5G type 35/Plate01/pm01c10.gif]

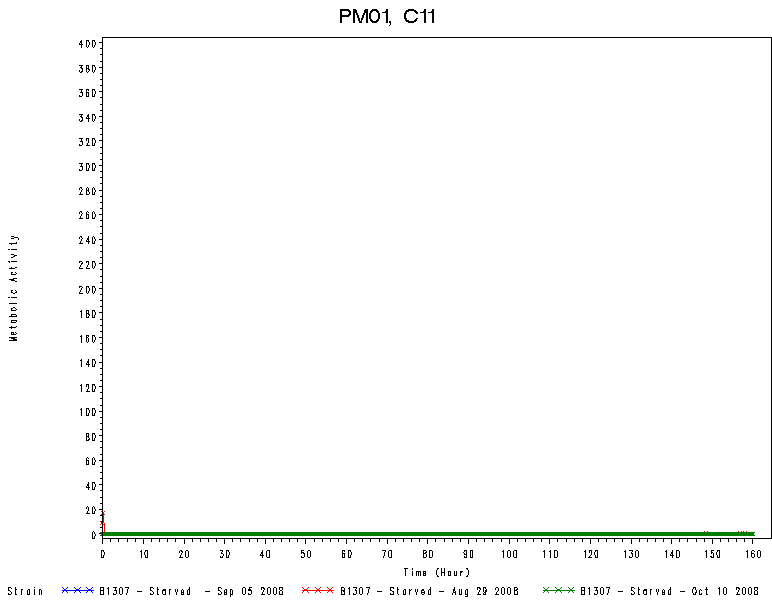

Supplement: Figure S5 — Kinetic curves for all PM plates with M. bovis Type 35 strains. Figures S1 to S5 were generated in SAS using a GPLOT procedure, as described in the methods. Each figure is a Zip file containing plots of Omnilog units (due to dye reduction) against time (0 to 168 h) for all wells of each of the six 96 well plates. Each well is identified by (plate, well) and a list of the contents of wells is in Supplementary Table S1. (ZIP) [file pone.0052673.s005.zip › suppl fig 5G type 35/Plate01/pm01c11.gif]

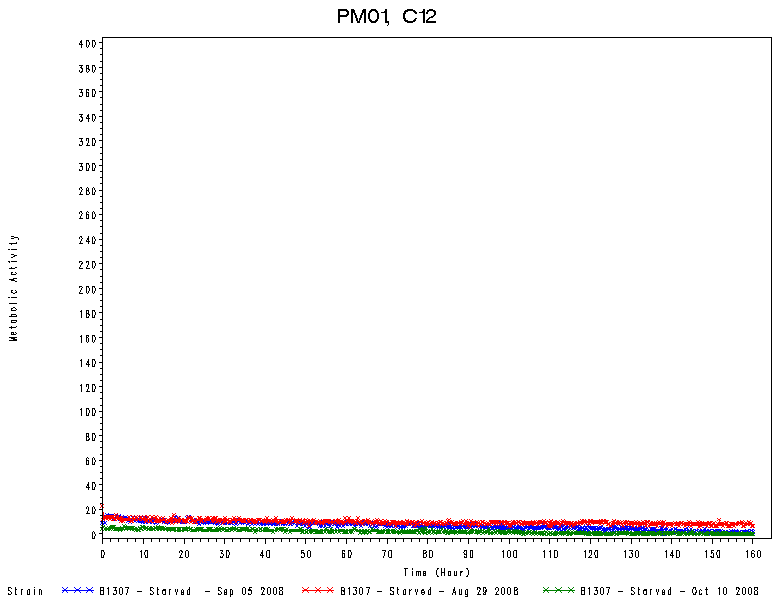

Supplement: Figure S5 — Kinetic curves for all PM plates with M. bovis Type 35 strains. Figures S1 to S5 were generated in SAS using a GPLOT procedure, as described in the methods. Each figure is a Zip file containing plots of Omnilog units (due to dye reduction) against time (0 to 168 h) for all wells of each of the six 96 well plates. Each well is identified by (plate, well) and a list of the contents of wells is in Supplementary Table S1. (ZIP) [file pone.0052673.s005.zip › suppl fig 5G type 35/Plate01/pm01c12.gif]

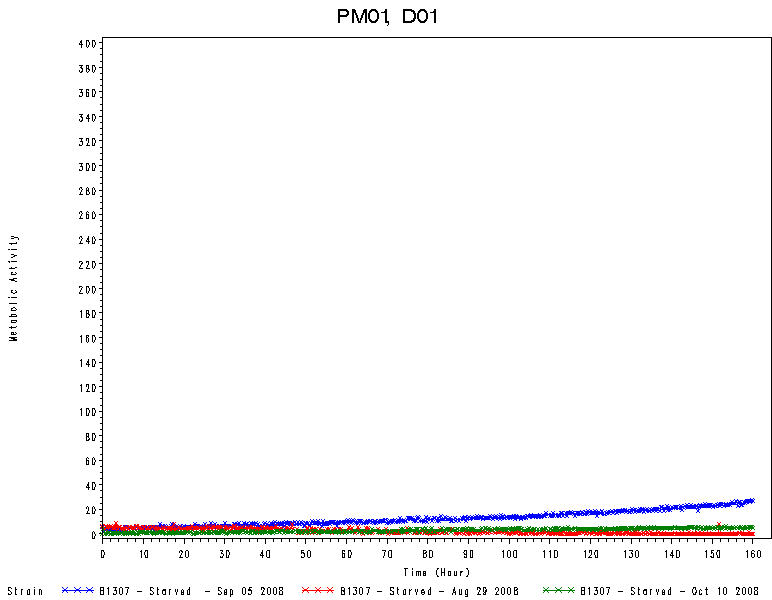

Supplement: Figure S5 — Kinetic curves for all PM plates with M. bovis Type 35 strains. Figures S1 to S5 were generated in SAS using a GPLOT procedure, as described in the methods. Each figure is a Zip file containing plots of Omnilog units (due to dye reduction) against time (0 to 168 h) for all wells of each of the six 96 well plates. Each well is identified by (plate, well) and a list of the contents of wells is in Supplementary Table S1. (ZIP) [file pone.0052673.s005.zip › suppl fig 5G type 35/Plate01/pm01d01.gif]

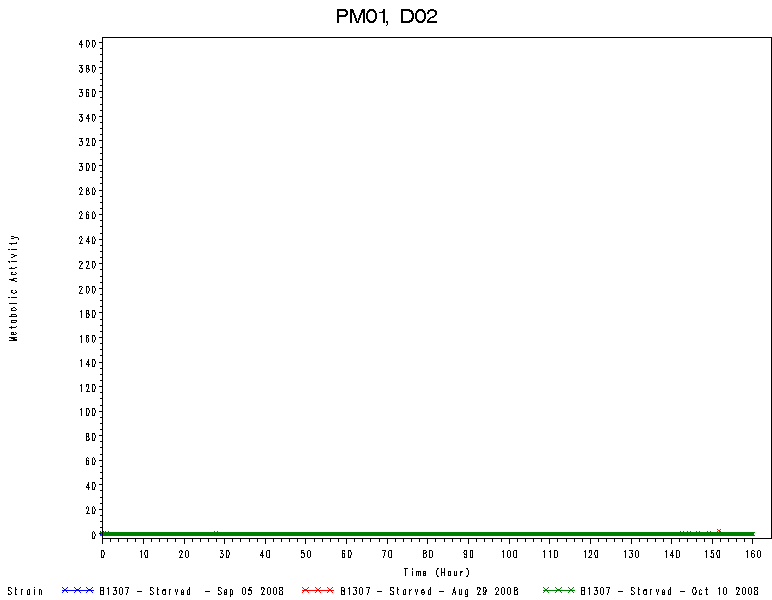

Supplement: Figure S5 — Kinetic curves for all PM plates with M. bovis Type 35 strains. Figures S1 to S5 were generated in SAS using a GPLOT procedure, as described in the methods. Each figure is a Zip file containing plots of Omnilog units (due to dye reduction) against time (0 to 168 h) for all wells of each of the six 96 well plates. Each well is identified by (plate, well) and a list of the contents of wells is in Supplementary Table S1. (ZIP) [file pone.0052673.s005.zip › suppl fig 5G type 35/Plate01/pm01d02.gif]

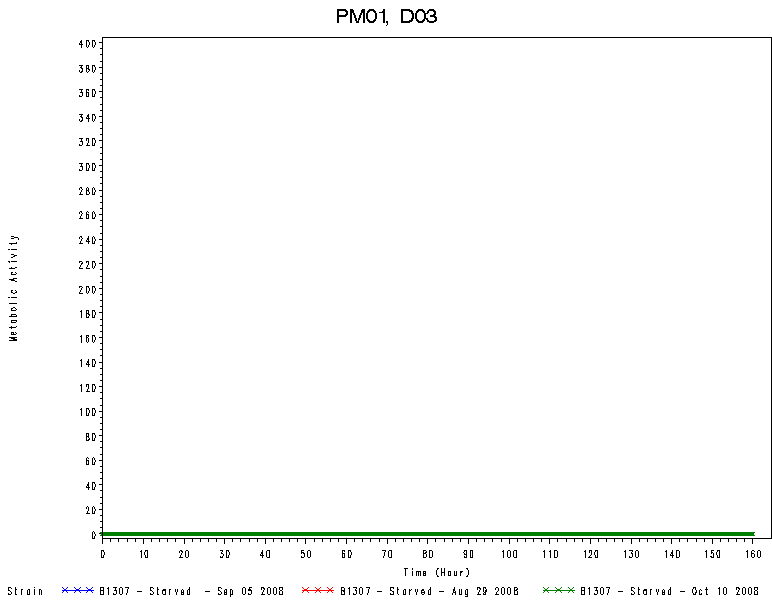

Supplement: Figure S5 — Kinetic curves for all PM plates with M. bovis Type 35 strains. Figures S1 to S5 were generated in SAS using a GPLOT procedure, as described in the methods. Each figure is a Zip file containing plots of Omnilog units (due to dye reduction) against time (0 to 168 h) for all wells of each of the six 96 well plates. Each well is identified by (plate, well) and a list of the contents of wells is in Supplementary Table S1. (ZIP) [file pone.0052673.s005.zip › suppl fig 5G type 35/Plate01/pm01d03.gif]

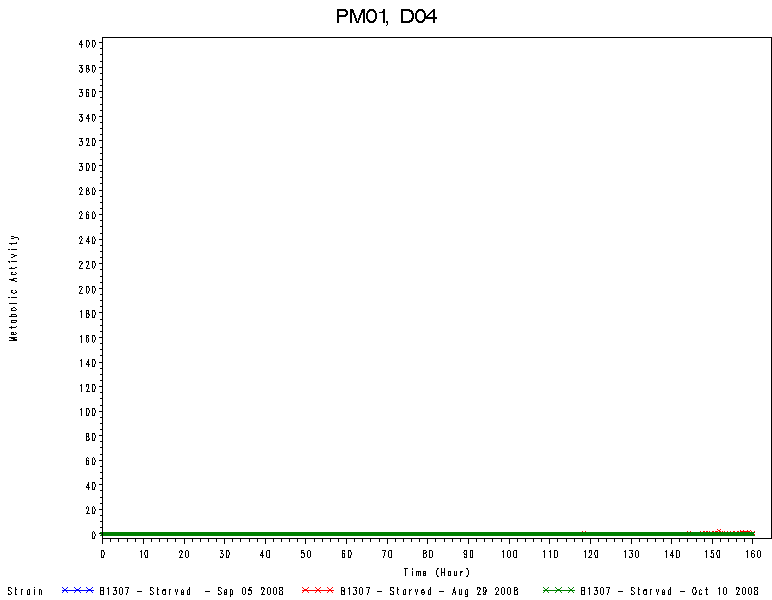

Supplement: Figure S5 — Kinetic curves for all PM plates with M. bovis Type 35 strains. Figures S1 to S5 were generated in SAS using a GPLOT procedure, as described in the methods. Each figure is a Zip file containing plots of Omnilog units (due to dye reduction) against time (0 to 168 h) for all wells of each of the six 96 well plates. Each well is identified by (plate, well) and a list of the contents of wells is in Supplementary Table S1. (ZIP) [file pone.0052673.s005.zip › suppl fig 5G type 35/Plate01/pm01d04.gif]

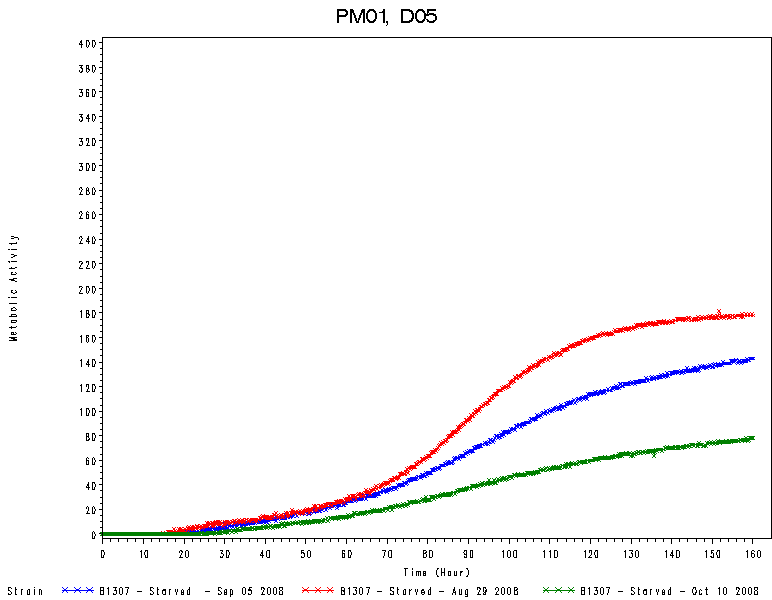

Supplement: Figure S5 — Kinetic curves for all PM plates with M. bovis Type 35 strains. Figures S1 to S5 were generated in SAS using a GPLOT procedure, as described in the methods. Each figure is a Zip file containing plots of Omnilog units (due to dye reduction) against time (0 to 168 h) for all wells of each of the six 96 well plates. Each well is identified by (plate, well) and a list of the contents of wells is in Supplementary Table S1. (ZIP) [file pone.0052673.s005.zip › suppl fig 5G type 35/Plate01/pm01d05.gif]

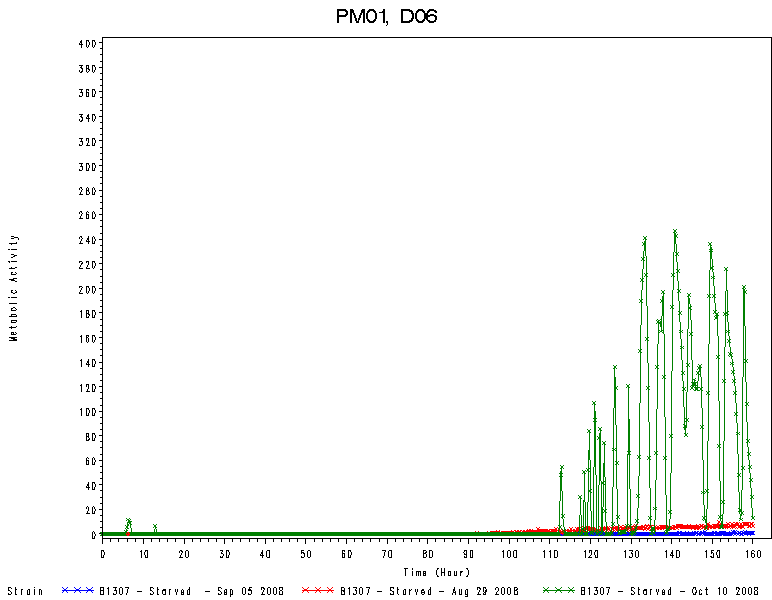

Supplement: Figure S5 — Kinetic curves for all PM plates with M. bovis Type 35 strains. Figures S1 to S5 were generated in SAS using a GPLOT procedure, as described in the methods. Each figure is a Zip file containing plots of Omnilog units (due to dye reduction) against time (0 to 168 h) for all wells of each of the six 96 well plates. Each well is identified by (plate, well) and a list of the contents of wells is in Supplementary Table S1. (ZIP) [file pone.0052673.s005.zip › suppl fig 5G type 35/Plate01/pm01d06.gif]

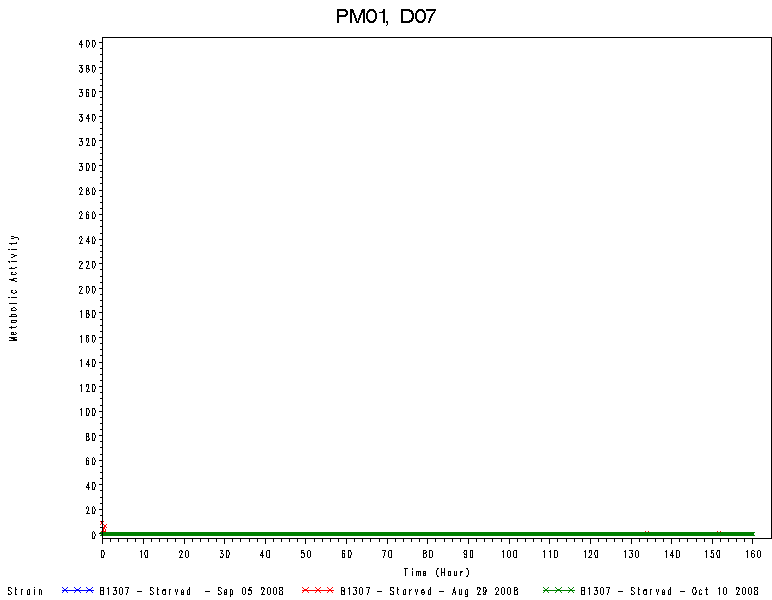

Supplement: Figure S5 — Kinetic curves for all PM plates with M. bovis Type 35 strains. Figures S1 to S5 were generated in SAS using a GPLOT procedure, as described in the methods. Each figure is a Zip file containing plots of Omnilog units (due to dye reduction) against time (0 to 168 h) for all wells of each of the six 96 well plates. Each well is identified by (plate, well) and a list of the contents of wells is in Supplementary Table S1. (ZIP) [file pone.0052673.s005.zip › suppl fig 5G type 35/Plate01/pm01d07.gif]

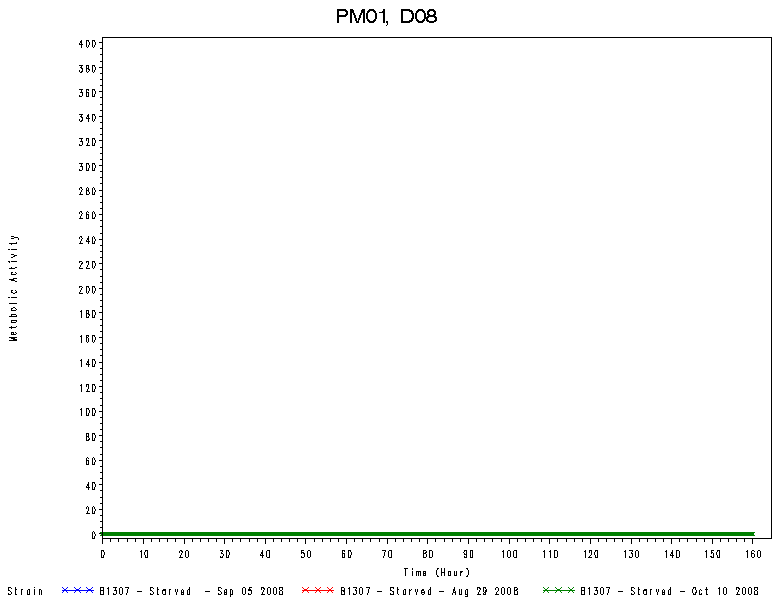

Supplement: Figure S5 — Kinetic curves for all PM plates with M. bovis Type 35 strains. Figures S1 to S5 were generated in SAS using a GPLOT procedure, as described in the methods. Each figure is a Zip file containing plots of Omnilog units (due to dye reduction) against time (0 to 168 h) for all wells of each of the six 96 well plates. Each well is identified by (plate, well) and a list of the contents of wells is in Supplementary Table S1. (ZIP) [file pone.0052673.s005.zip › suppl fig 5G type 35/Plate01/pm01d08.gif]

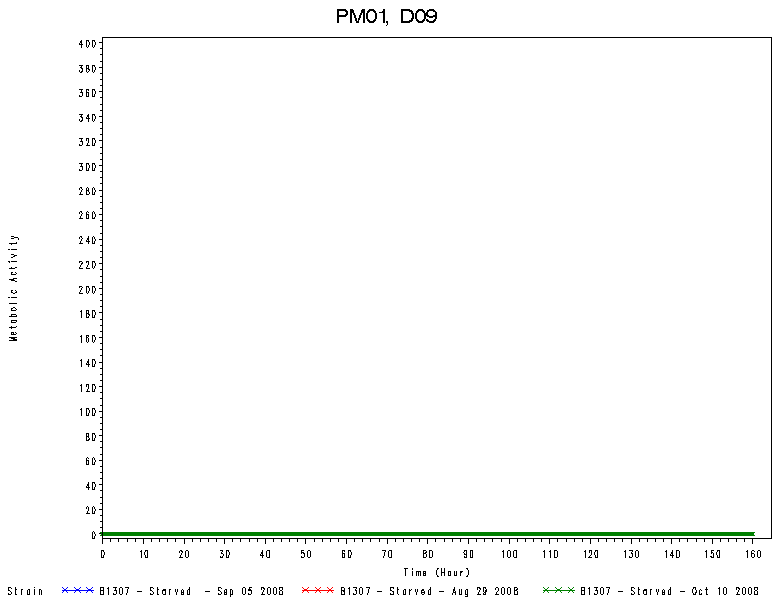

Supplement: Figure S5 — Kinetic curves for all PM plates with M. bovis Type 35 strains. Figures S1 to S5 were generated in SAS using a GPLOT procedure, as described in the methods. Each figure is a Zip file containing plots of Omnilog units (due to dye reduction) against time (0 to 168 h) for all wells of each of the six 96 well plates. Each well is identified by (plate, well) and a list of the contents of wells is in Supplementary Table S1. (ZIP) [file pone.0052673.s005.zip › suppl fig 5G type 35/Plate01/pm01d09.gif]

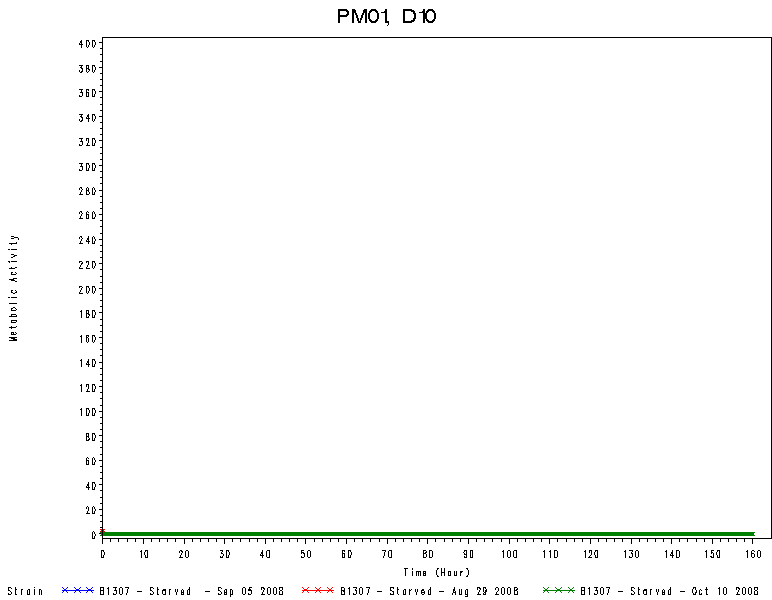

Supplement: Figure S5 — Kinetic curves for all PM plates with M. bovis Type 35 strains. Figures S1 to S5 were generated in SAS using a GPLOT procedure, as described in the methods. Each figure is a Zip file containing plots of Omnilog units (due to dye reduction) against time (0 to 168 h) for all wells of each of the six 96 well plates. Each well is identified by (plate, well) and a list of the contents of wells is in Supplementary Table S1. (ZIP) [file pone.0052673.s005.zip › suppl fig 5G type 35/Plate01/pm01d10.gif]

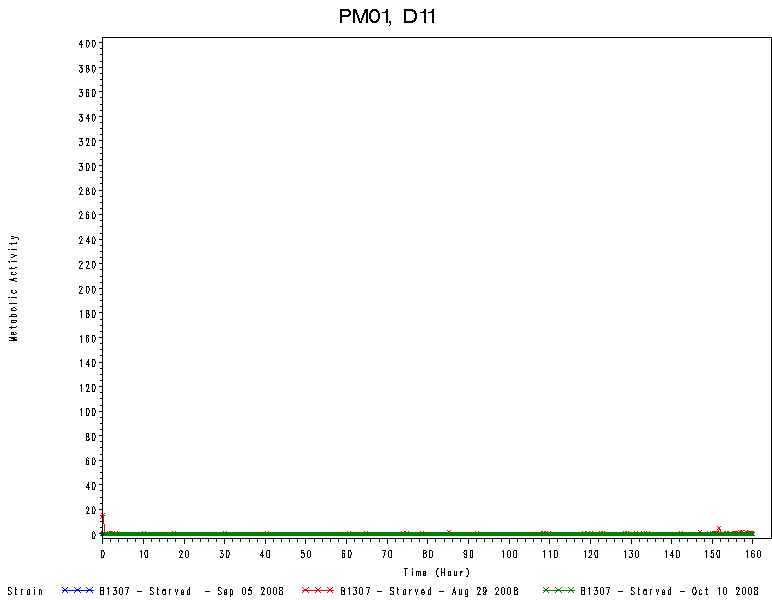

Supplement: Figure S5 — Kinetic curves for all PM plates with M. bovis Type 35 strains. Figures S1 to S5 were generated in SAS using a GPLOT procedure, as described in the methods. Each figure is a Zip file containing plots of Omnilog units (due to dye reduction) against time (0 to 168 h) for all wells of each of the six 96 well plates. Each well is identified by (plate, well) and a list of the contents of wells is in Supplementary Table S1. (ZIP) [file pone.0052673.s005.zip › suppl fig 5G type 35/Plate01/pm01d11.gif]

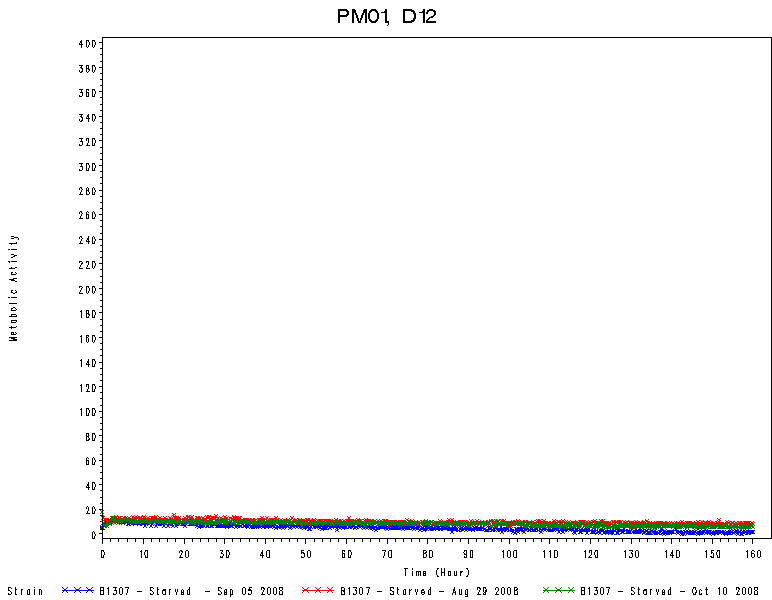

Supplement: Figure S5 — Kinetic curves for all PM plates with M. bovis Type 35 strains. Figures S1 to S5 were generated in SAS using a GPLOT procedure, as described in the methods. Each figure is a Zip file containing plots of Omnilog units (due to dye reduction) against time (0 to 168 h) for all wells of each of the six 96 well plates. Each well is identified by (plate, well) and a list of the contents of wells is in Supplementary Table S1. (ZIP) [file pone.0052673.s005.zip › suppl fig 5G type 35/Plate01/pm01d12.gif]

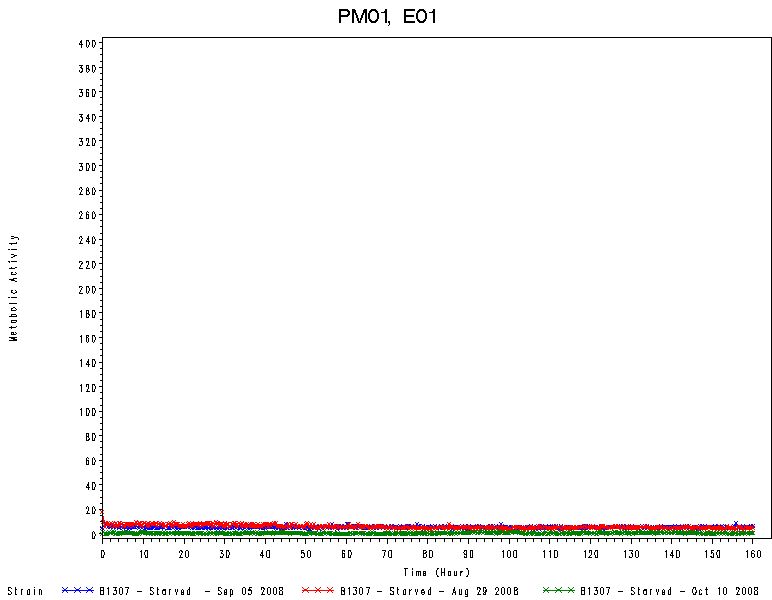

Supplement: Figure S5 — Kinetic curves for all PM plates with M. bovis Type 35 strains. Figures S1 to S5 were generated in SAS using a GPLOT procedure, as described in the methods. Each figure is a Zip file containing plots of Omnilog units (due to dye reduction) against time (0 to 168 h) for all wells of each of the six 96 well plates. Each well is identified by (plate, well) and a list of the contents of wells is in Supplementary Table S1. (ZIP) [file pone.0052673.s005.zip › suppl fig 5G type 35/Plate01/pm01e01.gif]

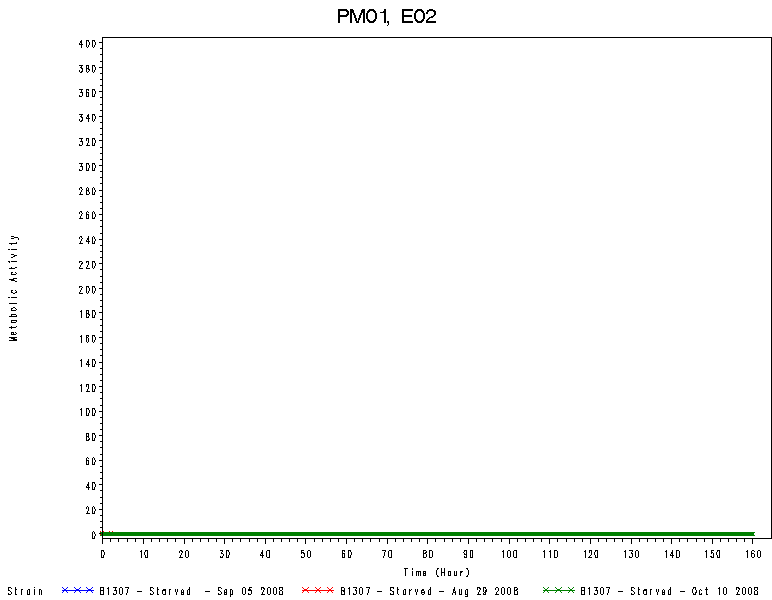

Supplement: Figure S5 — Kinetic curves for all PM plates with M. bovis Type 35 strains. Figures S1 to S5 were generated in SAS using a GPLOT procedure, as described in the methods. Each figure is a Zip file containing plots of Omnilog units (due to dye reduction) against time (0 to 168 h) for all wells of each of the six 96 well plates. Each well is identified by (plate, well) and a list of the contents of wells is in Supplementary Table S1. (ZIP) [file pone.0052673.s005.zip › suppl fig 5G type 35/Plate01/pm01e02.gif]

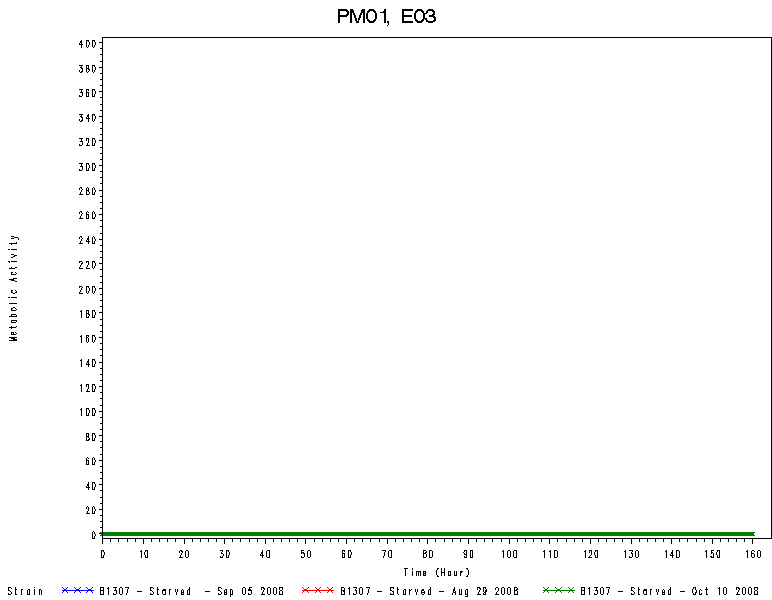

Supplement: Figure S5 — Kinetic curves for all PM plates with M. bovis Type 35 strains. Figures S1 to S5 were generated in SAS using a GPLOT procedure, as described in the methods. Each figure is a Zip file containing plots of Omnilog units (due to dye reduction) against time (0 to 168 h) for all wells of each of the six 96 well plates. Each well is identified by (plate, well) and a list of the contents of wells is in Supplementary Table S1. (ZIP) [file pone.0052673.s005.zip › suppl fig 5G type 35/Plate01/pm01e03.gif]

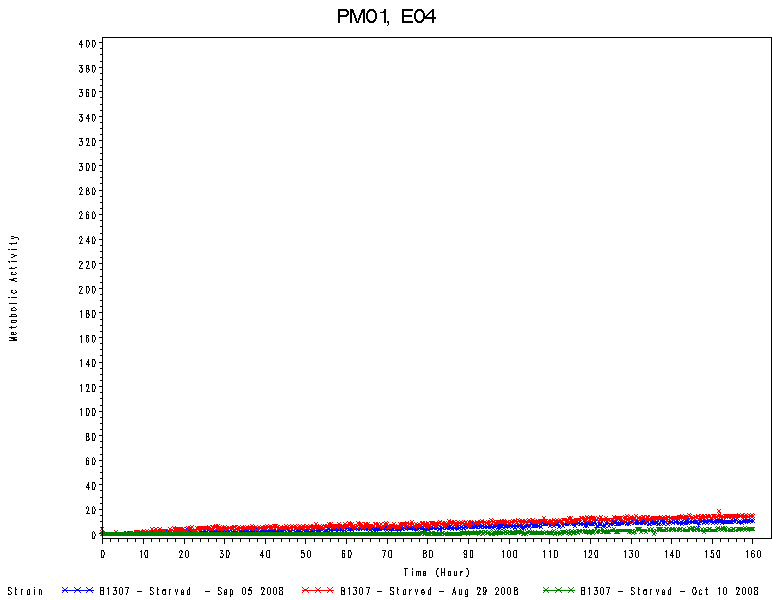

Supplement: Figure S5 — Kinetic curves for all PM plates with M. bovis Type 35 strains. Figures S1 to S5 were generated in SAS using a GPLOT procedure, as described in the methods. Each figure is a Zip file containing plots of Omnilog units (due to dye reduction) against time (0 to 168 h) for all wells of each of the six 96 well plates. Each well is identified by (plate, well) and a list of the contents of wells is in Supplementary Table S1. (ZIP) [file pone.0052673.s005.zip › suppl fig 5G type 35/Plate01/pm01e04.gif]

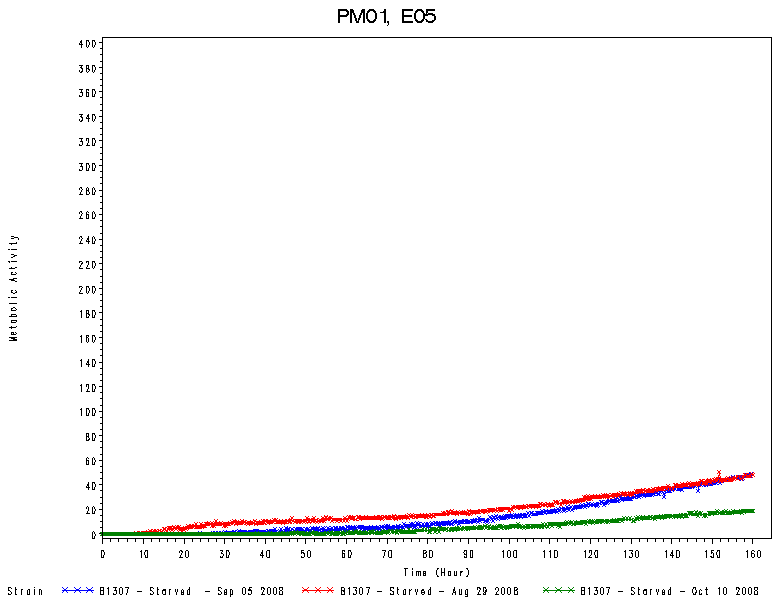

Supplement: Figure S5 — Kinetic curves for all PM plates with M. bovis Type 35 strains. Figures S1 to S5 were generated in SAS using a GPLOT procedure, as described in the methods. Each figure is a Zip file containing plots of Omnilog units (due to dye reduction) against time (0 to 168 h) for all wells of each of the six 96 well plates. Each well is identified by (plate, well) and a list of the contents of wells is in Supplementary Table S1. (ZIP) [file pone.0052673.s005.zip › suppl fig 5G type 35/Plate01/pm01e05.gif]

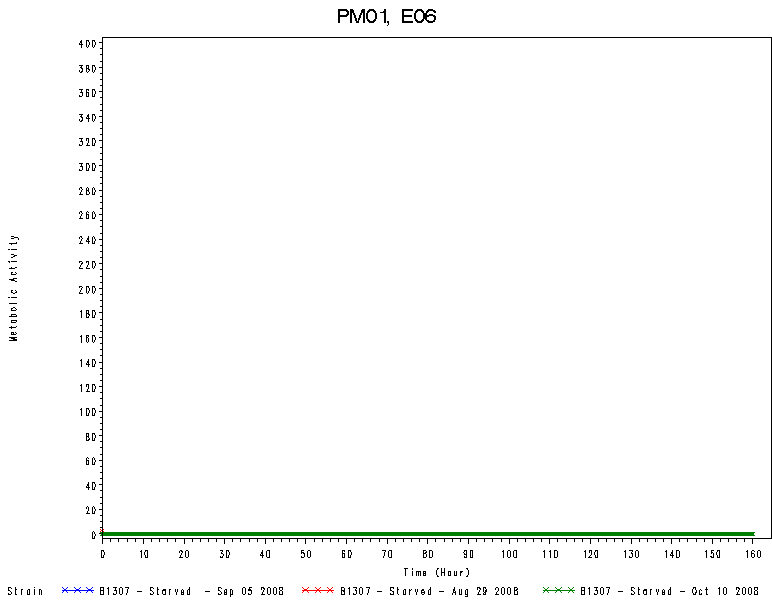

Supplement: Figure S5 — Kinetic curves for all PM plates with M. bovis Type 35 strains. Figures S1 to S5 were generated in SAS using a GPLOT procedure, as described in the methods. Each figure is a Zip file containing plots of Omnilog units (due to dye reduction) against time (0 to 168 h) for all wells of each of the six 96 well plates. Each well is identified by (plate, well) and a list of the contents of wells is in Supplementary Table S1. (ZIP) [file pone.0052673.s005.zip › suppl fig 5G type 35/Plate01/pm01e06.gif]

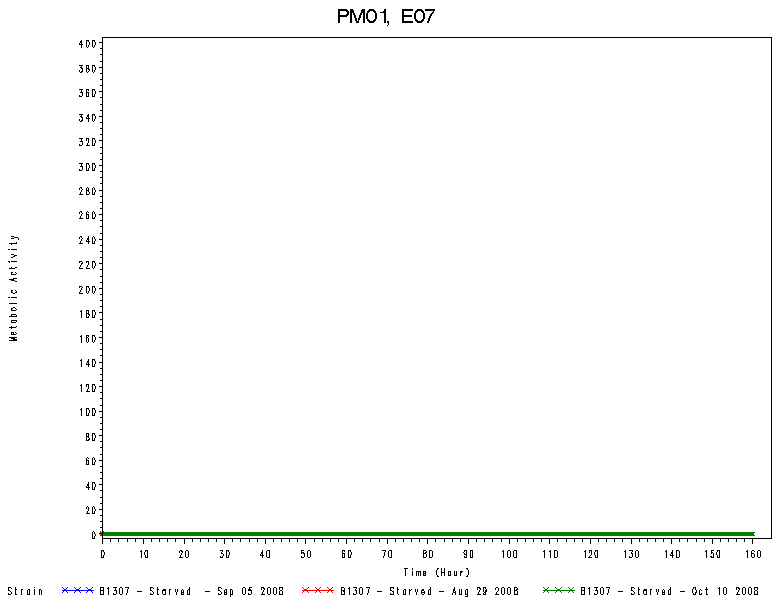

Supplement: Figure S5 — Kinetic curves for all PM plates with M. bovis Type 35 strains. Figures S1 to S5 were generated in SAS using a GPLOT procedure, as described in the methods. Each figure is a Zip file containing plots of Omnilog units (due to dye reduction) against time (0 to 168 h) for all wells of each of the six 96 well plates. Each well is identified by (plate, well) and a list of the contents of wells is in Supplementary Table S1. (ZIP) [file pone.0052673.s005.zip › suppl fig 5G type 35/Plate01/pm01e07.gif]

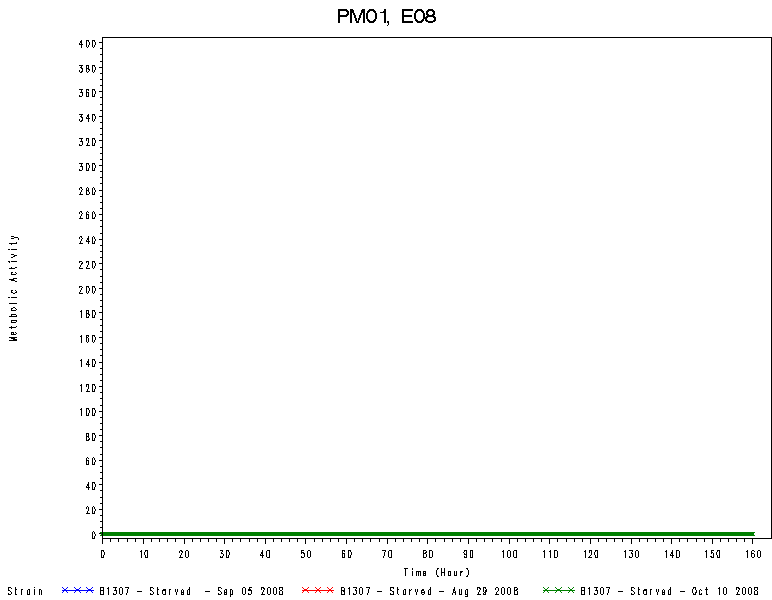

Supplement: Figure S5 — Kinetic curves for all PM plates with M. bovis Type 35 strains. Figures S1 to S5 were generated in SAS using a GPLOT procedure, as described in the methods. Each figure is a Zip file containing plots of Omnilog units (due to dye reduction) against time (0 to 168 h) for all wells of each of the six 96 well plates. Each well is identified by (plate, well) and a list of the contents of wells is in Supplementary Table S1. (ZIP) [file pone.0052673.s005.zip › suppl fig 5G type 35/Plate01/pm01e08.gif]

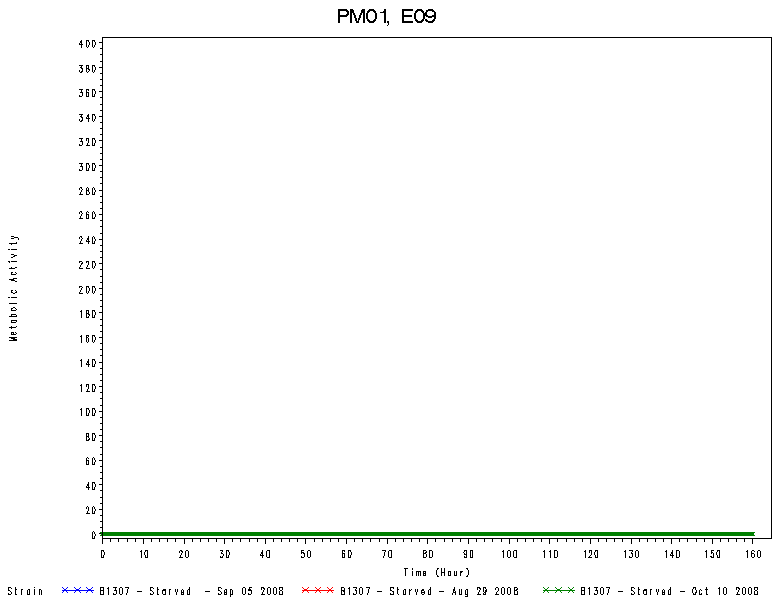

Supplement: Figure S5 — Kinetic curves for all PM plates with M. bovis Type 35 strains. Figures S1 to S5 were generated in SAS using a GPLOT procedure, as described in the methods. Each figure is a Zip file containing plots of Omnilog units (due to dye reduction) against time (0 to 168 h) for all wells of each of the six 96 well plates. Each well is identified by (plate, well) and a list of the contents of wells is in Supplementary Table S1. (ZIP) [file pone.0052673.s005.zip › suppl fig 5G type 35/Plate01/pm01e09.gif]

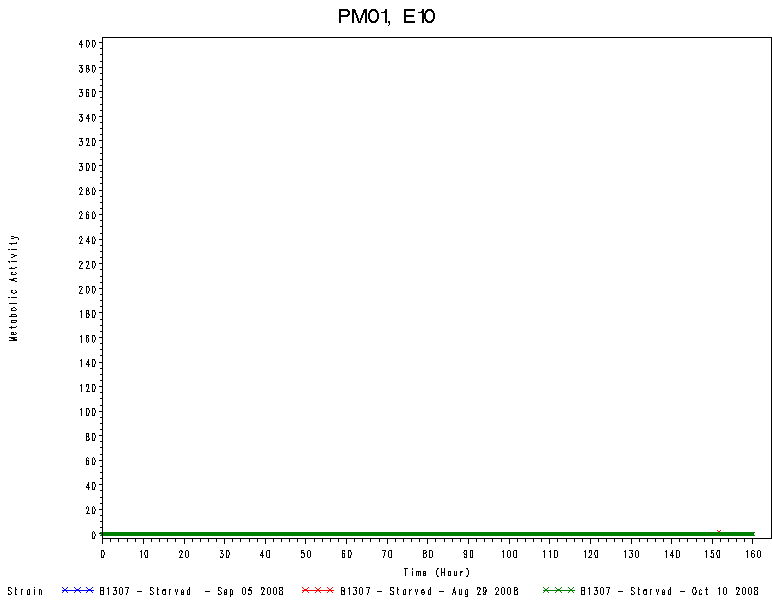

Supplement: Figure S5 — Kinetic curves for all PM plates with M. bovis Type 35 strains. Figures S1 to S5 were generated in SAS using a GPLOT procedure, as described in the methods. Each figure is a Zip file containing plots of Omnilog units (due to dye reduction) against time (0 to 168 h) for all wells of each of the six 96 well plates. Each well is identified by (plate, well) and a list of the contents of wells is in Supplementary Table S1. (ZIP) [file pone.0052673.s005.zip › suppl fig 5G type 35/Plate01/pm01e10.gif]

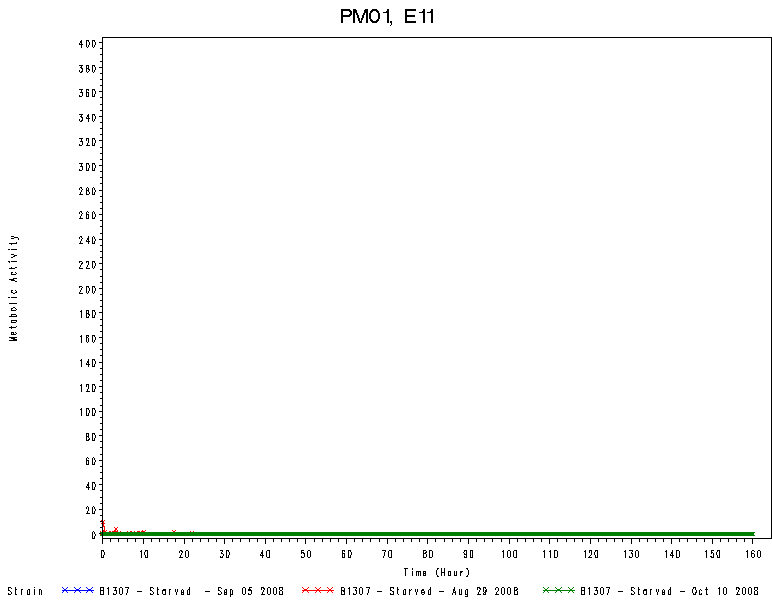

Supplement: Figure S5 — Kinetic curves for all PM plates with M. bovis Type 35 strains. Figures S1 to S5 were generated in SAS using a GPLOT procedure, as described in the methods. Each figure is a Zip file containing plots of Omnilog units (due to dye reduction) against time (0 to 168 h) for all wells of each of the six 96 well plates. Each well is identified by (plate, well) and a list of the contents of wells is in Supplementary Table S1. (ZIP) [file pone.0052673.s005.zip › suppl fig 5G type 35/Plate01/pm01e11.gif]

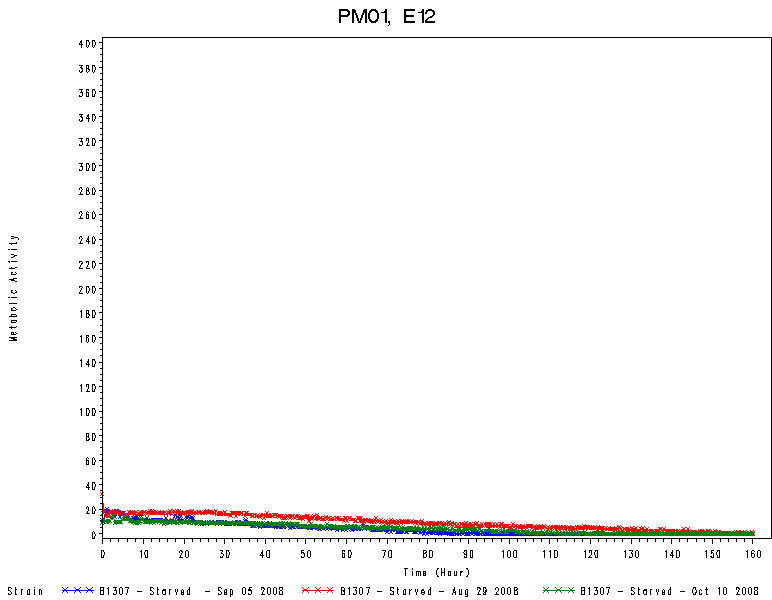

Supplement: Figure S5 — Kinetic curves for all PM plates with M. bovis Type 35 strains. Figures S1 to S5 were generated in SAS using a GPLOT procedure, as described in the methods. Each figure is a Zip file containing plots of Omnilog units (due to dye reduction) against time (0 to 168 h) for all wells of each of the six 96 well plates. Each well is identified by (plate, well) and a list of the contents of wells is in Supplementary Table S1. (ZIP) [file pone.0052673.s005.zip › suppl fig 5G type 35/Plate01/pm01e12.gif]

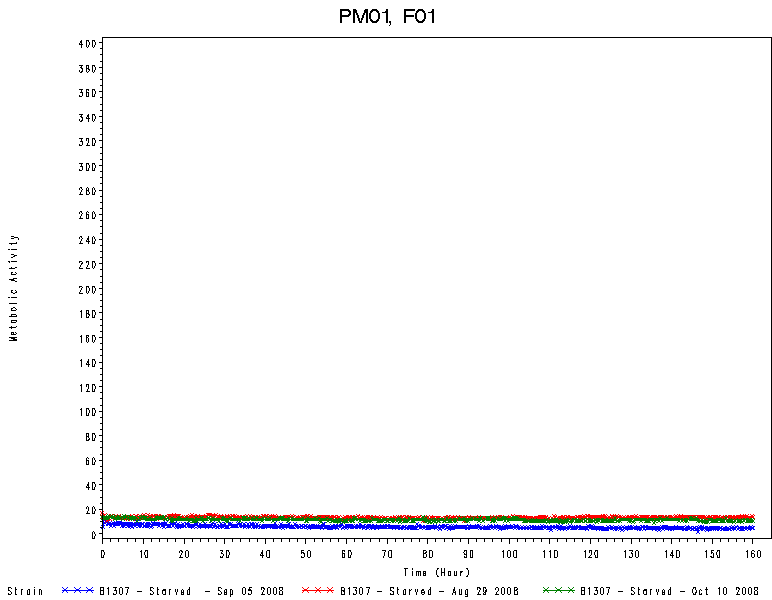

Supplement: Figure S5 — Kinetic curves for all PM plates with M. bovis Type 35 strains. Figures S1 to S5 were generated in SAS using a GPLOT procedure, as described in the methods. Each figure is a Zip file containing plots of Omnilog units (due to dye reduction) against time (0 to 168 h) for all wells of each of the six 96 well plates. Each well is identified by (plate, well) and a list of the contents of wells is in Supplementary Table S1. (ZIP) [file pone.0052673.s005.zip › suppl fig 5G type 35/Plate01/pm01f01.gif]

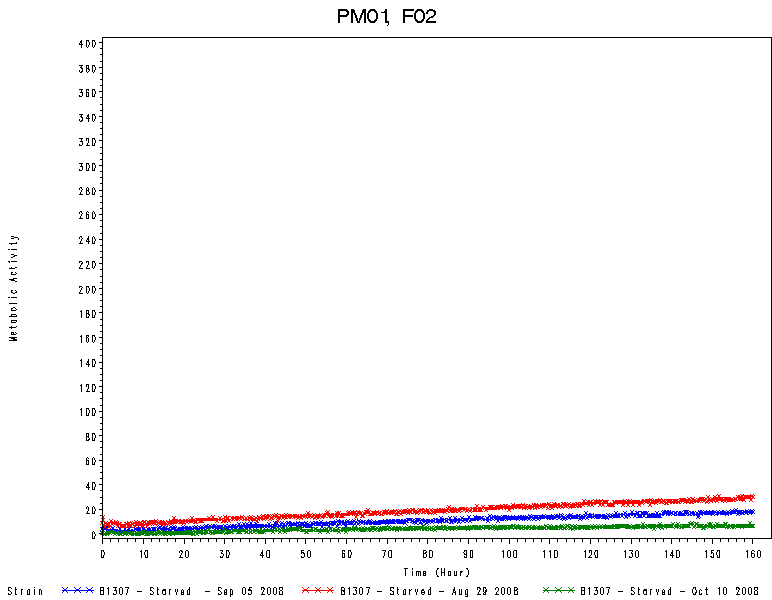

Supplement: Figure S5 — Kinetic curves for all PM plates with M. bovis Type 35 strains. Figures S1 to S5 were generated in SAS using a GPLOT procedure, as described in the methods. Each figure is a Zip file containing plots of Omnilog units (due to dye reduction) against time (0 to 168 h) for all wells of each of the six 96 well plates. Each well is identified by (plate, well) and a list of the contents of wells is in Supplementary Table S1. (ZIP) [file pone.0052673.s005.zip › suppl fig 5G type 35/Plate01/pm01f02.gif]

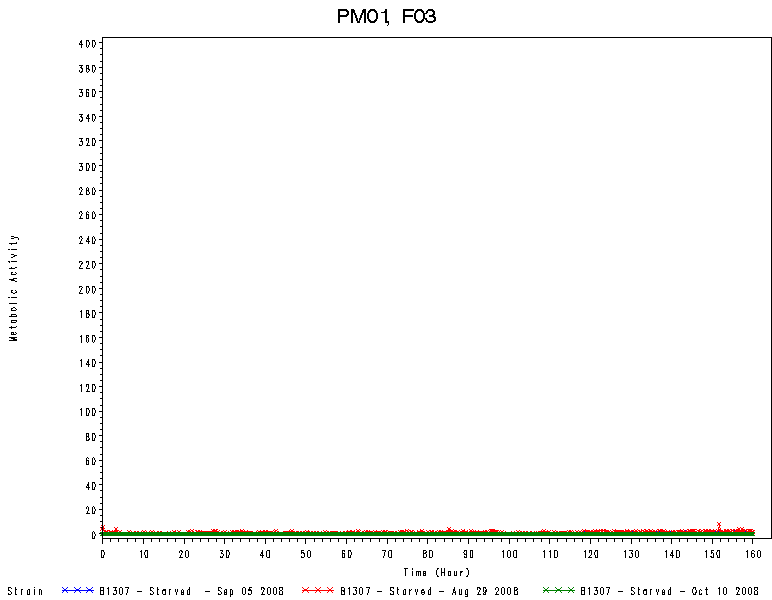

Supplement: Figure S5 — Kinetic curves for all PM plates with M. bovis Type 35 strains. Figures S1 to S5 were generated in SAS using a GPLOT procedure, as described in the methods. Each figure is a Zip file containing plots of Omnilog units (due to dye reduction) against time (0 to 168 h) for all wells of each of the six 96 well plates. Each well is identified by (plate, well) and a list of the contents of wells is in Supplementary Table S1. (ZIP) [file pone.0052673.s005.zip › suppl fig 5G type 35/Plate01/pm01f03.gif]

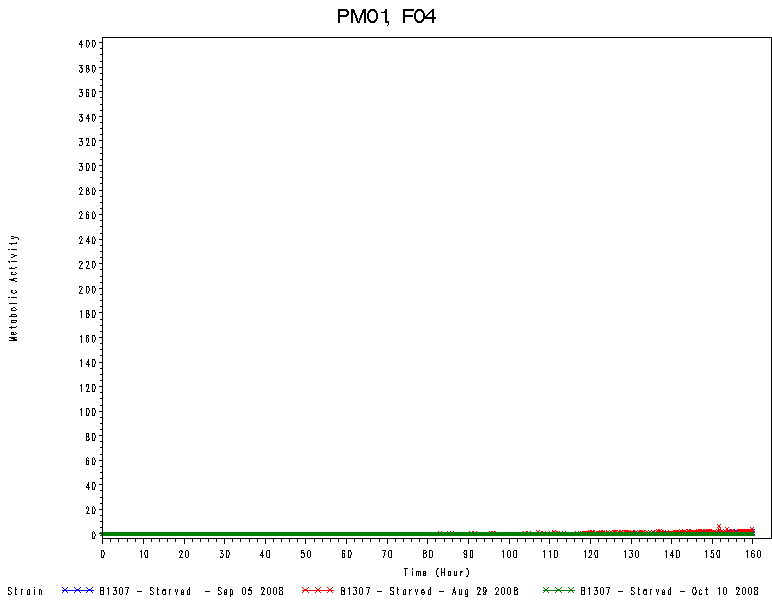

Supplement: Figure S5 — Kinetic curves for all PM plates with M. bovis Type 35 strains. Figures S1 to S5 were generated in SAS using a GPLOT procedure, as described in the methods. Each figure is a Zip file containing plots of Omnilog units (due to dye reduction) against time (0 to 168 h) for all wells of each of the six 96 well plates. Each well is identified by (plate, well) and a list of the contents of wells is in Supplementary Table S1. (ZIP) [file pone.0052673.s005.zip › suppl fig 5G type 35/Plate01/pm01f04.gif]

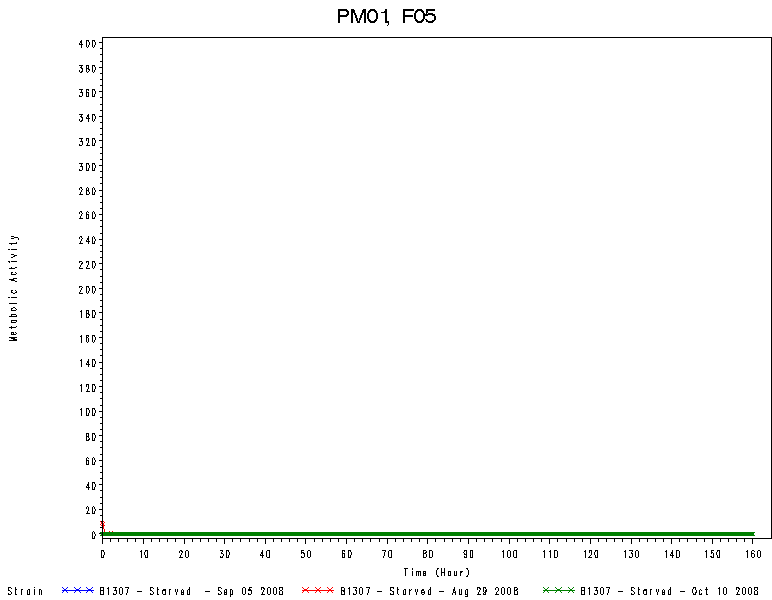

Supplement: Figure S5 — Kinetic curves for all PM plates with M. bovis Type 35 strains. Figures S1 to S5 were generated in SAS using a GPLOT procedure, as described in the methods. Each figure is a Zip file containing plots of Omnilog units (due to dye reduction) against time (0 to 168 h) for all wells of each of the six 96 well plates. Each well is identified by (plate, well) and a list of the contents of wells is in Supplementary Table S1. (ZIP) [file pone.0052673.s005.zip › suppl fig 5G type 35/Plate01/pm01f05.gif]

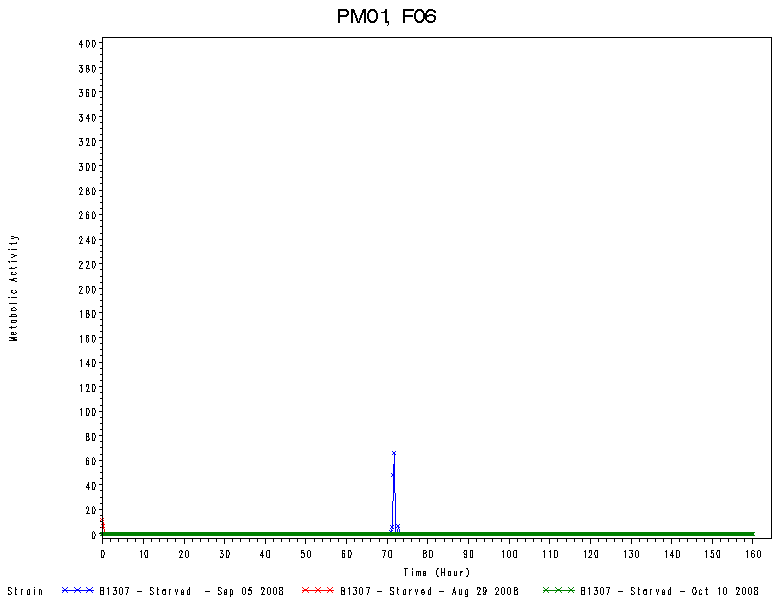

Supplement: Figure S5 — Kinetic curves for all PM plates with M. bovis Type 35 strains. Figures S1 to S5 were generated in SAS using a GPLOT procedure, as described in the methods. Each figure is a Zip file containing plots of Omnilog units (due to dye reduction) against time (0 to 168 h) for all wells of each of the six 96 well plates. Each well is identified by (plate, well) and a list of the contents of wells is in Supplementary Table S1. (ZIP) [file pone.0052673.s005.zip › suppl fig 5G type 35/Plate01/pm01f06.gif]

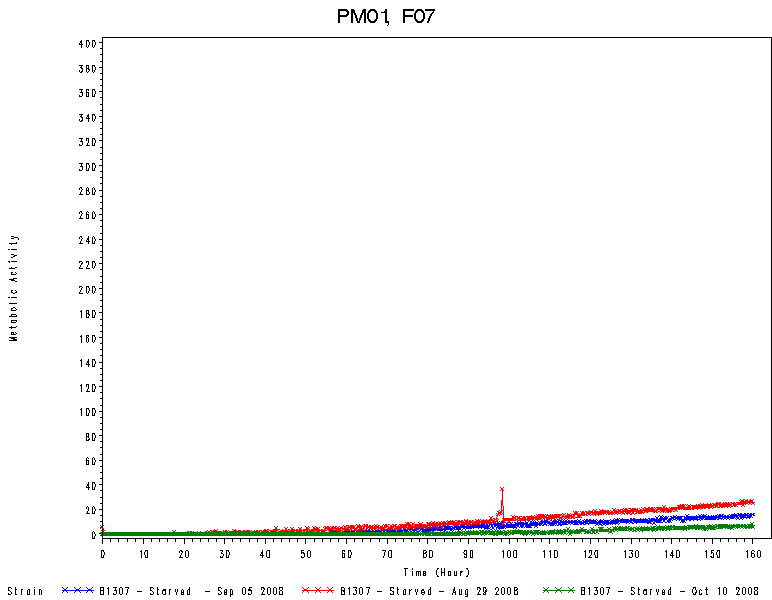

Supplement: Figure S5 — Kinetic curves for all PM plates with M. bovis Type 35 strains. Figures S1 to S5 were generated in SAS using a GPLOT procedure, as described in the methods. Each figure is a Zip file containing plots of Omnilog units (due to dye reduction) against time (0 to 168 h) for all wells of each of the six 96 well plates. Each well is identified by (plate, well) and a list of the contents of wells is in Supplementary Table S1. (ZIP) [file pone.0052673.s005.zip › suppl fig 5G type 35/Plate01/pm01f07.gif]

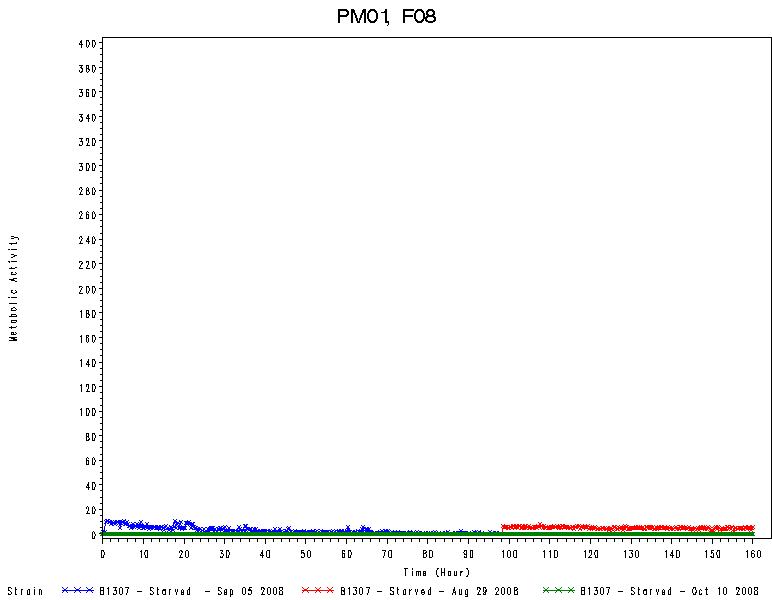

Supplement: Figure S5 — Kinetic curves for all PM plates with M. bovis Type 35 strains. Figures S1 to S5 were generated in SAS using a GPLOT procedure, as described in the methods. Each figure is a Zip file containing plots of Omnilog units (due to dye reduction) against time (0 to 168 h) for all wells of each of the six 96 well plates. Each well is identified by (plate, well) and a list of the contents of wells is in Supplementary Table S1. (ZIP) [file pone.0052673.s005.zip › suppl fig 5G type 35/Plate01/pm01f08.gif]

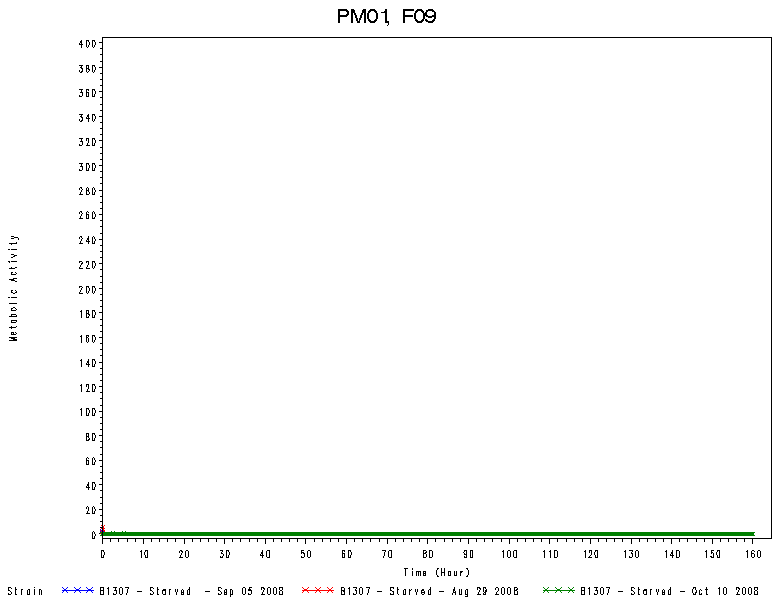

Supplement: Figure S5 — Kinetic curves for all PM plates with M. bovis Type 35 strains. Figures S1 to S5 were generated in SAS using a GPLOT procedure, as described in the methods. Each figure is a Zip file containing plots of Omnilog units (due to dye reduction) against time (0 to 168 h) for all wells of each of the six 96 well plates. Each well is identified by (plate, well) and a list of the contents of wells is in Supplementary Table S1. (ZIP) [file pone.0052673.s005.zip › suppl fig 5G type 35/Plate01/pm01f09.gif]

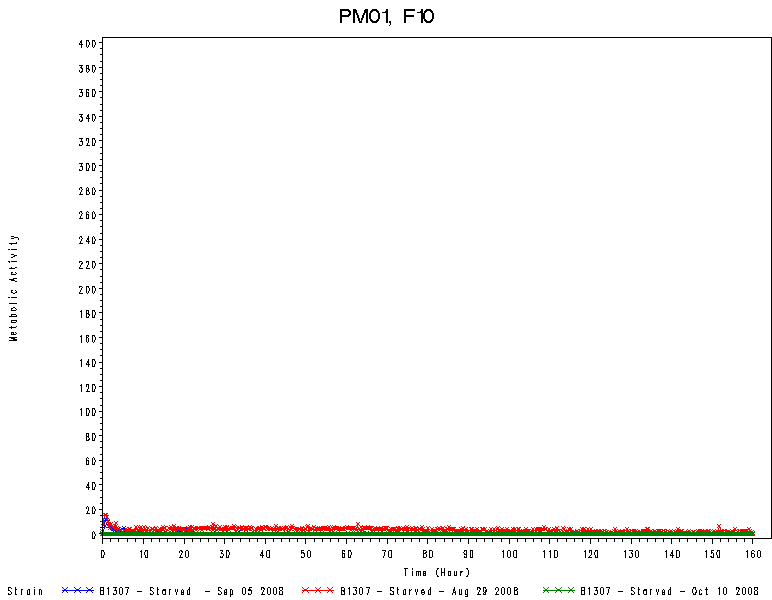

Supplement: Figure S5 — Kinetic curves for all PM plates with M. bovis Type 35 strains. Figures S1 to S5 were generated in SAS using a GPLOT procedure, as described in the methods. Each figure is a Zip file containing plots of Omnilog units (due to dye reduction) against time (0 to 168 h) for all wells of each of the six 96 well plates. Each well is identified by (plate, well) and a list of the contents of wells is in Supplementary Table S1. (ZIP) [file pone.0052673.s005.zip › suppl fig 5G type 35/Plate01/pm01f10.gif]

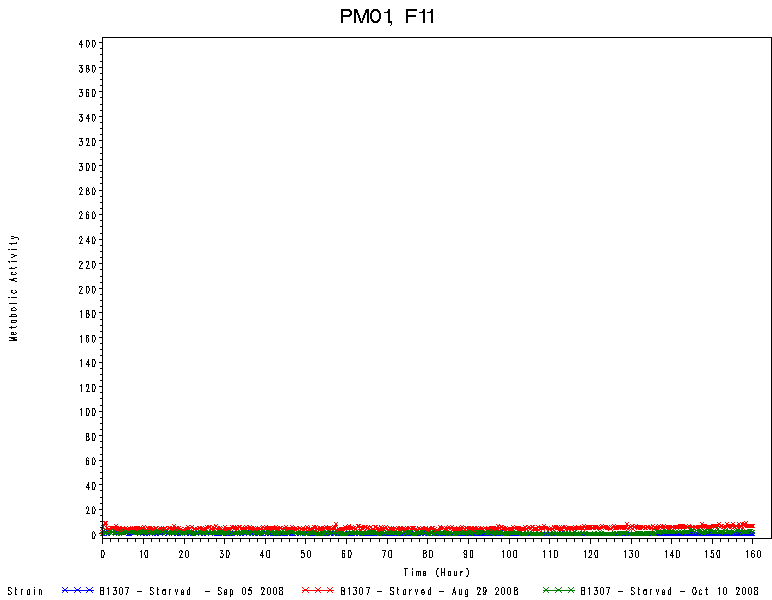

Supplement: Figure S5 — Kinetic curves for all PM plates with M. bovis Type 35 strains. Figures S1 to S5 were generated in SAS using a GPLOT procedure, as described in the methods. Each figure is a Zip file containing plots of Omnilog units (due to dye reduction) against time (0 to 168 h) for all wells of each of the six 96 well plates. Each well is identified by (plate, well) and a list of the contents of wells is in Supplementary Table S1. (ZIP) [file pone.0052673.s005.zip › suppl fig 5G type 35/Plate01/pm01f11.gif]

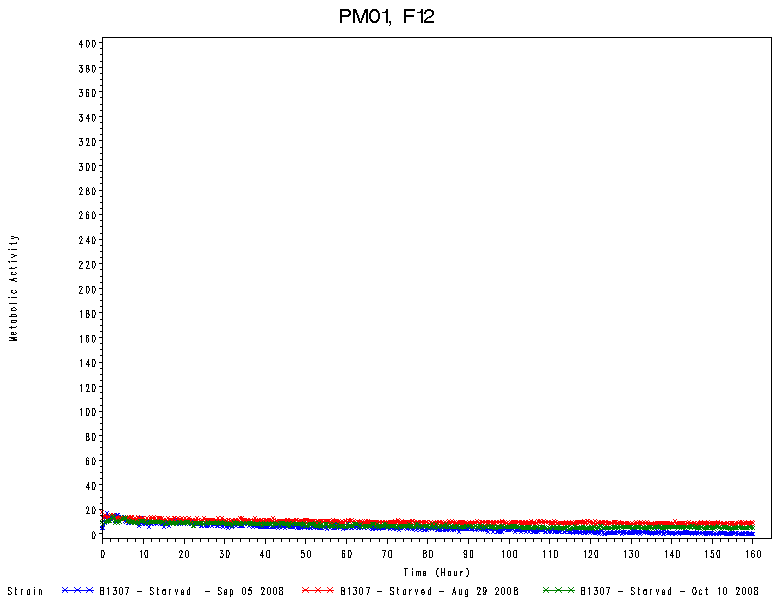

Supplement: Figure S5 — Kinetic curves for all PM plates with M. bovis Type 35 strains. Figures S1 to S5 were generated in SAS using a GPLOT procedure, as described in the methods. Each figure is a Zip file containing plots of Omnilog units (due to dye reduction) against time (0 to 168 h) for all wells of each of the six 96 well plates. Each well is identified by (plate, well) and a list of the contents of wells is in Supplementary Table S1. (ZIP) [file pone.0052673.s005.zip › suppl fig 5G type 35/Plate01/pm01f12.gif]

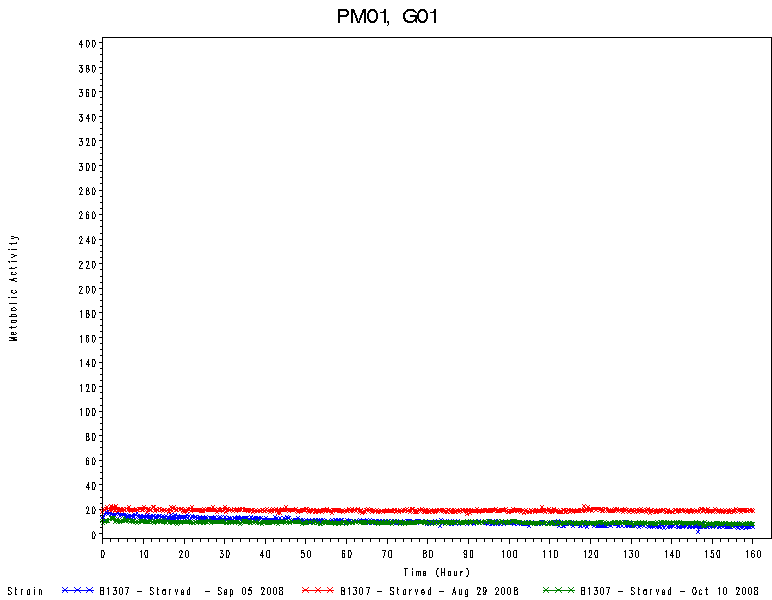

Supplement: Figure S5 — Kinetic curves for all PM plates with M. bovis Type 35 strains. Figures S1 to S5 were generated in SAS using a GPLOT procedure, as described in the methods. Each figure is a Zip file containing plots of Omnilog units (due to dye reduction) against time (0 to 168 h) for all wells of each of the six 96 well plates. Each well is identified by (plate, well) and a list of the contents of wells is in Supplementary Table S1. (ZIP) [file pone.0052673.s005.zip › suppl fig 5G type 35/Plate01/pm01g01.gif]

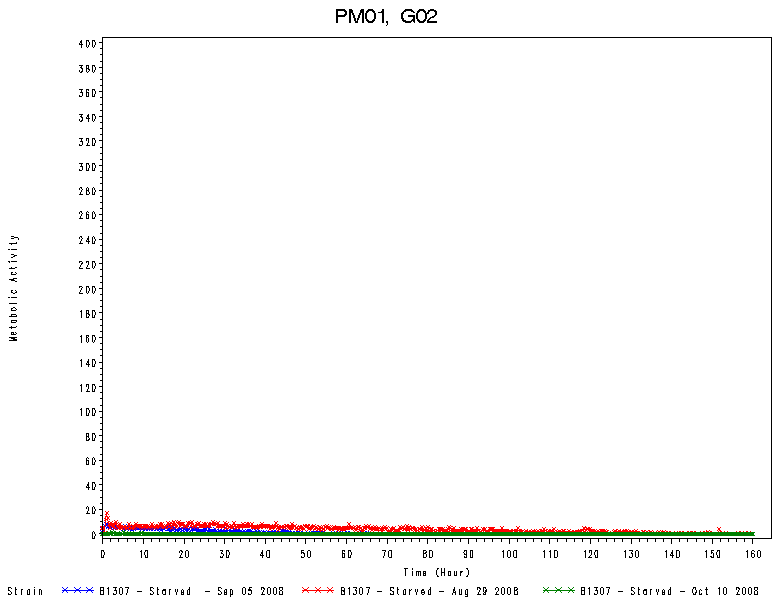

Supplement: Figure S5 — Kinetic curves for all PM plates with M. bovis Type 35 strains. Figures S1 to S5 were generated in SAS using a GPLOT procedure, as described in the methods. Each figure is a Zip file containing plots of Omnilog units (due to dye reduction) against time (0 to 168 h) for all wells of each of the six 96 well plates. Each well is identified by (plate, well) and a list of the contents of wells is in Supplementary Table S1. (ZIP) [file pone.0052673.s005.zip › suppl fig 5G type 35/Plate01/pm01g02.gif]

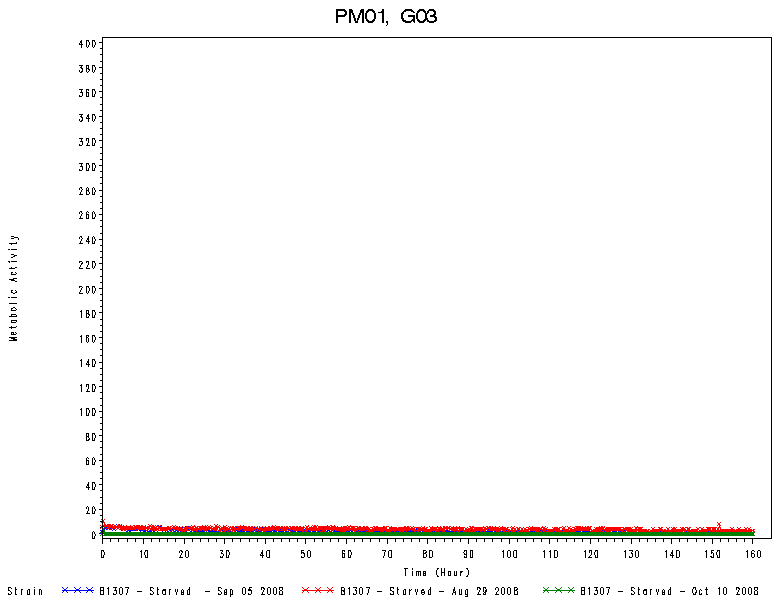

Supplement: Figure S5 — Kinetic curves for all PM plates with M. bovis Type 35 strains. Figures S1 to S5 were generated in SAS using a GPLOT procedure, as described in the methods. Each figure is a Zip file containing plots of Omnilog units (due to dye reduction) against time (0 to 168 h) for all wells of each of the six 96 well plates. Each well is identified by (plate, well) and a list of the contents of wells is in Supplementary Table S1. (ZIP) [file pone.0052673.s005.zip › suppl fig 5G type 35/Plate01/pm01g03.gif]

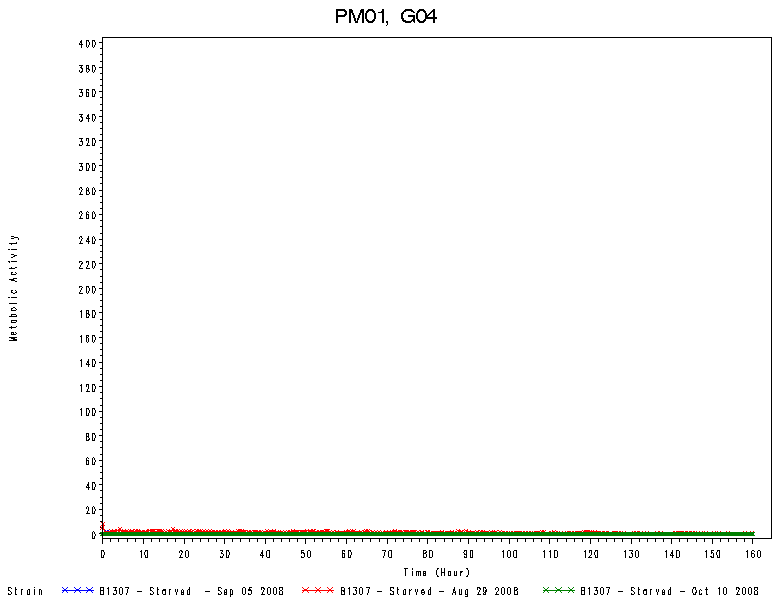

Supplement: Figure S5 — Kinetic curves for all PM plates with M. bovis Type 35 strains. Figures S1 to S5 were generated in SAS using a GPLOT procedure, as described in the methods. Each figure is a Zip file containing plots of Omnilog units (due to dye reduction) against time (0 to 168 h) for all wells of each of the six 96 well plates. Each well is identified by (plate, well) and a list of the contents of wells is in Supplementary Table S1. (ZIP) [file pone.0052673.s005.zip › suppl fig 5G type 35/Plate01/pm01g04.gif]

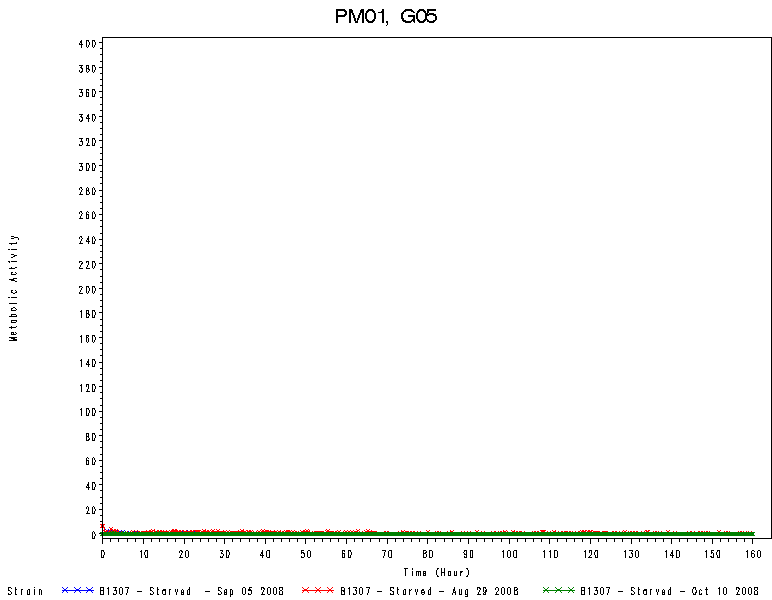

Supplement: Figure S5 — Kinetic curves for all PM plates with M. bovis Type 35 strains. Figures S1 to S5 were generated in SAS using a GPLOT procedure, as described in the methods. Each figure is a Zip file containing plots of Omnilog units (due to dye reduction) against time (0 to 168 h) for all wells of each of the six 96 well plates. Each well is identified by (plate, well) and a list of the contents of wells is in Supplementary Table S1. (ZIP) [file pone.0052673.s005.zip › suppl fig 5G type 35/Plate01/pm01g05.gif]

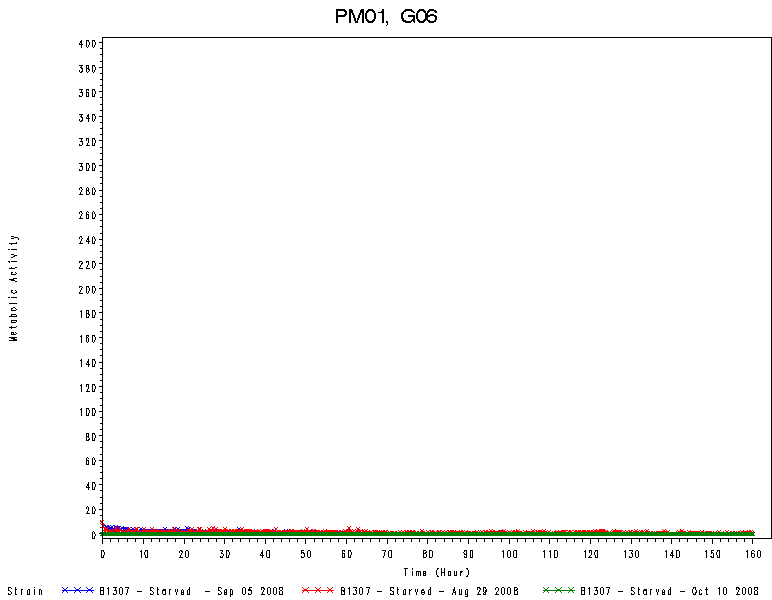

Supplement: Figure S5 — Kinetic curves for all PM plates with M. bovis Type 35 strains. Figures S1 to S5 were generated in SAS using a GPLOT procedure, as described in the methods. Each figure is a Zip file containing plots of Omnilog units (due to dye reduction) against time (0 to 168 h) for all wells of each of the six 96 well plates. Each well is identified by (plate, well) and a list of the contents of wells is in Supplementary Table S1. (ZIP) [file pone.0052673.s005.zip › suppl fig 5G type 35/Plate01/pm01g06.gif]

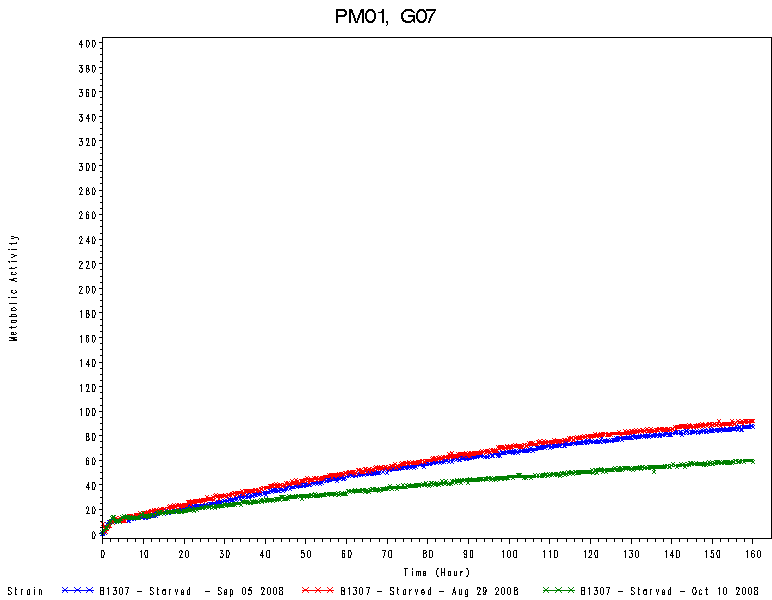

Supplement: Figure S5 — Kinetic curves for all PM plates with M. bovis Type 35 strains. Figures S1 to S5 were generated in SAS using a GPLOT procedure, as described in the methods. Each figure is a Zip file containing plots of Omnilog units (due to dye reduction) against time (0 to 168 h) for all wells of each of the six 96 well plates. Each well is identified by (plate, well) and a list of the contents of wells is in Supplementary Table S1. (ZIP) [file pone.0052673.s005.zip › suppl fig 5G type 35/Plate01/pm01g07.gif]

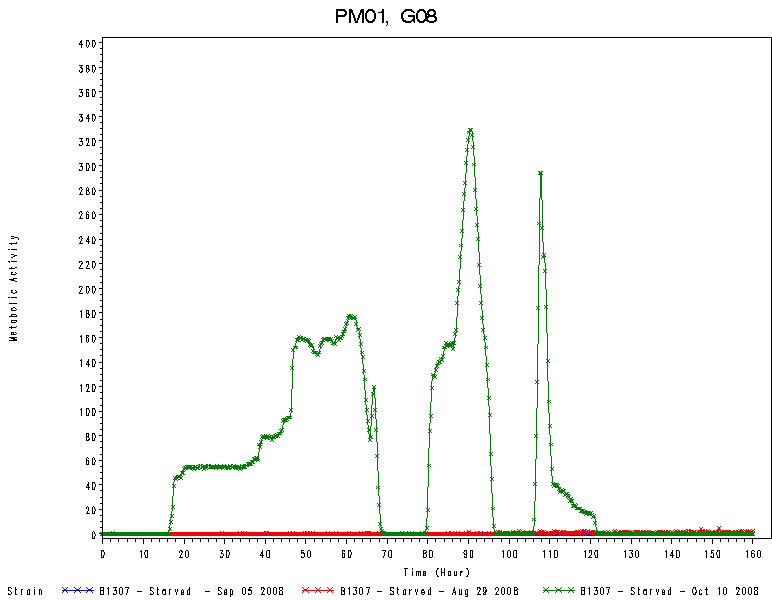

Supplement: Figure S5 — Kinetic curves for all PM plates with M. bovis Type 35 strains. Figures S1 to S5 were generated in SAS using a GPLOT procedure, as described in the methods. Each figure is a Zip file containing plots of Omnilog units (due to dye reduction) against time (0 to 168 h) for all wells of each of the six 96 well plates. Each well is identified by (plate, well) and a list of the contents of wells is in Supplementary Table S1. (ZIP) [file pone.0052673.s005.zip › suppl fig 5G type 35/Plate01/pm01g08.gif]

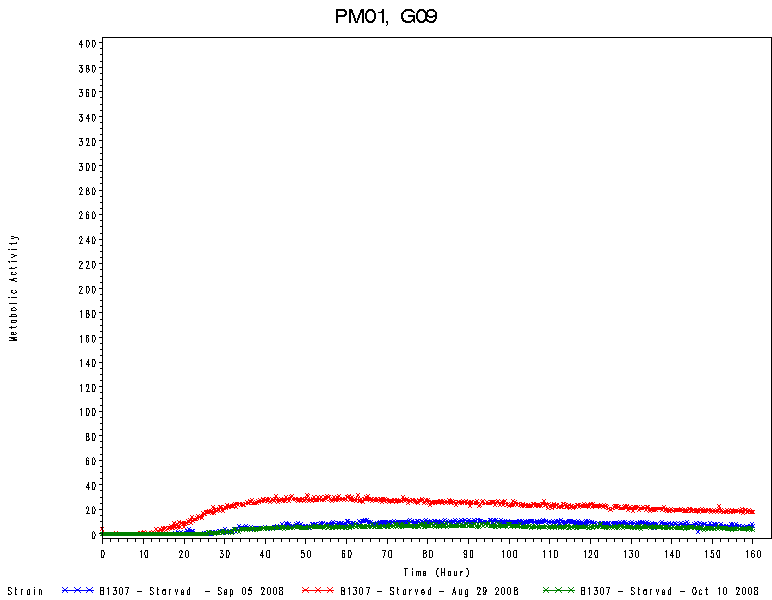

Supplement: Figure S5 — Kinetic curves for all PM plates with M. bovis Type 35 strains. Figures S1 to S5 were generated in SAS using a GPLOT procedure, as described in the methods. Each figure is a Zip file containing plots of Omnilog units (due to dye reduction) against time (0 to 168 h) for all wells of each of the six 96 well plates. Each well is identified by (plate, well) and a list of the contents of wells is in Supplementary Table S1. (ZIP) [file pone.0052673.s005.zip › suppl fig 5G type 35/Plate01/pm01g09.gif]

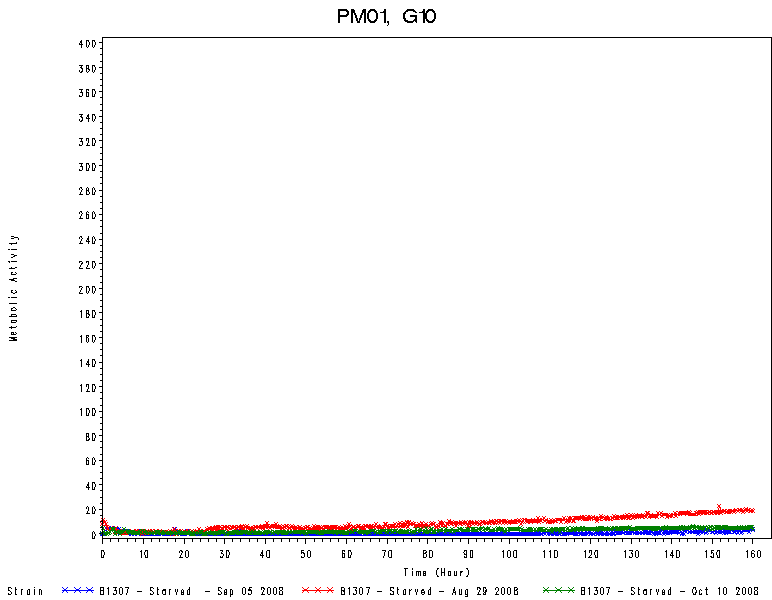

Supplement: Figure S5 — Kinetic curves for all PM plates with M. bovis Type 35 strains. Figures S1 to S5 were generated in SAS using a GPLOT procedure, as described in the methods. Each figure is a Zip file containing plots of Omnilog units (due to dye reduction) against time (0 to 168 h) for all wells of each of the six 96 well plates. Each well is identified by (plate, well) and a list of the contents of wells is in Supplementary Table S1. (ZIP) [file pone.0052673.s005.zip › suppl fig 5G type 35/Plate01/pm01g10.gif]

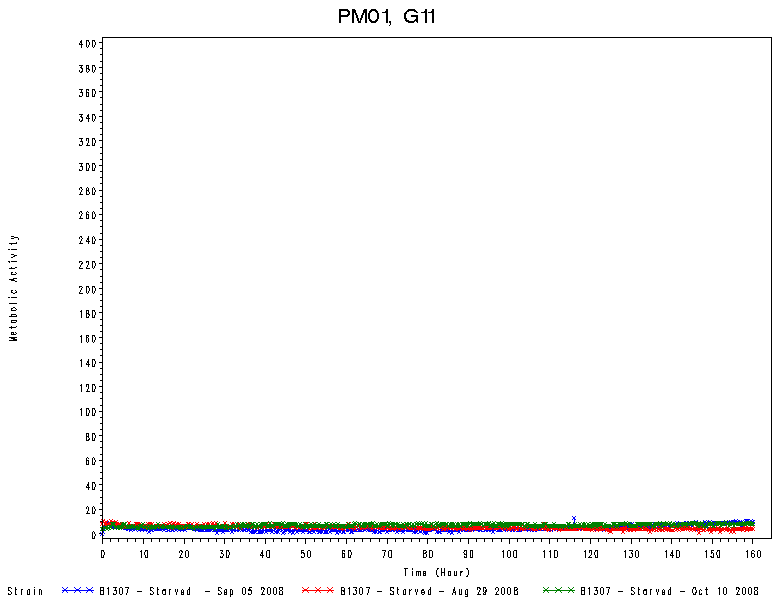

Supplement: Figure S5 — Kinetic curves for all PM plates with M. bovis Type 35 strains. Figures S1 to S5 were generated in SAS using a GPLOT procedure, as described in the methods. Each figure is a Zip file containing plots of Omnilog units (due to dye reduction) against time (0 to 168 h) for all wells of each of the six 96 well plates. Each well is identified by (plate, well) and a list of the contents of wells is in Supplementary Table S1. (ZIP) [file pone.0052673.s005.zip › suppl fig 5G type 35/Plate01/pm01g11.gif]

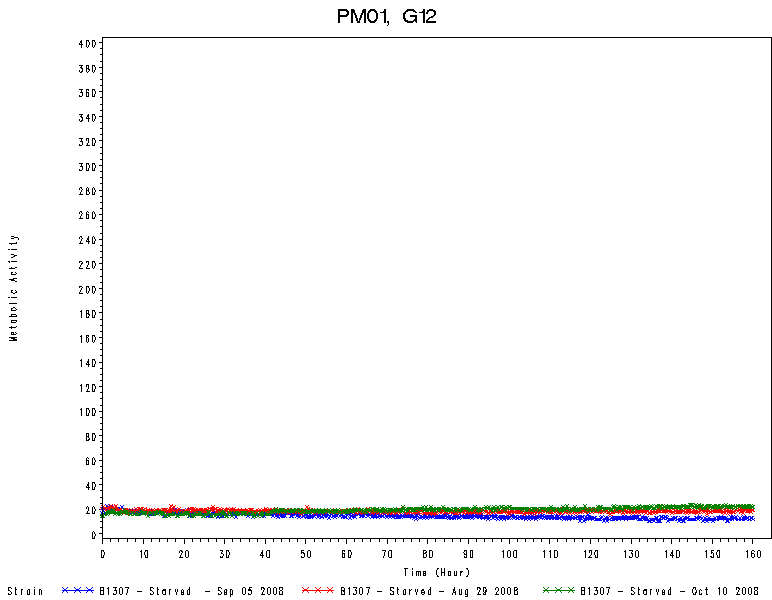

Supplement: Figure S5 — Kinetic curves for all PM plates with M. bovis Type 35 strains. Figures S1 to S5 were generated in SAS using a GPLOT procedure, as described in the methods. Each figure is a Zip file containing plots of Omnilog units (due to dye reduction) against time (0 to 168 h) for all wells of each of the six 96 well plates. Each well is identified by (plate, well) and a list of the contents of wells is in Supplementary Table S1. (ZIP) [file pone.0052673.s005.zip › suppl fig 5G type 35/Plate01/pm01g12.gif]

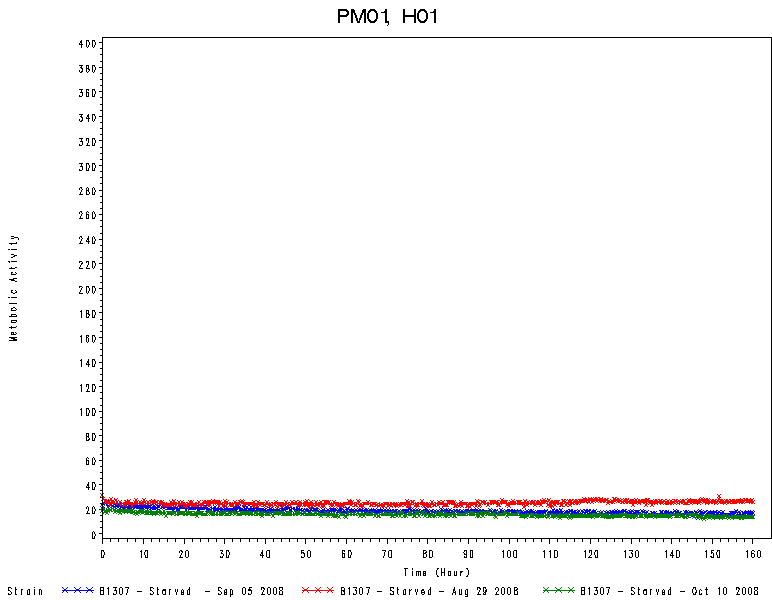

Supplement: Figure S5 — Kinetic curves for all PM plates with M. bovis Type 35 strains. Figures S1 to S5 were generated in SAS using a GPLOT procedure, as described in the methods. Each figure is a Zip file containing plots of Omnilog units (due to dye reduction) against time (0 to 168 h) for all wells of each of the six 96 well plates. Each well is identified by (plate, well) and a list of the contents of wells is in Supplementary Table S1. (ZIP) [file pone.0052673.s005.zip › suppl fig 5G type 35/Plate01/pm01h01.gif]

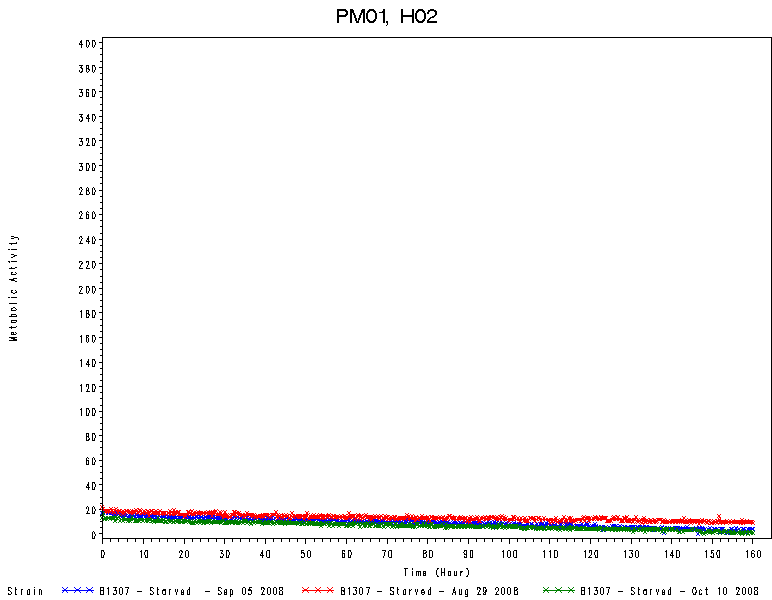

Supplement: Figure S5 — Kinetic curves for all PM plates with M. bovis Type 35 strains. Figures S1 to S5 were generated in SAS using a GPLOT procedure, as described in the methods. Each figure is a Zip file containing plots of Omnilog units (due to dye reduction) against time (0 to 168 h) for all wells of each of the six 96 well plates. Each well is identified by (plate, well) and a list of the contents of wells is in Supplementary Table S1. (ZIP) [file pone.0052673.s005.zip › suppl fig 5G type 35/Plate01/pm01h02.gif]

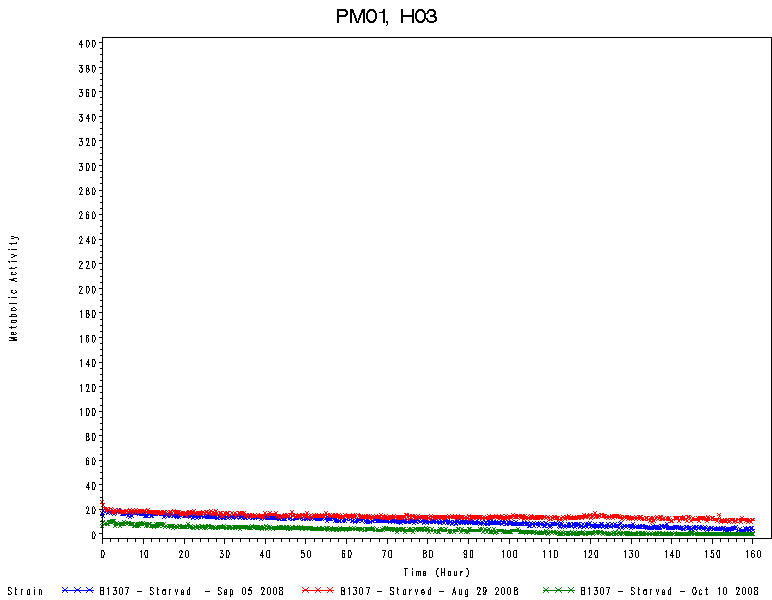

Supplement: Figure S5 — Kinetic curves for all PM plates with M. bovis Type 35 strains. Figures S1 to S5 were generated in SAS using a GPLOT procedure, as described in the methods. Each figure is a Zip file containing plots of Omnilog units (due to dye reduction) against time (0 to 168 h) for all wells of each of the six 96 well plates. Each well is identified by (plate, well) and a list of the contents of wells is in Supplementary Table S1. (ZIP) [file pone.0052673.s005.zip › suppl fig 5G type 35/Plate01/pm01h03.gif]

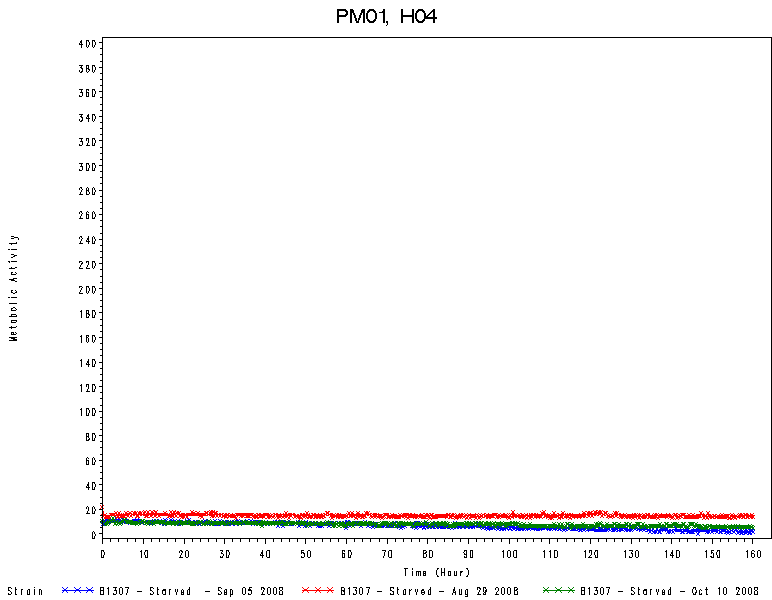

Supplement: Figure S5 — Kinetic curves for all PM plates with M. bovis Type 35 strains. Figures S1 to S5 were generated in SAS using a GPLOT procedure, as described in the methods. Each figure is a Zip file containing plots of Omnilog units (due to dye reduction) against time (0 to 168 h) for all wells of each of the six 96 well plates. Each well is identified by (plate, well) and a list of the contents of wells is in Supplementary Table S1. (ZIP) [file pone.0052673.s005.zip › suppl fig 5G type 35/Plate01/pm01h04.gif]

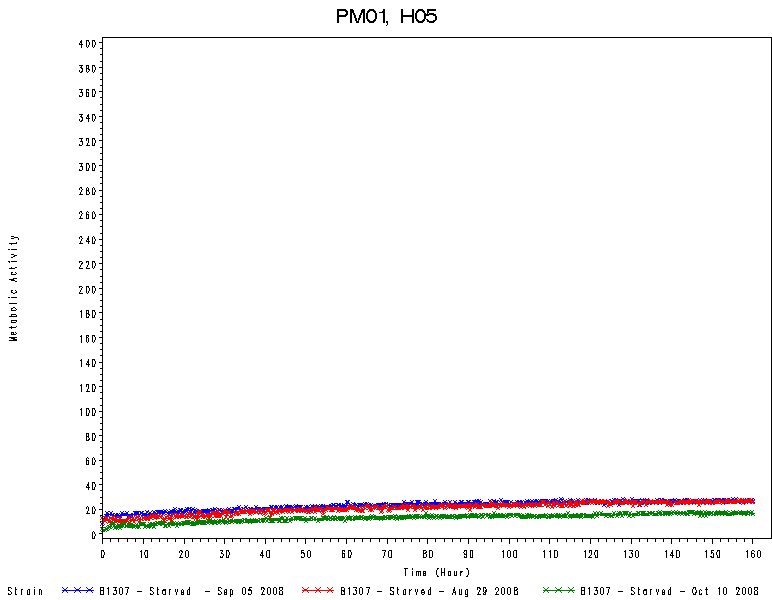

Supplement: Figure S5 — Kinetic curves for all PM plates with M. bovis Type 35 strains. Figures S1 to S5 were generated in SAS using a GPLOT procedure, as described in the methods. Each figure is a Zip file containing plots of Omnilog units (due to dye reduction) against time (0 to 168 h) for all wells of each of the six 96 well plates. Each well is identified by (plate, well) and a list of the contents of wells is in Supplementary Table S1. (ZIP) [file pone.0052673.s005.zip › suppl fig 5G type 35/Plate01/pm01h05.gif]

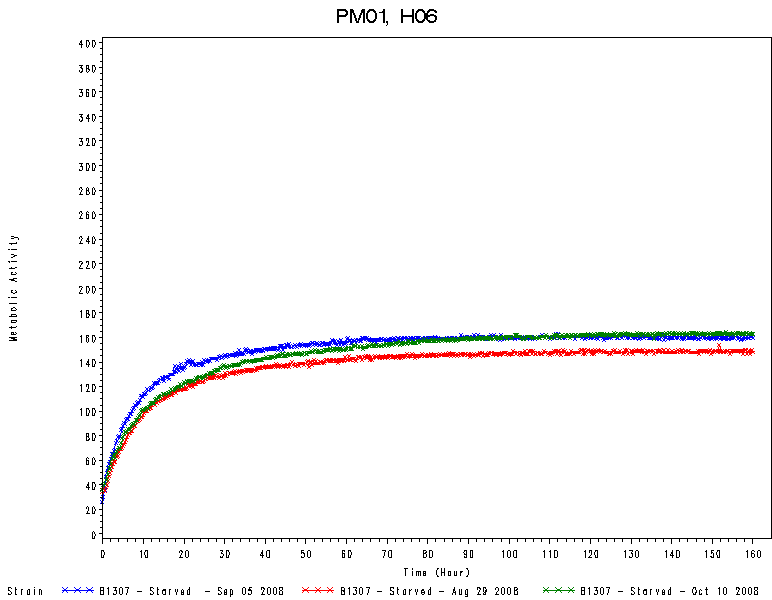

Supplement: Figure S5 — Kinetic curves for all PM plates with M. bovis Type 35 strains. Figures S1 to S5 were generated in SAS using a GPLOT procedure, as described in the methods. Each figure is a Zip file containing plots of Omnilog units (due to dye reduction) against time (0 to 168 h) for all wells of each of the six 96 well plates. Each well is identified by (plate, well) and a list of the contents of wells is in Supplementary Table S1. (ZIP) [file pone.0052673.s005.zip › suppl fig 5G type 35/Plate01/pm01h06.gif]

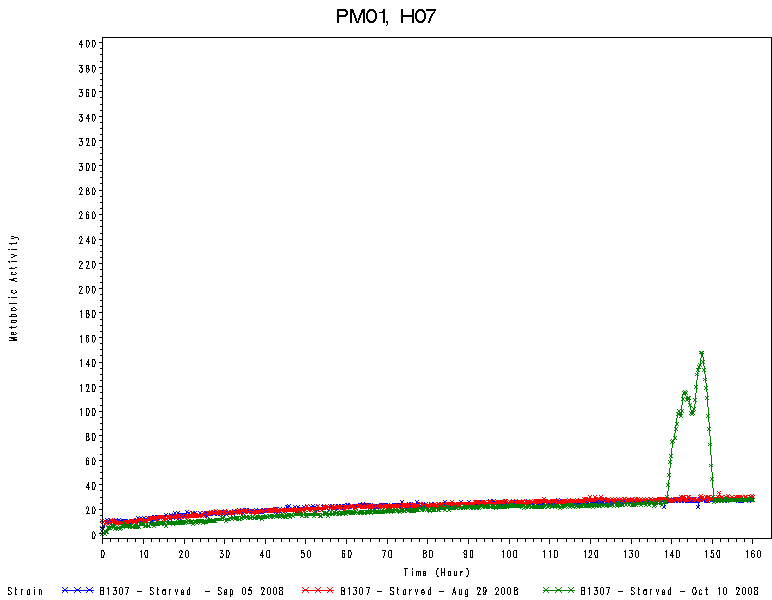

Supplement: Figure S5 — Kinetic curves for all PM plates with M. bovis Type 35 strains. Figures S1 to S5 were generated in SAS using a GPLOT procedure, as described in the methods. Each figure is a Zip file containing plots of Omnilog units (due to dye reduction) against time (0 to 168 h) for all wells of each of the six 96 well plates. Each well is identified by (plate, well) and a list of the contents of wells is in Supplementary Table S1. (ZIP) [file pone.0052673.s005.zip › suppl fig 5G type 35/Plate01/pm01h07.gif]

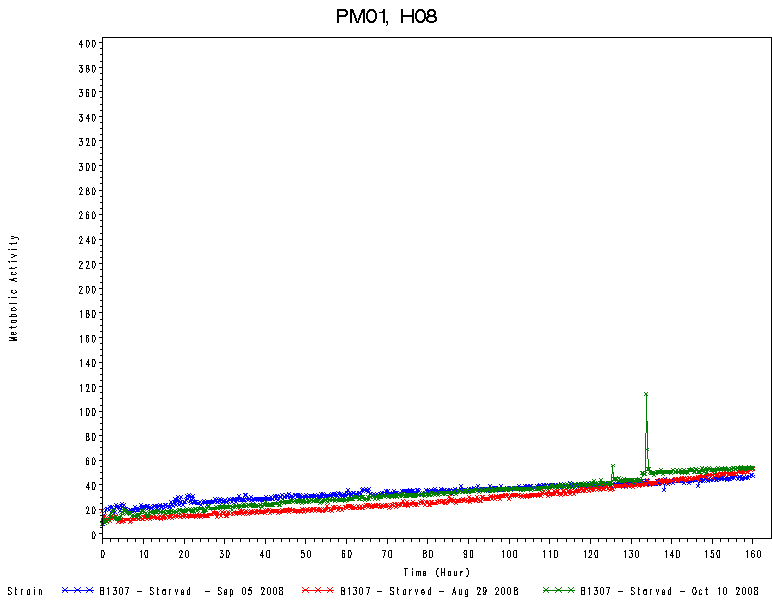

Supplement: Figure S5 — Kinetic curves for all PM plates with M. bovis Type 35 strains. Figures S1 to S5 were generated in SAS using a GPLOT procedure, as described in the methods. Each figure is a Zip file containing plots of Omnilog units (due to dye reduction) against time (0 to 168 h) for all wells of each of the six 96 well plates. Each well is identified by (plate, well) and a list of the contents of wells is in Supplementary Table S1. (ZIP) [file pone.0052673.s005.zip › suppl fig 5G type 35/Plate01/pm01h08.gif]

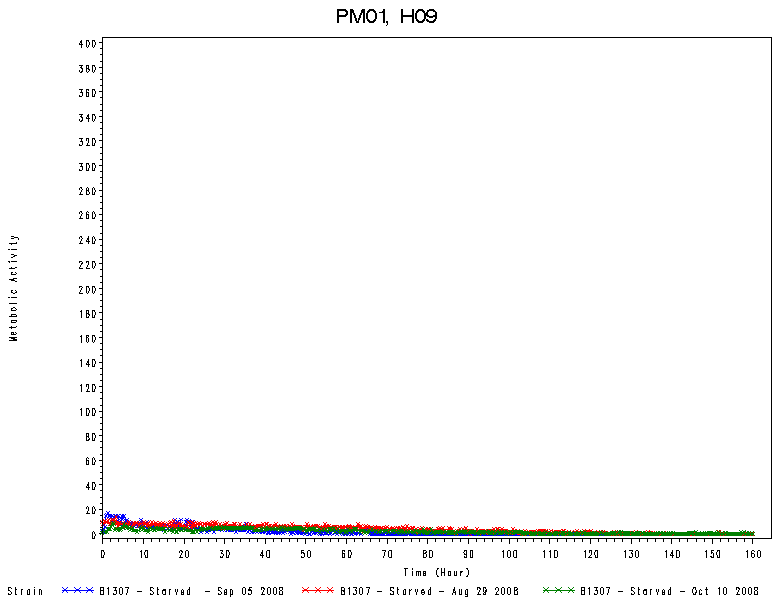

Supplement: Figure S5 — Kinetic curves for all PM plates with M. bovis Type 35 strains. Figures S1 to S5 were generated in SAS using a GPLOT procedure, as described in the methods. Each figure is a Zip file containing plots of Omnilog units (due to dye reduction) against time (0 to 168 h) for all wells of each of the six 96 well plates. Each well is identified by (plate, well) and a list of the contents of wells is in Supplementary Table S1. (ZIP) [file pone.0052673.s005.zip › suppl fig 5G type 35/Plate01/pm01h09.gif]

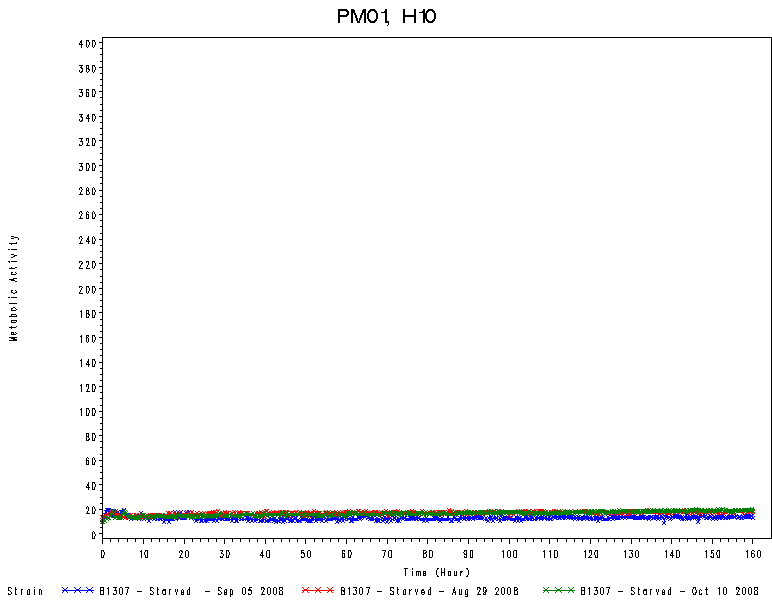

Supplement: Figure S5 — Kinetic curves for all PM plates with M. bovis Type 35 strains. Figures S1 to S5 were generated in SAS using a GPLOT procedure, as described in the methods. Each figure is a Zip file containing plots of Omnilog units (due to dye reduction) against time (0 to 168 h) for all wells of each of the six 96 well plates. Each well is identified by (plate, well) and a list of the contents of wells is in Supplementary Table S1. (ZIP) [file pone.0052673.s005.zip › suppl fig 5G type 35/Plate01/pm01h10.gif]

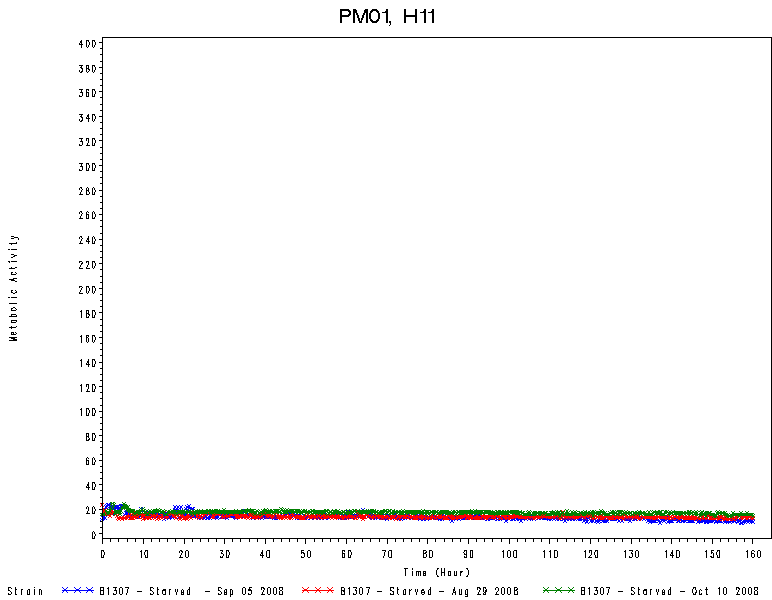

Supplement: Figure S5 — Kinetic curves for all PM plates with M. bovis Type 35 strains. Figures S1 to S5 were generated in SAS using a GPLOT procedure, as described in the methods. Each figure is a Zip file containing plots of Omnilog units (due to dye reduction) against time (0 to 168 h) for all wells of each of the six 96 well plates. Each well is identified by (plate, well) and a list of the contents of wells is in Supplementary Table S1. (ZIP) [file pone.0052673.s005.zip › suppl fig 5G type 35/Plate01/pm01h11.gif]

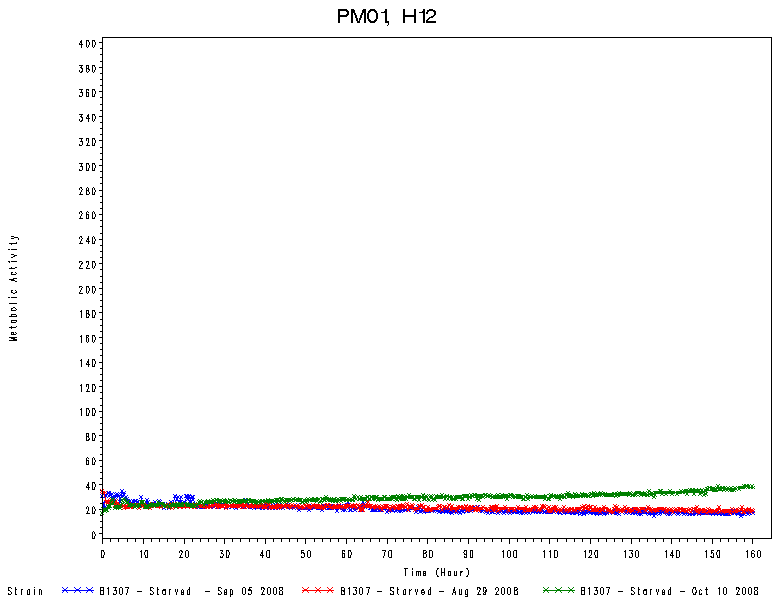

Supplement: Figure S5 — Kinetic curves for all PM plates with M. bovis Type 35 strains. Figures S1 to S5 were generated in SAS using a GPLOT procedure, as described in the methods. Each figure is a Zip file containing plots of Omnilog units (due to dye reduction) against time (0 to 168 h) for all wells of each of the six 96 well plates. Each well is identified by (plate, well) and a list of the contents of wells is in Supplementary Table S1. (ZIP) [file pone.0052673.s005.zip › suppl fig 5G type 35/Plate01/pm01h12.gif]

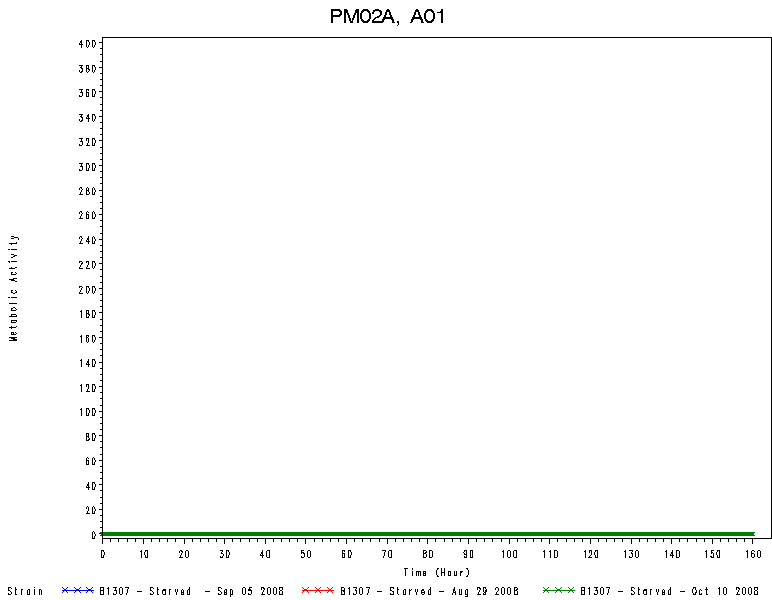

Supplement: Figure S5 — Kinetic curves for all PM plates with M. bovis Type 35 strains. Figures S1 to S5 were generated in SAS using a GPLOT procedure, as described in the methods. Each figure is a Zip file containing plots of Omnilog units (due to dye reduction) against time (0 to 168 h) for all wells of each of the six 96 well plates. Each well is identified by (plate, well) and a list of the contents of wells is in Supplementary Table S1. (ZIP) [file pone.0052673.s005.zip › suppl fig 5G type 35/Plate02A/pm02aa01.gif]

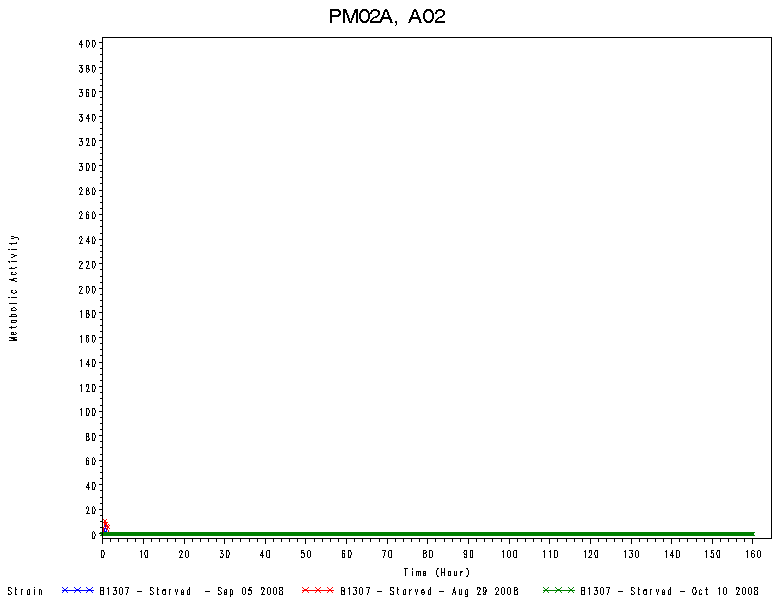

Supplement: Figure S5 — Kinetic curves for all PM plates with M. bovis Type 35 strains. Figures S1 to S5 were generated in SAS using a GPLOT procedure, as described in the methods. Each figure is a Zip file containing plots of Omnilog units (due to dye reduction) against time (0 to 168 h) for all wells of each of the six 96 well plates. Each well is identified by (plate, well) and a list of the contents of wells is in Supplementary Table S1. (ZIP) [file pone.0052673.s005.zip › suppl fig 5G type 35/Plate02A/pm02aa02.gif]

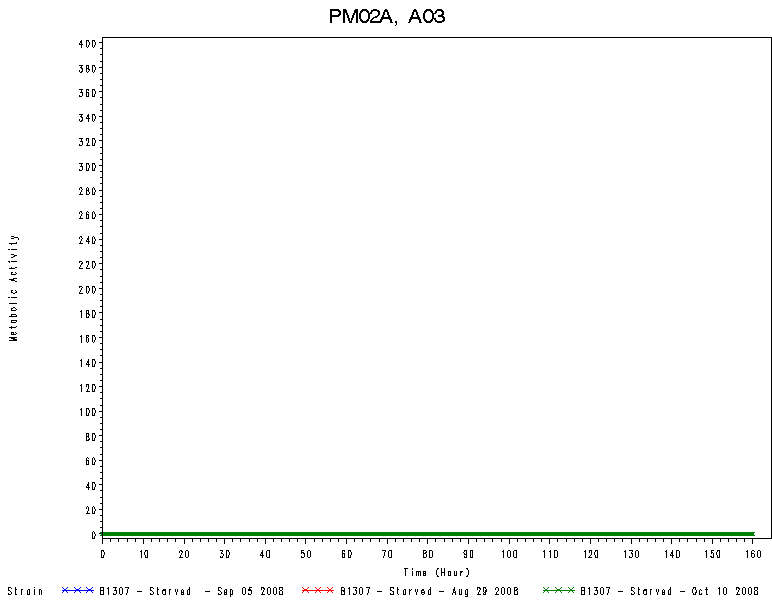

Supplement: Figure S5 — Kinetic curves for all PM plates with M. bovis Type 35 strains. Figures S1 to S5 were generated in SAS using a GPLOT procedure, as described in the methods. Each figure is a Zip file containing plots of Omnilog units (due to dye reduction) against time (0 to 168 h) for all wells of each of the six 96 well plates. Each well is identified by (plate, well) and a list of the contents of wells is in Supplementary Table S1. (ZIP) [file pone.0052673.s005.zip › suppl fig 5G type 35/Plate02A/pm02aa03.gif]

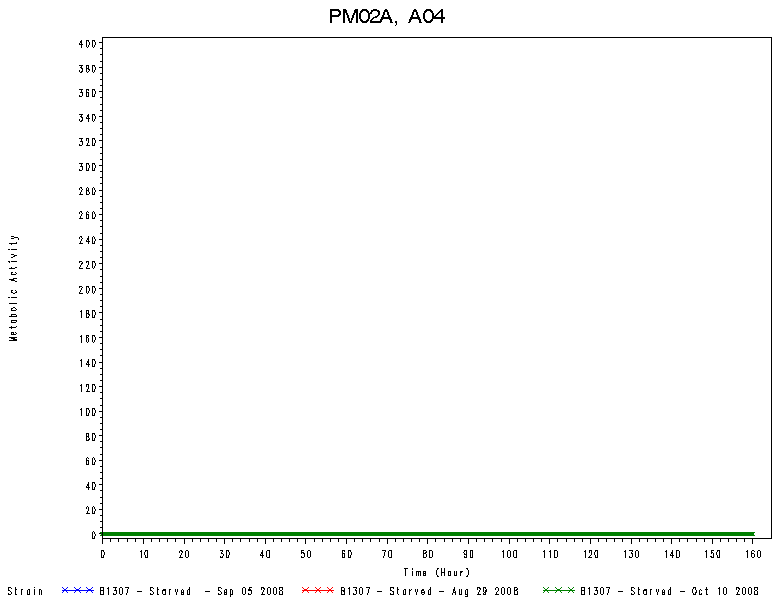

Supplement: Figure S5 — Kinetic curves for all PM plates with M. bovis Type 35 strains. Figures S1 to S5 were generated in SAS using a GPLOT procedure, as described in the methods. Each figure is a Zip file containing plots of Omnilog units (due to dye reduction) against time (0 to 168 h) for all wells of each of the six 96 well plates. Each well is identified by (plate, well) and a list of the contents of wells is in Supplementary Table S1. (ZIP) [file pone.0052673.s005.zip › suppl fig 5G type 35/Plate02A/pm02aa04.gif]
