# Supplementary figures and images for: High Throughput Phenotypic Analysis of Mycobacterium tuberculosis and Mycobacterium bovis Strains' Metabolism Using Biolog Phenotype Microarrays (part 11 of 11)
Source: PLoS One. 2013 Jan 10;8(1):e52673. doi: 10.1371/journal.pone.0052673 (PMC3542357; doi:10.1371/journal.pone.0052673)

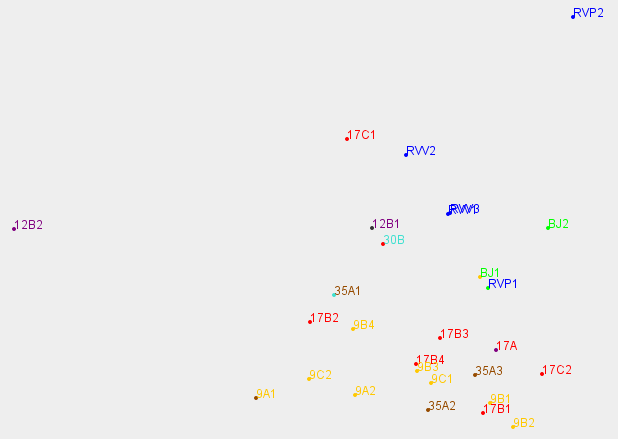

Supplement: Figure S6 — Clustering of strains from PM1 to PM4 data, in 2 dimensions. An aspect of the 3D clustering is presented in main paper and the key is in Table 1 (DOC) [file pone.0052673.s006.doc]

## Slide 1
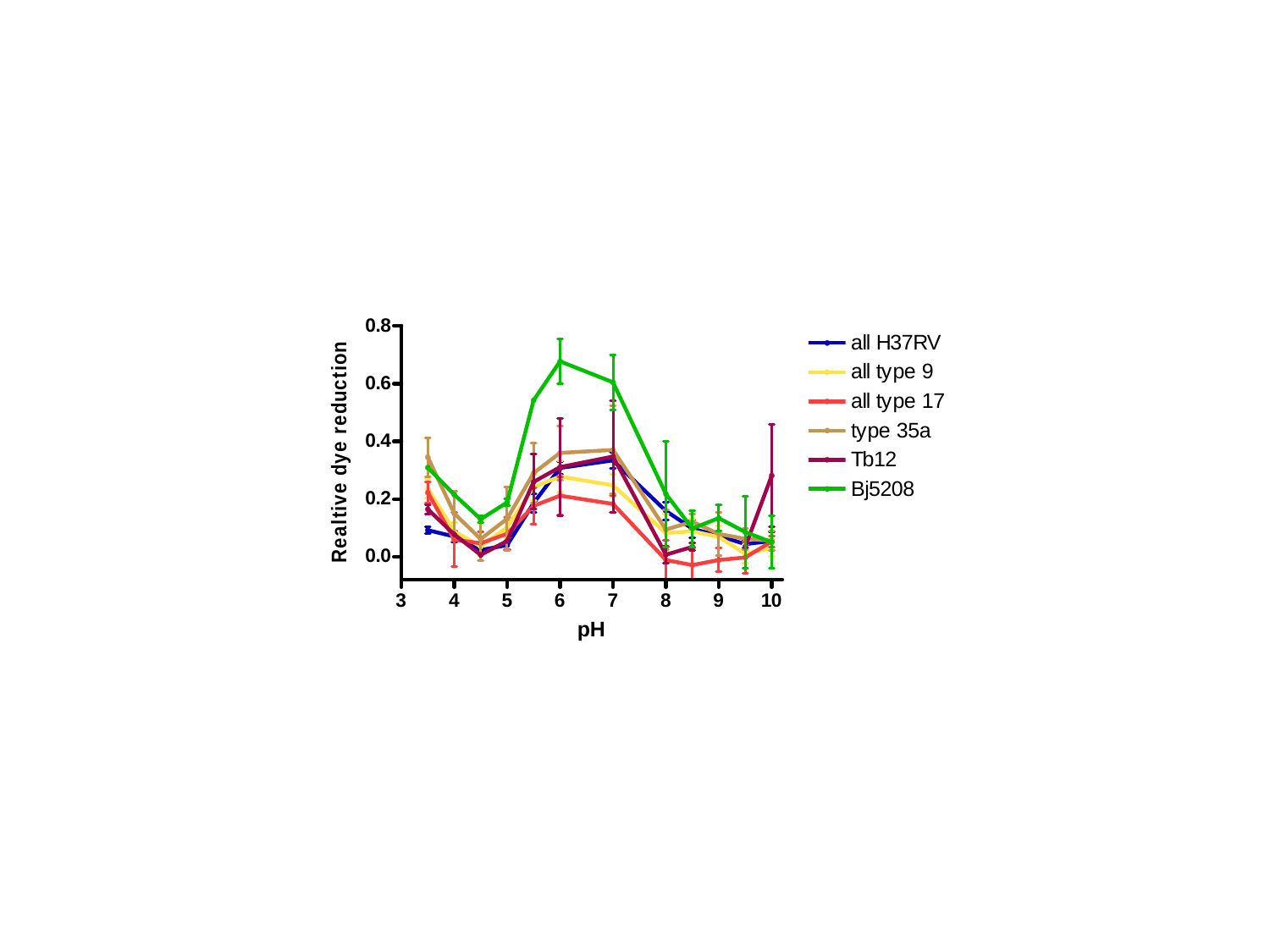

Supplement: Figure S7 — pH optima of strains. Mean ± SEM relative dye reduction values for 2 to 4 experiments are shown for each strain. (PPT) [file pone.0052673.s007.ppt]
