# Supplementary material for: High Throughput Phenotypic Analysis of Mycobacterium tuberculosis and Mycobacterium bovis Strains' Metabolism Using Biolog Phenotype Microarrays
Source: PLoS One. 2013 Jan 10;8(1):e52673. doi: 10.1371/journal.pone.0052673 (PMC3542357; doi:10.1371/journal.pone.0052673)
Supplement: Text S1 — Method development for preparing suspensions of the M. tuberculosis complex to inoculate PM plates (DOC) [file pone.0052673.s008.doc]

**Supplementary text file S1.**

**Method development for preparing suspensions of the *M. tuberculosis* complex to inoculate PM plates**

We made extensive changes in preparing the inoculum for PM plates to the method described- for *M. smegmatis-* in the company’s literature. Initially, strains were cultured on solid medium- Middlebrook 7H11 agar- as directed. Colonies of bacteria were collected and resuspended in phosphate-buffered saline (PBS) with added 0.025% (w/v) tyloxapol and mixed with IF-0a, but with the *M. tuberculosis* complex strains it proved difficult to prepare homogenous suspensions. They gave uneven dye production, high background and inconsistent readings: the reduced dye had a granular appearance around the clumps of bacteria. The time to obtain reasonable suspensions varied greatly between batches. Overall preculture on 7H11 was unsuitable as a reproducible methodology with *M. tuberculosis* complex strains. Therefore, bacteria were grown in a liquid medium, Middlebrook 7H9 including 0.05% Tween 80. The bacteria were washed by centrifugation and resuspension and finally suspended in PBS with added 0.025% (w/v) tyloxapol and IF-0a. These were homogenous suspensions but they gave high background dye reduction. The initial experiments were done with *M. bovis* BCG Pasteur and Russia strains grown with glycerol (0.2 % v/v) and background readings were in the A1 well of PM plates 1 to 4. In these plates the A1 well has no nutrient source while all the other 95 wells contain one potential nutrient source.

Therefore the next objective was to diminish background dye reduction. , i.e. focus on just one well of the 96 plus a few positive controls revealed in the PM1 plate. “Home made” plates (Nunc-immuno plates polystyrene) were made including wells with glycerol, pyruvate and Tween 80 as positive controls (each at 0.25 mM and 5 mM) and wells with no carbon source as negative controls to develop a protocol to diminish background. As well as being strongly positive in PM1 plates, the carbon sources chosen are classic growth substrates for *M. tuberculosis* and *M. bovis* BCG though glycerol does not support growth of *M. bovis* . The effects of using either Dye D or G, using or omitting IF-0a in the resuspending medium, and starvation at 25°C or 37°C for 24 or 48 h versus unstarved were tested in chequerboard design experiments. The optimum conditions, giving low background without carbon sources and colour development in wells with carbon sources, were to starve in PBS with added 0.025% (w/v) tyloxapol for 24 h at 25°C, suspend in IF-0a and use DyeG. The differentiation between background and positive well colour was even clearer when PM (PM1) plates were used in place of “home made” plates.

With *M. bovis* BCG strains, the Russia strain consistently gave stronger dye reduction across all plates than the Pasteur strain. Indeed, in some experiments out of four replicates, the Pasteur strain failed to give any dye reduction at all in some plates. As a result, it was decided in this study to investigate a wide range of strains to discover ones that gave good dye reduction in PM plates. In practice, nearly all the strains used (Table 1 in the main text) gave good dye reduction with differentiation between the A1 well and wells with substrates being clearer than when *M .bovis* BCG strains were used.

The original method for preparing suspensions to inoculate Phenotype MicroArray (PM) plates is described in the company’s literature describing a protocol for use with *M. smegmatis* (dated 28 January 2008). The current version, together with improvements in methodology that are available pre-publication can be obtained by contacting Barry Bochner, Biolog, Inc., Hayward, CA, USA, email: [bbochner@biolog.com](mailto:bbochner@biolog.com).). Method development summarised above is described in full in a MSc thesis (Khatri, B., 2010: Metabolic phenotype of bovine tuberculosis field strains. School of Life Sciences, Kingston University, London, UK),

**Reference:**

1. Keating LA, Wheeler PR, Mansoor H, Inwald JK, Dale J, et al. (2005) The pyruvate requirement of some members of the *Mycobacterium tuberculosis* complex is due to an inactive pyruvate kinase: implications for in vivo growth. Mol Microbiol 56: 163-174.
