# Supplementary material for: High Throughput Phenotypic Analysis of Mycobacterium tuberculosis and Mycobacterium bovis Strains' Metabolism Using Biolog Phenotype Microarrays
Source: PLoS One. 2013 Jan 10;8(1):e52673. doi: 10.1371/journal.pone.0052673 (PMC3542357; doi:10.1371/journal.pone.0052673)
Supplement: Text S2 — Wells with abiotic dye reduction (DOC) [file pone.0052673.s009.doc]

## Supplementary text file S2.

**Wells with abiotic dye reduction**

# PM1 - A2 (L-arabinose)

# B8 (D-xylose),

# C4 (D-ribose),

# H6 (L-lyxose)

PM2 - B5 (D-arabinose),

B9 (2-deoxy-D-ribose),

E5 (D-glucosamine),

E12 (5-keto-D-gluconic acid),

F9(sorbic acid)

H9 (dihydroxyacetone)

PM3 - E10 (D-mannosamine),

G4 (alloxan)

PM9 - C6 (NaCl 6% + glutathione)

### PM10- D10 (ethylene glycol 10%)
