# Supplementary material for: High Throughput Phenotypic Analysis of Mycobacterium tuberculosis and Mycobacterium bovis Strains' Metabolism Using Biolog Phenotype Microarrays
Source: PLoS One. 2013 Jan 10;8(1):e52673. doi: 10.1371/journal.pone.0052673 (PMC3542357; doi:10.1371/journal.pone.0052673)
Supplement: Table S2 — Preparation and concentration of PM additives for PM plates used in this study. (DOC) [file pone.0052673.s011.doc]

**Supplementary Table S2. Preparation and concentration of PM additives for PM plates used in this study.**

| **Component** | Final concn in well | | Stock solutions (g /100 ml) | **PM** | | **PM** | **PM** | **PM** |
| --- | --- | --- | --- | --- | --- | --- | --- | --- |
|  |  |  |  | **1 & 2** | | **3** | **4** | **9 & 10** |
| Tricarballylic acid, pH 7.1 | 20mM | | 14.088 | - | | 30ml | 30ml | - |
| MgCl_2_, 6H_2_O  Ca Cl_2_, 2 H_2_O | 2mM  1mM | | 4.88  1.76 | 10ml | | 10ml | 10ml | 10ml |
| L-arginine, HCl | 25μM | | 0.063 | 10ml | | - | 10ml | - |
| L-glutamic acid | 50μM | | 0.101 |  | |  |  |  |
| Sodium thiophosphate, 12H_2_O, pH 7.1 | 25μM | | 0.119 | 10ml | | 10ml | - | - |
| 5’-UMP, Na_2_ | 25μM | | 0.110 | 10ml | | 10ml | - | - |
| Hypoxanthine  β-NAD, Na | | 25μM  5μM | 0.041  0.040 | 10ml | | 10ml | 10ml | 10ml |
| Yeast extract  Bovine Serum Albumin  Middlebrook 7H9 broth | | 0.005%  0.02%  1:100 of 1x | 0.60  2.40  0.420 | 10ml | | 10ml | 10ml | 10ml |
| Tween 80 | 0.01% | | 1.2 | 10ml | | 10ml | 10ml | 10ml |
| D-glucose  Pyruvate, Na  L-malic acid | 2.5mM  5mM  5mM | | 5.4  6.6  8.045 | - | 10ml | | 10ml | - |
| Sterile distilled water |  | |  | 30ml | | - | 10ml | 50ml |
| Total |  | |  | 100ml | | 100ml | 100ml | 100ml |

Components were mixed from the stock solutions below, filter sterilised, and 1 part added as “PM additives” in 12 to other components to give the incubation mixture as described in the methods section of the full paper.
