# Supplementary material for: High Throughput Phenotypic Analysis of Mycobacterium tuberculosis and Mycobacterium bovis Strains' Metabolism Using Biolog Phenotype Microarrays
Source: PLoS One. 2013 Jan 10;8(1):e52673. doi: 10.1371/journal.pone.0052673 (PMC3542357; doi:10.1371/journal.pone.0052673)
Supplement: Table S5 — Roisin's medium. (DOC) [file pone.0052673.s014.doc]

**Supplementary Table S5. Roisin’s medium.**

For Roisin’s base, the carbons sources glycerol and tween 80 were omitted: if no Tweens were subsequently added to the base to give the modified medium, 0.25 ml tyloxapol/l was added.

| Chemical | Amount / L |
| --- | --- |
| KH_2_PO_4_ | 1.0 g |
| Na_2_HPO_4_ | 2.5 g |
| NH_4_Cl | 5.9 g |
| K_2_SO_4_ | 2.0 g |
| Trace element Solution | 1 ml |
| 1M CaCl2 | 0.5 ml |
| 1M MgCl2 | 0.5 ml |
| Glycerol | 5 ml |
| Tween 80 | 2 ml |
| Antifoam (fermenters) | 0.5 ml |
|  | |
| **1000 X Trace element Solution** | |
| Chemical | Amount / L |
| ZnCl_2_ | 80 mg |
| FeCl_3_-6H_2_0 | 400 mg |
| CuCl_2_-2H_2_O | 20 mg |
| MnCl_2_-4H_2_O | 20 mg |
| Na_2_B_4_O_7_-10H_2_O | 20 mg |
| (NH_4_)6Mo_7_O_2_4-4H_2_0 | 20 mg |

- Add each component in order to avoid precipitation of the salts to approximately 500 ml of milli Q water.
- When all components are added the pH should be approximately pH 6.9.
- pH with concentrated HCL to pH 6.6.
- Make the volume up to 1L.
- Leave in the fridge for several hours if antifoam has been added (antifoam will only go into solution at 4^0^C.
- Autoclave or filter sterilize.
